# The DevTox Germ Layer Reporter Platform: An Assay Adaptation of the Human Pluripotent Stem Cell Test

**DOI:** 10.3390/toxics10070392

**Published:** 2022-07-13

**Authors:** John T. Gamble, Kristen Hopperstad, Chad Deisenroth

**Affiliations:** 1 Center for Computational Toxicology and Exposure, Office of Research and Development, U.S. Environmental Protection Agency, Research Triangle Park, Durham, NC 27711, USA; gamble.john@epa.gov (J.T.G.); hopperstad.kristen@epa.gov (K.H.); 2 Oak Ridge Institute for Science and Education (ORISE), Oak Ridge, TN 37831, USA

**Keywords:** high-throughput screening, pluripotent stem cells, developmental toxicity, endoderm differentiation

## Abstract

Environmental chemical exposures are a contributing factor to birth defects affecting infant morbidity and mortality. The USA EPA is committed to developing new approach methods (NAMs) to detect chemical risks to susceptible populations, including pregnant women. NAM-based coverage for cellular mechanisms associated with early human development could enhance identification of potential developmental toxicants (DevTox) for new and existing data-poor chemicals. The human pluripotent stem cell test (hPST) is an in vitro test method for rapidly identifying potential human developmental toxicants that employs directed differentiation of embryonic stem cells to measure reductions in SOX17 biomarker expression and nuclear localization. The objective of this study was to expand on the hPST principles to develop a model platform (DevTox GLR) that utilizes the transgenic RUES2-GLR cell line expressing fluorescent reporter fusion protein biomarkers for SOX17 (endoderm marker), BRA (mesoderm marker), and SOX2 (ectoderm and pluripotency marker). Initial assay adaption to definitive endoderm (DevTox GLR-Endo) was performed to emulate the hPST SOX17 endpoint and enable comparative evaluation of concordant chemical effects. Assay duration was reduced to two days and screening throughput scaled to 384-well format for enhanced speed and efficiency. Assay performance for 66 chemicals derived from reference and training set data resulted in a balanced accuracy of 72% (79% sensitivity and 65% specificity). The DevTox GLR-Endo assay demonstrates successful adaptation of the hPST concept with increased throughput, shorter assay duration, and minimal endpoint processing. The DevTox GLR model platform expands the in vitro NAM toolbox to rapidly identify potential developmental hazards and mechanistically characterize toxicant effects on pathways and processes associated with early human development.

## 1. Introduction

Birth defects impact approximately 3% of births in the United States annually and are a major contributor to infant morbidity and mortality [1,2]. The majority of developmental anomalies are of unknown etiology but there is increasing evidence that exposure to certain environmental chemicals is a contributing factor [3]. Identifying prenatal developmental toxicants is challenging since target effects can span critical stages of fetal development (e.g., conception through organogenesis), resulting in adverse outcomes including low birth weight, congenital defects, functional deficits, and pregnancy loss [3]. Further complicating hazard identification are fetal–maternal interactions, where altered chemical toxicokinetic and toxicodynamic parameters can be ascribed to xenobiotic metabolism [4,5], placental transport functions [6,7,8], or general adverse effects on maternal physiology.

Regulatory decisions on chemical safety are currently reliant on animal testing to ascertain adverse reproductive and developmental toxicity [9]. Key provisions in the Frank R. Lautenberg Chemical Safety for the 21st Century Act, an amendment to the Toxic Substance Control Act (TSCA) of 1976, promote the use of non-animal, new approach methods (NAMs) to identify chemical risks to susceptible populations including infants, children, and pregnant women [10]. There is recognition that the reduction, refinement, and replacement of in vivo testing requires implementation of human in vitro NAMs to enhance testing capabilities for new and existing chemicals in commerce with limited safety data, reduce the time and cost of testing, and improve the mechanistic understanding and human relevance of developmental toxicity hazard identification [11,12,13]. The U.S. Environmental Protection Agency (U.S. EPA) has instituted strategic initiatives to reduce and replace animal testing in favor of in vitro data and in silico models for NAM-based chemical safety assessments [14,15,16]. The U.S. EPA currently employs high-throughput screening approaches to identify environmental chemicals that may perturb key biological processes generally related to cellular growth, differentiation, and development, but the scope of chemicals that perturb key mechanistic events during pregnancy and fetal development is less clear [17,18,19,20]. Continued development and application of human in vitro NAMs that recapitulate defined endpoints of early embryonic development are necessary to address these challenges [13].

Pluripotent stem cells (PSCs), whether derived from an embryo or reprogrammed from a somatic cell [21], are capable of differentiating in vitro into each of the three germ layer lineages (ectoderm, mesoderm and endoderm) that develop during gastrulation, providing a suitable model of early embryonic differentiation to investigate adverse chemical effects [22,23]. Early embryogenesis is primarily controlled by five evolutionarily conserved molecular signaling pathways: Hedgehog, transforming growth factor β (TGF-β), Notch, Wingless-Int (Wnt), and receptor tyrosine kinase (RTK) pathways [12,24]. Tight coordination and regulation across these signaling nodes are essential for maintaining the self-organizing principles that culminate in stem cell differentiation to myriad cell types and tissue lineages [25,26]. Depending on the cell culture model and conditions, germ layer differentiation can arise spontaneously (e.g., embryoid bodies) or via directed differentiation to specific lineages [27].

The mouse Embryonic Stem Cell Test (mEST) became the first validated cell-based method for identifying developmental toxicants [28]. The assay evaluates the ability of a compound to inhibit nondirected myocardial differentiation in three-dimensional embryoid bodies and has validated bioactivity of a diverse set of pharmaceutical and environmental chemicals with a predictive accuracy of 75–78% [28,29]. Modifications and alternatives to the mEST have been designed to increase testing throughput, decrease assay duration, add mechanistic insight, enhance human relevance, improve applicability domains and predictive accuracy, and avoid noted limitations with qualitative scoring of contracting cardiomyocytes. Modifications include molecular fluorescence-activated cell sorting (FACS)-EST which utilizes quantitative flow cytometry to detect expression of α-actinin and myosin heavy chain (MHC) biomarkers indicative of perturbed cardiomyocyte differentiation and embryotoxic potential [30], the adherent cell differentiation and cytotoxicity (ACDC) assay which uses an In-Cell Western technique to quantify MHC protein levels and count cells for concurrent viability assessment [31,32], stable transgenic *Hand1* and *Cyma1* embryonic stem cell (ESC) lines that monitor luciferase reporter activity for cardiomyocyte differentiation [33,34], and the ReProGlo assay that utilizes transient transfection of a Wnt signaling pathway reporter in mouse ESCs to rapidly identify chemical effects [35]. Application of gene expression panels as a surrogate to phenotypic measurements shortens the assay duration and identifies perturbations to cardiomyocyte differentiation with, in some cases, a similar degree of accuracy as the mEST [36,37,38]. Alternative models include replacement of murine cell lines with human PSCs (hPSCs) for rapid, non-targeted [39] and targeted [40] metabolomics approaches with accuracies reaching 88 and 77%, respectively. Increasing model system complexity in pursuit of enhanced biological relevance has brought about biomimetic models that recapitulate spatiotemporal and morphological features of gastrulation including axial elongation and germ layer patterning at the expense of screening throughput [41,42,43,44,45,46].

The human pluripotent stem cell test (hPST) utilizes directed endoderm differentiation of the H9 human ESC line to identify developmental toxicants that perturb SRY-box transcription factor 17 (SOX17) expression and nuclear localization [47]. During gastrulation, SOX17 is a key endodermal biomarker regulated by NODAL/WNT signaling which aids in establishing endoderm differentiation in conjunction with other transcription factors (e.g., EOMES and FOXA2) [48]. SOX17 is essential to formation of the primitive gut tube which gives rise to the associated organs of the respiratory and digestive tracts during organogenesis [49,50]. Evaluation of the hPST predictive performance against 71 drug-like compounds revealed a balanced accuracy of 94% (97% sensitivity, 92% specificity) when filtered by a potency threshold of 30 µM. Likewise, a panel of 15 environmental toxicants with diverse physicochemical properties exhibited strong concordance to developmental toxic outcomes documented from in vivo guideline studies [47]. The human relevance and high performance of the hPST single-biomarker approach make it an ideal candidate assay to provide broader coverage of new and existing data-poor chemicals while maintaining appreciable predictive accuracy.

The objective of this study was to adapt the principles of the hPST assay for increased throughput, decreased assay duration, and broader mechanistic evaluation. The RUES2-GLR (germ layer reporter) human PSC line [51] supplanted H9 ESCs to expand the biomarker profile to all three gastrulation lineages, thereby establishing the multi-lineage DevTox GLR model platform. The RUES2-GLR cell line has been transgenically modified by CRISPR-Cas9 technology to endogenously express fluorescent reporter fusion protein biomarkers from native gene loci for SOX17 (endoderm marker), Brachyury [BRA] (mesoderm marker), and SRY-box transcription factor 2 [SOX2] (ectoderm and pluripotency marker). The cell line enables SOX17, BRA, and SOX2 protein expression evaluation without the need for fluorescent antibodies and provides additional mechanistic insight into pluripotency and differentiation pathways. Initial assessment focused on directed differentiation to definitive endoderm (DevTox GLR-Endo assay mode) to enable comparison to the hPST with a common benchmark chemical set. For enhanced throughput and screening efficiency, the assay was scaled to a 384-well format and endoderm differentiation time reduced to two days. Assay feasibility was determined with four reference developmental toxicants and four non-developmental toxicants. For evaluation of assay performance, a training set of 58 chemicals was screened to determine sensitivity, specificity, and balanced accuracy. Comparative analysis of assay performance was conducted against existing hPSC developmental toxicity assays (hPST and devTOX *quick*Predict) [47,52]. This study adapted principles of the hPST to establish the multi-lineage DevTox GLR model platform that successfully recapitulated chemical effects during directed endoderm differentiation (DevTox GLR-Endo assay mode).

## 2. Materials and Methods

### 2.1. RUES2-GLR Pluripotent Stem Cell Culture Maintenance

RUES2-GLR (Rockefeller University Embryonic Stem cell line 2-Germ Layer Reporter) cells have been transgenically modified from the RUES2 cell line (NIH registration number 0013; approval number NIHhESC-09-0013) to endogenously express fluorescent reporter fusion protein biomarkers: SOX17–tdTomato (endoderm), BRA–mCerulean (mesoderm), and SOX2–mCitrine (ectoderm/pluripotency) [51]. RUES2-GLR cells were maintained with feeder-free complete Essential 8 Flex medium (Gibco, Waltham, MA, USA) using manufacturer’s recommendations on vitronectin (Gibco, Waltham, MA, USA)-coated (0.5 µg/cm^2^) Costar Clear 6-well (9.5 cm^2^/well), non-TC-treated plates (Corning, Corning, NY, USA). Medium was changed every one to two days for maintenance and sub-cultured every three to five days. Cultures were passaged in colonies at 70–85% confluence using non-enzymatic cell dissociation with Versene EDTA solution (0.48 mM) (Life Technologies, Carlsbad, CA, USA). All experiments were conducted with cells from passage 2–12 with >75% post-harvest viability. RUES2-GLR pluripotency was visually inspected on a regular basis for morphological consistency with phenotypically distinct populations maintained at <15%. Cultures were routinely tested for mycoplasma contamination using MycoAlert Detection Kit (Lonza, Morristown, NJ, USA). All cell incubations were performed at 37 °C in a humidified atmosphere of 5% CO_2_ in air.

### 2.2. TaqMan hPSC Scorecard Gene Expression Panel

**Figure 1:**
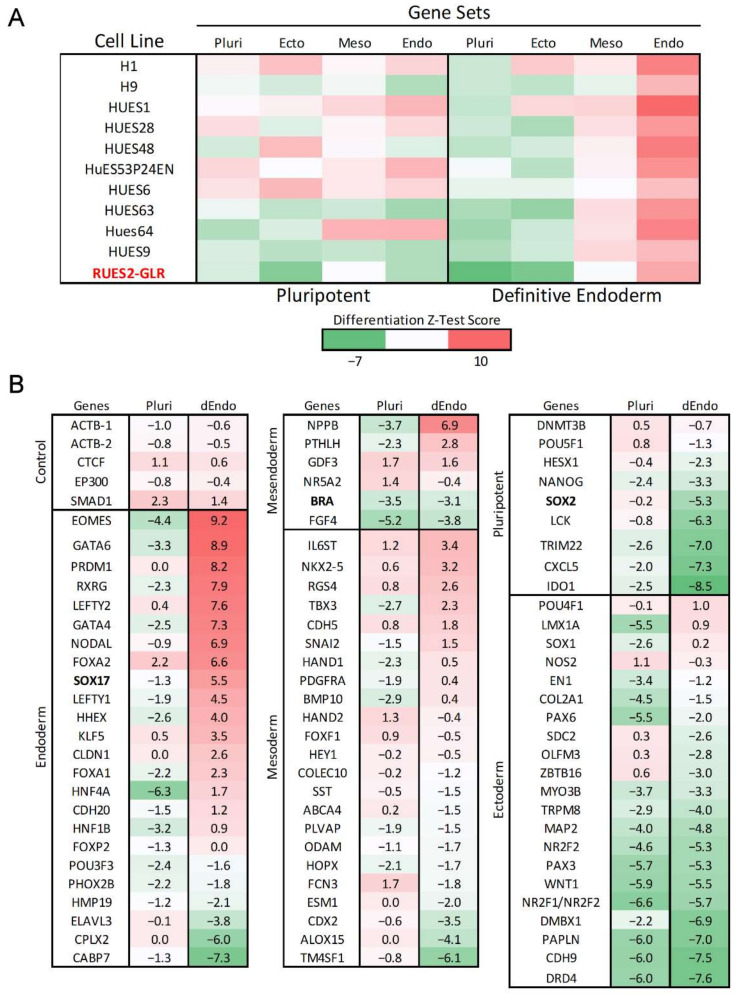
Gene expression of RUES2-GLR directed endoderm differentiation. Gene expression of RUES2-GLR at 48 h (Red) in pluripotent and directed endoderm states using the hPSC Taqman Scorecard array. (**A**) Heatmap of Z-test scores calculated from aggregate hPSC Scorecard gene data for multiple human pluripotent stem cell lines in pluripotent and directed endoderm conditions classified by differentiation state. All scores were compiled from published data [54] except RUES2-GLR which was generated for comparison to other cell lines. Pluripotent (Pluri), Ectoderm (Ecto), Mesoderm (Meso), Endoderm (Endo). (**B**) hPSC Scorecard log2 fold gene expression changes compared to published data of ten pluripotent stem cell lines for genes of each differentiation state (*n* = 3). Colorimetric scale indicates high (red), neutral (white), and low (green) gene expression. Directed Endoderm (dEndo).

To evaluate gene expression in the RUES2-GLR cell line, cells were seeded at day 0 into vitronectin-coated (0.5 µg/cm^2^) 6-well plates. StemPro Accutase cell dissociation reagent (Gibco, Waltham, MA, USA) was used to harvest cell cultures at 70–85% confluence to generate a single-cell suspension in complete Essential 8 Flex medium with 1× RevitaCell ROCK inhibitor (Gibco, Waltham, MA, USA). Cells were seeded at 2.0 × 10^5^ cells/well. For directed endoderm differentiation, induction was conducted with the PSC Definitive Endoderm Induction kit (Gibco, Waltham, MA, USA) according to the manufacturer’s protocol. Briefly, after 24 h post seeding, spent medium was aspirated and 2 mL of room temperature Medium A was added. After an additional 24 h, spent Medium A was aspirated and 2 mL of Medium B was added. The pluripotent control populations were maintained in Essential 8 Flex medium. Cells were harvested 72 h post-seeding with Accutase and centrifuged at 250× *g* for five minutes. The supernatant was removed and the mRNA from the cell pellet was purified using the RNeasy Mini Purification Kit (Qiagen, Hilden, DEU) according to manufacturer’s protocol. RNA quantification and integrity were measured using a Nanodrop 1000 (ThermoFisher Scientific, Waltham, MA, USA) with RNA quality observed at 2.0 ± 0.1 for the 260/280 nm absorbance ratio. TaqMan hPSC Scorecard Panels (Applied Biosystems, Waltham, MA, USA) were run according to manufacturer’s protocol (Applied Biosystems, Waltham, MA, USA). Using the High-Capacity cDNA Reverse Transcription Kit (Applied Biosystems, Waltham, MA, USA), 1 µg of RNA was combined with reverse transcription reagents and aliquoted into eight PCR tubes with 50 µL reaction mix. Following reverse transcription, reactions were diluted with water, mixed with qRT-PCR master mix, and 10 µL dispensed into each well of a sample quadrant. One aliquot was used across 12 wells. Samples were analyzed by qRT-PCR using a QuantStudio 7 Flex (Applied Biosystems, Waltham, MA, USA) with the following protocol: hold 2 min at 50 °C, hold 10 min at 95 °C, 40 cycles of 95 °C for 15 s, and 60 °C for 1 min. The qRT-PCR reaction Ct values were calculated using QuantStudio auto-thresholding for each well and ΔCt values determined by the change relative to the mean threshold cycle (Ct) value of five housekeeping genes (ACTB-1, ACTB-2, CTCF, EP300, SMAD1) used in the design of the Scorecard array [51]. Changes in gene expression were evaluated using the 2^−ΔΔCt^ method [53] where fold changes represent changes in expression compared to the average ΔCt values for 10 pluripotent stem cell lines maintained at pluripotency [54]. Z-test scores were calculated by comparing the gene expression to the same datasets (Figure 1A) using a Z-test algorithm [54]. Briefly, *p*-values were determined by a one-sample, one-sided t-test comparing the ΔCt values to the reference set of ΔCt values for each gene expressed in a pluripotent state. The *p*-values were aggregated together based on a classifier gene set (pluripotency, ectoderm, mesoderm, endoderm) using the Z-test formula,

(1) $$1-\Phi \;\left(\frac{\sum_{k=1}^{N}Z_{k}}{\sqrt{2\sum_{k<j}r_{k,j}}}\right)$$

To produce a differentiation state Z-test score, where $Z_{k}=\Phi {\;}^{-1}\left(1-P_{k}\right)$, ***P_k_*** is the *p* value for the ***k***-th gene set that contains N genes, **Φ** and **Φ**−**1** denote the standard normal cumulative distribution function and respective inverse, and ***r_k,j_*** is the correlation of ***Z_k_*** and ***Z_j_***. Differentiation state Z-test scores are interpreted as differentiation state gene set activity relative to an average human pluripotent stem cell state with higher scores indicating more genes exhibiting significantly increased expression than the average pluripotent state.

### 2.3. Test Chemical Selection and Preparation

Positive reference chemicals (all-trans-retinoic acid, lenalidomide, pomalidomide, thalidomide) were selected based on FDA pregnancy category X and demonstrated capacity to decrease in vitro SOX17 expression in potency ranges not confounded by loss of cell viability (Table 1 and Table S1) [47,55,56]. Negative reference chemicals (aspirin, caffeine, folic acid, and saccharin) were selected based on FDA pregnancy category (A, B, or C) and were predicted negative for developmental toxicity based on the inability to decrease in vitro SOX17 expression (Table 1 and Table S1) [47]. To evaluate performance relative to prior assay predictions, a training set of 58 chemicals (Table S1) were selected based on reported bioactivity in the hPST and devTOX *quick*Predict^TM^ (Stemina Biomarker Discovery, Madison, WI, USA) assays and classified as either positive or negative for developmental toxicity [47,52]. Bisphenol A was reclassified as a developmental toxicant [57,58,59] despite being classified as non-developmental toxicant for the *quick*Predict benchmark chemical set [52]. Transforming growth factor ß (TGF-ß) receptor inhibitor SB431542 (Tocris, Bristol, UK) was used as a developmental toxicant-positive control to verify successful inhibition of endoderm differentiation [47,60]. Chemicals were solubilized in dimethyl sulfoxide (DMSO) at a concentration of 100 mM or up to the limit of solubility (Table S1). Chemical source plates were prepared with Echo-qualified 384-well polypropylene 2.0 plates (Labcyte, San Jose, CA, USA). Parent source plates containing the top solubilized concentration were used to generate lower concentration daughter plates with an Echo 555 acoustic dispenser (Labcyte, San Jose, CA, USA). All chemical source plates were stored at −80 °C. For screening, source plates were thawed at room temperature (20–24 °C) for two days, mixed on a Multi-Microplate Genie mixer (Scientific Industries, Bohemia, NY, USA) at 700 rpm for 10 min prior to each experimental run, and stored, protected from light, in a desiccator throughout the duration of use.

### 2.4. DevTox GLR Endoderm Differentiation Assay (DevTox GLR-Endo)

RUES2-GLR cells were seeded 24 h prior to differentiation initiation into vitronectin-coated 384-well CellCarrier Ultra plates (10.6 mm^2^/well) (Perkin Elmer, Waltham, MA, USA). Vitronectin (0.5 µg/cm^2^)-coated plates were prepared and stored at 4 °C, wrapped in parafilm, for less than seven days and incubated at room temperature for one hour prior to cell seeding. At 70–85% confluence, Accutase was used to generate a single-cell suspension in complete Essential 8 Flex medium with 1× RevitaCell ROCK inhibitor. Cells were dispensed into 20 µL coating solution using a Certus FLEX Micro Dispenser (Fritz Gyger, Gwatt, Switzerland) with a 0.45 µm valve to a final volume of 100 µL and density of 2.8 × 10^3^ cells per well.

Following a 24 h post-seeding incubation period (Time = 0 h) (Figure 3A), directed endoderm differentiation was initiated with the PSC Definitive Endoderm Induction kit (Gibco, Waltham, MA, USA). For test chemical exposures, compounds were pre-dispensed into cell-free, sterile, white flat-bottom 384-well µClear plates (Greiner Bio-One, Kremsmünster, Austria) with an Echo 555 acoustic dispenser. A target dilution series (0.002–200 µM) was generated by dispensing variable nanoliter volumes of test chemical and backfilling with a complementary volume of DMSO using Echo Cherry Pick software (v.1.6.2). Wells were backfilled with 50 µL of induction medium using a Certus FLEX Micro Dispenser with 0.45 µm valves. Test compound treatment plates were mixed on a Multi-Microplate Genie mixer at 700 rpm for 10 min and subsequently incubated for two hours in a humidified atmosphere at 37 °C with 5% CO_2_. Each chemical treatment (0 h–Medium A; 24 h–Medium B) were prepared the day of treatment. At 0 h, cell culture medium was aspirated from the assay plate at a rate of 1 µL/s and 40 µL of Medium A ± test chemical applied using a CyBio FeliX automated liquid handler (Analytik Jena, Jena, Germany). At 24 h, conditioned cell culture medium was aspirated and 40 µL of Medium B ± test chemical was applied. At 48 h, cells were prepared for high-content image acquisition by fixation and nuclear staining. The total test chemical incubation period was 48 h. For fixation, a 4% paraformaldehyde (PFA) solution was achieved by adding 40 µL of 8% PFA directly to cell culture medium using a CyBio FeliX liquid handler. Following a 15 min incubation period at room temperature, fixative solution was aspirated and 20 µL of 1× HCS NuclearMask Deep Red Stain (Invitrogen, Waltham, MA, USA) in Dulbecco’s phosphate-buffered saline (DPBS) solution was dispensed. Following an additional 30 min incubation period at room temperature, staining solution was aspirated, 50 µL of DPBS was added, and plate sealed with MicroAmp Optical Adhesive Film (Applied Biosystems, Waltham, MA, USA).

Assay plate controls included non-differentiated pluripotent cells maintained in complete Essential 8 Flex medium (baseline control), endoderm differentiated cells (differentiation control), endoderm differentiated cells treated with DMSO (0.2%) (solvent control), and TGF-ß receptor inhibitor SB431542 (1 µM)-treated cells (developmental toxicant-positive control) (Figure S3). All experiments were completed with a minimum of four biological replicates (*n* = 4).

### 2.5. Image Acquisition and Analysis

Images were acquired within 12 h post-fixation using an Opera Phenix High-Content Screening System (Perkin Elmer, Waltham, MA, USA). Brightfield and fluorescent images for biomarkers BRA (mCerulean; ex 433/em 475), SOX2 (mCitrine; ex 516/em 529), SOX17 (tdTomato; ex 554/em 581) and nuclear stain (HCS NuclearMask Deep Red Stain; ex 636/em 685) were acquired from five fields per well with a 20× water immersion objective using 2 × 2-pixel binning. Cell identification and mean biomarker fluorescent intensities were calculated using Harmony image analysis software (Perkin Elmer, Waltham, MA, USA). Cell nuclei were identified using the object classifying algorithm C of the software with border objects excluded using the “Remove Border Objects” feature. Mean cell biomarker intensity statistics were gathered from each object and exported for further analysis using a custom script in Python version 3.7.4 [61] (Supplementary Materials). For each plate, pluripotent-negative control wells were used to establish baseline intensity values and a cutoff threshold for determining SOX17 and BRA biomarker-positive cells. Endoderm differentiated positive control wells were used to establish baseline intensity values and a cutoff threshold for SOX2-positive cells. Cells with a mean intensity five times the baseline median absolute deviation (5*bmad) above the median cell intensity for plate-based control population technical replicates were considered positive. Well-based cell counts, reflected by the sum of total counted objects from all fields, were used to calculate the percent responders for each respective biomarker and evaluate cytotoxicity. Well-level results were exported for data modeling in the ToxCast Pipeline. Additional graphs were produced using Matplotlib version 3.1.1 in Python [62].

### 2.6. ToxCast Pipeline Analysis

The ToxCast Pipeline R package (tcpl v.2.0.2) was used to normalize, fit, and qualify concentration-response screening data [63]. Raw data were received and loaded into the ToxCast database, invitrodb (to be released in version 3.5, expected Summer 2022) as the following assay endpoints (Table 2) under the Center for Computational Toxicology and Exposure assay source identifier, abbreviated as CCTE:

Well-level raw data values (rval) were obtained as either the percentage of SOX17-, SOX2-, or BRA-positive cells (endoderm assay) or total cell counts (cytotoxicity assay). For the SOX17 and total cell count assay endpoints, rvals were normalized as response percent activity relative to DMSO solvent control using the following equation,

(2) $$resp=100\frac{rval-bval}{bval}$$

where ***resp*** is the normalized response to be fit and bval is the median value of DMSO control wells on the same plate. The **resp** values for SOX17-positive and cell count-positive (AEIDs 3093 and 3097) endpoints were multiplied by negative one to invert data in the positive direction. The SOX2 and BRA assay rvals (AEIDs 3095 and 3096) required no additional normalization due to minimal changes in expression resulting from directed endoderm differentiation.

Efficacy cutoffs (coff) were defined as three times baseline median absolute deviation (3*bmad), where the baseline median absolute deviation (bmad) was calculated using the responses at the DMSO solvent control wells across each endpoint. If the maximum median value was greater than or equal to the coff, the sample was considered active with a positive hit call (hitc = 1). Cautionary flags are also assigned to identify anomalies with curve fits (e.g., noise, borderline hit, and borderline miss).

### 2.7. Assay Performance Evaluation

Percent SOX17-positive intra- and inter-plate assay performance metrics were calculated based on results from training chemical experiments, encompassing 12 technical replicates per control with 12 plates total. Robust metrics were calculated as follows [64,65]:

(3) $$Signal\;to\;Background\;ratio\left(S/B\right)=\frac{x_{Pos}}{x_{Neg}}$$

(4) $$Robust\;Coeffecient\;of\;Variation\;\left(rCV\right)=\frac{x_{Pos}}{MAD_{Pos}}*100$$

(5) $$Robust\;Z^{\prime }-factor\;\left(rZ^{\prime }\right)=\frac{\left(3mad_{Pos}+3mad\right)}{\left|x_{Pos}-x_{Neg}\right|}$$

where ***x*** represents the median percent SOX17-positive cell value of the control wells, mad is the median absolute deviation of the control wells, Pos is the positive control of either the 0.2% DMSO solvent exposed directed endoderm control or the directed endoderm control and Neg is the non-differentiated pluripotent control. The inter-plate rCV calculation is the median of all plate-based rCV values.

### 2.8. Comparative Analysis of Assay Predictive Performance

Chemical response comparisons between the DevTox GLR-Endo and the hPST and devTOX *quick*Predict assays were done using reported potency values and hit determination calls reported previously [47,52].

## 3. Results

### 3.1. Directed Endoderm Differentiation with the RUES2-GLR Cell Line

The human pluripotent stem cell test (hPST) measures perturbations to cellular SOX17 expression and nuclear localization by fluorescent immunocytochemistry following a three-day endoderm induction period of pluripotent stem cells [47]. To provide additional mechanistic data across gastrulation lineages, the RUES2-GLR human embryonic stem cell line was adapted to the hPST in place of H9 embryonic stem cells. The RUES2-GLR cell line has been transgenically modified to express fluorescent fusion proteins: SOX17–tdTomato (endoderm marker), BRA–mCerulean (mesoderm marker), and SOX2–mCitrine (ectoderm/pluripotency marker) [51]. Of note, SOX17 and BRA fusion proteins feature a self-cleaving peptide sequence that separates the native protein from the fluorescent moiety, whereas SOX2 protein remains fused to the fluorescent moiety. As a result, there is little concern for haploinsufficiency or loss of function of the endogenous genes. 

To evaluate baseline differentiation potential, RUES2-GLR cells were induced towards definitive endoderm over the course of 48 h. Endoderm differentiation efficiency was evaluated using the Taqman hPSC Scorecard gene expression panel that contains 96 gene markers selected for unique expression profiles that classify pluripotency and trilineage differentiation potential across ectoderm, mesoderm, and endoderm lineages [54]. RUES2-GLR expression values were compared to published hPSC Scorecard datasets from 10 other pluripotent stem cell lines by using a Z-test algorithm that aggregates significant gene expression changes by ectoderm, mesoderm, endoderm, and pluripotency states [54]. As expected, RUES2-GLR displayed the greatest increase in endodermal gene markers compared to other differentiation states with a substantial decrease in pluripotent gene expression relative to cells maintained under pluripotent conditions (Figure 1A). Z-test scores demonstrated similar germ layer lineage and pluripotency states under both pluripotent and directed endoderm conditions, consistent with H9 and other pluripotent stem cell lines (Figure 1A). Key endodermal markers EOMES, GATA6, and NODAL exhibited log2 fold-change values of 9.2, 8.9, and 6.9, respectively, following endoderm induction (Figure 1B). Log2 fold-change values for RUES2-GLR biomarkers SOX2, BRA and SOX17 were −5.3, −3.1, and 5.5, respectively (Figure 1B).

### 3.2. DevTox GLR-Endo Assay Workflow Optimization

To increase screening throughput, the assay was adapted from a 96- to a 384-well format. Since stem cell seeding density can affect cellular differentiation efficiency, four densities of 1.4 × 10^3^, 2.1 × 10^3^, 2.8 × 10^3^ and 3.5 × 10^3^ cells per well were tested for SOX17-dependent endoderm induction efficiency [66,67,68,69]. The percentage of SOX17-positive cells increased with higher seeding densities up to 2.8 × 10^3^ cells. Since no significant increase in SOX17-positive cells was observed at the two highest densities, a final well seeding density of 2.8 × 10^3^ cells was selected for the assay in a 384-well format (Figure S1).

**Figure 2:**
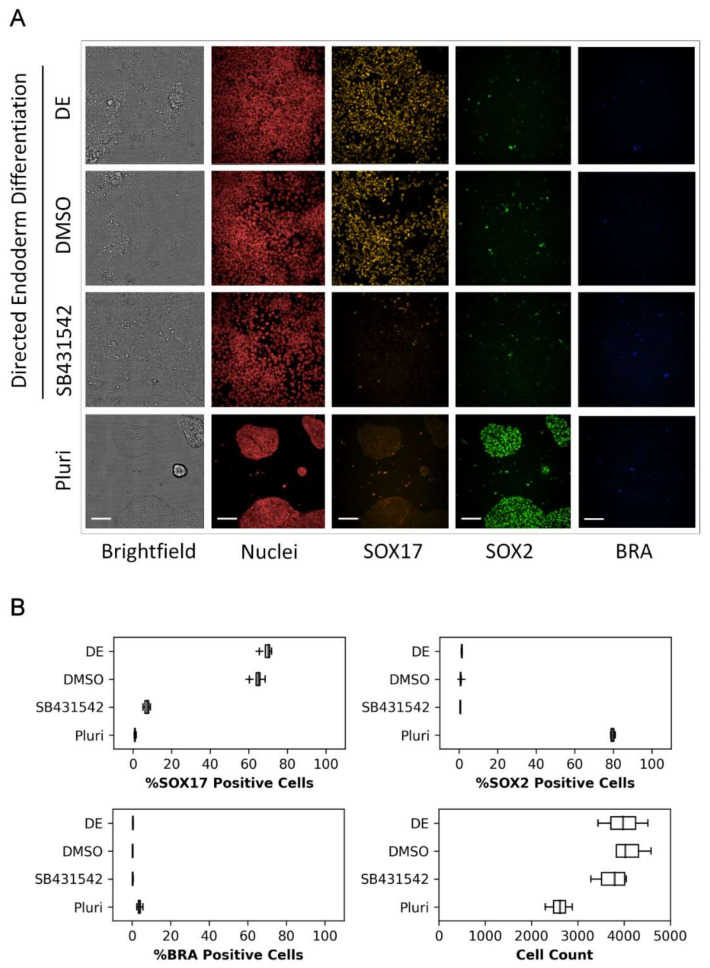
Representative RUES2-GLR biomarker expression images and data. RUES2-GLR cells represented across four conditions: directed endoderm (DE), directed endoderm with solvent control (DMSO), directed endoderm with TGF-ß receptor inhibitor control (SB431542), and pluripotent (Pluri). (**A**) Confocal images of cells displaying biomarkers SOX17 (yellow), SOX2 (green), BRA (blue), nuclear stain (red) and brightfield; 200 µm scale bar. (**B**) Quantitative % responder and cell count data across the four conditions. Box and whisker plots: center line-median, box-first and third quartiles, whiskers-1.5*inter-quartile range, + outliers; (*n* = 4).

To identify biomarker-positive populations during directed endoderm differentiation, RUES2-GLR cells were evaluated by high-content confocal imaging. Following 48 h of endoderm induction, clear morphological changes were observed with reduced colony formation and compactness, and a mesenchymal phenotype exhibiting enhanced spreading (Figure 2A). A qualitative loss of SOX2 expression and increased SOX17 expression were evident, indicating loss in pluripotency and commitment to definitive endoderm, respectively (Figure 2A). Quantitative image analysis revealed that the majority of cells were SOX17 positive (70.2 ± 2.8%) and BRA negative (0.45 ± 0.1%), further supporting an endodermal phenotype (Figure 2B). The addition of SB431542, a TGF-ß type 1 receptor inhibitor that inhibits activation of the Activin/Nodal signaling pathway, resulted in loss of pluripotency and endoderm differentiation, evident with loss of both SOX2 and SOX17, respectively. DMSO solvent exposure (0.2%) reduced the SOX17 population by 19.5% without affecting cell counts (Figure S2) but did not affect SOX2 and BRA expression, or the mesenchymal phenotype (data not shown). Despite the solvent-induced reduction in the SOX17-positive population, the assay endpoint demonstrated appreciable dynamic range with and without solvent exposure. These data indicate the assay maintained a dynamic range suitable for high-throughput screening with low technical and experimental variability. 

### 3.3. Reference Chemical Testing

**Figure 3:**
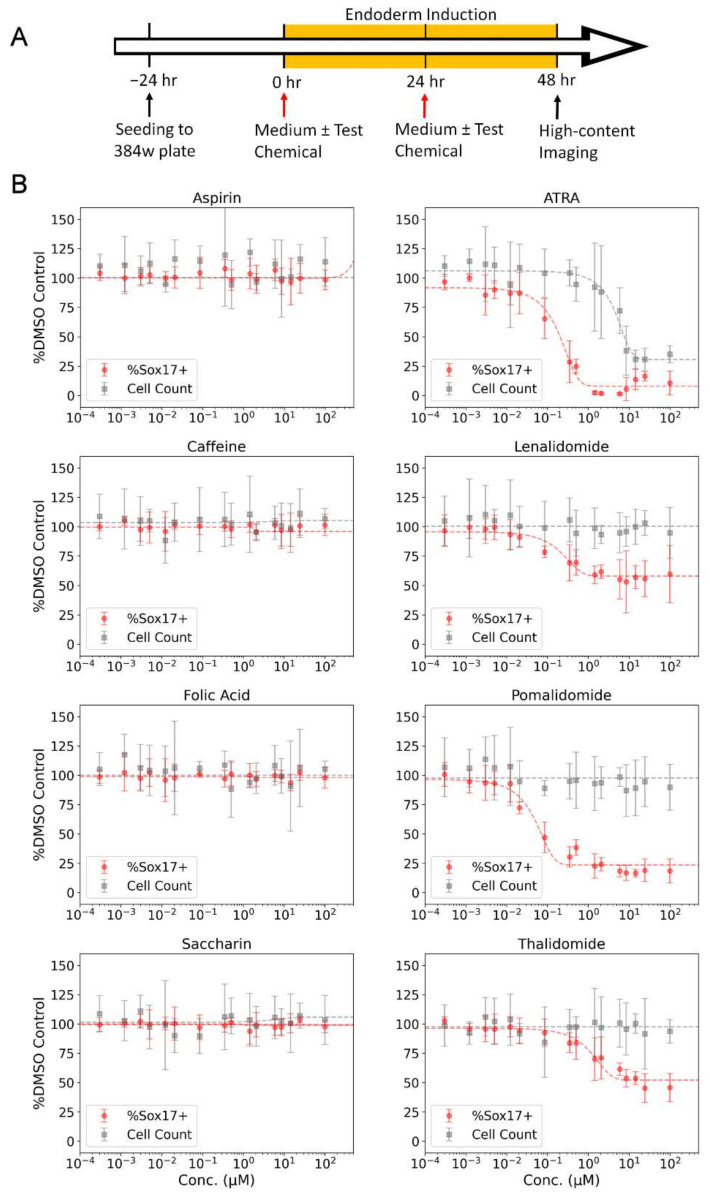
Reference chemical testing in the DevTox GLR-Endo assay. (**A**) Timeline for DevTox GLR-Endo assay. RUES2-GLR cells were exposed to reference chemicals for 48 h during directed endoderm differentiation. (**B**) Negative reference chemicals (aspirin, caffeine, folic acid, and saccharin) are on the left column, positive reference chemicals (all-trans retinoic acid (ATRA), lenalidomide, pomalidomide, thalidomide) are on the right column. Gray points display average cells counted per well (cell count) and red points display the average percentage of SOX17-positive cells per well (%SOX17+). Dashed lines represent regression curve fits. Error bars-2*bmad; (*n* = 8).

Initial evaluation of the DevTox GLR-Endo assay and semi-automated workflow was conducted with a set of eight reference chemicals to determine assay performance (Table 1). Positive chemicals (all-trans-retinoic acid, thalidomide, lenalidomide, pomalidomide) are classified as FDA category X chemicals with demonstrated fetal abnormalities during human development and have been reported to decrease endoderm differentiation in the hPST assay [47,55]. Negative chemicals (aspirin, caffeine, folic acid, and saccharin) were included since they have tested negative in other cell-based developmental toxicity assays [28,47,52,70,71]. Chemical exposure concentration ranged from 1 pM to 200 µM, depending on chemical solubility in DMSO (Table S1). Following a 48 h exposure period with two consecutive test chemical treatments at 24 h intervals, the cells were fixed, nuclei stained, and imaged by high-content fluorescent microscopy (Figure 3A). The assay correctly identified anticipated bioactivity for all eight chemicals where a decrease in the SOX17-positive population indicated potential for developmental toxicity independent of cytotoxicity (Figure 3B). Thalidomide, lenalidomide, and pomalidomide had significant reductions in the SOX17-positive sub-population while cell counts remained constant as chemical concentrations increased. The SOX17 IC_50_ potency values were 0.05, 0.13, 0.16 and 1.71 µM for pomalidomide, lenalidomide, all-trans-retinoic acid and thalidomide, respectively (Table 1). All-trans-retinoic acid displayed the largest effect size with 93% maximum inhibition, followed by pomalidomide (81%), thalidomide (58%) and lenalidomide (44%). Negative chemicals did not alter SOX17-positive cells at any concentration relative to solvent control (Figure 3B). 

### 3.4. Chemical Training Set Evaluation

**Figure 4:**
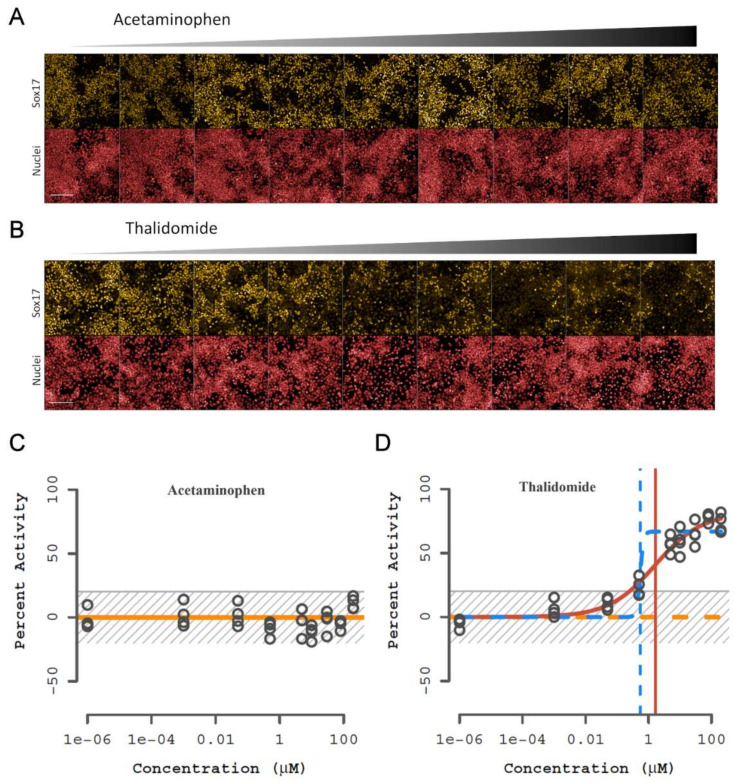
Representative images and curve fit plots from the chemical training set. Representative non-developmental toxicant (acetaminophen) and developmental toxicant (thalidomide) results. (**A**,**B**) Fluorescent images of SOX17 (yellow) expression and nuclei (red) with increasing concentration of chemical. RUES2-GLR cells were exposed to chemical during endoderm differentiation for 48 h; 1 mm scale bar. (**C**,**D**) Percentage activity relative to control for SOX17-positive cells. Horizonal lines represent different curve fits with winning model in solid and vertical lines indicate concentration at 50% activity; yellow—constant, orange—Hill, blue—gain-loss. The gray striped box is the baseline and noise band (0 ± 3*bmad); (*n* = 4).

To test the DevTox GLR-Endo assay predictive performance, a larger set of 58 positive and negative developmental toxicants identified from benchmark sets used to evaluate the hPST and devTOX *quick*Predict assays were selected [47,52] (Table S1). Chemicals from the hPST benchmark set were selected to enable direct comparison of SOX17-dependent changes in the hPST, while chemicals from the devTOX *quick*Predict benchmark set comprised a more general set of compounds used to evaluate other in vitro developmental toxicity assays [28,39,70,72,73]. Chemicals were tested at concentrations ranging from 1 pM to 200 µM, skewed towards the upper end of the concentration series. An empirical threshold (3*bmad above the solvent control) for determining assay bioactivity was set at 20.3%. Bioactive chemical curve fits yielded active concentration at cutoff (ACC) potency values representative of the baseline point of departure at the assay cutoff threshold. Consistent with expectations from the training set, acetaminophen was representative of no observed effect (Figure 4A) with consistent SOX17-positive cells across the concentration series (Figure 4C). In contrast, representative images for thalidomide displayed concentration-dependent loss of SOX17-positive cells (Figure 4B) and was classified as a bioactive chemical (ACC = 0.25 µM) (Figure 4D). Standard assay performance metrics calculated from plate-based controls indicated a high dynamic range (S/B: 63), high inter-well precision (rCV = 5.3%), and suitable screening quality (rZ’ = 0.78).

**Figure 5:**
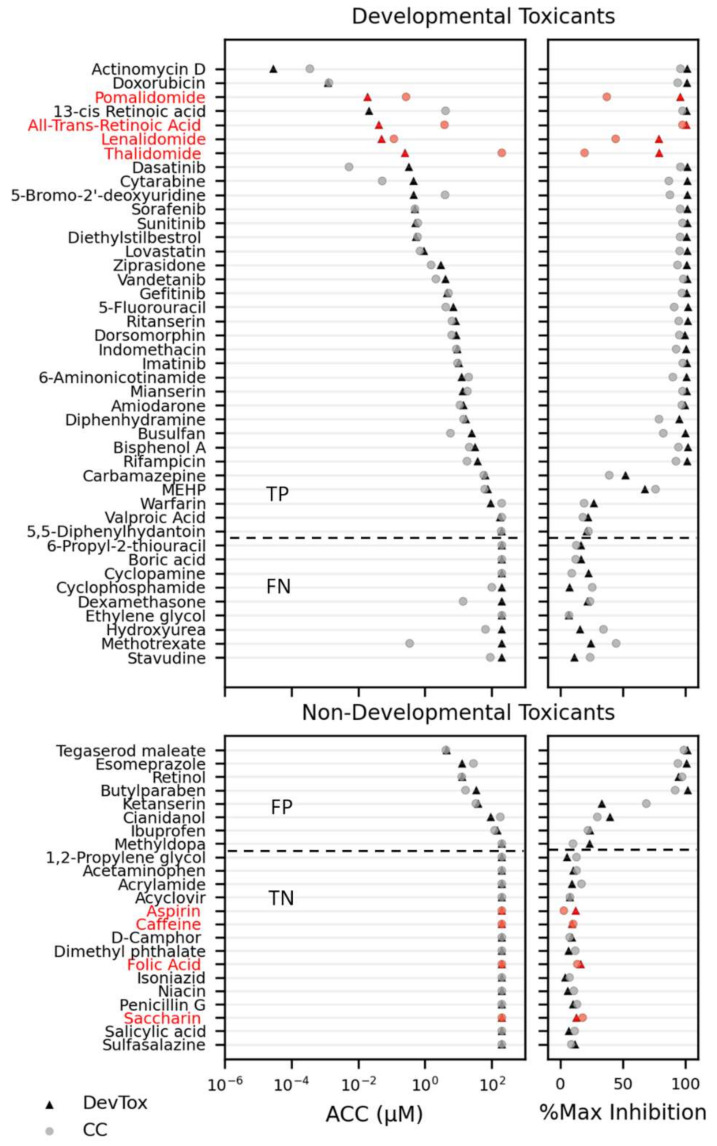
Chemical reference and training set screen results. Developmental toxicants and non-developmental toxicants are ranked by potency (left panels; activity concentration at cutoff (ACC)) with corresponding maximum effect size (right panels; % max inhibition) for percent SOX17-positive cell (DevTox—triangle) and cell count (CC—circle). Reference chemicals are indicated in red. The horizontal dashed line indicates separation of chemicals identified as positive (top) or negative (bottom) hit calls in the RUES2 GLR-Endo assay. (TP—true positive; FP—false positive; TN—true negative; FN—false negative).

Within the training set, the DevTox GLR-Endo assay predicted 38 positive and 20 negative chemicals with eight false-positive and nine false-negative designations (Figure 5 and Table S2). Chemical potency values (ACC) with corresponding efficacy (% maximum inhibition) are reported for both cell count and developmental toxicity. Twenty-four of the predicted positives had cytotoxic concentrations more potent than the SOX17-positive ACC, suggesting non-specific cytotoxic effects. In many instances, the chemical ACC values were below cytotoxic concentrations including 11 true-positive (13-cis retinoic acid, 5-bromo-2′-deoxyuridine, 6-aminonicotinamide, actinomycin D, diethylstilbestrol, doxorubicin, gefitinib, mianserin, sunitinib, valproic acid, and warfarin), and two false-positive (esomeprazole and cianidanol) compounds. Over half of the false-negative chemicals (cyclophosphamide, dexamethasone, hydroxyurea, methotrexate, and stavudine) had significant cell loss that did not impact SOX17-positive populations. In summary, the DevTox GLR-Endo assay performed with a balance accuracy of 67% (77% sensitivity, 58% specificity) for the 58 chemical training set.

### 3.5. Comparative Analysis of the DevTox GLR-Endo Assay Predictive Performance

Chemical classifications in the DevTox GLR-Endo assay were compared to concordant chemical effects in the hPST which both use the same SOX17 endpoint for predicting developmental hazards [47]. A subset of 34 benchmark chemicals common to both assays yielded a balanced accuracy of 96% (sensitivity 100%, specificity 92%) in the hPST. By comparison, the DevTox GLR-Endo assay demonstrated a balanced accuracy of 77% correctly predicting all true-positive developmental toxicants (100% sensitivity) but exhibiting a higher false-positive rate (54% specificity) (Table 3). One difference between the two assays is the analytical methodology used to determine and classify true-positive and true-negative effects. The hPST uses a potency threshold (IC_50_ = 30 µM) to stratify hit calls, whereas the DevTox GLR-Endo assay applies a data-driven threshold to effect size to define a binary outcome of active or inactive. Implementation of a 30 µM activity concentration at 50% (AC_50_) cutoff (a value equivalent to an IC_50_) to the DevTox GLR-Endo assay data decreased the sensitivity (86%) but improved the specificity (92%), resulting in a balanced accuracy (89%) similar to the hPST (96%).

The devTOX *quick*Predict assay measures perturbations to metabolite orthinine/cystine (o/c) ratios in H9 embryonic stem cells maintained in a pluripotent state [40]. The assay has been used to profile potential developmental hazards across a diverse cross-section of 1065 ToxCast chemicals [52]. The authors applied similar tcpl data modeling to the devTOX *quick*Predict assay dataset to determine hit calls. Potency values were reported for the Teratogen Index (TI), a metric previously established to ascribe a point of departure at the assay cutoff threshold [40] that is similar to the ACC potency value derived from tcpl. Comparing the TI and ACC values across the two assay platforms for a common benchmark set of 40 chemicals yielded a balanced accuracy of 83% (sensitivity 65%, specificity 100%) in the devTOX *quick*Predict assay (Table 4). In contrast, the DevTox GLR-Endo assay exhibited a balanced accuracy of 76% (sensitivity of 65%, specificity 86%). The DevTox GLR-Endo assay was more sensitive at predicting four positives (5,5-diphenylhydantoin, bisphenol A, diethylstilbestrol, warfarin) and lacked sensitivity for four other positives (dexamethasone, hydroxyurea, methotrexate, and stavudine) relative to the devTOX *quick*Predict assay. The four positive chemicals not correctly identified in the DevTox GLR-Endo assay induced cytotoxic effects without impacting the percentage of SOX17-positive cells, suggesting mode of action is not related to endoderm lineage commitment. 

## 4. Discussion

The Frank R. Lautenberg Chemical Safety for the 21st Century Act requires the EPA to designate chemical substances on the TSCA Chemical Substance Inventory as either “active” or “inactive” in U.S. commerce [10]. There are over 33,000 non-confidential chemicals manufactured or processed in the United States listed with an “active” designation (https://comptox.epa.gov/dashboard/chemical-lists/TSCA_ACTIVE_NCTI_0320, accessed on 16 February 2022). Considering only one third of the approximately eight million children born annually with birth defects are of defined etiology [74], there remains considerable uncertainty regarding the impact of thousands of data-poor environmental chemicals that could contribute to adverse developmental outcomes [3]. 

In this study, concepts previously published for the hPST assay were adapted to develop the DevTox GLR assay platform. A novel adaptation was the substitution of H9 ESCs with the RUES2-GLR ESC line. RUES2-GLR cells are engineered with endogenous gene reporters that enable the capacity to track progression of endoderm, mesoderm, and ectoderm lineages in real time. Under appropriate directed differentiation conditions, the assay platform has the potential to evaluate chemical effects on all three primary germ layer lineages. To bridge observations made in the hPST, this study evaluated chemical effects in the context of endoderm differentiation, thus establishing predictive performance of the DevTox GLR-Endo assay variant. Use of RUES2-GLR cells minimizes endpoint processing, negating the need for cost-and time-intensive immunocytochemistry for high-content imaging analysis. Transition to a higher-throughput 384-well format and a two-day differentiation protocol expands screening potential while shortening the assay duration for enhanced speed and efficiency at reduced cost.

Gene expression of RUES2-GLR ESCs was comparable to H9 ESCs under pluripotent and directed endoderm conditions (Figure 1A). Both cell lines have relatively low expression of ectodermal, mesodermal, and endodermal genes in a pluripotent state and exhibit a similar pattern in all differentiation states under directed endoderm induction, indicating the cell line is an appropriate substitution to H9 cells. The cell seeding density was optimized for efficient endoderm formation in a 384-well format and automated liquid handling technologies employed to enhance reproducibility of the workflow. Standard assay performance metrics for the DevTox GLR-Endo assay revealed a high dynamic range (S/B: 63), high inter-well precision (rCV = 5.3%), and suitable screening quality (rZ’ = 0.78) indicating a robust platform for high-throughput screening.

The hPST is reported to identify positive cells via SOX17 nuclear localization where it can act as a transcriptional regulator for endoderm differentiation [47]. Gene expression results in this study revealed SOX17 log2 fold-change values of 5.5 after two days of endoderm differentiation (Figure 1B). Since SOX family proteins contain conserved nuclear localization sequence domains, increased expression of SOX17 is concordant with nuclear localization and accumulation [75,76]. This suggests induction of SOX17 gene expression is the key initiating event to observing endoderm-dependent nuclear localization.

The outcome of a benchmark chemical training set, comprised of 58 chemicals deemed positive or negative for developmentally toxicity, resulted in a balanced accuracy of 67% (77% sensitivity, 58% specificity). In total, with all 66 chemicals tested, including the eight reference chemicals, the assay provides a balanced accuracy of 72% (79% sensitivity, 65% specificity). This predictive accuracy is comparable to the mEST (78%) [28,29], Molecular-EST (72–83%) [71], devTOX *quick*Predict (77%) [40], rat micromass test (70%) [28], rat whole embryo culture (68–80%) [28], and zebrafish embryotoxicity test (72%) [77,78]. The positive and negative predictive values suggest the assay was capable of identifying true-positive effects but was more prone to false-positive outcomes. Increasing the baseline threshold cutoff may decrease the false-positive rate whilst maintaining appreciable sensitivity to identify true developmental toxicant effects. With a focus on being health protective, a more conservative approach would maintain higher false-positive rates for screening campaigns entailing thousands of chemicals where the primary goal is to triage and prioritize chemicals with potential biological effects, and then pursue orthogonal testing in complementary assays to confirm or reject the findings. Either way, the predictive performance of the assay is, in part, dependent on the analytical methods used to define bioactivity, as evident in the comparative analyses with prior methods, and is suitable for primary screening of large data-poor chemical sets.

Stratification of the training set chemicals based on maximum observed effect level (% max inhibition) enabled consideration of inaccurate hit calls across the chemical series. Three classifications were as follows: (1) high (80–100% max inhibition), (2) moderate (20–80% max inhibition), and (3) weak (0–20% max inhibition). For the 29 high-response chemicals, all were predicted to be positive with 4/29 (butylparaben, tegaserod maleate, esomeprazole, and retinol) considered false positive. These four chemicals unambiguously impacted SOX17 expression but are classified as true negatives. False-positive calls in this assay could be attributed to exposure concentrations higher than in vivo exposures. For instance, retinol (vitamin A) is a necessary nutrient for human embryonic development but supraphysiological levels of exposure are known to cause neural tube, thymus and heart malformation defects [79]. Additionally, retinol can be metabolized to more potent retinoid metabolites (e.g., all-trans-retinoic acid) with greater developmental toxicity potential [72]. All 17 weak-response chemicals were predicted negative with 6/17 (boric acid, 6-proply-2-thiouracil, hydroxyurea, stavudine, cyclophosphamide, ethylene glycol) considered false negative. Cyclophosphamide and ethylene glycol are known to be bioactivated by liver metabolism, likely explaining how detection was missed [80,81,82,83]. The 12 moderate-response chemicals were the least accurate with five true positive, four false positive, and three false-negative designations for an overall predictive accuracy of 42%. The false-negative chemicals (methotrexate, cyclopamine, and dexamethasone) were borderline calls and perhaps would be positive if exposure time were increased or analytical parameters adjusted. Between the weak and moderate response groups, some chemicals affected total cell count without impacting the percentage of SOX17 cells. If decreases in total cell count is also used in predicting developmental toxicants, in conjunction with loss in SOX17-positive cells, five additional chemicals (cyclophosphamide, dexamethasone, hydroxyurea, methotrexate, and stavudine) are correctly identified, increasing the assay sensitivity to 87% (balanced accuracy of 73%). In addition to modifications to data analytical procedures, decreasing false-positive and false-negative rates may require incorporation of additional germ layer lineages (e.g., mesoderm and ectoderm), considerations of chemical treatment frequency, duration, and cumulative effect, as well as integration of other biological and mechanistic aspects of embryonic development (e.g., developmental stage, maternal-fetal interactions, and xenobiotic metabolism) into screening efforts. A more holistic approach inclusive of a battery of in vitro developmental test methods would contribute weight of evidence toward the probability and mechanistic underpinning of potential human developmental toxicants [84].

Comparing the 34 chemical overlap in the DevTox GLR-Endo reference and training set to the hPST dataset revealed chemical responses with similar potency values. Nearly all the true-positive chemicals had potency values within one order of magnitude. These results were anticipated given similar assay duration and SOX17 biomarker analysis. Five chemicals (cianidanol, esomeprazole, ketanserin, ibuprofen, and methyldopa) were identified as false positive in the DevTox GLR-Endo but not the hPST (Table 3). The potency values were similar between the hPST (IC_50_) and DevTox GLR-Endo (AC_50_) assays for cianidanol (97 µM vs. 93 µM), esomeprazole (32 µM vs. 34 µM) and methyldopa (272 µM vs. 113 µM). This conservation between different pluripotent stem cell lines propagates the notion of SOX17 expression being an effective endpoint measurement for endoderm-specific toxicity. 

Despite similar balanced accuracy in the devTOX *quick*Predict (83%) and DevTox GLR-Endo (76%) assay platforms for a common benchmark set of 40 chemicals, there were notable differences. The devTOX *quick*Predict assay correctly identified all six of the positive chemicals (5-fluorouracil, cytarabine, busulfan, hydroxyurea, methotrexate, and stavudine) that have known mechanisms of action as DNA synthesis inhibitors. The DevTox GLR-Endo assay correctly predicted only three (5-fluorouracil, cytarabine, busulfan) of the six. However, significant decreases in cell count were seen for all six chemicals (Table 4). The o/c ratio utilized in the devTOX *quick*Predict assay measures metabolic changes to the hPSC population that encompasses a broad range of metabolic pathways including reactive oxygen species and DNA stabilization pathways [40]. Inhibition of DNA synthesis during S phase can damage DNA, resulting in genetic lesions that may lead to programmed cell death if left unrepaired [85]. Ornithine is important for generating polyamines that are responsible for stabilizing newly synthesized DNA, so altered levels of ornithine could be indicative of DNA damage within the cell [86]. Given inhibition of DNA synthesis occurs in the absence of SOX17 expression perturbation and has a negative impact on embryonic cell proliferation and survival, the exclusive use of SOX17 as an indicator of developmental toxicity is unlikely to capture all relevant mechanistic developmental effects. In contrast, limitations in the devTOX *quick*Predict assay may be complemented by the DevTox GLR-Endo assay. For instance, a subset of false-negative observations in the devTOX *quick*Predict assay had a strong correlation to estrogenic chemicals [52]. The DevTox GLR-Endo assay was sensitive to the synthetic estrogen diethylstilbestrol while the devTOX *quick*Predict assay was not. The devTOX *quick*Predict assay responsiveness toward DNA damaging chemicals and DevTox GLR-Endo assay responsiveness toward estrogens illustrates how the two assays in combination could increase overall sensitivity.

The RUES2-GLR cell line provides the opportunity to collect additional mechanistic data using SOX2 and BRA fluorescent reporters as differentiation indicators of ectoderm or mesoderm lineages, respectively. No reference or training set chemicals tested in the DevTox GLR-Endo assay increased SOX2 (AEID 3095) or BRA (AEID 3096) protein expression levels. Modification of the model to assay modes specific for directed mesoderm (DevTox GLR-Meso) and directed ectoderm (DevTox GLR-Ecto) differentiation may provide additional mechanistic and predictive insight into chemical effects. 

While no significant changes due to chemical exposure were seen for SOX2 and BRA, expansion of the chemical space covered could identify compounds that modulate Activin/WNT signaling or stimulate other differentiation pathways, resulting in altered lineage trajectories. A toxicological “tipping point” concentration for all-trans-retinoic acid (ATRA) has previously been identified in H9 ESCs differentiated toward definitive endoderm; defining a transition point between adaptive and adverse cellular response [87]. To establish this concentration, global gene expression was evaluated at 6, 96, and 192 h time points with ATRA exposure. This analysis identified a “tipping point” of 17 ± 11 nM with an underlying developmental trajectory shift toward mesoderm and extraembryonic endoderm. The attenuation of endoderm markers at 96 h was concordant with a concentration-dependent increase in BRA (gene name *TBXT*) expression. Despite the lack of significant BRA perturbation by ATRA at 48 h of exposure in the DevTox GLR-Endo assay (AEID 3036), the DevTox GLR model may still be useful for evaluating developmental trajectory shifts under optimized conditions designed to address tipping point hypotheses. For example, SOX2, SOX17, and BRA fluorescent biomarkers can be monitored without fixation of cells, enabling live-cell imaging for observation of concentration-dependent trajectories of each biomarker over multiple paired time points. 

Currently, there are no validated non-animal in vitro test methods used in regulatory decision making for developmental toxicity due to challenges in replicating the predictive power in animal models [88]. International OECD test guidelines for developmental and reproductive toxicity mandate the use of animal models to characterize both the hazard and risk of potential toxicants [9]. In vivo animal testing is a time- and labor-intensive process that is costly, has limited testing throughput, and maintains difficulty in extrapolation of animal to human developmental effects. Given the high-throughput capacity, low cost, and reasonable predictive accuracy (72%), the DevTox GLR-Endo assay is a promising approach toward rapidly identifying and ascribing mechanisms of chemical toxicity associated with human development while being mindful of animal welfare.

## Figures and Tables

**Table 1:** Reference chemicals. Chemical name, CAS registry number (CASRN), DSSTox substance identifier (DTXSID), developmental toxicant classification, FDA pregnancy category, 50% inhibitory concentration (IC_50_) and percentage maximum inhibition (% max inhibition).

Chemical	CASRN	DTXSID	Developmental Toxicant Classification	FDA Pregnancy Category	IC_50_ (µM)	% Max Inhibition
All-Trans Retinoic Acid	302-79-4	DTXSID7021239	Positive	X	0.16	93
Lenalidomide	191732-72-6	DTXSID8046664	Positive	X	0.13	44
Pomalidomide	19171-19-8	DTXSID40893458	Positive	X	0.05	81
Thalidomide	50-35-1	DTXSID9022524	Positive	X	1.71	58
Aspirin	50-78-2	DTXSID5020108	Negative	C	-	-
Caffeine	58-08-2	DTXSID0020232	Negative	B	-	-
Folic Acid	59-30-3	DTXSID0022519	Negative	A	-	-
Saccharin	81-07-2	DTXSID5021251	Negative	A	-	-

**Table 2:** DevTox RUES2-GLR Endo Assay Endpoints**.** Assay endpoint IDs (AEID); Assay endpoint name; interpretation: Positive–increase in biomarker or cell count, negative–reduction in biomarker or cell count.

Assay Endpoint ID (AEID)	Assay Endpoint Name	Interpretation
3093	CCTE_Deisenroth_DEVTOX_RUES2-GLR_Endo_Sox17_up	SOX17 positive
3094	CCTE_Deisenroth_DEVTOX_RUES2-GLR_Endo_Sox17_dn	SOX17 negative
3095	CCTE_Deisenroth_DEVTOX_RUES2-GLR_Endo_Sox2_up	SOX2 positive
3096	CCTE_Deisenroth_DEVTOX_RUES2-GLR_Endo_Bra_up	BRA positive
3097	CCTE_Deisenroth_DEVTOX_RUES2-GLR_Endo_ CellCount_up	Cell count positive
3098	CCTE_Deisenroth_DEVTOX_RUES2-GLR_Endo_ CellCount_dn	Cell count negative

**Table 3:** Comparison of chemical responses between the DevTox GLR-Endo and hPST assays. Comparisons were drawn based on reported hPST assay analysis.

CASRN	Chemical Name	DT Class.	hPST			DevTox-GLR Endo		
			TC_50_ (µM)	IC_50_ (µM)	Call	CC_50_ (µM)	AC_50_ (µM)	Call
50-76-0	Actinomycin D	Pos	<0.01	<0.01	TP	4.8 × 10^−4^	3.7 × 10^−5^	TP
25316-40-9	Doxorubicin	Pos	<0.3	0.3	TP	3.3 × 10^−3^	1.9 × 10^−3^	TP
302-79-4	All-Trans-Retinoic Acid	Pos	33	0.07	TP	5.53	0.10	TP
4759-48-2	13-cis Retinoic Acid	Pos	140	0.2	TP	10.24	0.16	TP
59-14-3	5-Bromo-2’-deoxyuridine	Pos	3.4	0.2	TP	9.37	0.55	TP
284461-73-0	Sorafenib	Pos	<2	<2	TP	0.61	0.62	TP
341031-54-7	Sunitinib	Pos	3.6	2.8	TP	1.26	0.63	TP
56-53-1	Diethylstilbestrol	Pos	4.4	4.4	TP	0.71	0.64	TP
302962-49-8	Dasatinib	Pos	<1	<1	TP	0.05	0.72	TP
50-35-1	Thalidomide	Pos	>200	0.5	TP	>200	1.64	TP
146939-27-7	Ziprasidone	Pos	5.1	2.8	TP	2.42	3.57	TP
443913-73-3	Vandetanib	Pos	4.4	3.5	TP	4.32	4.90	TP
184475-35-2	Gefitinib	Pos	10	10	TP	6.60	5.75	TP
51-21-8	5-Fluorouracil	Pos	0.7	0.6	TP	5.72	8.48	TP
87051-43-2	Ritanserin	Pos	27	7.3	TP	9.47	9.98	TP
220127-57-1	Imatinib	Pos	22	11	TP	13.41	13.45	TP
329-89-5	6-Aminonicotinamide	Pos	8	7	TP	23.85	16.61	TP
866405-64-3	Dorsomorphin	Pos	2.4	0.3	TP	16.7	17.1	TP
21535-47-7	Mianserin	Pos	60	3.4	TP	33.56	34.85	TP
298-46-4	Carbamazepine	Pos	0.19	0.08	TP	136.8	102.7	TP
99-66-1	Valproic Acid	Pos	3.3	2.6	TP	>200	123.9	TP
59277-89-3	Acyclovir	Neg	>200	>200	TN	>200	>200	TN
50-78-2	Aspirin	Neg	>200	>200	TN	>200	>200	TN
58-08-2	Caffeine	Neg	>400	>400	TN	>200	>200	TN
59-30-3	Folic Acid	Neg	84	39	TN	>200	>200	TN
59-67-6	Niacin	Neg	>200	>200	TN	>200	>200	TN
113-98-4	Penicillin G	Neg	>400	>400	TN	>200	>200	TN
81-07-2	Saccharin	Neg	>400	>400	TN	>200	>200	TN
189188-57-6	Tegaserod Maleate	Neg	4.5	4.6	FP	5.06	5.32	FP
668985-31-7	Esomeprazole	Neg	101	32	TN	38.5	33.8	FP
74050-98-9	Ketanserin	Neg	>200	>200	TN	57.5	36.6	FP
154-23-4	Cianidanol	Neg	200	97	TN	193.6	92.9	FP
41372-08-1	Methyldopa	Neg	462	272	TN	>200	113.1	FP
15687-27-1	Ibuprofen	Neg	>200	>200	TN	79.4	161.8	FP
			Sensitivity		100.0%			100.0%
			Specificity		92.3%			53.8%
			Balanced Accuracy		96.2%			76.9%

Developmental toxicant classification (DT Class.), positive (Pos), negative (Neg); cytotoxicity half-maximal concentration (TC_50_); cell count half-maximal concentration (CC_50_); SOX17 half-maximal activation/inhibition concentration (AC_50_/IC_50_); hit calls (TP—true positive; FP—false positive; TN—true negative; FN—false negative). Sensitivity, specificity, and balanced accuracy are noted.

**Table 4:** Comparison of chemical responses between the DevTox GLR-Endo and devTOX *quick*Predict assays. Comparisons were drawn based on reported *quick*Predict assay analysis.

CASRN	Chemical Name	DT Class.	*quick*Predict			DevTox-GLR Endo		
			CV (µM)	TI (µM)	Call	CC (µM)	ACC (µM)	Call
302-79-4	All-Trans-Retinoic Acid	Pos	NA	0.003	TP	3.84	0.04	TP
50-35-1	Thalidomide	Pos	NA	1.27	TP	NA	0.25	TP
69-74-9	Cytarabine	Pos	0.083	0.054	TP	0.05	0.46	TP
56-53-1	Diethylstilbestrol	Pos	NA	NA	FN	0.60	0.54	TP
75330-75-5	Lovastatin	Pos	NA	5.1	TP	0.71	0.93	TP
51-21-8	5-Fluorouracil	Pos	1.45	2.02	TP	20.34	7.07	TP
53-86-1	Indomethacin	Pos	44.1	72.7	TP	8.70	9.38	TP
19774-82-4	Amiodarone	Pos	NA	5.1	TP	11.14	14.22	TP
147-24-0	Diphenhydramine	Pos	3.76	0.588	TP	14.34	16.78	TP
55-98-1	Busulfan	Pos	4.91	2.31	TP	5.79	25.26	TP
80-05-7	Bisphenol A	Pos	39.4	NA	FN	21.12	31.75	TP
13292-46-1	Rifampicin	Pos	NA	2.46	TP	18.11	37.85	TP
298-46-4	Carbamazepine	Pos	NA	2.29	TP	57.78	64.02	TP
4376-20-9	MEHP	Pos	NA	167	TP	61.55	76.33	TP
81-81-2	Warfarin	Pos	NA	NA	FN	197.75	93.33	TP
99-66-1	Valproic Acid	Pos	271	155	TP	NA	177.33	TP
57-41-0	5,5-Diphenylhydantoin	Pos	NA	NA	FN	191.82	201.56	TP
57-55-6	1,2-Propylene glycol	Neg	246,664	327,552	TN	NA	NA	TN
103-90-2	Acetaminophen	Neg	NA *	NA	TN	NA	NA	TN
79-06-1	Acrylamide	Neg	NA	NA	TN	NA	NA	TN
50-78-2	Aspirin	Neg	NA *	NA	TN	NA	NA	TN
58-08-2	Caffeine	Neg	NA	NA	TN	NA	NA	TN
464-49-3	D-Camphor	Neg	NA	NA	TN	NA	NA	TN
131-11-3	Dimethyl Phthalate	Neg	NA	NA	TN	NA	NA	TN
59-30-3	Folic Acid	Neg	NA	NA	TN	NA	NA	TN
54-85-3	Isoniazid	Neg	NA *	NA	TN	NA	NA	TN
81-07-2	Saccharin	Neg	NA	NA	TN	NA	NA	TN
69-72-7	Salicylic Acid	Neg	1795	513	TN	NA	NA	TN
599-79-1	Sulfasalazine	Neg	NA *	NA	TN	NA	NA	TN
68-26-8	Retinol	Neg	NA	NA	TN	12.63	13.05	FP
94-26-8	Butylparaben	Neg	NA	NA	TN	NA	34.47	FP
51-52-5	6-Propyl-2-thiouracil	Pos	NA	NA	FN	NA	NA	FN
10043-35-3	Boric acid	Pos	NA	NA	FN	NA	NA	FN
4449-51-8	Cyclopamine	Pos	NA	NA	FN	NA	NA	FN
6055-19-2	Cyclophosphamide	Pos	NA *	NA	FN	100.19	NA	FN
2392-39-4	Dexamethasone	Pos	21.8	37.7	TP	13.83	NA	FN
107-21-1	Ethylene Glycol	Pos	NA	NA	FN	NA	NA	FN
127-07-1	Hydroxyurea	Pos	237	74.9	TP	64.54	NA	FN
133073-73-1	Methotrexate	Pos	0.062	0.059	TP	0.35	NA	FN
3056-17-5	Stavudine	Pos	NA	32.5	TP	90.28	NA	FN
			Sensitivity		65.4%			65.38%
			Specificity		100.0%			85.7%
			Balanced Accuracy		82.7%			75.5%

Developmental toxicant classification (DT class.), positive (Pos), negative (Neg); CV-concentration at cell viability cutoff; concentration at cell count cutoff (CC); Teratogen Index (TI); activity concentration at cutoff (ACC); hit calls (TP—true positive; FP—false positive; TN—true negative; FN—false negative). Sensitivity, specificity, and balanced accuracy are noted. * Inferred inactivity from a single-concentration screen.

## Data Availability

Data is contained within the article or supplementary material. The raw data loaded into the ToxCast Pipeline database, invitrodb (to be released in version 3.5, expected Summer 2022), can be accessed at the U.S. EPA CompTox Chemicals Dashboard (https://comptox.epa.gov/dashboard, accessed on 19 May 2022).

## Supplementary Materials

The following supporting information can be downloaded at: https://www.mdpi.com/article/10.3390/toxics10070392/s1, README_Gamble_DevTox GLR_Sup Data_v1.docx, Gamble_DevTox GLR_Sup Fig_Submission_v1.docx, Gamble_DevTox GLR_Tables_Submission_v1.xlsx, Gamble_DevTox GLR_Sup Tables_Submission_v1.xlsx, ToxCast Pipeline plots for AEID 3093-3098.

## Acronyms and Abbreviations

AC50	activity concentration at 50%
ACC	active concentration at cutoff
bmad	baseline median absolute deviation
BRA	Brachyury
DevTox	developmental toxicity
Endo	endoderm
EPA	Environmental Protection Agency
ESC	embryonic stem cell
hPSC	human pluripotent stem cell
hPST	human pluripotent stem cell test
IC50	inhibition concentration at 50%
mEST	mouse Embryonic Stem Cell Test
NAM	new approach method
OECD	Organization of Economic-Co-operation and Development
PSC	pluripotent stem cell
rCV	robust coefficient of variation
RUES2-GLR	Rockefeller University Embryonic Stem cell line 2–Germ Layer Reporter
rZ’	robust Z-prime factor
SOX17	SRY-box transcription factor 17
SOX2	SRY-box transcription factor 2
tcpl	ToxCast Pipeline
TGF-β	transforming growth factor β
TSCA	Toxic Substance Control Act
