# Supplementary material for: The DevTox Germ Layer Reporter Platform: An Assay Adaptation of the Human Pluripotent Stem Cell Test
Source: Toxics. 2022 Jul 13;10(7):392. doi: 10.3390/toxics10070392 (PMC9321663; doi:10.3390/toxics10070392)
Supplement: Supplementary file 1 [file toxics-10-00392-s001.zip › AEID3093_L6_CCTE_Deisenroth_DEVTOX_RUES2-GLR_Sox17_up_210917.pdf]

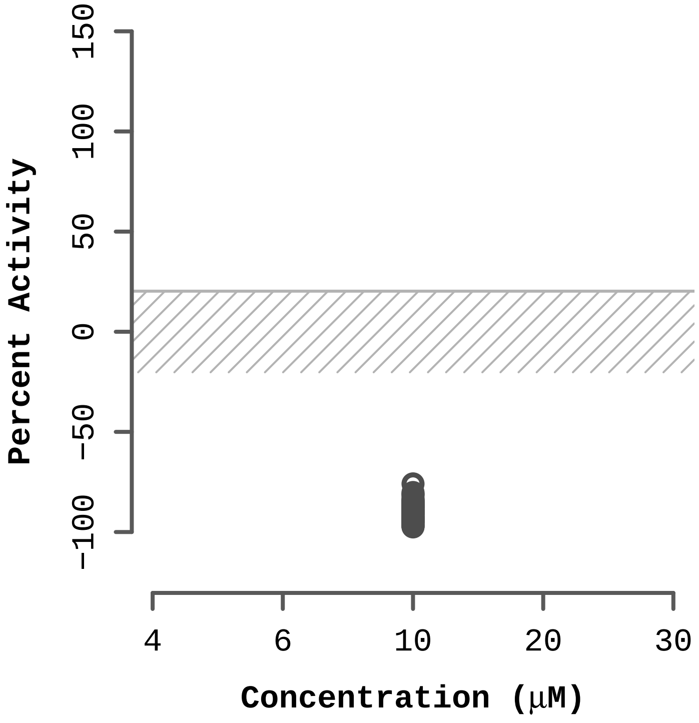

ASSAY: AEID3093 (CCTE_Deisenroth_DEVTOX_RUES2-GLR_Sox[illegible]

NAME: SB−431542
CHID: 42693 CASRN: 301836−41−9
SPID(S): ES211_56_56
M4ID: 42675855

HILL MODEL: Not applicable.

GAIN−LOSS MODEL: Not applicable.

	CNST	HILL	GNLS
AIC:	NA	NA	NA
PROB:	NA	NA	NA
RMSE:	NA	NA	NA

MAX_MEAN: −90 MAX_MED: −90.4 BMAD: 6.75

COFF: 20.3 HIT−CALL: −1 FITC: 2 ACTP: NA

FLAGS:

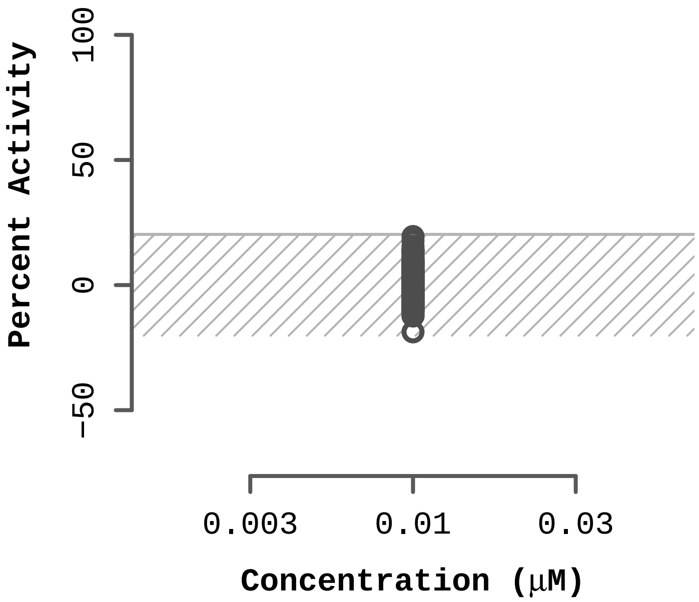

ASSAY: AEID3093 (CCTE_Deisenroth_DEVTOX_RUES2-GLR_Sox:

NAME: DMSO (spid not in DB)
CHID: NA CASRN: NA
SPID(S): DMSO
M4ID: 42675870

HILL MODEL: Not applicable.

GAIN−LOSS MODEL: Not applicable.

	CNST	HILL	GNLS
AIC:	NA	NA	NA
PROB:	NA	NA	NA
RMSE:	NA	NA	NA

MAX_MEAN: 0.5 MAX_MED: −5.73e−15 BMAD: 6.75

COFF: 20.3 HIT−CALL: −1 FITC: 2 ACTP: NA

FLAGS:

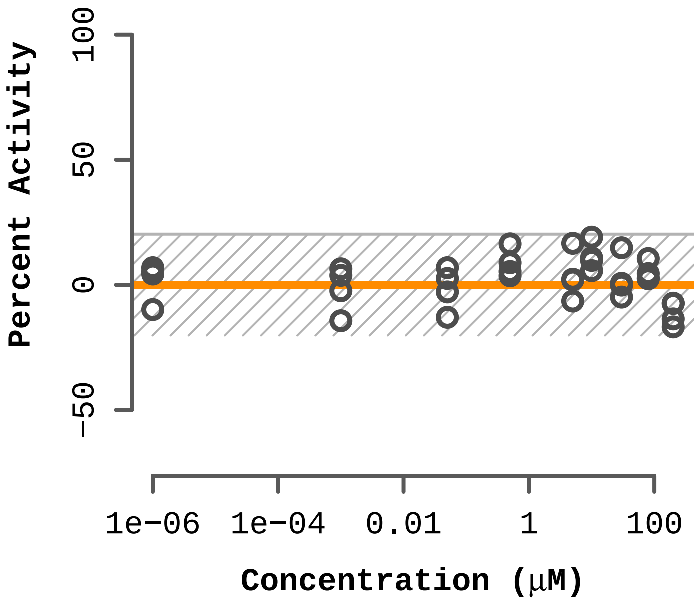

ASSAY: AEID3093 (CCTE_Deisenroth_DEVTOX_RUES2-GLR_Sox:

NAME: Acetaminophen
CHID: 20006 CASRN: 103−90−2
SPID(S): ES211_9_9
M4ID: 42675840

HILL MODEL: Not applicable.

GAIN−LOSS MODEL: Not applicable.

	CNST	HILL	GNLS
AIC:	266.51	NA	NA
PROB:	1	NA	NA
RMSE:	9.09	NA	NA

MAX_MEAN: 11.3 MAX_MED: 10.3 BMAD: 6.75

COFF: 20.3 HIT−CALL: 0 FITC: 4 ACTP: 0

FLAGS:

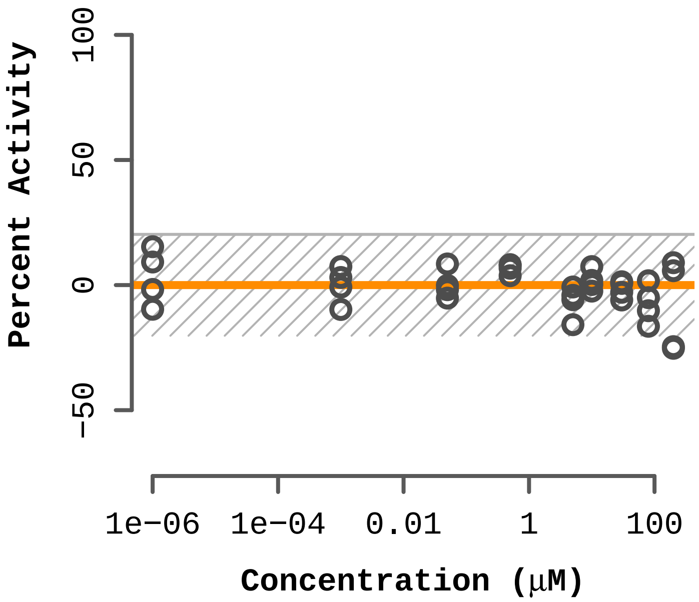

ASSAY: AEID3093 (CCTE_Deisenroth_DEVTOX_RUES2-GLR_Sox

NAME: Acrylamide
CHID: 20027 CASRN: 79-06-1
SPID(S): ES211_10_10
M4ID: 42675899

HILL MODEL: Not applicable.

GAIN-LOSS MODEL: Not applicable.

	CNST	HILL	GNLS
AIC:	262.31	NA	NA
PROB:	1	NA	NA
RMSE:	9.19	NA	NA

MAX_MEAN: 6.64 MAX_MED: 7.31 BMAD: 6.75

COFF: 20.3 HIT-CALL: 0 FITC: 4 ACTP: 0

FLAGS:

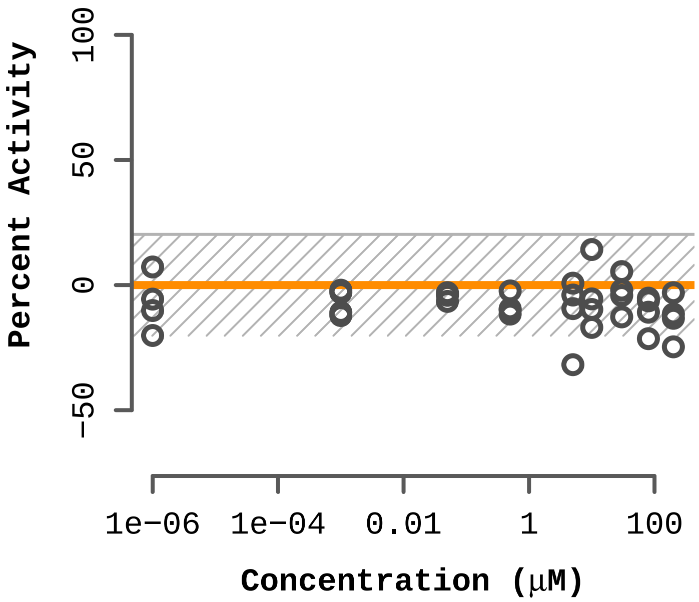

ASSAY: AEID3093 (CCTE_Deisenroth_DEVTOX_RUES2-GLR_Sox

NAME: Aspirin
CHID: 20108 CASRN: 50-78-2
SPID(S): ES211_15_15
M4ID: 42675837

HILL MODEL: Not applicable.

GAIN-LOSS MODEL: Not applicable.

	CNST	HILL	GNLS
AIC:	281.49	NA	NA
PROB:	1	NA	NA
RMSE:	11.54	NA	NA

MAX_MEAN: -3.36 MAX_MED: -3.07 BMAD: 6.75

COFF: 20.3 HIT-CALL: 0 FITC: 4 ACTP: 0

FLAGS:

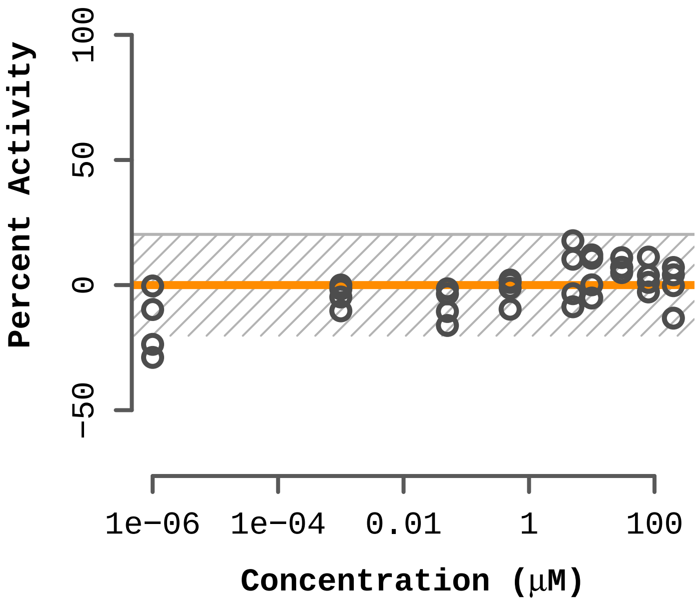

ASSAY: AEID3093 (CCTE_Deisenroth_DEVTOX_RUES2-GLR_Sox

NAME: Boric acid (H3BO3)
CHID: 20194 CASRN: 10043-35-3
SPID(S): ES211_17_17
M4ID: 42675883

HILL MODEL: Not applicable.

GAIN-LOSS MODEL: Not applicable.

	CNST	HILL	GNLS
AIC:	268.7	NA	NA
PROB:	1	NA	NA
RMSE:	9.92	NA	NA

MAX_MEAN: 7.53 MAX_MED: 7.06 BMAD: 6.75

COFF: 20.3 HIT-CALL: 0 FITC: 4 ACTP: 0

FLAGS:

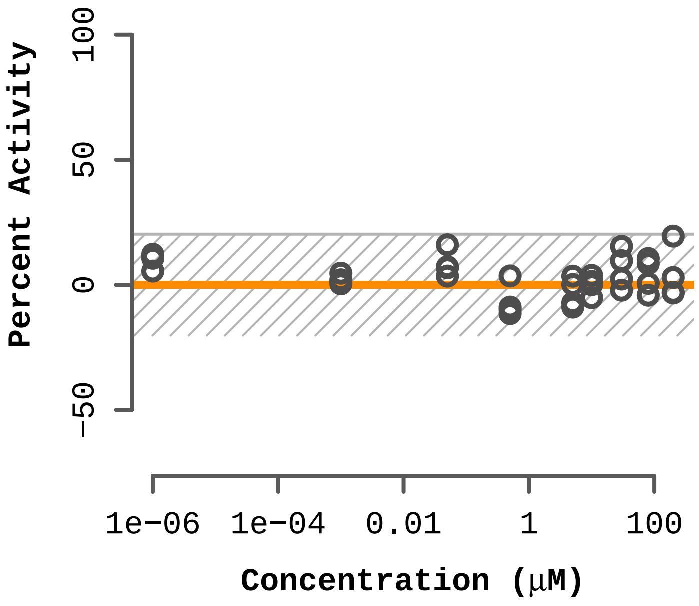

ASSAY: AEID3093 (CCTE_Deisenroth_DEVTOX_RUES2-GLR_Sox:

NAME: Caffeine
CHID: 20232 CASRN: 58−08−2
SPID(S): ES211_20_20
M4ID: 42675867

HILL MODEL: Not applicable.

GAIN−LOSS MODEL: Not applicable.

	CNST	HILL	GNLS
AIC:	255.63	NA	NA
PROB:	1	NA	NA
RMSE:	7.94	NA	NA

MAX_MEAN: 9.91 MAX_MED: 11 BMAD: 6.75

COFF: 20.3 HIT−CALL: 0 FITC: 4 ACTP: 0

FLAGS:

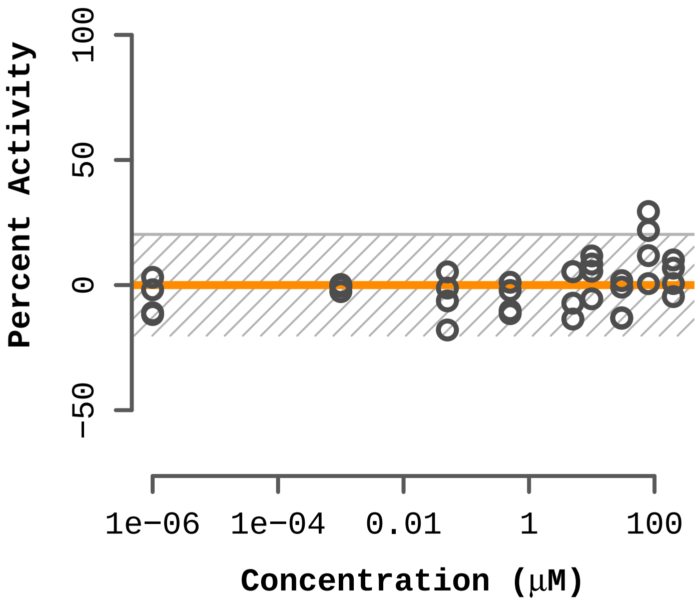

ASSAY: AEID3093 (CCTE_Deisenroth_DEVTOX_RUES2-GLR_Sox:

NAME: Ethylene glycol
CHID: 20597 CASRN: 107-21-1
SPID(S): ES211_32_32
M4ID: 42675871

HILL MODEL: Not applicable.

GAIN-LOSS MODEL: Not applicable.

	CNST	HILL	GNLS
AIC:	268.12	NA	NA
PROB:	1	NA	NA
RMSE:	9.82	NA	NA

MAX_MEAN: 15.9 MAX_MED: 16.8 BMAD: 6.75

COFF: 20.3 HIT-CALL: 0 FITC: 4 ACTP: 0

FLAGS:

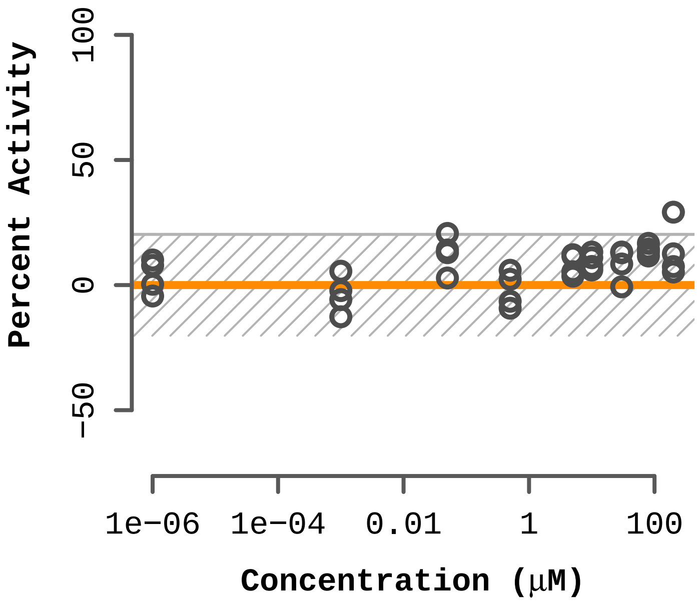

ASSAY: AEID3093 (CCTE_Deisenroth_DEVTOX_RUES2-GLR_Sox

NAME: Isoniazid
CHID: 20755 CASRN: 54-85-3
SPID(S): ES211_39_39
M4ID: 42675850

HILL MODEL: Not applicable.

GAIN-LOSS MODEL: Not applicable.

	CNST	HILL	GNLS
AIC:	280.85	NA	NA
PROB:	1	NA	NA
RMSE:	11.04	NA	NA

MAX_MEAN: 14 MAX_MED: 13.9 BMAD: 6.75

COFF: 20.3 HIT-CALL: 0 FITC: 4 ACTP: 0

FLAGS:

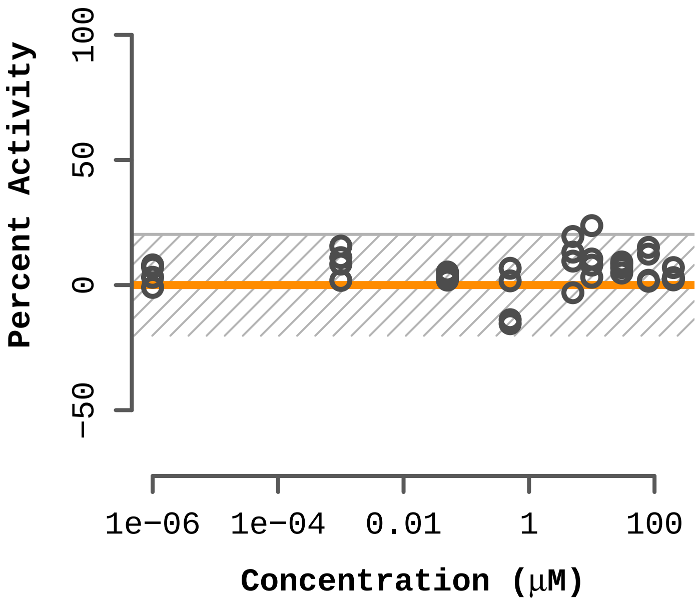

ASSAY: AEID3093 (CCTE_Deisenroth_DEVTOX_RUES2-GLR_Sox:

NAME: Nicotinic acid
CHID: 20932 CASRN: 59-67-6
SPID(S): ES211_48_48
M4ID: 42675842

HILL MODEL: Not applicable.

GAIN-LOSS MODEL: Not applicable.

	CNST	HILL	GNLS
AIC:	267.87	NA	NA
PROB:	1	NA	NA
RMSE:	9.41	NA	NA

MAX_MEAN: 11.3 MAX_MED: 11.4 BMAD: 6.75

COFF: 20.3 HIT-CALL: 0 FITC: 4 ACTP: 0

FLAGS:

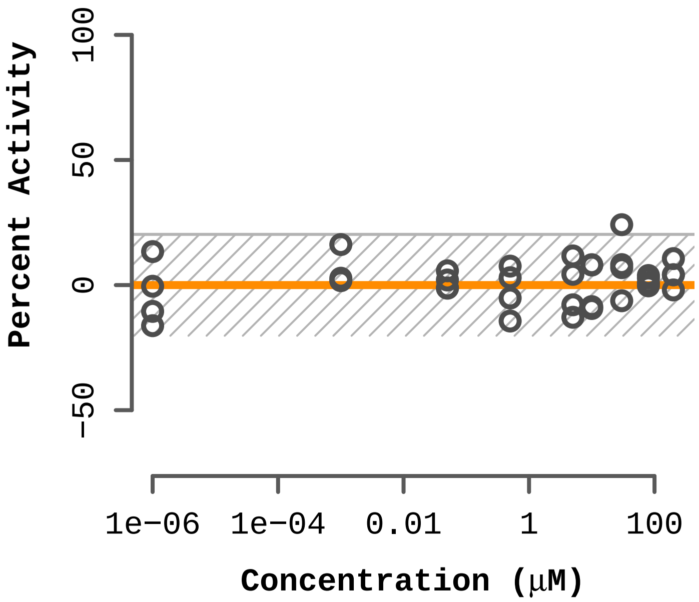

ASSAY: AEID3093 (CCTE_Deisenroth_DEVTOX_RUES2-GLR_Sox:

NAME: 1,2-Propylene glycol
CHID: 21206 CASRN: 57-55-6
SPID(S): ES211_1_1
M4ID: 42675841

HILL MODEL: Not applicable.

GAIN-LOSS MODEL: Not applicable.

	CNST	HILL	GNLS
AIC:	261.77	NA	NA
PROB:	1	NA	NA
RMSE:	8.75	NA	NA

MAX_MEAN: 8.27 MAX_MED: 7.58 BMAD: 6.75

COFF: 20.3 HIT-CALL: 0 FITC: 4 ACTP: 0

FLAGS:

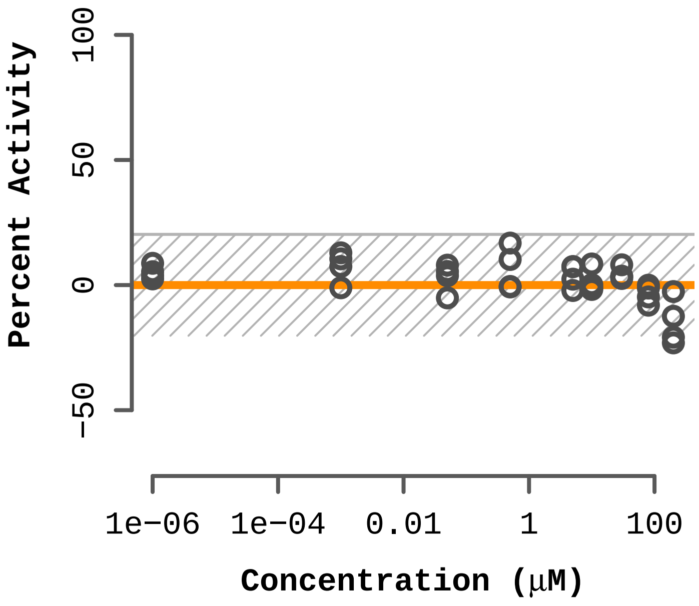

ASSAY: AEID3093 (CCTE_Deisenroth_DEVTOX_RUES2-GLR_Sox:

NAME: 6-Propyl-2-thiouracil
CHID: 21209 CASRN: 51-52-5
SPID(S): ES211_8_8
M4ID: 42675860

HILL MODEL: Not applicable.

GAIN-LOSS MODEL: Not applicable.

	CNST	HILL	GNLS
AIC:	255.62	NA	NA
PROB:	1	NA	NA
RMSE:	8.32	NA	NA

MAX_MEAN: 7.44 MAX_MED: 8.97 BMAD: 6.75

COFF: 20.3 HIT-CALL: 0 FITC: 4 ACTP: 0

FLAGS:

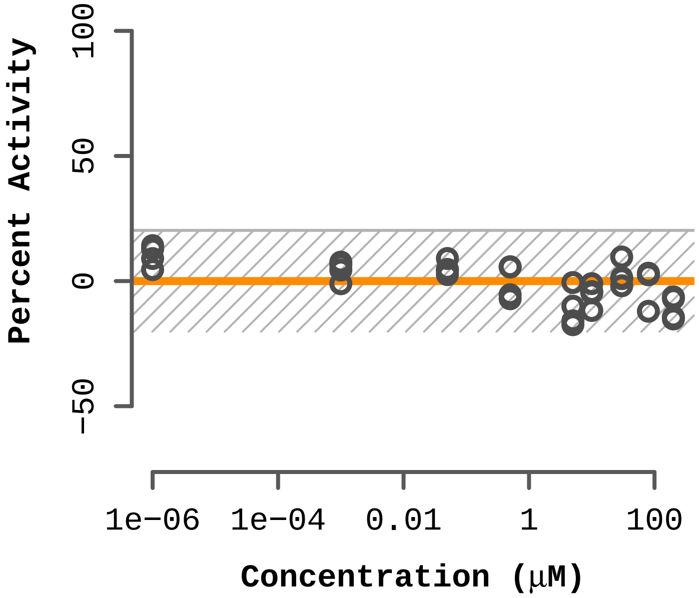

ASSAY: AEID3093 (CCTE_Deisenroth_DEVTOX_RUES2-GLR_Sox:

NAME: Saccharin
CHID: 21251 CASRN: 81-07-2
SPID(S): ES211_54_54
M4ID: 42675881

HILL MODEL: Not applicable.

GAIN-LOSS MODEL: Not applicable.

	CNST	HILL	GNLS
AIC:	260.05	NA	NA
PROB:	1	NA	NA
RMSE:	8.31	NA	NA

MAX_MEAN: 10.2 MAX_MED: 10.9 BMAD: 6.75

COFF: 20.3 HIT-CALL: 0 FITC: 4 ACTP: 0

FLAGS:

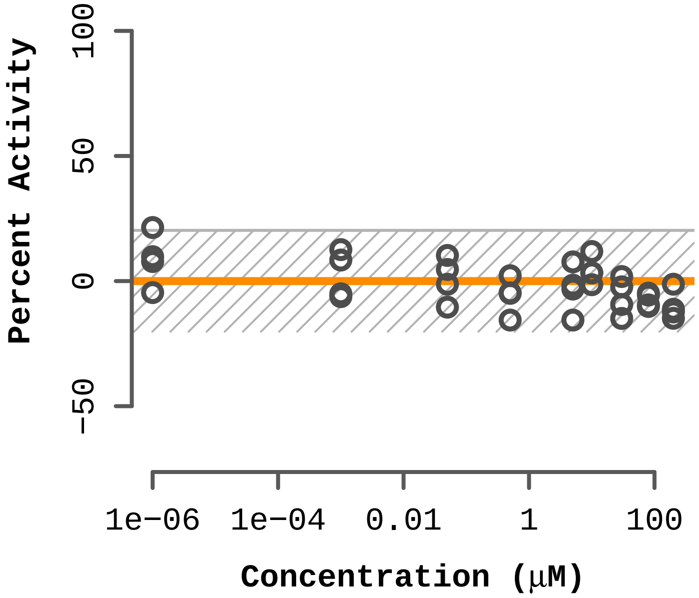

ASSAY: AEID3093 (CCTE_Deisenroth_DEVTOX_RUES2-GLR_Sox:

NAME: Sulfasalazine
CHID: 21256 CASRN: 599-79-1
SPID(S): ES211_59_59
M4ID: 42675897

HILL MODEL: Not applicable.

GAIN-LOSS MODEL: Not applicable.

	CNST	HILL	GNLS
AIC:	267.25	NA	NA
PROB:	1	NA	NA
RMSE:	9.14	NA	NA

MAX_MEAN: 8.6 MAX_MED: 8.79 BMAD: 6.75

COFF: 20.3 HIT-CALL: 0 FITC: 4 ACTP: 0

FLAGS:

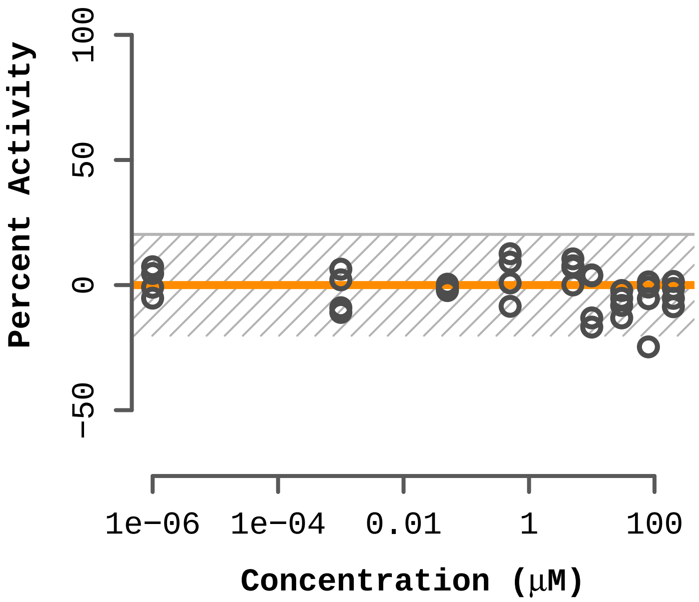

ASSAY: AEID3093 (CCTE_Deisenroth_DEVTOX_RUES2-GLR_Sox[illegible]

NAME: Dimethyl phthalate
CHID: 22455 CASRN: 131-11-3
SPID(S): ES211_30_30
M4ID: 42675847

HILL MODEL: Not applicable.

GAIN-LOSS MODEL: Not applicable.

	CNST	HILL	GNLS
AIC:	255.23	NA	NA
PROB:	1	NA	NA
RMSE:	8.16	NA	NA

MAX_MEAN: 6.35 MAX_MED: 7.43 BMAD: 6.75

COFF: 20.3 HIT-CALL: 0 FITC: 4 ACTP: 0

FLAGS:

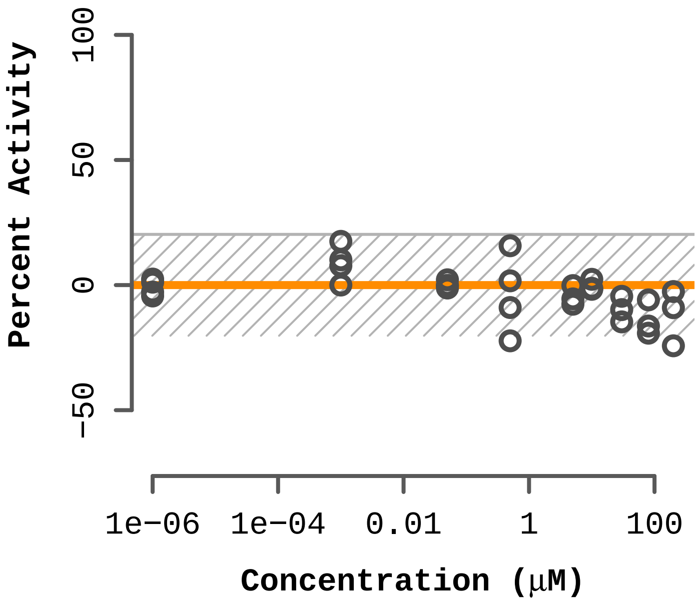

ASSAY: AEID3093 (CCTE_Deisenroth_DEVTOX_RUES2-GLR_Sox

NAME: Folic acid
CHID: 22519 CASRN: 59-30-3
SPID(S): ES211_33_33
M4ID: 42675891

HILL MODEL: Not applicable.

GAIN-LOSS MODEL: Not applicable.

	CNST	HILL	GNLS
AIC:	232.24	NA	NA
PROB:	1	NA	NA
RMSE:	10.02	NA	NA

MAX_MEAN: 8.84 MAX_MED: 8.92 BMAD: 6.75

COFF: 20.3 HIT-CALL: 0 FITC: 4 ACTP: 0

FLAGS:

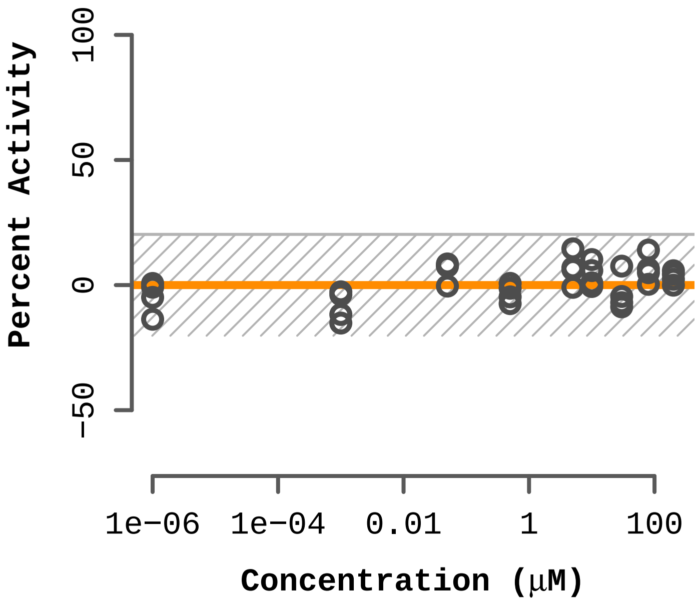

ASSAY: AEID3093 (CCTE_Deisenroth_DEVTOX_RUES2-GLR_Sox:

NAME: Acyclovir
CHID: 22556 CASRN: 59277-89-3
SPID(S): ES211_12_12
M4ID: 42675852

HILL MODEL: Not applicable.

GAIN-LOSS MODEL: Not applicable.

	CNST	HILL	GNLS
AIC:	248.4	NA	NA
PROB:	1	NA	NA
RMSE:	7.13	NA	NA

MAX_MEAN: 6.68 MAX_MED: 7.67 BMAD: 6.75

COFF: 20.3 HIT-CALL: 0 FITC: 4 ACTP: 0

FLAGS:

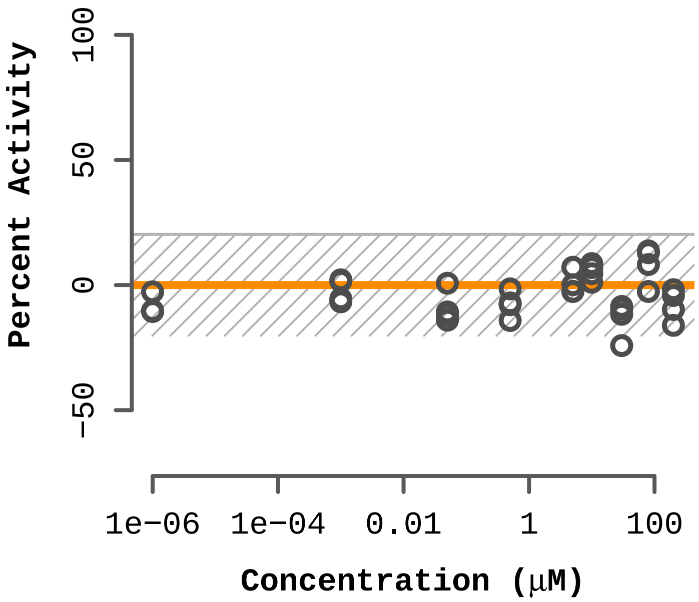

ASSAY: AEID3093 (CCTE_Deisenroth_DEVTOX_RUES2-GLR_Sox:

NAME: Stavudine
CHID: 23819 CASRN: 3056−17−5
SPID(S): ES211_58_58
M4ID: 42675890

HILL MODEL: Not applicable.

GAIN−LOSS MODEL: Not applicable.

	CNST	HILL	GNLS
AIC:	267.68	NA	NA
PROB:	1	NA	NA
RMSE:	9.25	NA	NA

MAX_MEAN: 8.07 MAX_MED: 10.6 BMAD: 6.75

COFF: 20.3 HIT−CALL: 0 FITC: 4 ACTP: 0

FLAGS:

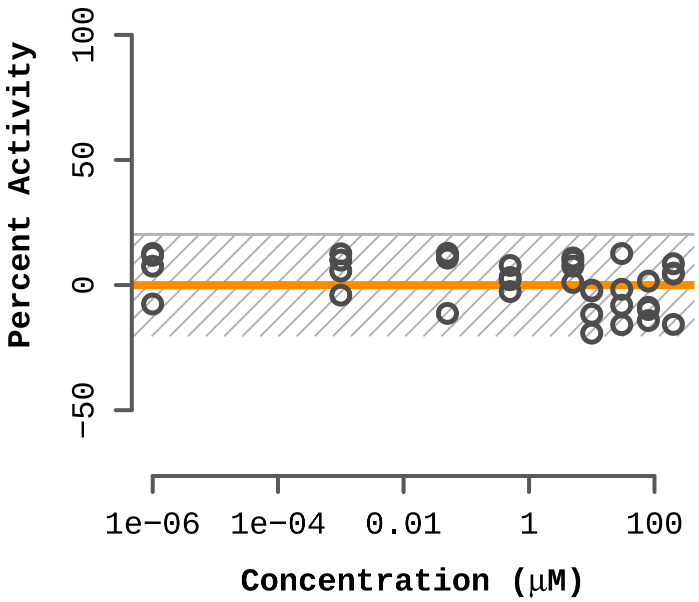

ASSAY: AEID3093 (CCTE_Deisenroth_DEVTOX_RUES2-GLR_Sox

NAME: D-Camphor
CHID: 24721 CASRN: 464-49-3
SPID(S): ES211_27_27
M4ID: 42675884

HILL MODEL: Not applicable.

GAIN-LOSS MODEL: Not applicable.

	CNST	HILL	GNLS
AIC:	273.16	NA	NA
PROB:	1	NA	NA
RMSE:	9.67	NA	NA

MAX_MEAN: 7.36 MAX_MED: 11.6 BMAD: 6.75

COFF: 20.3 HIT-CALL: 0 FITC: 4 ACTP: 0

FLAGS:

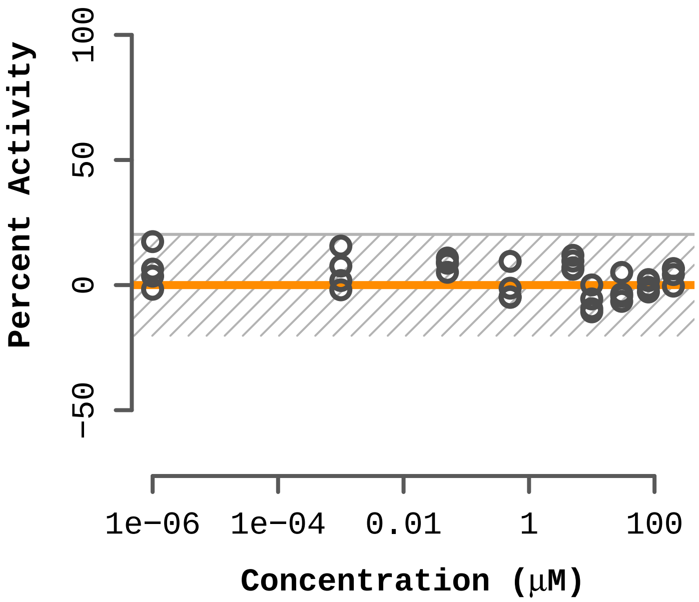

ASSAY: AEID3093 (CCTE_Deisenroth_DEVTOX_RUES2-GLR_Sox:

NAME: Cyclophosphamide monohydrate
CHID: 24888 CASRN: 6055−19−2
SPID(S): ES211_24_24
M4ID: 42675863

HILL MODEL: Not applicable.

GAIN−LOSS MODEL: Not applicable.

	CNST	HILL	GNLS
AIC:	249.82	NA	NA
PROB:	1	NA	NA
RMSE:	7.23	NA	NA

MAX_MEAN: 8.69 MAX_MED: 8.98 BMAD: 6.75

COFF: 20.3 HIT−CALL: 0 FITC: 4 ACTP: 0

FLAGS:

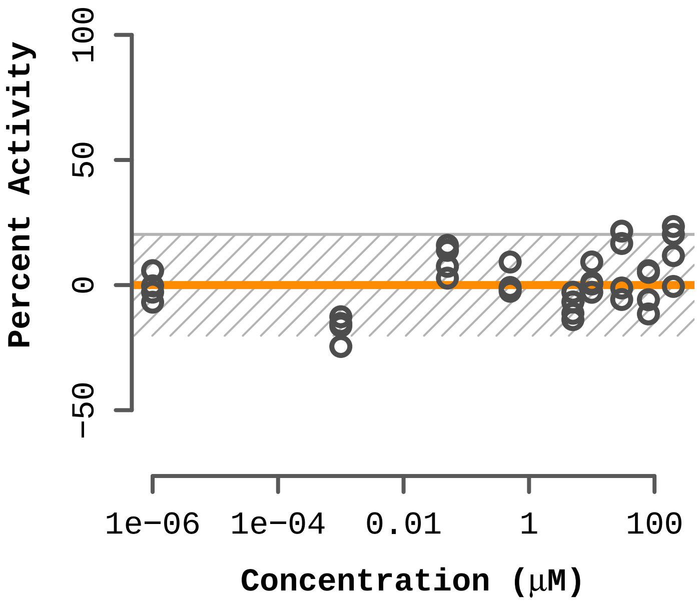

ASSAY: AEID3093 (CCTE_Deisenroth_DEVTOX_RUES2-GLR_Sox:

NAME: Hydroxyurea
CHID: 25438 CASRN: 127-07-1
SPID(S): ES211_35_35
M4ID: 42675845

HILL MODEL: Not applicable.

GAIN-LOSS MODEL: Not applicable.

	CNST	HILL	GNLS
AIC:	280.55	NA	NA
PROB:	1	NA	NA
RMSE:	11.22	NA	NA

MAX_MEAN: 13.7 MAX_MED: 16 BMAD: 6.75

COFF: 20.3 HIT-CALL: 0 FITC: 4 ACTP: 0

FLAGS:

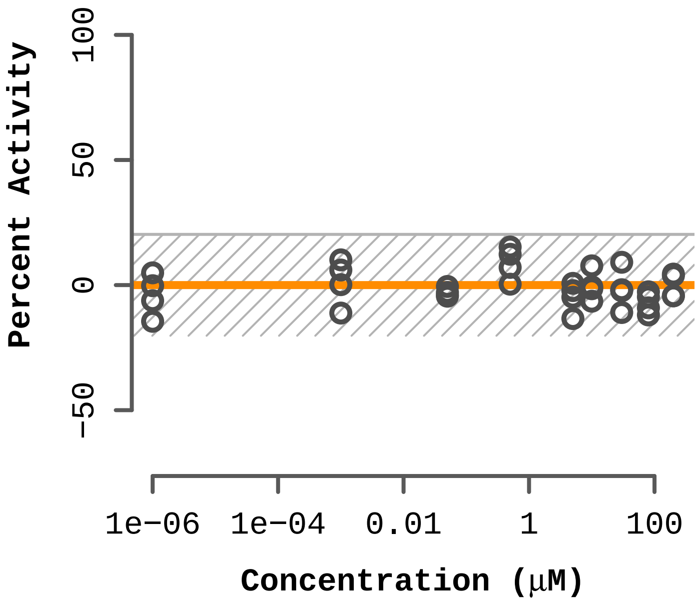

ASSAY: AEID3093 (CCTE_Deisenroth_DEVTOX_RUES2-GLR_Sox:

NAME: Salicylic acid
CHID: 26368 CASRN: 69-72-7
SPID(S): ES211_55_55
M4ID: 42675879

HILL MODEL: Not applicable.

GAIN-LOSS MODEL: Not applicable.

	CNST	HILL	GNLS
AIC:	248.94	NA	NA
PROB:	1	NA	NA
RMSE:	7.19	NA	NA

MAX_MEAN: 8.74 MAX_MED: 9.69 BMAD: 6.75

COFF: 20.3 HIT-CALL: 0 FITC: 4 ACTP: 0

FLAGS:

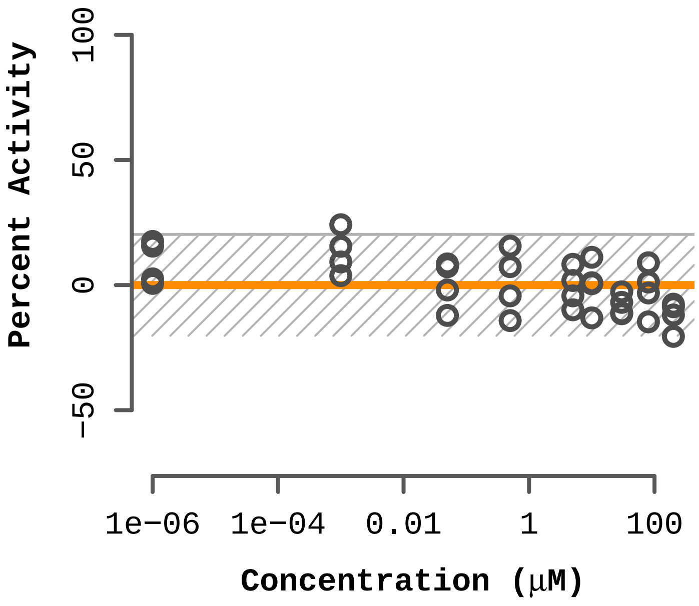

ASSAY: AEID3093 (CCTE_Deisenroth_DEVTOX_RUES2-GLR_Sox:

NAME: Penicillin G potassium
CHID: 45106 CASRN: 113−98−4
SPID(S): ES211_49_49
M4ID: 42675894

HILL MODEL: Not applicable.

GAIN−LOSS MODEL: Not applicable.

	CNST	HILL	GNLS
AIC:	278.39	NA	NA
PROB:	1	NA	NA
RMSE:	10.63	NA	NA

MAX_MEAN: 13.2 MAX_MED: 12.4 BMAD: 6.75

COFF: 20.3 HIT−CALL: 0 FITC: 4 ACTP: 0

FLAGS:

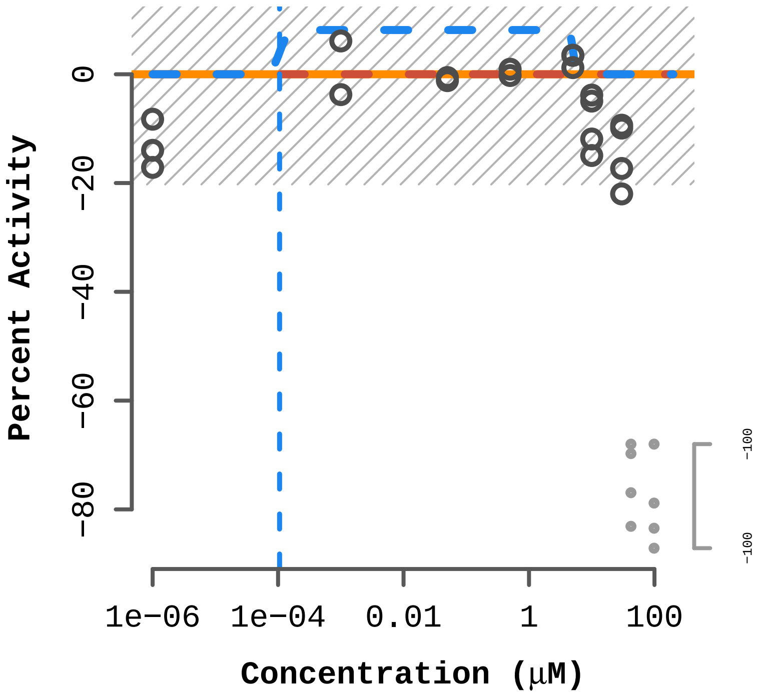

ASSAY: AEID3093 (CCTE_Deisenroth_DEVTOX_RUES2-GLR_Sox:

NAME: Bisphenol A
CHID: 20182 CASRN: 80-05-7
SPID(S): ES211_16_16
M4ID: 42675846 BRK

HILL MODEL (in red):

	tp	ga	gw
val:	0.853	2.8	7.98
sd:	NaN	NaN	NaN

GAIN-LOSS MODEL (in blue):

	tp	ga	gw	la	lw
val:	8.14	-3.98	6.59	0.705	17.4
sd:	7.31	994	6710	0.319	757

	CNST	HILL	GNLS
AIC:	377.01	383.01	385.7
PROB:	0.94	0.05	0.01
RMSE:	49.11	49.11	48.86

MAX_MEAN: 9.9 MAX_MED: 10.7 BMAD: 6.75

COFF: 20.3 HIT-CALL: 0 FITC: 7 ACTP: 0.06

FLAGS: 10

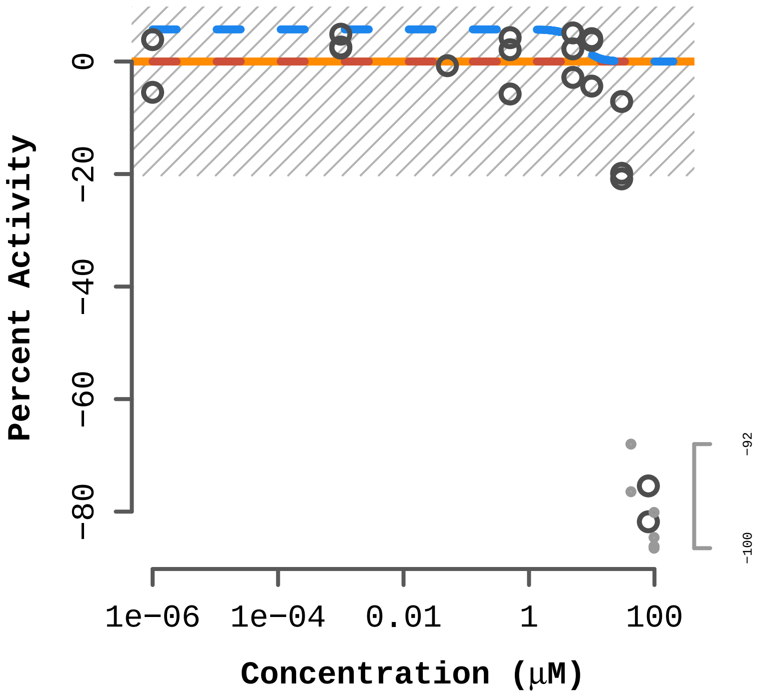

ASSAY: AEID3093 (CCTE_Deisenroth_DEVTOX_RUES2-GLR_Sox:

NAME: 4-Hydroxybenzoic acid butyl ester
CHID: 20209 CASRN: 94-26-8
SPID(S): ES211_19_19
M4ID: 42675858 BRK

HILL MODEL (in red):

	tp	ga	gw
val:	1.87e-05	2.79	4.58
sd:	NaN	NaN	NaN

GAIN-LOSS MODEL (in blue):

	tp	ga	gw	la	lw
val:	5.71	-6.54	7.75	0.827	3.21
sd:	NaN	NaN	NaN	NaN	NaN

	CNST	HILL	GNLS
AIC:	369.23	375.23	377.98
PROB:	0.94	0.05	0.01
RMSE:	45.27	45.27	45.09

MAX_MEAN: 8.67 MAX_MED: 10.2 BMAD: 6.75

COFF: 20.3 HIT-CALL: 0 FITC: 7 ACTP: 0.06

FLAGS: 10

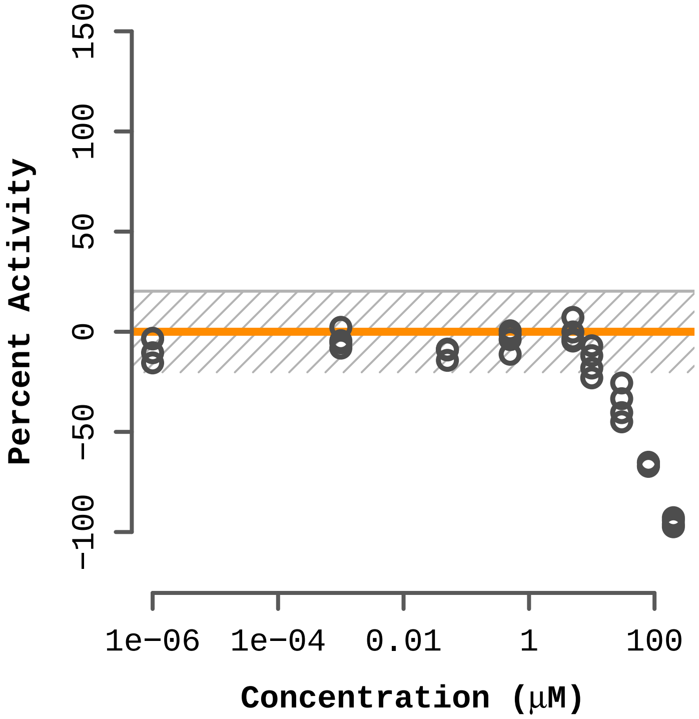

ASSAY: AEID3093 (CCTE_Deisenroth_DEVTOX_RUES2-GLR_Sox:

NAME: Diphenhydramine hydrochloride
CHID: 20537 CASRN: 147-24-0
SPID(S): ES211_31_31
M4ID: 42675880

HILL MODEL: Failed to converge.

GAIN-LOSS MODEL: Failed to converge.

	CNST	HILL	GNLS
AIC:	367.59	NA	NA
PROB:	1	NA	NA
RMSE:	41.28	NA	NA

MAX_MEAN: -0.267 MAX_MED: -1.82 BMAD: 6.75

COFF: 20.3 HIT-CALL: 0 FITC: 7 ACTP: 0

FLAGS: 10

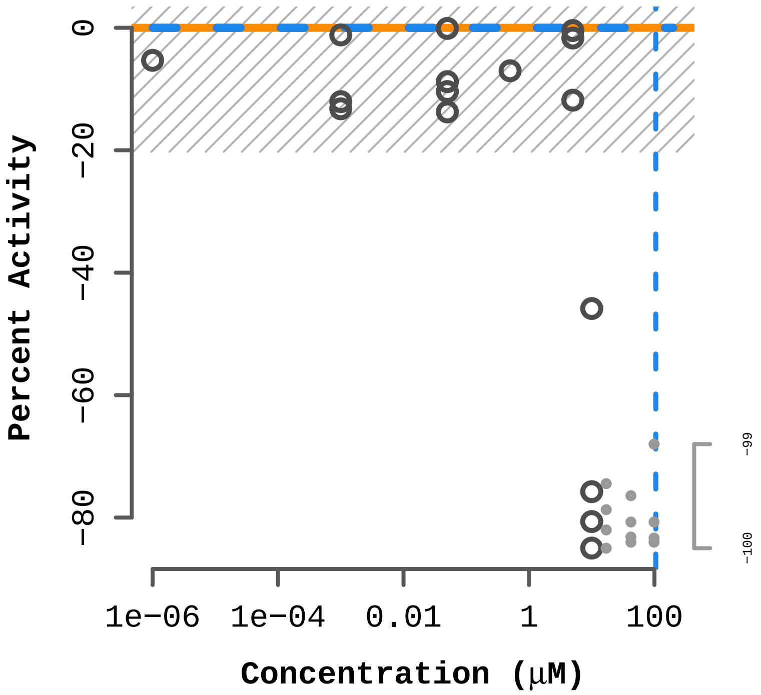

ASSAY: AEID3093 (CCTE_Deisenroth_DEVTOX_RUES2-GLR_Sox

NAME: 5-Fluorouracil
CHID: 20634 CASRN: 51-21-8
SPID(S): ES211_5_5
M4ID: 42675878 BRK

HILL MODEL (in red):

	tp	ga	gw
val:	1.77e-07	2.77	2.94
sd:	NaN	NaN	NaN

GAIN-LOSS MODEL (in blue):

	tp	ga	gw	la	lw
val:	9.09e-09	2.02	7.97	2.28	12.3
sd:	0.000332	7900	330000	1800	673000

	CNST	HILL	GNLS
AIC:	405.86	411.86	415.86
PROB:	0.95	0.05	0.01
RMSE:	63.9	63.9	63.9

MAX_MEAN: 3.38 MAX_MED: 4.48 BMAD: 6.75

COFF: 20.3 HIT-CALL: 0 FITC: 7 ACTP: 0.05

FLAGS: 10

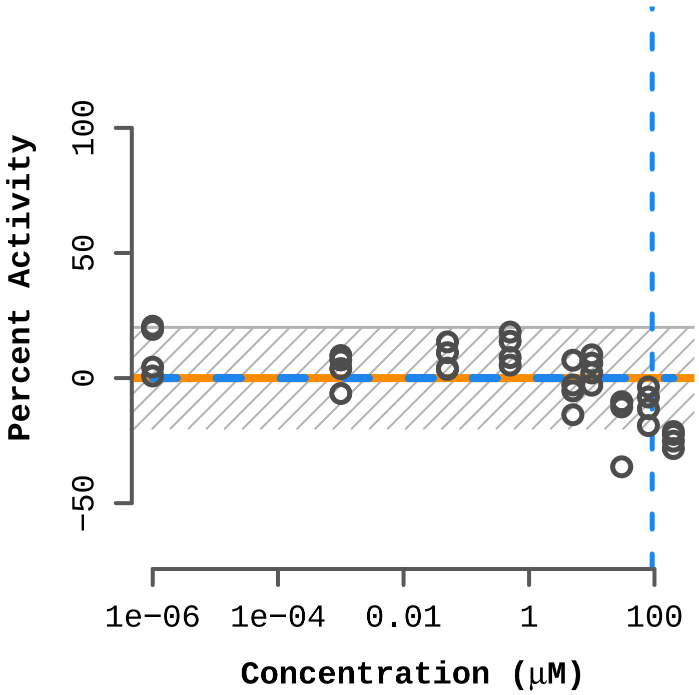

ASSAY: AEID3093 (CCTE_Deisenroth_DEVTOX_RUES2-GLR_Sox:

NAME: Ibuprofen
CHID: 20732 CASRN: 15687-27-1
SPID(S): ES211_36_36
M4ID: 42675896

HILL MODEL (in red):

	tp	ga	gw
val:	0.24	2.79	7.99
sd:	NaN	NaN	NaN

GAIN-LOSS MODEL (in blue):

	tp	ga	gw	la	lw
val:	5.18e-08	1.96	5.73	2.22	13.9
sd:	0.000318	1030	85000	1100	185000

	CNST	HILL	GNLS
AIC:	295.77	301.77	305.77
PROB:	0.95	0.05	0.01
RMSE:	13.92	13.92	13.92

MAX_MEAN: 11.6 MAX_MED: 11.9 BMAD: 6.75

COFF: 20.3 HIT-CALL: 0 FITC: 7 ACTP: 0.05

FLAGS:

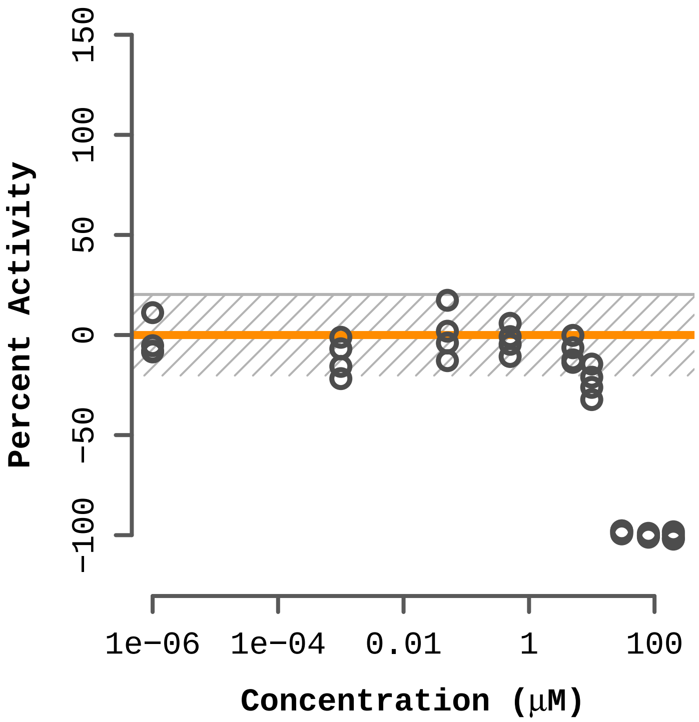

ASSAY: AEID3093 (CCTE_Deisenroth_DEVTOX_RUES2-GLR_Sox:

NAME: 1-(p-Chlorobenzoyl)-5-methoxy-2-methyl-Indole-:
CHID: 20740 CASRN: 53-86-1
SPID(S): ES211_38_38
M4ID: 42675889

HILL MODEL: Failed to converge.

GAIN-LOSS MODEL: Failed to converge.

	CNST	HILL	GNLS
AIC:	397.37	NA	NA
PROB:	1	NA	NA
RMSE:	58.6	NA	NA

MAX_MEAN: 0.625 MAX_MED: -1.03 BMAD: 6.75

COFF: 20.3 HIT-CALL: 0 FITC: 7 ACTP: 0

FLAGS: 10

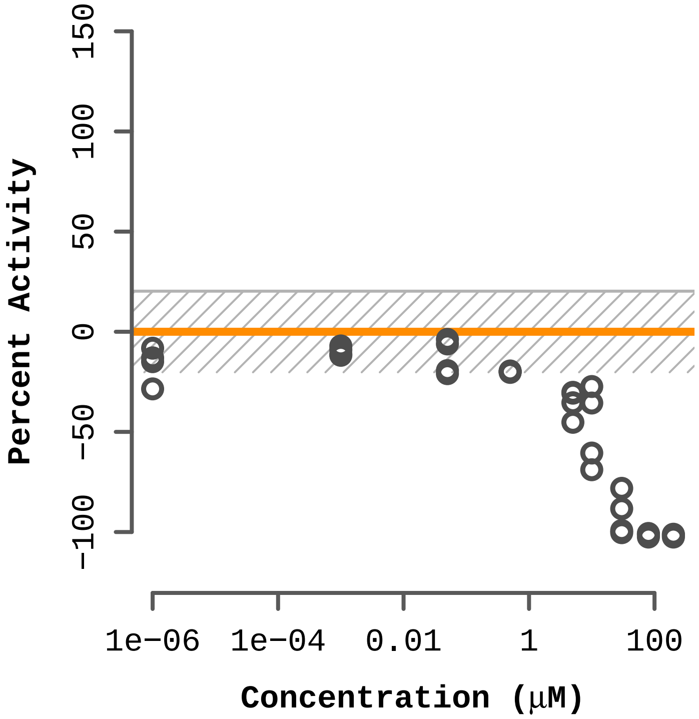

ASSAY: AEID3093 (CCTE_Deisenroth_DEVTOX_RUES2-GLR_Sox:

NAME: Lovastatin
CHID: 20784 CASRN: 75330-75-5
SPID(S): ES211_42_42
M4ID: 42675853

HILL MODEL: Failed to converge.

GAIN-LOSS MODEL: Failed to converge.

	CNST	HILL	GNLS
AIC:	403.65	NA	NA
PROB:	1	NA	NA
RMSE:	61.47	NA	NA

MAX_MEAN: -9.76 MAX_MED: -10.1 BMAD: 6.75

COFF: 20.3 HIT-CALL: 0 FITC: 7 ACTP: 0

FLAGS: 10

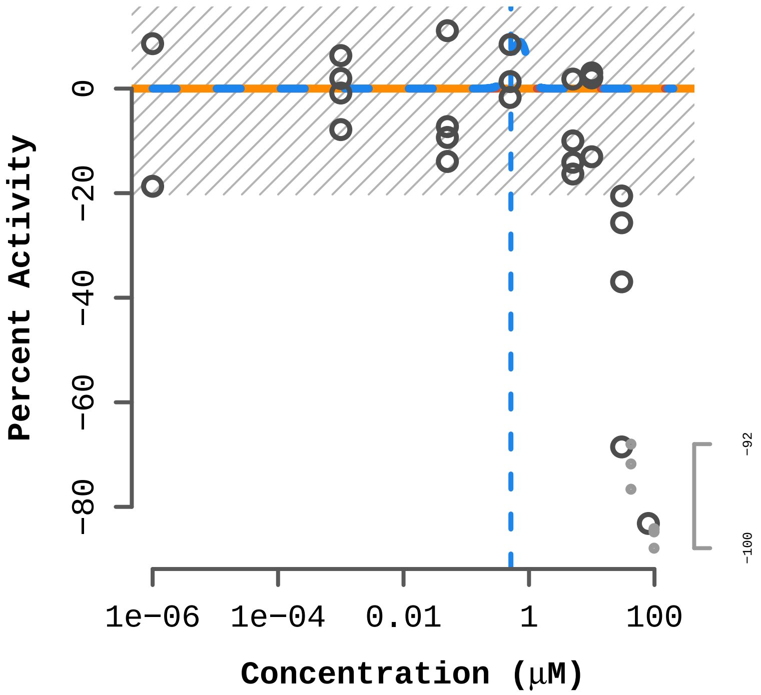

ASSAY: AEID3093 (CCTE_Deisenroth_DEVTOX_RUES2-GLR_Sox:

NAME: Busulfan
CHID: 20910 CASRN: 55-98-1
SPID(S): ES211_18_18
M4ID: 42675864 BRK

HILL MODEL (in red):

	tp	ga	gw
val:	0.298	2.8	7.93
sd:	NaN	NaN	NaN

GAIN-LOSS MODEL (in blue):

	tp	ga	gw	la	lw
val:	11.1	-0.289	6.04	-0.0228	8.54
sd:	2520	33.8	2030	92.6	1290

	CNST	HILL	GNLS
AIC:	378.91	384.91	388.79
PROB:	0.95	0.05	0.01
RMSE:	48.2	48.2	48.17

MAX_MEAN: 7.27 MAX_MED: 12.6 BMAD: 6.75

COFF: 20.3 HIT-CALL: 0 FITC: 7 ACTP: 0.05

FLAGS: 10

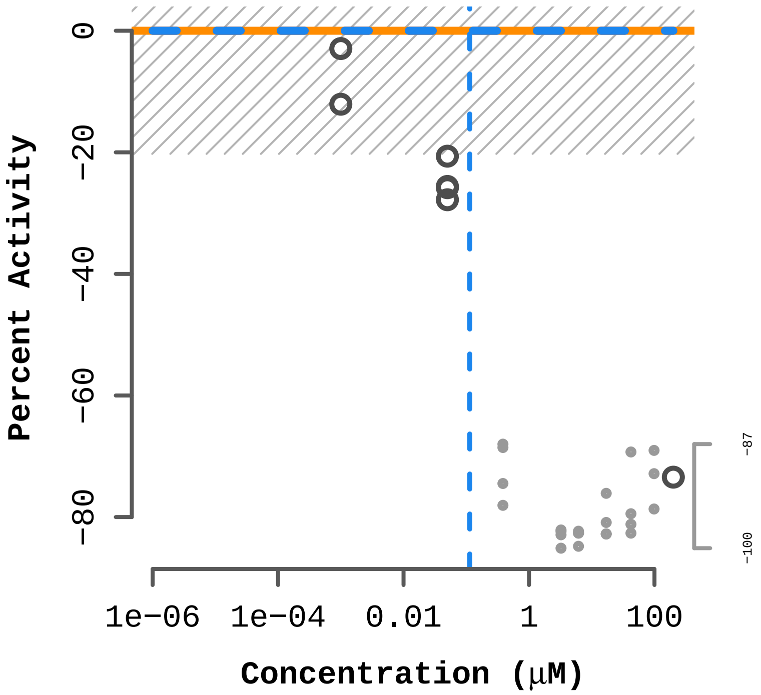

ASSAY: AEID3093 (CCTE_Deisenroth_DEVTOX_RUES2-GLR_Sox:

NAME: all-trans-Retinoic acid
CHID: 21239 CASRN: 302-79-4
SPID(S): ES211_13_13
M4ID: 42675895 BRK

HILL MODEL (in red):

	tp	ga	gw
val:	0.462	2.8	7.79
sd:	NaN	NaN	NaN

GAIN-LOSS MODEL (in blue):

	tp	ga	gw	la	lw
val:	0.00531	-0.945	5.84	-0.695	7.49
sd:	NaN	NaN	NaN	NaN	NaN

	CNST	HILL	GNLS
AIC:	425.46	431.46	435.46
PROB:	0.95	0.05	0.01
RMSE:	79.17	79.17	79.17

MAX_MEAN: 4.3 MAX_MED: 3.82 BMAD: 6.75

COFF: 20.3 HIT-CALL: 0 FITC: 7 ACTP: 0.05

FLAGS: 10

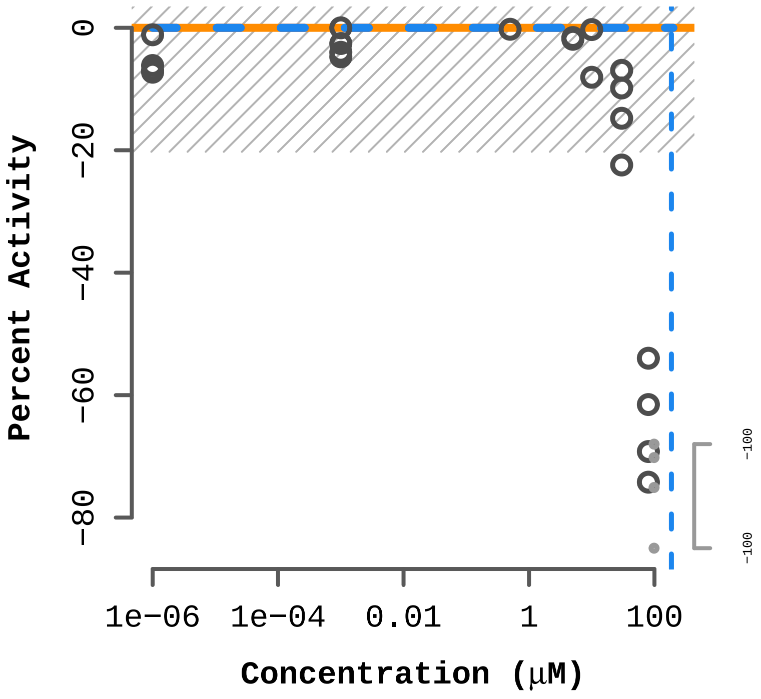

ASSAY: AEID3093 (CCTE_Deisenroth_DEVTOX_RUES2-GLR_Sox:

NAME: Rifampicin
CHID: 21244 CASRN: 13292-46-1
SPID(S): ES211_52_52
M4ID: 42675885 BRK

HILL MODEL (in red):

	tp	ga	gw
val:	2.85e-08	2.73	1.19
sd:	NaN	NaN	NaN

GAIN-LOSS MODEL (in blue):

	tp	ga	gw	la	lw
val:	6.63e-08	2.27	7.54	2.53	0.67
sd:	NaN	NaN	NaN	NaN	NaN

	CNST	HILL	GNLS
AIC:	359.69	365.69	369.69
PROB:	0.95	0.05	0.01
RMSE:	40.9	40.9	40.9

MAX_MEAN: 7.3 MAX_MED: 7.36 BMAD: 6.75

COFF: 20.3 HIT-CALL: 0 FITC: 7 ACTP: 0.05

FLAGS: 10

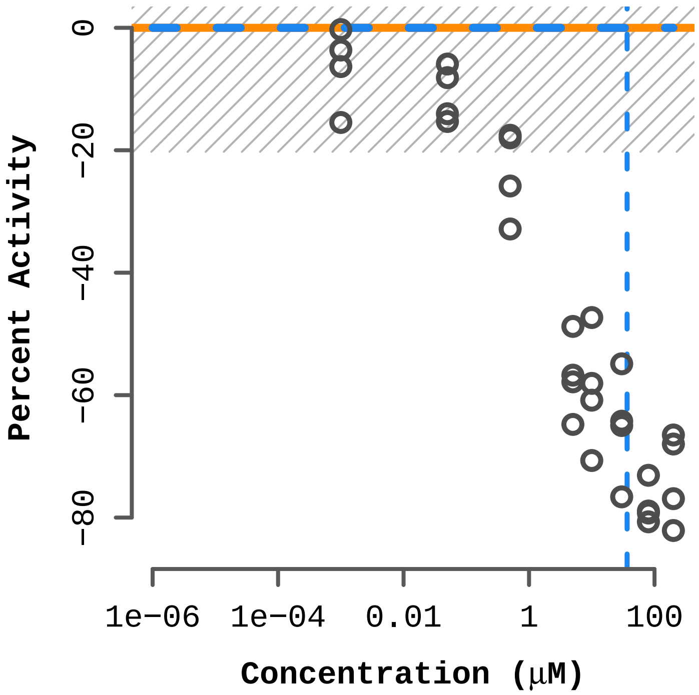

ASSAY: AEID3093 (CCTE_Deisenroth_DEVTOX_RUES2-GLR_Sox:

NAME: Thalidomide
CHID: 22524 CASRN: 50-35-1
SPID(S): ES211_62_62
M4ID: 42675868

HILL MODEL (in red):

	tp	ga	gw
val:	7.67e-07	2.69	2.67
sd:	NaN	NaN	NaN

GAIN-LOSS MODEL (in blue):

	tp	ga	gw	la	lw
val:	1.78e-08	1.56	4.16	1.83	5.38
sd:	0.000223	12800	328000	8690	494000

	CNST	HILL	GNLS
AIC:	392.55	398.55	402.55
PROB:	0.95	0.05	0.01
RMSE:	51.11	51.11	51.11

MAX_MEAN: 4.62 MAX_MED: 3.35 BMAD: 6.75

COFF: 20.3 HIT-CALL: 0 FITC: 7 ACTP: 0.05

FLAGS: 10

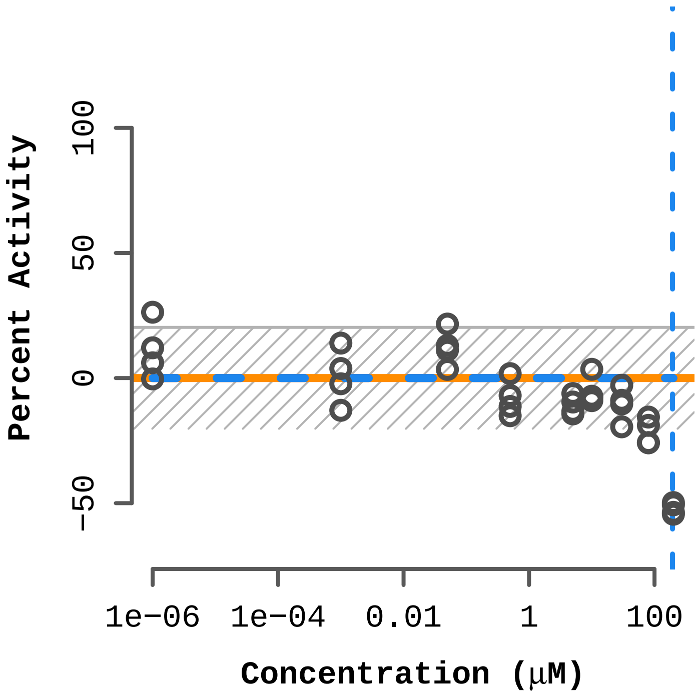

ASSAY: AEID3093 (CCTE_Deisenroth_DEVTOX_RUES2-GLR_Sox:

NAME: Carbamazepine
CHID: 22731 CASRN: 298-46-4
SPID(S): ES211_21_21
M4ID: 42675843

HILL MODEL (in red):

	tp	ga	gw
val:	13.3	2.8	7.98
sd:	NaN	NaN	NaN

GAIN-LOSS MODEL (in blue):

	tp	ga	gw	la	lw
val:	4.14e-09	2.29	7.81	2.69	11.8
sd:	NA	NA	NA	NA	NA

	CNST	HILL	GNLS
AIC:	321.73	327.73	331.73
PROB:	0.95	0.05	0.01
RMSE:	21.44	21.44	21.44

MAX_MEAN: 12.3 MAX_MED: 12.1 BMAD: 6.75

COFF: 20.3 HIT-CALL: 0 FITC: 7 ACTP: 0.05

FLAGS: 10

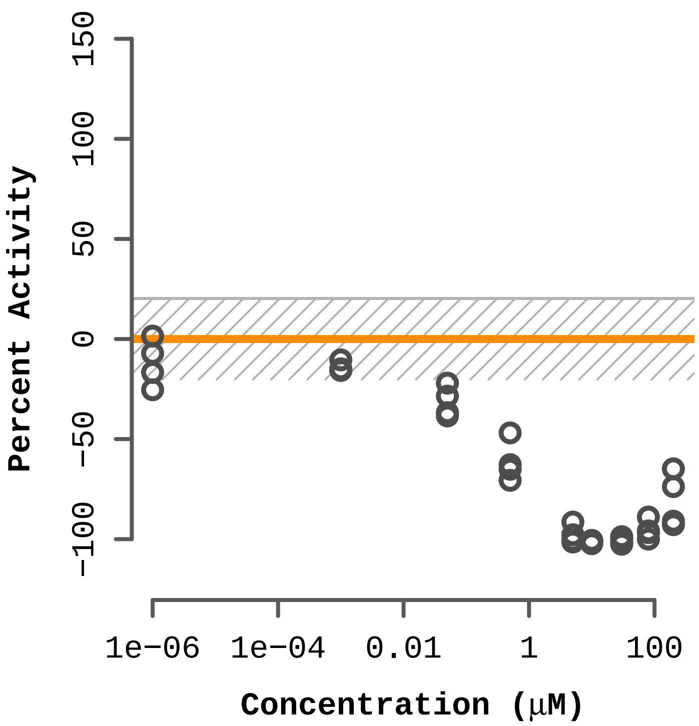

ASSAY: AEID3093 (CCTE_Deisenroth_DEVTOX_RUES2-GLR_Sox:

NAME: 13-cis Retinoic acid
CHID: 23177 CASRN: 4759-48-2
SPID(S): ES211_2_2
M4ID: 42675851

HILL MODEL: Failed to converge.

GAIN-LOSS MODEL: Failed to converge.

	CNST	HILL	GNLS
AIC:	421.64	NA	NA
PROB:	1	NA	NA
RMSE:	75.29	NA	NA

MAX_MEAN: -11.9 MAX_MED: -11.9 BMAD: 6.75

COFF: 20.3 HIT-CALL: 0 FITC: 7 ACTP: 0

FLAGS: 10

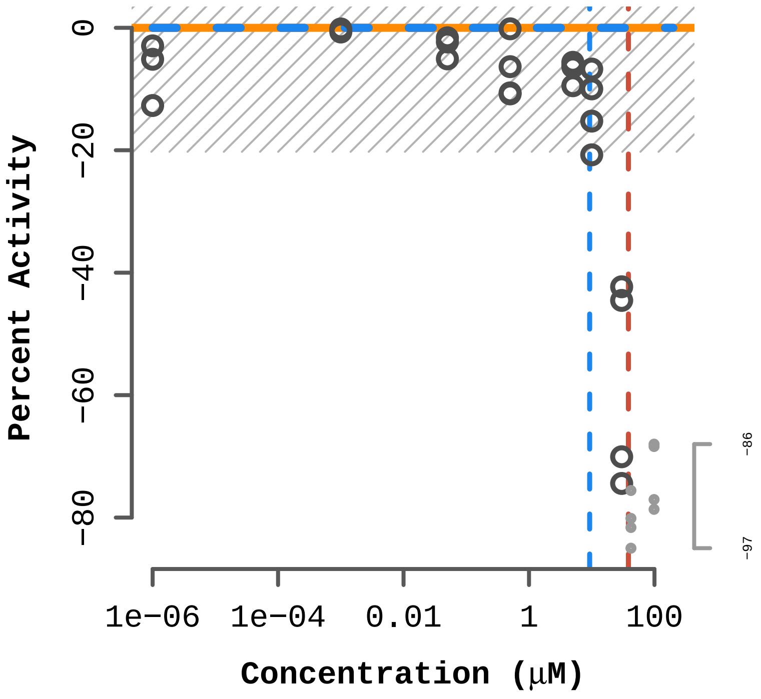

ASSAY: AEID3093 (CCTE_Deisenroth_DEVTOX_RUES2-GLR_Sox:

NAME: Retinol
CHID: 23556 CASRN: 68-26-8
SPID(S): ES211_51_51
M4ID: 42675861 BRK

HILL MODEL (in red):

	tp	ga	gw
val:	1.8e-08	1.58	0.926
sd:	NaN	NaN	NaN

GAIN-LOSS MODEL (in blue):

	tp	ga	gw	la	lw
val:	2.52e-08	0.967	2.08	1.45	5.79
sd:	NaN	NaN	NaN	NaN	NaN

	CNST	HILL	GNLS
AIC:	380.89	386.89	390.89
PROB:	0.95	0.05	0.01
RMSE:	48.22	48.22	48.22

MAX_MEAN: 1.1 MAX_MED: 1.06 BMAD: 6.75

COFF: 20.3 HIT-CALL: 0 FITC: 7 ACTP: 0.05

FLAGS: 10

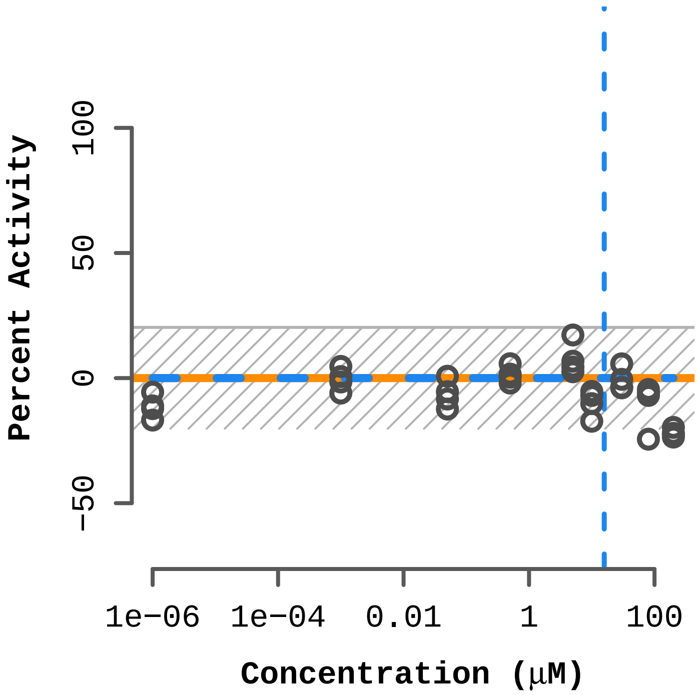

ASSAY: AEID3093 (CCTE_Deisenroth_DEVTOX_RUES2-GLR_Sox

NAME: Valproic acid
CHID: 23733 CASRN: 99-66-1
SPID(S): ES211_63_63
M4ID: 42675869

HILL MODEL (in red):

	tp	ga	gw
val:	9.92e-07	2.79	3.8
sd:	NaN	NaN	NaN

GAIN-LOSS MODEL (in blue):

	tp	ga	gw	la	lw
val:	9.32e-09	1.2	3.93	1.53	6.08
sd:	NaN	NaN	NaN	NaN	NaN

	CNST	HILL	GNLS
AIC:	278.68	284.68	288.68
PROB:	0.95	0.05	0.01
RMSE:	11.16	11.16	11.16

MAX_MEAN: 7.73 MAX_MED: 5.59 BMAD: 6.75

COFF: 20.3 HIT-CALL: 0 FITC: 7 ACTP: 0.05

FLAGS:

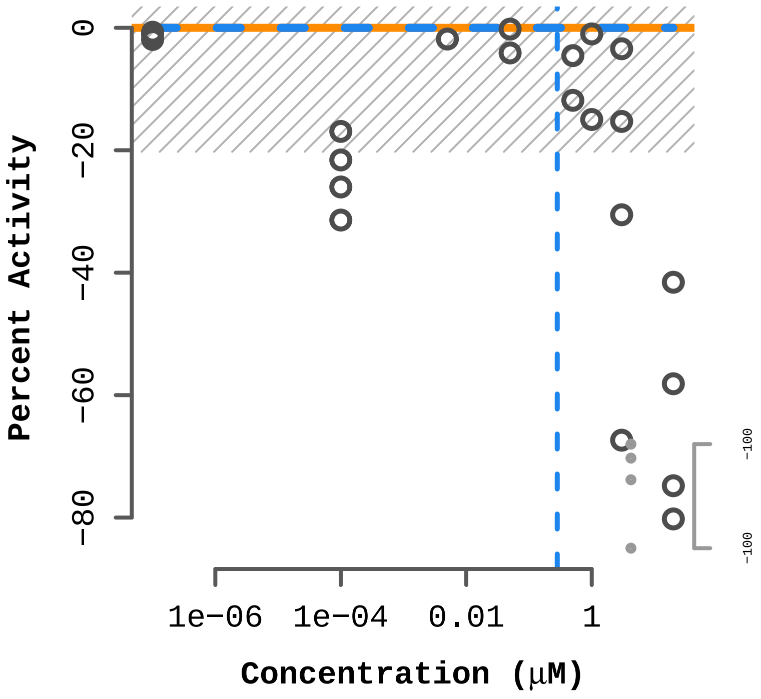

ASSAY: AEID3093 (CCTE_Deisenroth_DEVTOX_RUES2-GLR_Sox:

NAME: Ziprasidone
CHID: 23753 CASRN: 146939-27-7
SPID(S): ES211_66_66
M4ID: 42675836 BRK

HILL MODEL (in red):

	tp	ga	gw
val:	2.3e-07	1.78	1.34
sd:	NaN	NaN	NaN

GAIN-LOSS MODEL (in blue):

	tp	ga	gw	la	lw
val:	3.43e-08	-0.55	2.64	-0.155	5.69
sd:	0.0116	34600	254000	40600	1390000

	CNST	HILL	GNLS
AIC:	368.85	374.85	378.85
PROB:	0.95	0.05	0.01
RMSE:	43.15	43.15	43.15

MAX_MEAN: 3.27 MAX_MED: 4.35 BMAD: 6.75

COFF: 20.3 HIT-CALL: 0 FITC: 7 ACTP: 0.05

FLAGS: 10

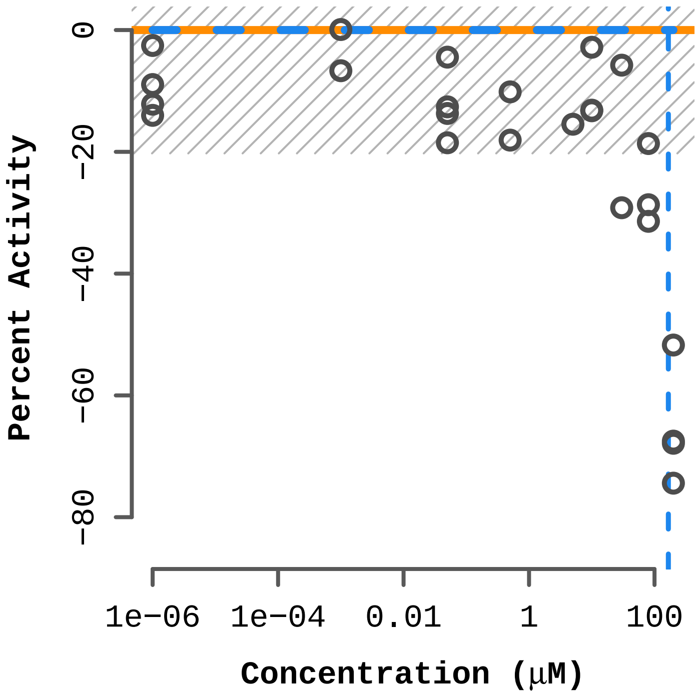

ASSAY: AEID3093 (CCTE_Deisenroth_DEVTOX_RUES2-GLR_Sox:

NAME: MEHP
CHID: 25680 CASRN: 4376-20-9
SPID(S): ES211_44_44
M4ID: 42675839

HILL MODEL (in red):

	tp	ga	gw
val:	0.352	2.8	7.98
sd:	NaN	NaN	NaN

GAIN-LOSS MODEL (in blue):

	tp	ga	gw	la	lw
val:	2.32e-08	2.22	4.89	3.93	9.84
sd:	NA	NA	NA	NA	NA

	CNST	HILL	GNLS
AIC:	330.17	336.17	340.17
PROB:	0.95	0.05	0.01
RMSE:	25.62	25.62	25.62

MAX_MEAN: 6.64 MAX_MED: 12.4 BMAD: 6.75

COFF: 20.3 HIT-CALL: 0 FITC: 7 ACTP: 0.05

FLAGS: 10

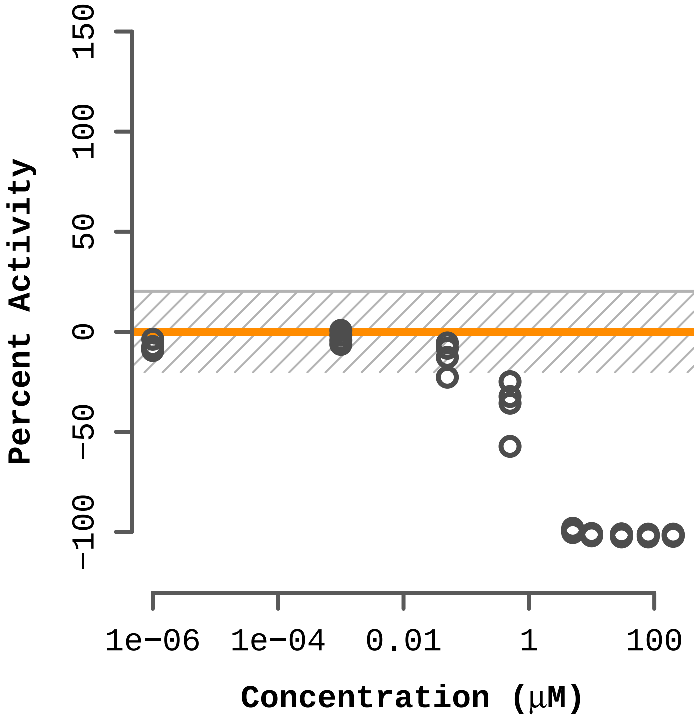

ASSAY: AEID3093 (CCTE_Deisenroth_DEVTOX_RUES2-GLR_Sox

NAME: 5-Bromo-2'-deoxyuridine
CHID: 33105 CASRN: 59-14-3
SPID(S): ES211_4_4
M4ID: 42675882

HILL MODEL: Failed to converge.

GAIN-LOSS MODEL: Failed to converge.

	CNST	HILL	GNLS
AIC:	448.66	NA	NA
PROB:	1	NA	NA
RMSE:	76.67	NA	NA

MAX_MEAN: -2.88 MAX_MED: -2.98 BMAD: 6.75

COFF: 20.3 HIT-CALL: 0 FITC: 7 ACTP: 0

FLAGS: 10

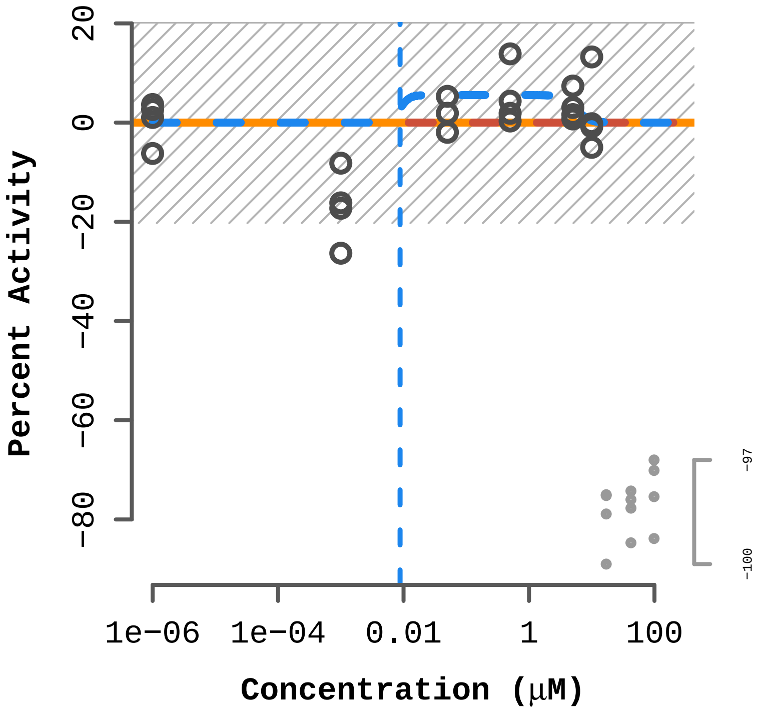

ASSAY: AEID3093 (CCTE_Deisenroth_DEVTOX_RUES2-GLR_Sox

NAME: Amiodarone hydrochloride
CHID: 37185 CASRN: 19774-82-4
SPID(S): ES211_14_14
M4ID: 42675849 BRK

HILL MODEL (in red):

	tp	ga	gw
val:	15.2	2.8	7.96
sd:	NaN	NaN	NaN

GAIN-LOSS MODEL (in blue):

	tp	ga	gw	la	lw
val:	5.55	-2.06	5.94	0.75	4.21
sd:	13.9	85.8	574	1.76	26.6

	CNST	HILL	GNLS
AIC:	396.09	402.09	405.89
PROB:	0.95	0.05	0.01
RMSE:	58.16	58.16	58.09

MAX_MEAN: 6.18 MAX_MED: 3.59 BMAD: 6.75

COFF: 20.3 HIT-CALL: 0 FITC: 7 ACTP: 0.05

FLAGS: 10

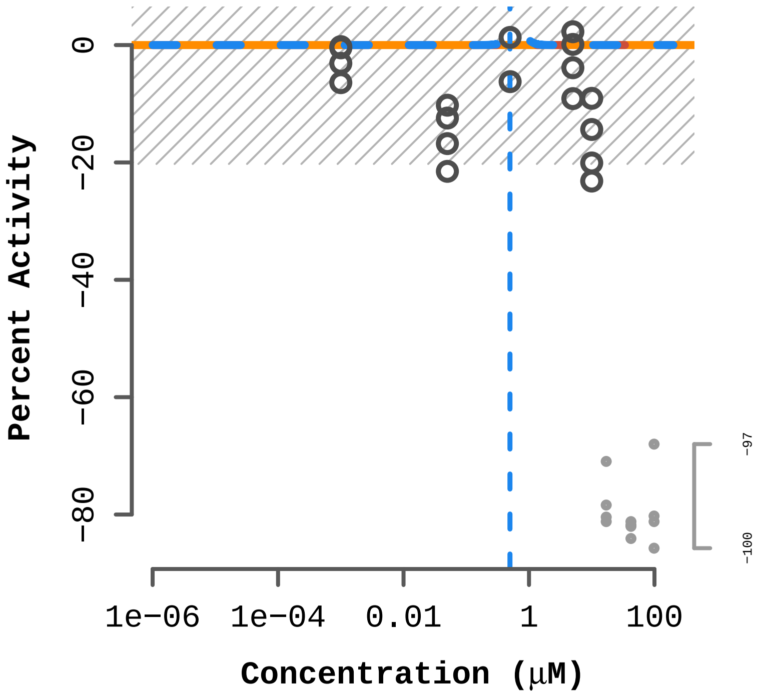

ASSAY: AEID3093 (CCTE_Deisenroth_DEVTOX_RUES2-GLR_Sox:

NAME: Imatinib mesylate
CHID: 40502 CASRN: 220127-57-1
SPID(S): ES211_37_37
M4ID: 42675892 BRK

HILL MODEL (in red):

	tp	ga	gw
val:	0.157	2.8	7.85
sd:	NaN	NaN	NaN

GAIN-LOSS MODEL (in blue):

	tp	ga	gw	la	lw
val:	2.8	-0.304	5.68	-0.0514	6.72
sd:	NaN	NaN	NaN	NaN	NaN

	CNST	HILL	GNLS
AIC:	396.96	402.96	406.95
PROB:	0.95	0.05	0.01
RMSE:	58.73	58.73	58.73

MAX_MEAN: 7.83 MAX_MED: 8.88 BMAD: 6.75

COFF: 20.3 HIT-CALL: 0 FITC: 7 ACTP: 0.05

FLAGS: 10

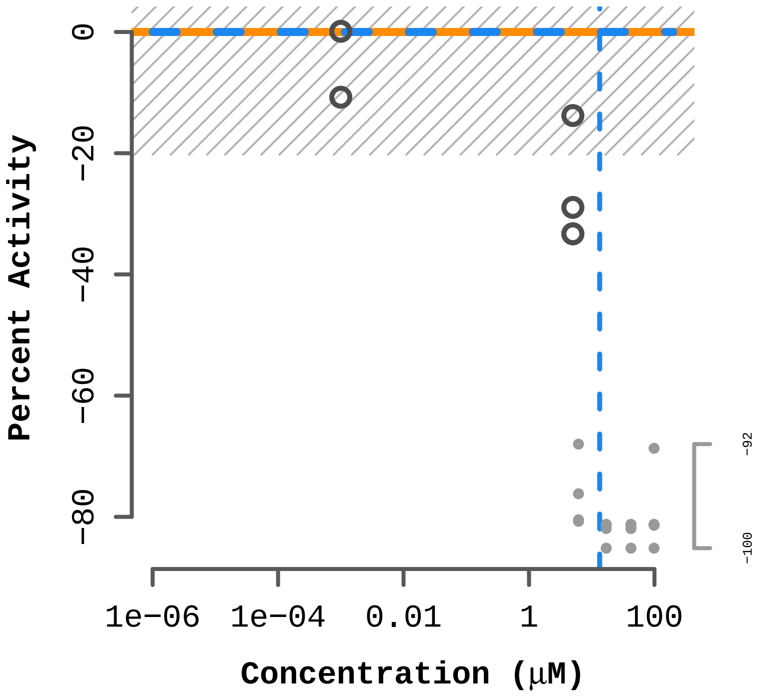

ASSAY: AEID3093 (CCTE_Deisenroth_DEVTOX_RUES2-GLR_Sox:

NAME: Gefitinib
CHID: 41034 CASRN: 184475-35-2
SPID(S): ES211_34_34
M4ID: 42675876 BRK

HILL MODEL (in red):

	tp	ga	gw
val:	0.656	2.8	7.91
sd:	NaN	NaN	NaN

GAIN-LOSS MODEL (in blue):

	tp	ga	gw	la	lw
val:	1.64e-07	1.13	7.19	1.38	6.88
sd:	NaN	NaN	NaN	NaN	NaN

	CNST	HILL	GNLS
AIC:	411.1	417.1	421.1
PROB:	0.95	0.05	0.01
RMSE:	67.66	67.66	67.66

MAX_MEAN: 10.5 MAX_MED: 7.83 BMAD: 6.75

COFF: 20.3 HIT-CALL: 0 FITC: 7 ACTP: 0.05

FLAGS: 10

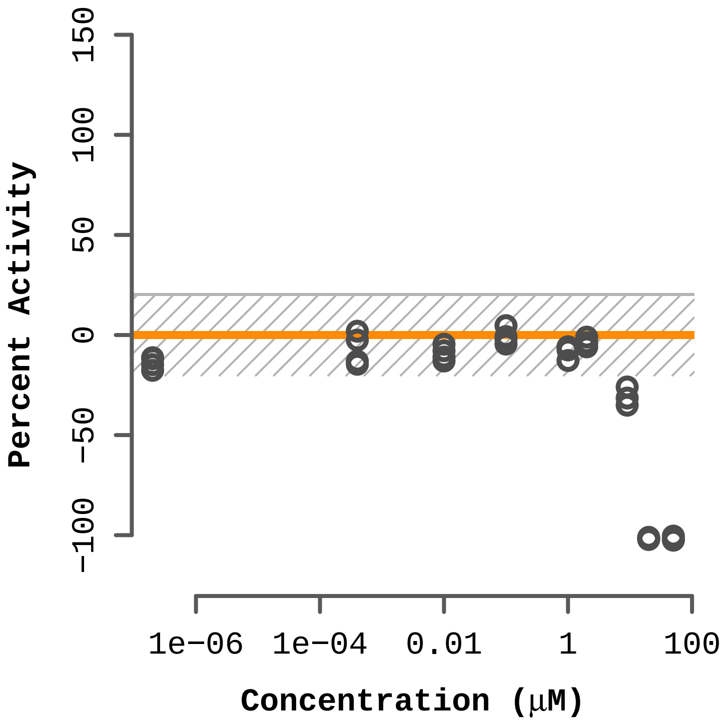

ASSAY: AEID3093 (CCTE_Deisenroth_DEVTOX_RUES2-GLR_Sox:

NAME: Ritanserin
CHID: 42594 CASRN: 87051-43-2
SPID(S): ES211_53_53
M4ID: 42675856

HILL MODEL: Failed to converge.

GAIN-LOSS MODEL: Failed to converge.

	CNST	HILL	GNLS
AIC:	319.97	NA	NA
PROB:	1	NA	NA
RMSE:	46.41	NA	NA

MAX_MEAN: -0.644 MAX_MED: -1.38 BMAD: 6.75

COFF: 20.3 HIT-CALL: 0 FITC: 7 ACTP: 0

FLAGS: 10

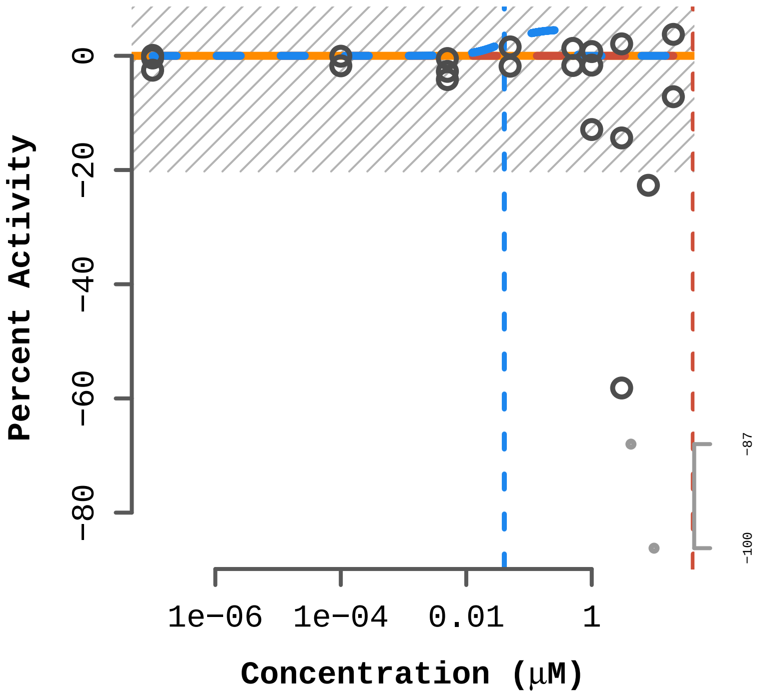

ASSAY: AEID3093 (CCTE_Deisenroth_DEVTOX_RUES2-GLR_Sox:

NAME: Cyclopamine
CHID: 43709 CASRN: 4449-51-8
SPID(S): ES211_23_23
M4ID: 42675838 BRK

HILL MODEL (in red):

	tp	ga	gw
val:	4.15e-08	1.62	1.63
sd:	NaN	NaN	NaN

GAIN-LOSS MODEL (in blue):

	tp	ga	gw	la	lw
val:	4.67	-1.39	1.72	-0.304	17
sd:	296	36.3	54	2.85	632

	CNST	HILL	GNLS
AIC:	261.4	267.4	270.6
PROB:	0.94	0.05	0.01
RMSE:	26.99	26.99	26.96

MAX_MEAN: 3.34 MAX_MED: 3.36 BMAD: 6.75

COFF: 20.3 HIT-CALL: 0 FITC: 7 ACTP: 0.06

FLAGS: 10

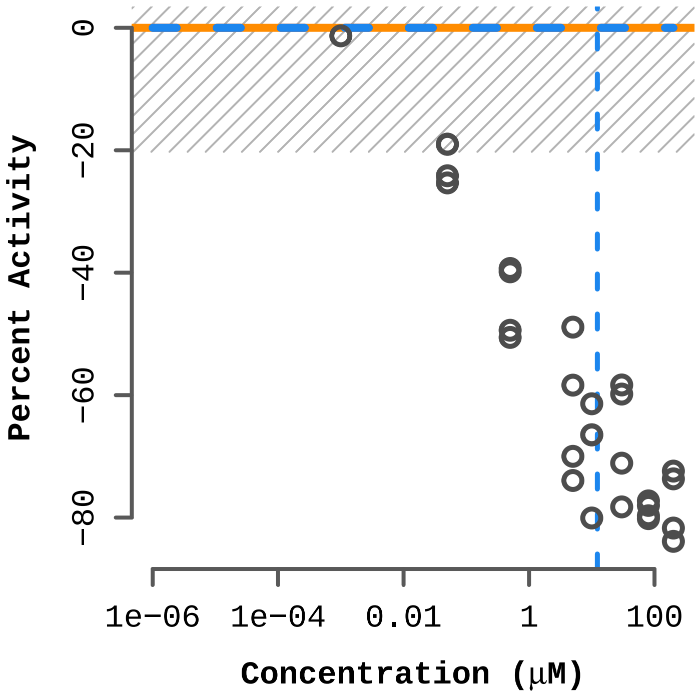

ASSAY: AEID3093 (CCTE_Deisenroth_DEVTOX_RUES2-GLR_Sox:

NAME: Lenalidomide
CHID: 46664 CASRN: 191732-72-6
SPID(S): ES211_41_41
M4ID: 42675877

HILL MODEL (in red):

	tp	ga	gw
val:	0.00239	2.78	7.08
sd:	NaN	NaN	NaN

GAIN-LOSS MODEL (in blue):

	tp	ga	gw	la	lw
val:	3.4e-07	1.09	5.42	1.35	13.6
sd:	NaN	NaN	NaN	NaN	NaN

	CNST	HILL	GNLS
AIC:	400.18	406.18	410.18
PROB:	0.95	0.05	0.01
RMSE:	56.09	56.09	56.09

MAX_MEAN: 10.3 MAX_MED: 9.38 BMAD: 6.75

COFF: 20.3 HIT-CALL: 0 FITC: 7 ACTP: 0.05

FLAGS: 10

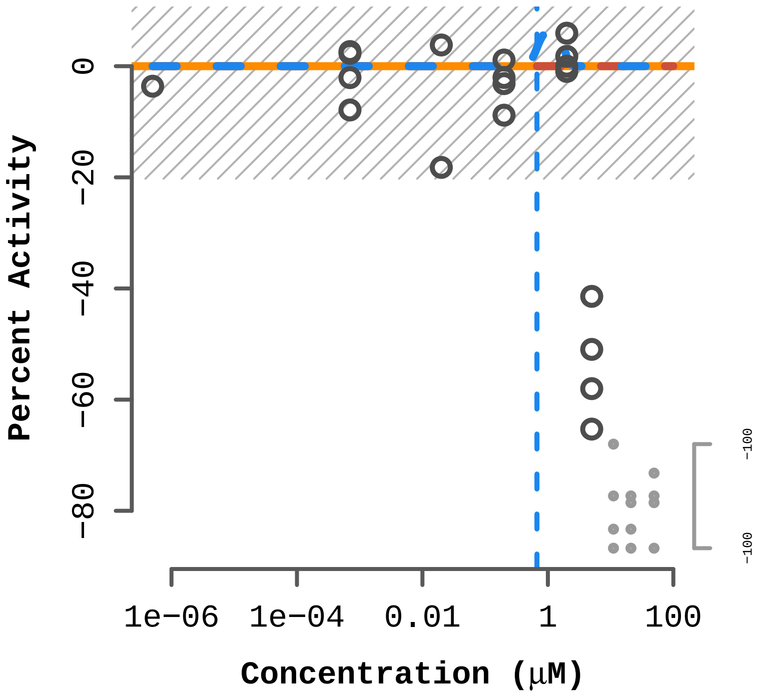

ASSAY: AEID3093 (CCTE_Deisenroth_DEVTOX_RUES2-GLR_Sox:

NAME: Vandetanib
CHID: 46681 CASRN: 443913-73-3
SPID(S): ES211_64_64
M4ID: 42675848 BRK

HILL MODEL (in red):

	tp	ga	gw
val:	0.288	2.5	7.88
sd:	NaN	NaN	NaN

GAIN-LOSS MODEL (in blue):

	tp	ga	gw	la	lw
val:	6.57	-0.174	7.48	0.264	12.4
sd:	1630	56.3	803	12.8	812

	CNST	HILL	GNLS
AIC:	402.56	408.56	412.56
PROB:	0.95	0.05	0.01
RMSE:	61.64	61.64	61.63

MAX_MEAN: 7.33 MAX_MED: 9.78 BMAD: 6.75

COFF: 20.3 HIT-CALL: 0 FITC: 7 ACTP: 0.05

FLAGS: 10

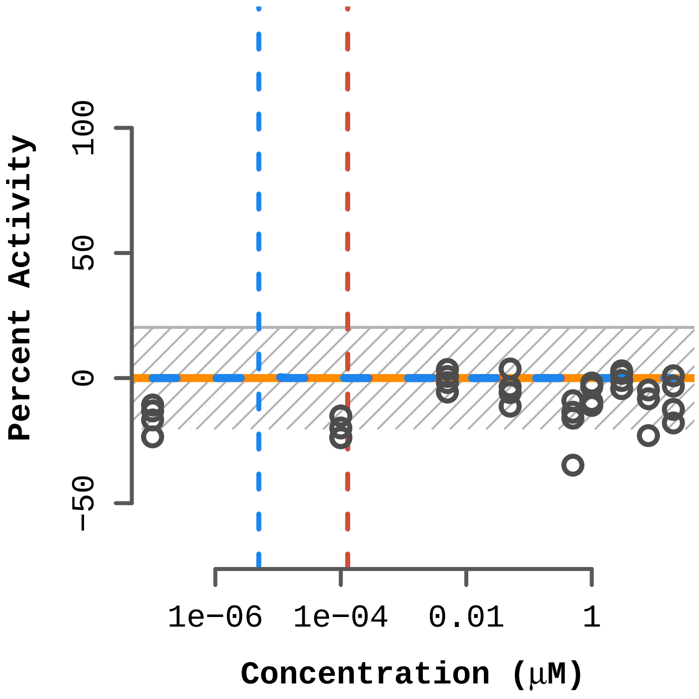

ASSAY: AEID3093 (CCTE_Deisenroth_DEVTOX_RUES2-GLR_Sox:

NAME: Dexamethasone sodium phosphate
CHID: 47429 CASRN: 2392-39-4
SPID(S): ES211_28_28
M4ID: 42675857

HILL MODEL (in red):

	tp	ga	gw
val:	2.26e-10	-3.89	1.54
sd:	4.43e-05	27300	390000

GAIN-LOSS MODEL (in blue):

	tp	ga	gw	la	lw
val:	0.945	-5.31	4.14	-5	6.91
sd:	NaN	NaN	NaN	NaN	NaN

	CNST	HILL	GNLS
AIC:	283.94	289.94	293.94
PROB:	0.95	0.05	0.01
RMSE:	13.32	13.32	13.32

MAX_MEAN: -0.0942 MAX_MED: 0.457 BMAD: 6.75

COFF: 20.3 HIT-CALL: 0 FITC: 7 ACTP: 0.05

FLAGS:

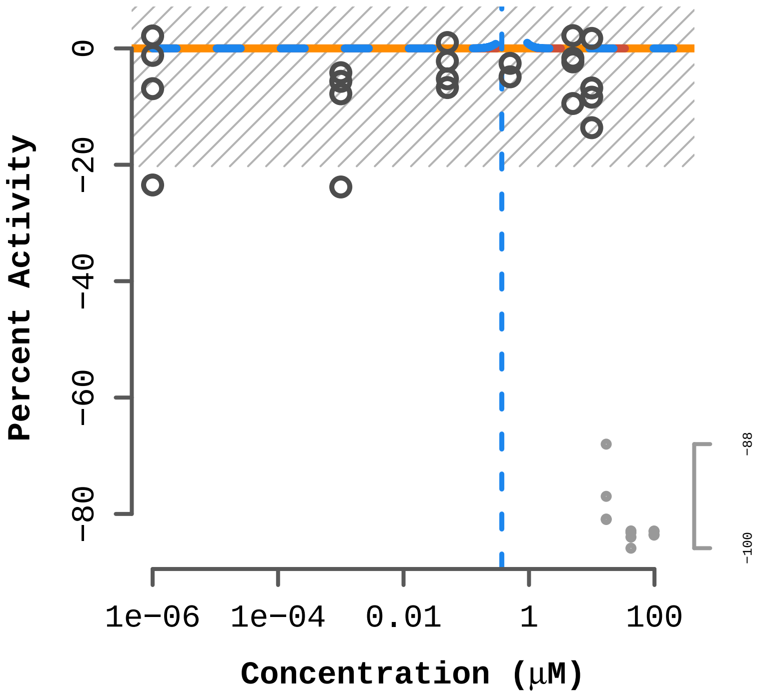

ASSAY: AEID3093 (CCTE_Deisenroth_DEVTOX_RUES2-GLR_Sox:

NAME: 6-aminopyridine-3-carboxamide
CHID: 51446 CASRN: 329-89-5
SPID(S): ES211_7_7
M4ID: 42675865 BRK

HILL MODEL (in red):

	tp	ga	gw
val:	3.1e-05	2.75	5.36
sd:	NaN	NaN	NaN

GAIN-LOSS MODEL (in blue):

	tp	ga	gw	la	lw
val:	3.37	-0.434	5.33	-0.0899	6.49
sd:	406	50	572	76.8	1110

	CNST	HILL	GNLS
AIC:	395.36	401.36	405.34
PROB:	0.95	0.05	0.01
RMSE:	57.69	57.69	57.69

MAX_MEAN: 2.7 MAX_MED: 3 BMAD: 6.75

COFF: 20.3 HIT-CALL: 0 FITC: 7 ACTP: 0.05

FLAGS: 10

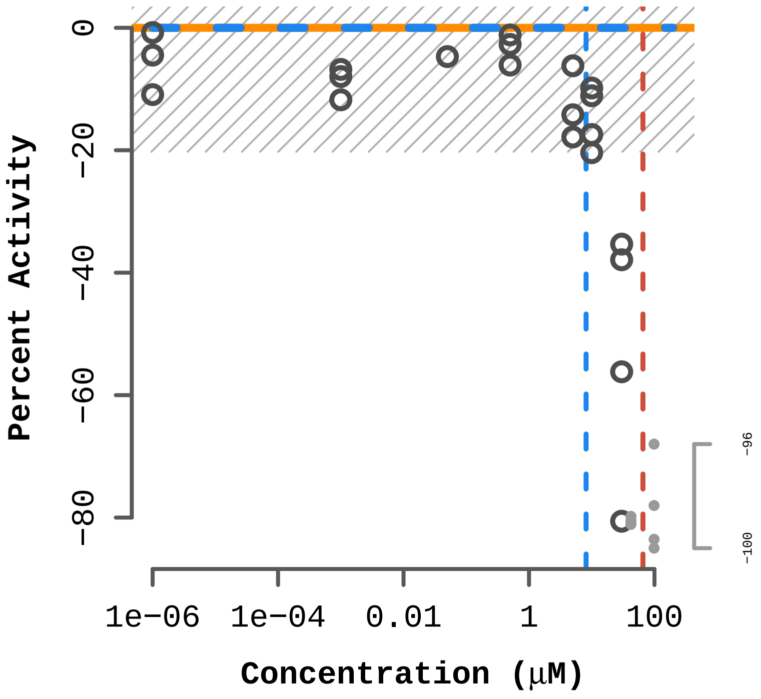

ASSAY: AEID3093 (CCTE_Deisenroth_DEVTOX_RUES2-GLR_Sox:

NAME: Esomeprazole magnesium hydrate
CHID: 746660 CASRN: 668985-31-7
SPID(S): ES211_43_43
M4ID: 42675898 BRK

HILL MODEL (in red):

	tp	ga	gw
val:	2.46e-09	1.82	2.21
sd:	NaN	NaN	NaN

GAIN-LOSS MODEL (in blue):

	tp	ga	gw	la	lw
val:	4.79e-09	0.91	2.15	1.18	6.28
sd:	0.00206	10500	995000	19300	1290000

	CNST	HILL	GNLS
AIC:	384.57	390.57	394.57
PROB:	0.95	0.05	0.01
RMSE:	51.46	51.46	51.46

MAX_MEAN: 0.101 MAX_MED: 1.23 BMAD: 6.75

COFF: 20.3 HIT-CALL: 0 FITC: 7 ACTP: 0.05

FLAGS: 10

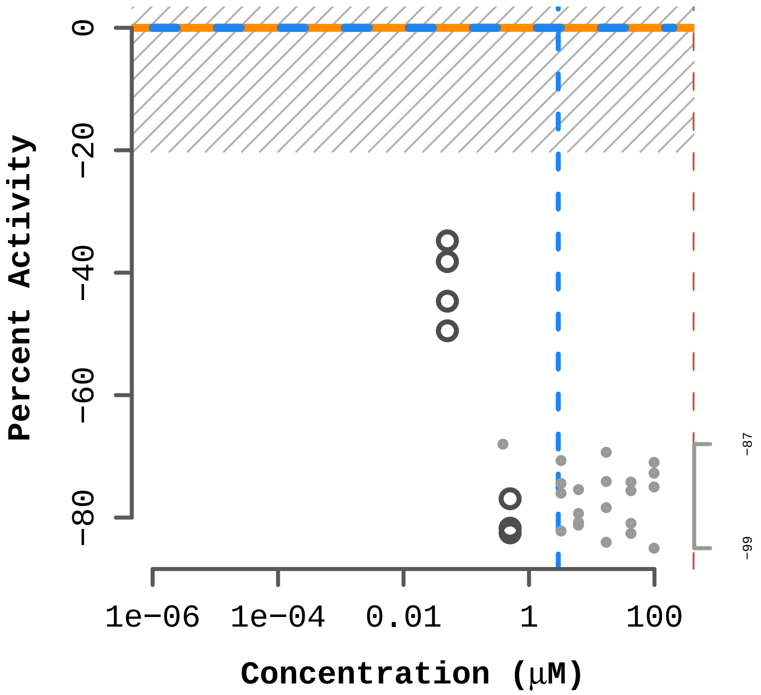

ASSAY: AEID3093 (CCTE_Deisenroth_DEVTOX_RUES2-GLR_Sox:

NAME: Pomalidomide
CHID: 893458 CASRN: 19171-19-8
SPID(S): ES211_50_50
M4ID: 42675862 BRK

HILL MODEL (in red):

	tp	ga	gw
val:	3.29e-06	2.65	6.28
sd:	NaN	NaN	NaN

GAIN-LOSS MODEL (in blue):

	tp	ga	gw	la	lw
val:	1.6e-07	0.467	6.75	0.889	11.4
sd:	NaN	NaN	NaN	NaN	NaN

	CNST	HILL	GNLS
AIC:	423.35	429.35	433.35
PROB:	0.95	0.05	0.01
RMSE:	76.56	76.56	76.56

MAX_MEAN: 8.72 MAX_MED: 7.3 BMAD: 6.75

COFF: 20.3 HIT-CALL: 0 FITC: 7 ACTP: 0.05

FLAGS: 10

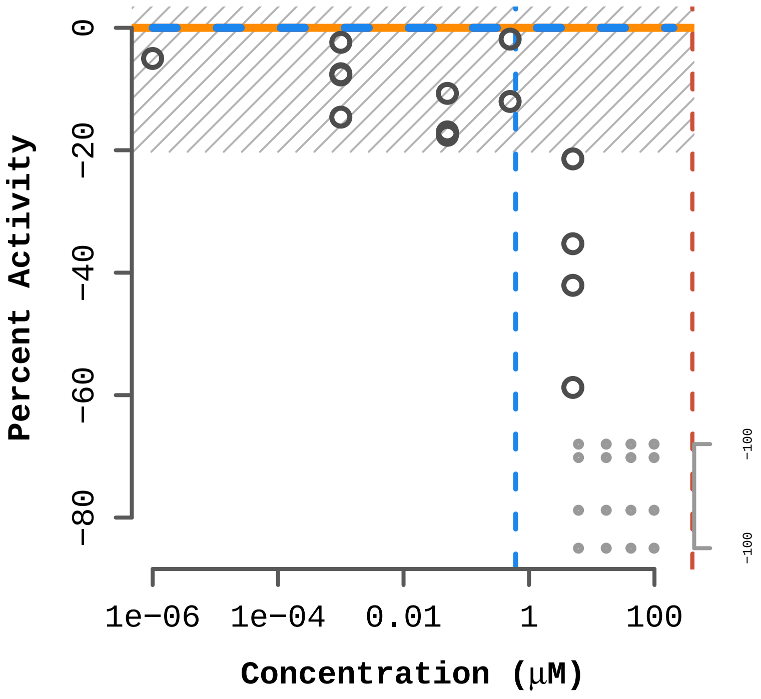

ASSAY: AEID3093 (CCTE_Deisenroth_DEVTOX_RUES2-GLR_Sox:

NAME: Tegaserod maleate
CHID: 904761 CASRN: 189188-57-6
SPID(S): ES211_61_61
M4ID: 42675893 BRK

HILL MODEL (in red):

	tp	ga	gw
val:	8.57e-07	2.61	2.43
sd:	NaN	NaN	NaN

GAIN-LOSS MODEL (in blue):

	tp	ga	gw	la	lw
val:	2.12e-05	-0.213	6.44	0.249	6.19
sd:	NaN	NaN	NaN	NaN	NaN

	CNST	HILL	GNLS
AIC:	413.53	419.53	423.53
PROB:	0.95	0.05	0.01
RMSE:	69.54	69.54	69.54

MAX_MEAN: 6.89 MAX_MED: 6.94 BMAD: 6.75

COFF: 20.3 HIT-CALL: 0 FITC: 7 ACTP: 0.05

FLAGS: 10

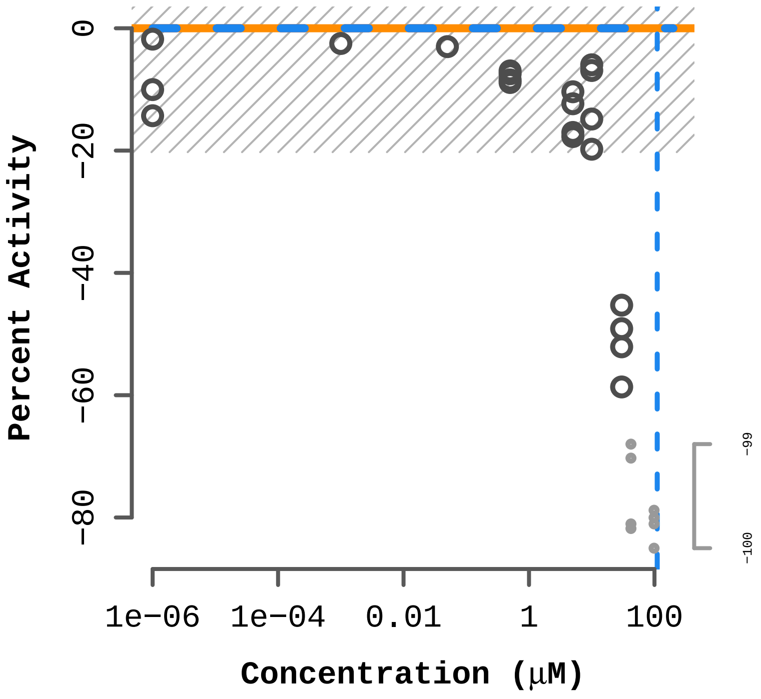

ASSAY: AEID3093 (CCTE_Deisenroth_DEVTOX_RUES2-GLR_Sox:

NAME: Mianserin hydrochloride
CHID: 944145 CASRN: 21535-47-7
SPID(S): ES211_47_47
M4ID: 42675903 BRK

HILL MODEL (in red):

	tp	ga	gw
val:	0.074	2.8	7.78
sd:	NaN	NaN	NaN

GAIN-LOSS MODEL (in blue):

	tp	ga	gw	la	lw
val:	9.22e-10	2.04	0.857	2.36	17.9
sd:	NaN	NaN	NaN	NaN	NaN

	CNST	HILL	GNLS
AIC:	384.33	390.33	394.33
PROB:	0.95	0.05	0.01
RMSE:	51.11	51.11	51.11

MAX_MEAN: 2.7 MAX_MED: 3.98 BMAD: 6.75

COFF: 20.3 HIT-CALL: 0 FITC: 7 ACTP: 0.05

FLAGS: 10

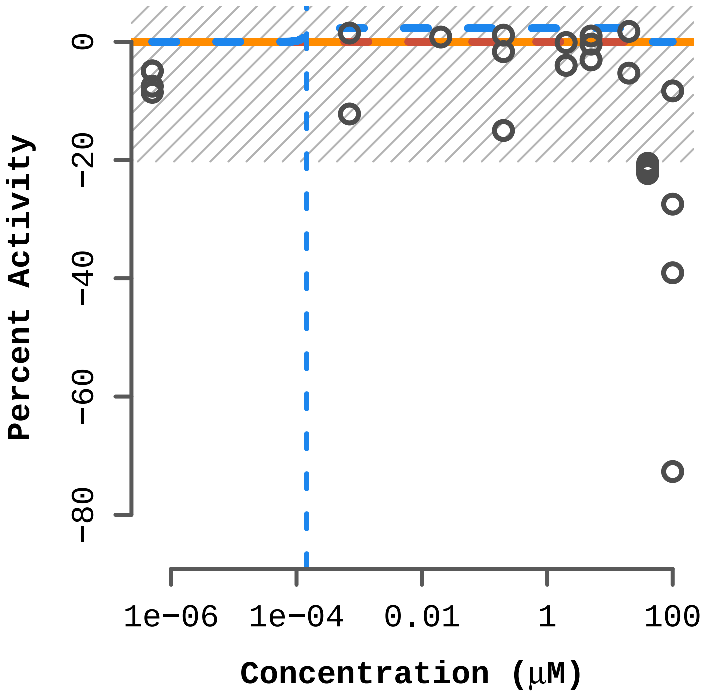

ASSAY: AEID3093 (CCTE_Deisenroth_DEVTOX_RUES2-GLR_Sox:

NAME: Ketanserin tartrate
CHID: 1003945 CASRN: 83846-83-7
SPID(S): ES211_40_40
M4ID: 42675874

HILL MODEL (in red):

	tp	ga	gw
val:	0.0744	2.49	7.96
sd:	NaN	NaN	NaN

GAIN-LOSS MODEL (in blue):

	tp	ga	gw	la	lw
val:	2.29	-3.84	7.22	1.43	18
sd:	2.03	235	2480	0.562	66.5

	CNST	HILL	GNLS
AIC:	295.8	301.8	304.58
PROB:	0.94	0.05	0.01
RMSE:	17.34	17.34	17.26

MAX_MEAN: 5.18 MAX_MED: 5.62 BMAD: 6.75

COFF: 20.3 HIT-CALL: 0 FITC: 7 ACTP: 0.06

FLAGS:

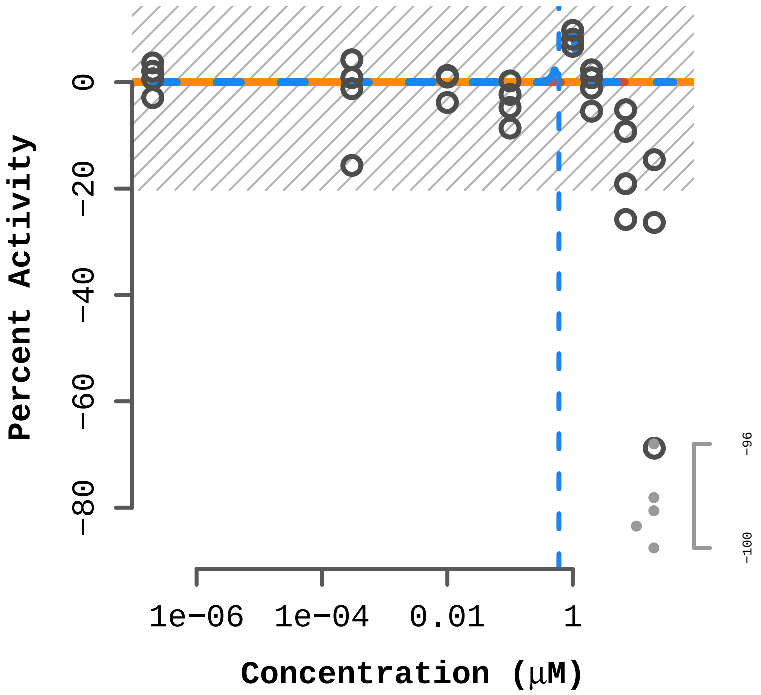

ASSAY: AEID3093 (CCTE_Deisenroth_DEVTOX_RUES2-GLR_Sox:

NAME: Dorsomorphin
CHID: 1006988 CASRN: 866405-64-3
SPID(S): ES211_6_6
M4ID: 42675888 BRK

HILL MODEL (in red):

	tp	ga	gw
val:	0.0027	2.08	7.45
sd:	NaN	NaN	NaN

GAIN-LOSS MODEL (in blue):

	tp	ga	gw	la	lw
val:	9.8	−0.22	8	0.0662	17.9
sd:	NaN	NaN	NaN	NaN	NaN

	CNST	HILL	GNLS
AIC:	351.83	357.83	360.33
PROB:	0.94	0.05	0.01
RMSE:	39.9	39.9	39.79

MAX_MEAN: 9.04 MAX_MED: 8.93 BMAD: 6.75

COFF: 20.3 HIT-CALL: 0 FITC: 7 ACTP: 0.06

FLAGS: 10

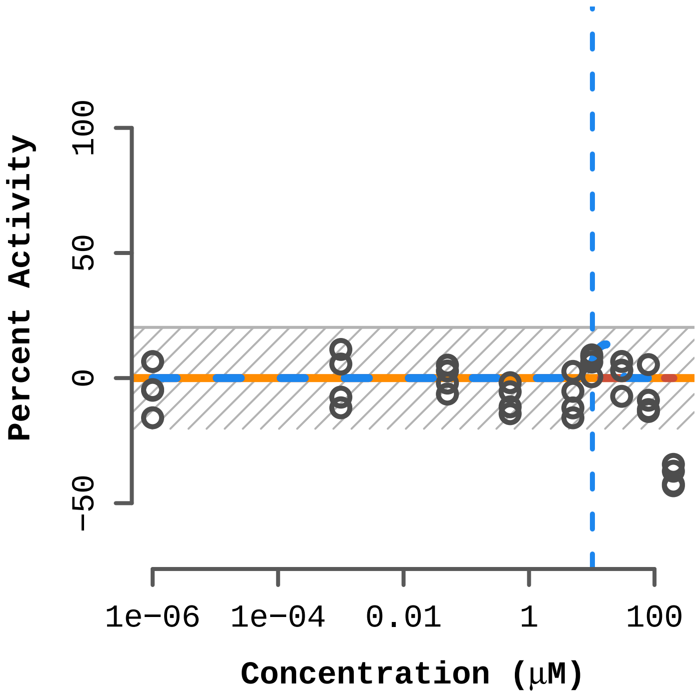

ASSAY: AEID3093 (CCTE_Deisenroth_DEVTOX_RUES2-GLR_Sox:

NAME: (2R,3S)-2-(3,4-Dihydroxyphenyl)-3,4-dihydro-2H-
CHID: 1017388 CASRN: 225937-10-0
SPID(S): ES211_22_22
M4ID: 42675887

HILL MODEL (in red):

	tp	ga	gw
val:	0.117	2.8	7.97
sd:	NaN	NaN	NaN

GAIN-LOSS MODEL (in blue):

	tp	ga	gw	la	lw
val:	13.7	1.01	8	1.41	13.6
sd:	NaN	NaN	NaN	NaN	NaN

	CNST	HILL	GNLS
AIC:	295	301	303.25
PROB:	0.94	0.05	0.02
RMSE:	15.42	15.42	15.28

MAX_MEAN: 6.18 MAX_MED: 7.45 BMAD: 6.75

COFF: 20.3 HIT-CALL: 0 FITC: 7 ACTP: 0.06

FLAGS:

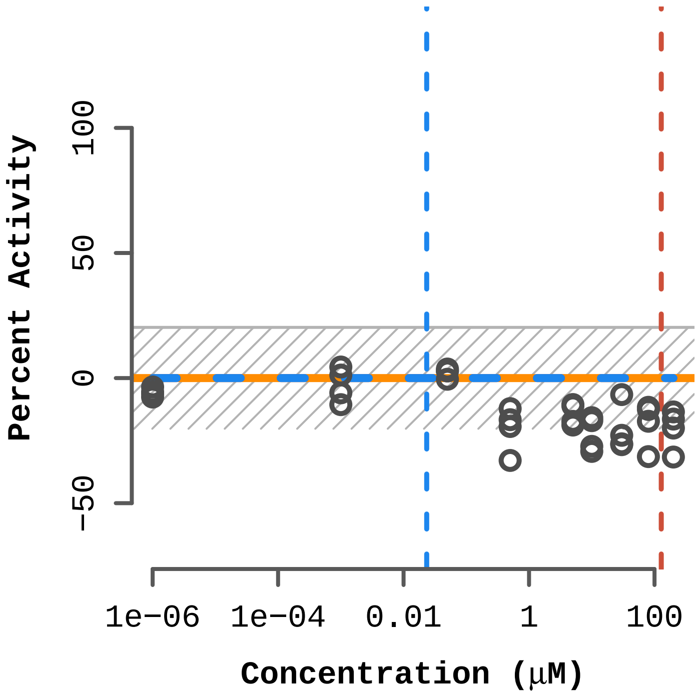

ASSAY: AEID3093 (CCTE_Deisenroth_DEVTOX_RUES2-GLR_Sox:

NAME: Methotrexate hydrate(1:x)
CHID: 1017640 CASRN: 133073-73-1
SPID(S): ES211_45_45
M4ID: 42675859

HILL MODEL (in red):

	tp	ga	gw
val:	8.02e-10	2.11	1.36
sd:	NaN	NaN	NaN

GAIN-LOSS MODEL (in blue):

	tp	ga	gw	la	lw
val:	1.55e-08	-1.63	0.509	-1.32	0.443
sd:	NaN	NaN	NaN	NaN	NaN

	CNST	HILL	GNLS
AIC:	313.19	319.19	323.19
PROB:	0.95	0.05	0.01
RMSE:	17.19	17.19	17.19

MAX_MEAN: 2.23 MAX_MED: 2.87 BMAD: 6.75

COFF: 20.3 HIT-CALL: 0 FITC: 7 ACTP: 0.05

FLAGS:

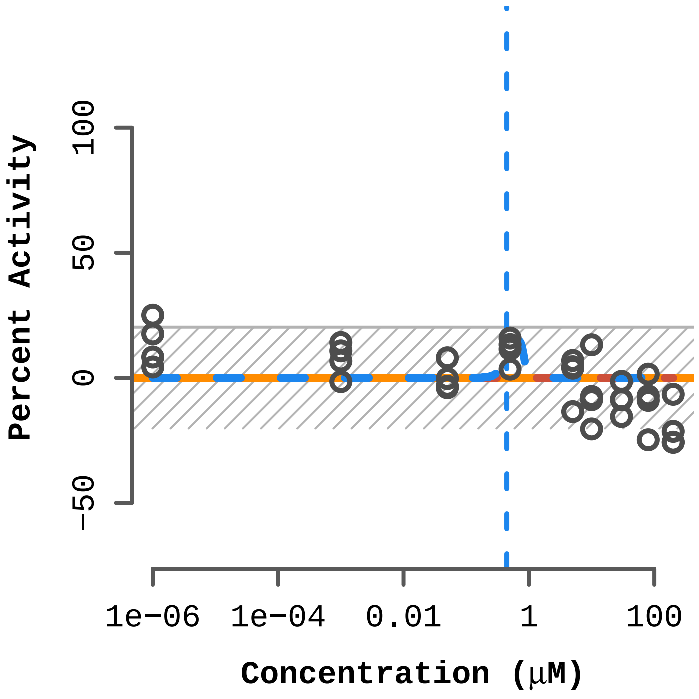

ASSAY: AEID3093 (CCTE_Deisenroth_DEVTOX_RUES2-GLR_Sox:

NAME: Methyldopa sesquihydrate
CHID: 20863 CASRN: 41372-08-1
SPID(S): ES211_46_46
M4ID: 42675866

HILL MODEL (in red):

	tp	ga	gw
val:	1.17e-08	2.79	1.73
sd:	NaN	NaN	NaN

GAIN-LOSS MODEL (in blue):

	tp	ga	gw	la	lw
val:	17	-0.353	5.52	-0.0782	15.5
sd:	854	8.83	544	192	13400

	CNST	HILL	GNLS
AIC:	291.48	297.48	297.09
PROB:	0.9	0.04	0.05
RMSE:	12.91	12.91	12.38

MAX_MEAN: 13.8 MAX_MED: 12.9 BMAD: 6.75

COFF: 20.3 HIT-CALL: 0 FITC: 9 ACTP: 0.1

FLAGS:

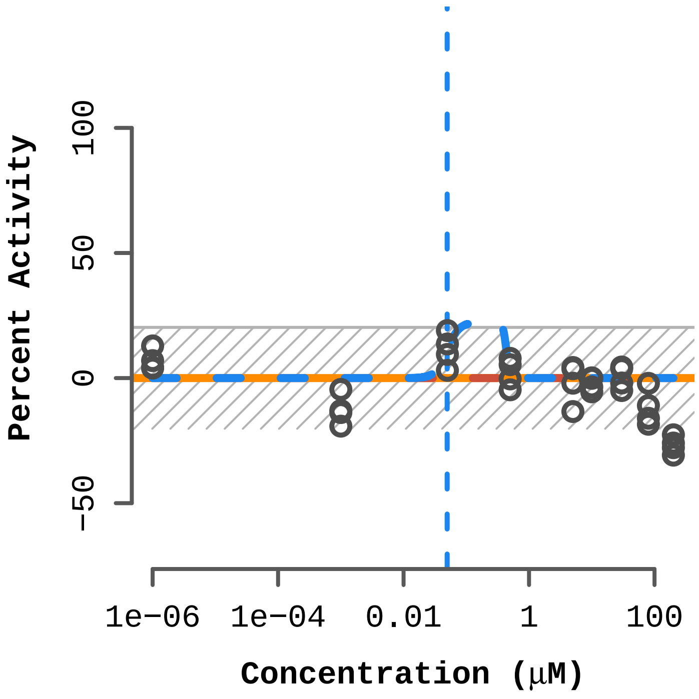

ASSAY: AEID3093 (CCTE_Deisenroth_DEVTOX_RUES2-GLR_Sox:

NAME: Warfarin
CHID: 23742 CASRN: 81-81-2
SPID(S): ES211_65_65
M4ID: 42675873

HILL MODEL (in red):

	tp	ga	gw
val:	2.57	2.8	8
sd:	NaN	NaN	NaN

GAIN-LOSS MODEL (in blue):

	tp	ga	gw	la	lw
val:	22.3	-1.31	4.64	-0.361	16.2
sd:	NaN	NaN	NaN	NaN	NaN

	CNST	HILL	GNLS
AIC:	286.97	292.97	291.72
PROB:	0.87	0.04	0.08
RMSE:	12.59	12.59	11.99

MAX_MEAN: 11.3 MAX_MED: 11.6 BMAD: 6.75

COFF: 20.3 HIT-CALL: 0 FITC: 10 ACTP: 0.13

FLAGS:

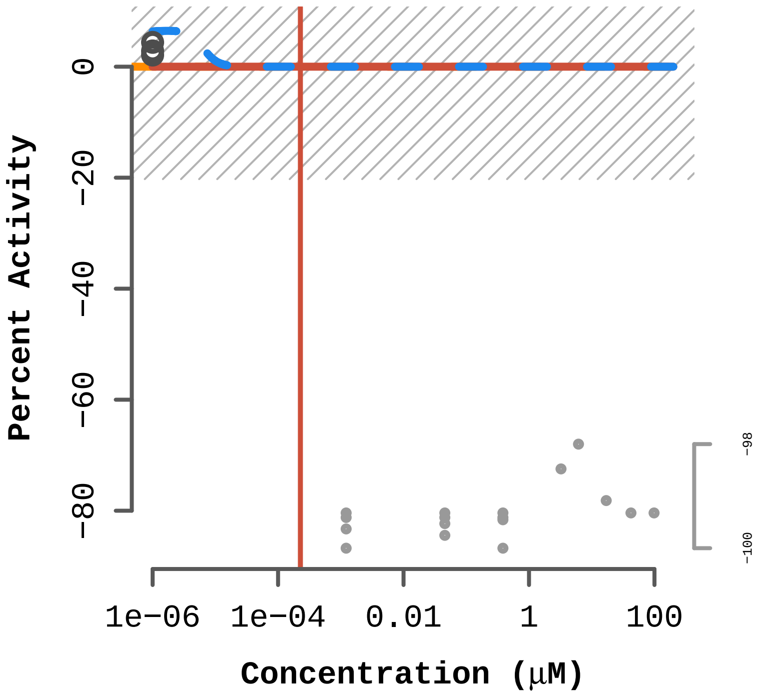

ASSAY: AEID3093 (CCTE_Deisenroth_DEVTOX_RUES2-GLR_Sox:

NAME: Actinomycin D
CHID: 20031 CASRN: 50−76−0
SPID(S): ES211_11_11
M4ID: 42675875 BRK

HILL MODEL (in red):

	tp	ga	gw
val:	2.27e−08	−3.64	1.38
sd:	NaN	NaN	NaN

GAIN−LOSS MODEL (in blue):

	tp	ga	gw	la	lw
val:	6.67	−7	1.3	−5.19	3.7
sd:	770	965	1260	1470	3600

	CNST	HILL	GNLS
AIC:	501.14	261.53	265.51
PROB:	0	0.88	0.12
RMSE:	91.05	91.05	91.01

MAX_MEAN: 6.35 MAX_MED: 3.62 BMAD: 6.75

COFF: 20.3 HIT−CALL: 0 FITC: 13 ACTP: 1

FLAGS: 10

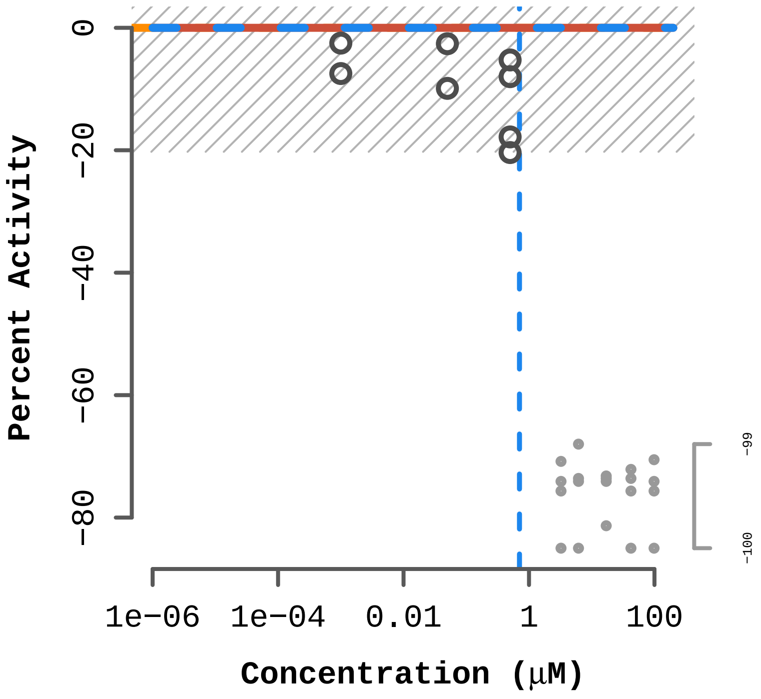

ASSAY: AEID3093 (CCTE_Deisenroth_DEVTOX_RUES2-GLR_Sox:

NAME: Diethylstilbestrol
CHID: 20465 CASRN: 56-53-1
SPID(S): ES211_29_29
M4ID: 42675854 BRK

HILL MODEL (in red):

	tp	ga	gw
val:	6.46e-07	2.79	3.26
sd:	NaN	NaN	NaN

GAIN-LOSS MODEL (in blue):

	tp	ga	gw	la	lw
val:	7.05e-07	-0.152	1.14	0.12	4.67
sd:	NaN	NaN	NaN	NaN	NaN

	CNST	HILL	GNLS
AIC:	440.17	426.95	430.95
PROB:	0	0.88	0.12
RMSE:	75.79	75.79	75.79

MAX_MEAN: 6.93 MAX_MED: 6.73 BMAD: 6.75

COFF: 20.3 HIT-CALL: 0 FITC: 13 ACTP: 1

FLAGS: 10

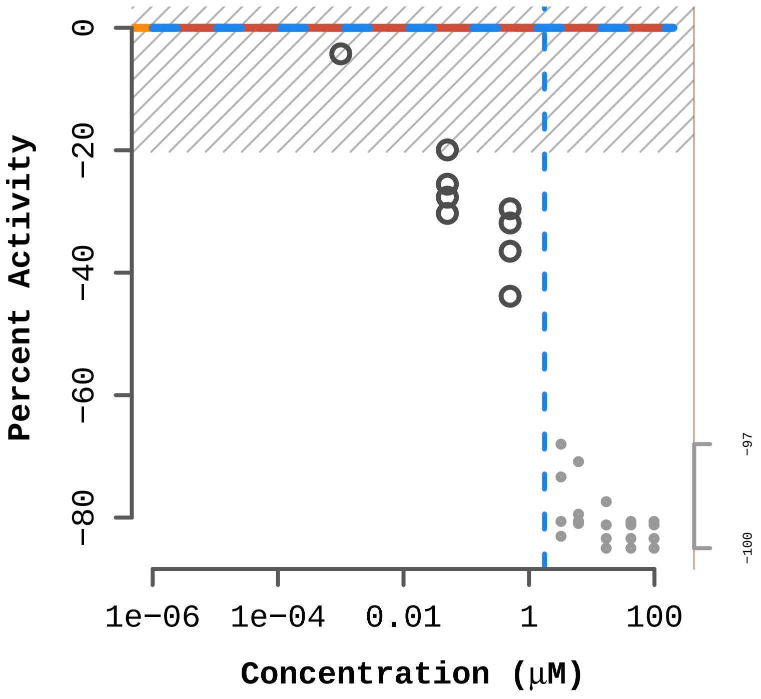

ASSAY: AEID3093 (CCTE_Deisenroth_DEVTOX_RUES2-GLR_Sox:

NAME: Dasatinib
CHID: 40979 CASRN: 302962-49-8
SPID(S): ES211_26_26
M4ID: 42675844 BRK

HILL MODEL (in red):

	tp	ga	gw
val:	1.12e-07	2.66	1.03
sd:	NaN	NaN	NaN

GAIN-LOSS MODEL (in blue):

	tp	ga	gw	la	lw
val:	2.44e-07	0.247	2.65	0.508	4.95
sd:	NaN	NaN	NaN	NaN	NaN

	CNST	HILL	GNLS
AIC:	432.03	428.43	432.43
PROB:	0.13	0.77	0.1
RMSE:	76.76	76.76	76.76

MAX_MEAN: 5.98 MAX_MED: 3.77 BMAD: 6.75

COFF: 20.3 HIT-CALL: 0 FITC: 13 ACTP: 0.87

FLAGS: 10

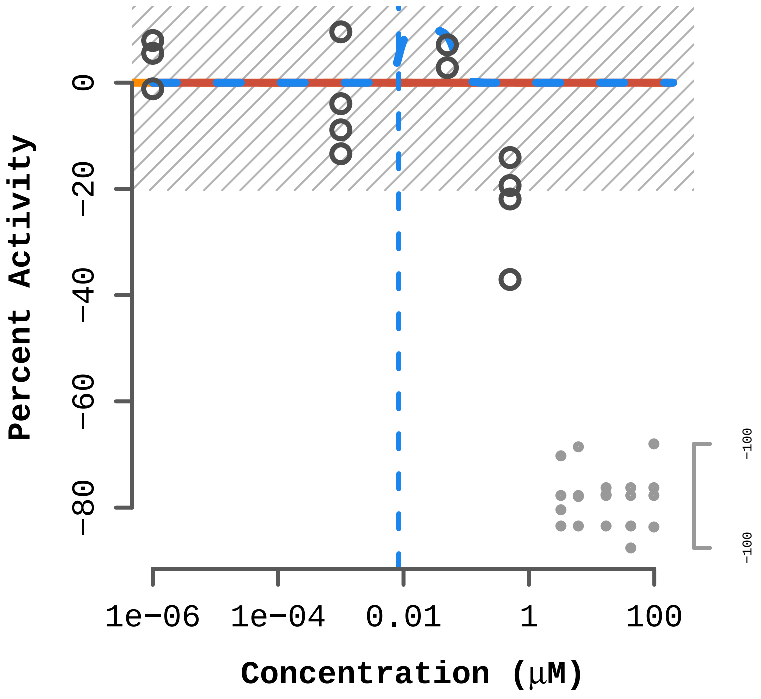

ASSAY: AEID3093 (CCTE_Deisenroth_DEVTOX_RUES2-GLR_Sox:

NAME: Sorafenib
CHID: 41128 CASRN: 284461-73-0
SPID(S): ES211_57_57
M4ID: 42675902 BRK

HILL MODEL (in red):

	tp	ga	gw
val:	0.000281	2.78	5.33
sd:	NaN	NaN	NaN

GAIN-LOSS MODEL (in blue):

	tp	ga	gw	la	lw
val:	9.88	-2.08	7.87	-1.17	6.56
sd:	531	2840	24300	27.7	632

	CNST	HILL	GNLS
AIC:	501.52	427.5	431.42
PROB:	0	0.88	0.12
RMSE:	76.16	76.16	76.11

MAX_MEAN: 8.73 MAX_MED: 9.19 BMAD: 6.75

COFF: 20.3 HIT-CALL: 0 FITC: 13 ACTP: 1

FLAGS: 10

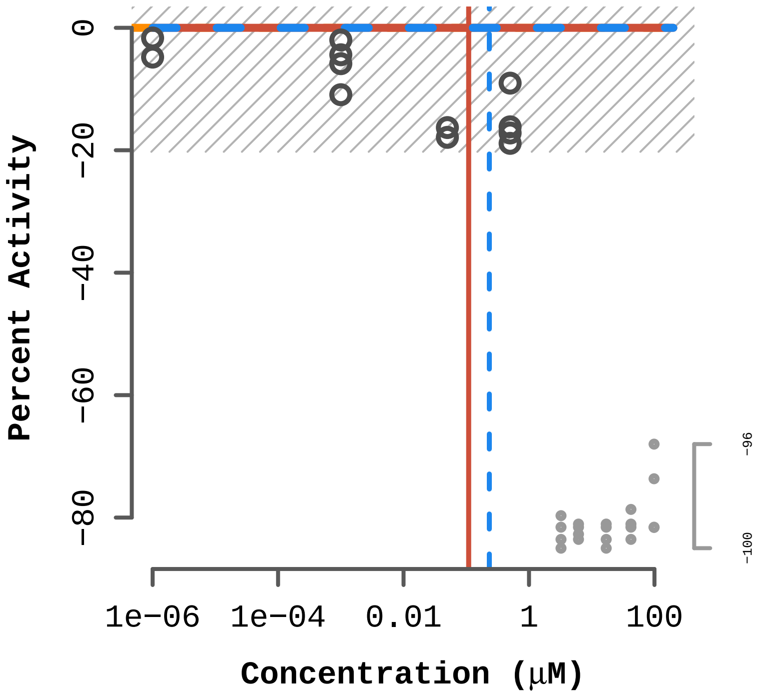

ASSAY: AEID3093 (CCTE_Deisenroth_DEVTOX_RUES2-GLR_Sox

NAME: Sunitinib malate
CHID: 46492 CASRN: 341031-54-7
SPID(S): ES211_60_60
M4ID: 42675901 BRK

HILL MODEL (in red):

	tp	ga	gw
val:	2.65e-09	-0.962	0.629
sd:	NaN	NaN	NaN

GAIN-LOSS MODEL (in blue):

	tp	ga	gw	la	lw
val:	5.43e-09	-0.632	0.434	1.5	5.09
sd:	NaN	NaN	NaN	NaN	NaN

	CNST	HILL	GNLS
AIC:	446.04	426.88	430.88
PROB:	0	0.88	0.12
RMSE:	75.66	75.66	75.66

MAX_MEAN: 0.538 MAX_MED: 0.596 BMAD: 6.75

COFF: 20.3 HIT-CALL: 0 FITC: 13 ACTP: 1

FLAGS: 10

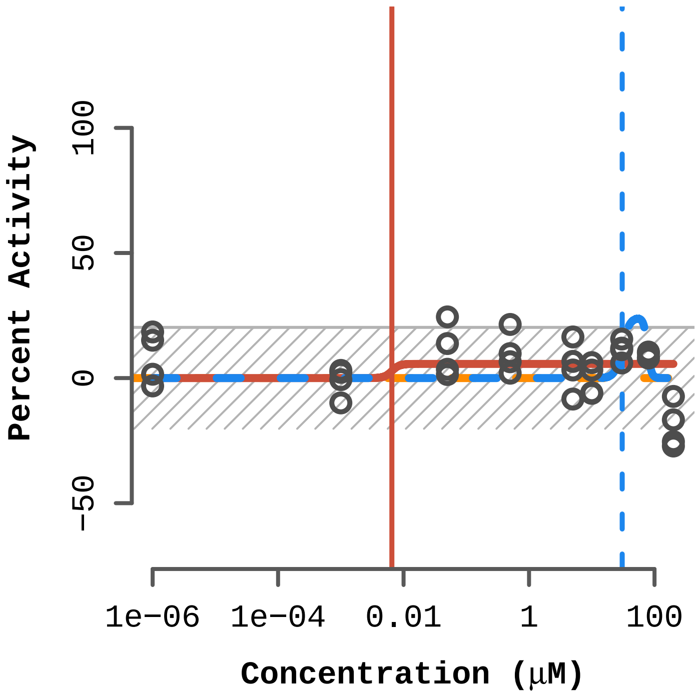

ASSAY: AEID3093 (CCTE_Deisenroth_DEVTOX_RUES2-GLR_Sox:

NAME: 5,5-Diphenylhydantoin
CHID: 20541 CASRN: 57-41-0
SPID(S): ES211_3_3
M4ID: 42675900

HILL MODEL (in red):

	tp	ga	gw
val:	5.66	-2.19	7.3
sd:	1.94	76.2	655

GAIN-LOSS MODEL (in blue):

	tp	ga	gw	la	lw
val:	24.2	1.49	7.39	1.89	14.1
sd:	NaN	NaN	NaN	NaN	NaN

	CNST	HILL	GNLS
AIC:	285.71	284.82	285.26
PROB:	0.26	0.41	0.33
RMSE:	12.05	11.73	11.03

MAX_MEAN: 11.2 MAX_MED: 11.7 BMAD: 6.75

COFF: 20.3 HIT-CALL: 0 FITC: 16 ACTP: 0.74

FLAGS: 12

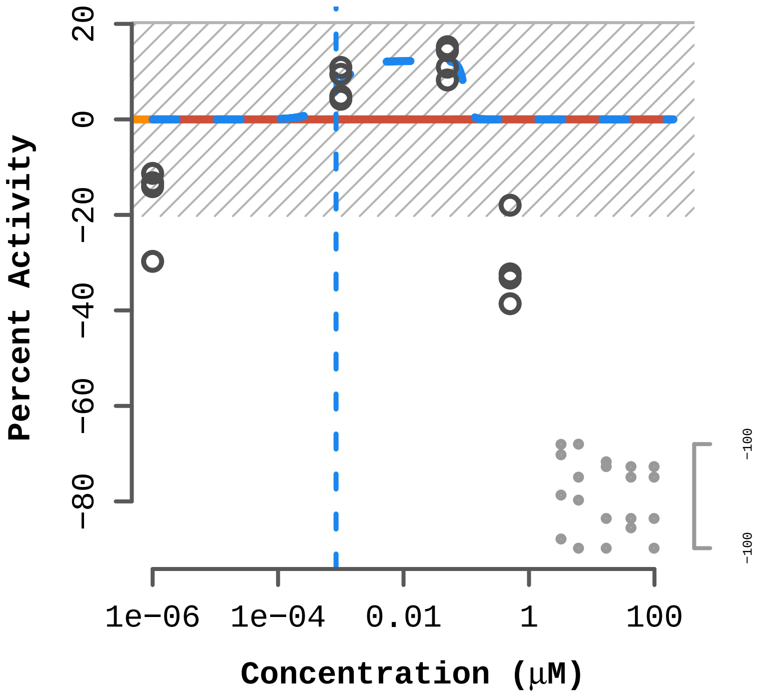

ASSAY: AEID3093 (CCTE_Deisenroth_DEVTOX_RUES2-GLR_Sox

NAME: Cytarabine hydrochloride
CHID: 24891 CASRN: 69-74-9
SPID(S): ES211_25_25
M4ID: 42675886 BRK

HILL MODEL (in red):

	tp	ga	gw
val:	18.3	2.8	7.41
sd:	NaN	NaN	NaN

GAIN-LOSS MODEL (in blue):

	tp	ga	gw	la	lw
val:	12.2	-3.08	2.35	-1.02	9.02
sd:	334	25.6	725	308	3910

	CNST	HILL	GNLS
AIC:	512.8	428.35	432.14
PROB:	0	0.87	0.13
RMSE:	76.84	76.84	76.69

MAX_MEAN: 12.2 MAX_MED: 12.7 BMAD: 6.75

COFF: 20.3 HIT-CALL: 0 FITC: 18 ACTP: 1

FLAGS: 10; 12

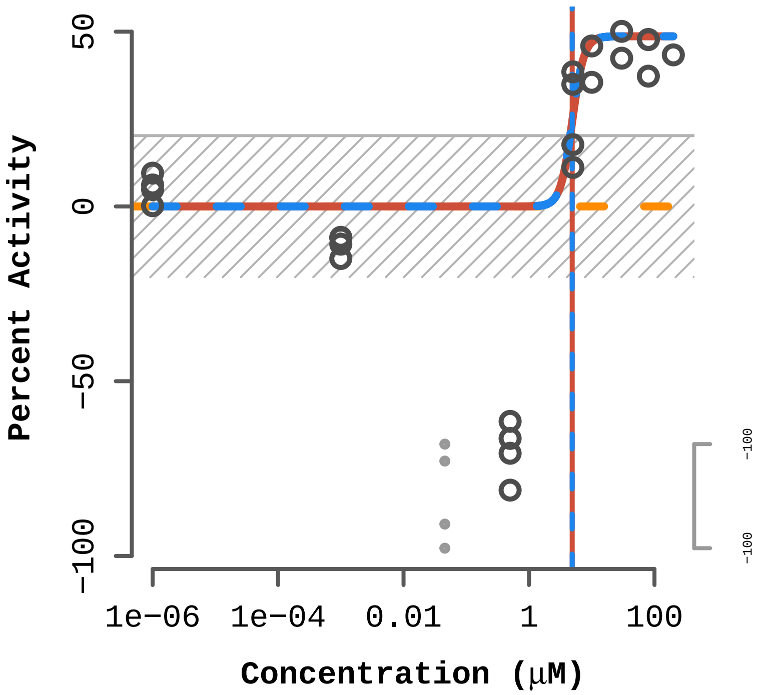

ASSAY: AEID3093 (CCTE_Deisenroth_DEVTOX_RUES2-GLR_Sox:

NAME: Adriamycin hydrochloride
CHID: 30636 CASRN: 25316-40-9
SPID(S): ES211_67_67
M4ID: 42675872 BRK

HILL MODEL (in red):

	tp	ga	gw
val:	48.7	0.689	4.61
sd:	5.54	0.0868	9.21

GAIN-LOSS MODEL (in blue):

	tp	ga	gw	la	lw
val:	48.7	0.689	4.61	4.2	8.17
sd:	NA	NA	NA	NA	NA

	CNST	HILL	GNLS
AIC:	394.89	367.26	371.26
PROB:	0	0.88	0.12
RMSE:	53.29	41.67	41.67

MAX_MEAN: 50.4 MAX_MED: 51.7 BMAD: 6.75

COFF: 20.3 HIT-CALL: 1 FITC: 41 ACTP: 1

FLAGS: 10; 17
