# Supplementary material for: The DevTox Germ Layer Reporter Platform: An Assay Adaptation of the Human Pluripotent Stem Cell Test
Source: Toxics. 2022 Jul 13;10(7):392. doi: 10.3390/toxics10070392 (PMC9321663; doi:10.3390/toxics10070392)
Supplement: Supplementary file 1 [file toxics-10-00392-s001.zip › AEID3094_L6_CCTE_Deisenroth_DEVTOX_RUES2-GLR_Sox17_dn_210917.pdf]

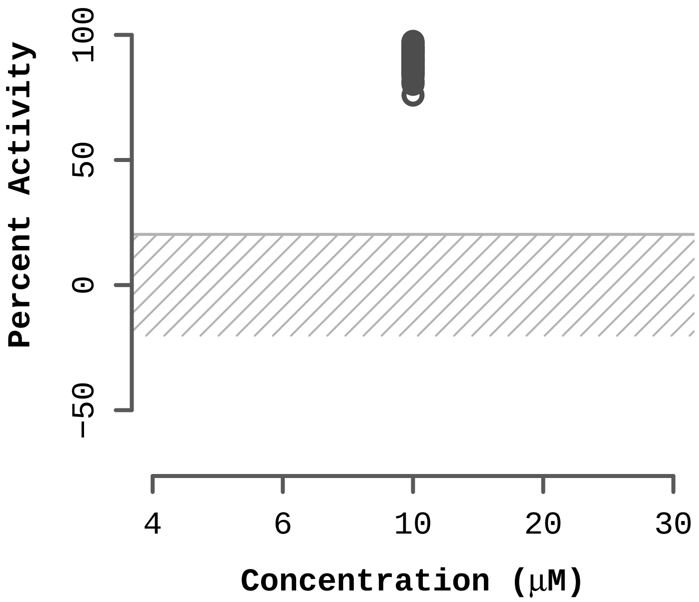

ASSAY: AEID3094 (CCTE_Deisenroth_DEVTOX_RUES2-GLR_Sox[illegible]

NAME: SB-431542
CHID: 42693 CASRN: 301836-41-9
SPID(S): ES211_56_56
M4ID: 42675915

HILL MODEL: Not applicable.

GAIN-LOSS MODEL: Not applicable.

	CNST	HILL	GNLS
AIC:	NA	NA	NA
PROB:	NA	NA	NA
RMSE:	NA	NA	NA

MAX_MEAN: 90 MAX_MED: 90.4 BMAD: 6.75

COFF: 20.3 HIT-CALL: -1 FITC: 2 ACTP: NA

FLAGS:

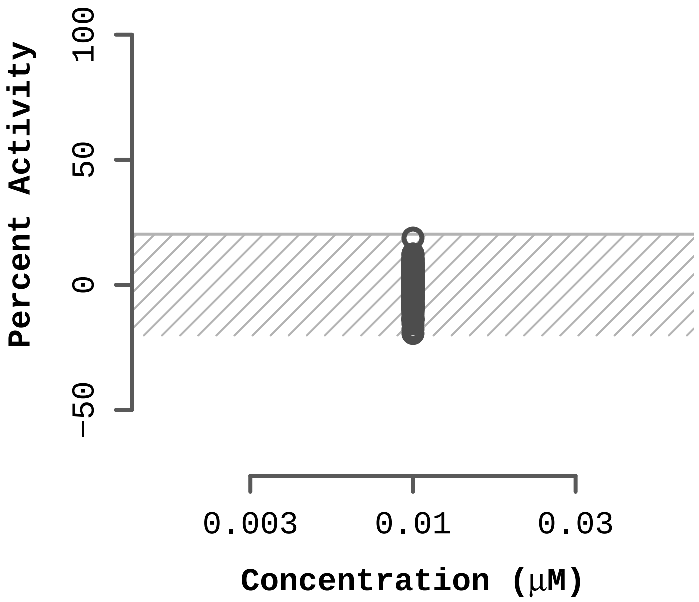

ASSAY: AEID3094 (CCTE_Deisenroth_DEVTOX_RUES2-GLR_Sox:

NAME: DMSO (spid not in DB)
CHID: NA CASRN: NA
SPID(S): DMSO
M4ID: 42675908

HILL MODEL: Not applicable.

GAIN-LOSS MODEL: Not applicable.

	CNST	HILL	GNLS
AIC:	NA	NA	NA
PROB:	NA	NA	NA
RMSE:	NA	NA	NA

MAX_MEAN: −0.5 MAX_MED: 5.73e−15 BMAD: 6.75

COFF: 20.3 HIT−CALL: −1 FITC: 2 ACTP: NA

FLAGS:

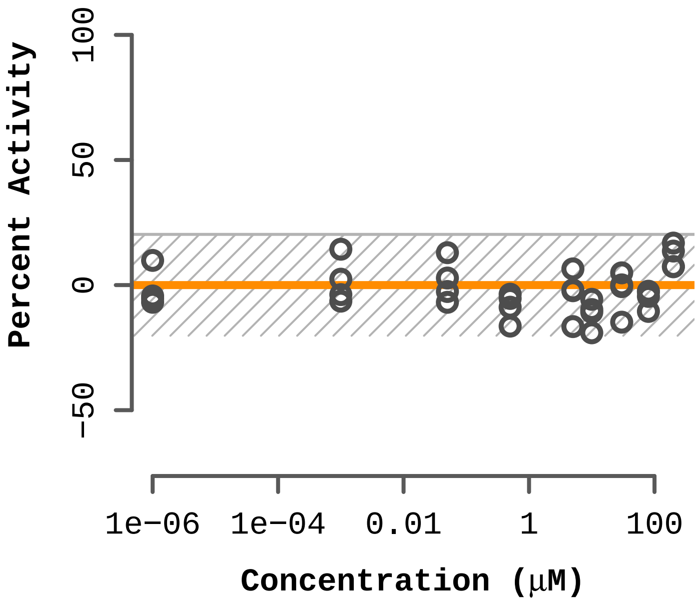

ASSAY: AEID3094 (CCTE_Deisenroth_DEVTOX_RUES2-GLR_Sox:

NAME: Acetaminophen
CHID: 20006 CASRN: 103−90−2
SPID(S): ES211_9_9
M4ID: 42675925

HILL MODEL: Not applicable.

GAIN−LOSS MODEL: Not applicable.

	CNST	HILL	GNLS
AIC:	266.51	NA	NA
PROB:	1	NA	NA
RMSE:	9.09	NA	NA

MAX_MEAN: 11.3 MAX_MED: 10.5 BMAD: 6.75

COFF: 20.3 HIT−CALL: 0 FITC: 4 ACTP: 0

FLAGS:

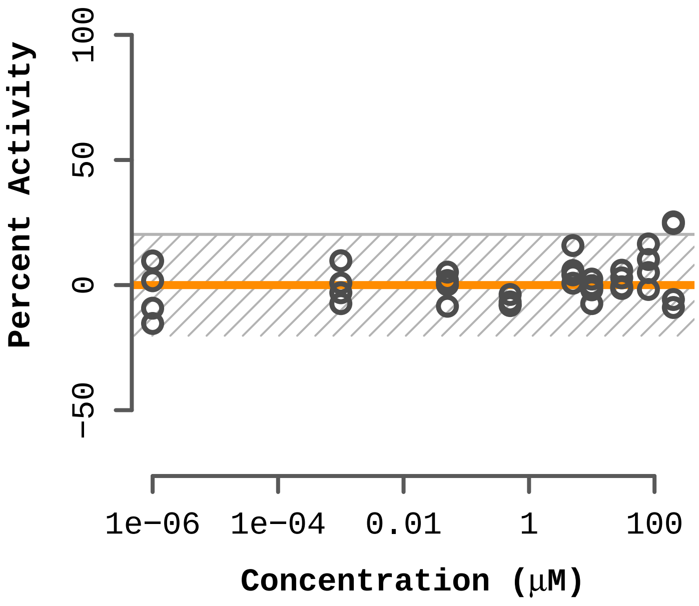

ASSAY: AEID3094 (CCTE_Deisenroth_DEVTOX_RUES2-GLR_Sox

NAME: Acrylamide
CHID: 20027 CASRN: 79-06-1
SPID(S): ES211_10_10
M4ID: 42675945

HILL MODEL: Not applicable.

GAIN-LOSS MODEL: Not applicable.

	CNST	HILL	GNLS
AIC:	262.31	NA	NA
PROB:	1	NA	NA
RMSE:	9.19	NA	NA

MAX_MEAN: 8.79 MAX_MED: 9.42 BMAD: 6.75

COFF: 20.3 HIT-CALL: 0 FITC: 4 ACTP: 0

FLAGS:

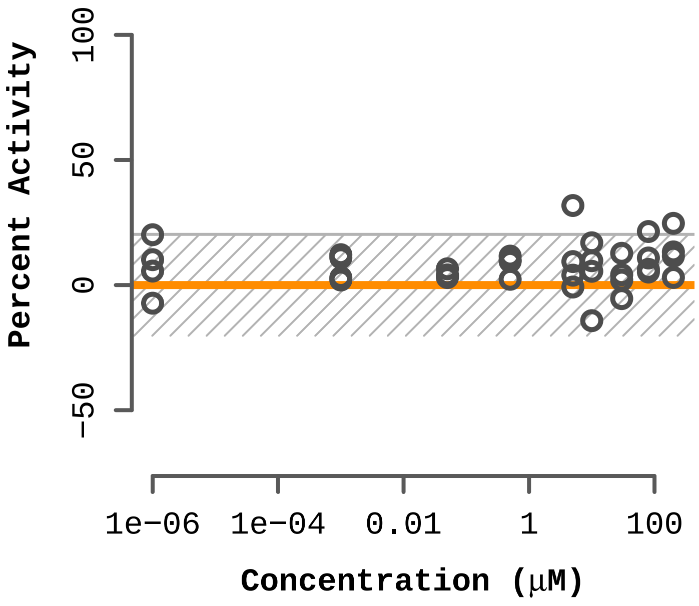

ASSAY: AEID3094 (CCTE_Deisenroth_DEVTOX_RUES2-GLR_Sox:

NAME: Aspirin
CHID: 20108 CASRN: 50−78−2
SPID(S): ES211_15_15
M4ID: 42675937

HILL MODEL: Not applicable.

GAIN−LOSS MODEL: Not applicable.

	CNST	HILL	GNLS
AIC:	281.49	NA	NA
PROB:	1	NA	NA
RMSE:	11.54	NA	NA

MAX_MEAN: 13.1 MAX_MED: 12.4 BMAD: 6.75

COFF: 20.3 HIT−CALL: 0 FITC: 4 ACTP: 0

FLAGS:

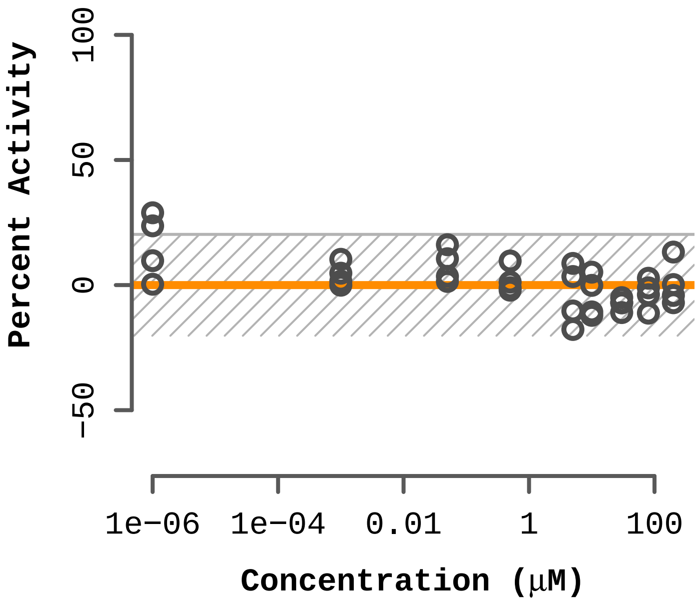

ASSAY: AEID3094 (CCTE_Deisenroth_DEVTOX_RUES2-GLR_Sox:

NAME: Boric acid (H3BO3)
CHID: 20194 CASRN: 10043-35-3
SPID(S): ES211_17_17
M4ID: 42675931

HILL MODEL: Not applicable.

GAIN-LOSS MODEL: Not applicable.

	CNST	HILL	GNLS
AIC:	268.7	NA	NA
PROB:	1	NA	NA
RMSE:	9.92	NA	NA

MAX_MEAN: 15.7 MAX_MED: 16.7 BMAD: 6.75

COFF: 20.3 HIT-CALL: 0 FITC: 4 ACTP: 0

FLAGS:

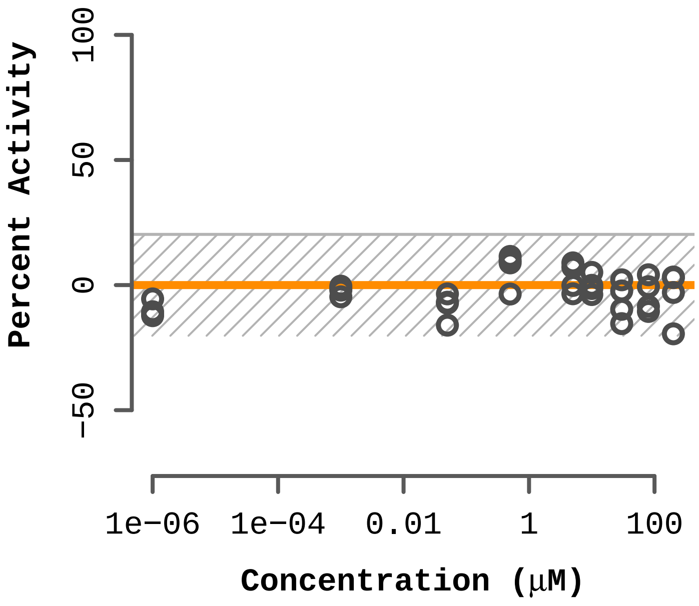

ASSAY: AEID3094 (CCTE_Deisenroth_DEVTOX_RUES2-GLR_Sox:

NAME: Caffeine
CHID: 20232 CASRN: 58−08−2
SPID(S): ES211_20_20
M4ID: 42675953

HILL MODEL: Not applicable.

GAIN−LOSS MODEL: Not applicable.

	CNST	HILL	GNLS
AIC:	255.63	NA	NA
PROB:	1	NA	NA
RMSE:	7.94	NA	NA

MAX_MEAN: 6.68 MAX_MED: 9.41 BMAD: 6.75

COFF: 20.3 HIT−CALL: 0 FITC: 4 ACTP: 0

FLAGS:

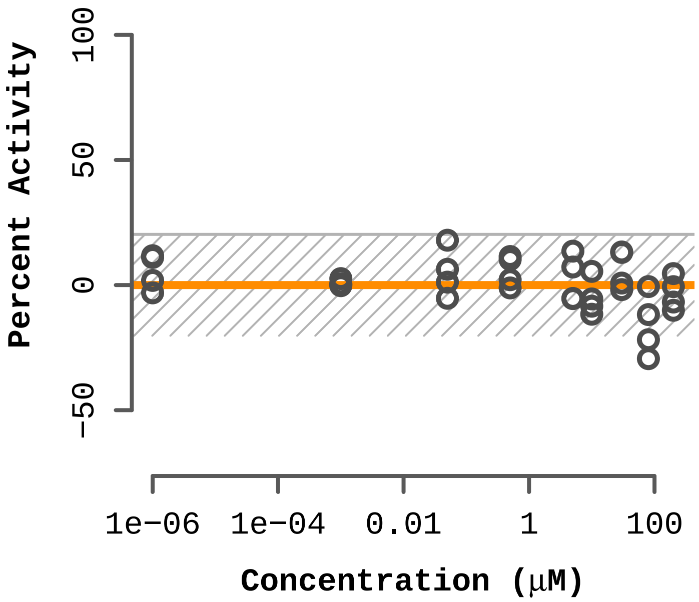

ASSAY: AEID3094 (CCTE_Deisenroth_DEVTOX_RUES2-GLR_Sox:

NAME: Ethylene glycol
CHID: 20597 CASRN: 107-21-1
SPID(S): ES211_32_32
M4ID: 42675946

HILL MODEL: Not applicable.

GAIN-LOSS MODEL: Not applicable.

	CNST	HILL	GNLS
AIC:	268.12	NA	NA
PROB:	1	NA	NA
RMSE:	9.82	NA	NA

MAX_MEAN: 6.33 MAX_MED: 6.93 BMAD: 6.75

COFF: 20.3 HIT-CALL: 0 FITC: 4 ACTP: 0

FLAGS:

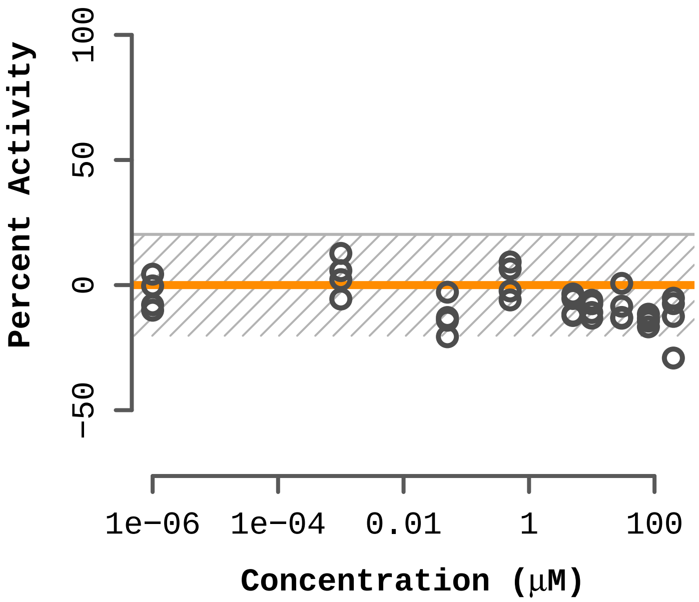

ASSAY: AEID3094 (CCTE_Deisenroth_DEVTOX_RUES2-GLR_Sox[illegible]

NAME: Isoniazid
CHID: 20755 CASRN: 54-85-3
SPID(S): ES211_39_39
M4ID: 42675939

HILL MODEL: Not applicable.

GAIN-LOSS MODEL: Not applicable.

	CNST	HILL	GNLS
AIC:	280.85	NA	NA
PROB:	1	NA	NA
RMSE:	11.04	NA	NA

MAX_MEAN: 3.77 MAX_MED: 3.99 BMAD: 6.75

COFF: 20.3 HIT-CALL: 0 FITC: 4 ACTP: 0

FLAGS:

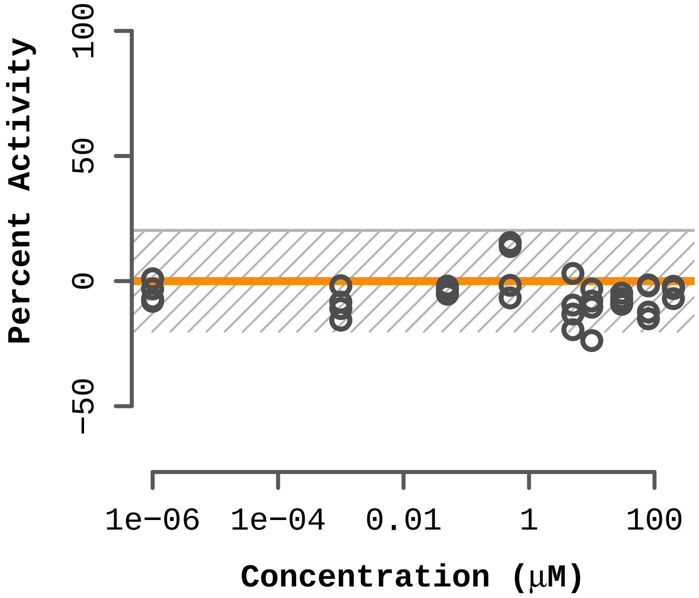

ASSAY: AEID3094 (CCTE_Deisenroth_DEVTOX_RUES2-GLR_Sox:

NAME: Nicotinic acid
CHID: 20932 CASRN: 59-67-6
SPID(S): ES211_48_48
M4ID: 42675924

HILL MODEL: Not applicable.

GAIN-LOSS MODEL: Not applicable.

	CNST	HILL	GNLS
AIC:	267.87	NA	NA
PROB:	1	NA	NA
RMSE:	9.41	NA	NA

MAX_MEAN: 5.21 MAX_MED: 6.06 BMAD: 6.75

COFF: 20.3 HIT-CALL: 0 FITC: 4 ACTP: 0

FLAGS:

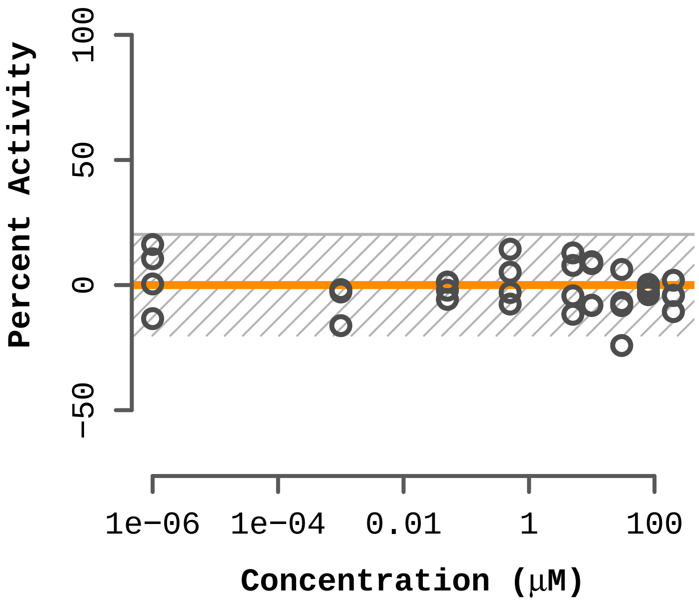

ASSAY: AEID3094 (CCTE_Deisenroth_DEVTOX_RUES2-GLR_Sox:

NAME: 1,2-Propylene glycol
CHID: 21206 CASRN: 57-55-6
SPID(S): ES211_1_1
M4ID: 42675907

HILL MODEL: Not applicable.

GAIN-LOSS MODEL: Not applicable.

	CNST	HILL	GNLS
AIC:	261.77	NA	NA
PROB:	1	NA	NA
RMSE:	8.75	NA	NA

MAX_MEAN: 3.45 MAX_MED: 5.48 BMAD: 6.75

COFF: 20.3 HIT-CALL: 0 FITC: 4 ACTP: 0

FLAGS:

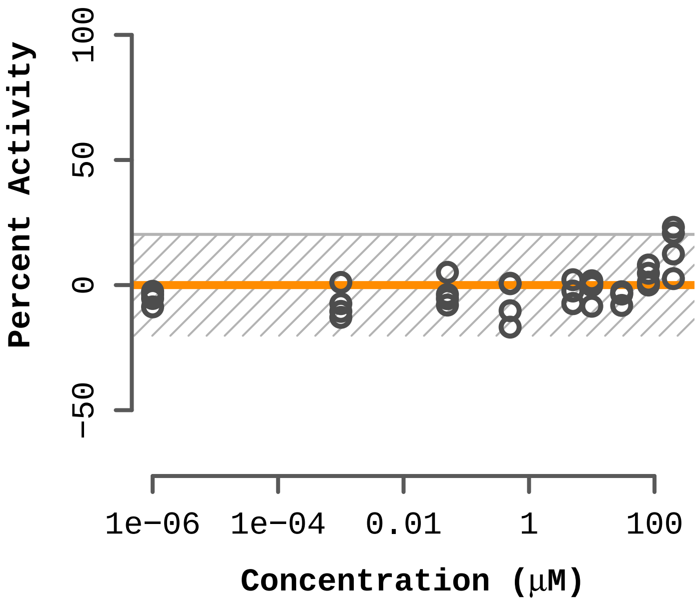

ASSAY: AEID3094 (CCTE_Deisenroth_DEVTOX_RUES2-GLR_Sox

NAME: 6-Propyl-2-thiouracil
CHID: 21209 CASRN: 51-52-5
SPID(S): ES211_8_8
M4ID: 42675926

HILL MODEL: Not applicable.

GAIN-LOSS MODEL: Not applicable.

	CNST	HILL	GNLS
AIC:	255.62	NA	NA
PROB:	1	NA	NA
RMSE:	8.32	NA	NA

MAX_MEAN: 14.7 MAX_MED: 16.6 BMAD: 6.75

COFF: 20.3 HIT-CALL: 0 FITC: 4 ACTP: 0

FLAGS:

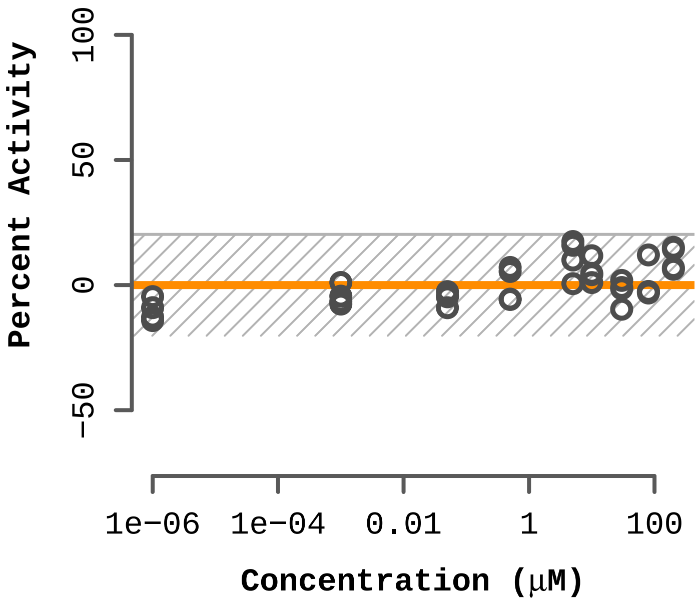

ASSAY: AEID3094 (CCTE_Deisenroth_DEVTOX_RUES2-GLR_Sox:

NAME: Saccharin
CHID: 21251 CASRN: 81-07-2
SPID(S): ES211_54_54
M4ID: 42675919

HILL MODEL: Not applicable.

GAIN-LOSS MODEL: Not applicable.

	CNST	HILL	GNLS
AIC:	260.05	NA	NA
PROB:	1	NA	NA
RMSE:	8.31	NA	NA

MAX_MEAN: 11 MAX_MED: 13 BMAD: 6.75

COFF: 20.3 HIT-CALL: 0 FITC: 4 ACTP: 0

FLAGS:

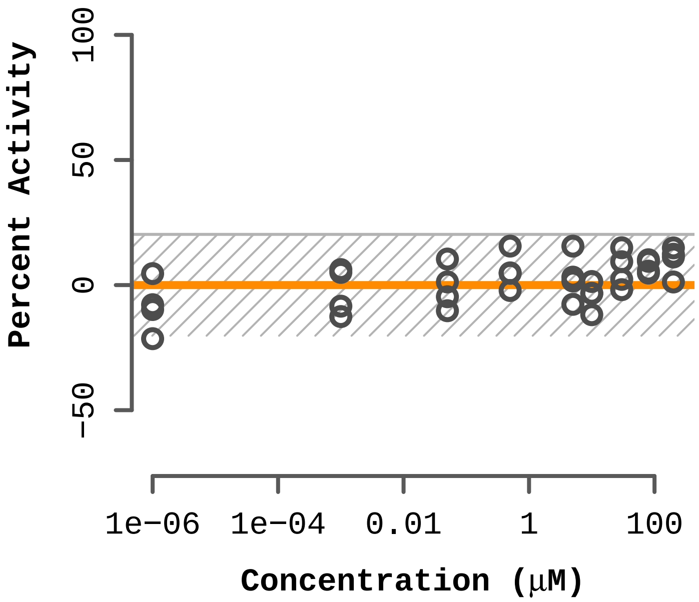

ASSAY: AEID3094 (CCTE_Deisenroth_DEVTOX_RUES2-GLR_Sox:

NAME: Sulfasalazine
CHID: 21256 CASRN: 599-79-1
SPID(S): ES211_59_59
M4ID: 42675928

HILL MODEL: Not applicable.

GAIN-LOSS MODEL: Not applicable.

	CNST	HILL	GNLS
AIC:	267.25	NA	NA
PROB:	1	NA	NA
RMSE:	9.14	NA	NA

MAX_MEAN: 9.93 MAX_MED: 11.8 BMAD: 6.75

COFF: 20.3 HIT-CALL: 0 FITC: 4 ACTP: 0

FLAGS:

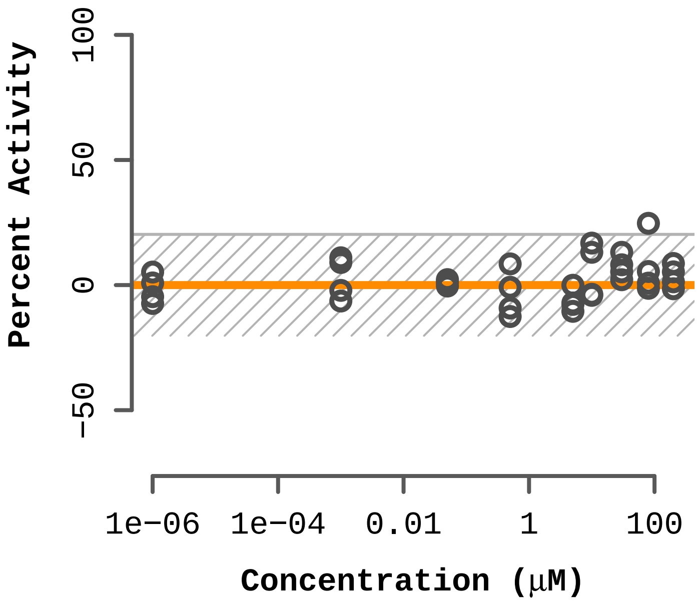

ASSAY: AEID3094 (CCTE_Deisenroth_DEVTOX_RUES2-GLR_Sox:

NAME: Dimethyl phthalate
CHID: 22455 CASRN: 131-11-3
SPID(S): ES211_30_30
M4ID: 42675957

HILL MODEL: Not applicable.

GAIN-LOSS MODEL: Not applicable.

	CNST	HILL	GNLS
AIC:	255.23	NA	NA
PROB:	1	NA	NA
RMSE:	8.16	NA	NA

MAX_MEAN: 7.41 MAX_MED: 6.7 BMAD: 6.75

COFF: 20.3 HIT-CALL: 0 FITC: 4 ACTP: 0

FLAGS:

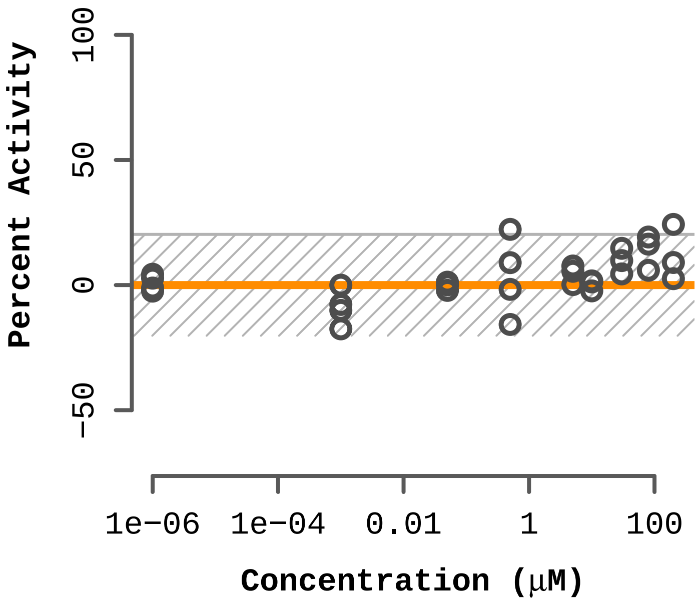

ASSAY: AEID3094 (CCTE_Deisenroth_DEVTOX_RUES2-GLR_Sox

NAME: Folic acid
CHID: 22519 CASRN: 59−30−3
SPID(S): ES211_33_33
M4ID: 42675914

HILL MODEL: Not applicable.

GAIN−LOSS MODEL: Not applicable.

	CNST	HILL	GNLS
AIC:	232.24	NA	NA
PROB:	1	NA	NA
RMSE:	10.02	NA	NA

MAX_MEAN: 13.8 MAX_MED: 16.4 BMAD: 6.75

COFF: 20.3 HIT−CALL: 0 FITC: 4 ACTP: 0

FLAGS:

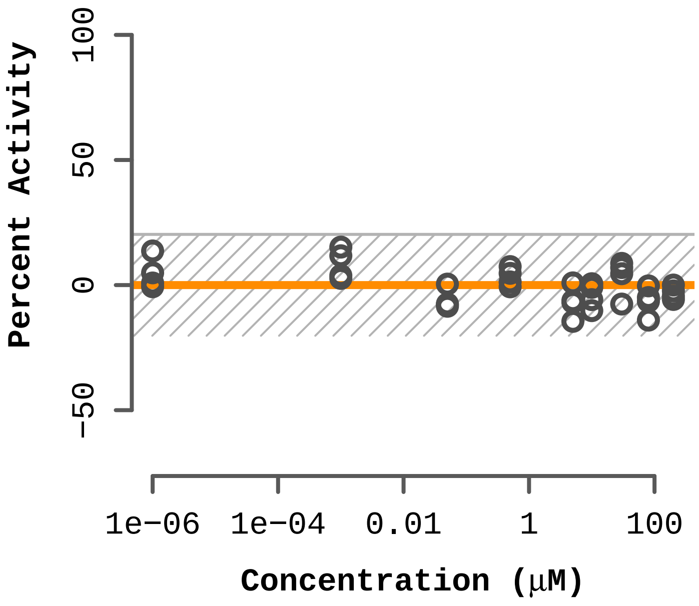

ASSAY: AEID3094 (CCTE_Deisenroth_DEVTOX_RUES2-GLR_Sox:

NAME: Acyclovir
CHID: 22556 CASRN: 59277-89-3
SPID(S): ES211_12_12
M4ID: 42675954

HILL MODEL: Not applicable.

GAIN-LOSS MODEL: Not applicable.

	CNST	HILL	GNLS
AIC:	248.4	NA	NA
PROB:	1	NA	NA
RMSE:	7.13	NA	NA

MAX_MEAN: 8.36 MAX_MED: 7.79 BMAD: 6.75

COFF: 20.3 HIT-CALL: 0 FITC: 4 ACTP: 0

FLAGS:

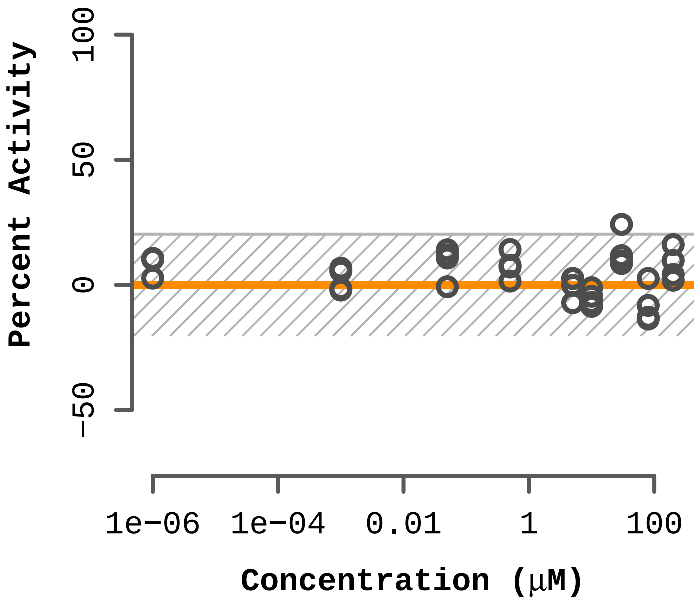

ASSAY: AEID3094 (CCTE_Deisenroth_DEVTOX_RUES2-GLR_Sox:

NAME: Stavudine
CHID: 23819 CASRN: 3056−17−5
SPID(S): ES211_58_58
M4ID: 42675913

HILL MODEL: Not applicable.

GAIN−LOSS MODEL: Not applicable.

	CNST	HILL	GNLS
AIC:	267.68	NA	NA
PROB:	1	NA	NA
RMSE:	9.25	NA	NA

MAX_MEAN: 13.5 MAX_MED: 11.4 BMAD: 6.75

COFF: 20.3 HIT−CALL: 0 FITC: 4 ACTP: 0

FLAGS:

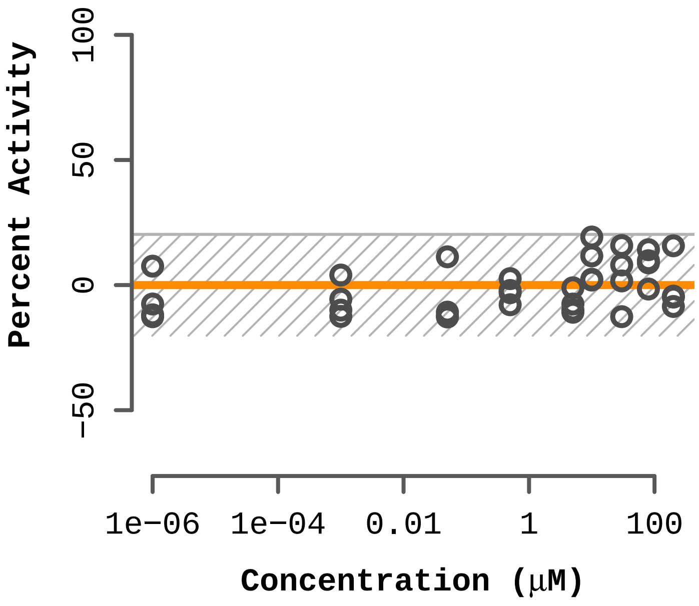

ASSAY: AEID3094 (CCTE_Deisenroth_DEVTOX_RUES2-GLR_Sox:

NAME: D-Camphor
CHID: 24721 CASRN: 464-49-3
SPID(S): ES211_27_27
M4ID: 42675938

HILL MODEL: Not applicable.

GAIN-LOSS MODEL: Not applicable.

	CNST	HILL	GNLS
AIC:	273.16	NA	NA
PROB:	1	NA	NA
RMSE:	9.67	NA	NA

MAX_MEAN: 8.81 MAX_MED: 9.33 BMAD: 6.75

COFF: 20.3 HIT-CALL: 0 FITC: 4 ACTP: 0

FLAGS:

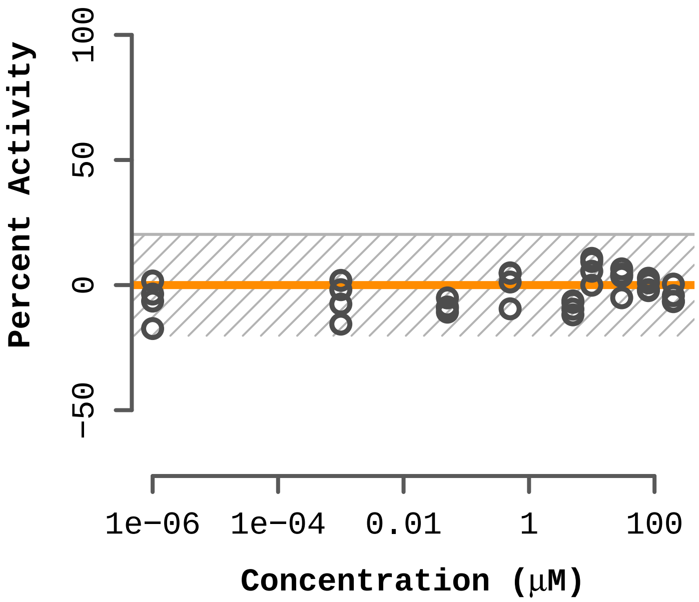

ASSAY: AEID3094 (CCTE_Deisenroth_DEVTOX_RUES2-GLR_Sox:

NAME: Cyclophosphamide monohydrate
CHID: 24888 CASRN: 6055-19-2
SPID(S): ES211_24_24
M4ID: 42675930

HILL MODEL: Not applicable.

GAIN-LOSS MODEL: Not applicable.

	CNST	HILL	GNLS
AIC:	249.82	NA	NA
PROB:	1	NA	NA
RMSE:	7.23	NA	NA

MAX_MEAN: 6.4 MAX_MED: 7.49 BMAD: 6.75

COFF: 20.3 HIT-CALL: 0 FITC: 4 ACTP: 0

FLAGS:

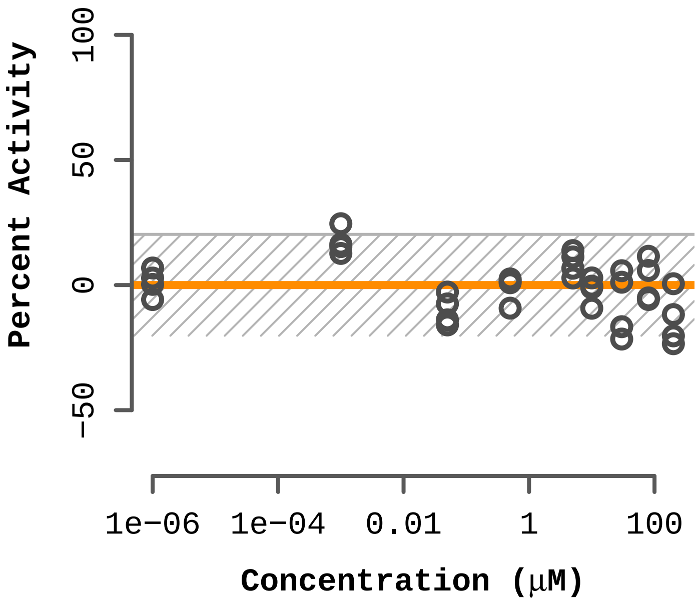

ASSAY: AEID3094 (CCTE_Deisenroth_DEVTOX_RUES2-GLR_Sox:

NAME: Hydroxyurea
CHID: 25438 CASRN: 127-07-1
SPID(S): ES211_35_35
M4ID: 42675909

HILL MODEL: Not applicable.

GAIN-LOSS MODEL: Not applicable.

	CNST	HILL	GNLS
AIC:	280.55	NA	NA
PROB:	1	NA	NA
RMSE:	11.22	NA	NA

MAX_MEAN: 17.2 MAX_MED: 15.9 BMAD: 6.75

COFF: 20.3 HIT-CALL: 0 FITC: 4 ACTP: 0

FLAGS:

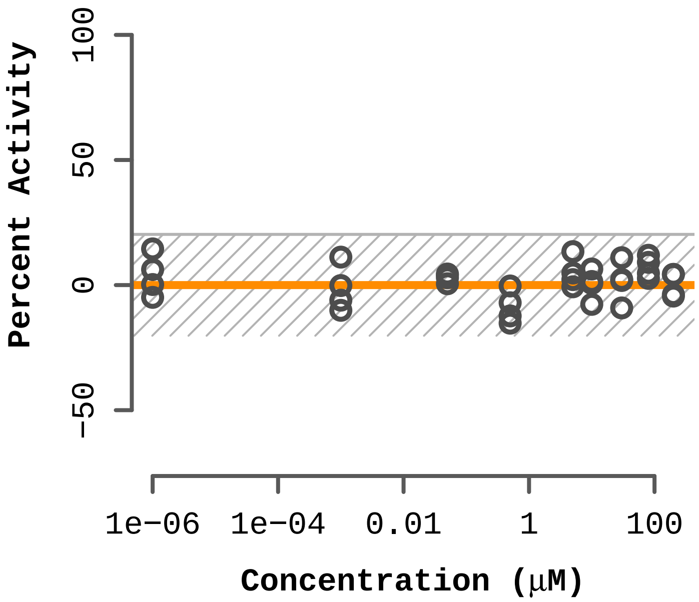

ASSAY: AEID3094 (CCTE_Deisenroth_DEVTOX_RUES2-GLR_Sox:

NAME: Salicylic acid
CHID: 26368 CASRN: 69−72−7
SPID(S): ES211_55_55
M4ID: 42675962

HILL MODEL: Not applicable.

GAIN−LOSS MODEL: Not applicable.

	CNST	HILL	GNLS
AIC:	248.94	NA	NA
PROB:	1	NA	NA
RMSE:	7.19	NA	NA

MAX_MEAN: 7.1 MAX_MED: 6.91 BMAD: 6.75

COFF: 20.3 HIT−CALL: 0 FITC: 4 ACTP: 0

FLAGS:

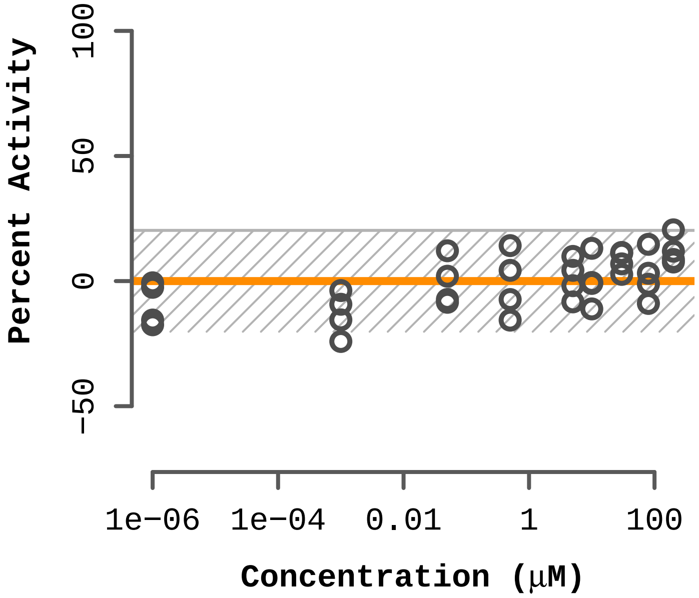

ASSAY: AEID3094 (CCTE_Deisenroth_DEVTOX_RUES2-GLR_Sox:

NAME: Penicillin G potassium
CHID: 45106 CASRN: 113−98−4
SPID(S): ES211_49_49
M4ID: 42675921

HILL MODEL: Not applicable.

GAIN−LOSS MODEL: Not applicable.

	CNST	HILL	GNLS
AIC:	278.39	NA	NA
PROB:	1	NA	NA
RMSE:	10.63	NA	NA

MAX_MEAN: 12.2 MAX_MED: 10.3 BMAD: 6.75

COFF: 20.3 HIT−CALL: 0 FITC: 4 ACTP: 0

FLAGS:

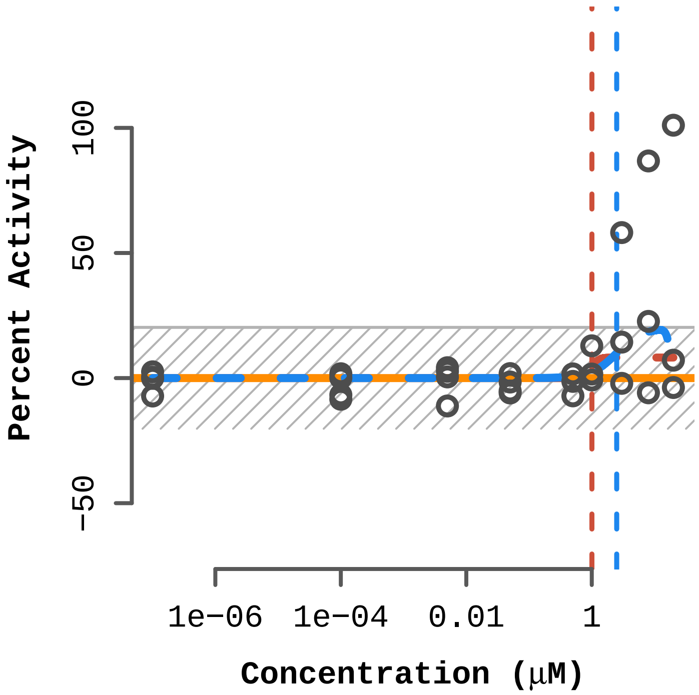

ASSAY: AEID3094 (CCTE_Deisenroth_DEVTOX_RUES2-GLR_Sox:

NAME: Cyclopamine
CHID: 43709 CASRN: 4449-51-8
SPID(S): ES211_23_23
M4ID: 42675968

HILL MODEL (in red):

	tp	ga	gw
val:	8.21	0.0021	8
sd:	5.08	0.147	24.6

GAIN-LOSS MODEL (in blue):

	tp	ga	gw	la	lw
val:	20	0.397	2.16	1.25	13.5
sd:	NaN	NaN	NaN	NaN	NaN

	CNST	HILL	GNLS
AIC:	261.4	263.7	266.33
PROB:	0.71	0.23	0.06
RMSE:	26.99	24.46	23.96

MAX_MEAN: 34.8 MAX_MED: 22.7 BMAD: 6.75

COFF: 20.3 HIT-CALL: 0 FITC: 9 ACTP: 0.29

FLAGS: 10

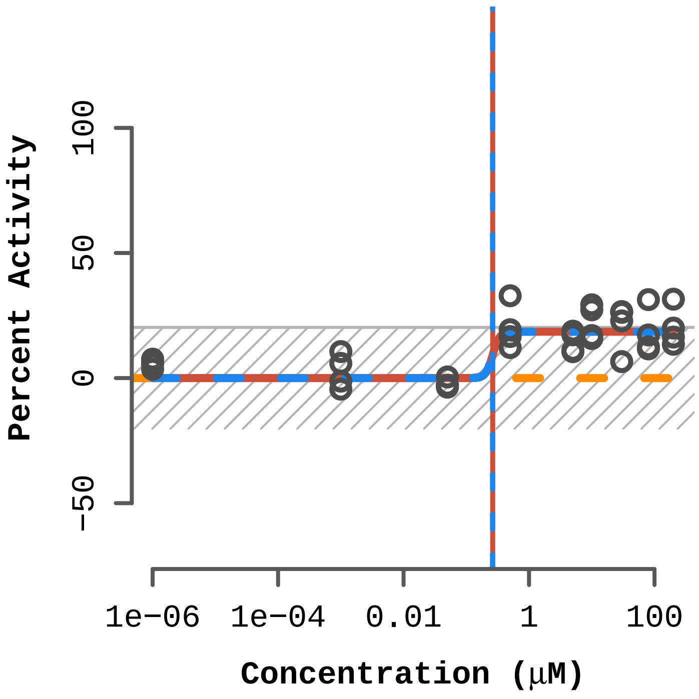

ASSAY: AEID3094 (CCTE_Deisenroth_DEVTOX_RUES2-GLR_Sox:

NAME: Methotrexate hydrate(1:x)
CHID: 1017640 CASRN: 133073-73-1
SPID(S): ES211_45_45
M4ID: 42675948

HILL MODEL (in red):

	tp	ga	gw
val:	18.5	-0.579	7.99
sd:	1.67	4.87	136

GAIN-LOSS MODEL (in blue):

	tp	ga	gw	la	lw
val:	18.5	-0.582	7.93	2.78	17.7
sd:	1.67	4.81	131	606	20800

	CNST	HILL	GNLS
AIC:	313.19	251.23	255.23
PROB:	0	0.88	0.12
RMSE:	17.19	6.77	6.77

MAX_MEAN: 22.4 MAX_MED: 24.6 BMAD: 6.75

COFF: 20.3 HIT-CALL: 0 FITC: 20 ACTP: 1

FLAGS: 8; 12

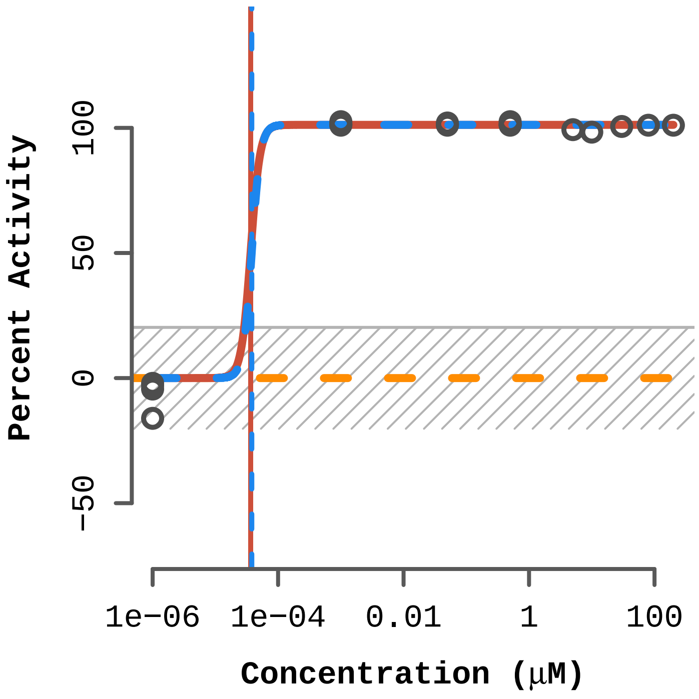

ASSAY: AEID3094 (CCTE_Deisenroth_DEVTOX_RUES2-GLR_Sox:

NAME: Actinomycin D
CHID: 20031 CASRN: 50−76−0
SPID(S): ES211_11_11
M4ID: 42675910

HILL MODEL (in red):

	tp	ga	gw
val:	101	−4.44	5.82
sd:	0.315	90.2	354

GAIN−LOSS MODEL (in blue):

	tp	ga	gw	la	lw
val:	101	−4.42	6.25	4.12	12.6
sd:	NA	NA	NA	NA	NA

	CNST	HILL	GNLS
AIC:	501.14	101.17	105.17
PROB:	0	0.88	0.12
RMSE:	91.05	3.83	3.83

MAX_MEAN: 102 MAX_MED: 101 BMAD: 6.75

COFF: 20.3 HIT−CALL: 1 FITC: 41 ACTP: 1

FLAGS:

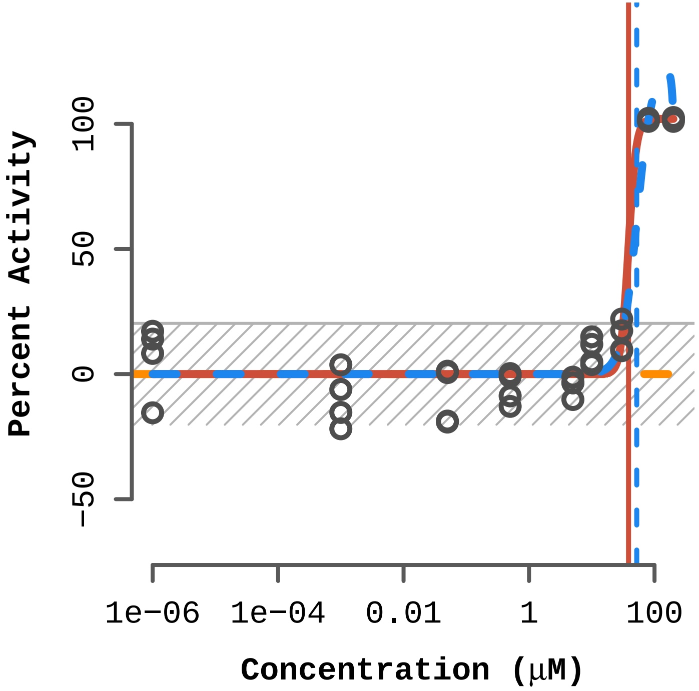

ASSAY: AEID3094 (CCTE_Deisenroth_DEVTOX_RUES2-GLR_Sox:

NAME: Bisphenol A
CHID: 20182 CASRN: 80-05-7
SPID(S): ES211_16_16
M4ID: 42675966

HILL MODEL (in red):

	tp	ga	gw
val:	102	1.59	7.16
sd:	5.43	0.282	18.3

GAIN-LOSS MODEL (in blue):

	tp	ga	gw	la	lw
val:	123	1.72	3.6	2.34	18
sd:	NaN	NaN	NaN	NaN	NaN

	CNST	HILL	GNLS
AIC:	377.01	271.59	275.29
PROB:	0	0.86	0.14
RMSE:	49.11	9.39	9.36

MAX_MEAN: 102 MAX_MED: 102 BMAD: 6.75

COFF: 20.3 HIT-CALL: 1 FITC: 41 ACTP: 1

FLAGS:

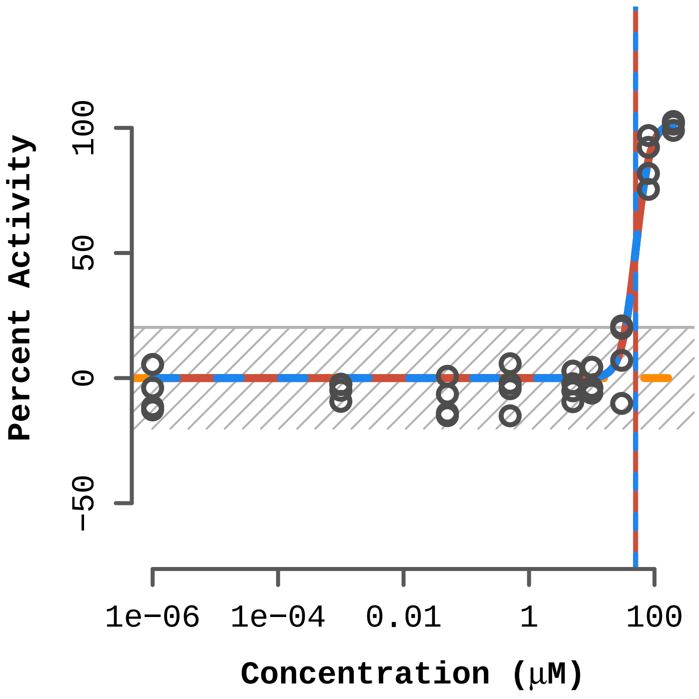

ASSAY: AEID3094 (CCTE_Deisenroth_DEVTOX_RUES2-GLR_Sox:

NAME: 4-Hydroxybenzoic acid butyl ester
CHID: 20209 CASRN: 94-26-8
SPID(S): ES211_19_19
M4ID: 42675935

HILL MODEL (in red):

	tp	ga	gw
val:	102	1.7	3.75
sd:	3.3	0.0385	0.645

GAIN-LOSS MODEL (in blue):

	tp	ga	gw	la	lw
val:	102	1.7	3.75	3.13	9.12
sd:	3.3	0.0385	0.645	1270	14000

	CNST	HILL	GNLS
AIC:	369.23	262.41	266.41
PROB:	0	0.88	0.12
RMSE:	45.27	8.17	8.17

MAX_MEAN: 101 MAX_MED: 102 BMAD: 6.75

COFF: 20.3 HIT-CALL: 1 FITC: 41 ACTP: 1

FLAGS:

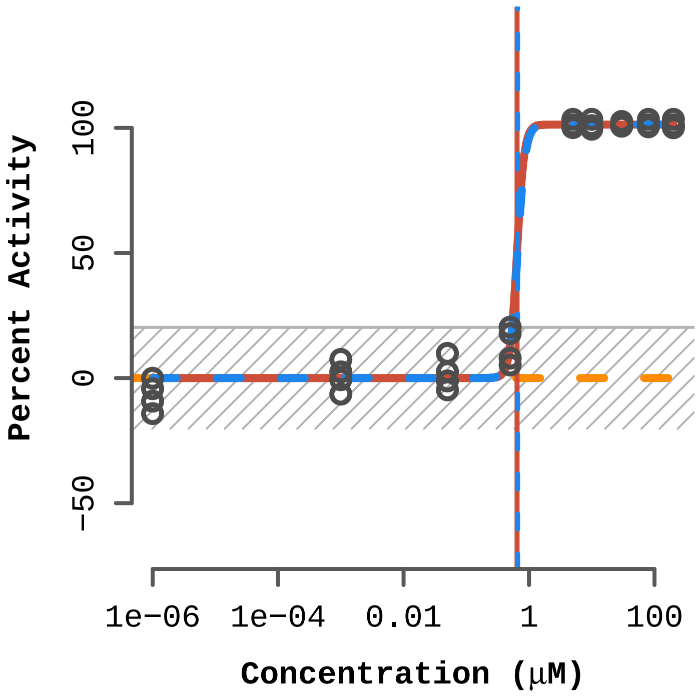

ASSAY: AEID3094 (CCTE_Deisenroth_DEVTOX_RUES2-GLR_Sox:

NAME: Diethylstilbestrol
CHID: 20465 CASRN: 56−53−1
SPID(S): ES211_29_29
M4ID: 42675961

HILL MODEL (in red):

	tp	ga	gw
val:	101	−0.191	7.51
sd:	0.637	2.5	170

GAIN−LOSS MODEL (in blue):

	tp	ga	gw	la	lw
val:	101	−0.181	6.91	4.3	6.43
sd:	0.637	3.17	182	173000	545000

	CNST	HILL	GNLS
AIC:	440.17	213.01	217.01
PROB:	0	0.88	0.12
RMSE:	75.79	4.49	4.49

MAX_MEAN: 101 MAX_MED: 101 BMAD: 6.75

COFF: 20.3 HIT−CALL: 1 FITC: 41 ACTP: 1

FLAGS:

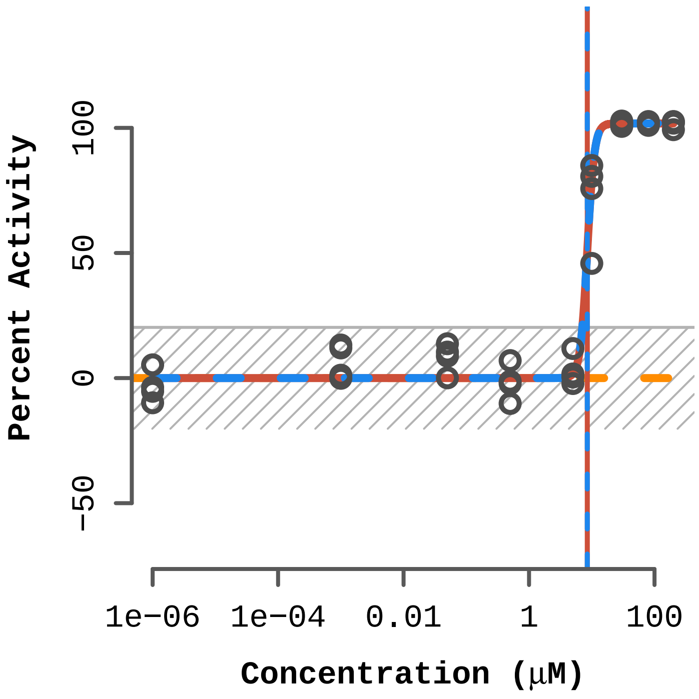

ASSAY: AEID3094 (CCTE_Deisenroth_DEVTOX_RUES2-GLR_Sox:

NAME: 5-Fluorouracil
CHID: 20634 CASRN: 51-21-8
SPID(S): ES211_5_5
M4ID: 42675906

HILL MODEL (in red):

	tp	ga	gw
val:	102	0.929	7.65
sd:	1.24	0.0226	2.29

GAIN-LOSS MODEL (in blue):

	tp	ga	gw	la	lw
val:	102	0.929	7.65	3.5	14.2
sd:	NA	NA	NA	NA	NA

	CNST	HILL	GNLS
AIC:	405.86	247.65	251.65
PROB:	0	0.88	0.12
RMSE:	63.9	8.02	8.02

MAX_MEAN: 102 MAX_MED: 102 BMAD: 6.75

COFF: 20.3 HIT-CALL: 1 FITC: 41 ACTP: 1

FLAGS:

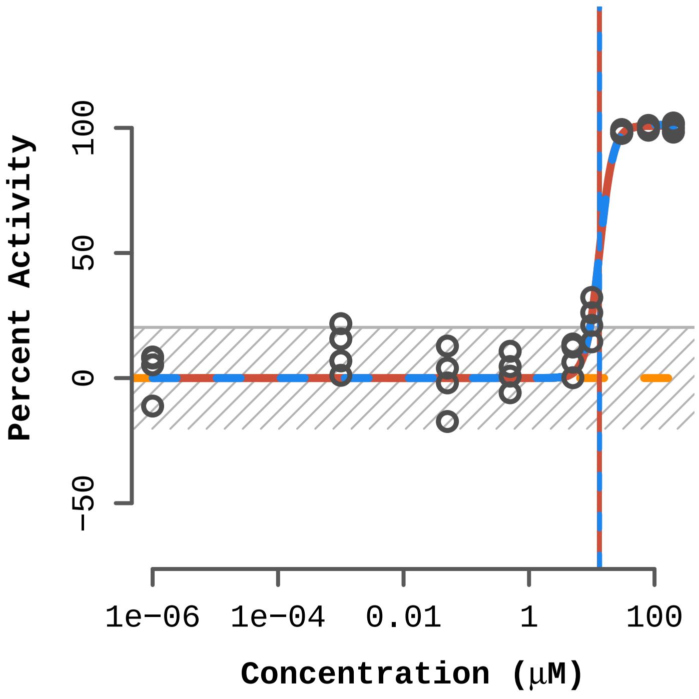

ASSAY: AEID3094 (CCTE_Deisenroth_DEVTOX_RUES2-GLR_Sox:

NAME: 1-(p-Chlorobenzoyl)-5-methoxy-2-methyl-Indole-:
CHID: 20740 CASRN: 53-86-1
SPID(S): ES211_38_38
M4ID: 42675911

HILL MODEL (in red):

	tp	ga	gw
val:	101	1.12	4.01
sd:	1.85	0.0276	0.795

GAIN-LOSS MODEL (in blue):

	tp	ga	gw	la	lw
val:	101	1.12	3.93	2.42	18
sd:	NaN	NaN	NaN	NaN	NaN

	CNST	HILL	GNLS
AIC:	397.37	256.47	260.41
PROB:	0	0.88	0.12
RMSE:	58.6	7.77	7.77

MAX_MEAN: 100 MAX_MED: 101 BMAD: 6.75

COFF: 20.3 HIT-CALL: 1 FITC: 41 ACTP: 1

FLAGS:

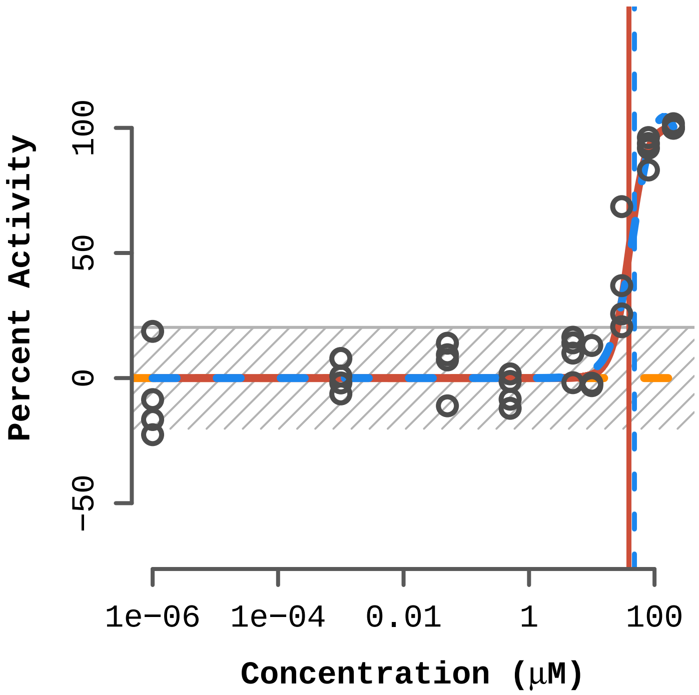

ASSAY: AEID3094 (CCTE_Deisenroth_DEVTOX_RUES2-GLR_Sox:

NAME: Busulfan
CHID: 20910 CASRN: 55−98−1
SPID(S): ES211_18_18
M4ID: 42675950

HILL MODEL (in red):

	tp	ga	gw
val:	101	1.59	3.17
sd:	4.24	0.0404	0.728

GAIN−LOSS MODEL (in blue):

	tp	ga	gw	la	lw
val:	122	1.68	2.36	2.67	2.09
sd:	NaN	NaN	NaN	NaN	NaN

	CNST	HILL	GNLS
AIC:	378.91	280.66	284.62
PROB:	0	0.88	0.12
RMSE:	48.2	11.11	11.1

MAX_MEAN: 100 MAX_MED: 100 BMAD: 6.75

COFF: 20.3 HIT−CALL: 1 FITC: 41 ACTP: 1

FLAGS:

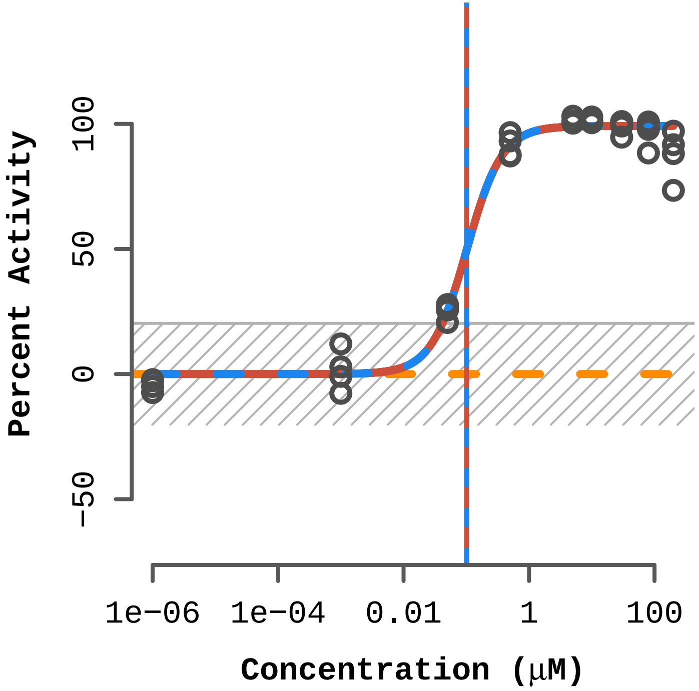

ASSAY: AEID3094 (CCTE_Deisenroth_DEVTOX_RUES2-GLR_Sox:

NAME: all-trans-Retinoic acid
CHID: 21239 CASRN: 302-79-4
SPID(S): ES211_13_13
M4ID: 42675942

HILL MODEL (in red):

	tp	ga	gw
val:	99.2	-0.995	1.53
sd:	1.03	0.0384	0.158

GAIN-LOSS MODEL (in blue):

	tp	ga	gw	la	lw
val:	99.2	-0.995	1.53	3.8	13.3
sd:	NA	NA	NA	NA	NA

	CNST	HILL	GNLS
AIC:	425.46	231.72	235.72
PROB:	0	0.88	0.12
RMSE:	79.17	6.31	6.31

MAX_MEAN: 101 MAX_MED: 101 BMAD: 6.75

COFF: 20.3 HIT-CALL: 1 FITC: 41 ACTP: 1

FLAGS:

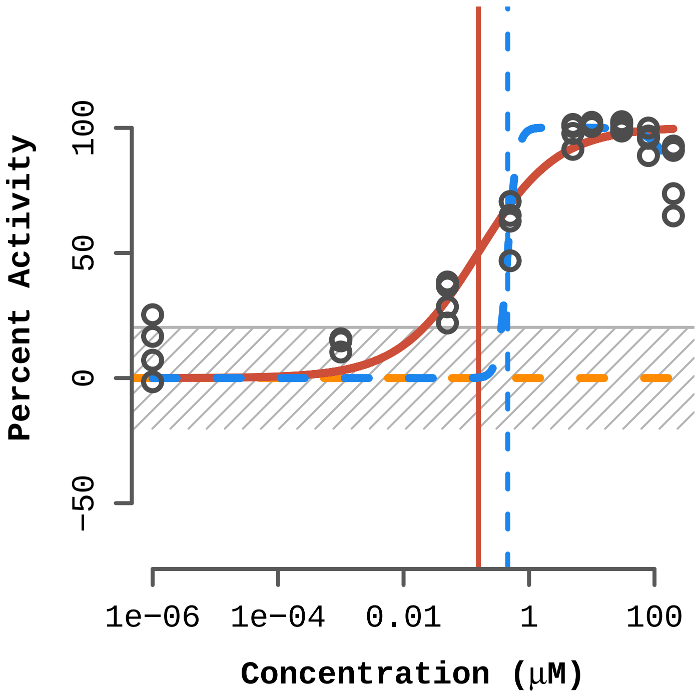

ASSAY: AEID3094 (CCTE_Deisenroth_DEVTOX_RUES2-GLR_Sox:

NAME: 13-cis Retinoic acid
CHID: 23177 CASRN: 4759-48-2
SPID(S): ES211_2_2
M4ID: 42675963

HILL MODEL (in red):

	tp	ga	gw
val:	100	-0.807	0.688
sd:	3.1	0.111	0.123

GAIN-LOSS MODEL (in blue):

	tp	ga	gw	la	lw
val:	100	-0.339	5.79	2.64	1.85
sd:	NaN	NaN	NaN	NaN	NaN

	CNST	HILL	GNLS
AIC:	421.64	283.01	300.17
PROB:	0	1	0
RMSE:	75.29	11.47	13.9

MAX_MEAN: 101 MAX_MED: 101 BMAD: 6.75

COFF: 20.3 HIT-CALL: 1 FITC: 41 ACTP: 1

FLAGS:

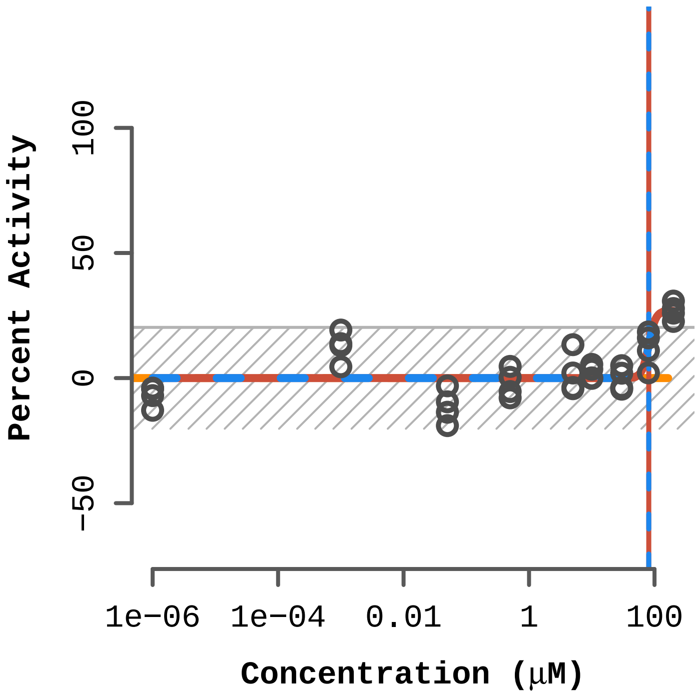

ASSAY: AEID3094 (CCTE_Deisenroth_DEVTOX_RUES2-GLR_Sox:

NAME: Warfarin
CHID: 23742 CASRN: 81-81-2
SPID(S): ES211_65_65
M4ID: 42675958

HILL MODEL (in red):

	tp	ga	gw
val:	26.8	1.91	8
sd:	3.14	0.0471	46.7

GAIN-LOSS MODEL (in blue):

	tp	ga	gw	la	lw
val:	26.8	1.91	8	3.9	7.79
sd:	NA	NA	NA	NA	NA

	CNST	HILL	GNLS
AIC:	286.97	259.79	263.79
PROB:	0	0.88	0.12
RMSE:	12.59	7.94	7.94

MAX_MEAN: 26.8 MAX_MED: 26.9 BMAD: 6.75

COFF: 20.3 HIT-CALL: 1 FITC: 41 ACTP: 1

FLAGS: 7; 17

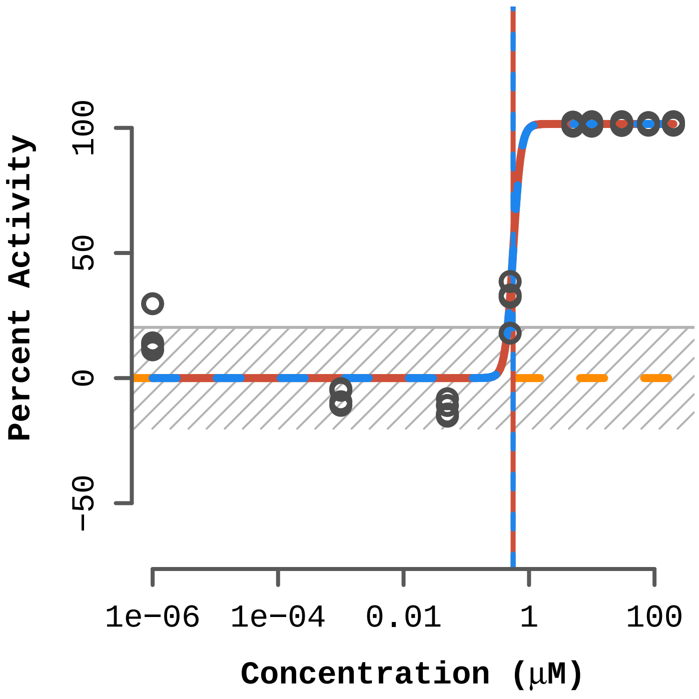

ASSAY: AEID3094 (CCTE_Deisenroth_DEVTOX_RUES2-GLR_Sox:

NAME: Cytarabine hydrochloride
CHID: 24891 CASRN: 69−74−9
SPID(S): ES211_25_25
M4ID: 42675965

HILL MODEL (in red):

	tp	ga	gw
val:	102	−0.254	6.89
sd:	1.05	0.474	69.1

GAIN−LOSS MODEL (in blue):

	tp	ga	gw	la	lw
val:	102	−0.253	6.83	3.09	10.2
sd:	1.05	0.653	93.3	794	10300

	CNST	HILL	GNLS
AIC:	512.8	255.28	259.28
PROB:	0	0.88	0.12
RMSE:	76.84	8.36	8.36

MAX_MEAN: 102 MAX_MED: 102 BMAD: 6.75

COFF: 20.3 HIT−CALL: 1 FITC: 41 ACTP: 1

FLAGS:

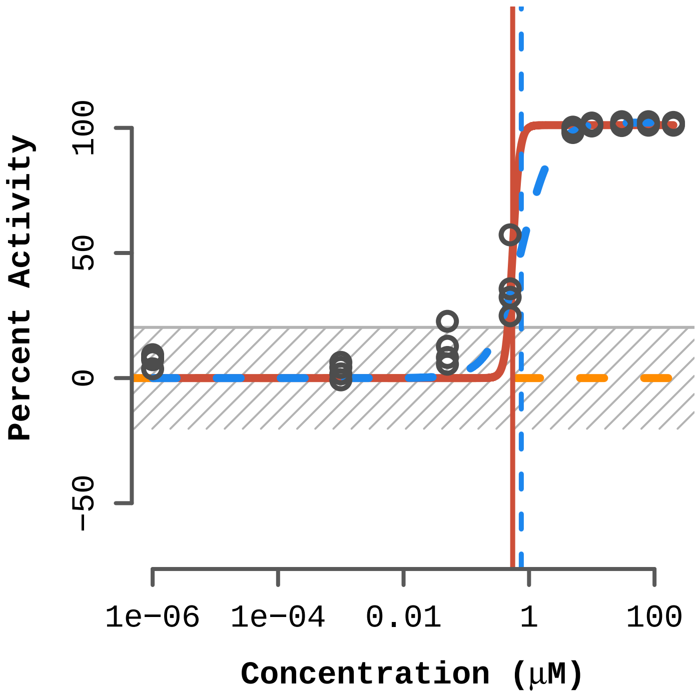

ASSAY: AEID3094 (CCTE_Deisenroth_DEVTOX_RUES2-GLR_Sox:

NAME: 5-Bromo-2'-deoxyuridine
CHID: 33105 CASRN: 59-14-3
SPID(S): ES211_4_4
M4ID: 42675922

HILL MODEL (in red):

	tp	ga	gw
val:	101	-0.261	7.77
sd:	0.758	0.372	71.6

GAIN-LOSS MODEL (in blue):

	tp	ga	gw	la	lw
val:	102	-0.123	1.74	2.48	12.5
sd:	1.23	0.0351	0.272	0.151	8.9

	CNST	HILL	GNLS
AIC:	448.66	234.53	235.1
PROB:	0	0.57	0.43
RMSE:	76.67	6.96	6.71

MAX_MEAN: 102 MAX_MED: 102 BMAD: 6.75

COFF: 20.3 HIT-CALL: 1 FITC: 41 ACTP: 1

FLAGS:

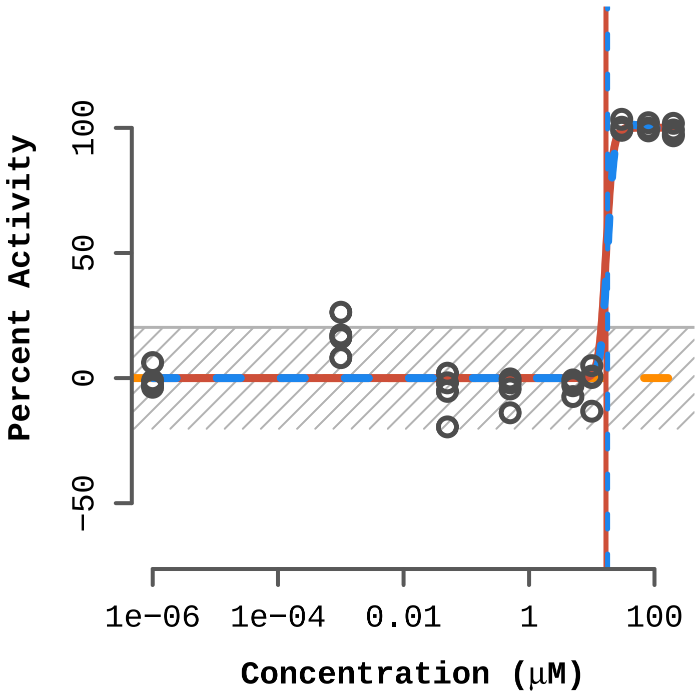

ASSAY: AEID3094 (CCTE_Deisenroth_DEVTOX_RUES2-GLR_Sox:

NAME: Amiodarone hydrochloride
CHID: 37185 CASRN: 19774-82-4
SPID(S): ES211_14_14
M4ID: 42675960

HILL MODEL (in red):

	tp	ga	gw
val:	100	1.23	8
sd:	1.4	0.0567	1.9

GAIN-LOSS MODEL (in blue):

	tp	ga	gw	la	lw
val:	103	1.25	8	4.3	0.677
sd:	13	0.0905	2.88	5.53	2.47

	CNST	HILL	GNLS
AIC:	396.09	250.63	253.87
PROB:	0	0.84	0.16
RMSE:	58.16	8.09	8.05

MAX_MEAN: 100 MAX_MED: 99.7 BMAD: 6.75

COFF: 20.3 HIT-CALL: 1 FITC: 41 ACTP: 1

FLAGS:

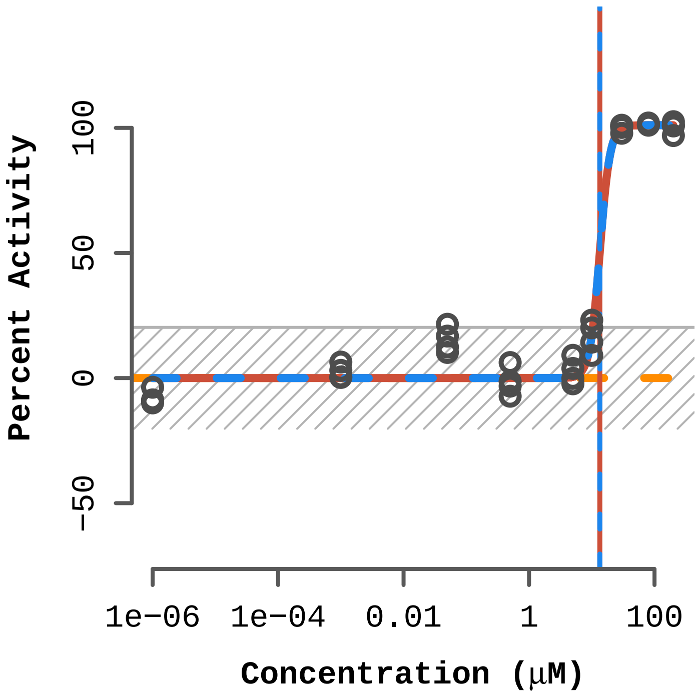

ASSAY: AEID3094 (CCTE_Deisenroth_DEVTOX_RUES2-GLR_Sox:

NAME: Imatinib mesylate
CHID: 40502 CASRN: 220127-57-1
SPID(S): ES211_37_37
M4ID: 42675929

HILL MODEL (in red):

	tp	ga	gw
val:	101	1.13	5.37
sd:	1.56	0.0447	1.78

GAIN-LOSS MODEL (in blue):

	tp	ga	gw	la	lw
val:	101	1.13	5.37	4.29	12.8
sd:	NA	NA	NA	NA	NA

	CNST	HILL	GNLS
AIC:	396.96	244.24	248.24
PROB:	0	0.88	0.12
RMSE:	58.73	6.8	6.8

MAX_MEAN: 101 MAX_MED: 101 BMAD: 6.75

COFF: 20.3 HIT-CALL: 1 FITC: 41 ACTP: 1

FLAGS:

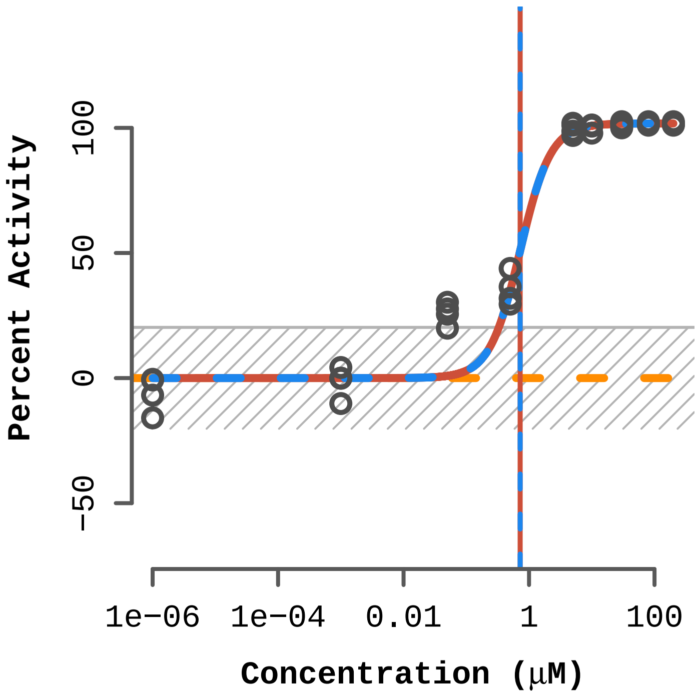

ASSAY: AEID3094 (CCTE_Deisenroth_DEVTOX_RUES2-GLR_Sox[illegible]

NAME: Dasatinib
CHID: 40979 CASRN: 302962-49-8
SPID(S): ES211_26_26
M4ID: 42675949

HILL MODEL (in red):

	tp	ga	gw
val:	102	−0.142	1.78
sd:	1.04	0.0377	0.343

GAIN-LOSS MODEL (in blue):

	tp	ga	gw	la	lw
val:	102	−0.142	1.78	4.2	11.2
sd:	NA	NA	NA	NA	NA

	CNST	HILL	GNLS
AIC:	432.03	251.71	255.71
PROB:	0	0.88	0.12
RMSE:	76.76	9.33	9.33

MAX_MEAN: 102 MAX_MED: 102 BMAD: 6.75

COFF: 20.3 HIT-CALL: 1 FITC: 41 ACTP: 1

FLAGS:

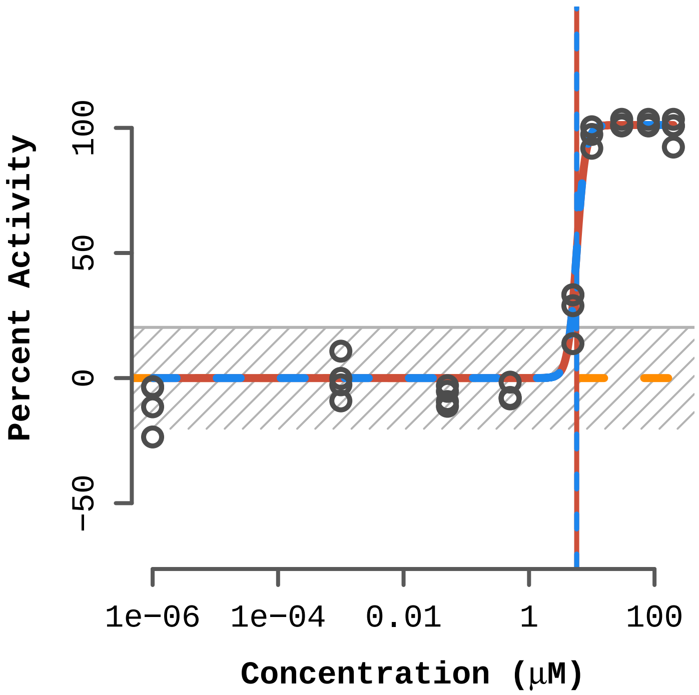

ASSAY: AEID3094 (CCTE_Deisenroth_DEVTOX_RUES2-GLR_Sox:

NAME: Gefitinib
CHID: 41034 CASRN: 184475-35-2
SPID(S): ES211_34_34
M4ID: 42675943

HILL MODEL (in red):

	tp	ga	gw
val:	101	0.76	6.11
sd:	1.36	0.0154	1.29

GAIN-LOSS MODEL (in blue):

	tp	ga	gw	la	lw
val:	101	0.76	6.11	3.59	17.2
sd:	NA	NA	NA	NA	NA

	CNST	HILL	GNLS
AIC:	411.1	246.31	250.31
PROB:	0	0.88	0.12
RMSE:	67.66	7.06	7.06

MAX_MEAN: 102 MAX_MED: 101 BMAD: 6.75

COFF: 20.3 HIT-CALL: 1 FITC: 41 ACTP: 1

FLAGS:

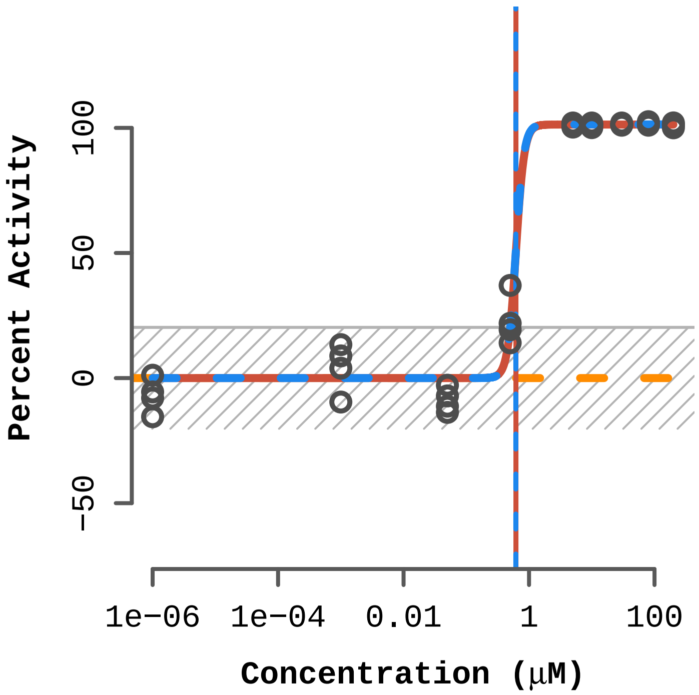

ASSAY: AEID3094 (CCTE_Deisenroth_DEVTOX_RUES2-GLR_Sox:

NAME: Sorafenib
CHID: 41128 CASRN: 284461-73-0
SPID(S): ES211_57_57
M4ID: 42675940

HILL MODEL (in red):

	tp	ga	gw
val:	101	-0.209	6.54
sd:	0.783	2.6	184

GAIN-LOSS MODEL (in blue):

	tp	ga	gw	la	lw
val:	101	-0.21	6.68	2.52	11.5
sd:	0.883	1.16	85.7	0.331	18.5

	CNST	HILL	GNLS
AIC:	501.52	235.35	239.33
PROB:	0	0.88	0.12
RMSE:	76.16	6.25	6.25

MAX_MEAN: 102 MAX_MED: 102 BMAD: 6.75

COFF: 20.3 HIT-CALL: 1 FITC: 41 ACTP: 1

FLAGS:

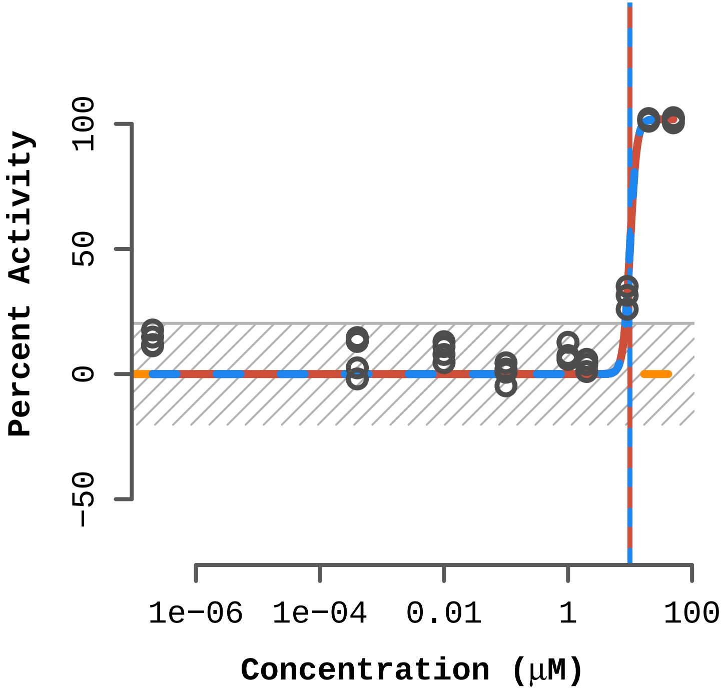

ASSAY: AEID3094 (CCTE_Deisenroth_DEVTOX_RUES2-GLR_Sox:

NAME: Ritanserin
CHID: 42594 CASRN: 87051-43-2
SPID(S): ES211_53_53
M4ID: 42675947

HILL MODEL (in red):

	tp	ga	gw
val:	102	0.999	8
sd:	2.62	0.0414	7.14

GAIN-LOSS MODEL (in blue):

	tp	ga	gw	la	lw
val:	102	0.999	8	3.61	16
sd:	NA	NA	NA	NA	NA

	CNST	HILL	GNLS
AIC:	319.97	225.87	229.87
PROB:	0	0.88	0.12
RMSE:	46.41	7.94	7.94

MAX_MEAN: 102 MAX_MED: 102 BMAD: 6.75

COFF: 20.3 HIT-CALL: 1 FITC: 41 ACTP: 1

FLAGS:

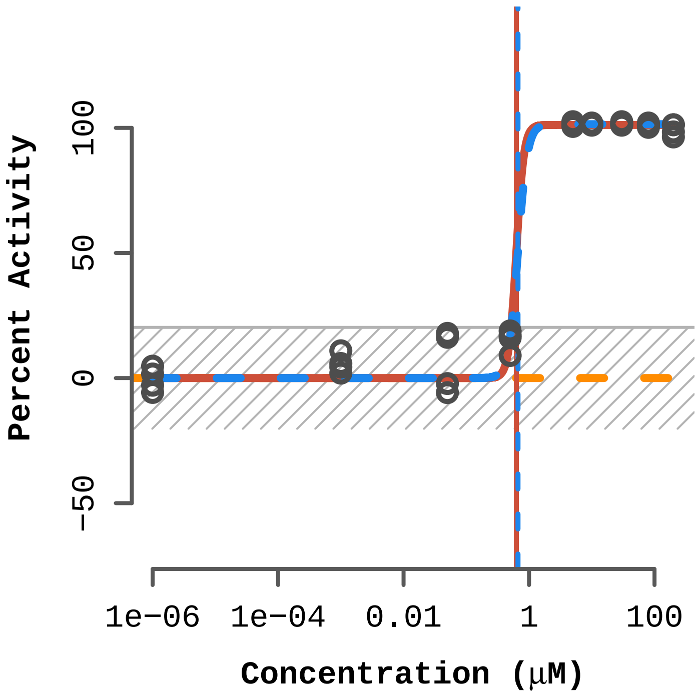

ASSAY: AEID3094 (CCTE_Deisenroth_DEVTOX_RUES2-GLR_Sox

NAME: Sunitinib malate
CHID: 46492 CASRN: 341031-54-7
SPID(S): ES211_60_60
M4ID: 42675918

HILL MODEL (in red):

	tp	ga	gw
val:	101	-0.202	7.2
sd:	0.589	0.922	67

GAIN-LOSS MODEL (in blue):

	tp	ga	gw	la	lw
val:	101	-0.176	5.72	2.4	17.9
sd:	NaN	NaN	NaN	NaN	NaN

	CNST	HILL	GNLS
AIC:	446.04	213.91	216.28
PROB:	0	0.77	0.23
RMSE:	75.66	5.18	5.14

MAX_MEAN: 102 MAX_MED: 102 BMAD: 6.75

COFF: 20.3 HIT-CALL: 1 FITC: 41 ACTP: 1

FLAGS:

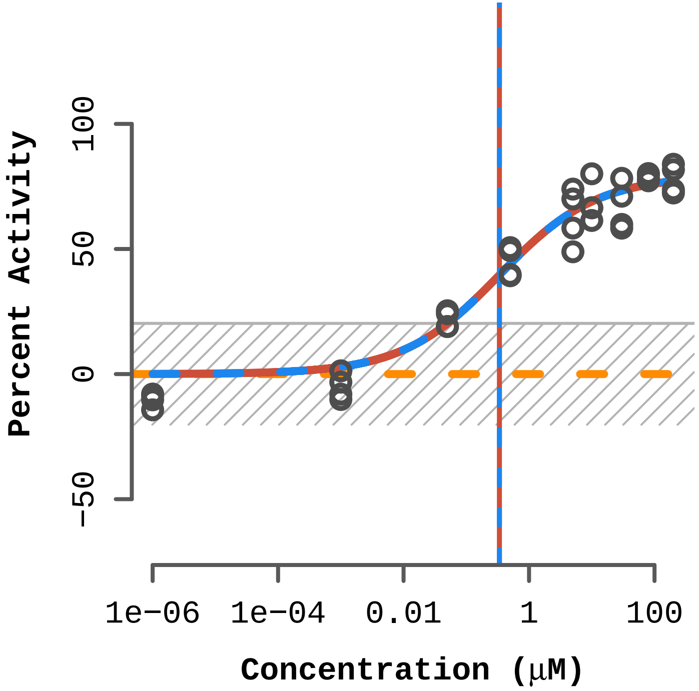

ASSAY: AEID3094 (CCTE_Deisenroth_DEVTOX_RUES2-GLR_Sox:

NAME: Lenalidomide
CHID: 46664 CASRN: 191732-72-6
SPID(S): ES211_41_41
M4ID: 42675941

HILL MODEL (in red):

	tp	ga	gw
val:	79.2	-0.472	0.561
sd:	3.72	0.158	0.0897

GAIN-LOSS MODEL (in blue):

	tp	ga	gw	la	lw
val:	79.2	-0.472	0.561	4.3	11.6
sd:	NA	NA	NA	NA	NA

	CNST	HILL	GNLS
AIC:	400.18	260.37	264.37
PROB:	0	0.88	0.12
RMSE:	56.09	7.67	7.67

MAX_MEAN: 78.8 MAX_MED: 78.9 BMAD: 6.75

COFF: 20.3 HIT-CALL: 1 FITC: 41 ACTP: 1

FLAGS:

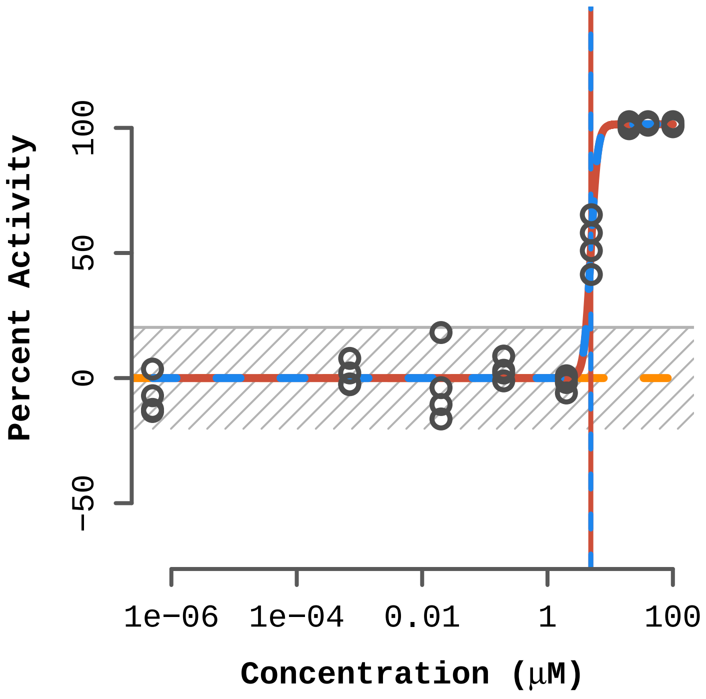

ASSAY: AEID3094 (CCTE_Deisenroth_DEVTOX_RUES2-GLR_Sox:

NAME: Vandetanib
CHID: 46681 CASRN: 443913-73-3
SPID(S): ES211_64_64
M4ID: 42675964

HILL MODEL (in red):

	tp	ga	gw
val:	101	0.691	8
sd:	1.22	0.0111	7.01

GAIN-LOSS MODEL (in blue):

	tp	ga	gw	la	lw
val:	101	0.691	8	4	18
sd:	NA	NA	NA	NA	NA

	CNST	HILL	GNLS
AIC:	402.56	244.06	248.06
PROB:	0	0.88	0.12
RMSE:	61.64	6.78	6.78

MAX_MEAN: 102 MAX_MED: 102 BMAD: 6.75

COFF: 20.3 HIT-CALL: 1 FITC: 41 ACTP: 1

FLAGS:

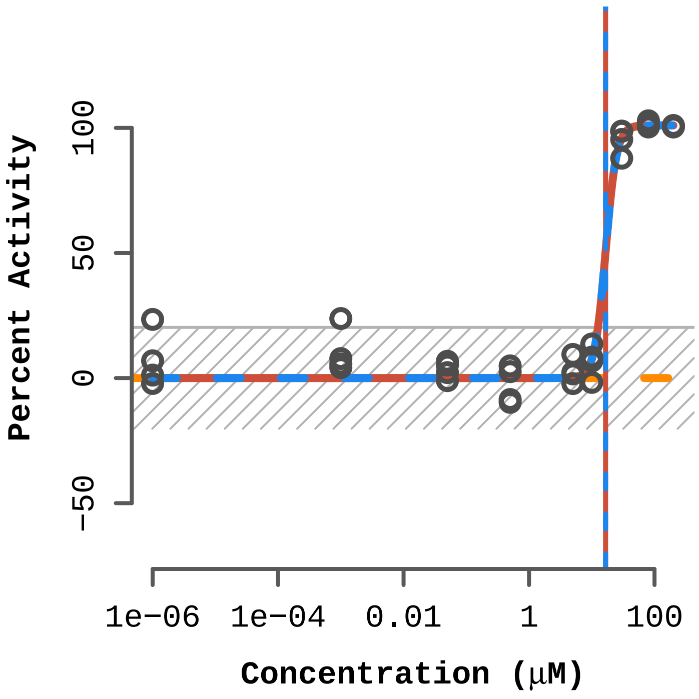

ASSAY: AEID3094 (CCTE_Deisenroth_DEVTOX_RUES2-GLR_Sox:

NAME: 6-aminopyridine-3-carboxamide
CHID: 51446 CASRN: 329-89-5
SPID(S): ES211_7_7
M4ID: 42675967

HILL MODEL (in red):

	tp	ga	gw
val:	101	1.22	4.95
sd:	1.46	0.0304	0.648

GAIN-LOSS MODEL (in blue):

	tp	ga	gw	la	lw
val:	101	1.22	4.95	3.38	8.79
sd:	NaN	NaN	NaN	NaN	NaN

	CNST	HILL	GNLS
AIC:	395.36	242.51	246.51
PROB:	0	0.88	0.12
RMSE:	57.69	7.2	7.2

MAX_MEAN: 101 MAX_MED: 101 BMAD: 6.75

COFF: 20.3 HIT-CALL: 1 FITC: 41 ACTP: 1

FLAGS:

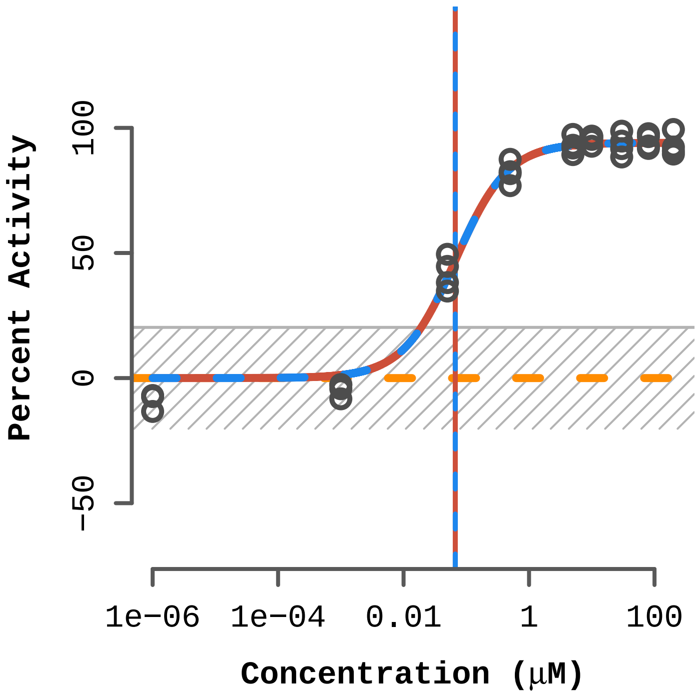

ASSAY: AEID3094 (CCTE_Deisenroth_DEVTOX_RUES2-GLR_Sox:

NAME: Pomalidomide
CHID: 893458 CASRN: 19171-19-8
SPID(S): ES211_50_50
M4ID: 42675933

HILL MODEL (in red):

	tp	ga	gw
val:	94	−1.18	1.03
sd:	1.1	0.0525	0.116

GAIN-LOSS MODEL (in blue):

	tp	ga	gw	la	lw
val:	94	−1.18	1.03	4.3	17.9
sd:	NA	NA	NA	NA	NA

	CNST	HILL	GNLS
AIC:	423.35	228.14	232.14
PROB:	0	0.88	0.12
RMSE:	76.56	4.98	4.98

MAX_MEAN: 95.3 MAX_MED: 95.9 BMAD: 6.75

COFF: 20.3 HIT-CALL: 1 FITC: 41 ACTP: 1

FLAGS:

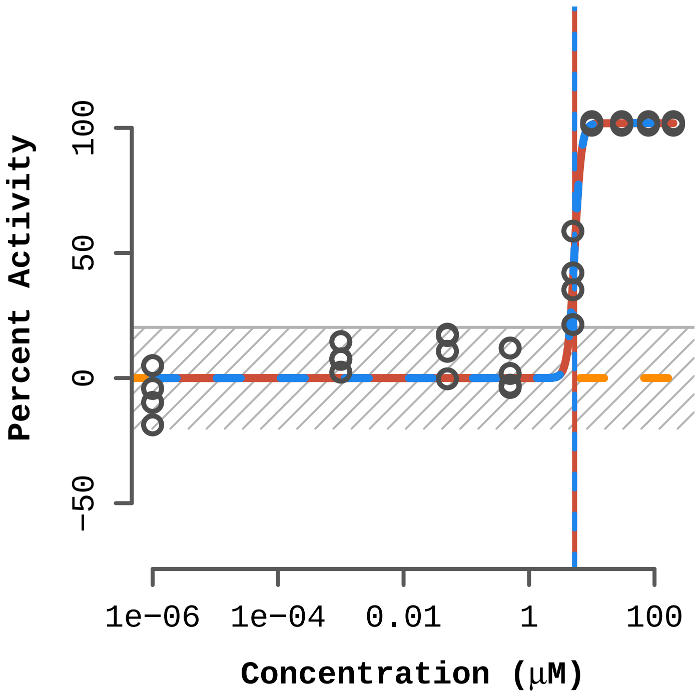

ASSAY: AEID3094 (CCTE_Deisenroth_DEVTOX_RUES2-GLR_Sox:

NAME: Tegaserod maleate
CHID: 904761 CASRN: 189188-57-6
SPID(S): ES211_61_61
M4ID: 42675944

HILL MODEL (in red):

	tp	ga	gw
val:	102	0.726	8
sd:	1.33	0.018	4.46

GAIN-LOSS MODEL (in blue):

	tp	ga	gw	la	lw
val:	102	0.726	8	2.45	18
sd:	NaN	NaN	NaN	NaN	NaN

	CNST	HILL	GNLS
AIC:	413.53	257.26	261.25
PROB:	0	0.88	0.12
RMSE:	69.54	8.22	8.22

MAX_MEAN: 102 MAX_MED: 102 BMAD: 6.75

COFF: 20.3 HIT-CALL: 1 FITC: 41 ACTP: 1

FLAGS:

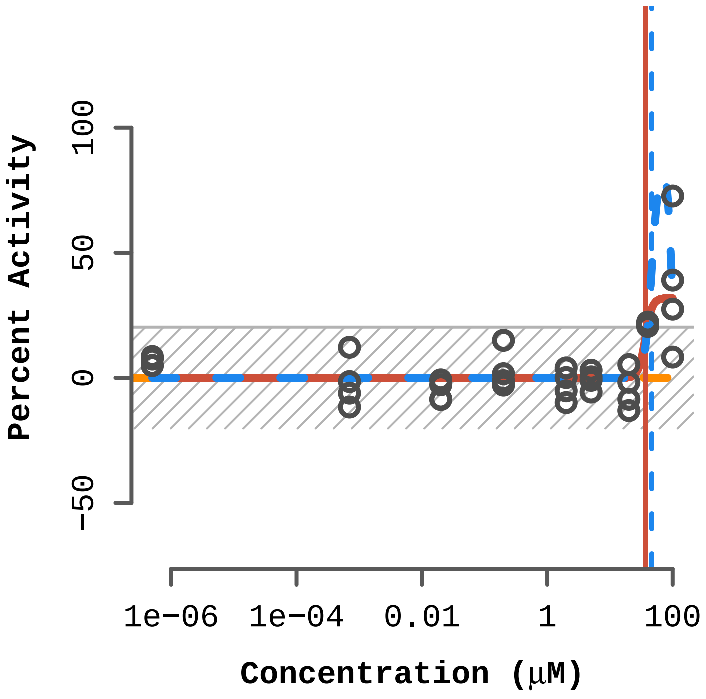

ASSAY: AEID3094 (CCTE_Deisenroth_DEVTOX_RUES2-GLR_Sox:

NAME: Ketanserin tartrate
CHID: 1003945 CASRN: 83846-83-7
SPID(S): ES211_40_40
M4ID: 42675959

HILL MODEL (in red):

	tp	ga	gw
val:	31.8	1.56	8
sd:	5.94	0.041	5.16

GAIN-LOSS MODEL (in blue):

	tp	ga	gw	la	lw
val:	87.2	1.67	7.65	1.98	11.8
sd:	NaN	NaN	NaN	NaN	NaN

	CNST	HILL	GNLS
AIC:	295.8	265.65	269.58
PROB:	0	0.88	0.12
RMSE:	17.34	10.08	10.09

MAX_MEAN: 36.9 MAX_MED: 33.3 BMAD: 6.75

COFF: 20.3 HIT-CALL: 1 FITC: 41 ACTP: 1

FLAGS: 17

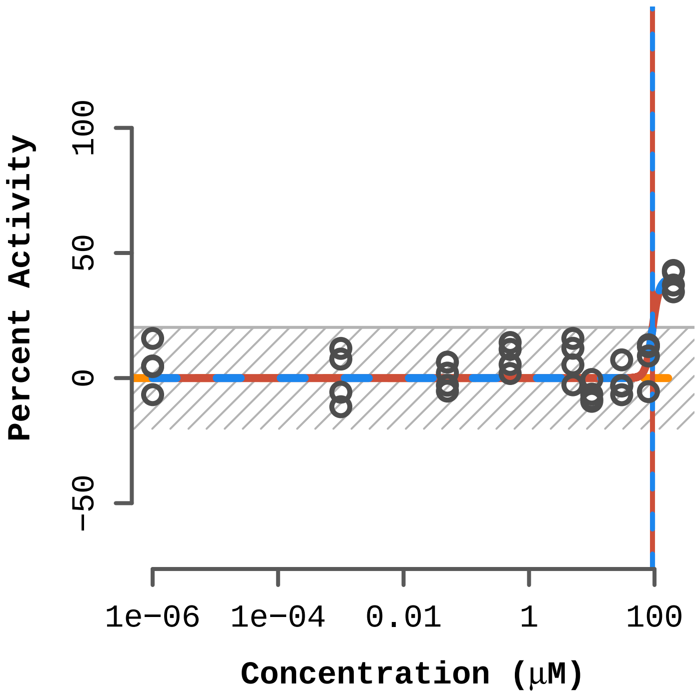


```
ASSAY:    AEID3094 (CCTE_Deisenroth_DEVTOX_RUES2-GLR_Sox:

NAME:     (2R,3S)-2-(3,4-Dihydroxyphenyl)-3,4-dihydro-2H-
CHID:     1017388 CASRN: 225937-10-0
SPID(S):  ES211_22_22
M4ID:     42675971

HILL MODEL (in red):
      tp        ga        gw
val:  39.4      1.97      8
sd:   4.54      0.31      37.5

GAIN-LOSS MODEL (in blue):
      tp        ga        gw        la        lw
val:  39.7      1.97      8         2.78      4.32
sd:   NaN       NaN       NaN       NaN       NaN

      CNST        HILL        GNLS
AIC:  295         261.35      265.35
PROB: 0           0.88        0.12
RMSE: 15.42       7.79        7.79

MAX_MEAN: 39.3      MAX_MED: 39.8      BMAD: 6.75

COFF: 20.3   HIT-CALL: 1    FITC: 41   ACTP: 1

FLAGS: 7; 17
```

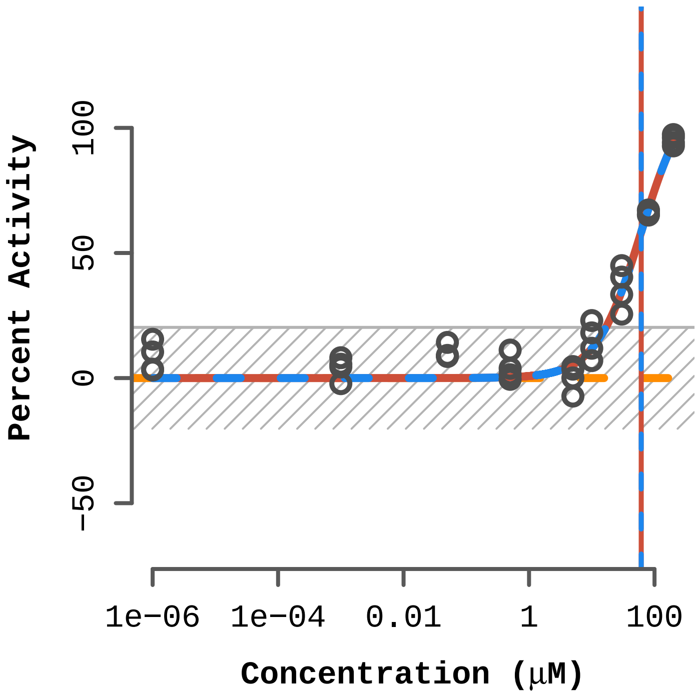

ASSAY: AEID3094 (CCTE_Deisenroth_DEVTOX_RUES2-GLR_Sox:

NAME: Diphenhydramine hydrochloride
CHID: 20537 CASRN: 147-24-0
SPID(S): ES211_31_31
M4ID: 42675916

HILL MODEL (in red):

	tp	ga	gw
val:	117	1.79	1.2
sd:	11	0.089	0.157

GAIN-LOSS MODEL (in blue):

	tp	ga	gw	la	lw
val:	117	1.79	1.2	3.2	11.4
sd:	11	0.089	0.157	3410	43000

	CNST	HILL	GNLS
AIC:	367.59	251.02	255.02
PROB:	0	0.88	0.12
RMSE:	41.28	6.87	6.87

MAX_MEAN: 95.1 MAX_MED: 95.1 BMAD: 6.75

COFF: 20.3 HIT-CALL: 1 FITC: 42 ACTP: 1

FLAGS:

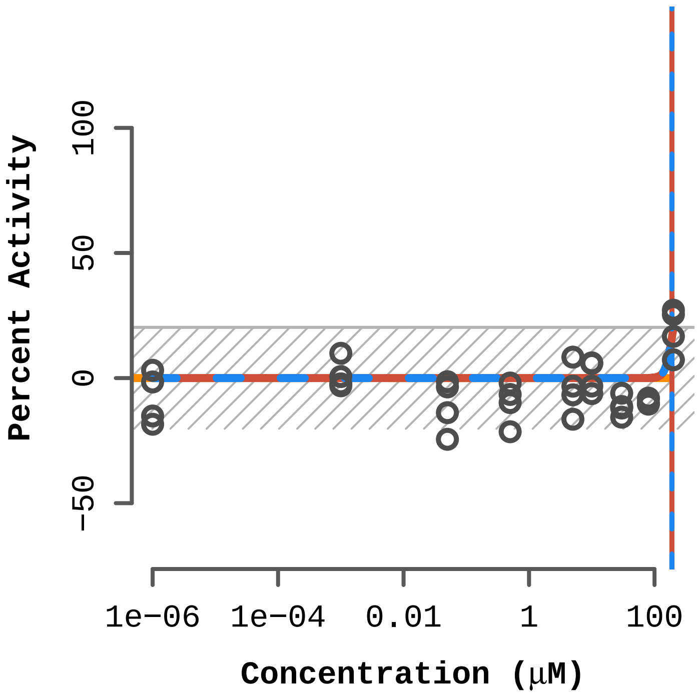

ASSAY: AEID3094 (CCTE_Deisenroth_DEVTOX_RUES2-GLR_Sox

NAME: 5,5-Diphenylhydantoin
CHID: 20541 CASRN: 57-41-0
SPID(S): ES211_3_3
M4ID: 42675955

HILL MODEL (in red):

	tp	ga	gw
val:	32.6	2.28	8
sd:	105	0.424	11.8

GAIN-LOSS MODEL (in blue):

	tp	ga	gw	la	lw
val:	32.6	2.28	8	3.37	18
sd:	NA	NA	NA	NA	NA

	CNST	HILL	GNLS
AIC:	285.71	280.38	284.38
PROB:	0.06	0.83	0.11
RMSE:	12.05	10.22	10.22

MAX_MEAN: 19.1 MAX_MED: 21 BMAD: 6.75

COFF: 20.3 HIT-CALL: 1 FITC: 42 ACTP: 0.94

FLAGS: 7; 11; 17

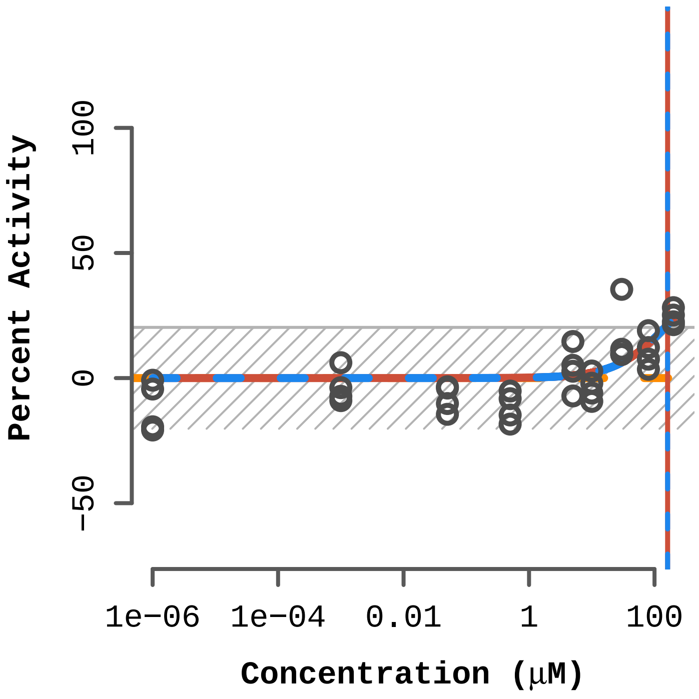

ASSAY: AEID3094 (CCTE_Deisenroth_DEVTOX_RUES2-GLR_Sox:

NAME: Ibuprofen
CHID: 20732 CASRN: 15687-27-1
SPID(S): ES211_36_36
M4ID: 42675917

HILL MODEL (in red):

	tp	ga	gw
val:	42.6	2.21	1.05
sd:	49.5	0.979	0.591

GAIN-LOSS MODEL (in blue):

	tp	ga	gw	la	lw
val:	42.6	2.21	1.05	3.56	9.76
sd:	49.5	0.979	0.591	88700	662000

	CNST	HILL	GNLS
AIC:	295.77	275.33	279.33
PROB:	0	0.88	0.12
RMSE:	13.92	10.02	10.02

MAX_MEAN: 24.3 MAX_MED: 23.9 BMAD: 6.75

COFF: 20.3 HIT-CALL: 1 FITC: 42 ACTP: 1

FLAGS: 7; 11; 17

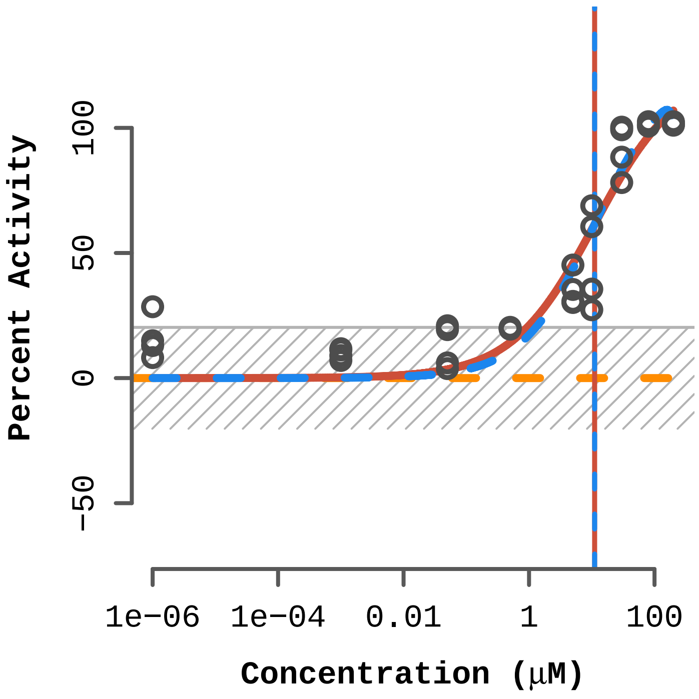

ASSAY: AEID3094 (CCTE_Deisenroth_DEVTOX_RUES2-GLR_Sox:

NAME: Lovastatin
CHID: 20784 CASRN: 75330-75-5
SPID(S): ES211_42_42
M4ID: 42675956

HILL MODEL (in red):

	tp	ga	gw
val:	123	1.05	0.656
sd:	15.5	0.205	0.191

GAIN-LOSS MODEL (in blue):

	tp	ga	gw	la	lw
val:	123	1.05	0.753	2.42	9.02
sd:	NaN	NaN	NaN	NaN	NaN

	CNST	HILL	GNLS
AIC:	403.65	293.04	294.84
PROB:	0	0.71	0.29
RMSE:	61.47	12.46	12.16

MAX_MEAN: 102 MAX_MED: 102 BMAD: 6.75

COFF: 20.3 HIT-CALL: 1 FITC: 42 ACTP: 1

FLAGS:

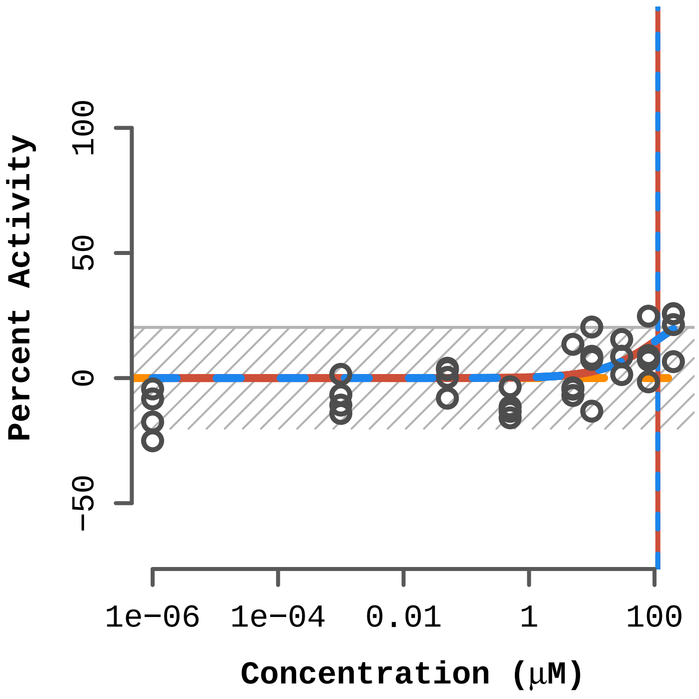

ASSAY: AEID3094 (CCTE_Deisenroth_DEVTOX_RUES2-GLR_Sox:

NAME: Methyldopa sesquihydrate
CHID: 20863 CASRN: 41372-08-1
SPID(S): ES211_46_46
M4ID: 42675934

HILL MODEL (in red):

	tp	ga	gw
val:	30.9	2.05	0.978
sd:	30.6	0.949	0.582

GAIN-LOSS MODEL (in blue):

	tp	ga	gw	la	lw
val:	30.9	2.05	0.978	4.3	3.51
sd:	30.7	0.951	0.583	1050	1870

	CNST	HILL	GNLS
AIC:	291.48	280.52	284.52
PROB:	0	0.88	0.12
RMSE:	12.91	10.17	10.17

MAX_MEAN: 19.9 MAX_MED: 23.5 BMAD: 6.75

COFF: 20.3 HIT-CALL: 1 FITC: 42 ACTP: 1

FLAGS: 7; 11; 17

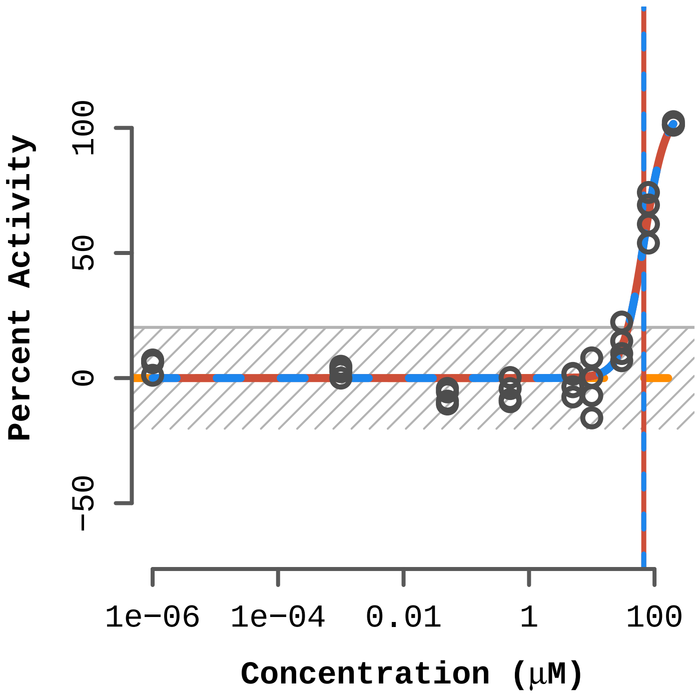

ASSAY: AEID3094 (CCTE_Deisenroth_DEVTOX_RUES2-GLR_Sox

NAME: Rifampicin
CHID: 21244 CASRN: 13292-46-1
SPID(S): ES211_52_52
M4ID: 42675932

HILL MODEL (in red):

	tp	ga	gw
val:	108	1.83	2.54
sd:	4.64	0.033	0.366

GAIN-LOSS MODEL (in blue):

	tp	ga	gw	la	lw
val:	108	1.83	2.54	2.74	16.4
sd:	NaN	NaN	NaN	NaN	NaN

	CNST	HILL	GNLS
AIC:	359.69	245.9	249.9
PROB:	0	0.88	0.12
RMSE:	40.9	6.37	6.37

MAX_MEAN: 102 MAX_MED: 101 BMAD: 6.75

COFF: 20.3 HIT-CALL: 1 FITC: 42 ACTP: 1

FLAGS:

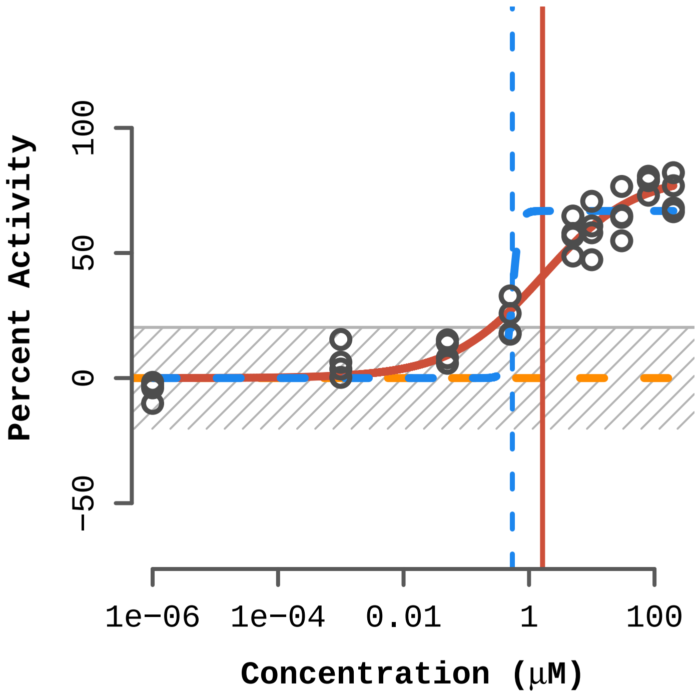

ASSAY: AEID3094 (CCTE_Deisenroth_DEVTOX_RUES2-GLR_Sox:

NAME: Thalidomide
CHID: 22524 CASRN: 50-35-1
SPID(S): ES211_62_62
M4ID: 42675912

HILL MODEL (in red):

	tp	ga	gw
val:	81.4	0.216	0.59
sd:	5.23	0.153	0.101

GAIN-LOSS MODEL (in blue):

	tp	ga	gw	la	lw
val:	66.8	-0.266	7.74	4.02	5.24
sd:	NaN	NaN	NaN	NaN	NaN

	CNST	HILL	GNLS
AIC:	392.55	252.75	280.47
PROB:	0	1	0
RMSE:	51.11	6.91	9.5

MAX_MEAN: 78 MAX_MED: 79.1 BMAD: 6.75

COFF: 20.3 HIT-CALL: 1 FITC: 42 ACTP: 1

FLAGS:

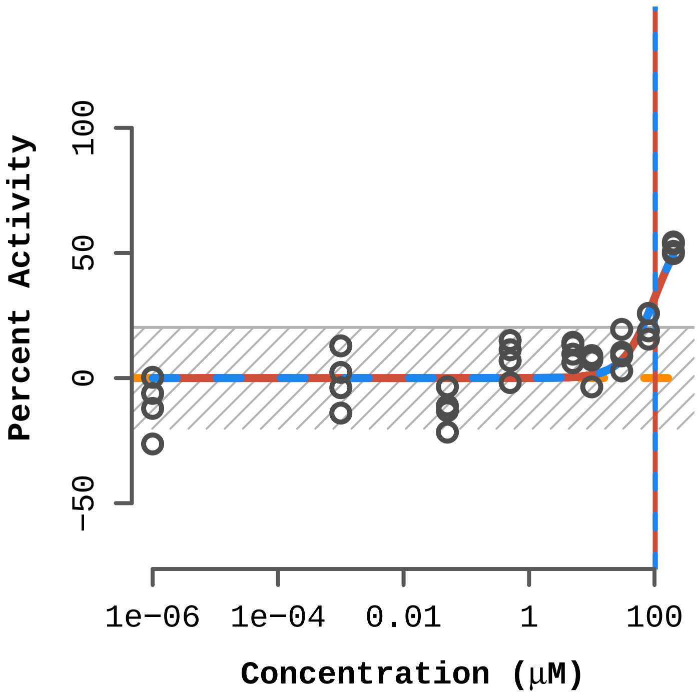

ASSAY: AEID3094 (CCTE_Deisenroth_DEVTOX_RUES2-GLR_Sox:

NAME: Carbamazepine
CHID: 22731 CASRN: 298−46−4
SPID(S): ES211_21_21
M4ID: 42675970

HILL MODEL (in red):

	tp	ga	gw
val:	65.3	2.01	1.69
sd:	14.8	0.139	0.686

GAIN−LOSS MODEL (in blue):

	tp	ga	gw	la	lw
val:	65.3	2.01	1.69	3.38	18
sd:	NA	NA	NA	NA	NA

	CNST	HILL	GNLS
AIC:	321.73	276.21	280.21
PROB:	0	0.88	0.12
RMSE:	21.44	9.8	9.8

MAX_MEAN: 52.1 MAX_MED: 52.2 BMAD: 6.75

COFF: 20.3 HIT−CALL: 1 FITC: 42 ACTP: 1

FLAGS:

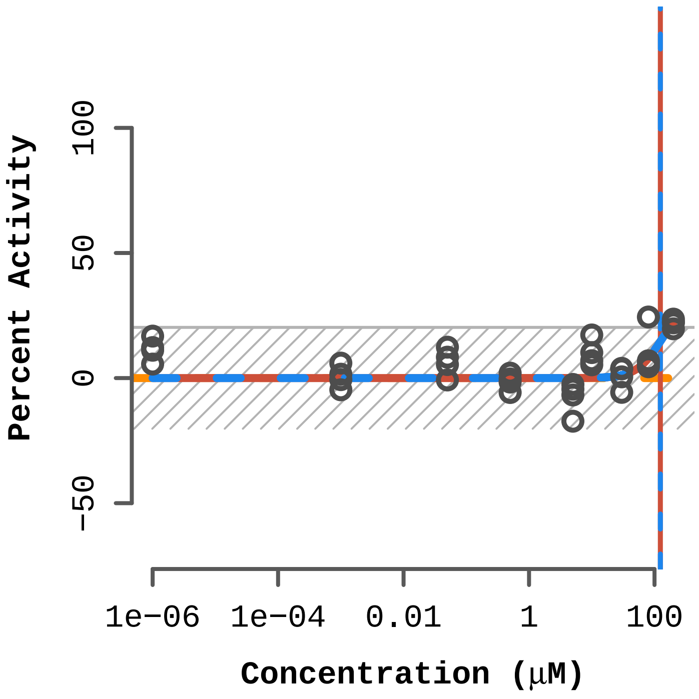

ASSAY: AEID3094 (CCTE_Deisenroth_DEVTOX_RUES2-GLR_Sox:

NAME: Valproic acid
CHID: 23733 CASRN: 99-66-1
SPID(S): ES211_63_63
M4ID: 42675923

HILL MODEL (in red):

	tp	ga	gw
val:	29.3	2.09	2.25
sd:	33.7	0.577	3.32

GAIN-LOSS MODEL (in blue):

	tp	ga	gw	la	lw
val:	29.3	2.09	2.25	3.2	8.18
sd:	33.7	0.577	3.32	1230	11200

	CNST	HILL	GNLS
AIC:	278.68	256.82	260.82
PROB:	0	0.88	0.12
RMSE:	11.16	7.7	7.7

MAX_MEAN: 22 MAX_MED: 22.3 BMAD: 6.75

COFF: 20.3 HIT-CALL: 1 FITC: 42 ACTP: 1

FLAGS: 7; 11; 17

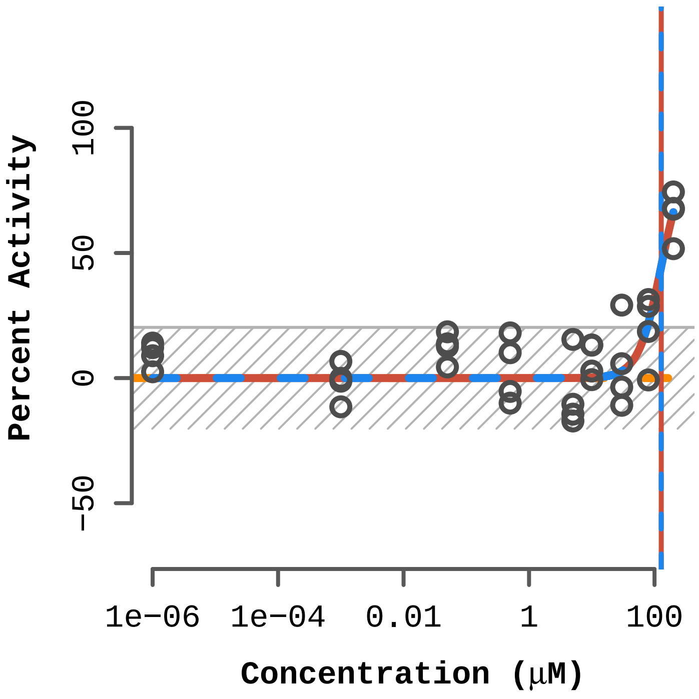

ASSAY: AEID3094 (CCTE_Deisenroth_DEVTOX_RUES2-GLR_Sox:

NAME: MEHP
CHID: 25680 CASRN: 4376-20-9
SPID(S): ES211_44_44
M4ID: 42675969

HILL MODEL (in red):

	tp	ga	gw
val:	89.3	2.11	2.37
sd:	129	0.708	3.93

GAIN-LOSS MODEL (in blue):

	tp	ga	gw	la	lw
val:	89.3	2.11	2.37	4.12	6.33
sd:	NaN	NaN	NaN	NaN	NaN

	CNST	HILL	GNLS
AIC:	330.17	291.71	295.71
PROB:	0	0.88	0.12
RMSE:	25.62	11.72	11.72

MAX_MEAN: 65.4 MAX_MED: 67.7 BMAD: 6.75

COFF: 20.3 HIT-CALL: 1 FITC: 42 ACTP: 1

FLAGS:

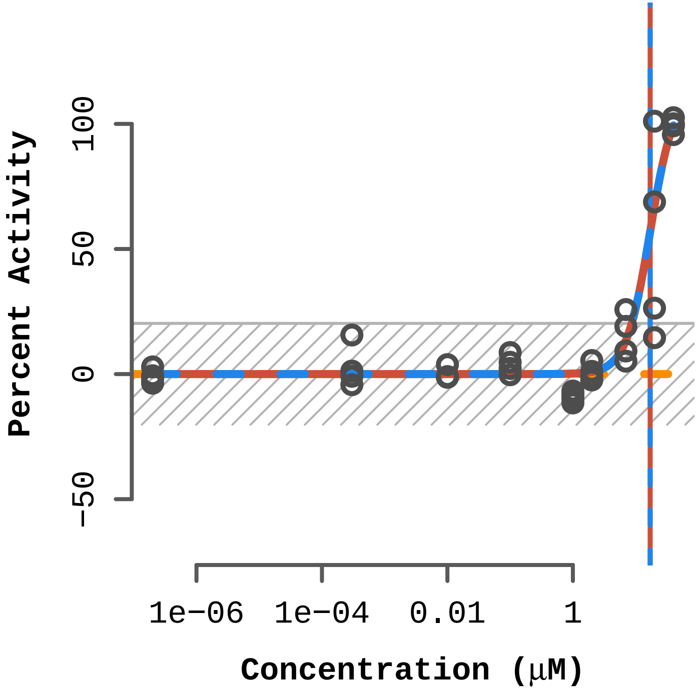

ASSAY: AEID3094 (CCTE_Deisenroth_DEVTOX_RUES2-GLR_Sox:

NAME: Dorsomorphin
CHID: 1006988 CASRN: 866405-64-3
SPID(S): ES211_6_6
M4ID: 42675952

HILL MODEL (in red):

	tp	ga	gw
val:	114	1.23	2.25
sd:	11.8	0.0752	0.472

GAIN-LOSS MODEL (in blue):

	tp	ga	gw	la	lw
val:	114	1.23	2.25	2.33	9.53
sd:	11.8	0.0752	0.472	489	6410

	CNST	HILL	GNLS
AIC:	351.83	274.88	278.88
PROB:	0	0.88	0.12
RMSE:	39.9	13.61	13.61

MAX_MEAN: 99.4 MAX_MED: 99.6 BMAD: 6.75

COFF: 20.3 HIT-CALL: 1 FITC: 42 ACTP: 1

FLAGS:

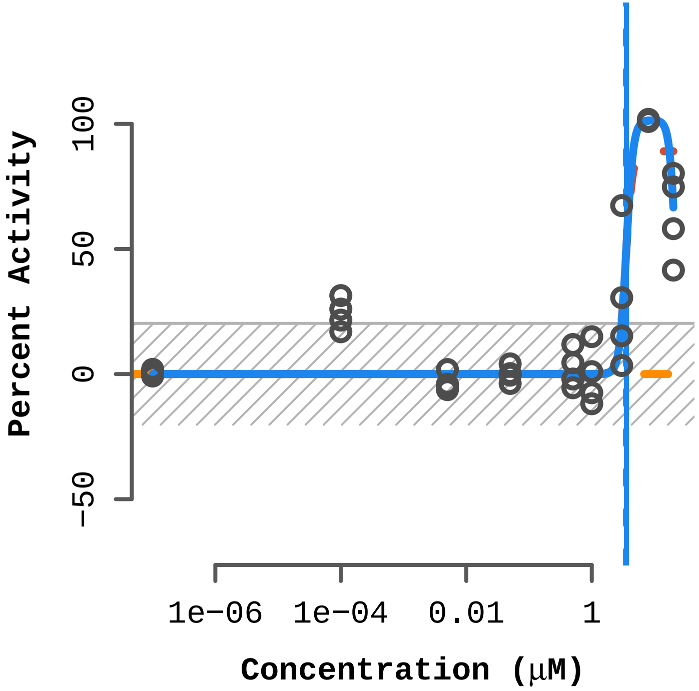

ASSAY: AEID3094 (CCTE_Deisenroth_DEVTOX_RUES2-GLR_Sox:

NAME: Ziprasidone
CHID: 23753 CASRN: 146939-27-7
SPID(S): ES211_66_66
M4ID: 42675936

HILL MODEL (in red):

	tp	ga	gw
val:	89.1	0.538	8
sd:	6.69	0.047	5.24

GAIN-LOSS MODEL (in blue):

	tp	ga	gw	la	lw
val:	101	0.552	7.99	1.33	9.06
sd:	NaN	NaN	NaN	NaN	NaN

	CNST	HILL	GNLS
AIC:	368.85	307.7	295.46
PROB:	0	0	1
RMSE:	43.15	16.48	13.66

MAX_MEAN: 101 MAX_MED: 101 BMAD: 6.75

COFF: 20.3 HIT-CALL: 1 FITC: 50 ACTP: 1

FLAGS:

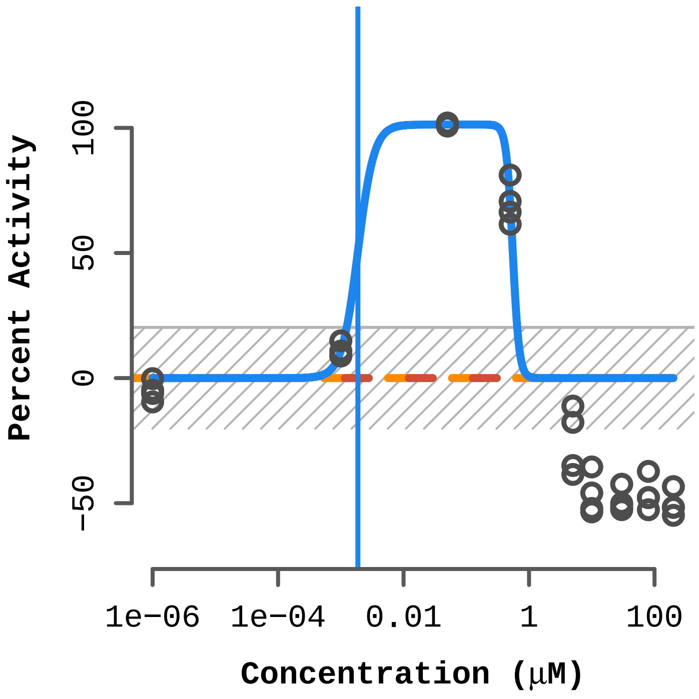


```
ASSAY:     AEID3094 (CCTE_Deisenroth_DEVTOX_RUES2-GLR_Sox:

NAME:      Adriamycin hydrochloride
CHID:      30636    CASRN: 25316-40-9
SPID(S):   ES211_67_67
M4ID:      42675905

HILL MODEL (in red):
      tp        ga        gw
val:  81.1      2.8       8
sd:   NaN       NaN       NaN

GAIN-LOSS MODEL (in blue):
      tp        ga        gw        la        lw
val:  101       -2.73     3.38      -0.26     8.39
sd:   NaN       NaN       NaN       NaN       NaN

      CNST         HILL         GNLS
AIC:  394.89       400.89       371.68
PROB: 0            0            1
RMSE: 53.29        53.29        33.8

MAX_MEAN: 101       MAX_MED: 101       BMAD: 6.75

COFF: 20.3   HIT-CALL: 1    FITC: 50   ACTP: 1

FLAGS: 10
```

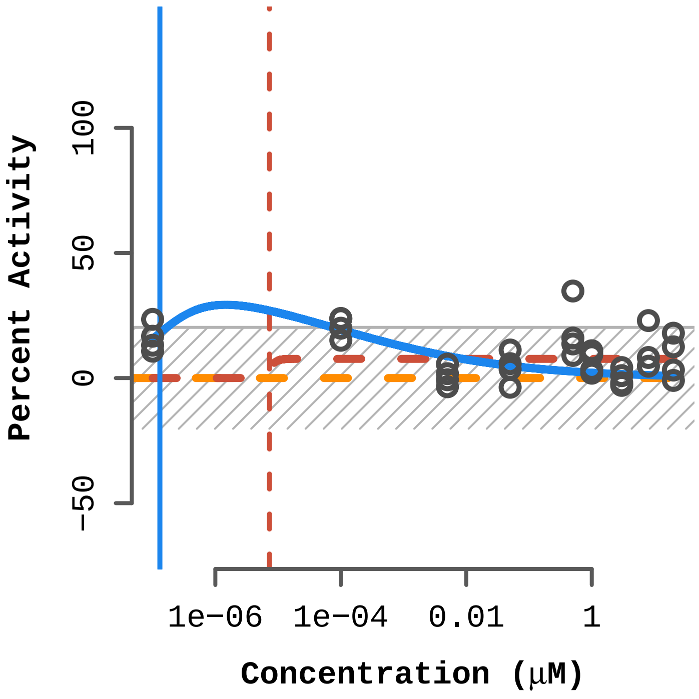

ASSAY: AEID3094 (CCTE_Deisenroth_DEVTOX_RUES2-GLR_Sox:

NAME: Dexamethasone sodium phosphate
CHID: 47429 CASRN: 2392-39-4
SPID(S): ES211_28_28
M4ID: 42675920

HILL MODEL (in red):

	tp	ga	gw
val:	7.67	−5.14	7.58
sd:	1.83	1670	11100

GAIN-LOSS MODEL (in blue):

	tp	ga	gw	la	lw
val:	41.8	−6.88	1.07	−4.22	0.3
sd:	NaN	NaN	NaN	NaN	NaN

	CNST	HILL	GNLS
AIC:	283.94	274.02	263.66
PROB:	0	0.01	0.99
RMSE:	13.32	10.52	9.39

MAX_MEAN: 20.7 MAX_MED: 21.8 BMAD: 6.75

COFF: 20.3 HIT-CALL: 1 FITC: 50 ACTP: 1

FLAGS: 7; 11; 17

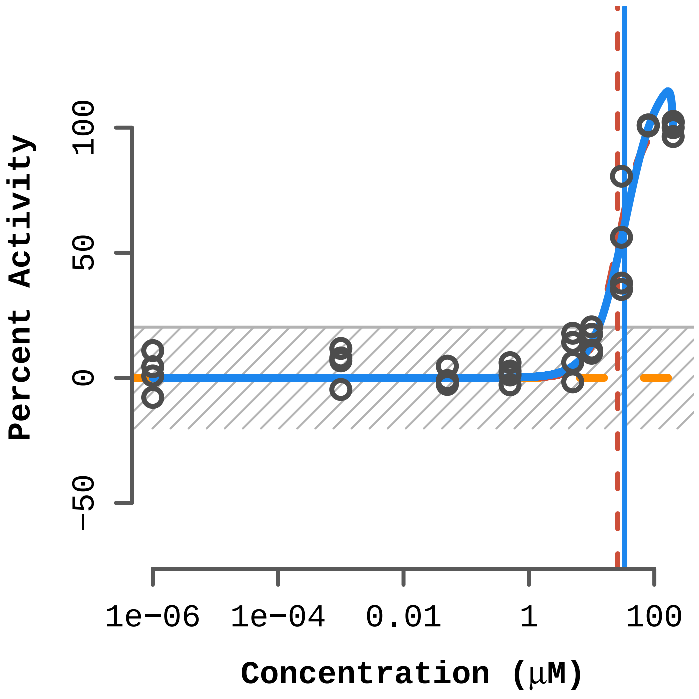

ASSAY: AEID3094 (CCTE_Deisenroth_DEVTOX_RUES2-GLR_Sox:

NAME: Esomeprazole magnesium hydrate
CHID: 746660 CASRN: 668985-31-7
SPID(S): ES211_43_43
M4ID: 42675951

HILL MODEL (in red):

	tp	ga	gw
val:	106	1.42	2.03
sd:	3.52	0.0506	0.271

GAIN-LOSS MODEL (in blue):

	tp	ga	gw	la	lw
val:	123	1.53	1.69	2.34	18
sd:	12.6	0.0846	0.251	0.00572	5.21

	CNST	HILL	GNLS
AIC:	384.57	262.87	258.75
PROB:	0	0.11	0.89
RMSE:	51.46	8.7	8.01

MAX_MEAN: 101 MAX_MED: 101 BMAD: 6.75

COFF: 20.3 HIT-CALL: 1 FITC: 50 ACTP: 1

FLAGS:

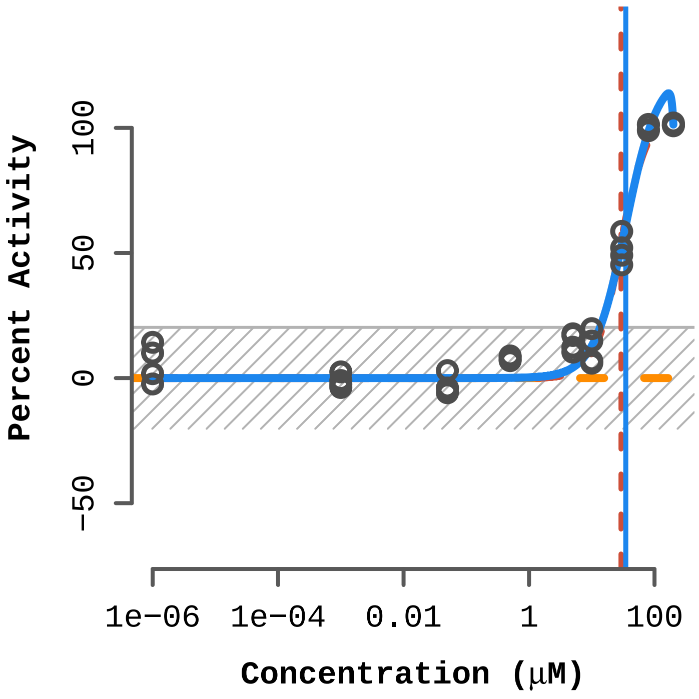

ASSAY: AEID3094 (CCTE_Deisenroth_DEVTOX_RUES2-GLR_Sox:

NAME: Mianserin hydrochloride
CHID: 944145 CASRN: 21535-47-7
SPID(S): ES211_47_47
M4ID: 42675904

HILL MODEL (in red):

	tp	ga	gw
val:	106	1.47	2.09
sd:	3.5	0.0292	0.385

GAIN-LOSS MODEL (in blue):

	tp	ga	gw	la	lw
val:	122	1.54	1.71	2.35	17.9
sd:	11.8	0.063	0.303	0.00557	4.92

	CNST	HILL	GNLS
AIC:	384.33	252.43	247.4
PROB:	0	0.07	0.93
RMSE:	51.11	6.88	6.18

MAX_MEAN: 101 MAX_MED: 101 BMAD: 6.75

COFF: 20.3 HIT-CALL: 1 FITC: 50 ACTP: 1

FLAGS:

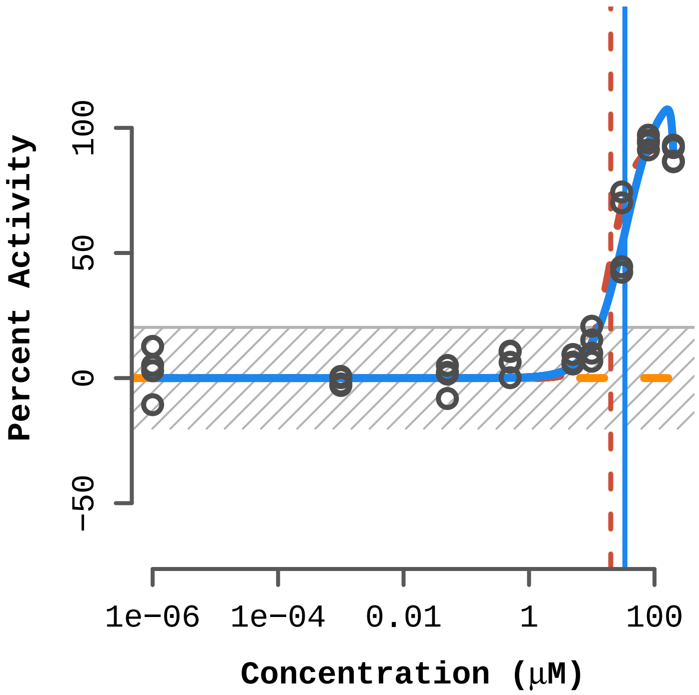

ASSAY: AEID3094 (CCTE_Deisenroth_DEVTOX_RUES2-GLR_Sox

NAME: Retinol
CHID: 23556 CASRN: 68-26-8
SPID(S): ES211_51_51
M4ID: 42675927

HILL MODEL (in red):

	tp	ga	gw
val:	93.5	1.3	2.46
sd:	2.52	0.0375	0.327

GAIN-LOSS MODEL (in blue):

	tp	ga	gw	la	lw
val:	117	1.53	1.64	2.34	15.7
sd:	29.8	0.244	0.46	0.00608	9.54

	CNST	HILL	GNLS
AIC:	380.89	254.68	253.07
PROB:	0	0.31	0.69
RMSE:	48.22	8.03	7.14

MAX_MEAN: 94.3 MAX_MED: 94.5 BMAD: 6.75

COFF: 20.3 HIT-CALL: 1 FITC: 51 ACTP: 1

FLAGS:
