# Supplementary material for: The DevTox Germ Layer Reporter Platform: An Assay Adaptation of the Human Pluripotent Stem Cell Test
Source: Toxics. 2022 Jul 13;10(7):392. doi: 10.3390/toxics10070392 (PMC9321663; doi:10.3390/toxics10070392)
Supplement: Supplementary file 1 [file toxics-10-00392-s001.zip › AEID3095_L6_CCTE_Deisenroth_DEVTOX_RUES2-GLR_Sox2_up_210917.pdf]

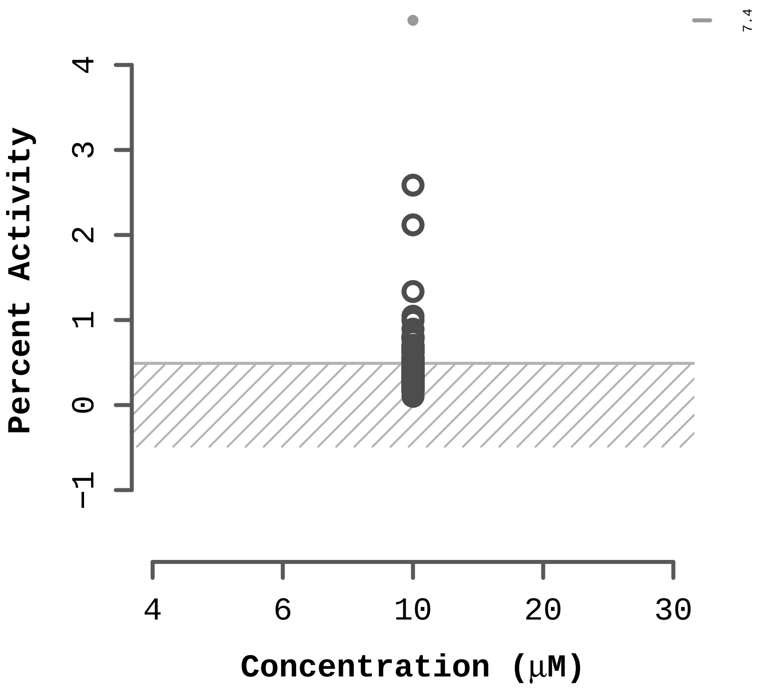

ASSAY: AEID3095 (CCTE_Deisenroth_DEVTOX_RUES2-GLR_Sox2

NAME: SB-431542
CHID: 42693 CASRN: 301836-41-9
SPID(S): ES211_56_56
M4ID: 42675976 BRK

HILL MODEL: Not applicable.

GAIN-LOSS MODEL: Not applicable.

	CNST	HILL	GNLS
AIC:	NA	NA	NA
PROB:	NA	NA	NA
RMSE:	NA	NA	NA

MAX_MEAN: 0.547 MAX_MED: 0.394 BMAD: 0.164

COFF: 0.491 HIT-CALL: -1 FITC: 2 ACTP: NA

FLAGS:

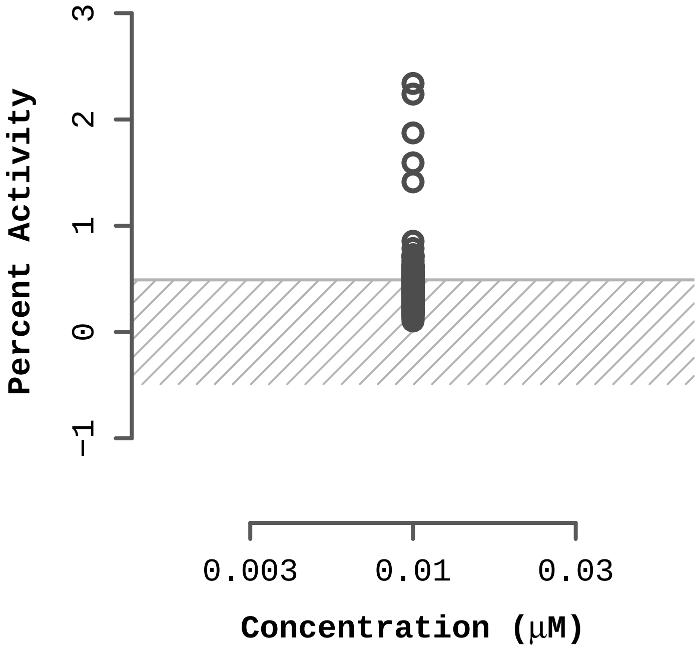

ASSAY: AEID3095 (CCTE_Deisenroth_DEVTOX_RUES2-GLR_Sox2

NAME: DMSO (spid not in DB)
CHID: NA CASRN: NA
SPID(S): DMSO
M4ID: 42675979

HILL MODEL: Not applicable.

GAIN−LOSS MODEL: Not applicable.

	CNST	HILL	GNLS
AIC:	NA	NA	NA
PROB:	NA	NA	NA
RMSE:	NA	NA	NA

MAX_MEAN: 0.432 MAX_MED: 0.387 BMAD: 0.164

COFF: 0.491 HIT−CALL: −1 FITC: 2 ACTP: NA

FLAGS:

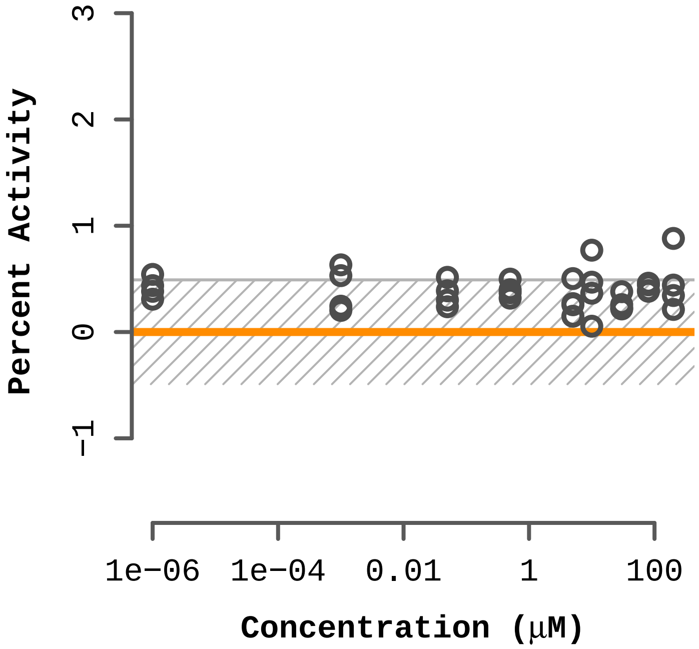

ASSAY: AEID3095 (CCTE_Deisenroth_DEVTOX_RUES2-GLR_Sox2

NAME: Acrylamide
CHID: 20027 CASRN: 79-06-1
SPID(S): ES211_10_10
M4ID: 42676034

HILL MODEL: Not applicable.

GAIN-LOSS MODEL: Not applicable.

	CNST	HILL	GNLS
AIC:	47.06	NA	NA
PROB:	1	NA	NA
RMSE:	0.42	NA	NA

MAX_MEAN: 0.47 MAX_MED: 0.417 BMAD: 0.164

COFF: 0.491 HIT-CALL: 0 FITC: 4 ACTP: 0

FLAGS:

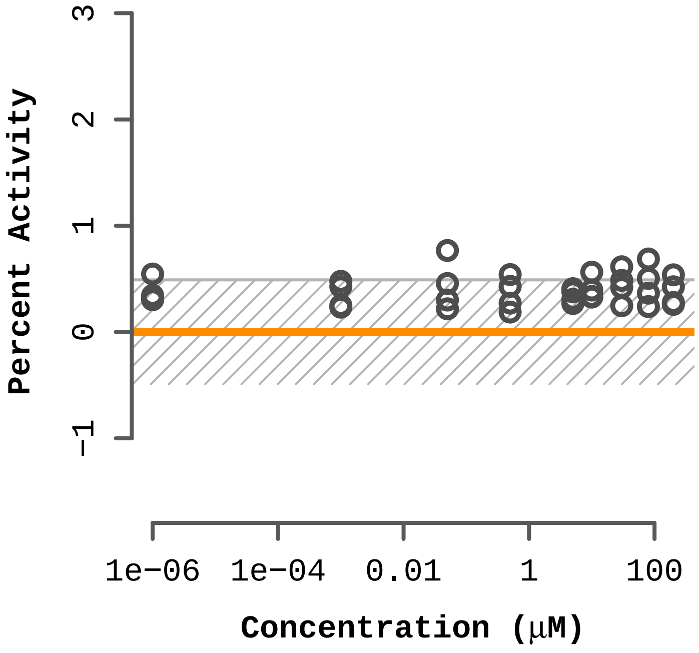

ASSAY: AEID3095 (CCTE_Deisenroth_DEVTOX_RUES2-GLR_Sox2

NAME: Boric acid (H3BO3)
CHID: 20194 CASRN: 10043-35-3
SPID(S): ES211_17_17
M4ID: 42676001

HILL MODEL: Not applicable.

GAIN-LOSS MODEL: Not applicable.

	CNST	HILL	GNLS
AIC:	47.77	NA	NA
PROB:	1	NA	NA
RMSE:	0.42	NA	NA

MAX_MEAN: 0.45 MAX_MED: 0.453 BMAD: 0.164

COFF: 0.491 HIT-CALL: 0 FITC: 4 ACTP: 0

FLAGS:

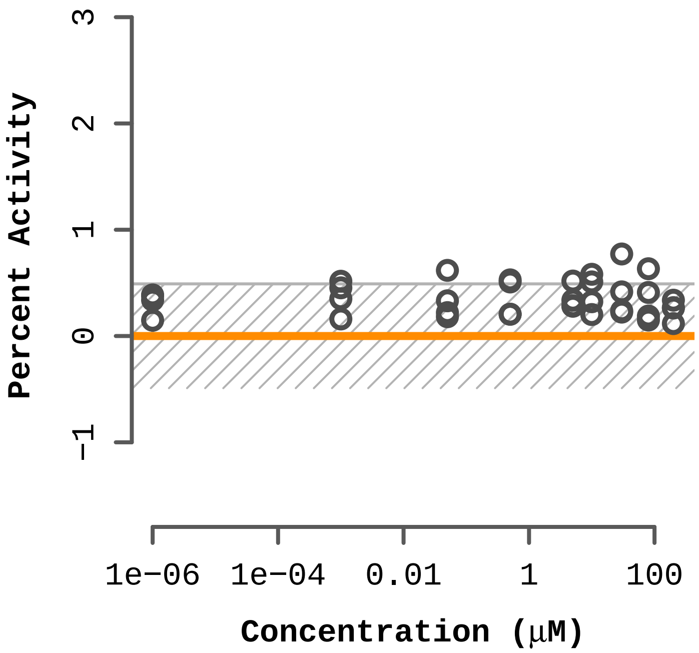

ASSAY: AEID3095 (CCTE_Deisenroth_DEVTOX_RUES2-GLR_Sox2

NAME: Caffeine
CHID: 20232 CASRN: 58−08−2
SPID(S): ES211_20_20
M4ID: 42676002

HILL MODEL: Not applicable.

GAIN−LOSS MODEL: Not applicable.

	CNST	HILL	GNLS
AIC:	41.07	NA	NA
PROB:	1	NA	NA
RMSE:	0.38	NA	NA

MAX_MEAN: 0.413 MAX_MED: 0.416 BMAD: 0.164

COFF: 0.491 HIT−CALL: 0 FITC: 4 ACTP: 0

FLAGS:

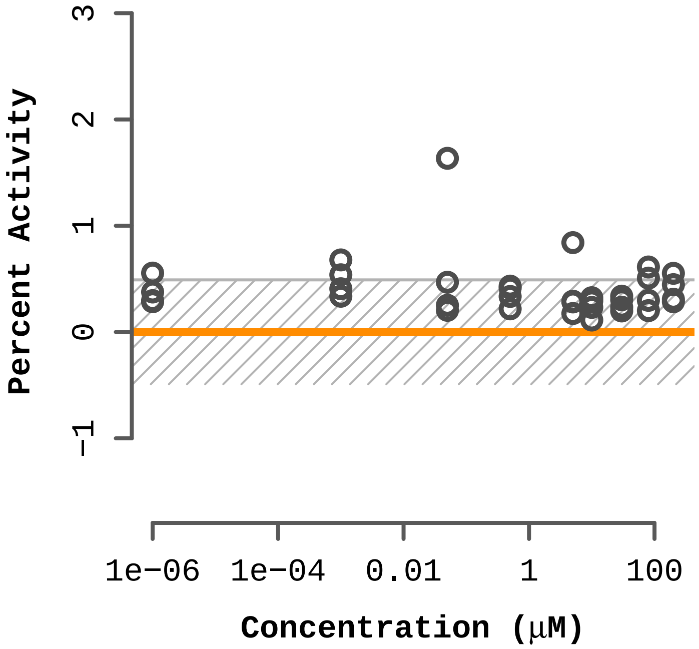

ASSAY: AEID3095 (CCTE_Deisenroth_DEVTOX_RUES2-GLR_Sox2

NAME: Thalidomide
CHID: 22524 CASRN: 50-35-1
SPID(S): ES211_62_62
M4ID: 42676014

HILL MODEL: Not applicable.

GAIN-LOSS MODEL: Not applicable.

	CNST	HILL	GNLS
AIC:	50.29	NA	NA
PROB:	1	NA	NA
RMSE:	0.47	NA	NA

MAX_MEAN: 0.64 MAX_MED: 0.473 BMAD: 0.164

COFF: 0.491 HIT-CALL: 0 FITC: 4 ACTP: 0

FLAGS:

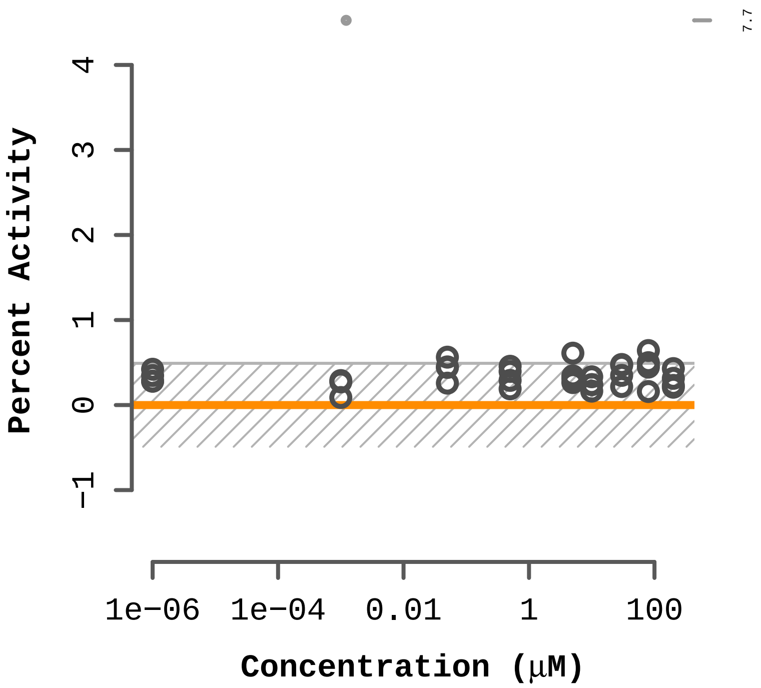

ASSAY: AEID3095 (CCTE_Deisenroth_DEVTOX_RUES2-GLR_Sox2

NAME: Acyclovir
CHID: 22556 CASRN: 59277-89-3
SPID(S): ES211_12_12
M4ID: 42675982 BRK

HILL MODEL: Not applicable.

GAIN-LOSS MODEL: Not applicable.

	CNST	HILL	GNLS
AIC:	61.14	NA	NA
PROB:	1	NA	NA
RMSE:	1.33	NA	NA

MAX_MEAN: 2.09 MAX_MED: 0.473 BMAD: 0.164

COFF: 0.491 HIT-CALL: 0 FITC: 4 ACTP: 0

FLAGS: 10

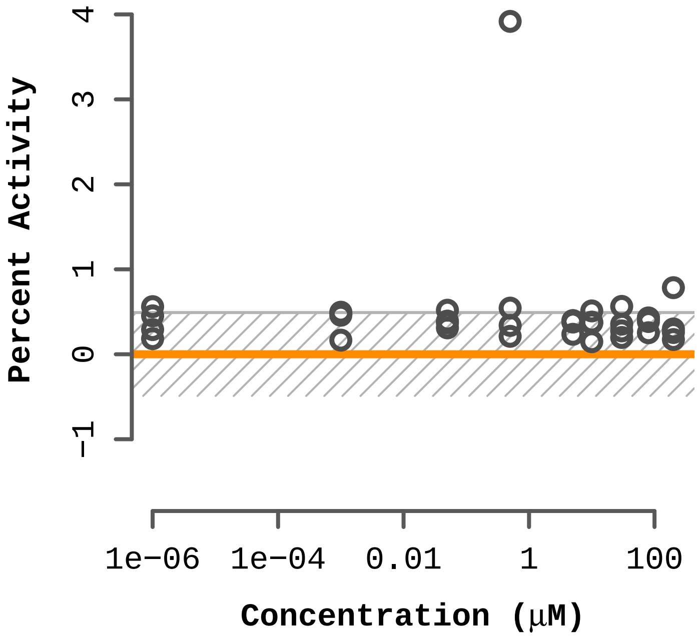

ASSAY: AEID3095 (CCTE_Deisenroth_DEVTOX_RUES2-GLR_Sox2

NAME: Valproic acid
CHID: 23733 CASRN: 99-66-1
SPID(S): ES211_63_63
M4ID: 42676018

HILL MODEL: Not applicable.

GAIN-LOSS MODEL: Not applicable.

	CNST	HILL	GNLS
AIC:	59.36	NA	NA
PROB:	1	NA	NA
RMSE:	0.76	NA	NA

MAX_MEAN: 1.25 MAX_MED: 0.475 BMAD: 0.164

COFF: 0.491 HIT-CALL: 0 FITC: 4 ACTP: 0

FLAGS: 10

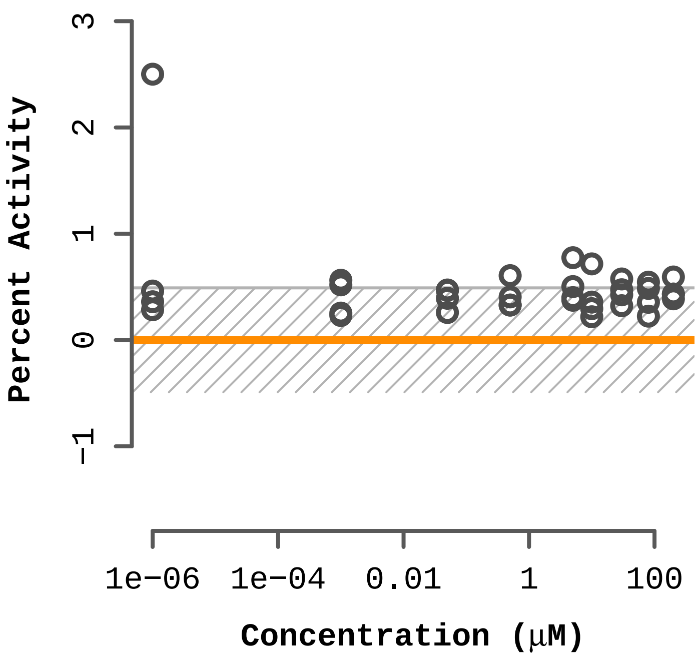

ASSAY: AEID3095 (CCTE_Deisenroth_DEVTOX_RUES2-GLR_Sox[illegible]

NAME: D-Camphor
CHID: 24721 CASRN: 464-49-3
SPID(S): ES211_27_27
M4ID: 42676032

HILL MODEL: Not applicable.

GAIN-LOSS MODEL: Not applicable.

	CNST	HILL	GNLS
AIC:	62.84	NA	NA
PROB:	1	NA	NA
RMSE:	0.6	NA	NA

MAX_MEAN: 0.902 MAX_MED: 0.451 BMAD: 0.164

COFF: 0.491 HIT-CALL: 0 FITC: 4 ACTP: 0

FLAGS: 10

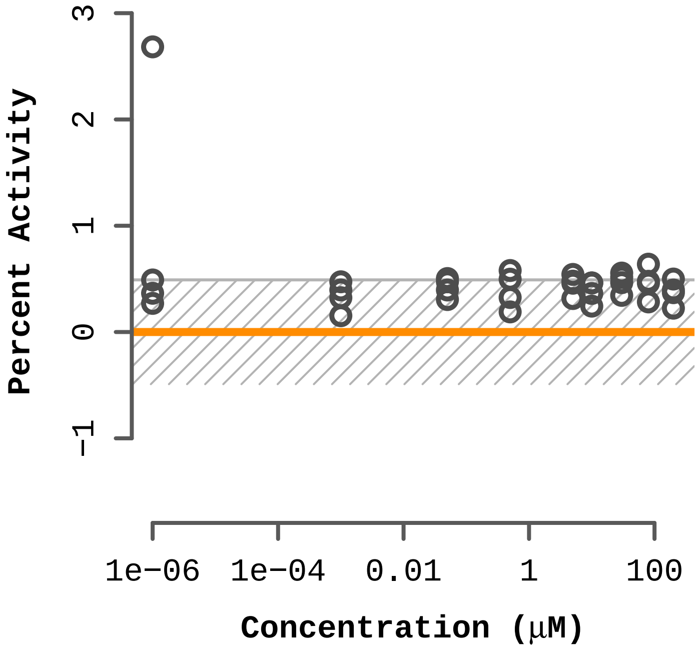

ASSAY: AEID3095 (CCTE_Deisenroth_DEVTOX_RUES2-GLR_Sox[illegible]

NAME: Cyclophosphamide monohydrate
CHID: 24888 CASRN: 6055−19−2
SPID(S): ES211_24_24
M4ID: 42675991

HILL MODEL: Not applicable.

GAIN−LOSS MODEL: Not applicable.

	CNST	HILL	GNLS
AIC:	60.94	NA	NA
PROB:	1	NA	NA
RMSE:	0.61	NA	NA

MAX_MEAN: 0.952 MAX_MED: 0.491 BMAD: 0.164

COFF: 0.491 HIT−CALL: 0 FITC: 4 ACTP: 0

FLAGS: 10

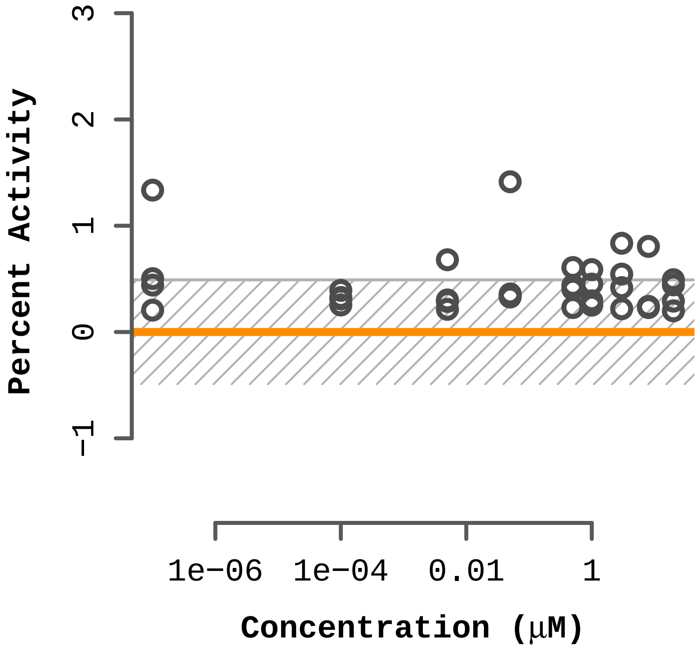

ASSAY: AEID3095 (CCTE_Deisenroth_DEVTOX_RUES2-GLR_Sox2

NAME: Dexamethasone sodium phosphate
CHID: 47429 CASRN: 2392-39-4
SPID(S): ES211_28_28
M4ID: 42675983

HILL MODEL: Not applicable.

GAIN-LOSS MODEL: Not applicable.

	CNST	HILL	GNLS
AIC:	58.18	NA	NA
PROB:	1	NA	NA
RMSE:	0.53	NA	NA

MAX_MEAN: 0.622 MAX_MED: 0.482 BMAD: 0.164

COFF: 0.491 HIT-CALL: 0 FITC: 4 ACTP: 0

FLAGS: 10

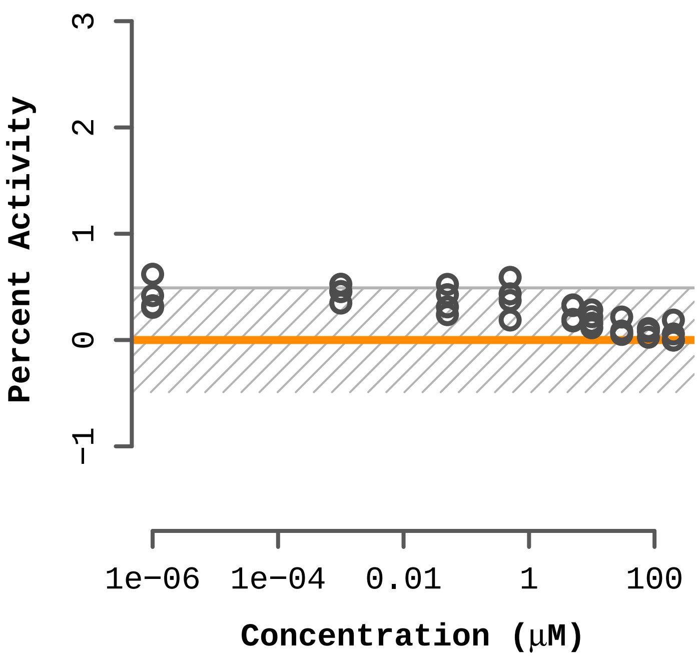

ASSAY: AEID3095 (CCTE_Deisenroth_DEVTOX_RUES2-GLR_Sox2

NAME: (2R,3S)-2-(3,4-Dihydroxyphenyl)-3,4-dihydro-2H-
CHID: 1017388 CASRN: 225937-10-0
SPID(S): ES211_22_22
M4ID: 42676008

HILL MODEL: Not applicable.

GAIN-LOSS MODEL: Not applicable.

	CNST	HILL	GNLS
AIC:	24.33	NA	NA
PROB:	1	NA	NA
RMSE:	0.31	NA	NA

MAX_MEAN: 0.447 MAX_MED: 0.456 BMAD: 0.164

COFF: 0.491 HIT-CALL: 0 FITC: 4 ACTP: 0

FLAGS:

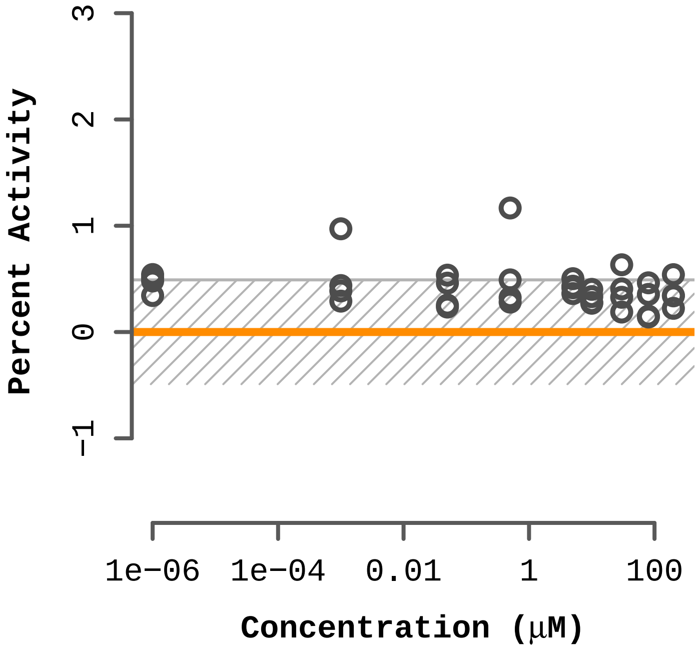

ASSAY: AEID3095 (CCTE_Deisenroth_DEVTOX_RUES2-GLR_Sox2

NAME: Warfarin
CHID: 23742 CASRN: 81−81−2
SPID(S): ES211_65_65
M4ID: 42675989

HILL MODEL: Failed to converge.

GAIN−LOSS MODEL: Failed to converge.

	CNST	HILL	GNLS
AIC:	52.25	NA	NA
PROB:	1	NA	NA
RMSE:	0.46	NA	NA

MAX_MEAN: 0.566 MAX_MED: 0.495 BMAD: 0.164

COFF: 0.491 HIT−CALL: 0 FITC: 7 ACTP: 0

FLAGS:

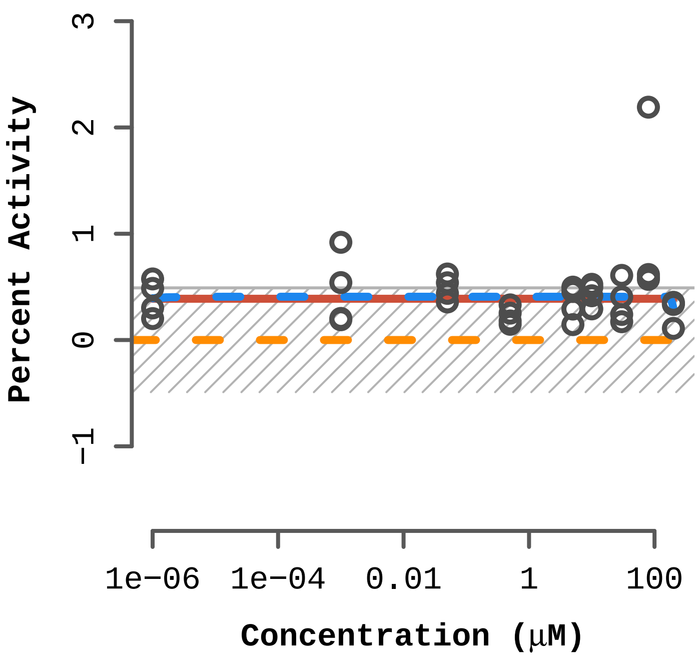

ASSAY: AEID3095 (CCTE_Deisenroth_DEVTOX_RUES2-GLR_Sox2

NAME: Acetaminophen
CHID: 20006 CASRN: 103-90-2
SPID(S): ES211_9_9
M4ID: 42676004

HILL MODEL (in red):

	tp	ga	gw
val:	0.388	-6.87	7.68
sd:	NaN	NaN	NaN

GAIN-LOSS MODEL (in blue):

	tp	ga	gw	la	lw
val:	0.407	-6.85	1.67	2.33	16.4
sd:	0.0388	129	254	0.0333	21.4

	CNST	HILL	GNLS
AIC:	58.94	5.06	7.57
PROB:	0	0.78	0.22
RMSE:	0.56	0.35	0.34

MAX_MEAN: 0.992 MAX_MED: 0.603 BMAD: 0.164

COFF: 0.491 HIT-CALL: 0 FITC: 15 ACTP: 1

FLAGS: 12

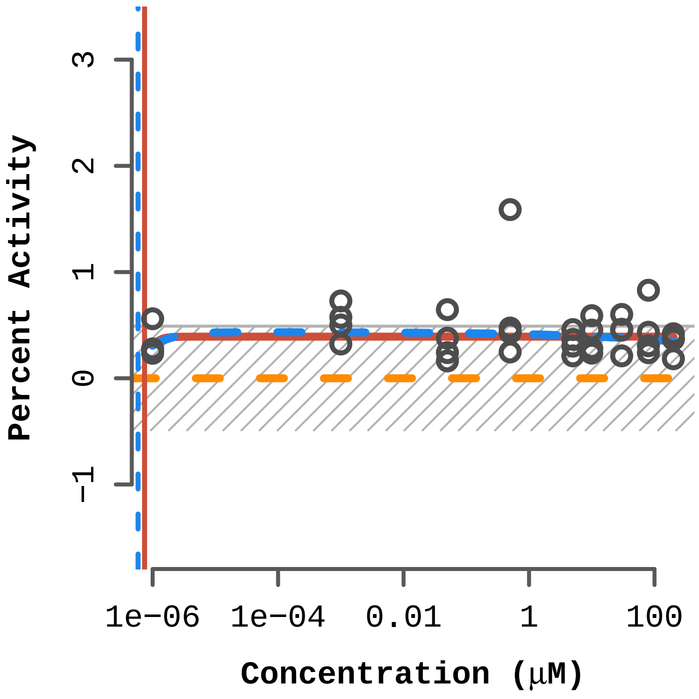

ASSAY: AEID3095 (CCTE_Deisenroth_DEVTOX_RUES2-GLR_Sox[illegible]

NAME: Isoniazid
CHID: 20755 CASRN: 54-85-3
SPID(S): ES211_39_39
M4ID: 42675985

HILL MODEL (in red):

	tp	ga	gw
val:	0.392	-6.13	4.36
sd:	0.0302	0.718	24.7

GAIN-LOSS MODEL (in blue):

	tp	ga	gw	la	lw
val:	0.435	-6.23	1.64	4.29	0.3
sd:	NaN	NaN	NaN	NaN	NaN

	CNST	HILL	GNLS
AIC:	55.7	-7.19	-3.96
PROB:	0	0.83	0.17
RMSE:	0.5	0.25	0.25

MAX_MEAN: 0.685 MAX_MED: 0.538 BMAD: 0.164

COFF: 0.491 HIT-CALL: 0 FITC: 15 ACTP: 1

FLAGS: 12

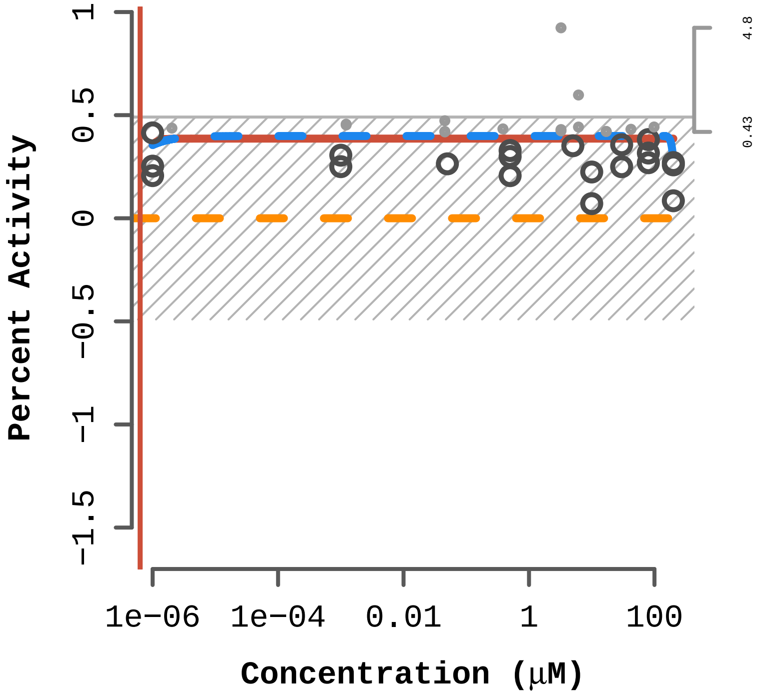

ASSAY: AEID3095 (CCTE_Deisenroth_DEVTOX_RUES2-GLR_Sox:

NAME: Stavudine
CHID: 23819 CASRN: 3056-17-5
SPID(S): ES211_58_58
M4ID: 42676038 BRK

HILL MODEL (in red):

	tp	ga	gw
val:	0.386	-6.2	5.33
sd:	NaN	NaN	NaN

GAIN-LOSS MODEL (in blue):

	tp	ga	gw	la	lw
val:	0.399	-6.56	1.62	2.32	17.8
sd:	0.0401	24.1	69.7	0.0326	28.7

	CNST	HILL	GNLS
AIC:	74.35	29.3	32.22
PROB:	0	0.81	0.19
RMSE:	0.97	0.8	0.8

MAX_MEAN: 1.54 MAX_MED: 0.512 BMAD: 0.164

COFF: 0.491 HIT-CALL: 0 FITC: 15 ACTP: 1

FLAGS: 8; 10; 12

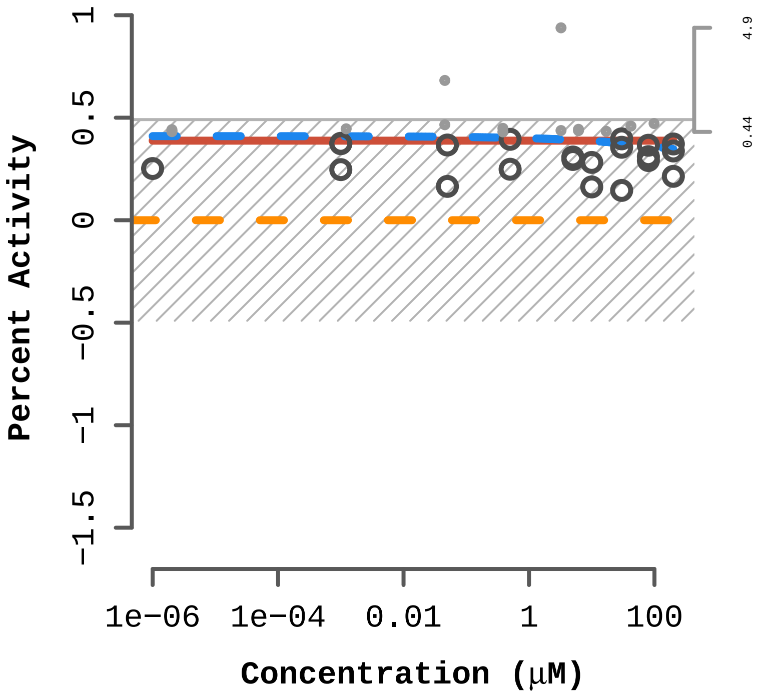

ASSAY: AEID3095 (CCTE_Deisenroth_DEVTOX_RUES2-GLR_Sox[illegible]

NAME: Hydroxyurea
CHID: 25438 CASRN: 127-07-1
SPID(S): ES211_35_35
M4ID: 42676007 BRK

HILL MODEL (in red):

	tp	ga	gw
val:	0.388	-6.95	7.58
sd:	0.0301	226	1460

GAIN-LOSS MODEL (in blue):

	tp	ga	gw	la	lw
val:	0.41	-6.83	7.38	4.3	0.368
sd:	0.0615	100	722	4.35	0.859

	CNST	HILL	GNLS
AIC:	76.54	24.26	27.9
PROB:	0	0.86	0.14
RMSE:	1.02	0.86	0.85

MAX_MEAN: 1.5 MAX_MED: 0.557 BMAD: 0.164

COFF: 0.491 HIT-CALL: 0 FITC: 15 ACTP: 1

FLAGS: 10; 12

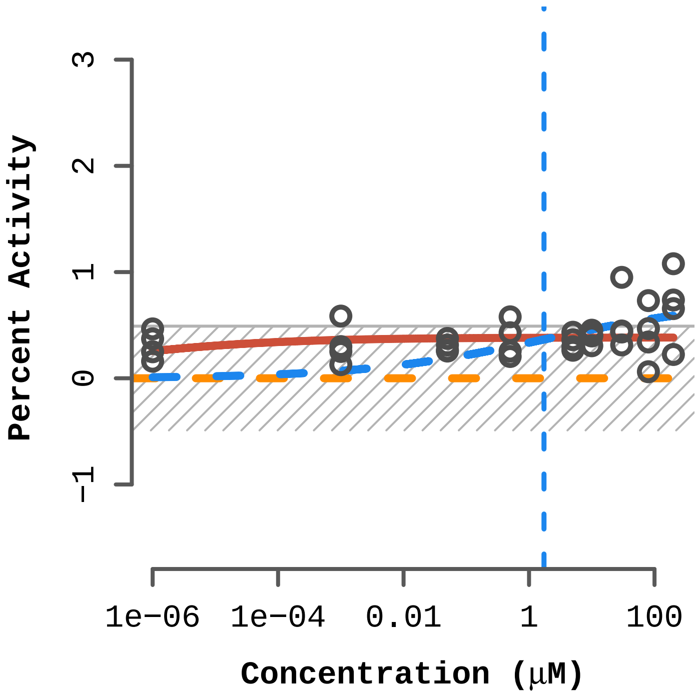

ASSAY: AEID3095 (CCTE_Deisenroth_DEVTOX_RUES2-GLR_Sox2

NAME: Lenalidomide
CHID: 46664 CASRN: 191732-72-6
SPID(S): ES211_41_41
M4ID: 42675993

HILL MODEL (in red):

	tp	ga	gw
val:	0.384	-7	0.3
sd:	NaN	NaN	NaN

GAIN-LOSS MODEL (in blue):

	tp	ga	gw	la	lw
val:	0.731	0.238	0.3	4.3	9.63
sd:	NA	NA	NA	NA	NA

	CNST	HILL	GNLS
AIC:	52.1	-7	10.87
PROB:	0	1	0
RMSE:	0.46	0.21	0.23

MAX_MEAN: 0.674 MAX_MED: 0.697 BMAD: 0.164

COFF: 0.491 HIT-CALL: 0 FITC: 16 ACTP: 1

FLAGS: 12

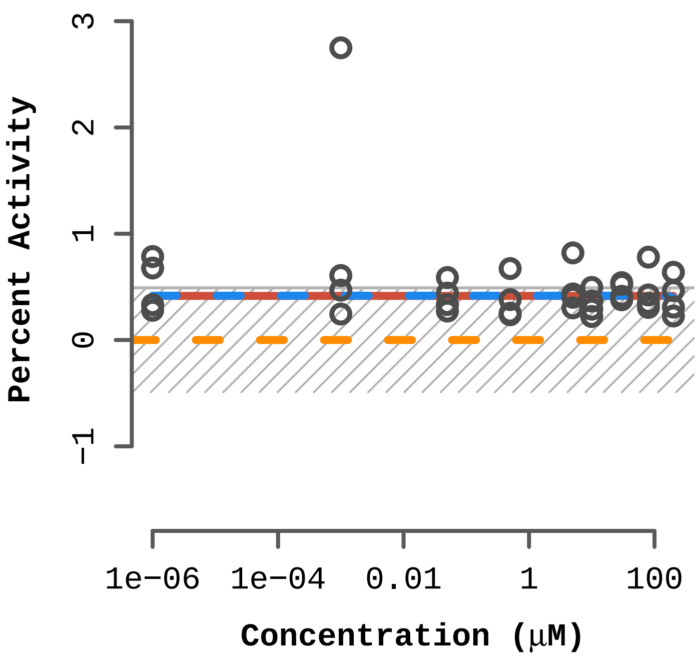

ASSAY: AEID3095 (CCTE_Deisenroth_DEVTOX_RUES2-GLR_Sox2

NAME: Ethylene glycol
CHID: 20597 CASRN: 107-21-1
SPID(S): ES211_32_32
M4ID: 42676026

HILL MODEL (in red):

	tp	ga	gw
val:	0.415	-7	6.97
sd:	0.0306	233	1510

GAIN-LOSS MODEL (in blue):

	tp	ga	gw	la	lw
val:	0.417	-6.97	7.95	2.64	3.94
sd:	0.0328	412	2140	6.09	71

	CNST	HILL	GNLS
AIC:	66.31	3.47	7.44
PROB:	0	0.88	0.12
RMSE:	0.65	0.42	0.42

MAX_MEAN: 1.02 MAX_MED: 0.539 BMAD: 0.164

COFF: 0.491 HIT-CALL: 0 FITC: 20 ACTP: 1

FLAGS: 8; 12

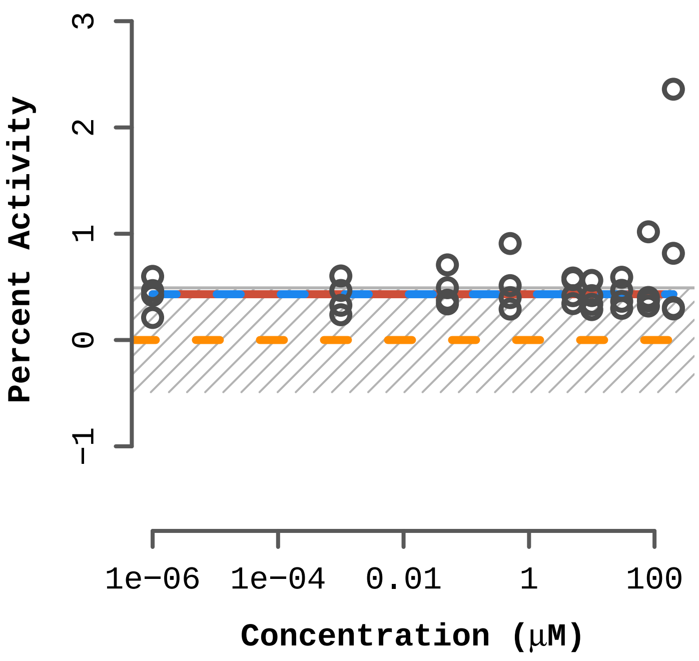

ASSAY: AEID3095 (CCTE_Deisenroth_DEVTOX_RUES2-GLR_Sox2

NAME: Ibuprofen
CHID: 20732 CASRN: 15687-27-1
SPID(S): ES211_36_36
M4ID: 42676025

HILL MODEL (in red):

	tp	ga	gw
val:	0.431	-7	6.07
sd:	0.0291	290	1690

GAIN-LOSS MODEL (in blue):

	tp	ga	gw	la	lw
val:	0.431	-7	6.42	3.44	17.8
sd:	NA	NA	NA	NA	NA

	CNST	HILL	GNLS
AIC:	68.34	2.51	6.51
PROB:	0	0.88	0.12
RMSE:	0.63	0.37	0.37

MAX_MEAN: 0.944 MAX_MED: 0.561 BMAD: 0.164

COFF: 0.491 HIT-CALL: 0 FITC: 20 ACTP: 1

FLAGS: 8; 12

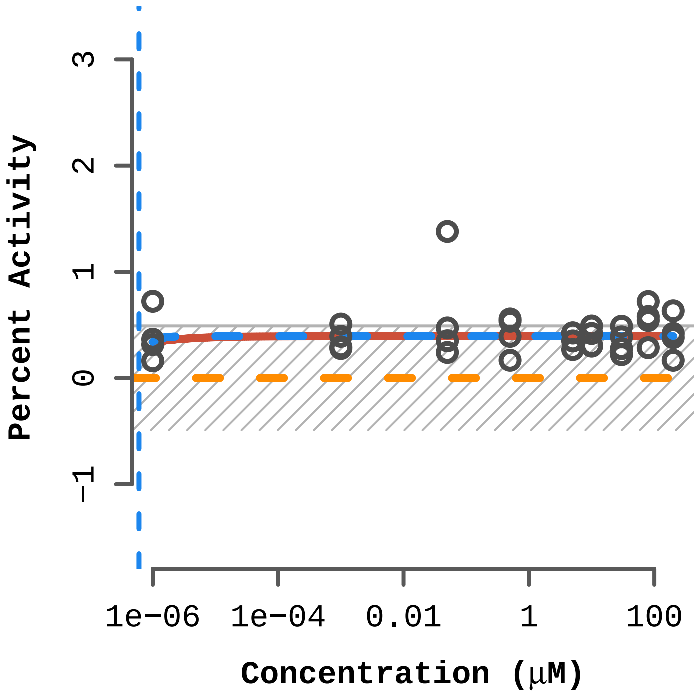

ASSAY: AEID3095 (CCTE_Deisenroth_DEVTOX_RUES2-GLR_Sox2

NAME: Nicotinic acid
CHID: 20932 CASRN: 59-67-6
SPID(S): ES211_48_48
M4ID: 42676024

HILL MODEL (in red):

	tp	ga	gw
val:	0.394	-7	0.793
sd:	NaN	NaN	NaN

GAIN-LOSS MODEL (in blue):

	tp	ga	gw	la	lw
val:	0.394	-6.22	3.65	3.8	4.69
sd:	NaN	NaN	NaN	NaN	NaN

	CNST	HILL	GNLS
AIC:	54.25	-15.04	-11.04
PROB:	0	0.88	0.12
RMSE:	0.47	0.22	0.22

MAX_MEAN: 0.611 MAX_MED: 0.561 BMAD: 0.164

COFF: 0.491 HIT-CALL: 0 FITC: 20 ACTP: 1

FLAGS: 12

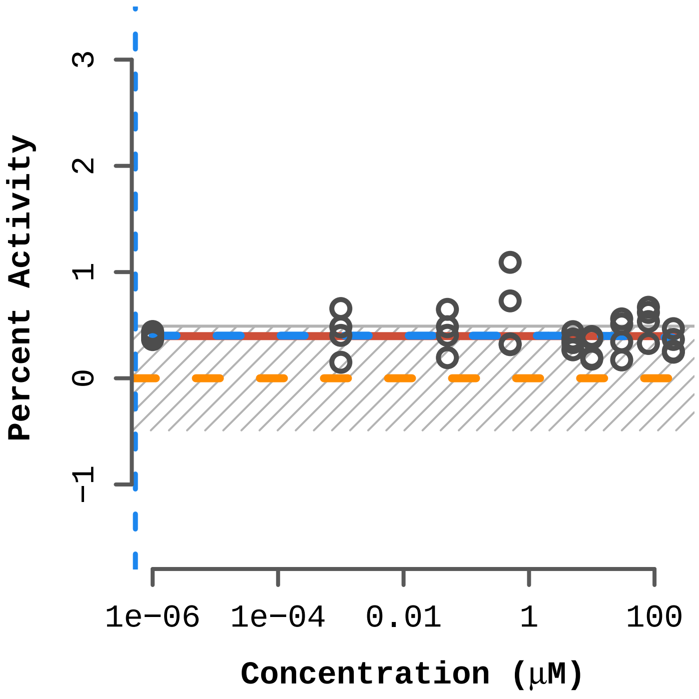

ASSAY: AEID3095 (CCTE_Deisenroth_DEVTOX_RUES2-GLR_Sox2

NAME: 1,2-Propylene glycol
CHID: 21206 CASRN: 57-55-6
SPID(S): ES211_1_1
M4ID: 42675977

HILL MODEL (in red):

	tp	ga	gw
val:	0.397	−7	6.18
sd:	0.0268	266	1560

GAIN-LOSS MODEL (in blue):

	tp	ga	gw	la	lw
val:	0.401	−6.27	8	3.74	0.866
sd:	NaN	NaN	NaN	NaN	NaN

	CNST	HILL	GNLS
AIC:	53.71	−15.24	−11.36
PROB:	0	0.87	0.13
RMSE:	0.46	0.19	0.19

MAX_MEAN: 0.616 MAX_MED: 0.577 BMAD: 0.164

COFF: 0.491 HIT-CALL: 0 FITC: 20 ACTP: 1

FLAGS: 8; 12

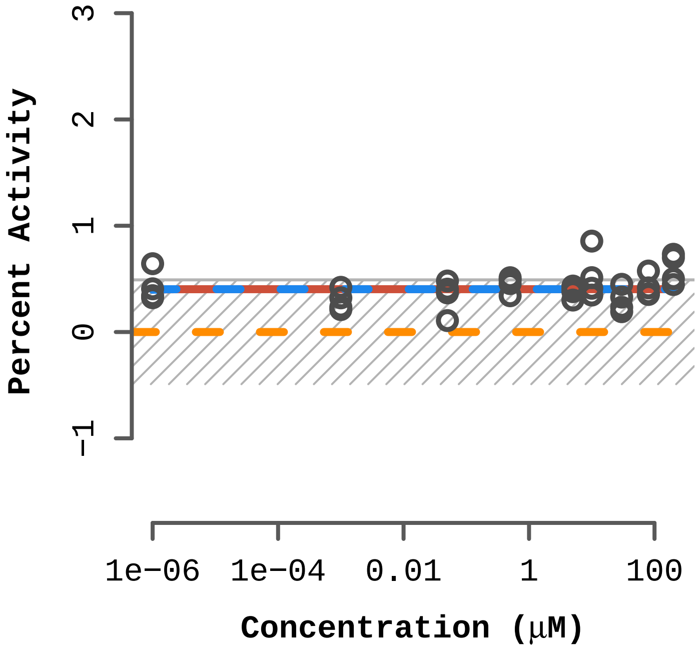

ASSAY: AEID3095 (CCTE_Deisenroth_DEVTOX_RUES2-GLR_Sox2

NAME: 6-Propyl-2-thiouracil
CHID: 21209 CASRN: 51-52-5
SPID(S): ES211_8_8
M4ID: 42675975

HILL MODEL (in red):

	tp	ga	gw
val:	0.403	-6.71	2.58
sd:	NaN	NaN	NaN

GAIN-LOSS MODEL (in blue):

	tp	ga	gw	la	lw
val:	0.403	-6.49	3.79	4.12	4.71
sd:	0.0227	3.89	31.2	1510	3900

	CNST	HILL	GNLS
AIC:	52.26	-29.16	-25.16
PROB:	0	0.88	0.12
RMSE:	0.44	0.15	0.15

MAX_MEAN: 0.595 MAX_MED: 0.601 BMAD: 0.164

COFF: 0.491 HIT-CALL: 0 FITC: 20 ACTP: 1

FLAGS: 12

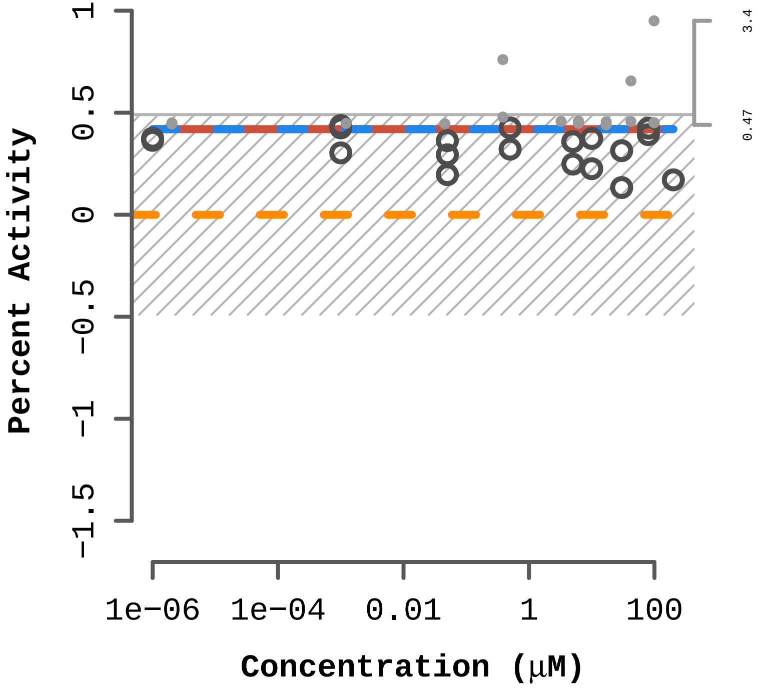

ASSAY: AEID3095 (CCTE_Deisenroth_DEVTOX_RUES2-GLR_Sox2

NAME: Dimethyl phthalate
CHID: 22455 CASRN: 131-11-3
SPID(S): ES211_30_30
M4ID: 42675997 BRK

HILL MODEL (in red):

	tp	ga	gw
val:	0.419	-7	6.71
sd:	0.0285	236	1500

GAIN-LOSS MODEL (in blue):

	tp	ga	gw	la	lw
val:	0.419	-7	6.79	4.1	4.22
sd:	0.0285	251	1600	1430	3320

	CNST	HILL	GNLS
AIC:	78.45	23.41	27.41
PROB:	0	0.88	0.12
RMSE:	0.84	0.63	0.63

MAX_MEAN: 1.15 MAX_MED: 0.556 BMAD: 0.164

COFF: 0.491 HIT-CALL: 0 FITC: 20 ACTP: 1

FLAGS: 8; 10; 12

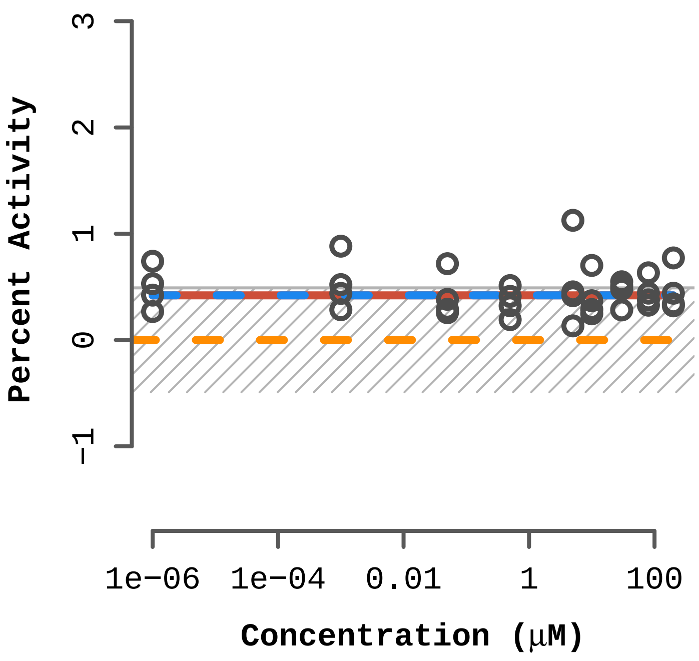

ASSAY: AEID3095 (CCTE_Deisenroth_DEVTOX_RUES2-GLR_Sox2

NAME: Salicylic acid
CHID: 26368 CASRN: 69-72-7
SPID(S): ES211_55_55
M4ID: 42676010

HILL MODEL (in red):

	tp	ga	gw
val:	0.42	-7	7.07
sd:	0.0297	223	1440

GAIN-LOSS MODEL (in blue):

	tp	ga	gw	la	lw
val:	0.42	-7	6.87	3.03	10.6
sd:	0.0297	197	1270	2600	37600

	CNST	HILL	GNLS
AIC:	59.59	-7.36	-3.36
PROB:	0	0.88	0.12
RMSE:	0.5	0.21	0.21

MAX_MEAN: 0.533 MAX_MED: 0.494 BMAD: 0.164

COFF: 0.491 HIT-CALL: 0 FITC: 20 ACTP: 1

FLAGS: 12

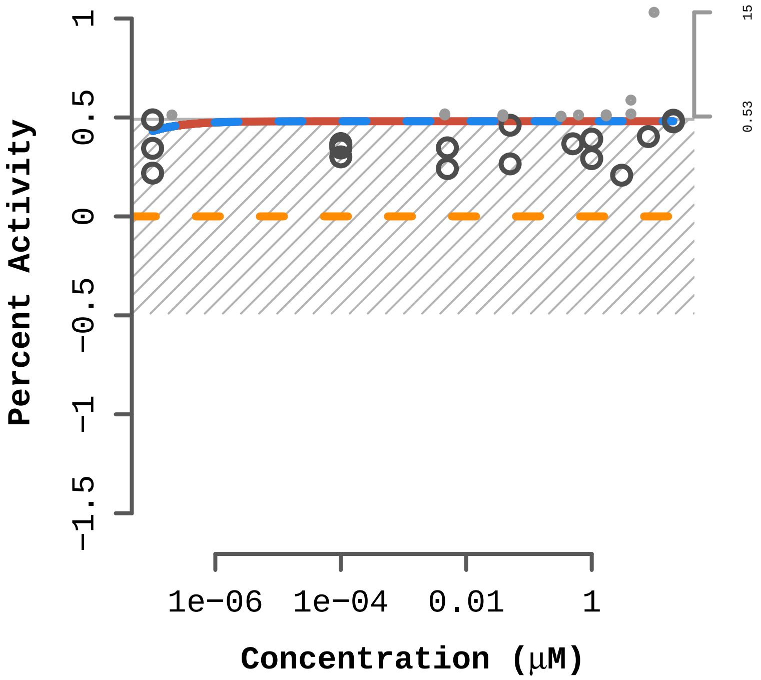

ASSAY: AEID3095 (CCTE_Deisenroth_DEVTOX_RUES2-GLR_Sox2

NAME: Cyclopamine
CHID: 43709 CASRN: 4449-51-8
SPID(S): ES211_23_23
M4ID: 42675992 BRK

HILL MODEL (in red):

	tp	ga	gw
val:	0.481	-8	0.939
sd:	NaN	NaN	NaN

GAIN-LOSS MODEL (in blue):

	tp	ga	gw	la	lw
val:	0.481	-8	0.942	3.29	18
sd:	NA	NA	NA	NA	NA

	CNST	HILL	GNLS
AIC:	90.06	50.95	54.95
PROB:	0	0.88	0.12
RMSE:	2.78	2.64	2.64

MAX_MEAN: 5.31 MAX_MED: 0.885 BMAD: 0.164

COFF: 0.491 HIT-CALL: 0 FITC: 20 ACTP: 1

FLAGS: 8; 10; 12

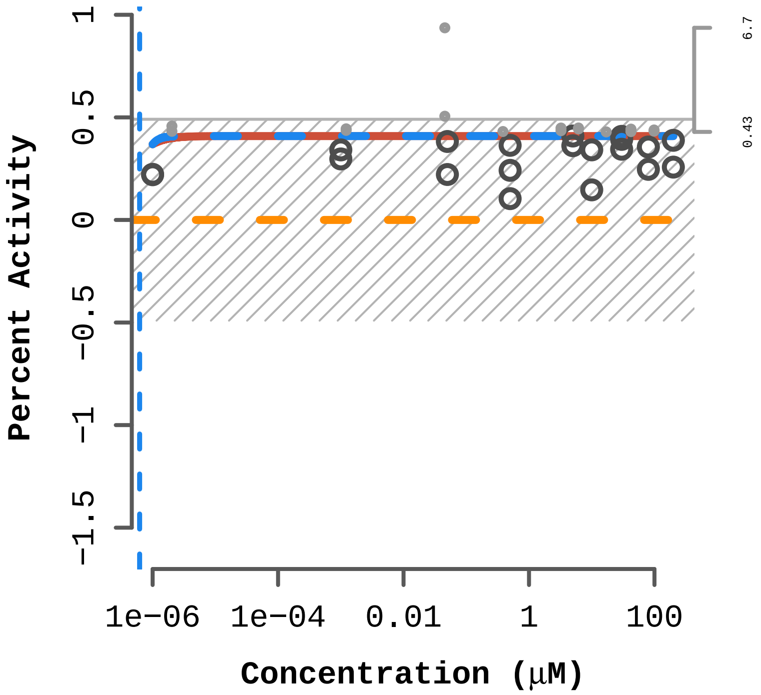

ASSAY: AEID3095 (CCTE_Deisenroth_DEVTOX_RUES2-GLR_Sox2

NAME: Penicillin G potassium
CHID: 45106 CASRN: 113-98-4
SPID(S): ES211_49_49
M4ID: 42675978 BRK

HILL MODEL (in red):

	tp	ga	gw
val:	0.409	-6.45	2.12
sd:	0.0318	14	65.5

GAIN-LOSS MODEL (in blue):

	tp	ga	gw	la	lw
val:	0.409	-6.21	4.65	3.86	4.65
sd:	NaN	NaN	NaN	NaN	NaN

	CNST	HILL	GNLS
AIC:	75.61	19.94	23.94
PROB:	0	0.88	0.12
RMSE:	1.22	1.07	1.07

MAX_MEAN: 2.17 MAX_MED: 0.88 BMAD: 0.164

COFF: 0.491 HIT-CALL: 0 FITC: 20 ACTP: 1

FLAGS: 10; 12

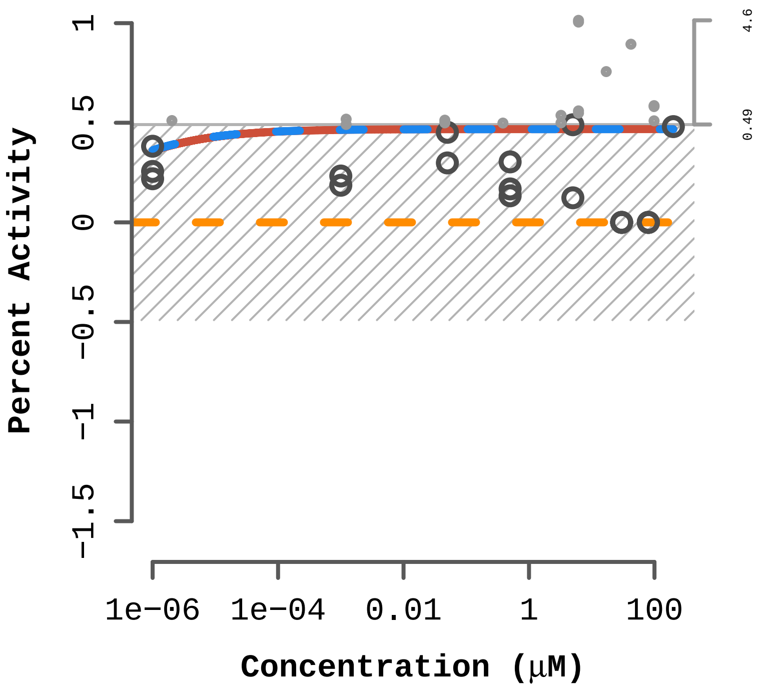

ASSAY: AEID3095 (CCTE_Deisenroth_DEVTOX_RUES2-GLR_Sox2

NAME: Tegaserod maleate
CHID: 904761 CASRN: 189188-57-6
SPID(S): ES211_61_61
M4ID: 42676033 BRK

HILL MODEL (in red):

	tp	ga	gw
val:	0.468	-7	0.521
sd:	NaN	NaN	NaN

GAIN-LOSS MODEL (in blue):

	tp	ga	gw	la	lw
val:	0.468	-7	0.532	3.16	17.9
sd:	NaN	NaN	NaN	NaN	NaN

	CNST	HILL	GNLS
AIC:	110.47	95.18	99.18
PROB:	0	0.88	0.12
RMSE:	1.41	1.21	1.21

MAX_MEAN: 2.78 MAX_MED: 2.79 BMAD: 0.164

COFF: 0.491 HIT-CALL: 0 FITC: 20 ACTP: 1

FLAGS: 8; 10; 12

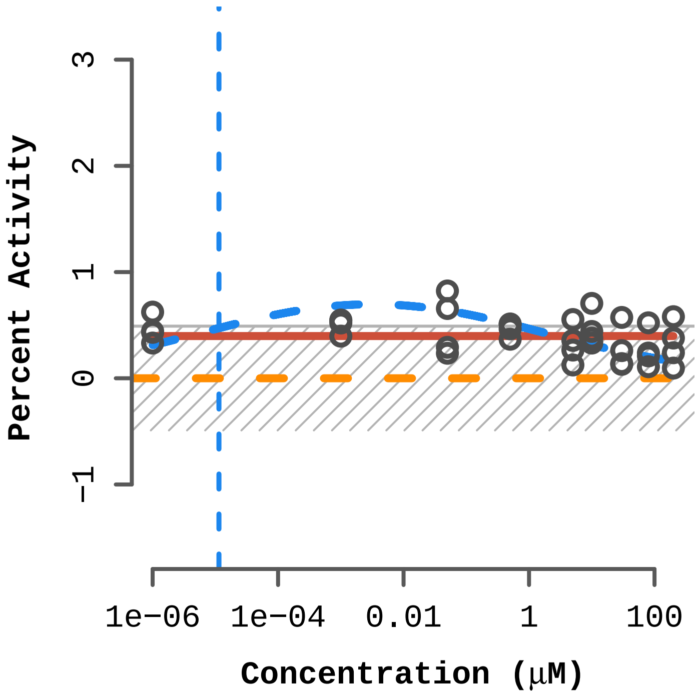

ASSAY: AEID3095 (CCTE_Deisenroth_DEVTOX_RUES2-GLR_Sox2

NAME: 5,5-Diphenylhydantoin
CHID: 20541 CASRN: 57-41-0
SPID(S): ES211_3_3
M4ID: 42676005

HILL MODEL (in red):

	tp	ga	gw
val:	0.397	-7	7.08
sd:	0.0316	207	1340

GAIN-LOSS MODEL (in blue):

	tp	ga	gw	la	lw
val:	0.977	-4.94	0.3	-0.0432	0.3
sd:	NaN	NaN	NaN	NaN	NaN

	CNST	HILL	GNLS
AIC:	50.79	-10.16	-1.85
PROB:	0	0.98	0.02
RMSE:	0.44	0.18	0.2

MAX_MEAN: 0.502 MAX_MED: 0.532 BMAD: 0.164

COFF: 0.491 HIT-CALL: 0 FITC: 21 ACTP: 1

FLAGS: 12

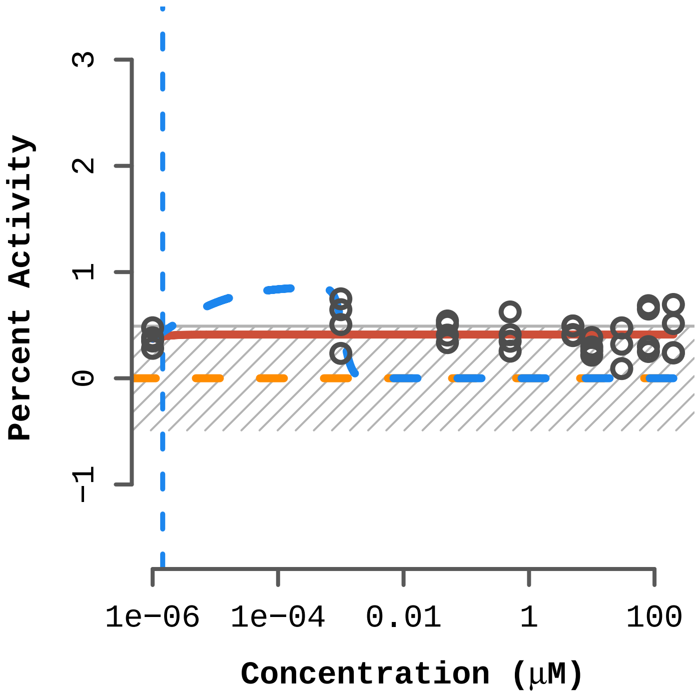

ASSAY: AEID3095 (CCTE_Deisenroth_DEVTOX_RUES2-GLR_Sox[illegible]

NAME: Carbamazepine
CHID: 22731 CASRN: 298-46-4
SPID(S): ES211_21_21
M4ID: 42676015

HILL MODEL (in red):

	tp	ga	gw
val:	0.412	-6.41	2.35
sd:	NaN	NaN	NaN

GAIN-LOSS MODEL (in blue):

	tp	ga	gw	la	lw
val:	0.869	-5.84	0.777	-2.97	6.58
sd:	NaN	NaN	NaN	NaN	NaN

	CNST	HILL	GNLS
AIC:	52.41	-21.11	51.36
PROB:	0	1	0
RMSE:	0.44	0.15	0.39

MAX_MEAN: 0.534 MAX_MED: 0.576 BMAD: 0.164

COFF: 0.491 HIT-CALL: 0 FITC: 21 ACTP: 1

FLAGS: 12

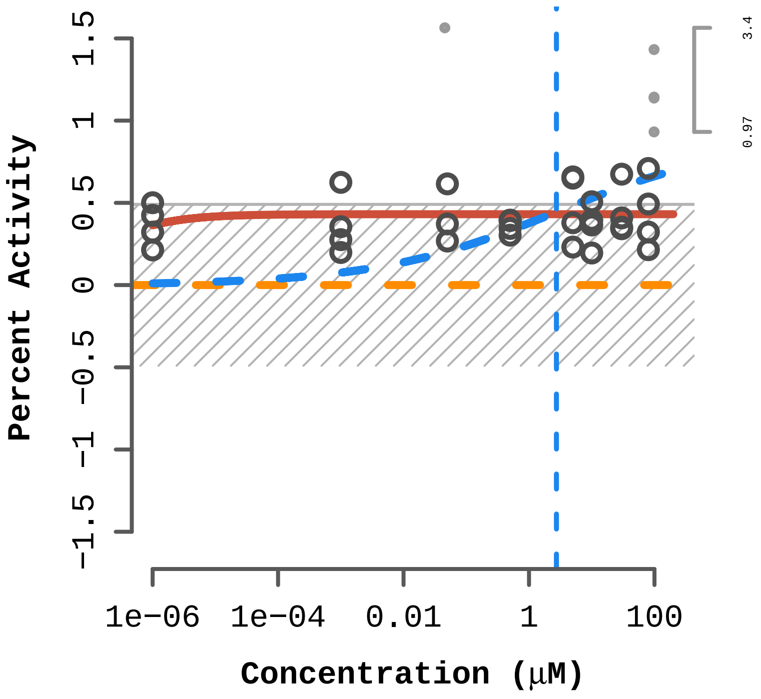

ASSAY: AEID3095 (CCTE_Deisenroth_DEVTOX_RUES2-GLR_Sox2

NAME: MEHP
CHID: 25680 CASRN: 4376-20-9
SPID(S): ES211_44_44
M4ID: 42676011 BRK

HILL MODEL (in red):

	tp	ga	gw
val:	0.431	-7	0.735
sd:	NaN	NaN	NaN

GAIN-LOSS MODEL (in blue):

	tp	ga	gw	la	lw
val:	0.887	0.437	0.3	4.3	5.17
sd:	NaN	NaN	NaN	NaN	NaN

	CNST	HILL	GNLS
AIC:	88.56	49.9	64.44
PROB:	0	1	0
RMSE:	0.95	0.73	0.73

MAX_MEAN: 1.85 MAX_MED: 1.77 BMAD: 0.164

COFF: 0.491 HIT-CALL: 0 FITC: 21 ACTP: 1

FLAGS: 8; 10; 12

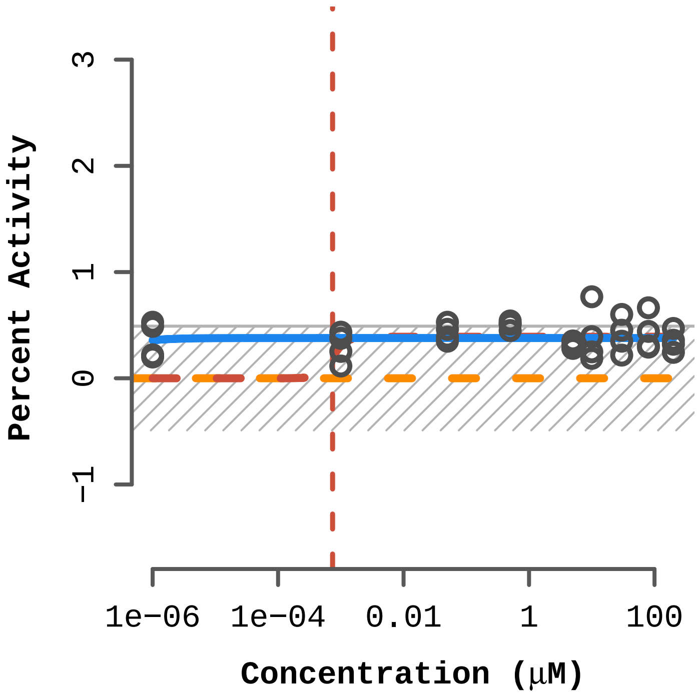


```
ASSAY:    AEID3095 (CCTE_Deisenroth_DEVTOX_RUES2-GLR_Sox2

NAME:     Aspirin
CHID:     20108   CASRN: 50-78-2
SPID(S):  ES211_15_15
M4ID:     42675995

HILL MODEL (in red):
      tp        ga        gw
val:  0.391     -3.13     4.08
sd:   NaN       NaN       NaN

GAIN-LOSS MODEL (in blue):
      tp        ga        gw        la        lw
val:  0.379     -7        1.25      4.14      7.43
sd:   NaN       NaN       NaN       NaN       NaN

      CNST        HILL        GNLS
AIC:  46.95       -14.87      -25.11
PROB: 0           0.01        0.99
RMSE: 0.41        0.18        0.14

MAX_MEAN: 0.503     MAX_MED: 0.512     BMAD: 0.164

COFF: 0.491  HIT-CALL: 0    FITC: 24   ACTP: 1

FLAGS:
```

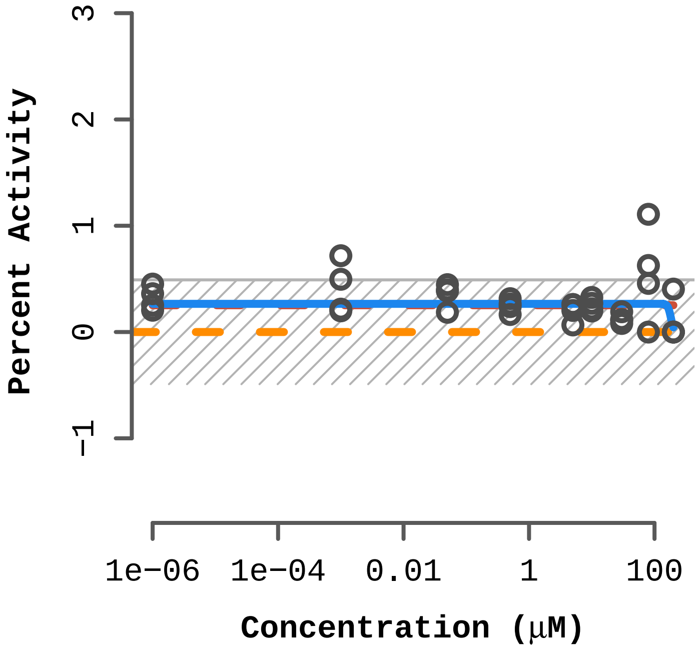

ASSAY: AEID3095 (CCTE_Deisenroth_DEVTOX_RUES2-GLR_Sox2

NAME: Rifampicin
CHID: 21244 CASRN: 13292-46-1
SPID(S): ES211_52_52
M4ID: 42676039

HILL MODEL (in red):

	tp	ga	gw
val:	0.252	-7	7.1
sd:	0.0293	242	1570

GAIN-LOSS MODEL (in blue):

	tp	ga	gw	la	lw
val:	0.265	-7	6.29	2.26	17.8
sd:	0.0281	143	877	0.0925	43.4

	CNST	HILL	GNLS
AIC:	30.51	-6.63	-7.76
PROB:	0	0.36	0.64
RMSE:	0.36	0.22	0.21

MAX_MEAN: 0.548 MAX_MED: 0.542 BMAD: 0.164

COFF: 0.491 HIT-CALL: 0 FITC: 24 ACTP: 1

FLAGS:

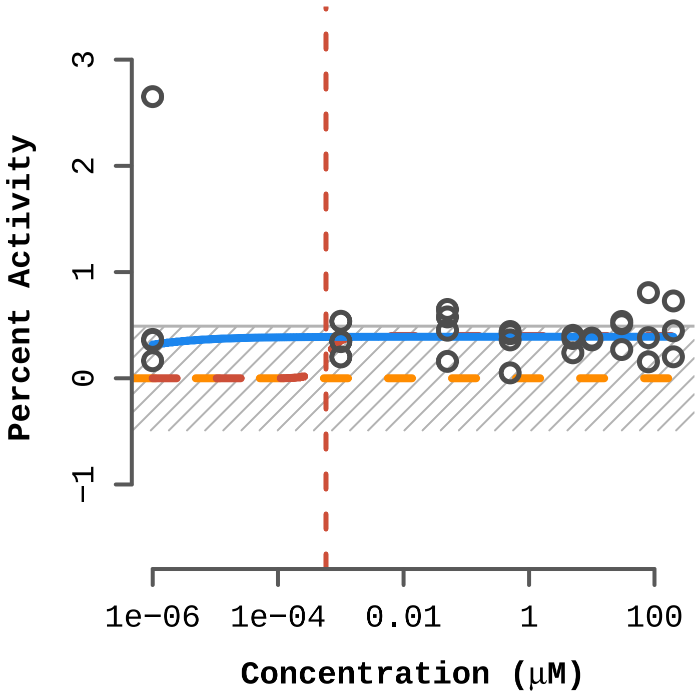

ASSAY: AEID3095 (CCTE_Deisenroth_DEVTOX_RUES2-GLR_Sox2

NAME: Folic acid
CHID: 22519 CASRN: 59-30-3
SPID(S): ES211_33_33
M4ID: 42676016

HILL MODEL (in red):

	tp	ga	gw
val:	0.398	-3.24	3.83
sd:	NaN	NaN	NaN

GAIN-LOSS MODEL (in blue):

	tp	ga	gw	la	lw
val:	0.391	-7	0.625	4.12	4.27
sd:	NaN	NaN	NaN	NaN	NaN

	CNST	HILL	GNLS
AIC:	52.89	14.09	8.86
PROB:	0	0.07	0.93
RMSE:	0.63	0.51	0.45

MAX_MEAN: 0.885 MAX_MED: 0.515 BMAD: 0.164

COFF: 0.491 HIT-CALL: 0 FITC: 26 ACTP: 1

FLAGS: 8; 12

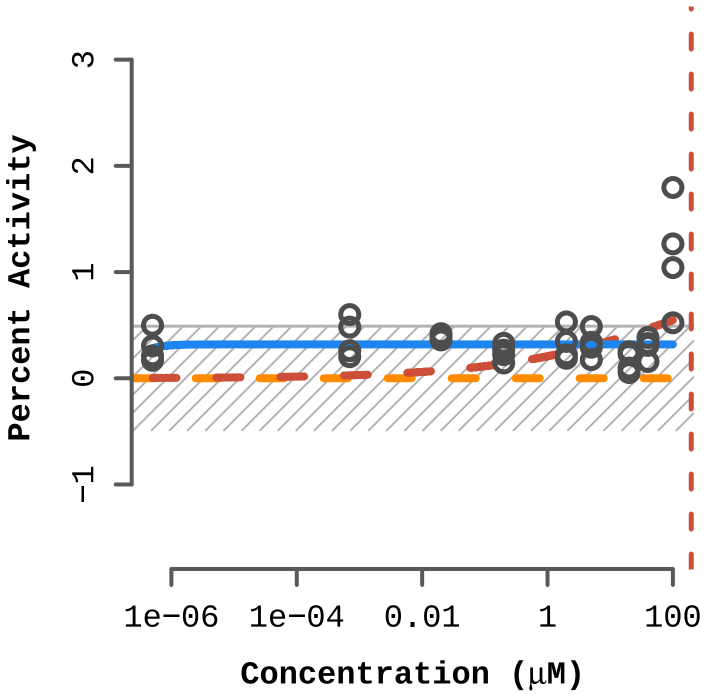

ASSAY: AEID3095 (CCTE_Deisenroth_DEVTOX_RUES2-GLR_Sox[illegible]

NAME: Ketanserin tartrate
CHID: 1003945 CASRN: 83846-83-7
SPID(S): ES211_40_40
M4ID: 42675996

HILL MODEL (in red):

	tp	ga	gw
val:	1.22	2.29	0.3
sd:	NaN	NaN	NaN

GAIN-LOSS MODEL (in blue):

	tp	ga	gw	la	lw
val:	0.319	-6.71	2.17	2.98	18
sd:	NA	NA	NA	NA	NA

	CNST	HILL	GNLS
AIC:	52.43	30.8	10.58
PROB:	0	0	1
RMSE:	0.52	0.35	0.34

MAX_MEAN: 1.16 MAX_MED: 1.15 BMAD: 0.164

COFF: 0.491 HIT-CALL: 0 FITC: 27 ACTP: 1

FLAGS: 12

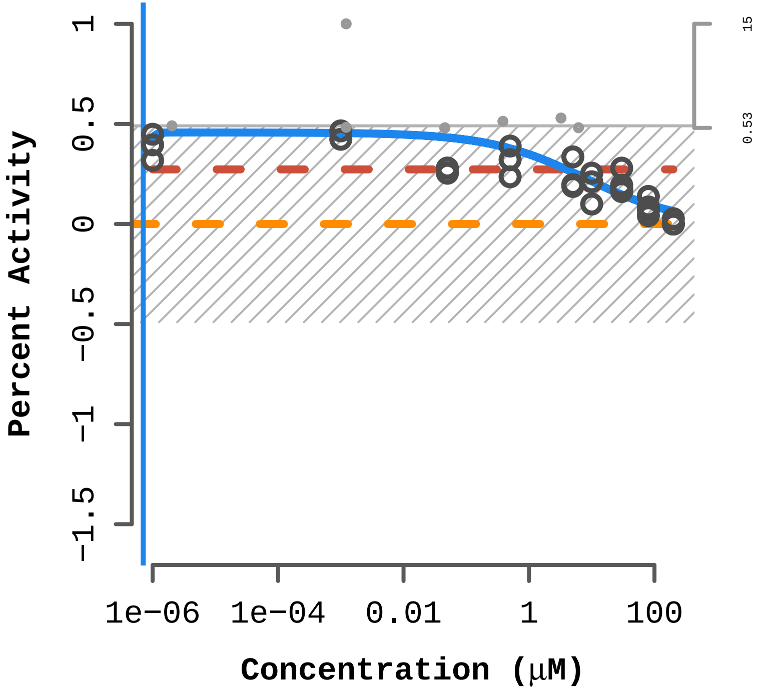

ASSAY: AEID3095 (CCTE_Deisenroth_DEVTOX_RUES2-GLR_Sox2

NAME: Methyldopa sesquihydrate
CHID: 20863 CASRN: 41372-08-1
SPID(S): ES211_46_46
M4ID: 42676003 BRK

HILL MODEL (in red):

	tp	ga	gw
val:	0.273	-7	6.79
sd:	0.0424	172	1110

GAIN-LOSS MODEL (in blue):

	tp	ga	gw	la	lw
val:	0.458	-6.15	7.95	0.897	0.562
sd:	NaN	NaN	NaN	NaN	NaN

	CNST	HILL	GNLS
AIC:	76.36	54.07	32.15
PROB:	0	0	1
RMSE:	2.49	2.42	2.39

MAX_MEAN: 4.03 MAX_MED: 0.534 BMAD: 0.164

COFF: 0.491 HIT-CALL: 0 FITC: 29 ACTP: 1

FLAGS: 10; 12

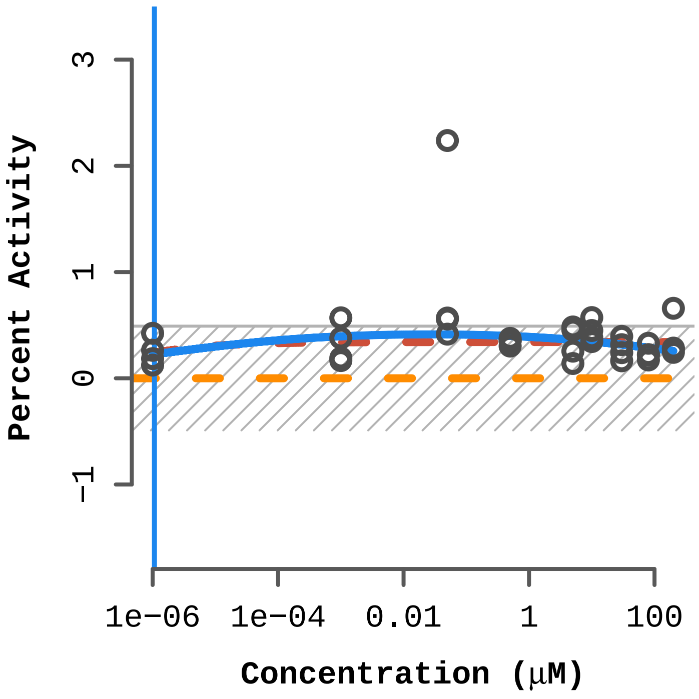

ASSAY: AEID3095 (CCTE_Deisenroth_DEVTOX_RUES2-GLR_Sox2

NAME: Saccharin
CHID: 21251 CASRN: 81-07-2
SPID(S): ES211_54_54
M4ID: 42675986

HILL MODEL (in red):

	tp	ga	gw
val:	0.341	-6.64	0.57
sd:	0.027	1.86	1.54

GAIN-LOSS MODEL (in blue):

	tp	ga	gw	la	lw
val:	0.453	-5.97	0.3	2.68	0.314
sd:	NaN	NaN	NaN	NaN	NaN

	CNST	HILL	GNLS
AIC:	48.9	-11.69	-12.25
PROB:	0	0.43	0.57
RMSE:	0.52	0.34	0.33

MAX_MEAN: 0.945 MAX_MED: 0.563 BMAD: 0.164

COFF: 0.491 HIT-CALL: 0 FITC: 29 ACTP: 1

FLAGS: 12

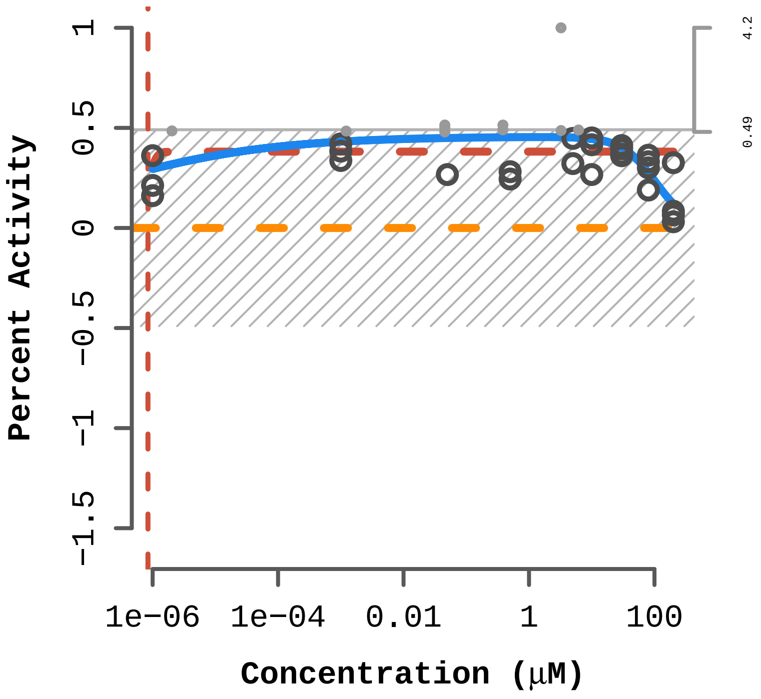

ASSAY: AEID3095 (CCTE_Deisenroth_DEVTOX_RUES2-GLR_Sox2

NAME: Sulfasalazine
CHID: 21256 CASRN: 599-79-1
SPID(S): ES211_59_59
M4ID: 42675981 BRK

HILL MODEL (in red):

	tp	ga	gw
val:	0.381	-6.07	7.96
sd:	0.0306	0.081	9.53

GAIN-LOSS MODEL (in blue):

	tp	ga	gw	la	lw
val:	0.457	-6.81	0.32	2.02	1.59
sd:	0.063	1.41	0.409	0.147	1.05

	CNST	HILL	GNLS
AIC:	62.08	6.66	-4.95
PROB:	0	0	1
RMSE:	0.81	0.66	0.64

MAX_MEAN: 1.38 MAX_MED: 0.57 BMAD: 0.164

COFF: 0.491 HIT-CALL: 0 FITC: 29 ACTP: 1

FLAGS: 8; 10; 12

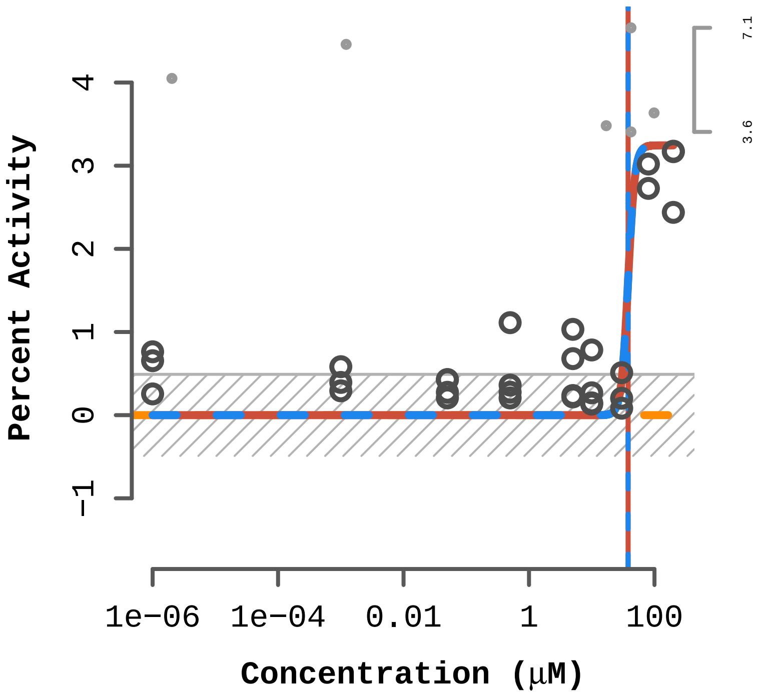

ASSAY: AEID3095 (CCTE_Deisenroth_DEVTOX_RUES2-GLR_Sox2

NAME: Bisphenol A
CHID: 20182 CASRN: 80-05-7
SPID(S): ES211_16_16
M4ID: 42676031 BRK

HILL MODEL (in red):

	tp	ga	gw
val:	3.24	1.58	8
sd:	0.287	0.306	23.4

GAIN-LOSS MODEL (in blue):

	tp	ga	gw	la	lw
val:	3.24	1.58	8	4.3	4.19
sd:	NaN	NaN	NaN	NaN	NaN

	CNST	HILL	GNLS
AIC:	161.85	120.88	124.88
PROB:	0	0.88	0.12
RMSE:	2.45	1.72	1.72

MAX_MEAN: 4.11 MAX_MED: 3.32 BMAD: 0.164

COFF: 0.491 HIT-CALL: 1 FITC: 41 ACTP: 1

FLAGS: 10

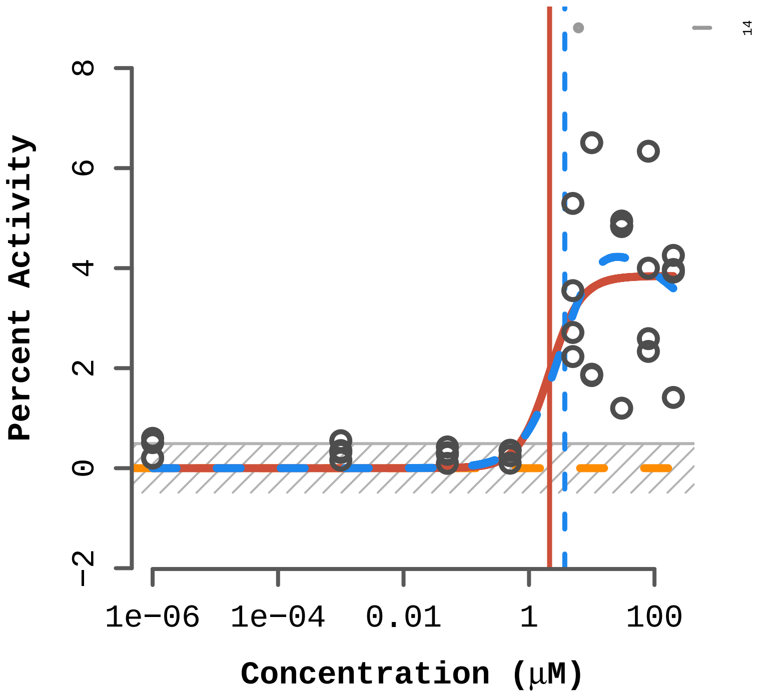

ASSAY: AEID3095 (CCTE_Deisenroth_DEVTOX_RUES2-GLR_Sox2

NAME: Diethylstilbestrol
CHID: 20465 CASRN: 56-53-1
SPID(S): ES211_29_29
M4ID: 42675973 BRK

HILL MODEL (in red):

	tp	ga	gw
val:	3.84	0.328	1.74
sd:	0.391	0.239	0.856

GAIN-LOSS MODEL (in blue):

	tp	ga	gw	la	lw
val:	6.4	0.571	1.39	2.67	0.3
sd:	33.8	0.872	0.762	14.6	2.37

	CNST	HILL	GNLS
AIC:	191.9	135.95	139.41
PROB:	0	0.85	0.15
RMSE:	3.71	2.1	2.06

MAX_MEAN: 6.09 MAX_MED: 4.84 BMAD: 0.164

COFF: 0.491 HIT-CALL: 1 FITC: 41 ACTP: 1

FLAGS: 10

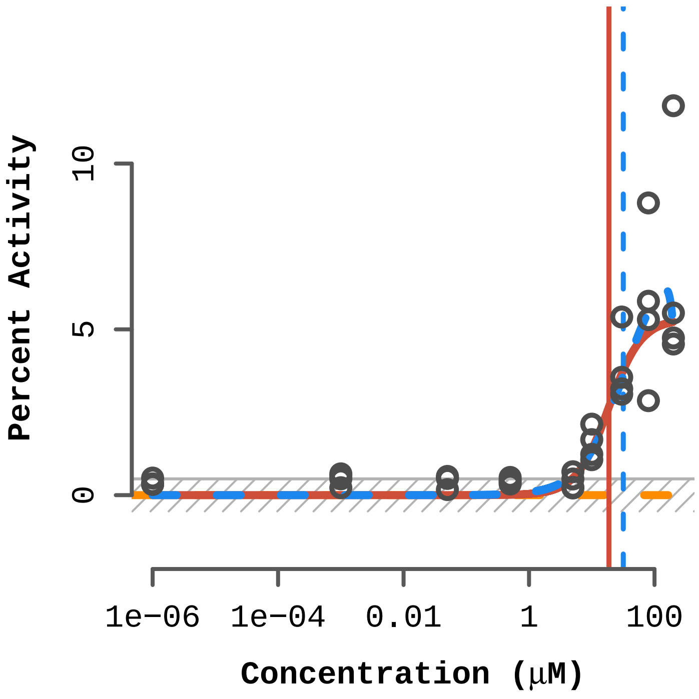

ASSAY: AEID3095 (CCTE_Deisenroth_DEVTOX_RUES2-GLR_Sox2

NAME: 5-Fluorouracil
CHID: 20634 CASRN: 51-21-8
SPID(S): ES211_5_5
M4ID: 42676009

HILL MODEL (in red):

	tp	ga	gw
val:	5.33	1.27	1.68
sd:	0.417	0.0806	0.337

GAIN-LOSS MODEL (in blue):

	tp	ga	gw	la	lw
val:	7.23	1.5	1.28	2.35	9.49
sd:	2.13	0.246	0.374	0.0223	6.4

	CNST	HILL	GNLS
AIC:	186.07	104.22	105.55
PROB:	0	0.66	0.34
RMSE:	3.48	1.41	1.42

MAX_MEAN: 6.64 MAX_MED: 5.57 BMAD: 0.164

COFF: 0.491 HIT-CALL: 1 FITC: 41 ACTP: 1

FLAGS: 10

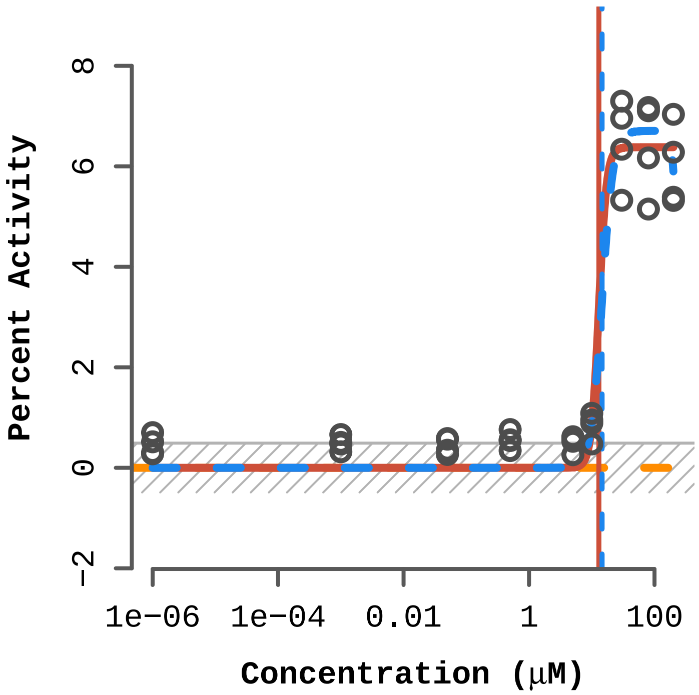

ASSAY: AEID3095 (CCTE_Deisenroth_DEVTOX_RUES2-GLR_Sox[illegible]

NAME: 1-(p-Chlorobenzoyl)-5-methoxy-2-methyl-Indole-[illegible]
CHID: 20740 CASRN: 53-86-1
SPID(S): ES211_38_38
M4ID: 42675984

HILL MODEL (in red):

	tp	ga	gw
val:	6.38	1.11	7.06
sd:	0.244	0.138	8.65

GAIN-LOSS MODEL (in blue):

	tp	ga	gw	la	lw
val:	6.71	1.16	4.96	2.38	11.1
sd:	NaN	NaN	NaN	NaN	NaN

	CNST	HILL	GNLS
AIC:	196.95	78.33	79.93
PROB:	0	0.69	0.31
RMSE:	3.69	0.6	0.59

MAX_MEAN: 6.48 MAX_MED: 6.65 BMAD: 0.164

COFF: 0.491 HIT-CALL: 1 FITC: 41 ACTP: 1

FLAGS: 10

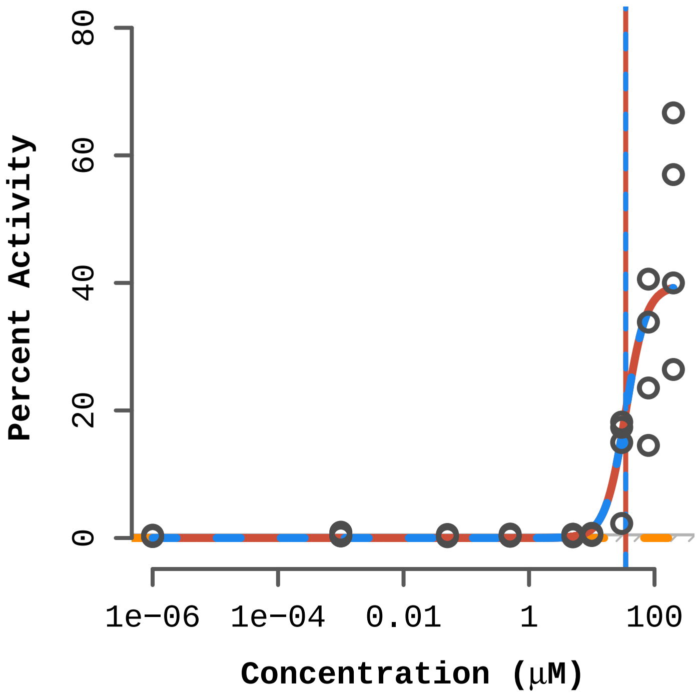

ASSAY: AEID3095 (CCTE_Deisenroth_DEVTOX_RUES2-GLR_Sox2

NAME: 13-cis Retinoic acid
CHID: 23177 CASRN: 4759-48-2
SPID(S): ES211_2_2
M4ID: 42676022

HILL MODEL (in red):

	tp	ga	gw
val:	39.6	1.54	2.65
sd:	2.26	0.0406	0.586

GAIN-LOSS MODEL (in blue):

	tp	ga	gw	la	lw
val:	39.6	1.54	2.65	3.38	13.9
sd:	NA	NA	NA	NA	NA

	CNST	HILL	GNLS
AIC:	324.4	226.05	230.05
PROB:	0	0.88	0.12
RMSE:	20.01	7.56	7.56

MAX_MEAN: 47.5 MAX_MED: 48.5 BMAD: 0.164

COFF: 0.491 HIT-CALL: 1 FITC: 41 ACTP: 1

FLAGS: 10

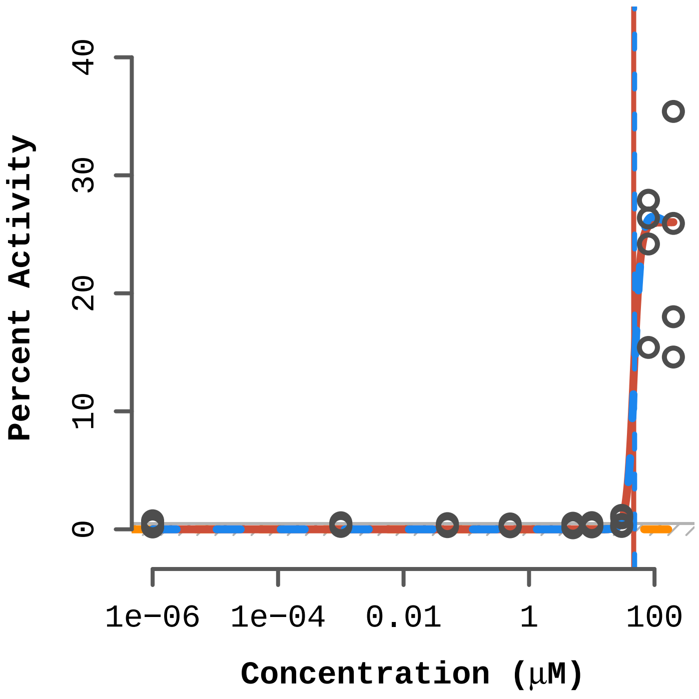

ASSAY: AEID3095 (CCTE_Deisenroth_DEVTOX_RUES2-GLR_Sox2

NAME: Retinol
CHID: 23556 CASRN: 68-26-8
SPID(S): ES211_51_51
M4ID: 42676028

HILL MODEL (in red):

	tp	ga	gw
val:	26	1.67	8
sd:	0.589	0.0477	1.96

GAIN-LOSS MODEL (in blue):

	tp	ga	gw	la	lw
val:	29	1.68	7.68	4.15	0.479
sd:	NaN	NaN	NaN	NaN	NaN

	CNST	HILL	GNLS
AIC:	264.23	151.73	154.93
PROB:	0	0.83	0.17
RMSE:	11.51	3.33	3.36

MAX_MEAN: 23.5 MAX_MED: 25.3 BMAD: 0.164

COFF: 0.491 HIT-CALL: 1 FITC: 41 ACTP: 1

FLAGS: 10

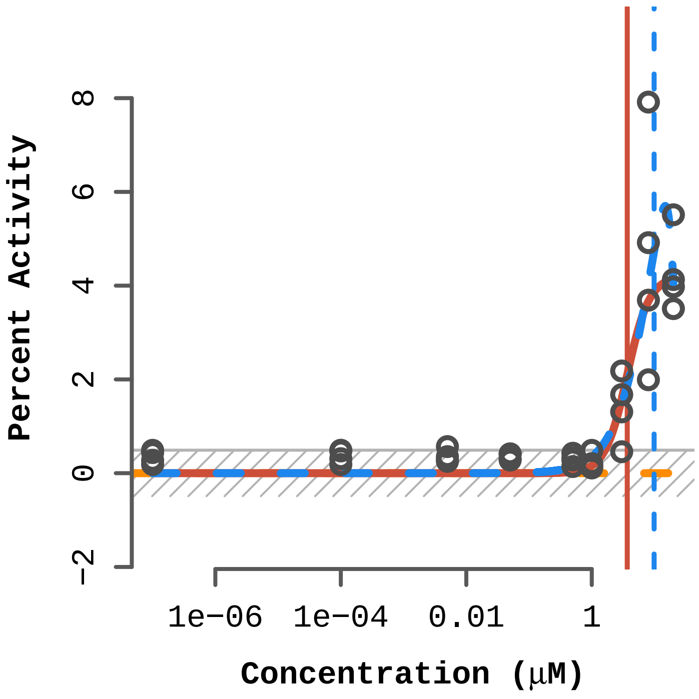

ASSAY: AEID3095 (CCTE_Deisenroth_DEVTOX_RUES2-GLR_Sox2

NAME: Ziprasidone
CHID: 23753 CASRN: 146939-27-7
SPID(S): ES211_66_66
M4ID: 42676019

HILL MODEL (in red):

	tp	ga	gw
val:	4.2	0.565	2.47
sd:	0.341	0.0669	1.19

GAIN-LOSS MODEL (in blue):

	tp	ga	gw	la	lw
val:	9.5	0.994	1.43	1.32	7.81
sd:	15.9	0.886	0.721	0.0217	40

	CNST	HILL	GNLS
AIC:	152.42	79.9	82.18
PROB:	0	0.76	0.24
RMSE:	2.31	0.9	0.86

MAX_MEAN: 4.63 MAX_MED: 4.3 BMAD: 0.164

COFF: 0.491 HIT-CALL: 1 FITC: 41 ACTP: 1

FLAGS: 10

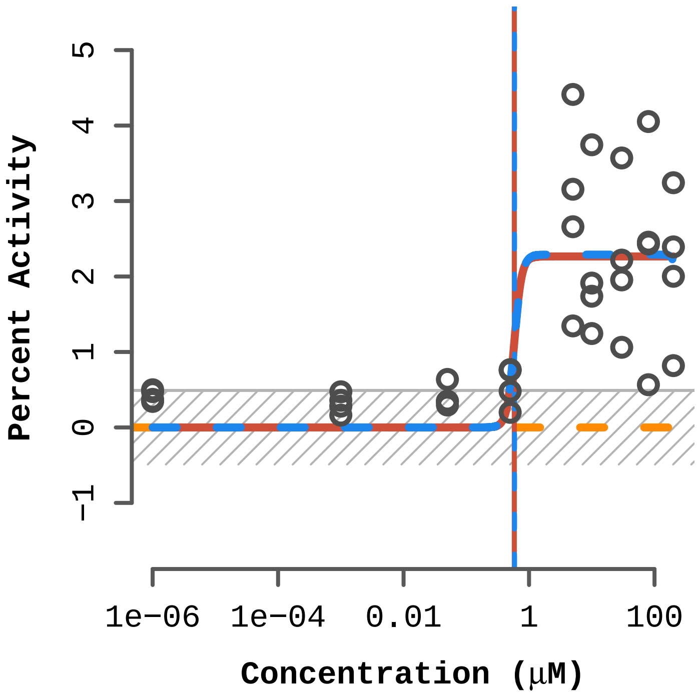

ASSAY: AEID3095 (CCTE_Deisenroth_DEVTOX_RUES2-GLR_Sox[illegible]

NAME: Cytarabine hydrochloride
CHID: 24891 CASRN: 69-74-9
SPID(S): ES211_25_25
M4ID: 42676000

HILL MODEL (in red):

	tp	ga	gw
val:	2.27	-0.235	7.39
sd:	0.202	2.31	258

GAIN-LOSS MODEL (in blue):

	tp	ga	gw	la	lw
val:	2.29	-0.231	7.12	2.41	13
sd:	NaN	NaN	NaN	NaN	NaN

	CNST	HILL	GNLS
AIC:	153.45	96.1	100.06
PROB:	0	0.88	0.12
RMSE:	1.95	0.83	0.83

MAX_MEAN: 2.89 MAX_MED: 2.91 BMAD: 0.164

COFF: 0.491 HIT-CALL: 1 FITC: 41 ACTP: 1

FLAGS: 10

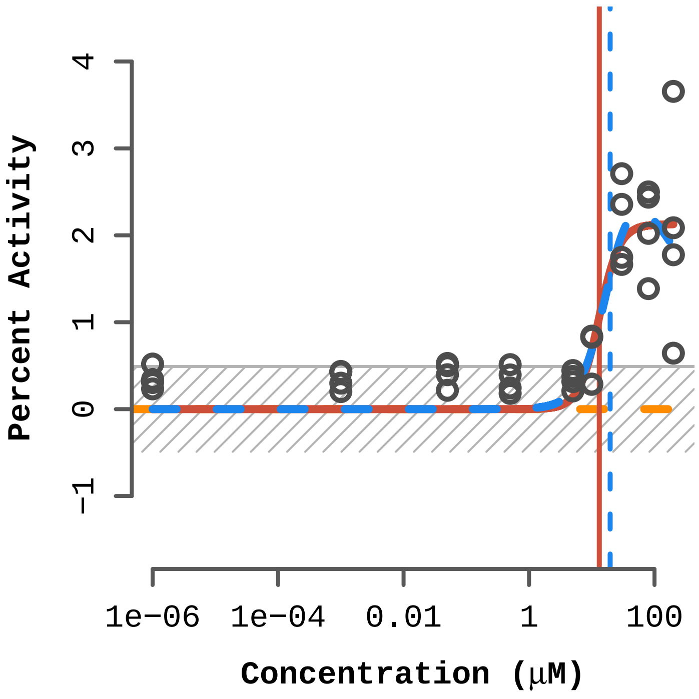

ASSAY: AEID3095 (CCTE_Deisenroth_DEVTOX_RUES2-GLR_Sox2

NAME: 5-Bromo-2'-deoxyuridine
CHID: 33105 CASRN: 59-14-3
SPID(S): ES211_4_4
M4ID: 42675994

HILL MODEL (in red):

	tp	ga	gw
val:	2.13	1.12	2.72
sd:	0.179	0.0751	1.19

GAIN-LOSS MODEL (in blue):

	tp	ga	gw	la	lw
val:	4.39	1.29	2.01	2.04	0.477
sd:	8.04	0.233	0.736	3.32	0.842

	CNST	HILL	GNLS
AIC:	121.96	58.39	60.62
PROB:	0	0.75	0.25
RMSE:	1.32	0.5	0.5

MAX_MEAN: 2.12 MAX_MED: 2.23 BMAD: 0.164

COFF: 0.491 HIT-CALL: 1 FITC: 41 ACTP: 1

FLAGS: 10

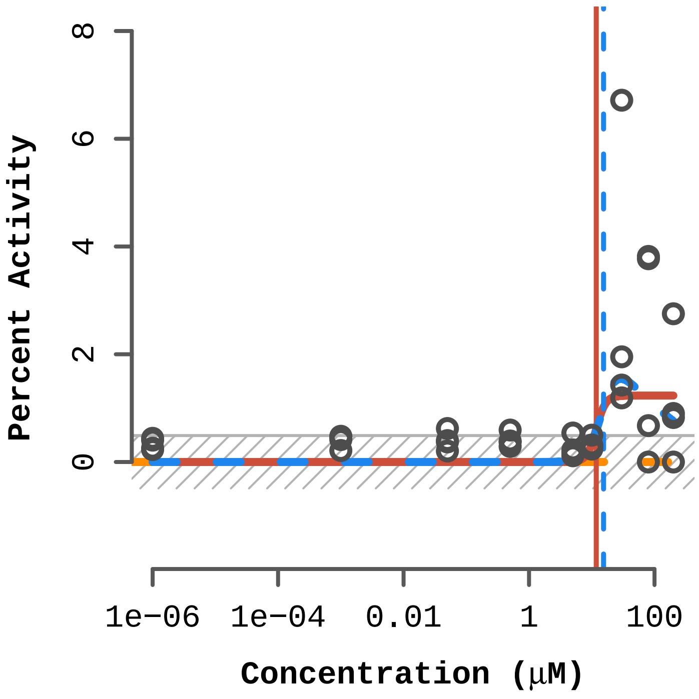

ASSAY: AEID3095 (CCTE_Deisenroth_DEVTOX_RUES2-GLR_Sox2

NAME: Amiodarone hydrochloride
CHID: 37185 CASRN: 19774-82-4
SPID(S): ES211_14_14
M4ID: 42675988

HILL MODEL (in red):

	tp	ga	gw
val:	1.23	1.07	5.81
sd:	0.269	0.132	11.6

GAIN-LOSS MODEL (in blue):

	tp	ga	gw	la	lw
val:	3.44	1.19	3.72	1.44	0.636
sd:	9.09	0.304	5.09	3.41	0.923

	CNST	HILL	GNLS
AIC:	116.26	99.56	100.6
PROB:	0	0.63	0.37
RMSE:	1.62	1.21	1.18

MAX_MEAN: 2.82 MAX_MED: 2.22 BMAD: 0.164

COFF: 0.491 HIT-CALL: 1 FITC: 41 ACTP: 1

FLAGS: 10

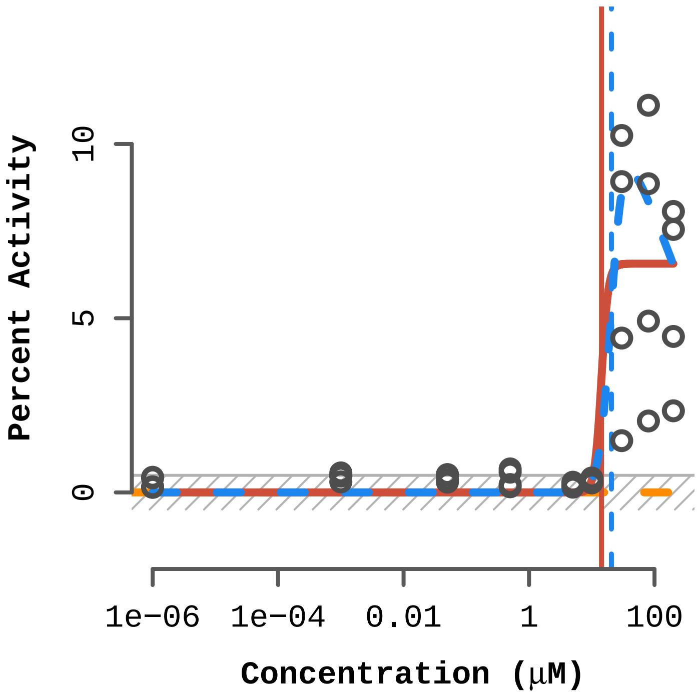

ASSAY: AEID3095 (CCTE_Deisenroth_DEVTOX_RUES2-GLR_Sox2

NAME: Imatinib mesylate
CHID: 40502 CASRN: 220127-57-1
SPID(S): ES211_37_37
M4ID: 42676027

HILL MODEL (in red):

	tp	ga	gw
val:	6.56	1.15	8
sd:	1.44	0.375	19.4

GAIN-LOSS MODEL (in blue):

	tp	ga	gw	la	lw
val:	13.3	1.31	4.72	2.27	0.611
sd:	68.4	0.412	3.73	7.35	4.04

	CNST	HILL	GNLS
AIC:	193.94	147.79	150.43
PROB:	0	0.79	0.21
RMSE:	4.04	1.88	2.1

MAX_MEAN: 6.74 MAX_MED: 6.89 BMAD: 0.164

COFF: 0.491 HIT-CALL: 1 FITC: 41 ACTP: 1

FLAGS: 10

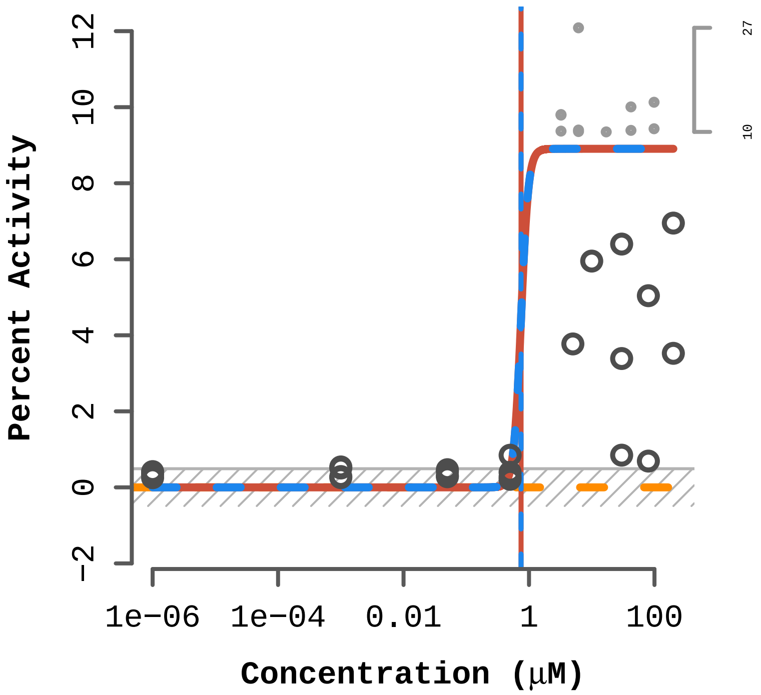

ASSAY: AEID3095 (CCTE_Deisenroth_DEVTOX_RUES2-GLR_Sox2

NAME: Sorafenib
CHID: 41128 CASRN: 284461-73-0
SPID(S): ES211_57_57
M4ID: 42676017 BRK

HILL MODEL (in red):

	tp	ga	gw
val:	8.9	-0.126	7.4
sd:	0.983	4.71	200

GAIN-LOSS MODEL (in blue):

	tp	ga	gw	la	lw
val:	8.91	-0.127	7.41	3.19	7.87
sd:	0.983	4.75	202	1890	17000

	CNST	HILL	GNLS
AIC:	251.27	205.91	209.91
PROB:	0	0.88	0.12
RMSE:	8.16	4.44	4.44

MAX_MEAN: 13.6 MAX_MED: 11.9 BMAD: 0.164

COFF: 0.491 HIT-CALL: 1 FITC: 41 ACTP: 1

FLAGS: 10

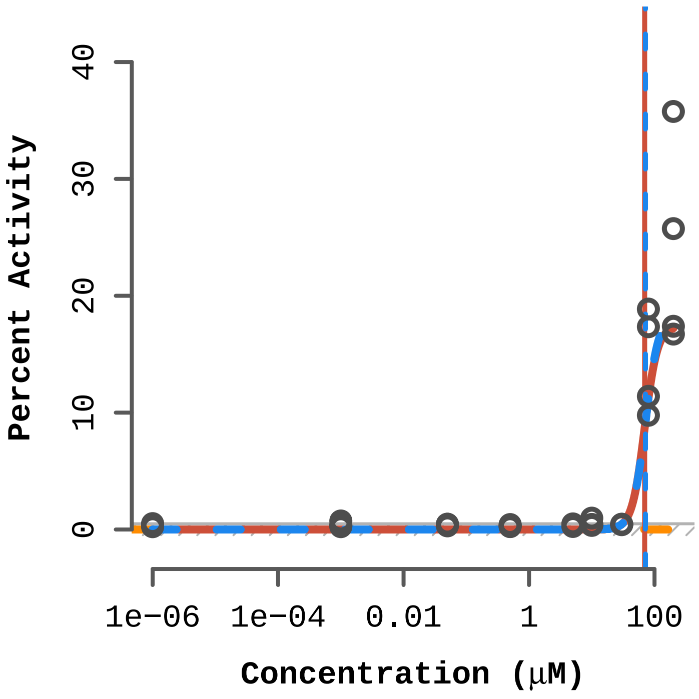

ASSAY: AEID3095 (CCTE_Deisenroth_DEVTOX_RUES2-GLR_Sox2

NAME: Esomeprazole magnesium hydrate
CHID: 746660 CASRN: 668985-31-7
SPID(S): ES211_43_43
M4ID: 42676036

HILL MODEL (in red):

	tp	ga	gw
val:	17.4	1.84	4.31
sd:	0.478	0.0177	0.675

GAIN-LOSS MODEL (in blue):

	tp	ga	gw	la	lw
val:	18.3	1.86	4.22	2.38	16.9
sd:	NaN	NaN	NaN	NaN	NaN

	CNST	HILL	GNLS
AIC:	254.05	143.75	147.75
PROB:	0	0.88	0.12
RMSE:	9.74	3.8	3.8

MAX_MEAN: 23.9 MAX_MED: 21.6 BMAD: 0.164

COFF: 0.491 HIT-CALL: 1 FITC: 41 ACTP: 1

FLAGS: 10

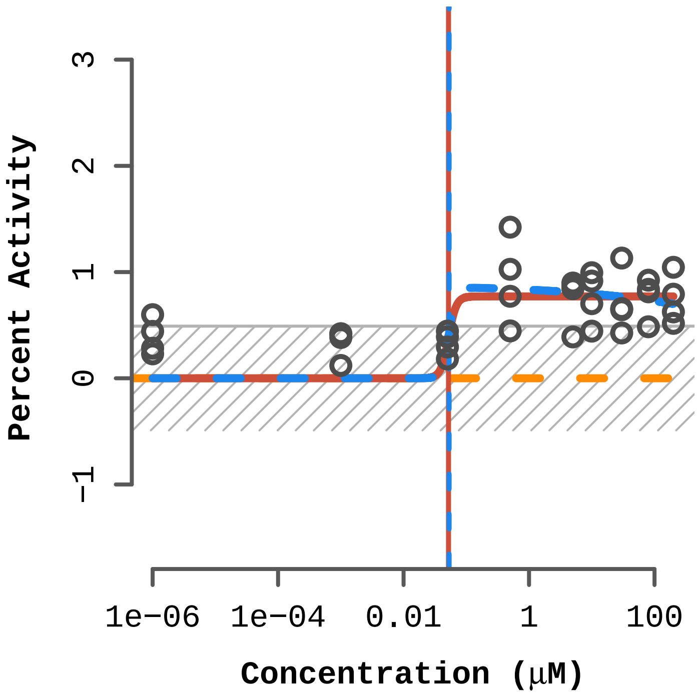

ASSAY: AEID3095 (CCTE_Deisenroth_DEVTOX_RUES2-GLR_Sox2

NAME: Methotrexate hydrate(1:x)
CHID: 1017640 CASRN: 133073-73-1
SPID(S): ES211_45_45
M4ID: 42675999

HILL MODEL (in red):

	tp	ga	gw
val:	0.771	−1.28	7.06
sd:	0.0558	0.636	250

GAIN-LOSS MODEL (in blue):

	tp	ga	gw	la	lw
val:	0.876	−1.27	7.58	4.3	0.3
sd:	NaN	NaN	NaN	NaN	NaN

	CNST	HILL	GNLS
AIC:	84.36	20.48	24.08
PROB:	0	0.86	0.14
RMSE:	0.7	0.28	0.27

MAX_MEAN: 0.917 MAX_MED: 0.899 BMAD: 0.164

COFF: 0.491 HIT-CALL: 1 FITC: 41 ACTP: 1

FLAGS:

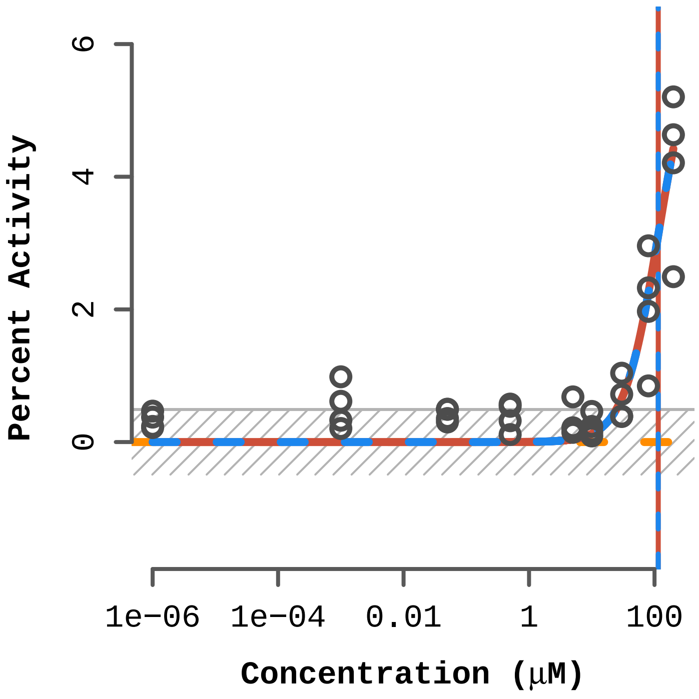

ASSAY: AEID3095 (CCTE_Deisenroth_DEVTOX_RUES2-GLR_Sox2

NAME: 4-Hydroxybenzoic acid butyl ester
CHID: 20209 CASRN: 94-26-8
SPID(S): ES211_19_19
M4ID: 42675998

HILL MODEL (in red):

	tp	ga	gw
val:	6.25	2.06	1.6
sd:	1.72	0.181	0.435

GAIN-LOSS MODEL (in blue):

	tp	ga	gw	la	lw
val:	6.25	2.06	1.6	3.62	5.96
sd:	1.72	0.181	0.435	944	4270

	CNST	HILL	GNLS
AIC:	126.3	63.74	67.74
PROB:	0	0.88	0.12
RMSE:	1.65	0.56	0.56

MAX_MEAN: 4.14 MAX_MED: 4.42 BMAD: 0.164

COFF: 0.491 HIT-CALL: 1 FITC: 42 ACTP: 1

FLAGS: 10

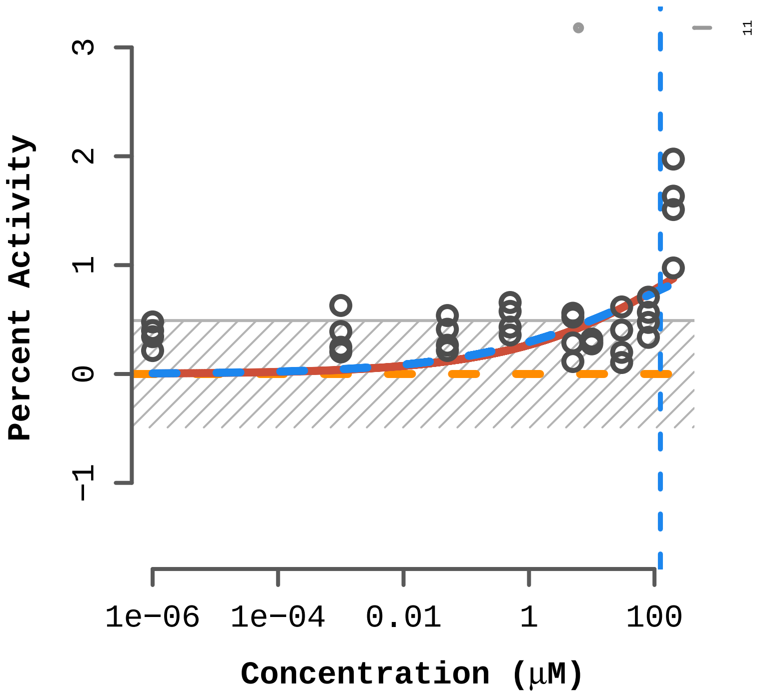

ASSAY: AEID3095 (CCTE_Deisenroth_DEVTOX_RUES2-GLR_Sox2

NAME: Diphenhydramine hydrochloride
CHID: 20537 CASRN: 147-24-0
SPID(S): ES211_31_31
M4ID: 42676021 BRK

HILL MODEL (in red):

	tp	ga	gw
val:	2.12	2.8	0.3
sd:	2.51	2.99	0.0674

GAIN-LOSS MODEL (in blue):

	tp	ga	gw	la	lw
val:	1.55	2.09	0.3	3.79	5.64
sd:	NaN	NaN	NaN	NaN	NaN

	CNST	HILL	GNLS
AIC:	94.39	66.92	71.24
PROB:	0	0.9	0.1
RMSE:	1.91	1.76	1.75

MAX_MEAN: 2.92 MAX_MED: 1.57 BMAD: 0.164

COFF: 0.491 HIT-CALL: 1 FITC: 42 ACTP: 1

FLAGS: 10

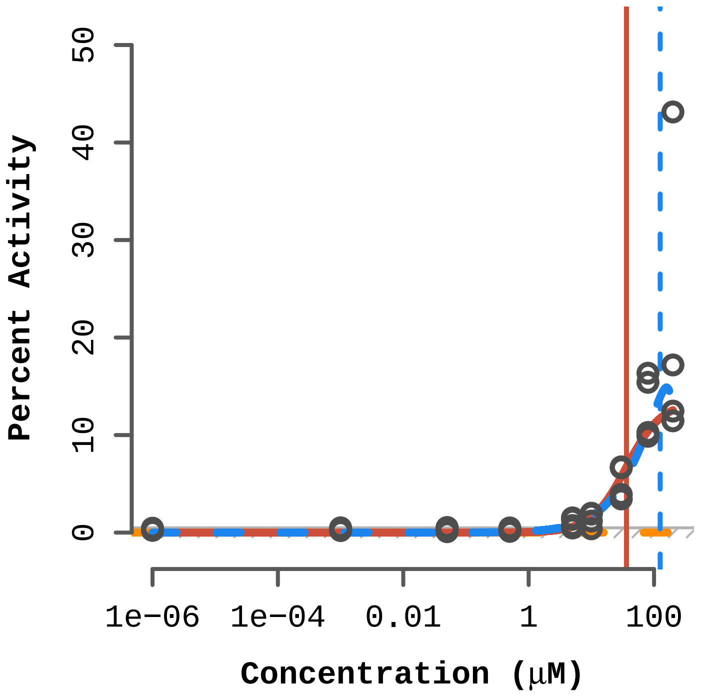

ASSAY: AEID3095 (CCTE_Deisenroth_DEVTOX_RUES2-GLR_Sox2

NAME: Lovastatin
CHID: 20784 CASRN: 75330-75-5
SPID(S): ES211_42_42
M4ID: 42676020

HILL MODEL (in red):

	tp	ga	gw
val:	13.5	1.56	1.55
sd:	1.21	0.0899	0.266

GAIN-LOSS MODEL (in blue):

	tp	ga	gw	la	lw
val:	28.1	2.1	1.09	2.35	8.09
sd:	NaN	NaN	NaN	NaN	NaN

	CNST	HILL	GNLS
AIC:	243.72	153.05	155.95
PROB:	0	0.81	0.19
RMSE:	9.55	5.36	5.37

MAX_MEAN: 21.1 MAX_MED: 14.8 BMAD: 0.164

COFF: 0.491 HIT-CALL: 1 FITC: 42 ACTP: 1

FLAGS: 10

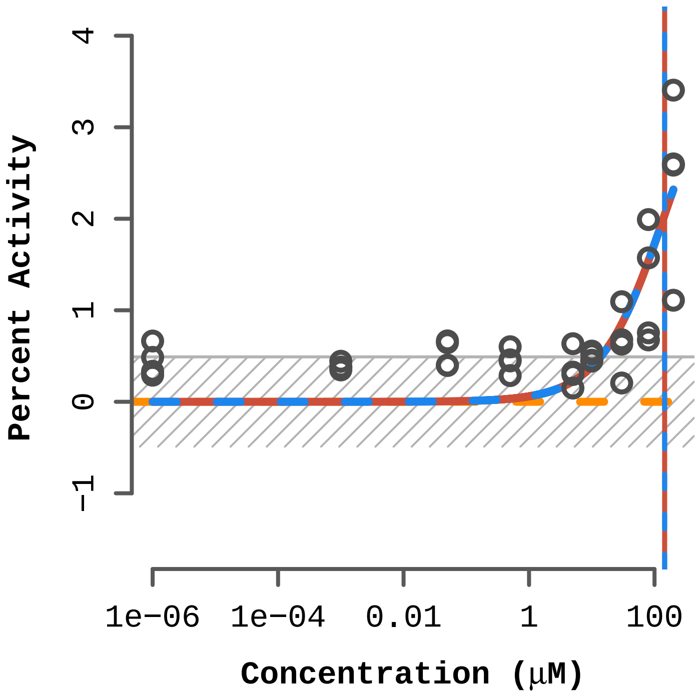

ASSAY: AEID3095 (CCTE_Deisenroth_DEVTOX_RUES2-GLR_Sox2

NAME: Busulfan
CHID: 20910 CASRN: 55-98-1
SPID(S): ES211_18_18
M4ID: 42676012

HILL MODEL (in red):

	tp	ga	gw
val:	4.09	2.16	0.846
sd:	1.58	0.384	0.253

GAIN-LOSS MODEL (in blue):

	tp	ga	gw	la	lw
val:	4.09	2.16	0.846	4.18	3.69
sd:	1.58	0.385	0.253	337	666

	CNST	HILL	GNLS
AIC:	102.5	60.87	64.87
PROB:	0	0.88	0.12
RMSE:	1.07	0.49	0.49

MAX_MEAN: 2.43 MAX_MED: 2.59 BMAD: 0.164

COFF: 0.491 HIT-CALL: 1 FITC: 42 ACTP: 1

FLAGS:

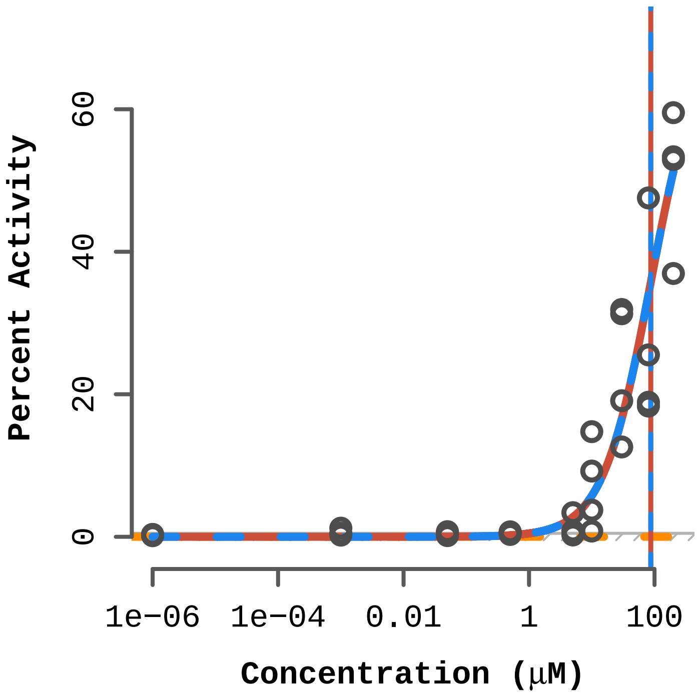

ASSAY: AEID3095 (CCTE_Deisenroth_DEVTOX_RUES2-GLR_Sox[illegible]

NAME: all-trans-Retinoic acid
CHID: 21239 CASRN: 302-79-4
SPID(S): ES211_13_13
M4ID: 42676029

HILL MODEL (in red):

	tp	ga	gw
val:	71.4	1.94	1.12
sd:	20.1	0.311	0.233

GAIN-LOSS MODEL (in blue):

	tp	ga	gw	la	lw
val:	71.4	1.94	1.12	3.47	7.6
sd:	20.1	0.311	0.233	1270	8260

	CNST	HILL	GNLS
AIC:	328.72	240.72	244.72
PROB:	0	0.88	0.12
RMSE:	21.74	6.72	6.72

MAX_MEAN: 50.7 MAX_MED: 53.1 BMAD: 0.164

COFF: 0.491 HIT-CALL: 1 FITC: 42 ACTP: 1

FLAGS: 10

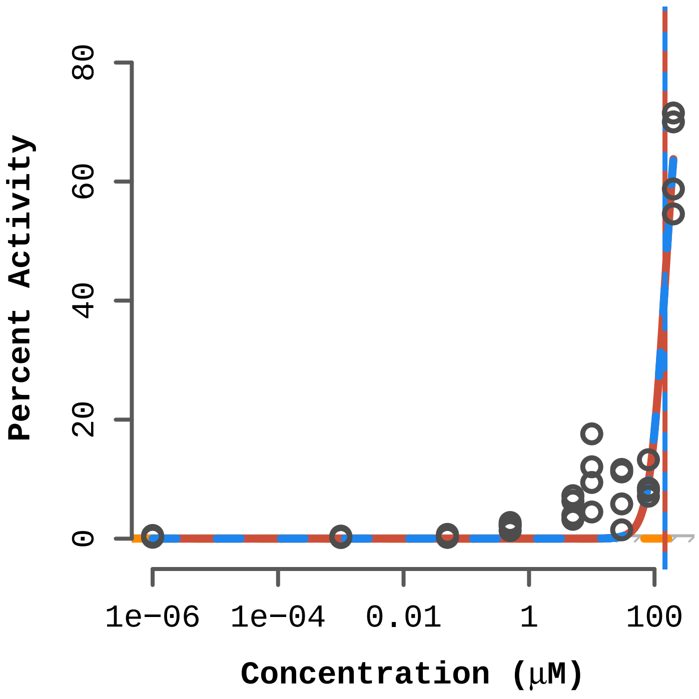

ASSAY: AEID3095 (CCTE_Deisenroth_DEVTOX_RUES2-GLR_Sox2

NAME: Dasatinib
CHID: 40979 CASRN: 302962-49-8
SPID(S): ES211_26_26
M4ID: 42675974

HILL MODEL (in red):

	tp	ga	gw
val:	85.8	2.17	3.44
sd:	NaN	NaN	NaN

GAIN-LOSS MODEL (in blue):

	tp	ga	gw	la	lw
val:	85.8	2.17	3.44	3.16	11.3
sd:	NaN	NaN	NaN	NaN	NaN

	CNST	HILL	GNLS
AIC:	299.98	233.63	237.63
PROB:	0	0.88	0.12
RMSE:	22.25	5.8	5.8

MAX_MEAN: 63.7 MAX_MED: 64.4 BMAD: 0.164

COFF: 0.491 HIT-CALL: 1 FITC: 42 ACTP: 1

FLAGS: 10

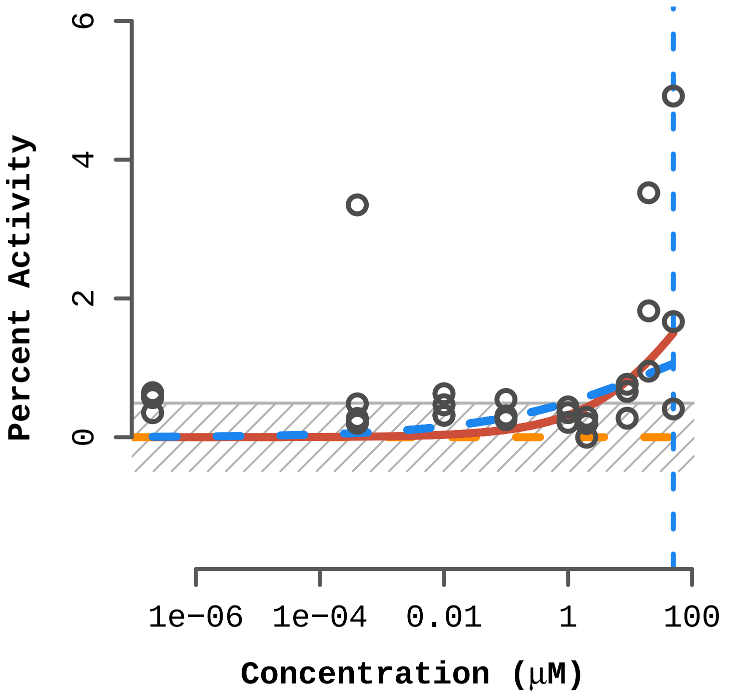

ASSAY: AEID3095 (CCTE_Deisenroth_DEVTOX_RUES2-GLR_Sox[illegible]

NAME: Ritanserin
CHID: 42594 CASRN: 87051-43-2
SPID(S): ES211_53_53
M4ID: 42675980

HILL MODEL (in red):

	tp	ga	gw
val:	4.15	2.2	0.493
sd:	7.48	3.09	0.561

GAIN-LOSS MODEL (in blue):

	tp	ga	gw	la	lw
val:	2.12	1.7	0.3	3.45	7.86
sd:	NaN	NaN	NaN	NaN	NaN

	CNST	HILL	GNLS
AIC:	95.45	80.89	85.31
PROB:	0	0.9	0.1
RMSE:	1.39	1.04	1.09

MAX_MEAN: 2.33 MAX_MED: 1.82 BMAD: 0.164

COFF: 0.491 HIT-CALL: 1 FITC: 42 ACTP: 1

FLAGS: 10

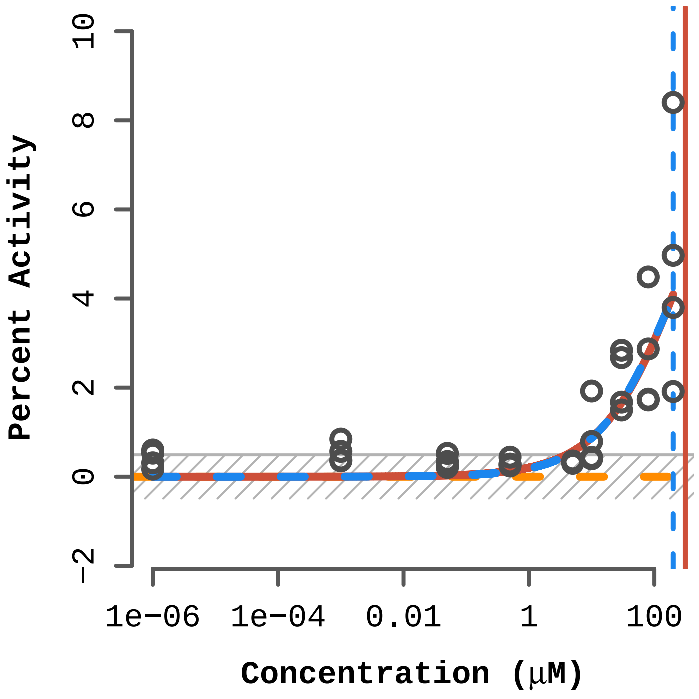

ASSAY: AEID3095 (CCTE_Deisenroth_DEVTOX_RUES2-GLR_Sox2

NAME: 6-aminopyridine-3-carboxamide
CHID: 51446 CASRN: 329-89-5
SPID(S): ES211_7_7
M4ID: 42676013

HILL MODEL (in red):

	tp	ga	gw
val:	9.6	2.49	0.668
sd:	28.2	3.39	0.631

GAIN-LOSS MODEL (in blue):

	tp	ga	gw	la	lw
val:	8.13	2.3	0.709	3.67	5.06
sd:	15.5	2.22	0.552	542	2030

	CNST	HILL	GNLS
AIC:	149.89	95.21	99.22
PROB:	0	0.88	0.12
RMSE:	2.21	1	1

MAX_MEAN: 4.77 MAX_MED: 4.39 BMAD: 0.164

COFF: 0.491 HIT-CALL: 1 FITC: 42 ACTP: 1

FLAGS: 10

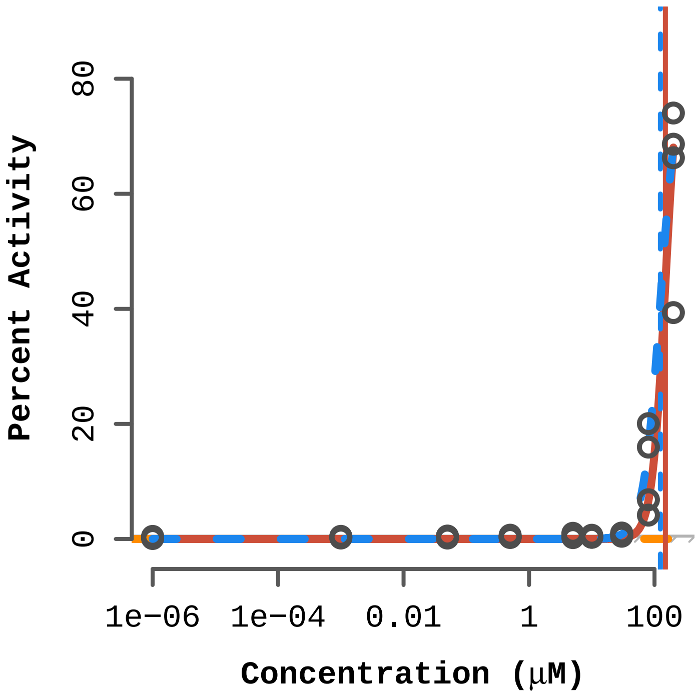

ASSAY: AEID3095 (CCTE_Deisenroth_DEVTOX_RUES2-GLR_Sox2

NAME: Pomalidomide
CHID: 893458 CASRN: 19171-19-8
SPID(S): ES211_50_50
M4ID: 42675987

HILL MODEL (in red):

	tp	ga	gw
val:	88.9	2.17	4.04
sd:	2.48	0.00953	0.232

GAIN-LOSS MODEL (in blue):

	tp	ga	gw	la	lw
val:	83.3	2.09	3.25	4.07	1.16
sd:	NaN	NaN	NaN	NaN	NaN

	CNST	HILL	GNLS
AIC:	274.19	166.49	169.9
PROB:	0	0.85	0.15
RMSE:	21.64	5.63	5.56

MAX_MEAN: 62.1 MAX_MED: 67.4 BMAD: 0.164

COFF: 0.491 HIT-CALL: 1 FITC: 42 ACTP: 1

FLAGS: 10

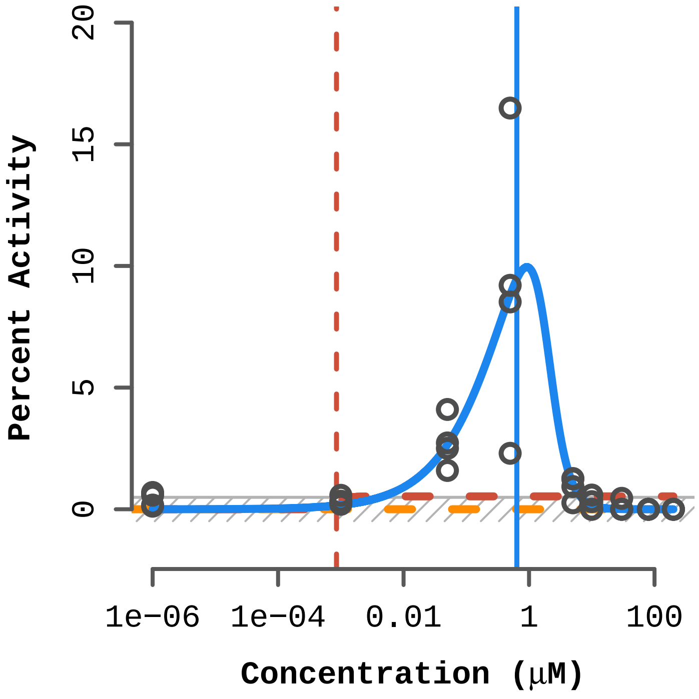

ASSAY: AEID3095 (CCTE_Deisenroth_DEVTOX_RUES2-GLR_Sox[illegible]

NAME: Adriamycin hydrochloride
CHID: 30636 CASRN: 25316-40-9
SPID(S): ES211_67_67
M4ID: 42676030

HILL MODEL (in red):

	tp	ga	gw
val:	0.529	-3.07	4.45
sd:	0.215	4.01	260

GAIN-LOSS MODEL (in blue):

	tp	ga	gw	la	lw
val:	19.8	-0.194	0.731	0.285	2.81
sd:	11	0.629	0.174	0.187	1.15

	CNST	HILL	GNLS
AIC:	155.86	154.12	87.17
PROB:	0	0	1
RMSE:	3.63	3.44	1.72

MAX_MEAN: 9.13 MAX_MED: 8.86 BMAD: 0.164

COFF: 0.491 HIT-CALL: 1 FITC: 50 ACTP: 1

FLAGS: 10

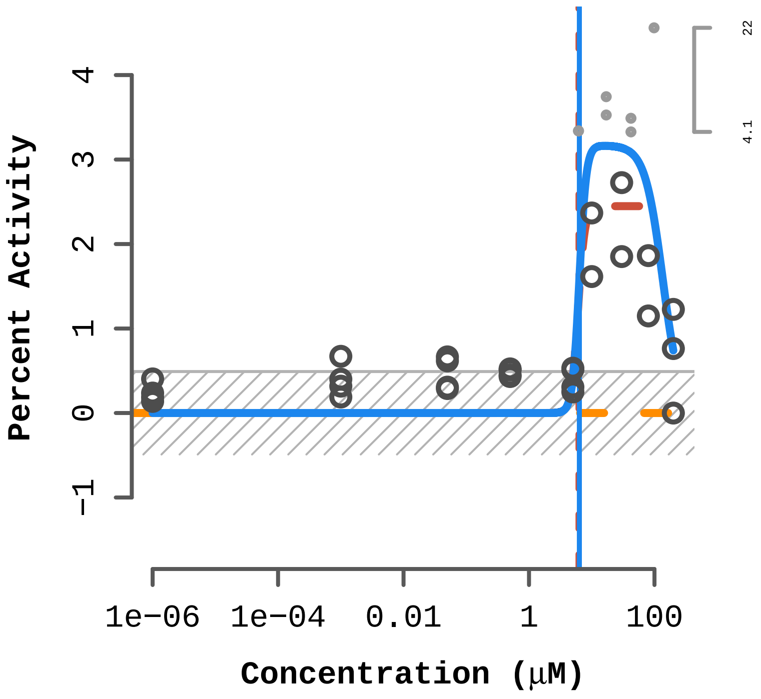

ASSAY: AEID3095 (CCTE_Deisenroth_DEVTOX_RUES2-GLR_Sox2

NAME: Gefitinib
CHID: 41034 CASRN: 184475-35-2
SPID(S): ES211_34_34
M4ID: 42675972 BRK

HILL MODEL (in red):

	tp	ga	gw
val:	2.45	0.781	8
sd:	0.448	0.0778	5.92

GAIN-LOSS MODEL (in blue):

	tp	ga	gw	la	lw
val:	3.17	0.803	8	2.13	3.06
sd:	0.705	0.142	10.1	0.197	3.13

	CNST	HILL	GNLS
AIC:	181.75	156.73	151.14
PROB:	0	0.06	0.94
RMSE:	4.59	3.74	3.89

MAX_MEAN: 5.99 MAX_MED: 4.85 BMAD: 0.164

COFF: 0.491 HIT-CALL: 1 FITC: 50 ACTP: 1

FLAGS: 10

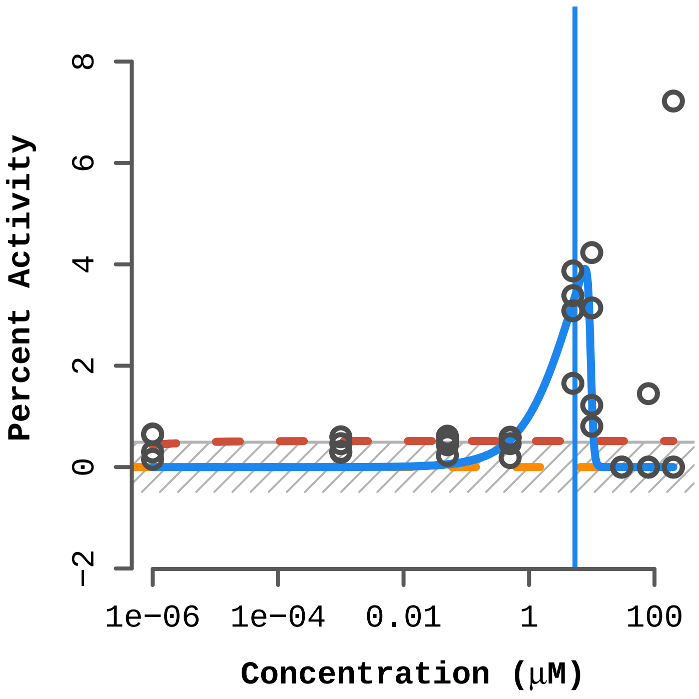

ASSAY: AEID3095 (CCTE_Deisenroth_DEVTOX_RUES2-GLR_Sox2

NAME: Sunitinib malate
CHID: 46492 CASRN: 341031-54-7
SPID(S): ES211_60_60
M4ID: 42676035

HILL MODEL (in red):

	tp	ga	gw
val:	0.514	−7	0.741
sd:	NaN	NaN	NaN

GAIN-LOSS MODEL (in blue):

	tp	ga	gw	la	lw
val:	6.74	0.733	1.03	0.983	18
sd:	NaN	NaN	NaN	NaN	NaN

	CNST	HILL	GNLS
AIC:	132.23	125.23	97.06
PROB:	0	0	1
RMSE:	1.87	1.65	1.4

MAX_MEAN: 3 MAX_MED: 3.23 BMAD: 0.164

COFF: 0.491 HIT-CALL: 1 FITC: 50 ACTP: 1

FLAGS: 10

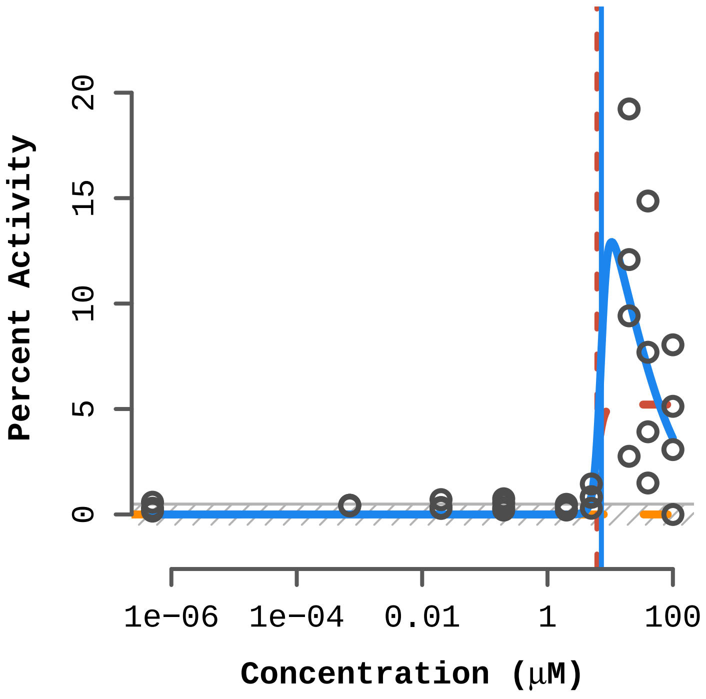

ASSAY: AEID3095 (CCTE_Deisenroth_DEVTOX_RUES2-GLR_Sox2

NAME: Vandetanib
CHID: 46681 CASRN: 443913-73-3
SPID(S): ES211_64_64
M4ID: 42676006

HILL MODEL (in red):

	tp	ga	gw
val:	5.21	0.788	7.97
sd:	1.17	0.7	62.8

GAIN-LOSS MODEL (in blue):

	tp	ga	gw	la	lw
val:	23.1	0.859	7.98	1.19	0.906
sd:	23.4	0.628	31.2	0.9	0.449

	CNST	HILL	GNLS
AIC:	203.88	175.35	169.54
PROB:	0	0.05	0.95
RMSE:	5.32	3.44	2.8

MAX_MEAN: 10.9 MAX_MED: 10.8 BMAD: 0.164

COFF: 0.491 HIT-CALL: 1 FITC: 50 ACTP: 1

FLAGS: 10

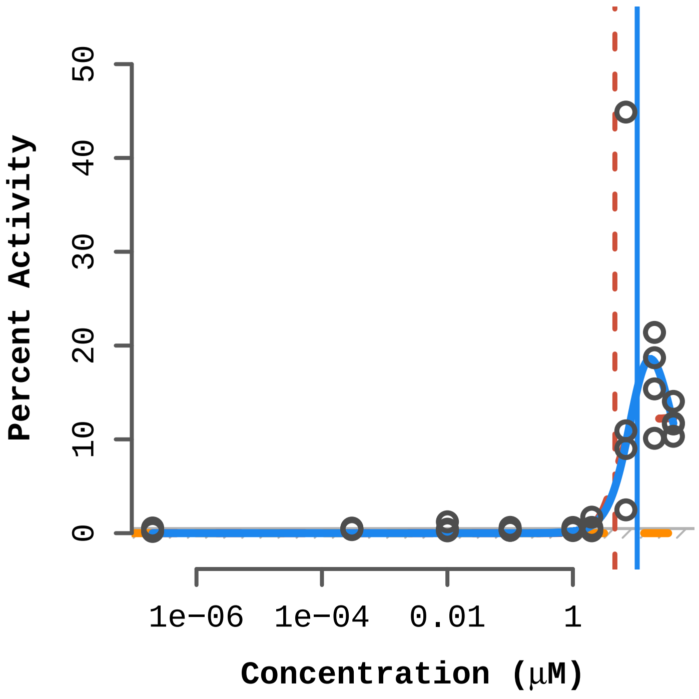

ASSAY: AEID3095 (CCTE_Deisenroth_DEVTOX_RUES2-GLR_Sox2

NAME: Dorsomorphin
CHID: 1006988 CASRN: 866405-64-3
SPID(S): ES211_6_6
M4ID: 42675990

HILL MODEL (in red):

	tp	ga	gw
val:	12.3	0.669	3.17
sd:	0.814	0.0624	0.713

GAIN-LOSS MODEL (in blue):

	tp	ga	gw	la	lw
val:	38.7	1.03	2.27	1.39	1.59
sd:	11.5	0.113	0.55	0.136	0.466

	CNST	HILL	GNLS
AIC:	261.96	174.11	165.95
PROB:	0	0.02	0.98
RMSE:	10.48	6.36	6.23

MAX_MEAN: 16.8 MAX_MED: 17 BMAD: 0.164

COFF: 0.491 HIT-CALL: 1 FITC: 50 ACTP: 1

FLAGS: 10

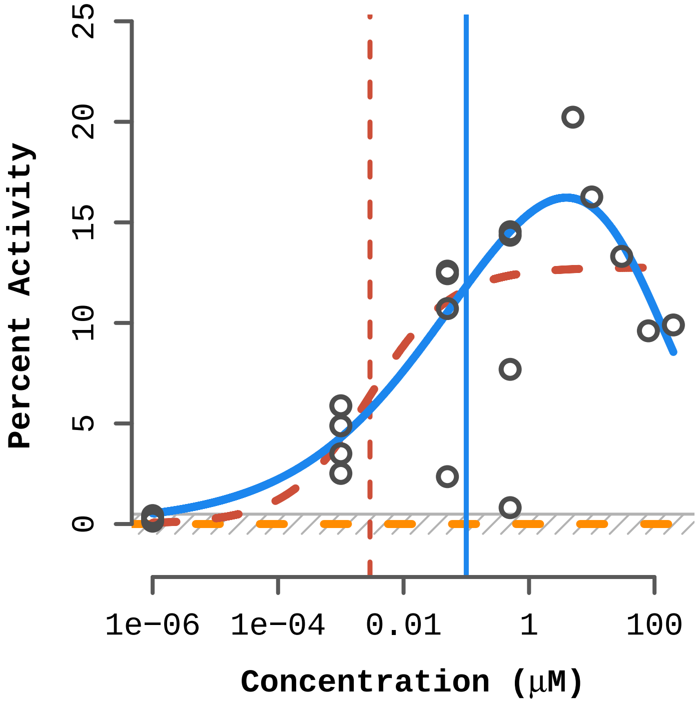

ASSAY: AEID3095 (CCTE_Deisenroth_DEVTOX_RUES2-GLR_Sox[illegible]

NAME: Actinomycin D
CHID: 20031 CASRN: 50−76−0
SPID(S): ES211_11_11
M4ID: 42676037

HILL MODEL (in red):

	tp	ga	gw
val:	12.8	−2.54	0.667
sd:	1.53	0.38	0.342

GAIN−LOSS MODEL (in blue):

	tp	ga	gw	la	lw
val:	24	−1	0.33	1.98	0.622
sd:	19.4	2.19	0.158	0.844	0.356

	CNST	HILL	GNLS
AIC:	159.74	121.87	119.45
PROB:	0	0.23	0.77
RMSE:	9.83	4.02	4.02

MAX_MEAN: 20.2 MAX_MED: 20.2 BMAD: 0.164

COFF: 0.491 HIT−CALL: 1 FITC: 51 ACTP: 1

FLAGS: 10

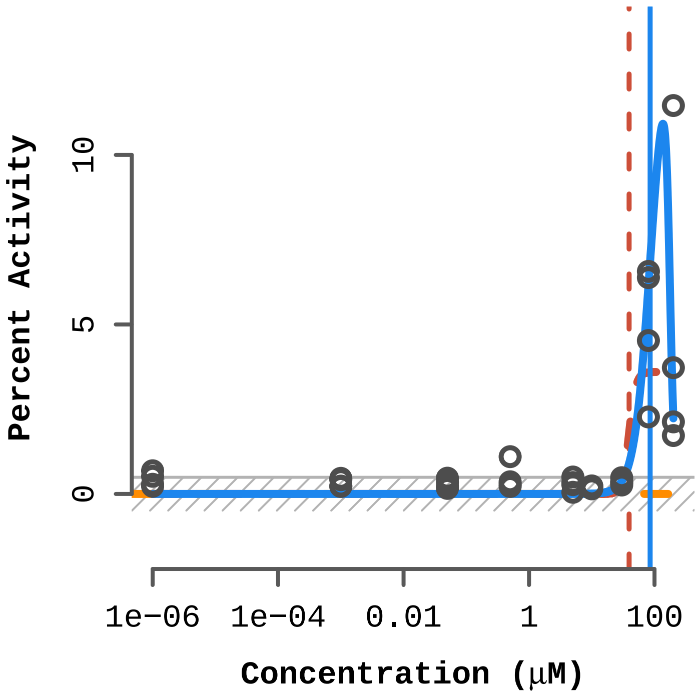

ASSAY: AEID3095 (CCTE_Deisenroth_DEVTOX_RUES2-GLR_Sox2

NAME: Mianserin hydrochloride
CHID: 944145 CASRN: 21535-47-7
SPID(S): ES211_47_47
M4ID: 42676023

HILL MODEL (in red):

	tp	ga	gw
val:	3.6	1.6	8
sd:	0.671	0.0868	5.57

GAIN-LOSS MODEL (in blue):

	tp	ga	gw	la	lw
val:	13.7	1.93	3.43	2.24	11.9
sd:	NaN	NaN	NaN	NaN	NaN

	CNST	HILL	GNLS
AIC:	147.02	108.78	99.19
PROB:	0	0.01	0.99
RMSE:	2.72	1.59	1.74

MAX_MEAN: 4.94 MAX_MED: 5.45 BMAD: 0.164

COFF: 0.491 HIT-CALL: 1 FITC: 51 ACTP: 1

FLAGS: 10
