# Supplementary material for: The DevTox Germ Layer Reporter Platform: An Assay Adaptation of the Human Pluripotent Stem Cell Test
Source: Toxics. 2022 Jul 13;10(7):392. doi: 10.3390/toxics10070392 (PMC9321663; doi:10.3390/toxics10070392)
Supplement: Supplementary file 1 [file toxics-10-00392-s001.zip › AEID3096_L6_CCTE_Deisenroth_DEVTOX_RUES2-GLR_Bra_up_210917.pdf]

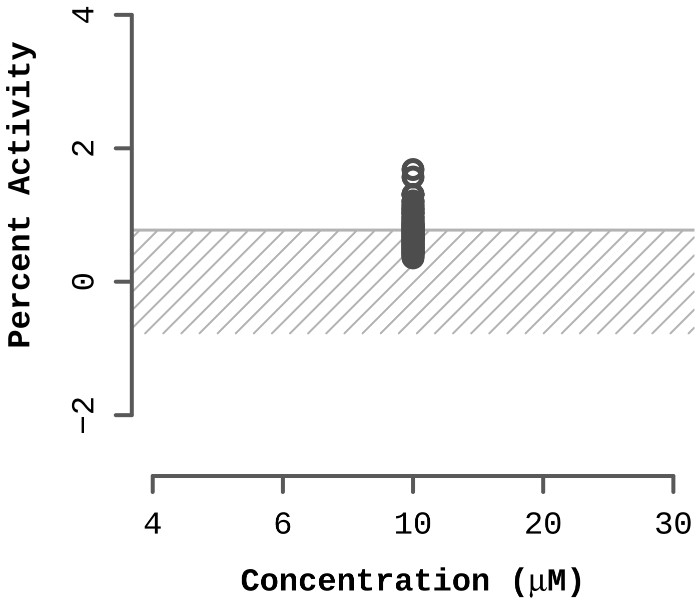

ASSAY: AEID3096 (CCTE_Deisenroth_DEVTOX_RUES2-GLR_Bra_

NAME: SB−431542
CHID: 42693 CASRN: 301836−41−9
SPID(S): ES211_56_56
M4ID: 42676087

HILL MODEL: Not applicable.

GAIN−LOSS MODEL: Not applicable.

	CNST	HILL	GNLS
AIC:	NA	NA	NA
PROB:	NA	NA	NA
RMSE:	NA	NA	NA

MAX_MEAN: 0.765 MAX_MED: 0.731 BMAD: 0.258

COFF: 0.774 HIT−CALL: −1 FITC: 2 ACTP: NA

FLAGS:

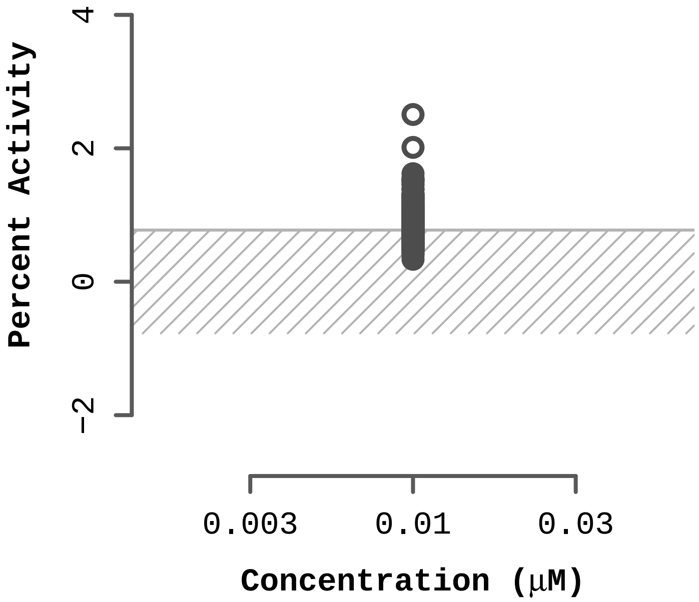

ASSAY: AEID3096 (CCTE_Deisenroth_DEVTOX_RUES2-GLR_Bra_

NAME: DMSO (spid not in DB)
CHID: NA CASRN: NA
SPID(S): DMSO
M4ID: 42676049

HILL MODEL: Not applicable.

GAIN−LOSS MODEL: Not applicable.

	CNST	HILL	GNLS
AIC:	NA	NA	NA
PROB:	NA	NA	NA
RMSE:	NA	NA	NA

MAX_MEAN: 0.884 MAX_MED: 0.828 BMAD: 0.258

COFF: 0.774 HIT−CALL: −1 FITC: 2 ACTP: NA

FLAGS:

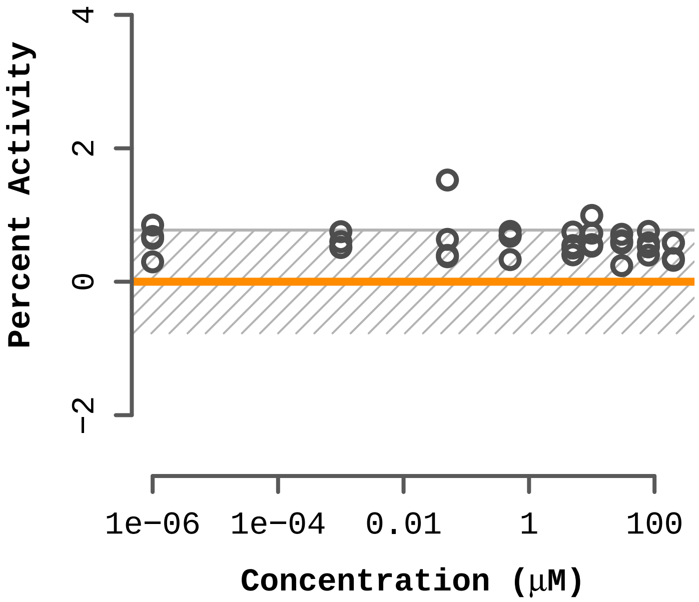

ASSAY: AEID3096 (CCTE_Deisenroth_DEVTOX_RUES2-GLR_Bra_

NAME: Hydroxyurea
CHID: 25438 CASRN: 127-07-1
SPID(S): ES211_35_35
M4ID: 42676097

HILL MODEL: Not applicable.

GAIN-LOSS MODEL: Not applicable.

	CNST	HILL	GNLS
AIC:	78.1	NA	NA
PROB:	1	NA	NA
RMSE:	0.64	NA	NA

MAX_MEAN: 0.733 MAX_MED: 0.69 BMAD: 0.258

COFF: 0.774 HIT-CALL: 0 FITC: 4 ACTP: 0

FLAGS:

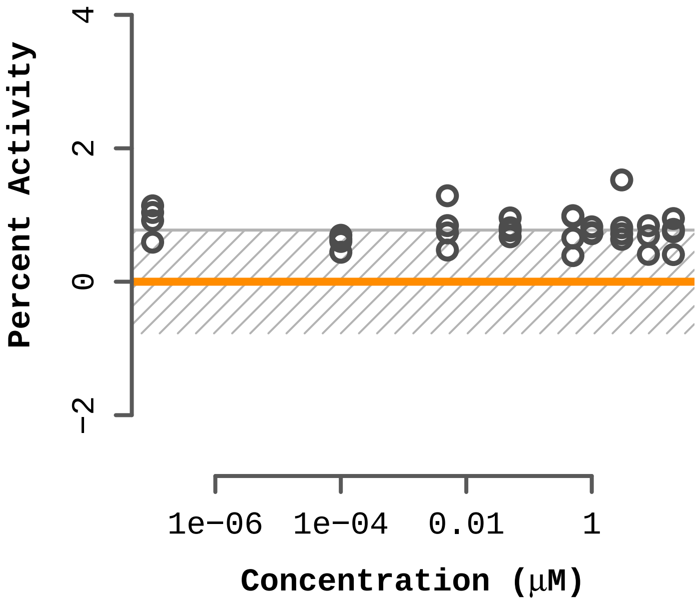

ASSAY: AEID3096 (CCTE_Deisenroth_DEVTOX_RUES2-GLR_Bra_

NAME: Dexamethasone sodium phosphate
CHID: 47429 CASRN: 2392−39−4
SPID(S): ES211_28_28
M4ID: 42676103

HILL MODEL: Failed to converge.

GAIN−LOSS MODEL: Failed to converge.

	CNST	HILL	GNLS
AIC:	93.73	NA	NA
PROB:	1	NA	NA
RMSE:	0.81	NA	NA

MAX_MEAN: 0.923 MAX_MED: 0.979 BMAD: 0.258

COFF: 0.774 HIT−CALL: 0 FITC: 7 ACTP: 0

FLAGS: 8; 10

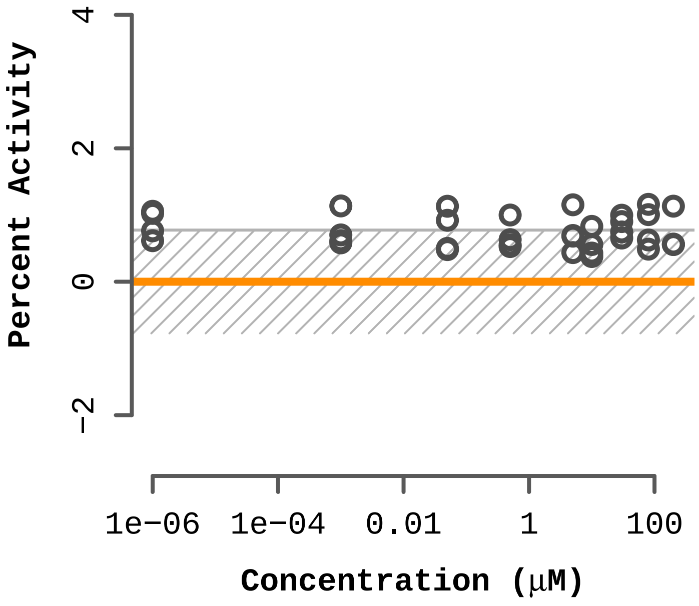

ASSAY: AEID3096 (CCTE_Deisenroth_DEVTOX_RUES2-GLR_Bra_

NAME: Pomalidomide
CHID: 893458 CASRN: 19171-19-8
SPID(S): ES211_50_50
M4ID: 42676070

HILL MODEL: Failed to converge.

GAIN-LOSS MODEL: Failed to converge.

	CNST	HILL	GNLS
AIC:	93.67	NA	NA
PROB:	1	NA	NA
RMSE:	0.78	NA	NA

MAX_MEAN: 0.865 MAX_MED: 0.894 BMAD: 0.258

COFF: 0.774 HIT-CALL: 0 FITC: 7 ACTP: 0

FLAGS: 8; 10

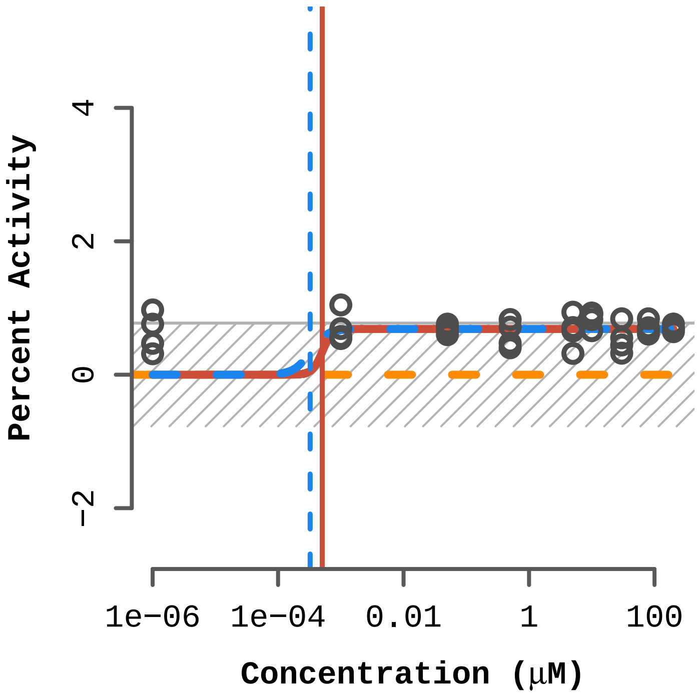

ASSAY: AEID3096 (CCTE_Deisenroth_DEVTOX_RUES2-GLR_Bra_

NAME: Acetaminophen
CHID: 20006 CASRN: 103-90-2
SPID(S): ES211_9_9
M4ID: 42676089

HILL MODEL (in red):

	tp	ga	gw
val:	0.686	-3.3	5.49
sd:	0.0371	4.62	85.8

GAIN-LOSS MODEL (in blue):

	tp	ga	gw	la	lw
val:	0.686	-3.49	3.33	3.92	4.88
sd:	NaN	NaN	NaN	NaN	NaN

	CNST	HILL	GNLS
AIC:	85.96	9.71	13.71
PROB:	0	0.88	0.12
RMSE:	0.7	0.28	0.28

MAX_MEAN: 0.829 MAX_MED: 0.865 BMAD: 0.258

COFF: 0.774 HIT-CALL: 0 FITC: 20 ACTP: 1

FLAGS: 12

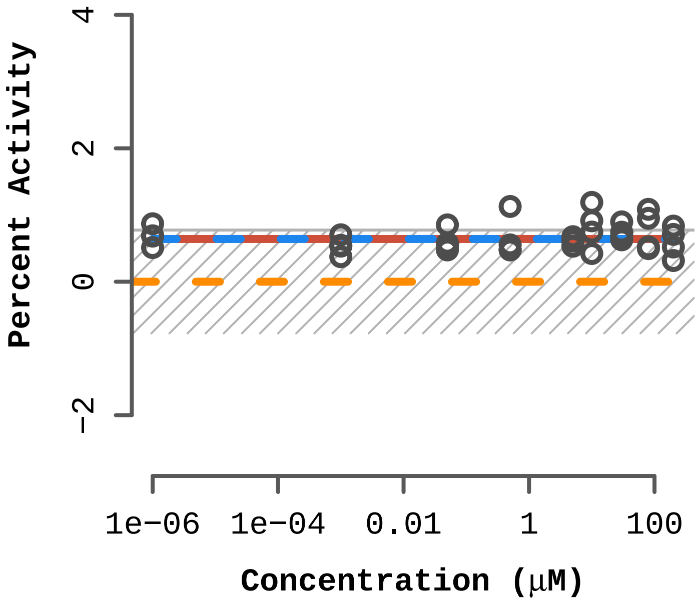

ASSAY: AEID3096 (CCTE_Deisenroth_DEVTOX_RUES2-GLR_Bra_

NAME: Boric acid (H3BO3)
CHID: 20194 CASRN: 10043-35-3
SPID(S): ES211_17_17
M4ID: 42676065

HILL MODEL (in red):

	tp	ga	gw
val:	0.641	-7	7.02
sd:	0.0335	186	1200

GAIN-LOSS MODEL (in blue):

	tp	ga	gw	la	lw
val:	0.641	-7	7.1	4.3	18
sd:	NA	NA	NA	NA	NA

	CNST	HILL	GNLS
AIC:	85.79	-2.29	1.71
PROB:	0	0.88	0.12
RMSE:	0.7	0.21	0.21

MAX_MEAN: 0.818 MAX_MED: 0.83 BMAD: 0.258

COFF: 0.774 HIT-CALL: 0 FITC: 20 ACTP: 1

FLAGS: 12

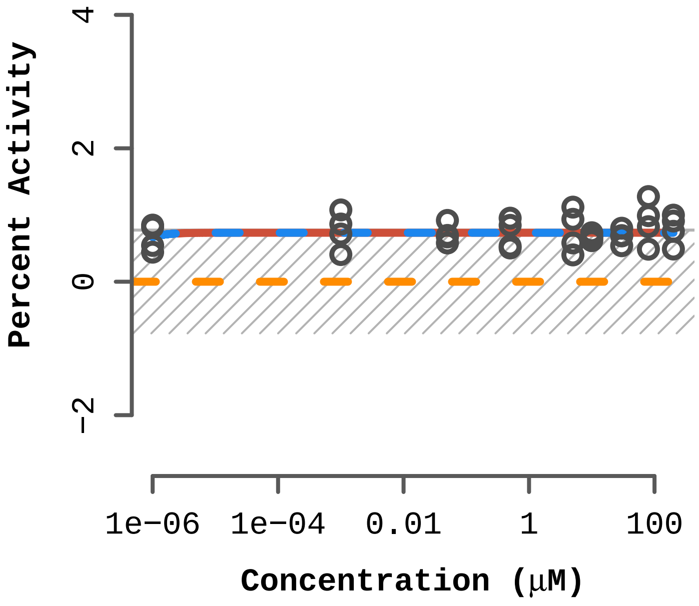

ASSAY: AEID3096 (CCTE_Deisenroth_DEVTOX_RUES2-GLR_Bra_

NAME: Ethylene glycol
CHID: 20597 CASRN: 107-21-1
SPID(S): ES211_32_32
M4ID: 42676050

HILL MODEL (in red):

	tp	ga	gw
val:	0.734	-6.47	2.14
sd:	0.0387	8.22	37.5

GAIN-LOSS MODEL (in blue):

	tp	ga	gw	la	lw
val:	0.734	-6.48	2.09	2.92	17.6
sd:	0.0387	8.77	38.2	5270	131000

	CNST	HILL	GNLS
AIC:	92.68	0.29	4.29
PROB:	0	0.88	0.12
RMSE:	0.77	0.21	0.21

MAX_MEAN: 0.895 MAX_MED: 0.908 BMAD: 0.258

COFF: 0.774 HIT-CALL: 0 FITC: 20 ACTP: 1

FLAGS: 8; 12

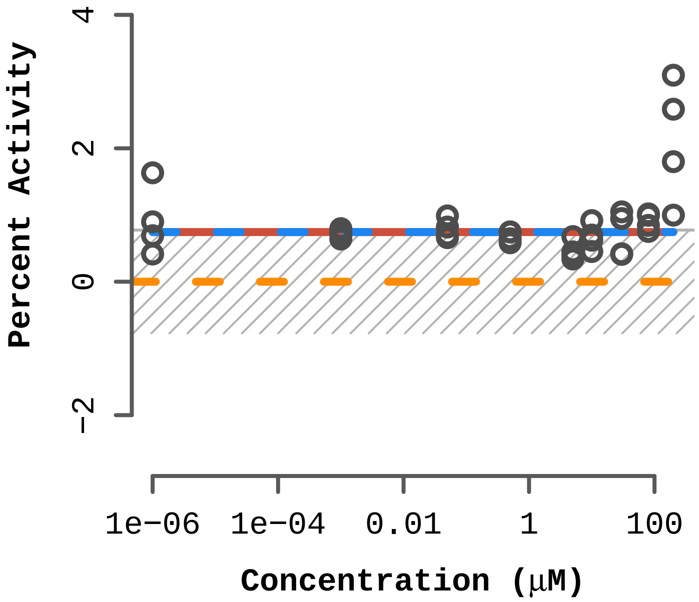

ASSAY: AEID3096 (CCTE_Deisenroth_DEVTOX_RUES2-GLR_Bra_

NAME: Busulfan
CHID: 20910 CASRN: 55-98-1
SPID(S): ES211_18_18
M4ID: 42676080

HILL MODEL (in red):

	tp	ga	gw
val:	0.745	-7	6.59
sd:	0.0493	262	1650

GAIN-LOSS MODEL (in blue):

	tp	ga	gw	la	lw
val:	0.745	-7	6.5	2.9	18
sd:	NaN	NaN	NaN	NaN	NaN

	CNST	HILL	GNLS
AIC:	108.43	46.22	50.22
PROB:	0	0.88	0.12
RMSE:	1.05	0.58	0.58

MAX_MEAN: 2.12 MAX_MED: 2.19 BMAD: 0.258

COFF: 0.774 HIT-CALL: 0 FITC: 20 ACTP: 1

FLAGS: 8; 12

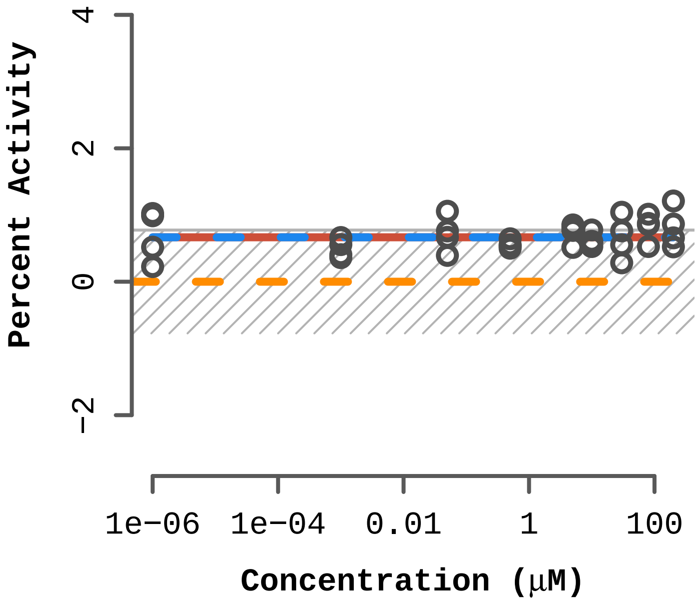

ASSAY: AEID3096 (CCTE_Deisenroth_DEVTOX_RUES2-GLR_Bra_

NAME: Nicotinic acid
CHID: 20932 CASRN: 59-67-6
SPID(S): ES211_48_48
M4ID: 42676105

HILL MODEL (in red):

	tp	ga	gw
val:	0.666	-7	7.14
sd:	0.0401	341	2200

GAIN-LOSS MODEL (in blue):

	tp	ga	gw	la	lw
val:	0.666	-7	6.91	4	4.97
sd:	0.0401	299	1930	2130	6200

	CNST	HILL	GNLS
AIC:	87.47	7.87	11.87
PROB:	0	0.88	0.12
RMSE:	0.72	0.23	0.23

MAX_MEAN: 0.818 MAX_MED: 0.865 BMAD: 0.258

COFF: 0.774 HIT-CALL: 0 FITC: 20 ACTP: 1

FLAGS: 8; 12

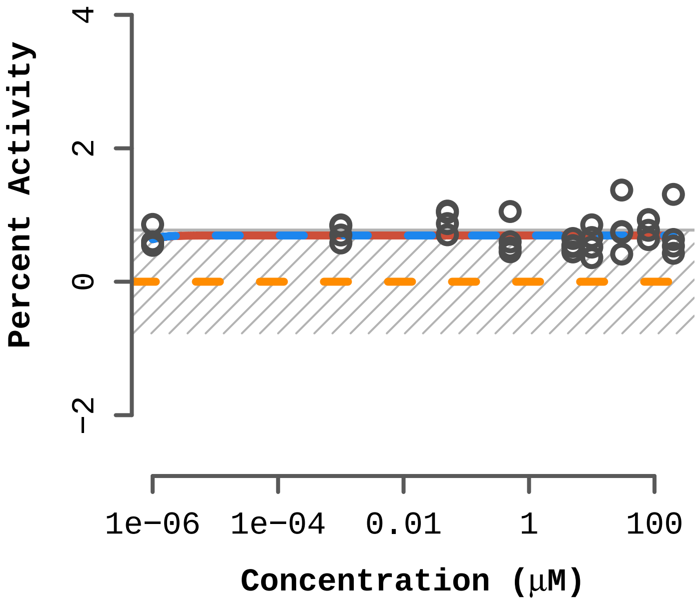

ASSAY: AEID3096 (CCTE_Deisenroth_DEVTOX_RUES2-GLR_Bra_

NAME: 1,2-Propylene glycol
CHID: 21206 CASRN: 57-55-6
SPID(S): ES211_1_1
M4ID: 42676067

HILL MODEL (in red):

	tp	ga	gw
val:	0.693	-6.44	2.37
sd:	0.0415	7.99	42.6

GAIN-LOSS MODEL (in blue):

	tp	ga	gw	la	lw
val:	0.693	-6.39	2.73	3.11	17.8
sd:	NaN	NaN	NaN	NaN	NaN

	CNST	HILL	GNLS
AIC:	91.07	6.96	10.96
PROB:	0	0.88	0.12
RMSE:	0.76	0.24	0.24

MAX_MEAN: 0.919 MAX_MED: 0.955 BMAD: 0.258

COFF: 0.774 HIT-CALL: 0 FITC: 20 ACTP: 1

FLAGS: 8; 12

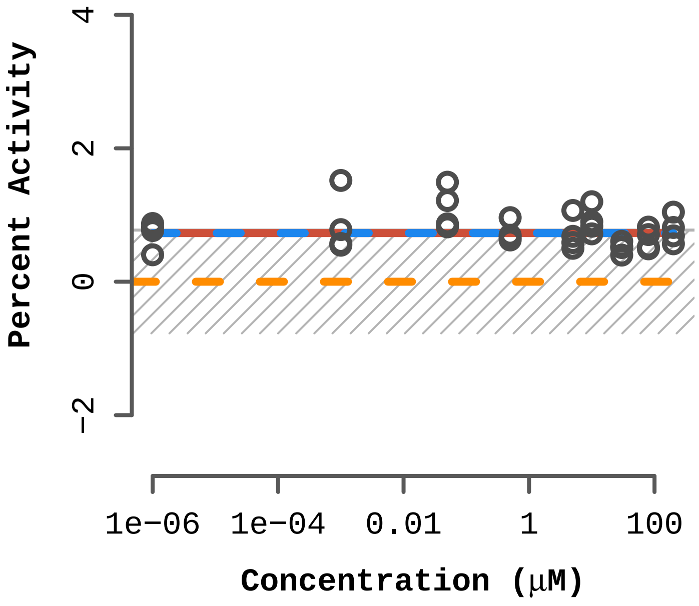

ASSAY: AEID3096 (CCTE_Deisenroth_DEVTOX_RUES2-GLR_Bra_

NAME: Saccharin
CHID: 21251 CASRN: 81-07-2
SPID(S): ES211_54_54
M4ID: 42676094

HILL MODEL (in red):

	tp	ga	gw
val:	0.729	-6.97	7.42
sd:	0.0396	305	1930

GAIN-LOSS MODEL (in blue):

	tp	ga	gw	la	lw
val:	0.729	-7	6.62	3.59	7.12
sd:	0.0396	234	1470	5020	27900

	CNST	HILL	GNLS
AIC:	96.42	12.96	16.96
PROB:	0	0.88	0.12
RMSE:	0.82	0.27	0.27

MAX_MEAN: 1.1 MAX_MED: 1.04 BMAD: 0.258

COFF: 0.774 HIT-CALL: 0 FITC: 20 ACTP: 1

FLAGS: 8; 12

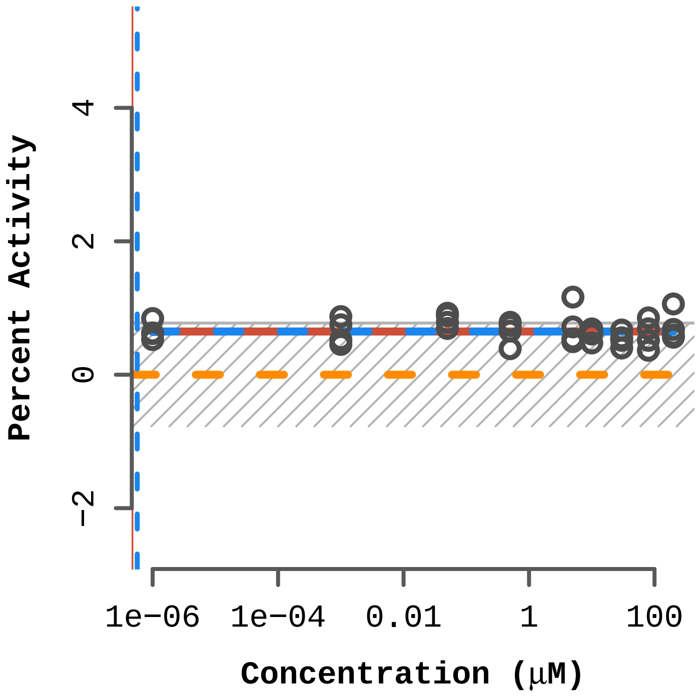

ASSAY: AEID3096 (CCTE_Deisenroth_DEVTOX_RUES2-GLR_Bra_

NAME: Dimethyl phthalate
CHID: 22455 CASRN: 131-11-3
SPID(S): ES211_30_30
M4ID: 42676098

HILL MODEL (in red):

	tp	ga	gw
val:	0.648	-6.35	5.65
sd:	0.0324	0.873	15.1

GAIN-LOSS MODEL (in blue):

	tp	ga	gw	la	lw
val:	0.648	-6.25	8	4.3	17
sd:	NA	NA	NA	NA	NA

	CNST	HILL	GNLS
AIC:	84.67	-13.08	-9.08
PROB:	0	0.88	0.12
RMSE:	0.69	0.18	0.18

MAX_MEAN: 0.821 MAX_MED: 0.835 BMAD: 0.258

COFF: 0.774 HIT-CALL: 0 FITC: 20 ACTP: 1

FLAGS: 12

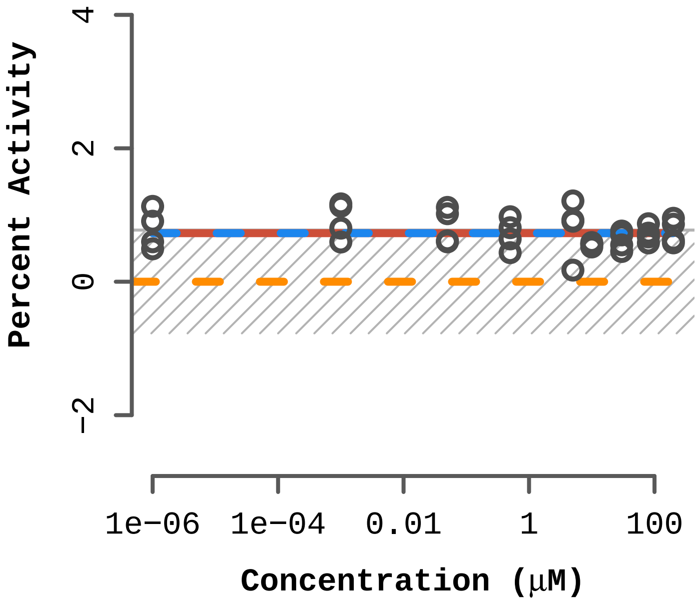

ASSAY: AEID3096 (CCTE_Deisenroth_DEVTOX_RUES2-GLR_Bra_

NAME: Thalidomide
CHID: 22524 CASRN: 50-35-1
SPID(S): ES211_62_62
M4ID: 42676106

HILL MODEL (in red):

	tp	ga	gw
val:	0.728	-7	6.92
sd:	0.0416	289	1860

GAIN-LOSS MODEL (in blue):

	tp	ga	gw	la	lw
val:	0.728	-6.94	7.78	4	18
sd:	NA	NA	NA	NA	NA

	CNST	HILL	GNLS
AIC:	93.79	9.97	13.97
PROB:	0	0.88	0.12
RMSE:	0.78	0.24	0.24

MAX_MEAN: 0.925 MAX_MED: 0.968 BMAD: 0.258

COFF: 0.774 HIT-CALL: 0 FITC: 20 ACTP: 1

FLAGS: 8; 12

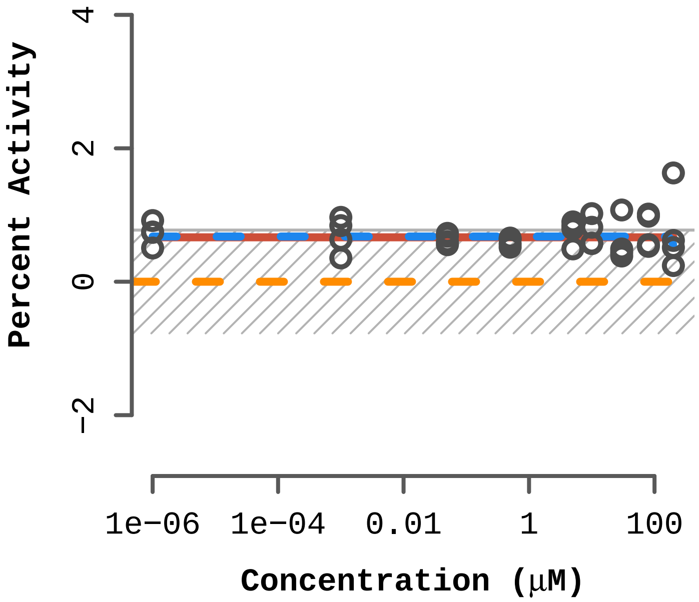

ASSAY: AEID3096 (CCTE_Deisenroth_DEVTOX_RUES2-GLR_Bra_

NAME: Valproic acid
CHID: 23733 CASRN: 99-66-1
SPID(S): ES211_63_63
M4ID: 42676056

HILL MODEL (in red):

	tp	ga	gw
val:	0.666	−7	7.4
sd:	0.0382	225	1420

GAIN-LOSS MODEL (in blue):

	tp	ga	gw	la	lw
val:	0.678	−6.99	8	2.34	15.9
sd:	0.0392	465	2120	0.0417	19.6

	CNST	HILL	GNLS
AIC:	89.32	8.3	11.01
PROB:	0	0.8	0.2
RMSE:	0.74	0.26	0.27

MAX_MEAN: 0.771 MAX_MED: 0.815 BMAD: 0.258

COFF: 0.774 HIT-CALL: 0 FITC: 20 ACTP: 1

FLAGS: 12

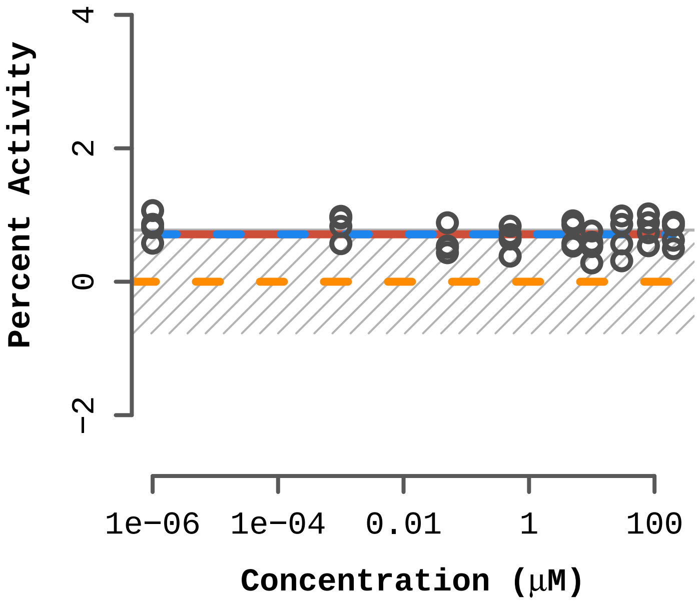

ASSAY: AEID3096 (CCTE_Deisenroth_DEVTOX_RUES2-GLR_Bra_

NAME: Salicylic acid
CHID: 26368 CASRN: 69-72-7
SPID(S): ES211_55_55
M4ID: 42676043

HILL MODEL (in red):

	tp	ga	gw
val:	0.711	−7	7.39
sd:	0.0386	173	1090

GAIN-LOSS MODEL (in blue):

	tp	ga	gw	la	lw
val:	0.711	−7	7.23	3.96	4.33
sd:	0.0386	156	1010	1440	3590

	CNST	HILL	GNLS
AIC:	89.56	1.52	5.52
PROB:	0	0.88	0.12
RMSE:	0.73	0.21	0.21

MAX_MEAN: 0.835 MAX_MED: 0.894 BMAD: 0.258

COFF: 0.774 HIT-CALL: 0 FITC: 20 ACTP: 1

FLAGS: 8; 12

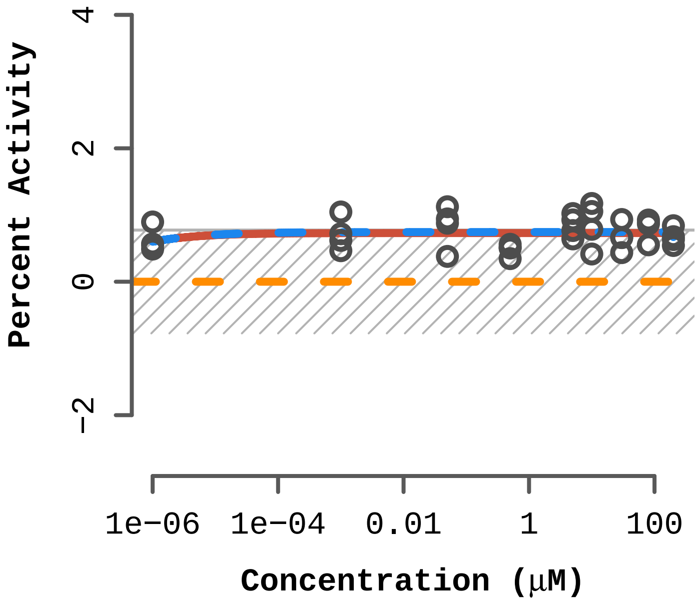

ASSAY: AEID3096 (CCTE_Deisenroth_DEVTOX_RUES2-GLR_Bra_

NAME: Penicillin G potassium
CHID: 45106 CASRN: 113-98-4
SPID(S): ES211_49_49
M4ID: 42676074

HILL MODEL (in red):

	tp	ga	gw
val:	0.73	−7	0.674
sd:	NaN	NaN	NaN

GAIN-LOSS MODEL (in blue):

	tp	ga	gw	la	lw
val:	0.745	−6.99	0.627	2.35	18
sd:	NaN	NaN	NaN	NaN	NaN

	CNST	HILL	GNLS
AIC:	91.42	5.9	9.42
PROB:	0	0.85	0.15
RMSE:	0.76	0.22	0.22

MAX_MEAN: 0.857 MAX_MED: 0.92 BMAD: 0.258

COFF: 0.774 HIT-CALL: 0 FITC: 20 ACTP: 1

FLAGS: 8; 12

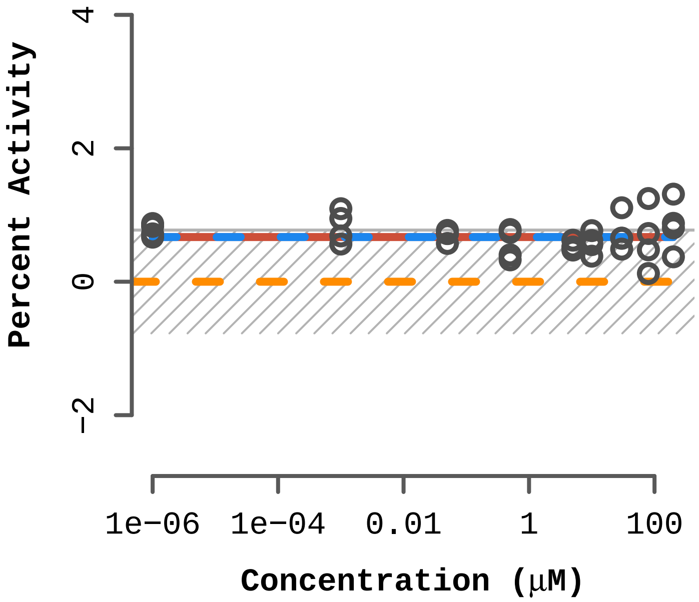

ASSAY: AEID3096 (CCTE_Deisenroth_DEVTOX_RUES2-GLR_Bra_

NAME: Lenalidomide
CHID: 46664 CASRN: 191732-72-6
SPID(S): ES211_41_41
M4ID: 42676064

HILL MODEL (in red):

	tp	ga	gw
val:	0.67	-7	7.49
sd:	0.0378	181	1120

GAIN-LOSS MODEL (in blue):

	tp	ga	gw	la	lw
val:	0.67	-7	7.53	2.94	18
sd:	NaN	NaN	NaN	NaN	NaN

	CNST	HILL	GNLS
AIC:	88.38	9.24	13.24
PROB:	0	0.88	0.12
RMSE:	0.73	0.25	0.25

MAX_MEAN: 0.843 MAX_MED: 0.842 BMAD: 0.258

COFF: 0.774 HIT-CALL: 0 FITC: 20 ACTP: 1

FLAGS: 8; 12

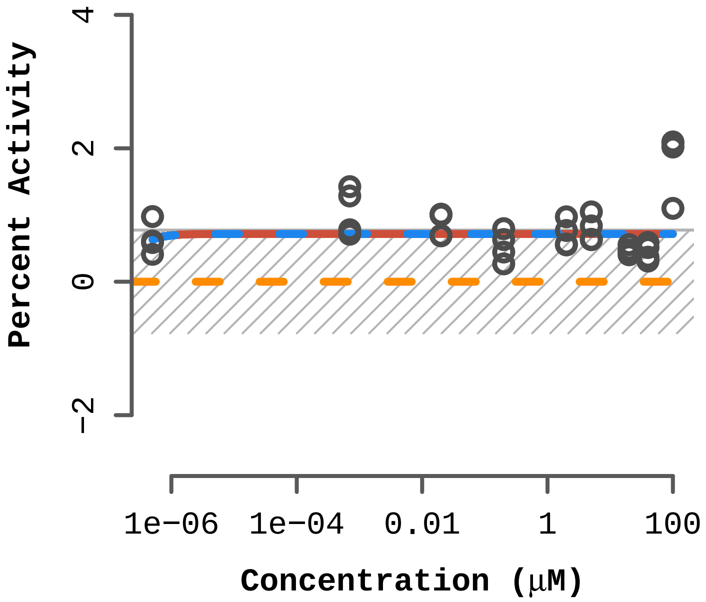

ASSAY: AEID3096 (CCTE_Deisenroth_DEVTOX_RUES2-GLR_Bra_

NAME: Ketanserin tartrate
CHID: 1003945 CASRN: 83846-83-7
SPID(S): ES211_40_40
M4ID: 42676085

HILL MODEL (in red):

	tp	ga	gw
val:	0.716	-6.71	2.15
sd:	NaN	NaN	NaN

GAIN-LOSS MODEL (in blue):

	tp	ga	gw	la	lw
val:	0.716	-6.74	1.99	2.98	11
sd:	NaN	NaN	NaN	NaN	NaN

	CNST	HILL	GNLS
AIC:	102.66	46.32	50.32
PROB:	0	0.88	0.12
RMSE:	0.93	0.47	0.47

MAX_MEAN: 1.82 MAX_MED: 2.05 BMAD: 0.258

COFF: 0.774 HIT-CALL: 0 FITC: 20 ACTP: 1

FLAGS: 8; 12

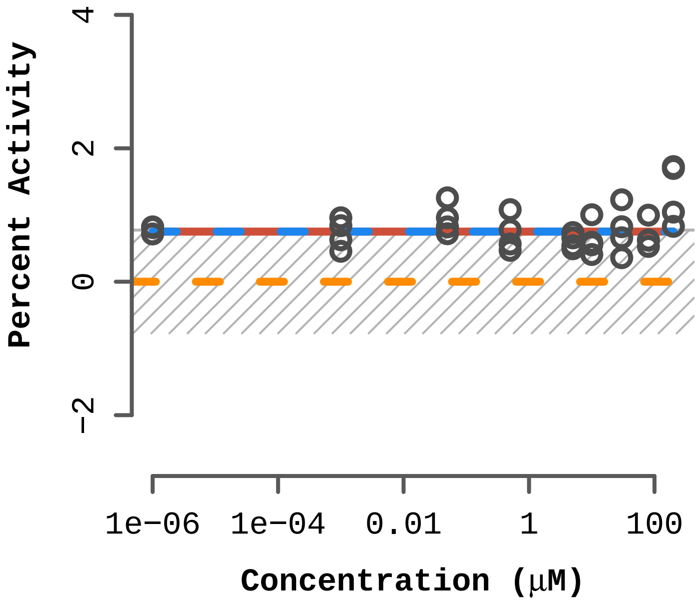

ASSAY: AEID3096 (CCTE_Deisenroth_DEVTOX_RUES2-GLR_Bra_

NAME: (2R,3S)-2-(3,4-Dihydroxyphenyl)-3,4-dihydro-2H-
CHID: 1017388 CASRN: 225937-10-0
SPID(S): ES211_22_22
M4ID: 42676068

HILL MODEL (in red):

	tp	ga	gw
val:	0.751	-7	6.94
sd:	0.0442	197	1270

GAIN-LOSS MODEL (in blue):

	tp	ga	gw	la	lw
val:	0.751	-6.96	7.95	4.29	16.6
sd:	NA	NA	NA	NA	NA

	CNST	HILL	GNLS
AIC:	99.44	21.58	25.58
PROB:	0	0.88	0.12
RMSE:	0.86	0.31	0.31

MAX_MEAN: 1.32 MAX_MED: 1.37 BMAD: 0.258

COFF: 0.774 HIT-CALL: 0 FITC: 20 ACTP: 1

FLAGS: 8; 12

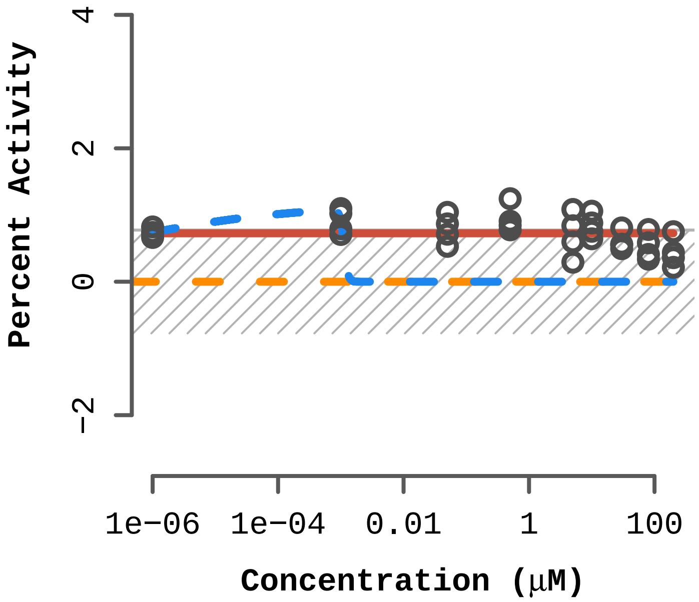

ASSAY: AEID3096 (CCTE_Deisenroth_DEVTOX_RUES2-GLR_Bra_

NAME: 5,5-Diphenylhydantoin
CHID: 20541 CASRN: 57-41-0
SPID(S): ES211_3_3
M4ID: 42676082

HILL MODEL (in red):

	tp	ga	gw
val:	0.726	-7	6.73
sd:	0.0402	279	1740

GAIN-LOSS MODEL (in blue):

	tp	ga	gw	la	lw
val:	1.16	-6.8	0.301	-2.95	14.2
sd:	NaN	NaN	NaN	NaN	NaN

	CNST	HILL	GNLS
AIC:	91.66	9.99	88.82
PROB:	0	1	0
RMSE:	0.76	0.24	0.65

MAX_MEAN: 0.943 MAX_MED: 0.909 BMAD: 0.258

COFF: 0.774 HIT-CALL: 0 FITC: 21 ACTP: 1

FLAGS: 8; 12

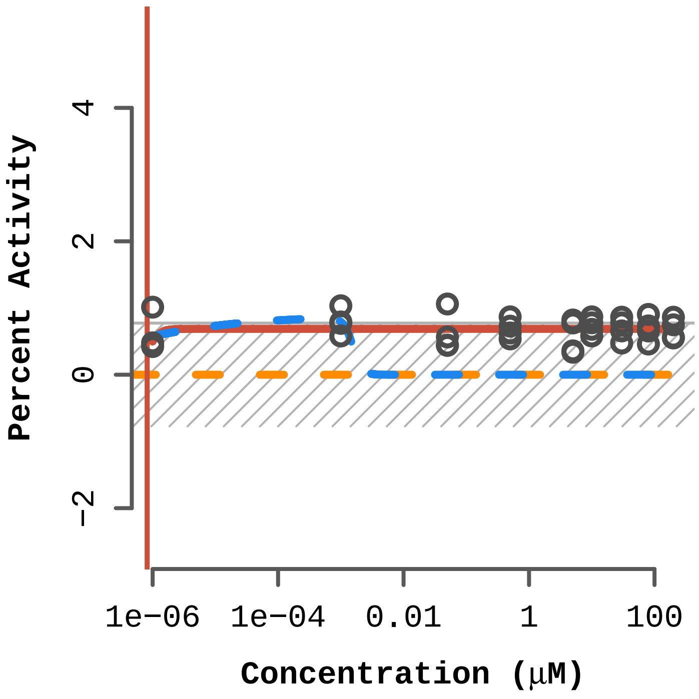

ASSAY: AEID3096 (CCTE_Deisenroth_DEVTOX_RUES2-GLR_Bra_

NAME: Isoniazid
CHID: 20755 CASRN: 54-85-3
SPID(S): ES211_39_39
M4ID: 42676055

HILL MODEL (in red):

	tp	ga	gw
val:	0.687	-6.09	4.82
sd:	0.034	0.0838	4.77

GAIN-LOSS MODEL (in blue):

	tp	ga	gw	la	lw
val:	0.876	-6.69	0.42	-2.81	5.98
sd:	NaN	NaN	NaN	NaN	NaN

	CNST	HILL	GNLS
AIC:	86.11	-7.4	86.08
PROB:	0	1	0
RMSE:	0.7	0.19	0.62

MAX_MEAN: 0.794 MAX_MED: 0.781 BMAD: 0.258

COFF: 0.774 HIT-CALL: 0 FITC: 21 ACTP: 1

FLAGS: 12

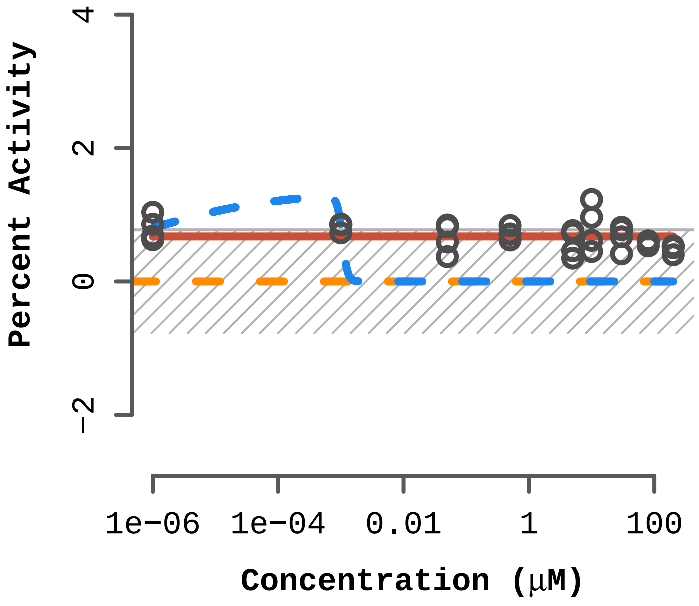

ASSAY: AEID3096 (CCTE_Deisenroth_DEVTOX_RUES2-GLR_Bra_

NAME: Methyldopa sesquihydrate
CHID: 20863 CASRN: 41372-08-1
SPID(S): ES211_46_46
M4ID: 42676079

HILL MODEL (in red):

	tp	ga	gw
val:	0.675	−6.98	7.4
sd:	0.032	142	919

GAIN-LOSS MODEL (in blue):

	tp	ga	gw	la	lw
val:	1.36	−6.42	0.38	−2.98	10.5
sd:	NaN	NaN	NaN	NaN	NaN

	CNST	HILL	GNLS
AIC:	86.93	−7.1	82.87
PROB:	0	1	0
RMSE:	0.71	0.19	0.6

MAX_MEAN: 0.823 MAX_MED: 0.851 BMAD: 0.258

COFF: 0.774 HIT-CALL: 0 FITC: 21 ACTP: 1

FLAGS: 8; 12

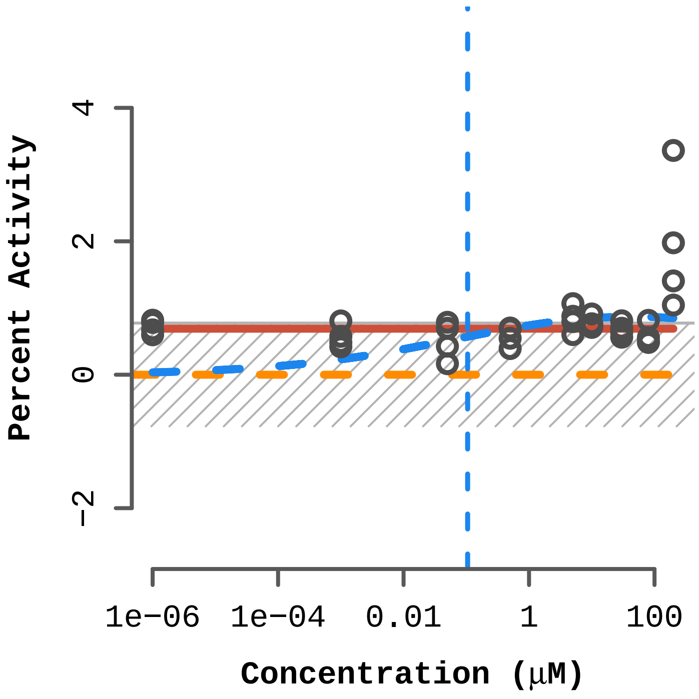


```
ASSAY:    AEID3096 (CCTE_Deisenroth_DEVTOX_RUES2-GLR_Bra_

NAME:     MEHP
CHID:     25680    CASRN: 4376-20-9
SPID(S):  ES211_44_44
M4ID:     42676069

HILL MODEL (in red):
      tp        ga        gw
val:  0.69      -7        6.72
sd:   0.0386    200       1270

GAIN-LOSS MODEL (in blue):
      tp        ga        gw        la        lw
val:  1.17      -0.979    0.3       4.3       0.3
sd:   NaN       NaN       NaN       NaN       NaN

      CNST        HILL        GNLS
AIC:  100.53      29.4        57.24
PROB: 0           1           0
RMSE: 0.96        0.54        0.56

MAX_MEAN: 1.95      MAX_MED: 1.69      BMAD: 0.258

COFF: 0.774  HIT-CALL: 0    FITC: 21    ACTP: 1

FLAGS: 8; 12
```

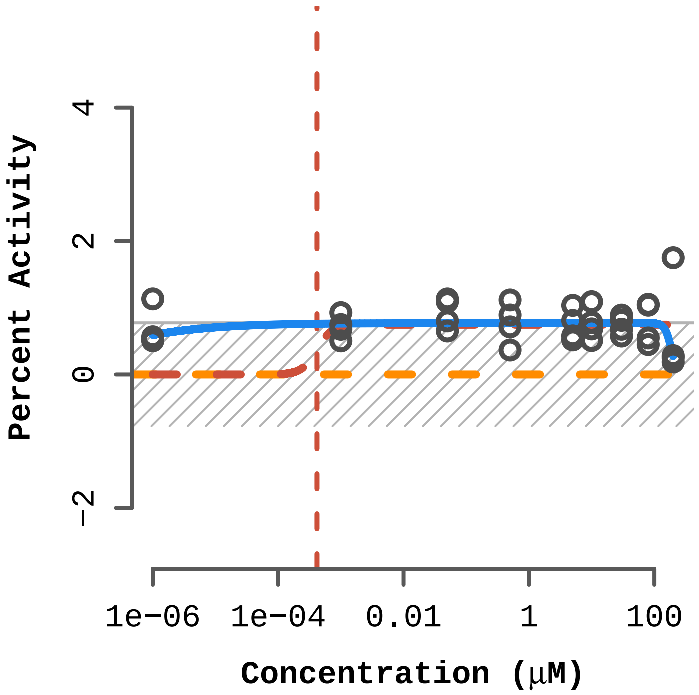

ASSAY: AEID3096 (CCTE_Deisenroth_DEVTOX_RUES2-GLR_Bra_

NAME: Sulfasalazine
CHID: 21256 CASRN: 599-79-1
SPID(S): ES211_59_59
M4ID: 42676092

HILL MODEL (in red):

	tp	ga	gw
val:	0.746	-3.38	3.55
sd:	0.0651	17.4	162

GAIN-LOSS MODEL (in blue):

	tp	ga	gw	la	lw
val:	0.77	-7	0.522	2.27	8.42
sd:	0.0571	4.75	2.66	0.0847	23.6

	CNST	HILL	GNLS
AIC:	95.11	40.09	20.18
PROB:	0	0	1
RMSE:	0.81	0.38	0.33

MAX_MEAN: 0.92 MAX_MED: 0.948 BMAD: 0.258

COFF: 0.774 HIT-CALL: 0 FITC: 31 ACTP: 1

FLAGS: 8; 12

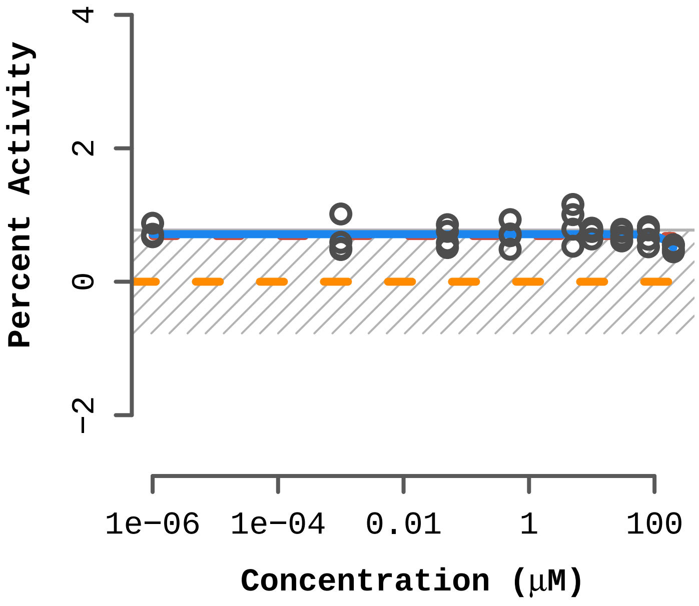

ASSAY: AEID3096 (CCTE_Deisenroth_DEVTOX_RUES2-GLR_Bra_

NAME: Cyclophosphamide monohydrate
CHID: 24888 CASRN: 6055-19-2
SPID(S): ES211_24_24
M4ID: 42676086

HILL MODEL (in red):

	tp	ga	gw
val:	0.685	-7	7.93
sd:	0.0278	327	1600

GAIN-LOSS MODEL (in blue):

	tp	ga	gw	la	lw
val:	0.712	-7	7.02	2.43	3.18
sd:	0.0288	197	1250	0.233	5.76

	CNST	HILL	GNLS
AIC:	88.09	-17.34	-21.37
PROB:	0	0.12	0.88
RMSE:	0.72	0.17	0.15

MAX_MEAN: 0.871 MAX_MED: 0.897 BMAD: 0.258

COFF: 0.774 HIT-CALL: 0 FITC: 31 ACTP: 1

FLAGS: 12

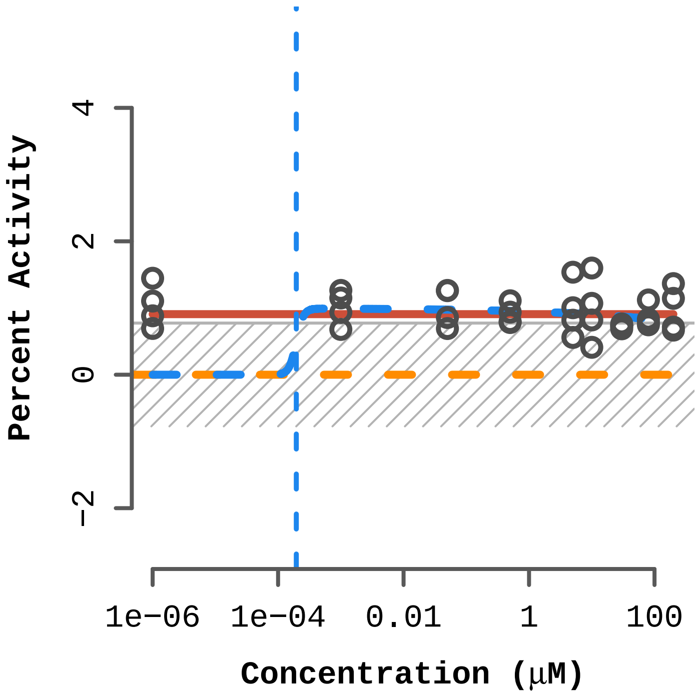

ASSAY: AEID3096 (CCTE_Deisenroth_DEVTOX_RUES2-GLR_Bra_

NAME: Acrylamide
CHID: 20027 CASRN: 79-06-1
SPID(S): ES211_10_10
M4ID: 42676052

HILL MODEL (in red):

	tp	ga	gw
val:	0.908	-6.98	7.28
sd:	0.0478	188	1230

GAIN-LOSS MODEL (in blue):

	tp	ga	gw	la	lw
val:	0.996	-3.71	7.71	4.3	0.3
sd:	0.236	76.3	829	2.88	0.634

	CNST	HILL	GNLS
AIC:	110.38	20.93	49.77
PROB:	0	1	0
RMSE:	0.98	0.28	0.44

MAX_MEAN: 1.03 MAX_MED: 1.06 BMAD: 0.258

COFF: 0.774 HIT-CALL: 1 FITC: 36 ACTP: 1

FLAGS: 11; 18

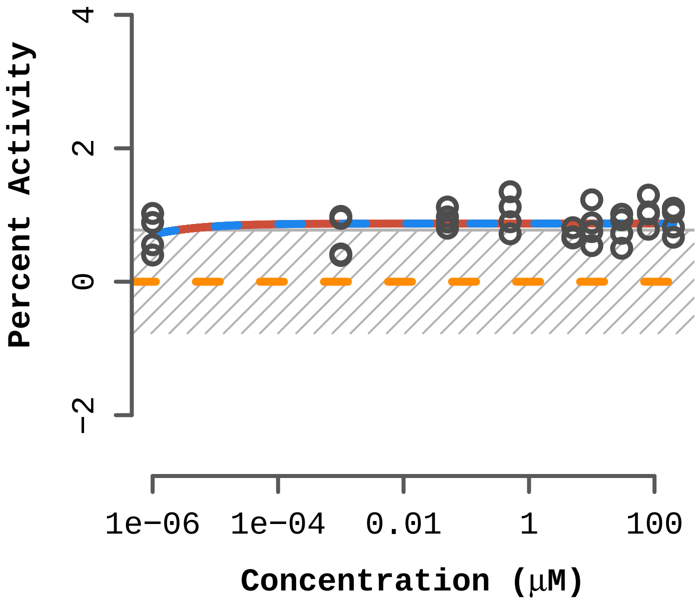

ASSAY: AEID3096 (CCTE_Deisenroth_DEVTOX_RUES2-GLR_Bra_

NAME: Aspirin
CHID: 20108 CASRN: 50-78-2
SPID(S): ES211_15_15
M4ID: 42676063

HILL MODEL (in red):

	tp	ga	gw
val:	0.872	-7	0.644
sd:	NaN	NaN	NaN

GAIN-LOSS MODEL (in blue):

	tp	ga	gw	la	lw
val:	0.872	-7	0.644	4.16	6.4
sd:	NaN	NaN	NaN	NaN	NaN

	CNST	HILL	GNLS
AIC:	102.99	10.1	14.1
PROB:	0	0.88	0.12
RMSE:	0.89	0.24	0.24

MAX_MEAN: 1.04 MAX_MED: 1.03 BMAD: 0.258

COFF: 0.774 HIT-CALL: 1 FITC: 36 ACTP: 1

FLAGS: 11; 18

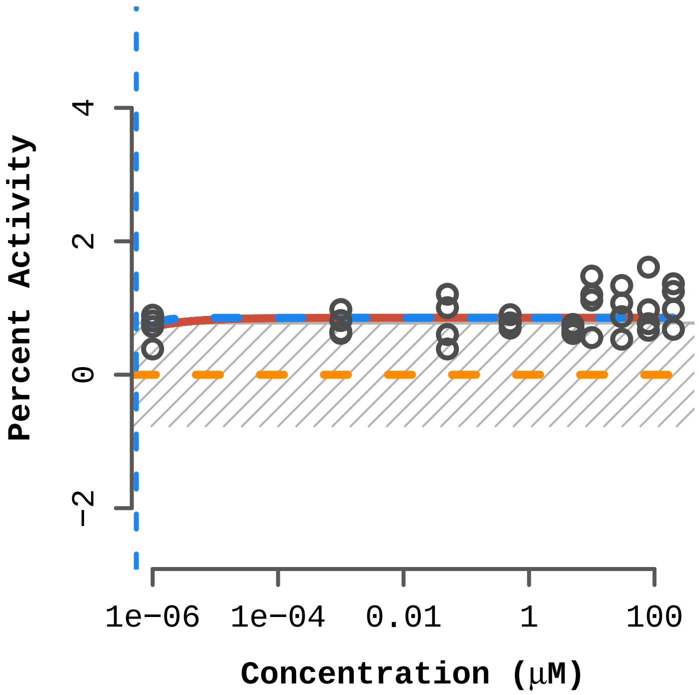

ASSAY: AEID3096 (CCTE_Deisenroth_DEVTOX_RUES2-GLR_Bra_

NAME: Caffeine
CHID: 20232 CASRN: 58-08-2
SPID(S): ES211_20_20
M4ID: 42676076

HILL MODEL (in red):

	tp	ga	gw
val:	0.854	-7	0.738
sd:	NaN	NaN	NaN

GAIN-LOSS MODEL (in blue):

	tp	ga	gw	la	lw
val:	0.853	-6.26	2.9	3.94	9.69
sd:	NA	NA	NA	NA	NA

	CNST	HILL	GNLS
AIC:	105.2	22.44	26.45
PROB:	0	0.88	0.12
RMSE:	0.92	0.29	0.29

MAX_MEAN: 1.09 MAX_MED: 1.16 BMAD: 0.258

COFF: 0.774 HIT-CALL: 1 FITC: 36 ACTP: 1

FLAGS: 11; 18

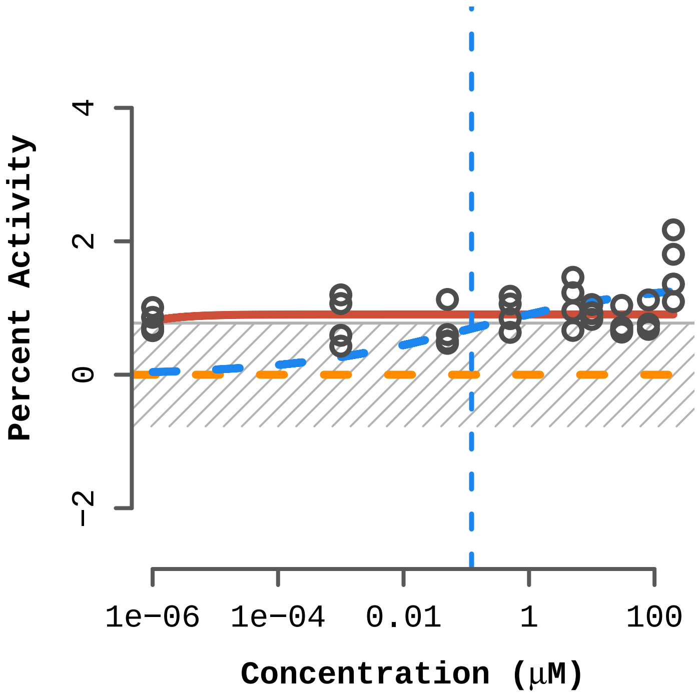

ASSAY: AEID3096 (CCTE_Deisenroth_DEVTOX_RUES2-GLR_Bra_

NAME: Diphenhydramine hydrochloride
CHID: 20537 CASRN: 147-24-0
SPID(S): ES211_31_31
M4ID: 42676100

HILL MODEL (in red):

	tp	ga	gw
val:	0.904	-7	0.918
sd:	NaN	NaN	NaN

GAIN-LOSS MODEL (in blue):

	tp	ga	gw	la	lw
val:	1.39	-0.916	0.3	3.19	9.75
sd:	NaN	NaN	NaN	NaN	NaN

	CNST	HILL	GNLS
AIC:	110.79	32.07	61.58
PROB:	0	1	0
RMSE:	1.01	0.36	0.46

MAX_MEAN: 1.61 MAX_MED: 1.58 BMAD: 0.258

COFF: 0.774 HIT-CALL: 1 FITC: 36 ACTP: 1

FLAGS: 11; 18

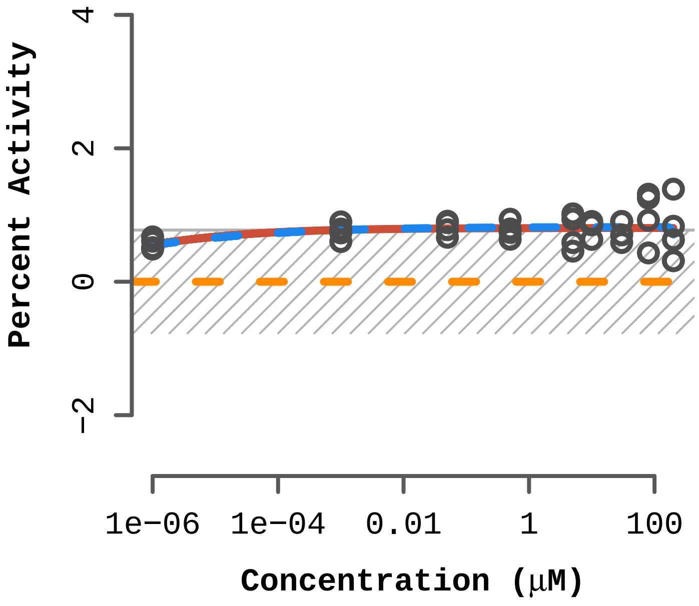

ASSAY: AEID3096 (CCTE_Deisenroth_DEVTOX_RUES2-GLR_Bra_

NAME: Acyclovir
CHID: 22556 CASRN: 59277-89-3
SPID(S): ES211_12_12
M4ID: 42676054

HILL MODEL (in red):

	tp	ga	gw
val:	0.806	−7	0.356
sd:	0.055	1.53	0.602

GAIN-LOSS MODEL (in blue):

	tp	ga	gw	la	lw
val:	0.82	−7	0.307	2.35	17.9
sd:	NaN	NaN	NaN	NaN	NaN

	CNST	HILL	GNLS
AIC:	96.69	−1.33	2.02
PROB:	0	0.84	0.16
RMSE:	0.81	0.22	0.22

MAX_MEAN: 0.981 MAX_MED: 1.09 BMAD: 0.258

COFF: 0.774 HIT-CALL: 1 FITC: 36 ACTP: 1

FLAGS: 11; 18

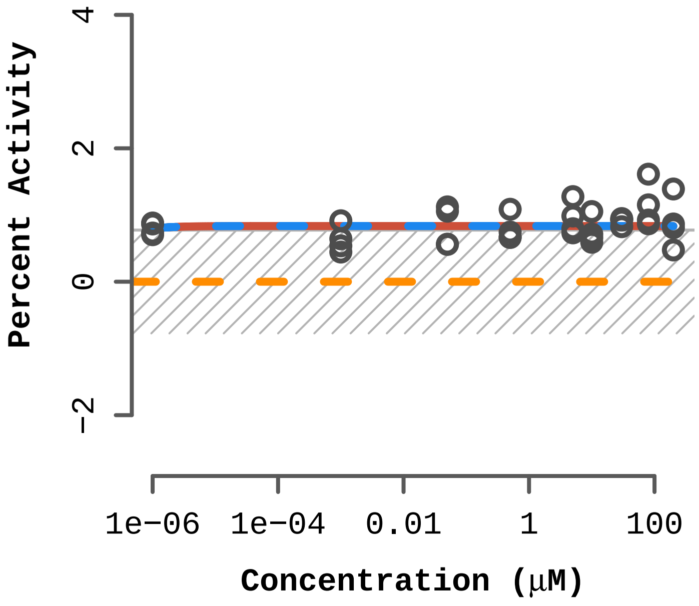

ASSAY: AEID3096 (CCTE_Deisenroth_DEVTOX_RUES2-GLR_Bra_

NAME: Carbamazepine
CHID: 22731 CASRN: 298−46−4
SPID(S): ES211_21_21
M4ID: 42676095

HILL MODEL (in red):

	tp	ga	gw
val:	0.835	−7	1.31
sd:	NaN	NaN	NaN

GAIN−LOSS MODEL (in blue):

	tp	ga	gw	la	lw
val:	0.835	−7	1.3	2.5	13
sd:	NaN	NaN	NaN	NaN	NaN

	CNST	HILL	GNLS
AIC:	103.09	10.08	14.08
PROB:	0	0.88	0.12
RMSE:	0.89	0.25	0.25

MAX_MEAN: 1.14 MAX_MED: 1.04 BMAD: 0.258

COFF: 0.774 HIT−CALL: 1 FITC: 36 ACTP: 1

FLAGS: 11; 18

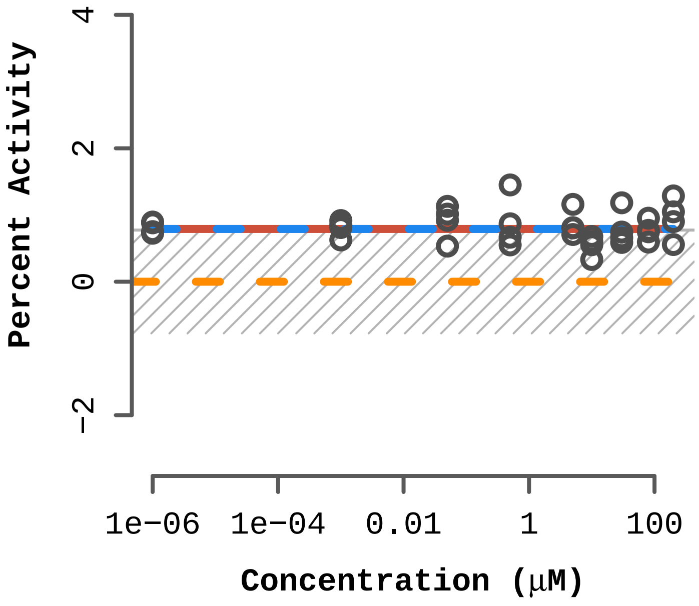

ASSAY: AEID3096 (CCTE_Deisenroth_DEVTOX_RUES2-GLR_Bra_

NAME: Warfarin
CHID: 23742 CASRN: 81-81-2
SPID(S): ES211_65_65
M4ID: 42676078

HILL MODEL (in red):

	tp	ga	gw
val:	0.791	-7	6.87
sd:	0.0368	201	1280

GAIN-LOSS MODEL (in blue):

	tp	ga	gw	la	lw
val:	0.791	-7	7.05	4.29	4.08
sd:	0.0368	227	1450	1320	2710

	CNST	HILL	GNLS
AIC:	99.59	5.22	9.22
PROB:	0	0.88	0.12
RMSE:	0.85	0.23	0.23

MAX_MEAN: 0.949 MAX_MED: 0.975 BMAD: 0.258

COFF: 0.774 HIT-CALL: 1 FITC: 36 ACTP: 1

FLAGS: 11; 18

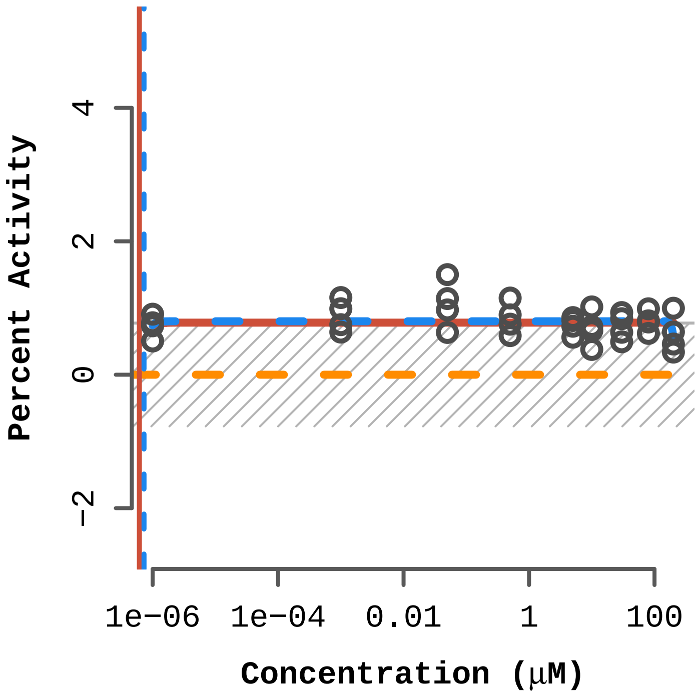

ASSAY: AEID3096 (CCTE_Deisenroth_DEVTOX_RUES2-GLR_Bra_

NAME: Stavudine
CHID: 23819 CASRN: 3056−17−5
SPID(S): ES211_58_58
M4ID: 42676047

HILL MODEL (in red):

	tp	ga	gw
val:	0.781	−6.21	6.01
sd:	NaN	NaN	NaN

GAIN-LOSS MODEL (in blue):

	tp	ga	gw	la	lw
val:	0.801	−6.14	8	2.32	18
sd:	NaN	NaN	NaN	NaN	NaN

	CNST	HILL	GNLS
AIC:	97.56	7.33	8.28
PROB:	0	0.62	0.38
RMSE:	0.82	0.24	0.23

MAX_MEAN: 1.06 MAX_MED: 1.06 BMAD: 0.258

COFF: 0.774 HIT−CALL: 1 FITC: 36 ACTP: 1

FLAGS: 11; 18

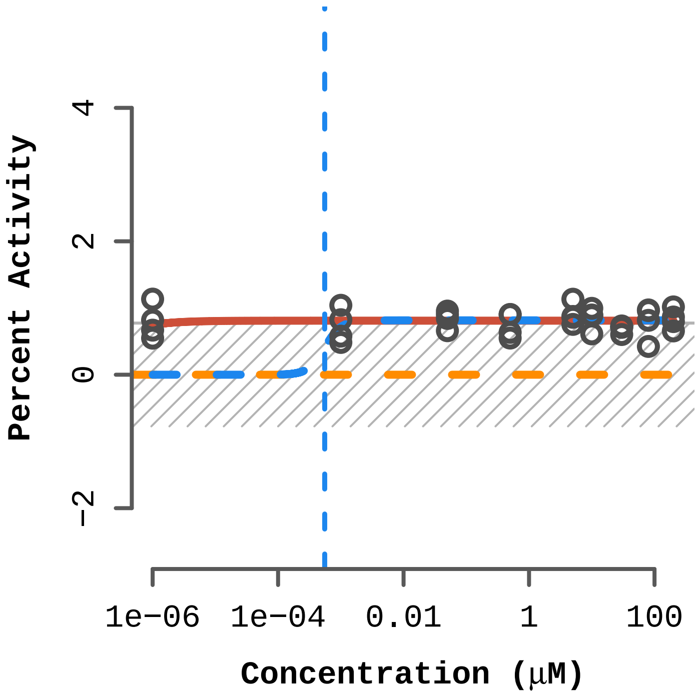

ASSAY: AEID3096 (CCTE_Deisenroth_DEVTOX_RUES2-GLR_Bra_

NAME: D-Camphor
CHID: 24721 CASRN: 464-49-3
SPID(S): ES211_27_27
M4ID: 42676088

HILL MODEL (in red):

	tp	ga	gw
val:	0.81	−7	1.08
sd:	NaN	NaN	NaN

GAIN-LOSS MODEL (in blue):

	tp	ga	gw	la	lw
val:	0.815	−3.26	3.35	3.7	5.41
sd:	0.0399	8.09	105	1170	4540

	CNST	HILL	GNLS
AIC:	97.63	−11.84	21.48
PROB:	0	1	0
RMSE:	0.82	0.18	0.32

MAX_MEAN: 0.906 MAX_MED: 0.891 BMAD: 0.258

COFF: 0.774 HIT-CALL: 1 FITC: 36 ACTP: 1

FLAGS: 11; 18

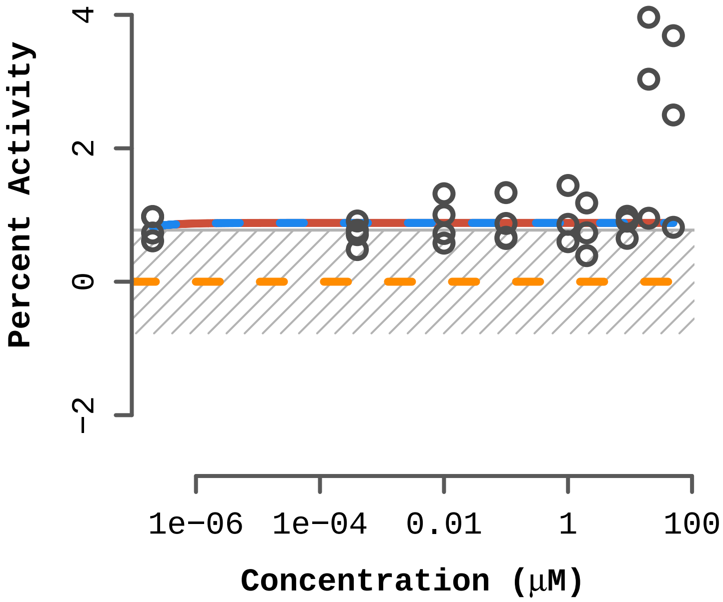

ASSAY: AEID3096 (CCTE_Deisenroth_DEVTOX_RUES2-GLR_Bra_

NAME: Ritanserin
CHID: 42594 CASRN: 87051-43-2
SPID(S): ES211_53_53
M4ID: 42676107

HILL MODEL (in red):

	tp	ga	gw
val:	0.881	-7.7	1.17
sd:	NaN	NaN	NaN

GAIN-LOSS MODEL (in blue):

	tp	ga	gw	la	lw
val:	0.881	-7.7	1.17	3.31	4.98
sd:	NaN	NaN	NaN	NaN	NaN

	CNST	HILL	GNLS
AIC:	111.82	69.44	73.44
PROB:	0	0.88	0.12
RMSE:	1.46	0.92	0.92

MAX_MEAN: 2.65 MAX_MED: 3.04 BMAD: 0.258

COFF: 0.774 HIT-CALL: 1 FITC: 36 ACTP: 1

FLAGS: 10; 11; 18

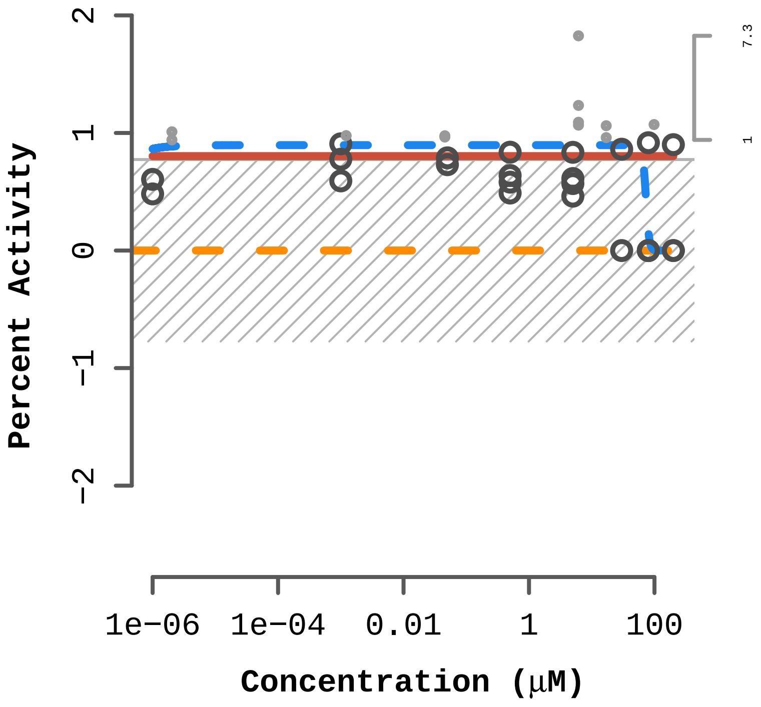

ASSAY: AEID3096 (CCTE_Deisenroth_DEVTOX_RUES2-GLR_Bra_

NAME: Tegaserod maleate
CHID: 904761 CASRN: 189188-57-6
SPID(S): ES211_61_61
M4ID: 42676045 BRK

HILL MODEL (in red):

	tp	ga	gw
val:	0.803	-7	6.11
sd:	0.106	238	1420

GAIN-LOSS MODEL (in blue):

	tp	ga	gw	la	lw
val:	0.896	-6.89	1.61	1.86	16.7
sd:	NaN	NaN	NaN	NaN	NaN

	CNST	HILL	GNLS
AIC:	125.37	95.67	95.96
PROB:	0	0.54	0.46
RMSE:	1.63	1.27	1.25

MAX_MEAN: 3.57 MAX_MED: 2.57 BMAD: 0.258

COFF: 0.774 HIT-CALL: 1 FITC: 36 ACTP: 1

FLAGS: 10; 11; 18

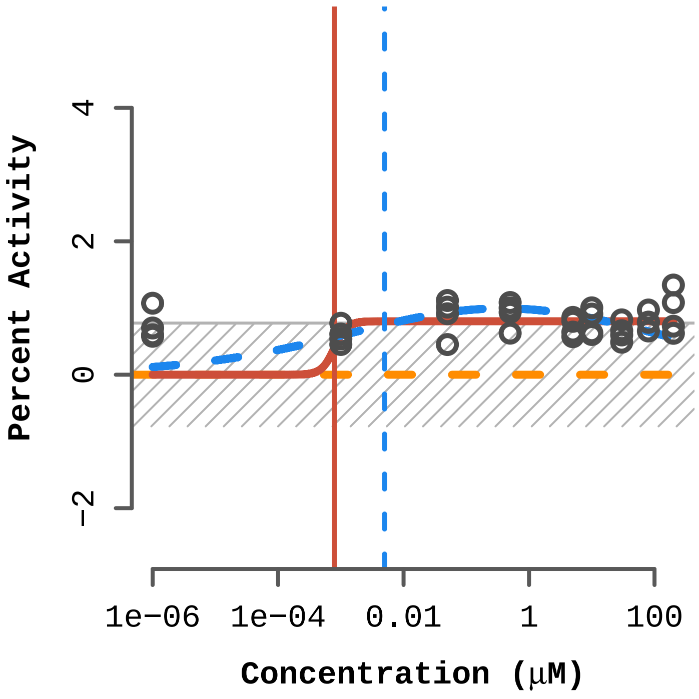


```
ASSAY:    AEID3096 (CCTE_Deisenroth_DEVTOX_RUES2-GLR_Bra_

NAME:     6-Propyl-2-thiouracil
CHID:     21209   CASRN: 51-52-5
SPID(S): ES211_8_8
M4ID:     42676073

HILL MODEL (in red):
      tp        ga        gw
val:  0.801     -3.1      4.3
sd:   0.0489    1.81      75.3

GAIN-LOSS MODEL (in blue):
      tp        ga        gw        la        lw
val:  1.61      -2.3      0.3       1.47      0.3
sd:   NaN       NaN       NaN       NaN       NaN

      CNST        HILL        GNLS
AIC:  96.29       23.62       29.15
PROB: 0           0.94        0.06
RMSE: 0.81        0.32        0.32

MAX_MEAN: 0.944    MAX_MED: 0.969    BMAD: 0.258

COFF: 0.774  HIT-CALL: 1   FITC: 37   ACTP: 1

FLAGS: 11
```

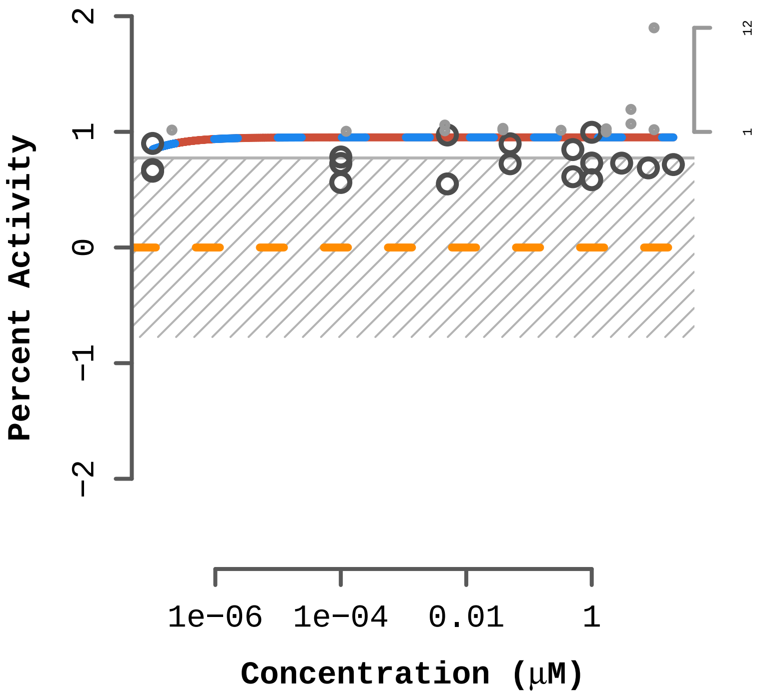

ASSAY: AEID3096 (CCTE_Deisenroth_DEVTOX_RUES2-GLR_Bra_

NAME: Cyclopamine
CHID: 43709 CASRN: 4449-51-8
SPID(S): ES211_23_23
M4ID: 42676072 BRK

HILL MODEL (in red):

	tp	ga	gw
val:	0.952	-8	0.91
sd:	NaN	NaN	NaN

GAIN-LOSS MODEL (in blue):

	tp	ga	gw	la	lw
val:	0.952	-8	0.91	3.22	17.9
sd:	NA	NA	NA	NA	NA

	CNST	HILL	GNLS
AIC:	119.61	71.14	75.14
PROB:	0	0.88	0.12
RMSE:	2.5	2.11	2.11

MAX_MEAN: 4.76 MAX_MED: 1.9 BMAD: 0.258

COFF: 0.774 HIT-CALL: 1 FITC: 40 ACTP: 1

FLAGS: 10; 18

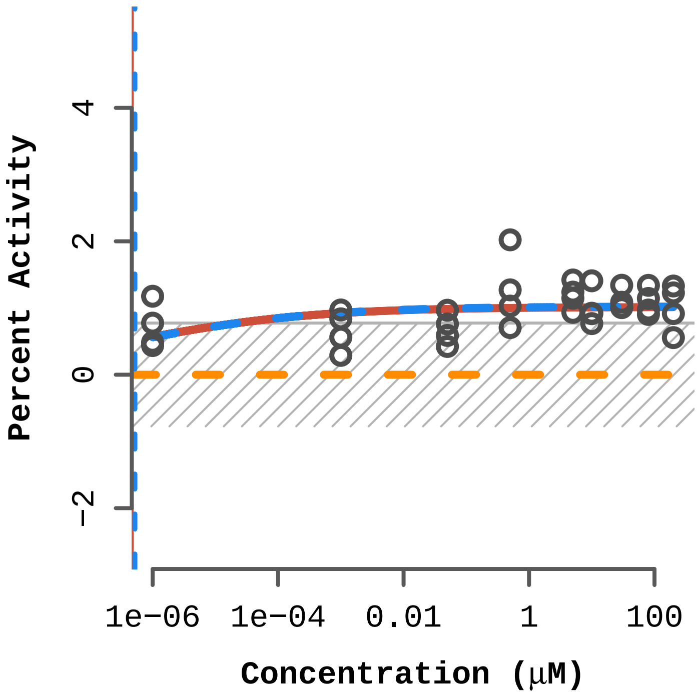

ASSAY: AEID3096 (CCTE_Deisenroth_DEVTOX_RUES2-GLR_Bra_

NAME: Methotrexate hydrate(1:x)
CHID: 1017640 CASRN: 133073-73-1
SPID(S): ES211_45_45
M4ID: 42676066

HILL MODEL (in red):

	tp	ga	gw
val:	1.02	−6.34	0.3
sd:	NaN	NaN	NaN

GAIN-LOSS MODEL (in blue):

	tp	ga	gw	la	lw
val:	1.02	−6.28	0.3	4.16	5.05
sd:	NaN	NaN	NaN	NaN	NaN

	CNST	HILL	GNLS
AIC:	113.13	28.07	32.05
PROB:	0	0.88	0.12
RMSE:	1.03	0.33	0.32

MAX_MEAN: 1.26 MAX_MED: 1.19 BMAD: 0.258

COFF: 0.774 HIT-CALL: 1 FITC: 40 ACTP: 1

FLAGS: 18

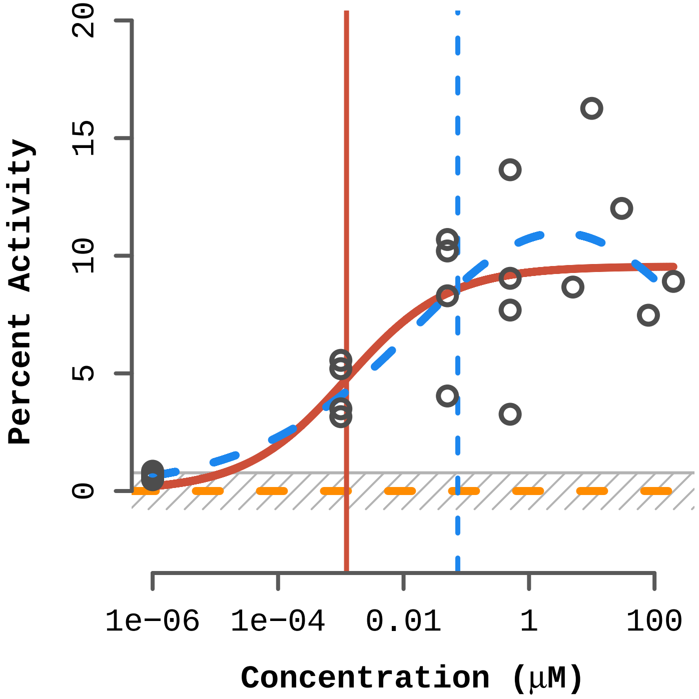

ASSAY: AEID3096 (CCTE_Deisenroth_DEVTOX_RUES2-GLR_Bra_

NAME: Actinomycin D
CHID: 20031 CASRN: 50−76−0
SPID(S): ES211_11_11
M4ID: 42676101

HILL MODEL (in red):

	tp	ga	gw
val:	9.55	−2.91	0.538
sd:	1.21	0.36	0.504

GAIN−LOSS MODEL (in blue):

	tp	ga	gw	la	lw
val:	19.2	−1.14	0.3	2.12	0.3
sd:	NaN	NaN	NaN	NaN	NaN

	CNST	HILL	GNLS
AIC:	151.5	106.5	110.95
PROB:	0	0.9	0.1
RMSE:	8	2.65	2.63

MAX_MEAN: 16.3 MAX_MED: 16.3 BMAD: 0.258

COFF: 0.774 HIT−CALL: 1 FITC: 41 ACTP: 1

FLAGS: 10

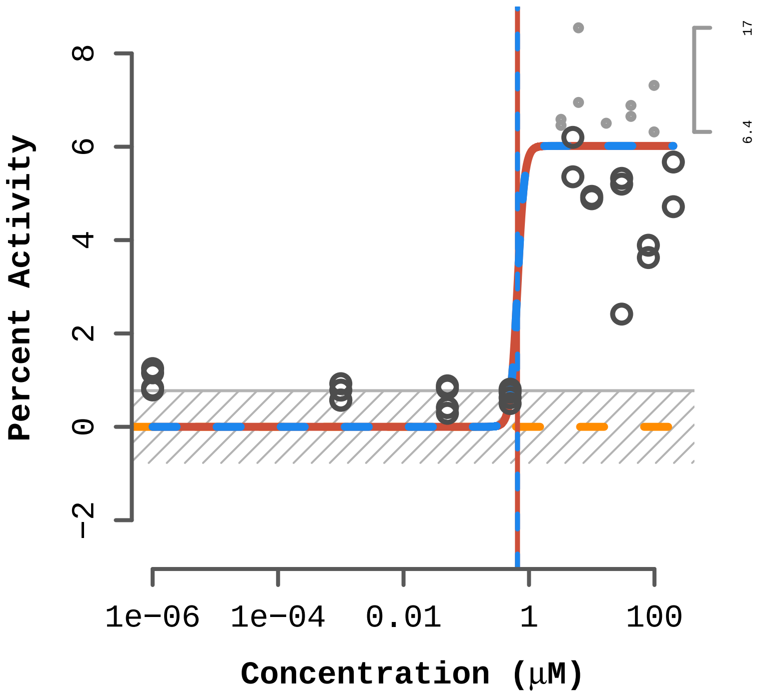

ASSAY: AEID3096 (CCTE_Deisenroth_DEVTOX_RUES2-GLR_Bra_

NAME: Diethylstilbestrol
CHID: 20465 CASRN: 56-53-1
SPID(S): ES211_29_29
M4ID: 42676042 BRK

HILL MODEL (in red):

	tp	ga	gw
val:	6.02	-0.184	7.69
sd:	0.414	4.17	273

GAIN-LOSS MODEL (in blue):

	tp	ga	gw	la	lw
val:	6.02	-0.183	7.65	4.1	5.56
sd:	0.414	4.56	296	74700	230000

	CNST	HILL	GNLS
AIC:	227.65	156.68	160.68
PROB:	0	0.88	0.12
RMSE:	5.62	2.49	2.49

MAX_MEAN: 9.15 MAX_MED: 7.2 BMAD: 0.258

COFF: 0.774 HIT-CALL: 1 FITC: 41 ACTP: 1

FLAGS: 10

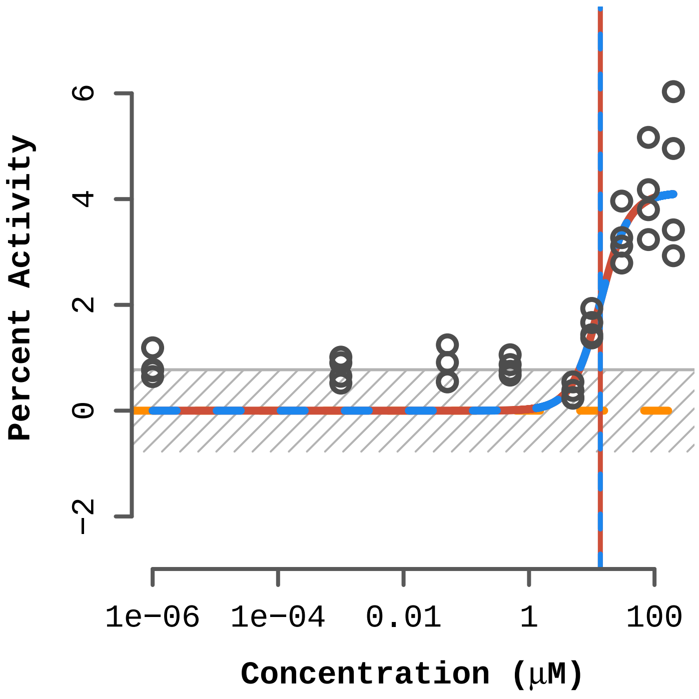

ASSAY: AEID3096 (CCTE_Deisenroth_DEVTOX_RUES2-GLR_Bra_

NAME: 5-Fluorouracil
CHID: 20634 CASRN: 51-21-8
SPID(S): ES211_5_5
M4ID: 42676057

HILL MODEL (in red):

	tp	ga	gw
val:	4.12	1.14	1.89
sd:	0.497	0.114	0.677

GAIN-LOSS MODEL (in blue):

	tp	ga	gw	la	lw
val:	4.12	1.14	1.89	3.47	6.49
sd:	0.497	0.114	0.677	1930	10700

	CNST	HILL	GNLS
AIC:	169.28	94.53	98.53
PROB:	0	0.88	0.12
RMSE:	2.45	0.76	0.76

MAX_MEAN: 4.33 MAX_MED: 4.19 BMAD: 0.258

COFF: 0.774 HIT-CALL: 1 FITC: 41 ACTP: 1

FLAGS:

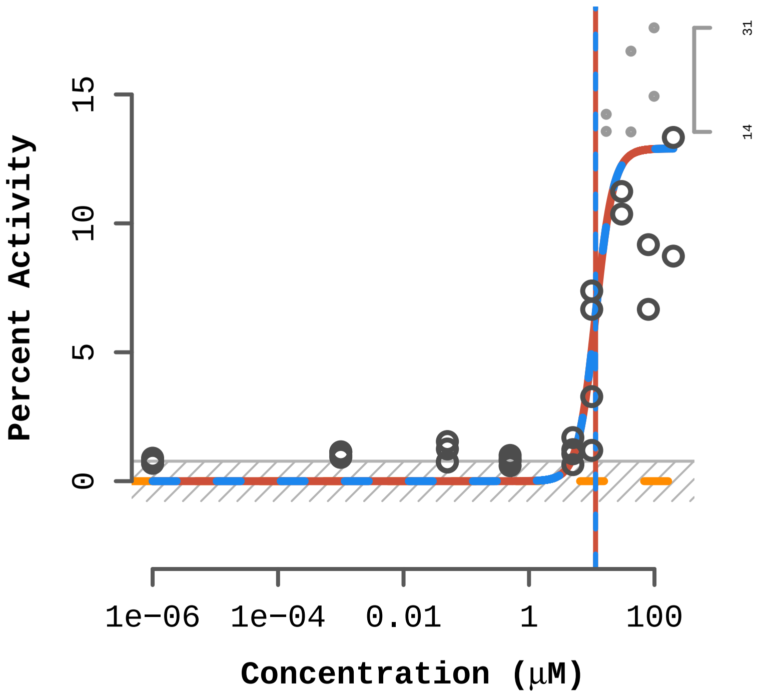

ASSAY: AEID3096 (CCTE_Deisenroth_DEVTOX_RUES2-GLR_Bra_

NAME: all-trans-Retinoic acid
CHID: 21239 CASRN: 302-79-4
SPID(S): ES211_13_13
M4ID: 42676058 BRK

HILL MODEL (in red):

	tp	ga	gw
val:	12.9	1.06	3.05
sd:	1.19	0.078	1.32

GAIN-LOSS MODEL (in blue):

	tp	ga	gw	la	lw
val:	12.9	1.06	3.05	4.21	7.71
sd:	NaN	NaN	NaN	NaN	NaN

	CNST	HILL	GNLS
AIC:	260.82	193.55	197.55
PROB:	0	0.88	0.12
RMSE:	9.79	4.38	4.38

MAX_MEAN: 18.1 MAX_MED: 16.4 BMAD: 0.258

COFF: 0.774 HIT-CALL: 1 FITC: 41 ACTP: 1

FLAGS: 10

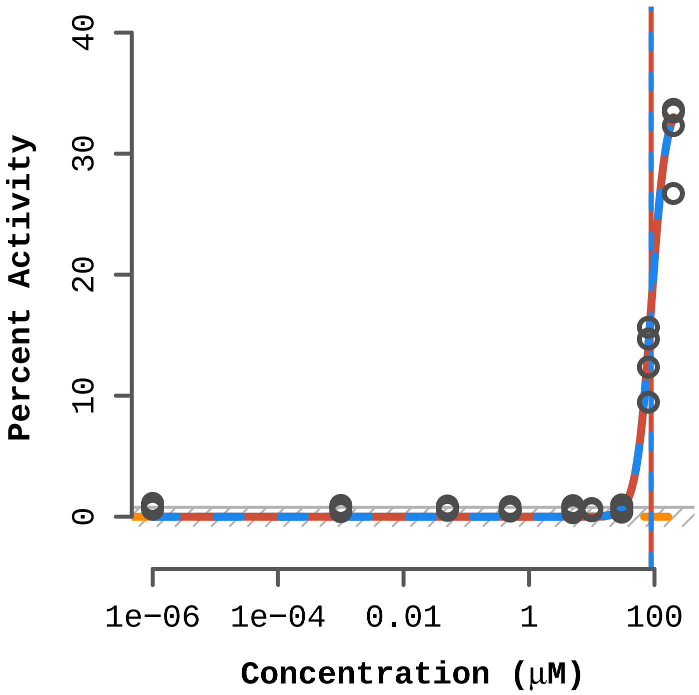

ASSAY: AEID3096 (CCTE_Deisenroth_DEVTOX_RUES2-GLR_Bra_

NAME: Rifampicin
CHID: 21244 CASRN: 13292-46-1
SPID(S): ES211_52_52
M4ID: 42676041

HILL MODEL (in red):

	tp	ga	gw
val:	34.7	1.95	3.67
sd:	1.89	0.0294	0.91

GAIN-LOSS MODEL (in blue):

	tp	ga	gw	la	lw
val:	34.7	1.95	3.67	2.89	18
sd:	1.89	0.0294	0.91	21800	589000

	CNST	HILL	GNLS
AIC:	256.11	118.41	122.41
PROB:	0	0.88	0.12
RMSE:	11.46	1.49	1.49

MAX_MEAN: 31.5 MAX_MED: 32.9 BMAD: 0.258

COFF: 0.774 HIT-CALL: 1 FITC: 41 ACTP: 1

FLAGS: 10

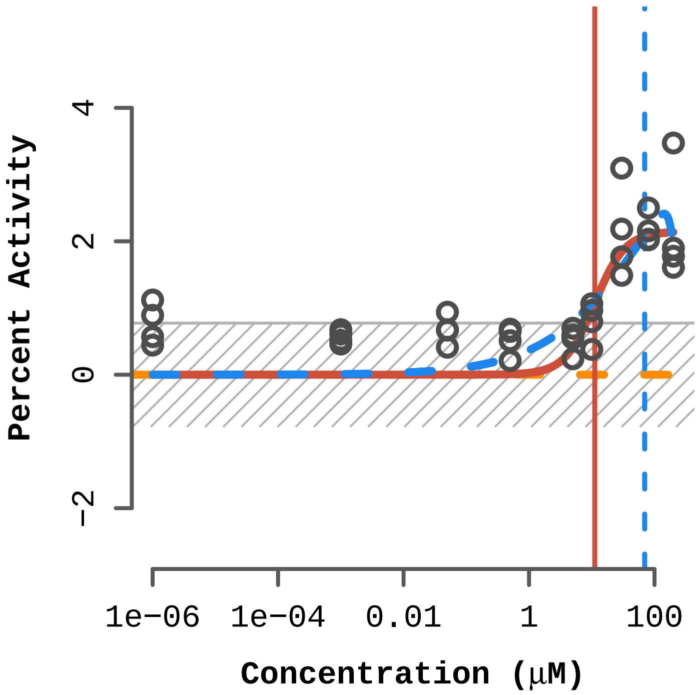

ASSAY: AEID3096 (CCTE_Deisenroth_DEVTOX_RUES2-GLR_Bra_

NAME: 5-Bromo-2'-deoxyuridine
CHID: 33105 CASRN: 59-14-3
SPID(S): ES211_4_4
M4ID: 42676104

HILL MODEL (in red):

	tp	ga	gw
val:	2.15	1.05	1.81
sd:	0.214	0.106	0.824

GAIN-LOSS MODEL (in blue):

	tp	ga	gw	la	lw
val:	4.17	1.84	0.549	2.35	7.59
sd:	2.83	1.15	0.379	0.0768	11.7

	CNST	HILL	GNLS
AIC:	130.66	72.65	74.37
PROB:	0	0.7	0.3
RMSE:	1.41	0.57	0.58

MAX_MEAN: 2.19 MAX_MED: 2.09 BMAD: 0.258

COFF: 0.774 HIT-CALL: 1 FITC: 41 ACTP: 1

FLAGS:

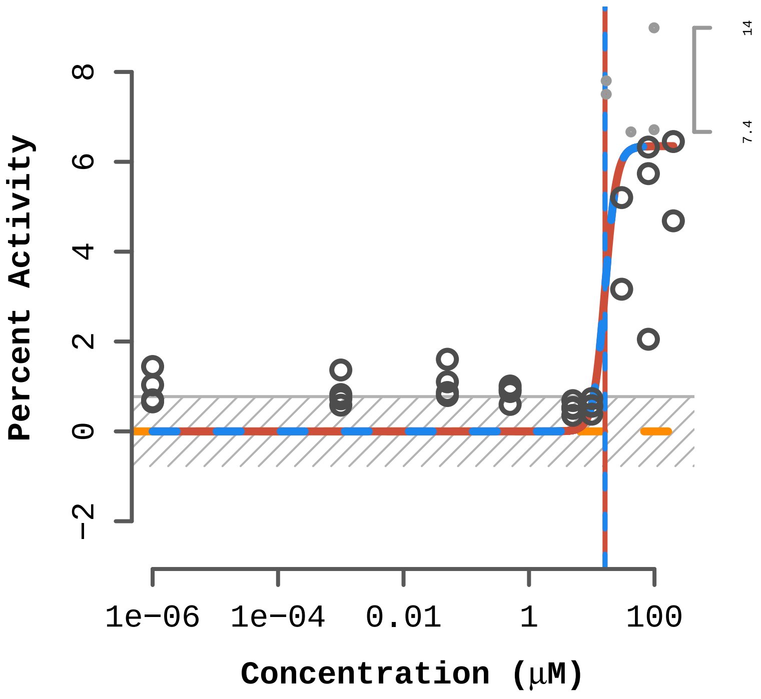

ASSAY: AEID3096 (CCTE_Deisenroth_DEVTOX_RUES2-GLR_Bra_

NAME: Imatinib mesylate
CHID: 40502 CASRN: 220127-57-1
SPID(S): ES211_37_37
M4ID: 42676053 BRK

HILL MODEL (in red):

	tp	ga	gw
val:	6.35	1.21	4.63
sd:	0.536	0.222	5.27

GAIN-LOSS MODEL (in blue):

	tp	ga	gw	la	lw
val:	6.35	1.21	4.63	3.73	5.76
sd:	0.536	0.222	5.27	1660	6700

	CNST	HILL	GNLS
AIC:	204.32	147.88	151.88
PROB:	0	0.88	0.12
RMSE:	4.48	2.03	2.03

MAX_MEAN: 8.22 MAX_MED: 7.53 BMAD: 0.258

COFF: 0.774 HIT-CALL: 1 FITC: 41 ACTP: 1

FLAGS: 10

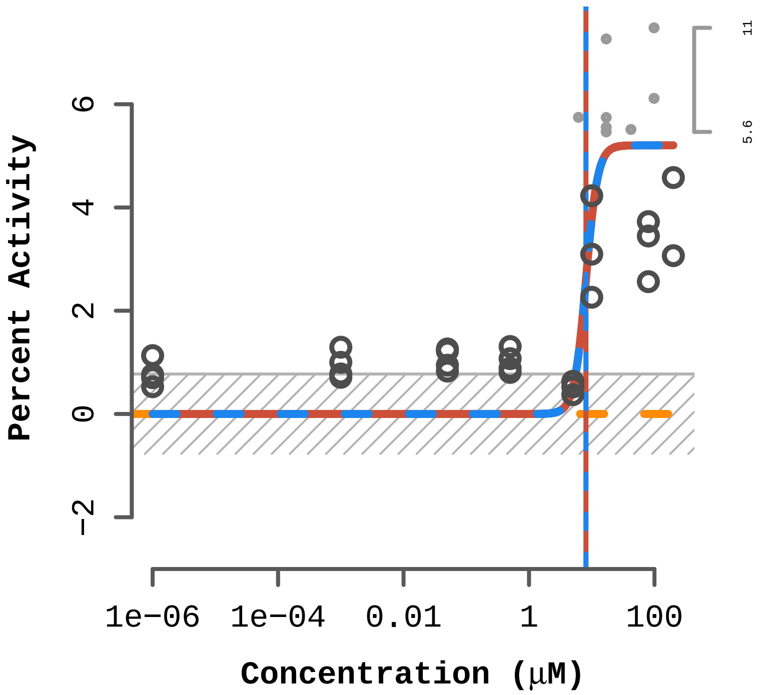

ASSAY: AEID3096 (CCTE_Deisenroth_DEVTOX_RUES2-GLR_Bra_

NAME: Gefitinib
CHID: 41034 CASRN: 184475-35-2
SPID(S): ES211_34_34
M4ID: 42676093 BRK

HILL MODEL (in red):

	tp	ga	gw
val:	5.2	0.908	4.61
sd:	0.523	0.0665	1.92

GAIN-LOSS MODEL (in blue):

	tp	ga	gw	la	lw
val:	5.2	0.908	4.61	4.27	17.3
sd:	NA	NA	NA	NA	NA

	CNST	HILL	GNLS
AIC:	201.81	145.25	149.25
PROB:	0	0.88	0.12
RMSE:	4.05	1.81	1.81

MAX_MEAN: 7.12 MAX_MED: 6.09 BMAD: 0.258

COFF: 0.774 HIT-CALL: 1 FITC: 41 ACTP: 1

FLAGS: 10

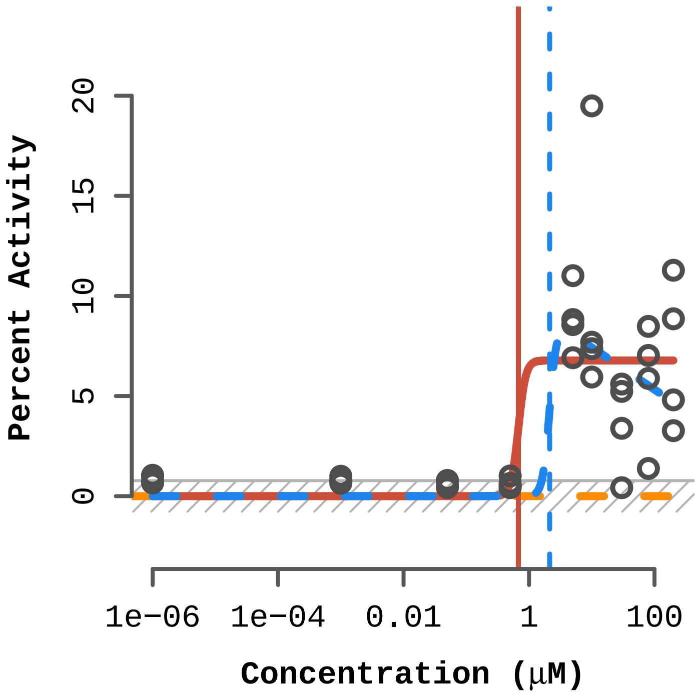

ASSAY: AEID3096 (CCTE_Deisenroth_DEVTOX_RUES2-GLR_Bra_

NAME: Sorafenib
CHID: 41128 CASRN: 284461-73-0
SPID(S): ES211_57_57
M4ID: 42676051

HILL MODEL (in red):

	tp	ga	gw
val:	6.78	-0.17	7.43
sd:	0.528	3.29	186

GAIN-LOSS MODEL (in blue):

	tp	ga	gw	la	lw
val:	12.3	0.329	8	1.6	0.3
sd:	25.2	1.34	29.3	6.28	0.6

	CNST	HILL	GNLS
AIC:	232.01	173.73	174.5
PROB:	0	0.6	0.4
RMSE:	6.07	3.01	2.92

MAX_MEAN: 10.1 MAX_MED: 8.7 BMAD: 0.258

COFF: 0.774 HIT-CALL: 1 FITC: 41 ACTP: 1

FLAGS: 10

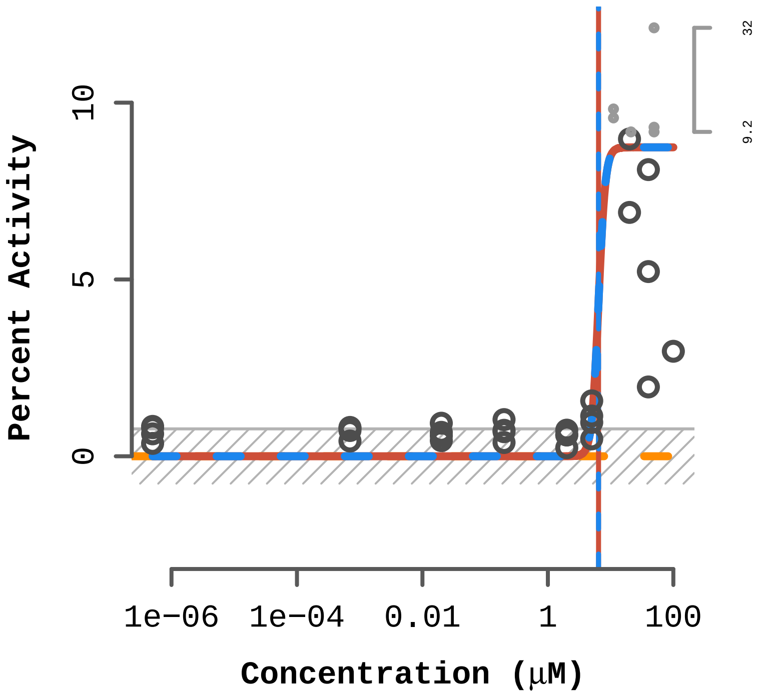

ASSAY: AEID3096 (CCTE_Deisenroth_DEVTOX_RUES2-GLR_Bra_

NAME: Vandetanib
CHID: 46681 CASRN: 443913-73-3
SPID(S): ES211_64_64
M4ID: 42676081 BRK

HILL MODEL (in red):

	tp	ga	gw
val:	8.74	0.808	8
sd:	0.588	0.569	42

GAIN-LOSS MODEL (in blue):

	tp	ga	gw	la	lw
val:	8.74	0.808	8	2.78	10.6
sd:	0.588	0.579	42.7	662	8950

	CNST	HILL	GNLS
AIC:	226.71	168.97	172.97
PROB:	0	0.88	0.12
RMSE:	7.29	4.41	4.41

MAX_MEAN: 13.7 MAX_MED: 10.6 BMAD: 0.258

COFF: 0.774 HIT-CALL: 1 FITC: 41 ACTP: 1

FLAGS: 10

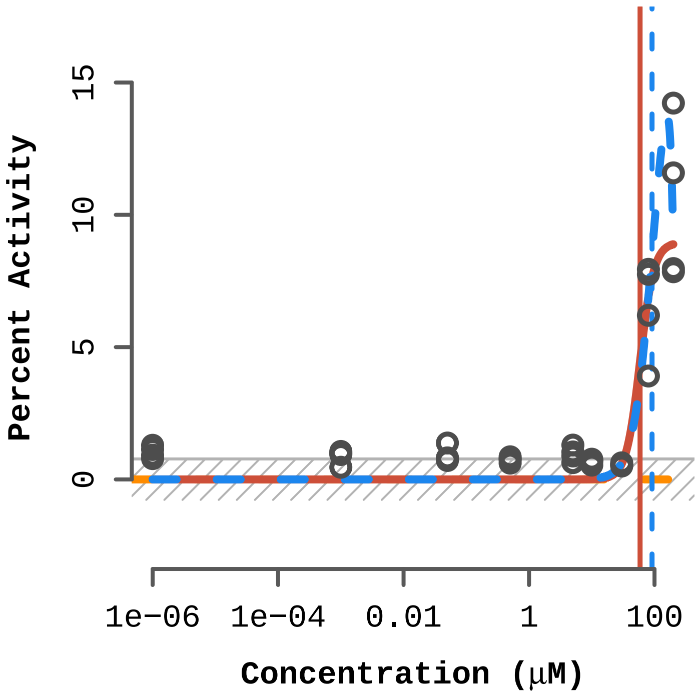

ASSAY: AEID3096 (CCTE_Deisenroth_DEVTOX_RUES2-GLR_Bra_

NAME: Esomeprazole magnesium hydrate
CHID: 746660 CASRN: 668985-31-7
SPID(S): ES211_43_43
M4ID: 42676083

HILL MODEL (in red):

	tp	ga	gw
val:	8.96	1.77	3.95
sd:	1.14	0.0644	1.1

GAIN-LOSS MODEL (in blue):

	tp	ga	gw	la	lw
val:	17.1	1.96	2.94	2.31	12.9
sd:	NaN	NaN	NaN	NaN	NaN

	CNST	HILL	GNLS
AIC:	194.2	126.47	130.35
PROB:	0	0.87	0.13
RMSE:	4.28	1.38	1.38

MAX_MEAN: 10.4 MAX_MED: 9.78 BMAD: 0.258

COFF: 0.774 HIT-CALL: 1 FITC: 41 ACTP: 1

FLAGS: 10

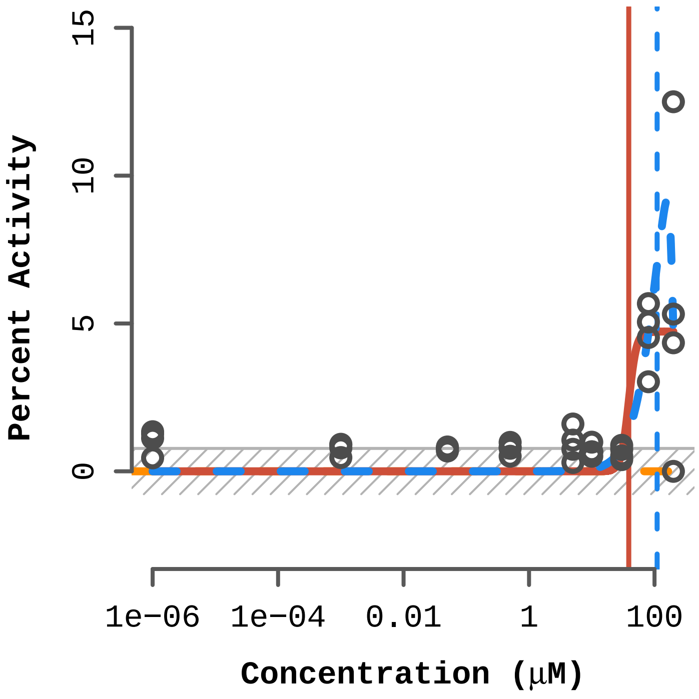

ASSAY: AEID3096 (CCTE_Deisenroth_DEVTOX_RUES2-GLR_Bra_

NAME: Mianserin hydrochloride
CHID: 944145 CASRN: 21535-47-7
SPID(S): ES211_47_47
M4ID: 42676075

HILL MODEL (in red):

	tp	ga	gw
val:	4.73	1.59	7.17
sd:	1.27	1.2	75.6

GAIN-LOSS MODEL (in blue):

	tp	ga	gw	la	lw
val:	14	2.04	2.16	2.29	14
sd:	NaN	NaN	NaN	NaN	NaN

	CNST	HILL	GNLS
AIC:	163.6	126.57	130.01
PROB:	0	0.85	0.15
RMSE:	2.94	1.72	1.71

MAX_MEAN: 5.54 MAX_MED: 4.83 BMAD: 0.258

COFF: 0.774 HIT-CALL: 1 FITC: 41 ACTP: 1

FLAGS: 10

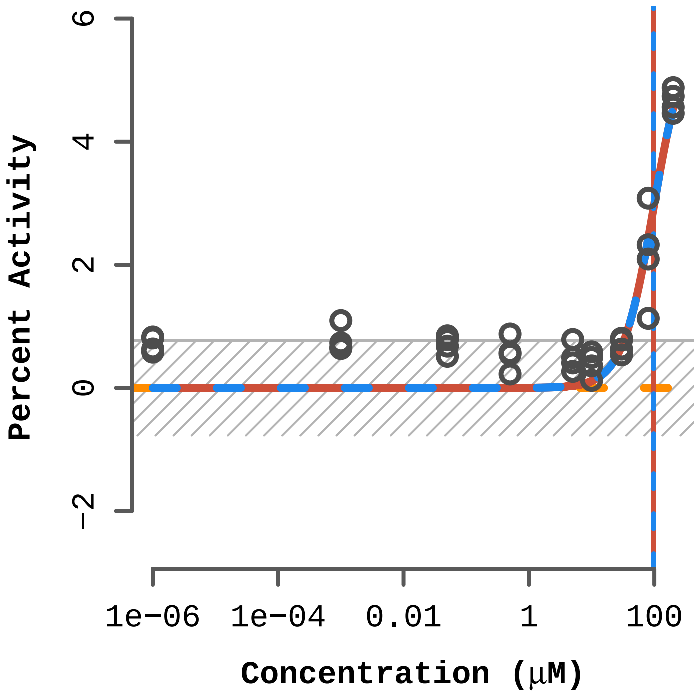

ASSAY: AEID3096 (CCTE_Deisenroth_DEVTOX_RUES2-GLR_Bra_

NAME: 4-Hydroxybenzoic acid butyl ester
CHID: 20209 CASRN: 94-26-8
SPID(S): ES211_19_19
M4ID: 42676062

HILL MODEL (in red):

	tp	ga	gw
val:	5.85	1.99	1.73
sd:	1.08	0.123	0.457

GAIN-LOSS MODEL (in blue):

	tp	ga	gw	la	lw
val:	5.85	1.99	1.73	4.06	4.76
sd:	1.08	0.123	0.457	1440	3910

	CNST	HILL	GNLS
AIC:	138.74	75	79
PROB:	0	0.88	0.12
RMSE:	1.82	0.58	0.58

MAX_MEAN: 4.66 MAX_MED: 4.65 BMAD: 0.258

COFF: 0.774 HIT-CALL: 1 FITC: 42 ACTP: 1

FLAGS:

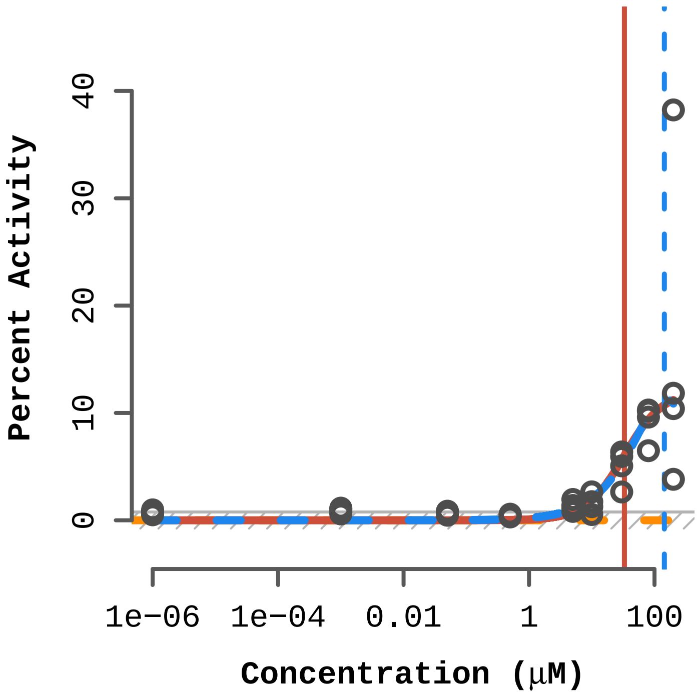

ASSAY: AEID3096 (CCTE_Deisenroth_DEVTOX_RUES2-GLR_Bra_

NAME: Lovastatin
CHID: 20784 CASRN: 75330-75-5
SPID(S): ES211_42_42
M4ID: 42676060

HILL MODEL (in red):

	tp	ga	gw
val:	12.1	1.52	1.41
sd:	1.22	0.093	0.285

GAIN-LOSS MODEL (in blue):

	tp	ga	gw	la	lw
val:	31	2.16	0.995	2.41	1.72
sd:	NaN	NaN	NaN	NaN	NaN

	CNST	HILL	GNLS
AIC:	224.93	148.19	150.62
PROB:	0	0.77	0.23
RMSE:	7.83	4.77	4.81

MAX_MEAN: 16.1 MAX_MED: 11.1 BMAD: 0.258

COFF: 0.774 HIT-CALL: 1 FITC: 42 ACTP: 1

FLAGS: 10

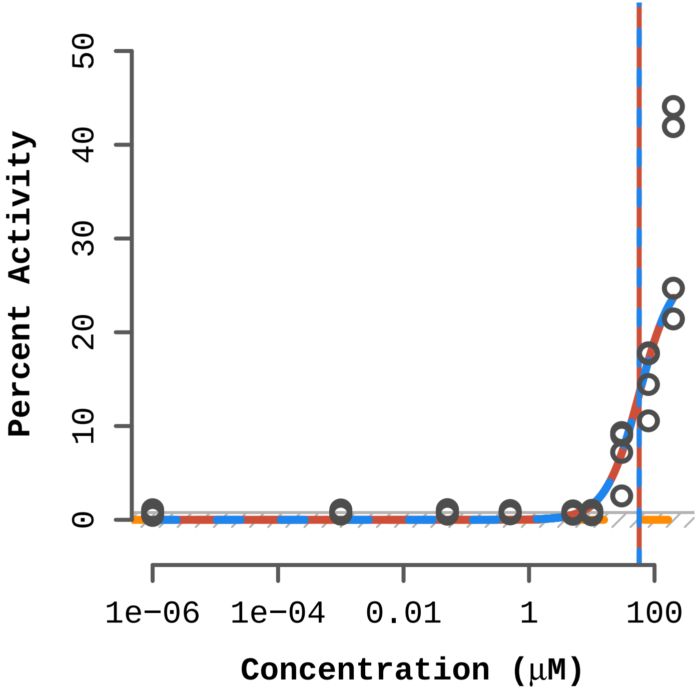

ASSAY: AEID3096 (CCTE_Deisenroth_DEVTOX_RUES2-GLR_Bra_

NAME: 13-cis Retinoic acid
CHID: 23177 CASRN: 4759-48-2
SPID(S): ES211_2_2
M4ID: 42676048

HILL MODEL (in red):

	tp	ga	gw
val:	26.9	1.76	1.6
sd:	3.3	0.0969	0.249

GAIN-LOSS MODEL (in blue):

	tp	ga	gw	la	lw
val:	26.9	1.76	1.6	4.14	4.14
sd:	3.3	0.0969	0.249	949	2160

	CNST	HILL	GNLS
AIC:	273.14	176.76	180.76
PROB:	0	0.88	0.12
RMSE:	12.87	4.85	4.85

MAX_MEAN: 33 MAX_MED: 33.3 BMAD: 0.258

COFF: 0.774 HIT-CALL: 1 FITC: 42 ACTP: 1

FLAGS: 10

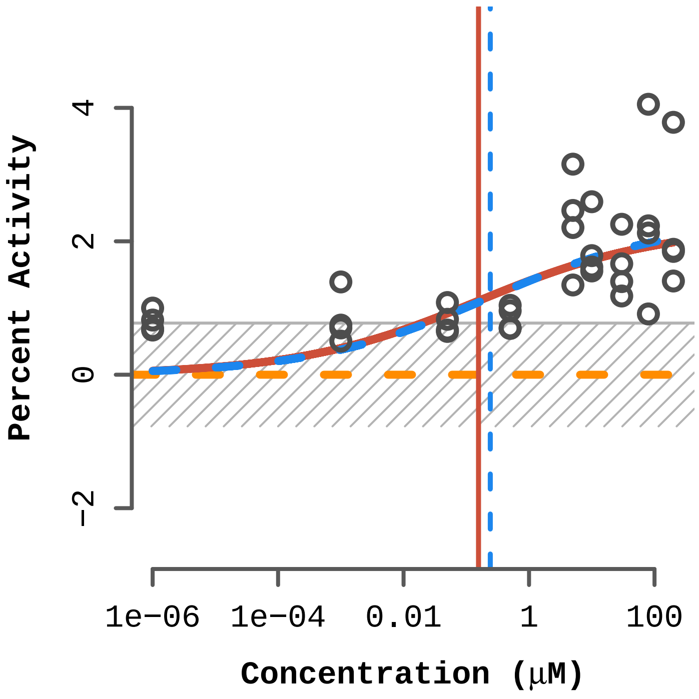

ASSAY: AEID3096 (CCTE_Deisenroth_DEVTOX_RUES2-GLR_Bra_

NAME: Cytarabine hydrochloride
CHID: 24891 CASRN: 69-74-9
SPID(S): ES211_25_25
M4ID: 42676099

HILL MODEL (in red):

	tp	ga	gw
val:	2.22	−0.805	0.3
sd:	NaN	NaN	NaN

GAIN-LOSS MODEL (in blue):

	tp	ga	gw	la	lw
val:	2.32	−0.618	0.3	2.38	13.3
sd:	NaN	NaN	NaN	NaN	NaN

	CNST	HILL	GNLS
AIC:	148.22	84.96	88.83
PROB:	0	0.87	0.13
RMSE:	1.75	0.72	0.72

MAX_MEAN: 2.33 MAX_MED: 2.33 BMAD: 0.258

COFF: 0.774 HIT-CALL: 1 FITC: 42 ACTP: 1

FLAGS:

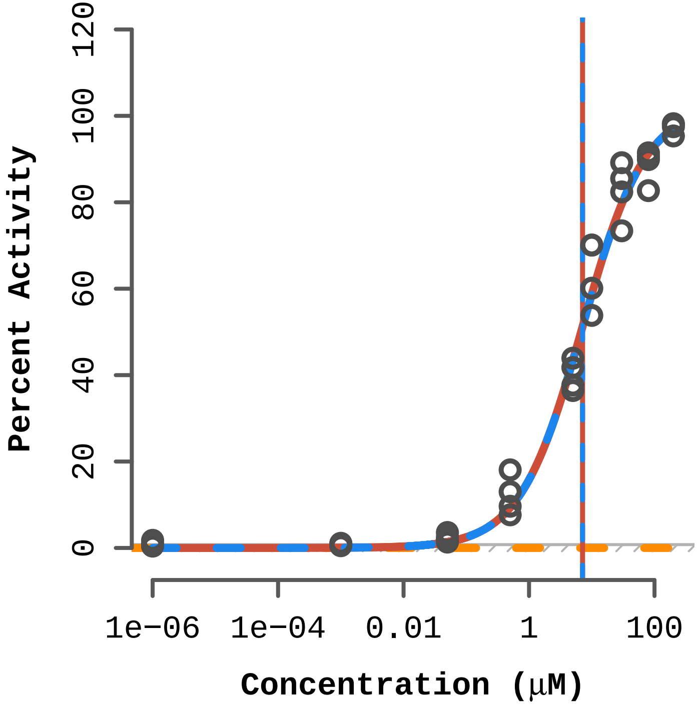

ASSAY: AEID3096 (CCTE_Deisenroth_DEVTOX_RUES2-GLR_Bra_

NAME: Adriamycin hydrochloride
CHID: 30636 CASRN: 25316-40-9
SPID(S): ES211_67_67
M4ID: 42676061

HILL MODEL (in red):

	tp	ga	gw
val:	102	0.853	0.867
sd:	2.75	0.0488	0.0841

GAIN-LOSS MODEL (in blue):

	tp	ga	gw	la	lw
val:	102	0.853	0.867	3.52	11.4
sd:	2.75	0.0488	0.0841	106000	653000

	CNST	HILL	GNLS
AIC:	399.74	214.15	218.15
PROB:	0	0.88	0.12
RMSE:	57.79	4.51	4.51

MAX_MEAN: 97.3 MAX_MED: 97.8 BMAD: 0.258

COFF: 0.774 HIT-CALL: 1 FITC: 42 ACTP: 1

FLAGS: 10

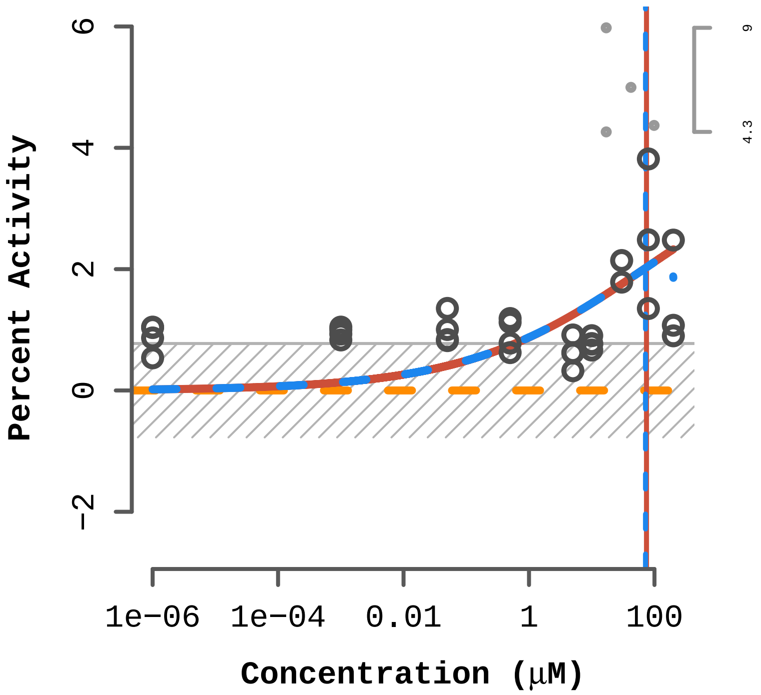

ASSAY: AEID3096 (CCTE_Deisenroth_DEVTOX_RUES2-GLR_Bra_

NAME: Amiodarone hydrochloride
CHID: 37185 CASRN: 19774-82-4
SPID(S): ES211_14_14
M4ID: 42676096 BRK

HILL MODEL (in red):

	tp	ga	gw
val:	4.06	1.87	0.3
sd:	NaN	NaN	NaN

GAIN-LOSS MODEL (in blue):

	tp	ga	gw	la	lw
val:	4.04	1.86	0.3	2.34	16.1
sd:	NaN	NaN	NaN	NaN	NaN

	CNST	HILL	GNLS
AIC:	158.84	129.65	133.16
PROB:	0	0.85	0.15
RMSE:	2.45	1.67	1.67

MAX_MEAN: 4.3 MAX_MED: 3.22 BMAD: 0.258

COFF: 0.774 HIT-CALL: 1 FITC: 42 ACTP: 1

FLAGS: 10

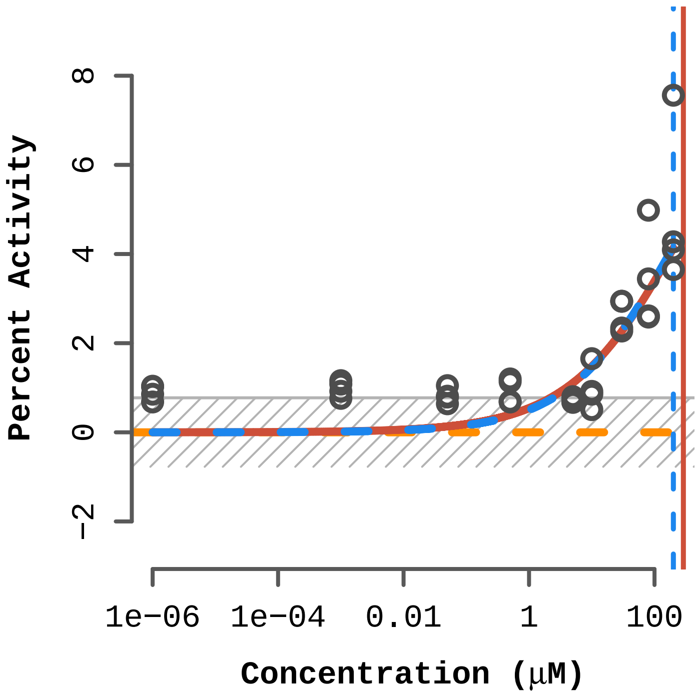

ASSAY: AEID3096 (CCTE_Deisenroth_DEVTOX_RUES2-GLR_Bra_

NAME: 6-aminopyridine-3-carboxamide
CHID: 51446 CASRN: 329-89-5
SPID(S): ES211_7_7
M4ID: 42676090

HILL MODEL (in red):

	tp	ga	gw
val:	9.08	2.46	0.486
sd:	6.51	1.25	0.21

GAIN-LOSS MODEL (in blue):

	tp	ga	gw	la	lw
val:	8.34	2.3	0.518	3.61	14
sd:	NA	NA	NA	NA	NA

	CNST	HILL	GNLS
AIC:	164.36	99.53	103.72
PROB:	0	0.89	0.11
RMSE:	2.38	0.91	0.91

MAX_MEAN: 4.9 MAX_MED: 4.18 BMAD: 0.258

COFF: 0.774 HIT-CALL: 1 FITC: 42 ACTP: 1

FLAGS: 10

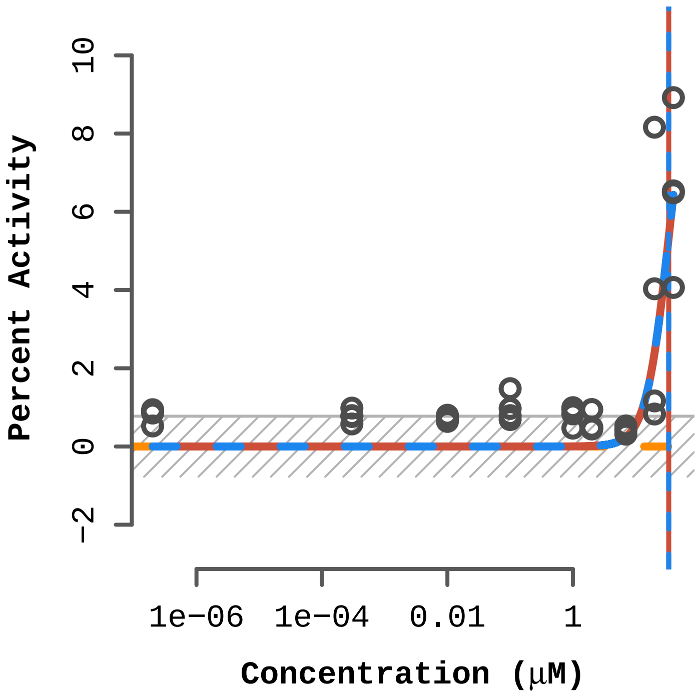

ASSAY: AEID3096 (CCTE_Deisenroth_DEVTOX_RUES2-GLR_Bra_

NAME: Dorsomorphin
CHID: 1006988 CASRN: 866405-64-3
SPID(S): ES211_6_6
M4ID: 42676084

HILL MODEL (in red):

	tp	ga	gw
val:	10.7	1.53	2.39
sd:	8.1	0.299	1.9

GAIN-LOSS MODEL (in blue):

	tp	ga	gw	la	lw
val:	10.7	1.53	2.39	3.6	3.51
sd:	8.1	0.299	1.9	989	1760

	CNST	HILL	GNLS
AIC:	161.29	123.14	127.14
PROB:	0	0.88	0.12
RMSE:	2.8	1.37	1.37

MAX_MEAN: 6.5 MAX_MED: 6.51 BMAD: 0.258

COFF: 0.774 HIT-CALL: 1 FITC: 42 ACTP: 1

FLAGS: 10

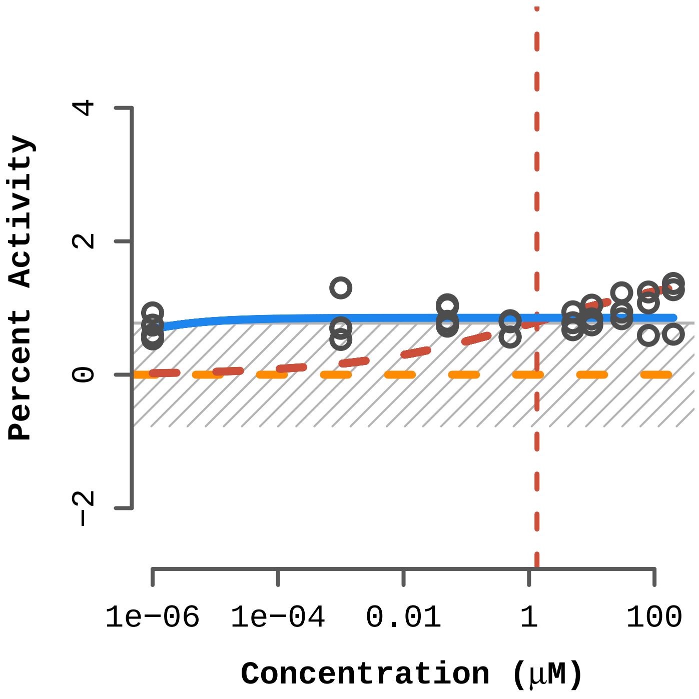

ASSAY: AEID3096 (CCTE_Deisenroth_DEVTOX_RUES2-GLR_Bra_

NAME: Folic acid
CHID: 22519 CASRN: 59-30-3
SPID(S): ES211_33_33
M4ID: 42676044

HILL MODEL (in red):

	tp	ga	gw
val:	1.59	0.127	0.3
sd:	NaN	NaN	NaN

GAIN-LOSS MODEL (in blue):

	tp	ga	gw	la	lw
val:	0.853	-7	0.608	4.27	17.8
sd:	NA	NA	NA	NA	NA

	CNST	HILL	GNLS
AIC:	89.21	47.51	13.03
PROB:	0	0	1
RMSE:	0.89	0.45	0.24

MAX_MEAN: 1.08 MAX_MED: 1.27 BMAD: 0.258

COFF: 0.774 HIT-CALL: 1 FITC: 45 ACTP: 1

FLAGS: 11; 18

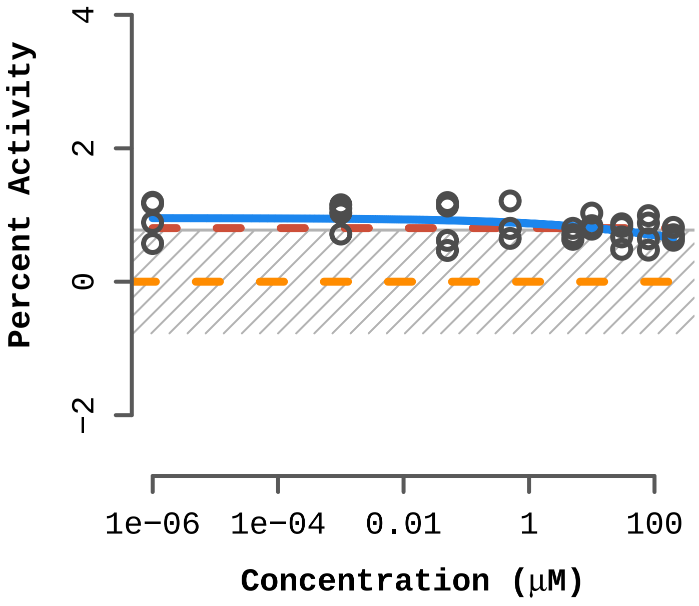

ASSAY: AEID3096 (CCTE_Deisenroth_DEVTOX_RUES2-GLR_Bra_

NAME: Ibuprofen
CHID: 20732 CASRN: 15687-27-1
SPID(S): ES211_36_36
M4ID: 42676091

HILL MODEL (in red):

	tp	ga	gw
val:	0.804	-7	7.35
sd:	0.0381	161	1030

GAIN-LOSS MODEL (in blue):

	tp	ga	gw	la	lw
val:	0.955	-6.7	7.4	3.46	0.3
sd:	NaN	NaN	NaN	NaN	NaN

	CNST	HILL	GNLS
AIC:	100.17	3.72	1.47
PROB:	0	0.24	0.76
RMSE:	0.85	0.22	0.2

MAX_MEAN: 1 MAX_MED: 1.07 BMAD: 0.258

COFF: 0.774 HIT-CALL: 1 FITC: 49 ACTP: 1

FLAGS: 18

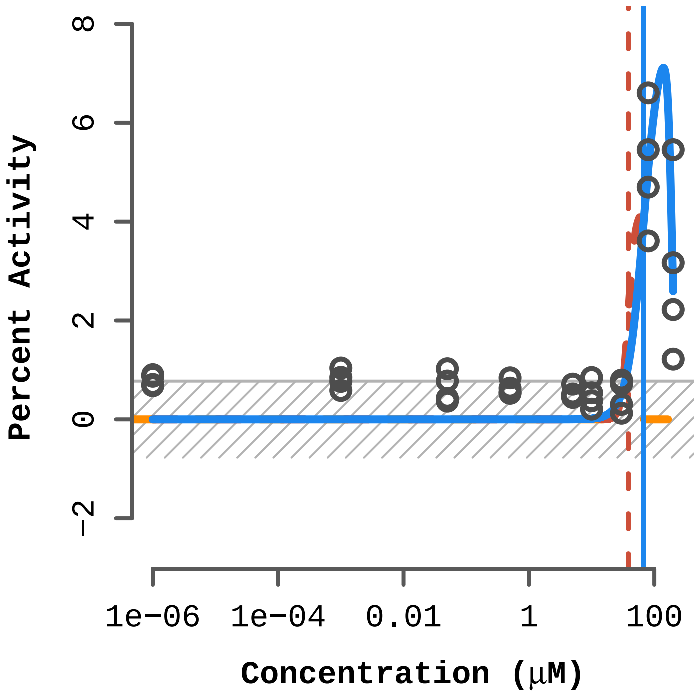

ASSAY: AEID3096 (CCTE_Deisenroth_DEVTOX_RUES2-GLR_Bra_

NAME: Bisphenol A
CHID: 20182 CASRN: 80-05-7
SPID(S): ES211_16_16
M4ID: 42676059

HILL MODEL (in red):

	tp	ga	gw
val:	4.24	1.59	8
sd:	0.556	0.0838	5.61

GAIN-LOSS MODEL (in blue):

	tp	ga	gw	la	lw
val:	7.92	1.83	3.29	2.28	12.5
sd:	23.2	0.947	5.58	0.0231	66.3

	CNST	HILL	GNLS
AIC:	148.11	108.64	103.71
PROB:	0	0.08	0.92
RMSE:	2.16	0.99	0.86

MAX_MEAN: 5.09 MAX_MED: 5.08 BMAD: 0.258

COFF: 0.774 HIT-CALL: 1 FITC: 50 ACTP: 1

FLAGS: 10

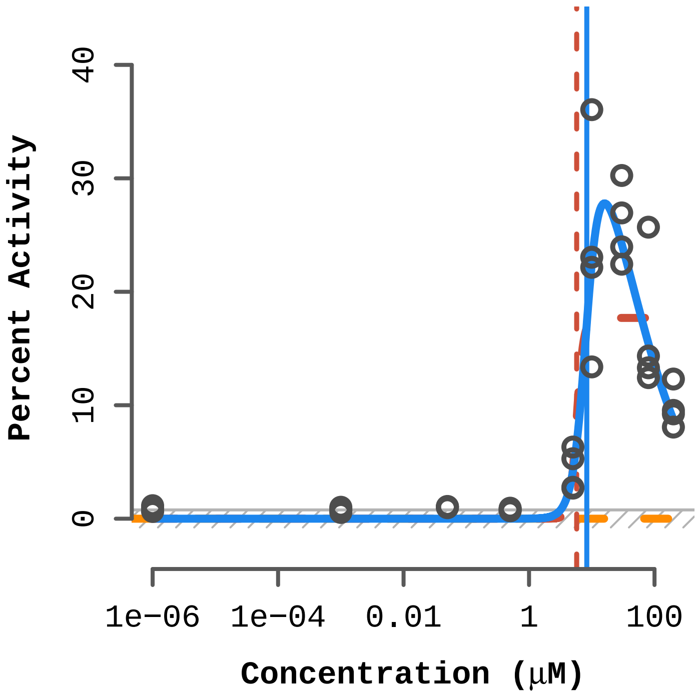

ASSAY: AEID3096 (CCTE_Deisenroth_DEVTOX_RUES2-GLR_Bra_

NAME: 1-(p-Chlorobenzoyl)-5-methoxy-2-methyl-Indole-3
CHID: 20740 CASRN: 53-86-1
SPID(S): ES211_38_38
M4ID: 42676077

HILL MODEL (in red):

	tp	ga	gw
val:	17.7	0.759	8
sd:	2.26	0.0415	4.36

GAIN-LOSS MODEL (in blue):

	tp	ga	gw	la	lw
val:	43.3	0.921	4.04	1.61	0.858
sd:	14	0.0404	0.542	0.335	0.187

	CNST	HILL	GNLS
AIC:	291.3	229.42	186.49
PROB:	0	0	1
RMSE:	13.85	5.59	3.62

MAX_MEAN: 25.9 MAX_MED: 25.5 BMAD: 0.258

COFF: 0.774 HIT-CALL: 1 FITC: 50 ACTP: 1

FLAGS: 10

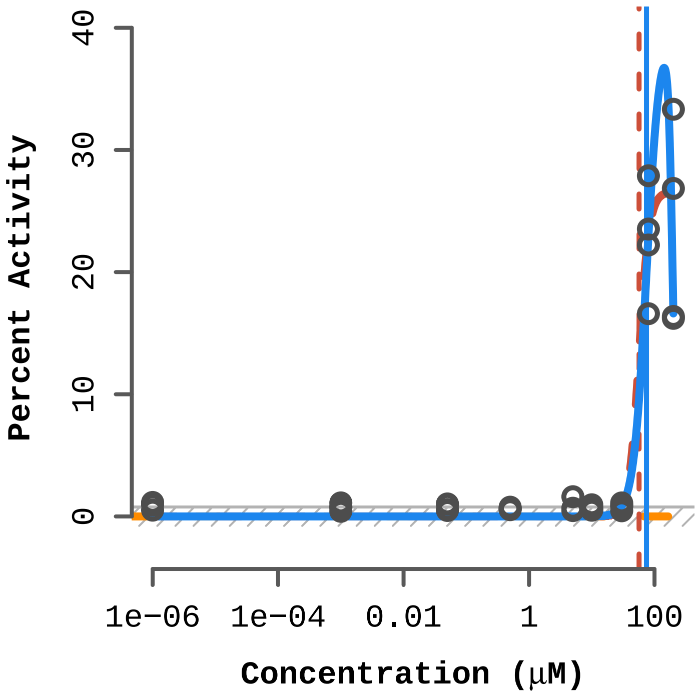

ASSAY: AEID3096 (CCTE_Deisenroth_DEVTOX_RUES2-GLR_Bra_

NAME: Retinol
CHID: 23556 CASRN: 68−26−8
SPID(S): ES211_51_51
M4ID: 42676071

HILL MODEL (in red):

	tp	ga	gw
val:	26.6	1.75	5.38
sd:	1.21	0.0304	0.794

GAIN-LOSS MODEL (in blue):

	tp	ga	gw	la	lw
val:	40	1.87	4.21	2.29	12.2
sd:	9.43	0.0457	0.998	0.0239	21

	CNST	HILL	GNLS
AIC:	261.81	164.91	161.86
PROB:	0	0.18	0.82
RMSE:	11.16	3.07	3.6

MAX_MEAN: 23.2 MAX_MED: 22.9 BMAD: 0.258

COFF: 0.774 HIT-CALL: 1 FITC: 50 ACTP: 1

FLAGS: 10

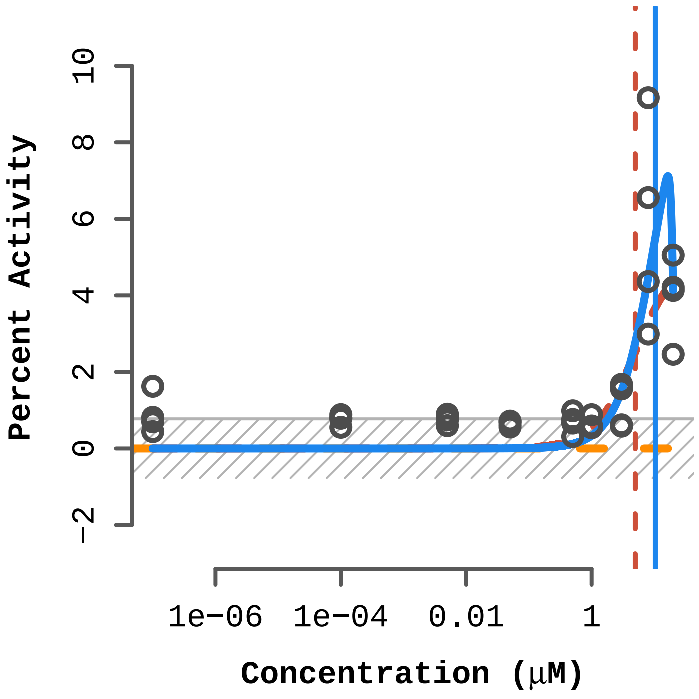

ASSAY: AEID3096 (CCTE_Deisenroth_DEVTOX_RUES2-GLR_Bra_

NAME: Ziprasidone
CHID: 23753 CASRN: 146939-27-7
SPID(S): ES211_66_66
M4ID: 42676046

HILL MODEL (in red):

	tp	ga	gw
val:	5.04	0.696	1.34
sd:	1.96	0.363	1.25

GAIN-LOSS MODEL (in blue):

	tp	ga	gw	la	lw
val:	11	1.02	1.48	1.3	17.9
sd:	NaN	NaN	NaN	NaN	NaN

	CNST	HILL	GNLS
AIC:	161.94	114.79	114.7
PROB:	0	0.49	0.51
RMSE:	2.59	1.34	1.14

MAX_MEAN: 5.77 MAX_MED: 5.46 BMAD: 0.258

COFF: 0.774 HIT-CALL: 1 FITC: 51 ACTP: 1

FLAGS: 10

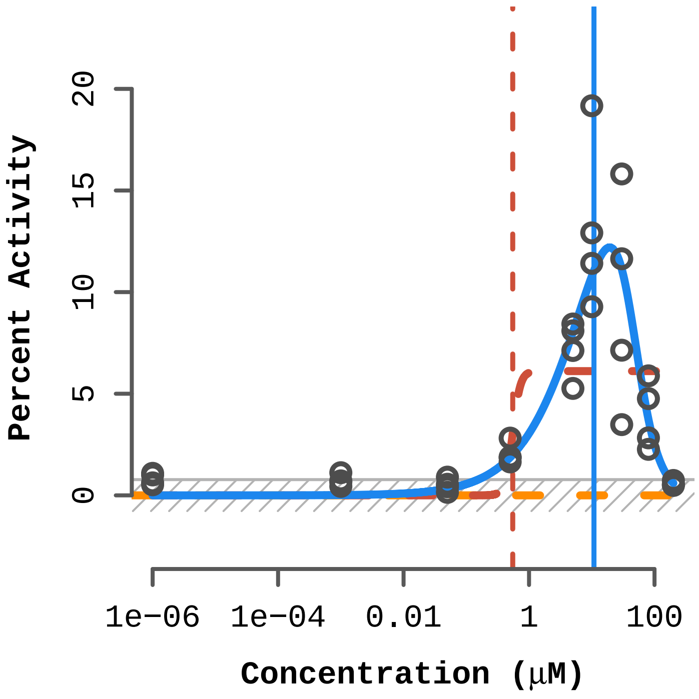

ASSAY: AEID3096 (CCTE_Deisenroth_DEVTOX_RUES2-GLR_Bra_

NAME: Dasatinib
CHID: 40979 CASRN: 302962-49-8
SPID(S): ES211_26_26
M4ID: 42676040

HILL MODEL (in red):

	tp	ga	gw
val:	6.11	-0.26	7.22
sd:	0.915	2.82	493

GAIN-LOSS MODEL (in blue):

	tp	ga	gw	la	lw
val:	23	1.04	0.789	1.64	2.31
sd:	12	0.505	0.174	0.126	0.484

	CNST	HILL	GNLS
AIC:	233.91	202.71	156.69
PROB:	0	0	1
RMSE:	6.48	3.93	2.35

MAX_MEAN: 13.2 MAX_MED: 12.2 BMAD: 0.258

COFF: 0.774 HIT-CALL: 1 FITC: 51 ACTP: 1

FLAGS: 10

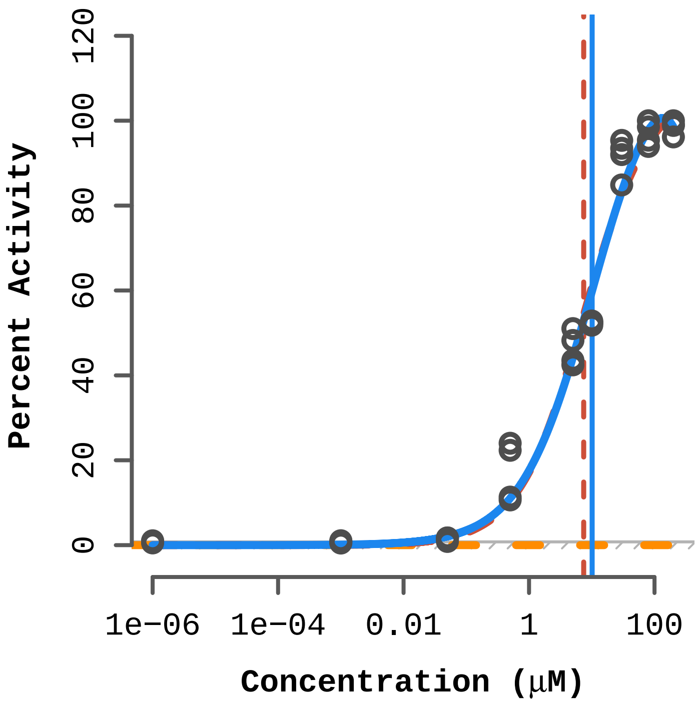

ASSAY: AEID3096 (CCTE_Deisenroth_DEVTOX_RUES2-GLR_Bra_

NAME: Sunitinib malate
CHID: 46492 CASRN: 341031-54-7
SPID(S): ES211_60_60
M4ID: 42676102

HILL MODEL (in red):

	tp	ga	gw
val:	108	0.871	0.829
sd:	3.88	0.0659	0.0944

GAIN-LOSS MODEL (in blue):

	tp	ga	gw	la	lw
val:	120	1.01	0.761	2.78	2.04
sd:	12.8	0.137	0.0805	0.461	2.5

	CNST	HILL	GNLS
AIC:	402.64	232.51	228.05
PROB:	0	0.1	0.9
RMSE:	60.31	5.65	5.13

MAX_MEAN: 98.5 MAX_MED: 99 BMAD: 0.258

COFF: 0.774 HIT-CALL: 1 FITC: 51 ACTP: 1

FLAGS: 10
