# Supplementary material for: The DevTox Germ Layer Reporter Platform: An Assay Adaptation of the Human Pluripotent Stem Cell Test
Source: Toxics. 2022 Jul 13;10(7):392. doi: 10.3390/toxics10070392 (PMC9321663; doi:10.3390/toxics10070392)
Supplement: Supplementary file 1 [file toxics-10-00392-s001.zip › AEID3097_L6_CCTE_Deisenroth_DEVTOX_RUES2-GLR_CellCount_up_210917.pdf]

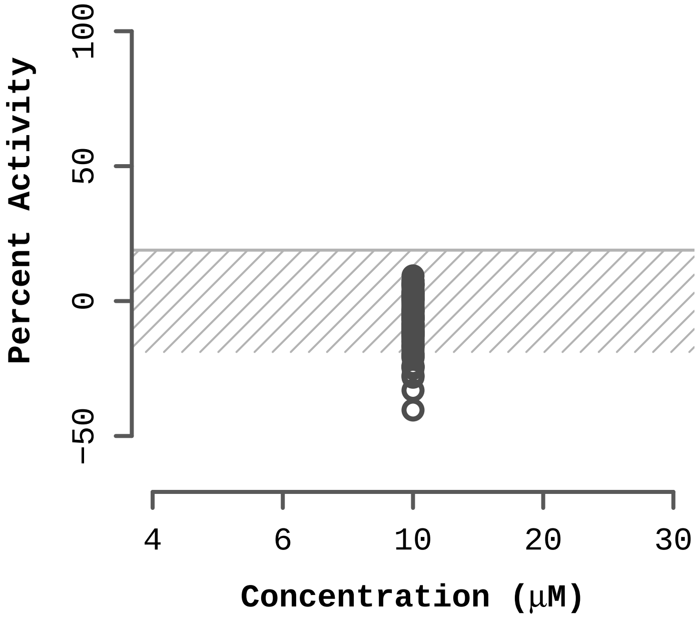

ASSAY: AEID3097 (CCTE_Deisenroth_DEVTOX_RUES2-GLR_Cel

NAME: SB-431542
CHID: 42693 CASRN: 301836-41-9
SPID(S): ES211_56_56
M4ID: 42676119

HILL MODEL: Not applicable.

GAIN-LOSS MODEL: Not applicable.

	CNST	HILL	GNLS
AIC:	NA	NA	NA
PROB:	NA	NA	NA
RMSE:	NA	NA	NA

MAX_MEAN: -8.7 MAX_MED: -8.21 BMAD: 6.29

COFF: 18.9 HIT-CALL: -1 FITC: 2 ACTP: NA

FLAGS:

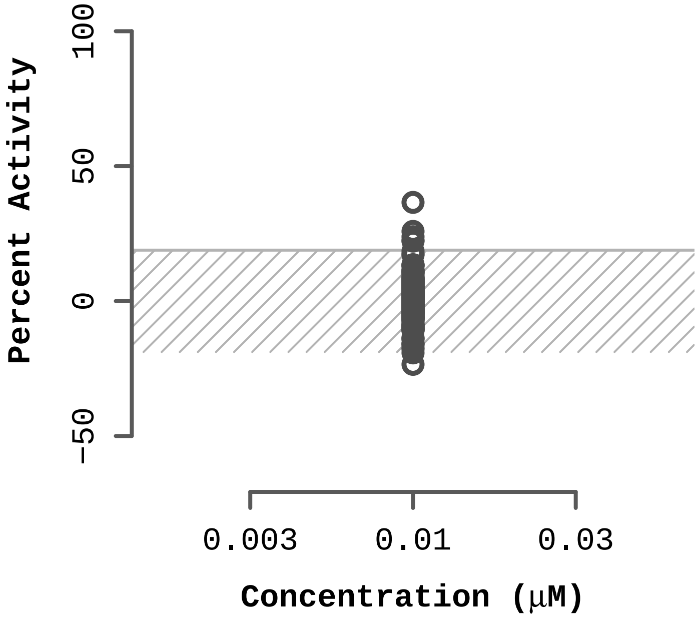

ASSAY: AEID3097 (CCTE_Deisenroth_DEVTOX_RUES2-GLR_Cel

NAME: DMSO (spid not in DB)
CHID: NA CASRN: NA
SPID(S): DMSO
M4ID: 42676123

HILL MODEL: Not applicable.

GAIN−LOSS MODEL: Not applicable.

	CNST	HILL	GNLS
AIC:	NA	NA	NA
PROB:	NA	NA	NA
RMSE:	NA	NA	NA

MAX_MEAN: 0.368 MAX_MED: 0 BMAD: 6.29

COFF: 18.9 HIT−CALL: −1 FITC: 2 ACTP: NA

FLAGS:

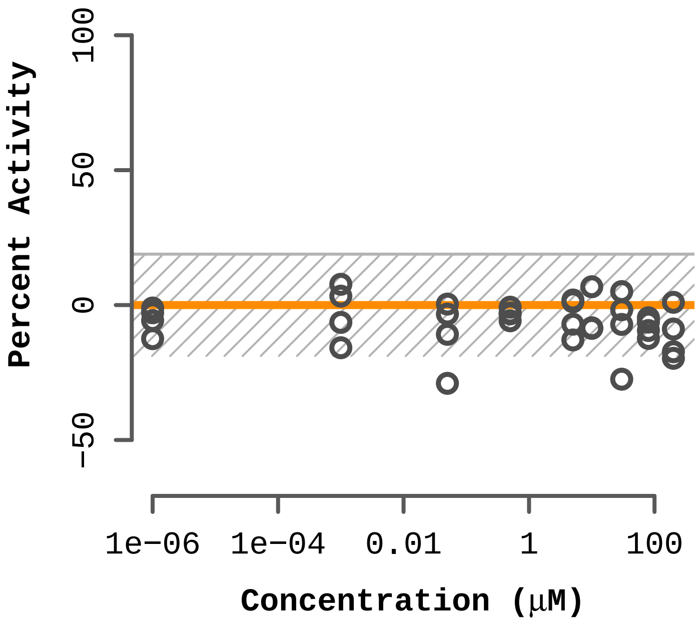

ASSAY: AEID3097 (CCTE_Deisenroth_DEVTOX_RUES2-GLR_Cel

NAME: Acetaminophen
CHID: 20006 CASRN: 103−90−2
SPID(S): ES211_9_9
M4ID: 42676147

HILL MODEL: Not applicable.

GAIN−LOSS MODEL: Not applicable.

	CNST	HILL	GNLS
AIC:	272.43	NA	NA
PROB:	1	NA	NA
RMSE:	10.5	NA	NA

MAX_MEAN: −2.8 MAX_MED: −1.55 BMAD: 6.29

COFF: 18.9 HIT−CALL: 0 FITC: 4 ACTP: 0

FLAGS:

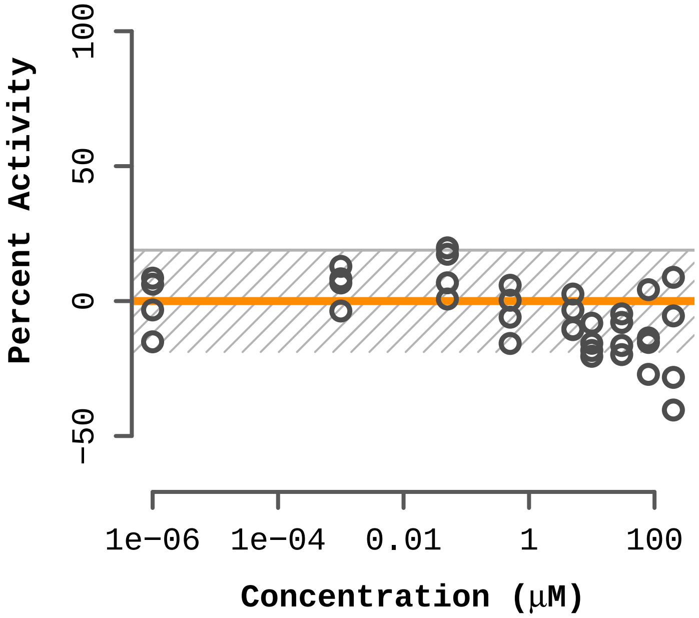

ASSAY: AEID3097 (CCTE_Deisenroth_DEVTOX_RUES2-GLR_Cel

NAME: Acrylamide
CHID: 20027 CASRN: 79−06−1
SPID(S): ES211_10_10
M4ID: 42676126

HILL MODEL: Not applicable.

GAIN−LOSS MODEL: Not applicable.

	CNST	HILL	GNLS
AIC:	297.76	NA	NA
PROB:	1	NA	NA
RMSE:	14.39	NA	NA

MAX_MEAN: 11.2 MAX_MED: 12.1 BMAD: 6.29

COFF: 18.9 HIT−CALL: 0 FITC: 4 ACTP: 0

FLAGS:

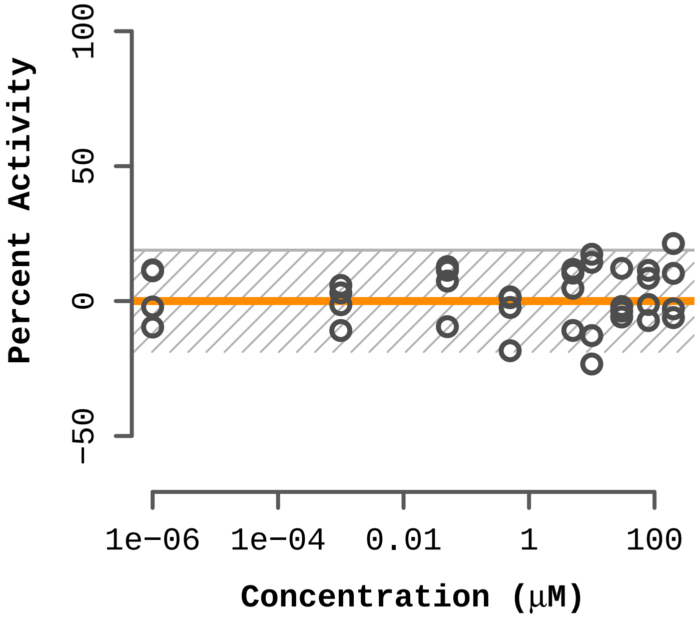

ASSAY: AEID3097 (CCTE_Deisenroth_DEVTOX_RUES2-GLR_Cel

NAME: Aspirin
CHID: 20108 CASRN: 50−78−2
SPID(S): ES211_15_15
M4ID: 42676143

HILL MODEL: Not applicable.

GAIN−LOSS MODEL: Not applicable.

	CNST	HILL	GNLS
AIC:	277.55	NA	NA
PROB:	1	NA	NA
RMSE:	10.5	NA	NA

MAX_MEAN: 5.66 MAX_MED: 9.42 BMAD: 6.29

COFF: 18.9 HIT−CALL: 0 FITC: 4 ACTP: 0

FLAGS:

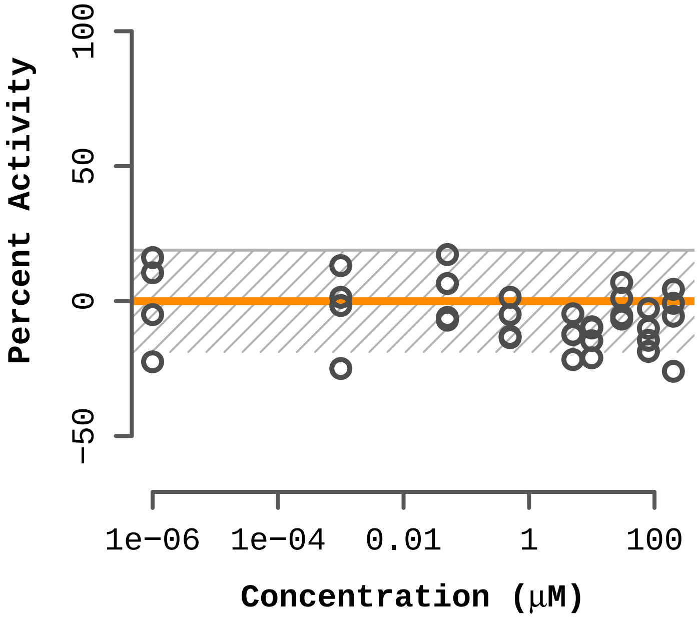

ASSAY: AEID3097 (CCTE_Deisenroth_DEVTOX_RUES2-GLR_Cel

NAME: Boric acid (H3BO3)
CHID: 20194 CASRN: 10043-35-3
SPID(S): ES211_17_17
M4ID: 42676161

HILL MODEL: Not applicable.

GAIN-LOSS MODEL: Not applicable.

	CNST	HILL	GNLS
AIC:	289.92	NA	NA
PROB:	1	NA	NA
RMSE:	12.55	NA	NA

MAX_MEAN: 2.66 MAX_MED: 2.75 BMAD: 6.29

COFF: 18.9 HIT-CALL: 0 FITC: 4 ACTP: 0

FLAGS:

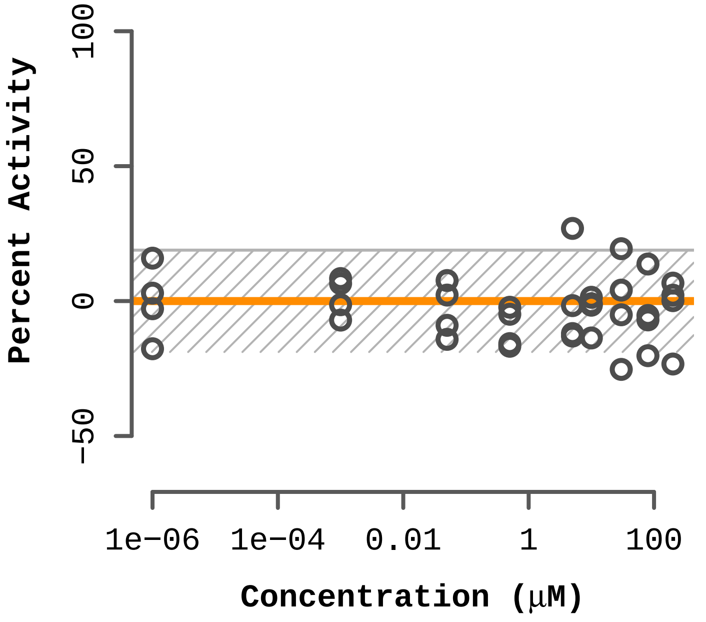

ASSAY: AEID3097 (CCTE_Deisenroth_DEVTOX_RUES2-GLR_Cel

NAME: Caffeine
CHID: 20232 CASRN: 58−08−2
SPID(S): ES211_20_20
M4ID: 42676116

HILL MODEL: Not applicable.

GAIN−LOSS MODEL: Not applicable.

	CNST	HILL	GNLS
AIC:	285.32	NA	NA
PROB:	1	NA	NA
RMSE:	12.03	NA	NA

MAX_MEAN: 1.62 MAX_MED: 2.58 BMAD: 6.29

COFF: 18.9 HIT−CALL: 0 FITC: 4 ACTP: 0

FLAGS:

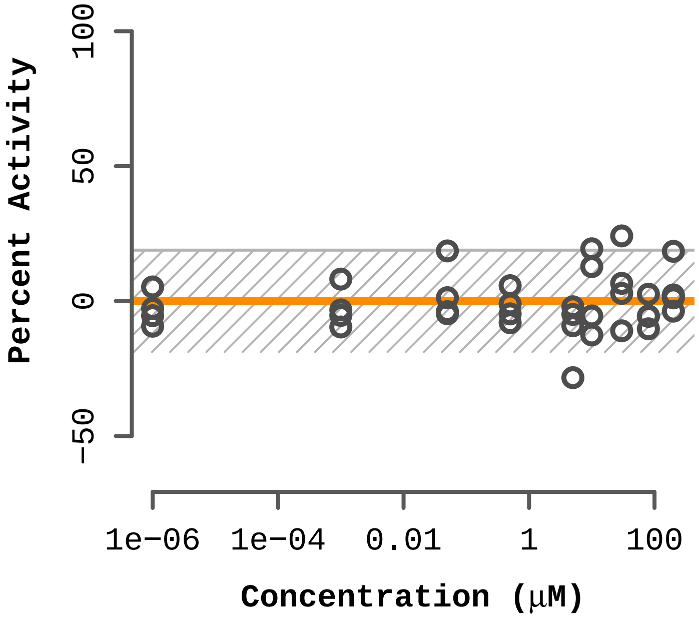

ASSAY: AEID3097 (CCTE_Deisenroth_DEVTOX_RUES2-GLR_Cel

NAME: Ethylene glycol
CHID: 20597 CASRN: 107−21−1
SPID(S): ES211_32_32
M4ID: 42676160

HILL MODEL: Not applicable.

GAIN−LOSS MODEL: Not applicable.

	CNST	HILL	GNLS
AIC:	271.02	NA	NA
PROB:	1	NA	NA
RMSE:	10.26	NA	NA

MAX_MEAN: 5.65 MAX_MED: 4.72 BMAD: 6.29

COFF: 18.9 HIT−CALL: 0 FITC: 4 ACTP: 0

FLAGS:

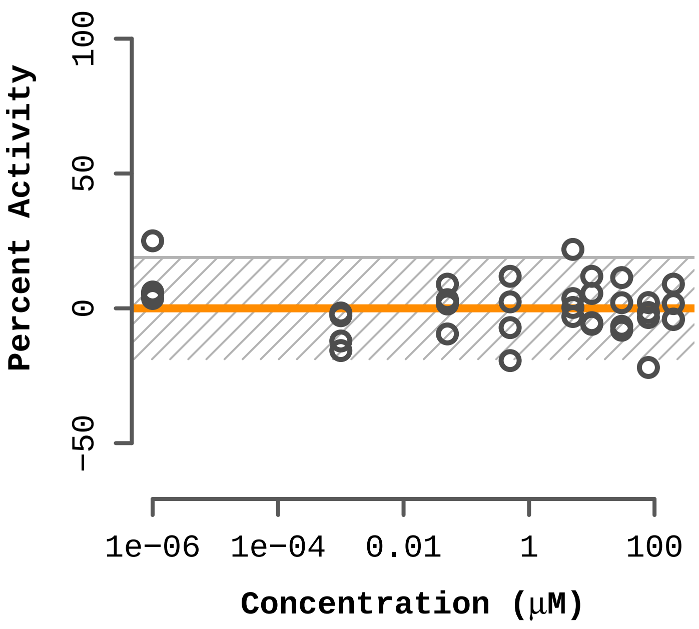

ASSAY: AEID3097 (CCTE_Deisenroth_DEVTOX_RUES2-GLR_Cel

NAME: Isoniazid
CHID: 20755 CASRN: 54-85-3
SPID(S): ES211_39_39
M4ID: 42676153

HILL MODEL: Not applicable.

GAIN-LOSS MODEL: Not applicable.

	CNST	HILL	GNLS
AIC:	267.93	NA	NA
PROB:	1	NA	NA
RMSE:	9.8	NA	NA

MAX_MEAN: 10 MAX_MED: 5.64 BMAD: 6.29

COFF: 18.9 HIT-CALL: 0 FITC: 4 ACTP: 0

FLAGS:

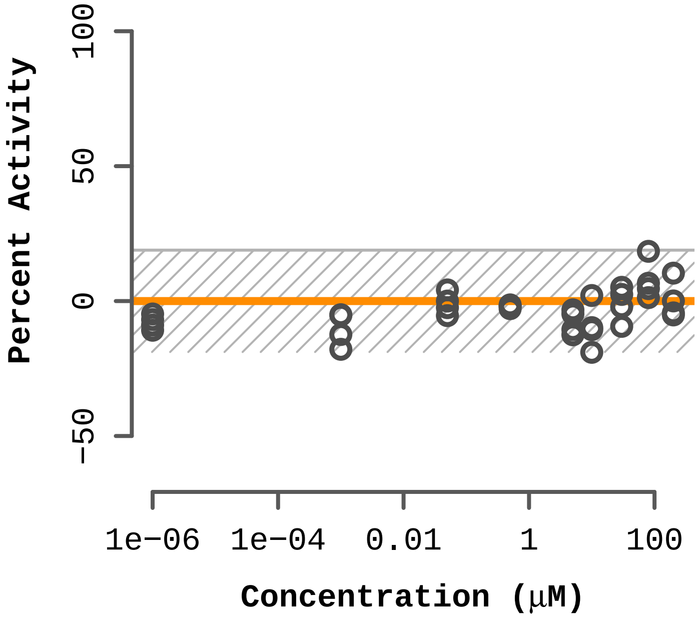

ASSAY: AEID3097 (CCTE_Deisenroth_DEVTOX_RUES2-GLR_Cel

NAME: Methyldopa sesquihydrate
CHID: 20863 CASRN: 41372-08-1
SPID(S): ES211_46_46
M4ID: 42676141

HILL MODEL: Not applicable.

GAIN-LOSS MODEL: Not applicable.

	CNST	HILL	GNLS
AIC:	257.13	NA	NA
PROB:	1	NA	NA
RMSE:	8.19	NA	NA

MAX_MEAN: 7.74 MAX_MED: 5.62 BMAD: 6.29

COFF: 18.9 HIT-CALL: 0 FITC: 4 ACTP: 0

FLAGS:

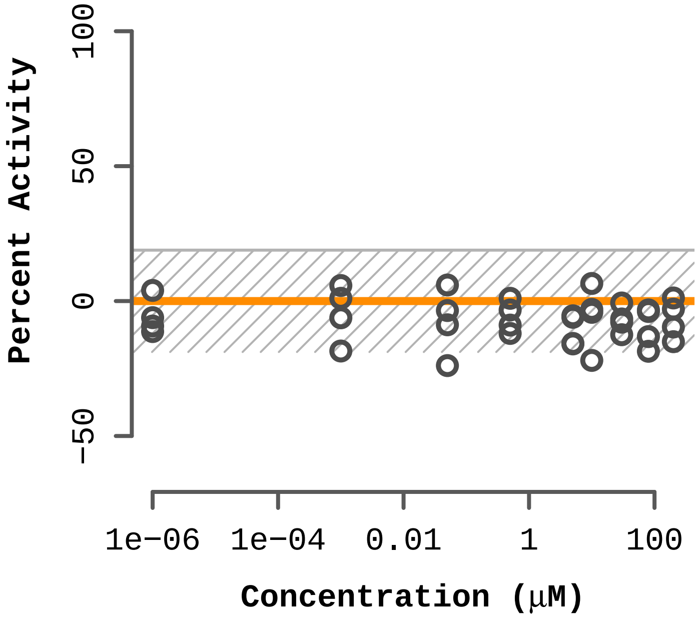

ASSAY: AEID3097 (CCTE_Deisenroth_DEVTOX_RUES2-GLR_Cel

NAME: Nicotinic acid
CHID: 20932 CASRN: 59-67-6
SPID(S): ES211_48_48
M4ID: 42676134

HILL MODEL: Not applicable.

GAIN-LOSS MODEL: Not applicable.

	CNST	HILL	GNLS
AIC:	274.93	NA	NA
PROB:	1	NA	NA
RMSE:	10.36	NA	NA

MAX_MEAN: -4.47 MAX_MED: -2.57 BMAD: 6.29

COFF: 18.9 HIT-CALL: 0 FITC: 4 ACTP: 0

FLAGS:

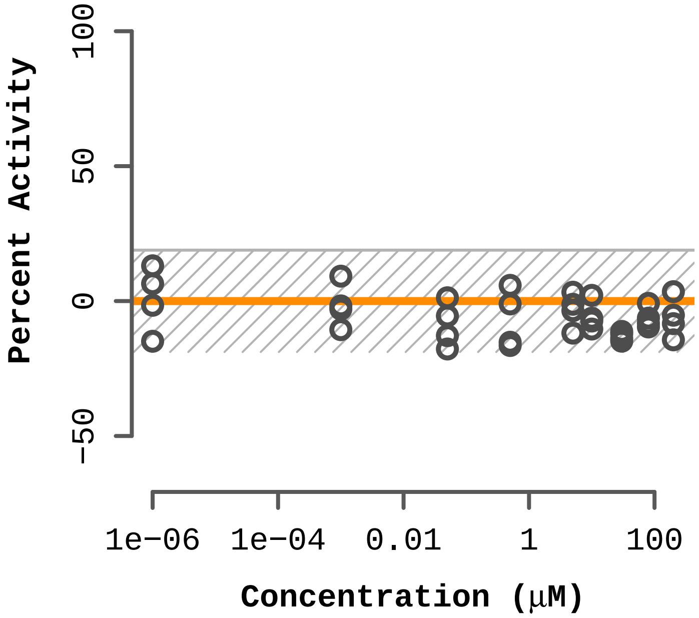

ASSAY: AEID3097 (CCTE_Deisenroth_DEVTOX_RUES2-GLR_Cel[illegible]

NAME: 1,2-Propylene glycol
CHID: 21206 CASRN: 57-55-6
SPID(S): ES211_1_1
M4ID: 42676146

HILL MODEL: Not applicable.

GAIN-LOSS MODEL: Not applicable.

	CNST	HILL	GNLS
AIC:	271.37	NA	NA
PROB:	1	NA	NA
RMSE:	9.53	NA	NA

MAX_MEAN: 0.777 MAX_MED: 2.45 BMAD: 6.29

COFF: 18.9 HIT-CALL: 0 FITC: 4 ACTP: 0

FLAGS:

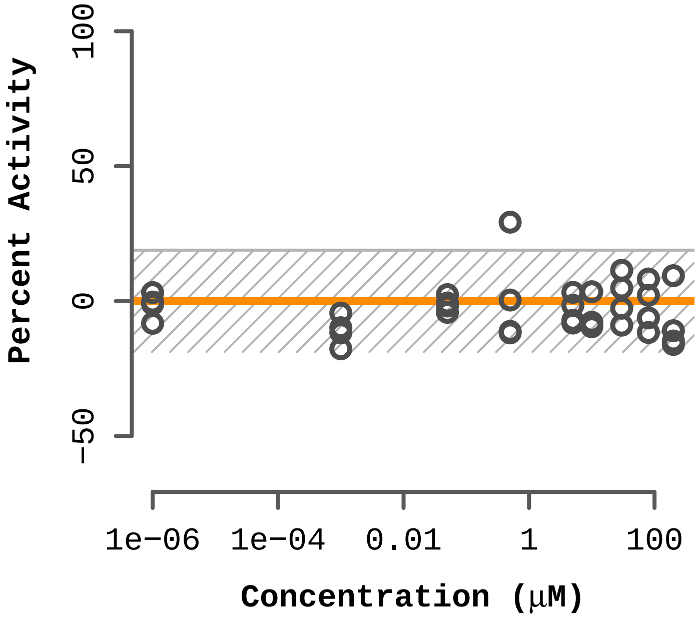

ASSAY: AEID3097 (CCTE_Deisenroth_DEVTOX_RUES2-GLR_Cel

NAME: 6−Propyl−2−thiouracil
CHID: 21209 CASRN: 51−52−5
SPID(S): ES211_8_8
M4ID: 42676151

HILL MODEL: Not applicable.

GAIN−LOSS MODEL: Not applicable.

	CNST	HILL	GNLS
AIC:	268.35	NA	NA
PROB:	1	NA	NA
RMSE:	9.63	NA	NA

MAX_MEAN: 1.66 MAX_MED: 1.1 BMAD: 6.29

COFF: 18.9 HIT−CALL: 0 FITC: 4 ACTP: 0

FLAGS:

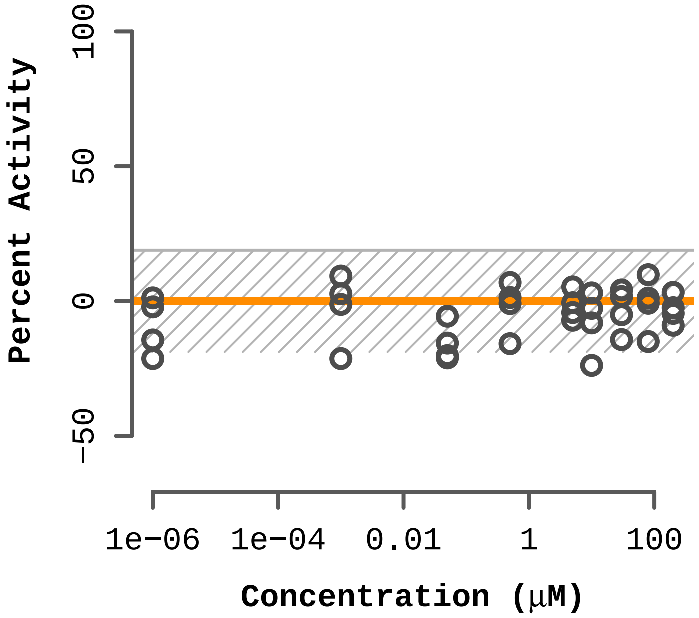

ASSAY: AEID3097 (CCTE_Deisenroth_DEVTOX_RUES2-GLR_Cel

NAME: Saccharin
CHID: 21251 CASRN: 81−07−2
SPID(S): ES211_54_54
M4ID: 42676125

HILL MODEL: Not applicable.

GAIN−LOSS MODEL: Not applicable.

	CNST	HILL	GNLS
AIC:	274.86	NA	NA
PROB:	1	NA	NA
RMSE:	10.64	NA	NA

MAX_MEAN: −1.22 MAX_MED: 0.748 BMAD: 6.29

COFF: 18.9 HIT−CALL: 0 FITC: 4 ACTP: 0

FLAGS:

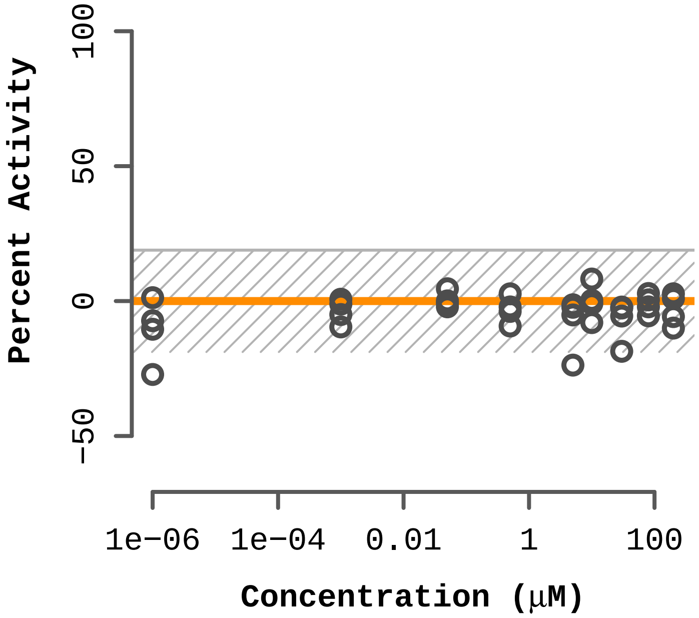

ASSAY: AEID3097 (CCTE_Deisenroth_DEVTOX_RUES2-GLR_Cel

NAME: Sulfasalazine
CHID: 21256 CASRN: 599-79-1
SPID(S): ES211_59_59
M4ID: 42676138

HILL MODEL: Not applicable.

GAIN-LOSS MODEL: Not applicable.

	CNST	HILL	GNLS
AIC:	247.51	NA	NA
PROB:	1	NA	NA
RMSE:	8.26	NA	NA

MAX_MEAN: 0.441 MAX_MED: -0.331 BMAD: 6.29

COFF: 18.9 HIT-CALL: 0 FITC: 4 ACTP: 0

FLAGS:

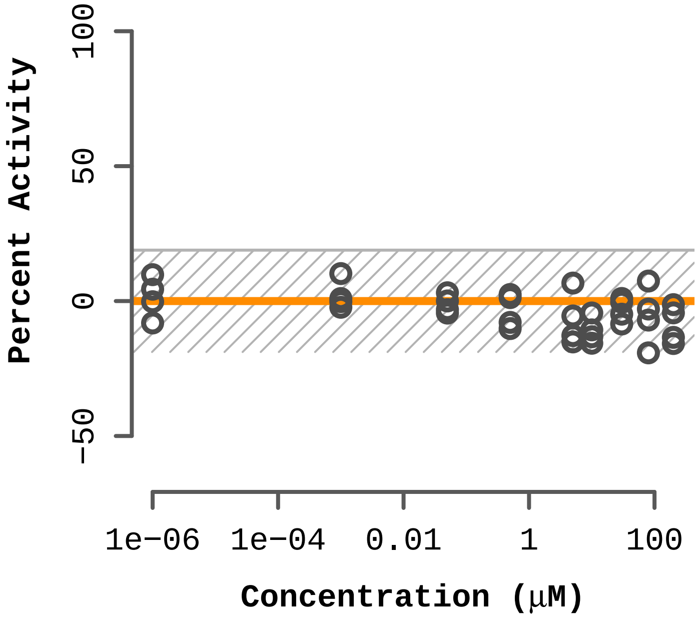

ASSAY: AEID3097 (CCTE_Deisenroth_DEVTOX_RUES2-GLR_Cel

NAME: Dimethyl phthalate
CHID: 22455 CASRN: 131-11-3
SPID(S): ES211_30_30
M4ID: 42676122

HILL MODEL: Not applicable.

GAIN-LOSS MODEL: Not applicable.

	CNST	HILL	GNLS
AIC:	260.09	NA	NA
PROB:	1	NA	NA
RMSE:	8.42	NA	NA

MAX_MEAN: 2.16 MAX_MED: 2.08 BMAD: 6.29

COFF: 18.9 HIT-CALL: 0 FITC: 4 ACTP: 0

FLAGS:

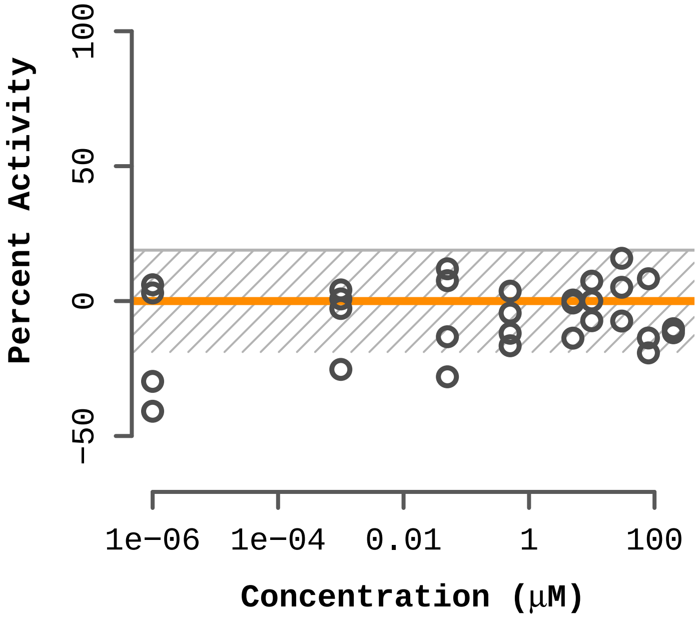

ASSAY: AEID3097 (CCTE_Deisenroth_DEVTOX_RUES2-GLR_Cel

NAME: Folic acid
CHID: 22519 CASRN: 59-30-3
SPID(S): ES211_33_33
M4ID: 42676130

HILL MODEL: Not applicable.

GAIN-LOSS MODEL: Not applicable.

	CNST	HILL	GNLS
AIC:	255.12	NA	NA
PROB:	1	NA	NA
RMSE:	14.46	NA	NA

MAX_MEAN: 4.53 MAX_MED: 5.12 BMAD: 6.29

COFF: 18.9 HIT-CALL: 0 FITC: 4 ACTP: 0

FLAGS:

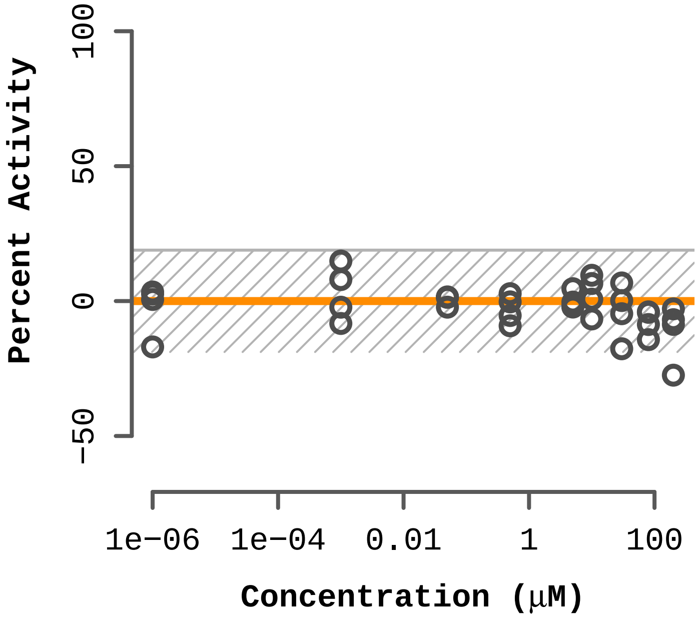

ASSAY: AEID3097 (CCTE_Deisenroth_DEVTOX_RUES2-GLR_Cel

NAME: Acyclovir
CHID: 22556 CASRN: 59277-89-3
SPID(S): ES211_12_12
M4ID: 42676113

HILL MODEL: Not applicable.

GAIN-LOSS MODEL: Not applicable.

	CNST	HILL	GNLS
AIC:	254.61	NA	NA
PROB:	1	NA	NA
RMSE:	8.47	NA	NA

MAX_MEAN: 3.07 MAX_MED: 3.62 BMAD: 6.29

COFF: 18.9 HIT-CALL: 0 FITC: 4 ACTP: 0

FLAGS:

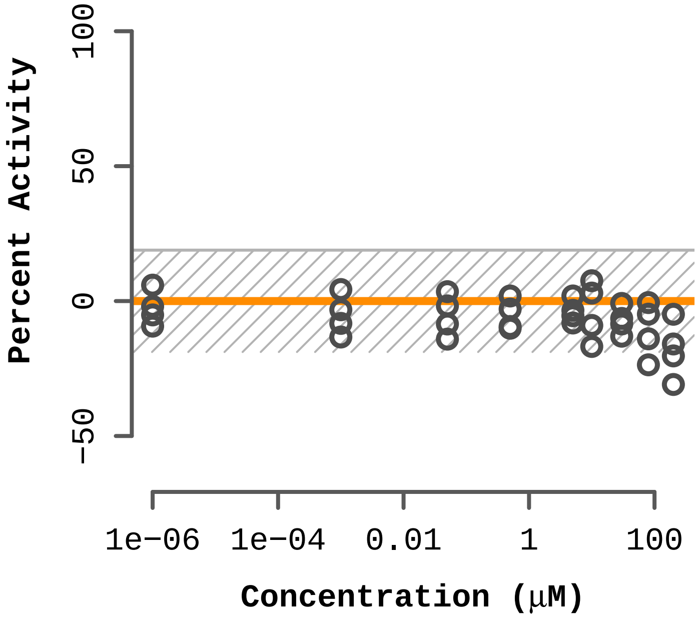

ASSAY: AEID3097 (CCTE_Deisenroth_DEVTOX_RUES2-GLR_Cel

NAME: Valproic acid
CHID: 23733 CASRN: 99-66-1
SPID(S): ES211_63_63
M4ID: 42676171

HILL MODEL: Not applicable.

GAIN-LOSS MODEL: Not applicable.

	CNST	HILL	GNLS
AIC:	275.23	NA	NA
PROB:	1	NA	NA
RMSE:	10.75	NA	NA

MAX_MEAN: -2.6 MAX_MED: -3.02 BMAD: 6.29

COFF: 18.9 HIT-CALL: 0 FITC: 4 ACTP: 0

FLAGS:

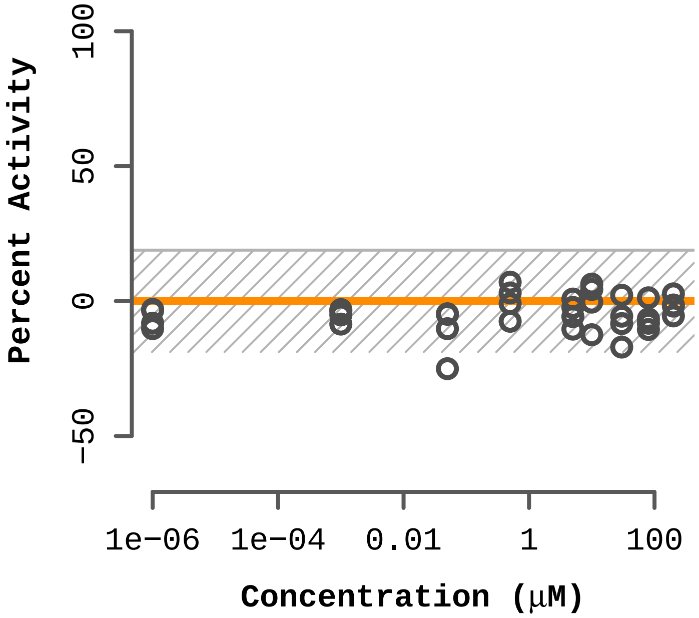

ASSAY: AEID3097 (CCTE_Deisenroth_DEVTOX_RUES2-GLR_Cel

NAME: D-Camphor
CHID: 24721 CASRN: 464-49-3
SPID(S): ES211_27_27
M4ID: 42676128

HILL MODEL: Not applicable.

GAIN-LOSS MODEL: Not applicable.

	CNST	HILL	GNLS
AIC:	252.32	NA	NA
PROB:	1	NA	NA
RMSE:	7.87	NA	NA

MAX_MEAN: 0.405 MAX_MED: 1.97 BMAD: 6.29

COFF: 18.9 HIT-CALL: 0 FITC: 4 ACTP: 0

FLAGS:

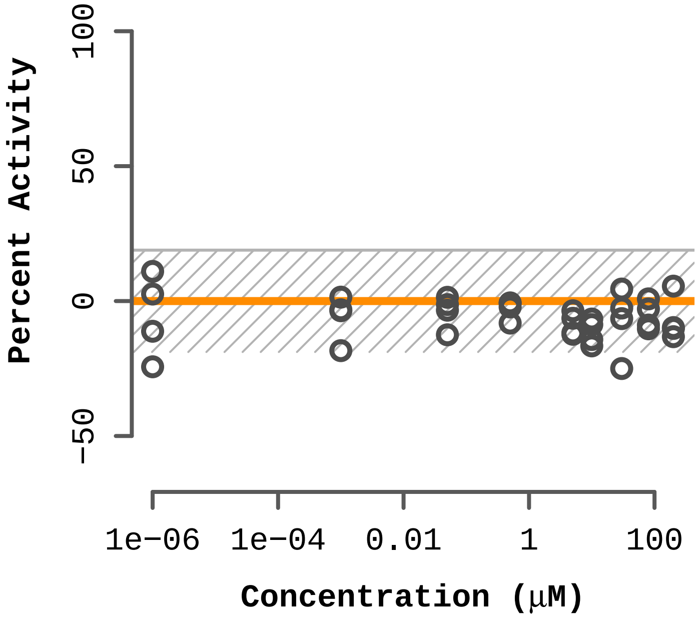

ASSAY: AEID3097 (CCTE_Deisenroth_DEVTOX_RUES2-GLR_Cel

NAME: Salicylic acid
CHID: 26368 CASRN: 69-72-7
SPID(S): ES211_55_55
M4ID: 42676174

HILL MODEL: Not applicable.

GAIN-LOSS MODEL: Not applicable.

	CNST	HILL	GNLS
AIC:	272.2	NA	NA
PROB:	1	NA	NA
RMSE:	10.11	NA	NA

MAX_MEAN: -2.94 MAX_MED: -1.48 BMAD: 6.29

COFF: 18.9 HIT-CALL: 0 FITC: 4 ACTP: 0

FLAGS:

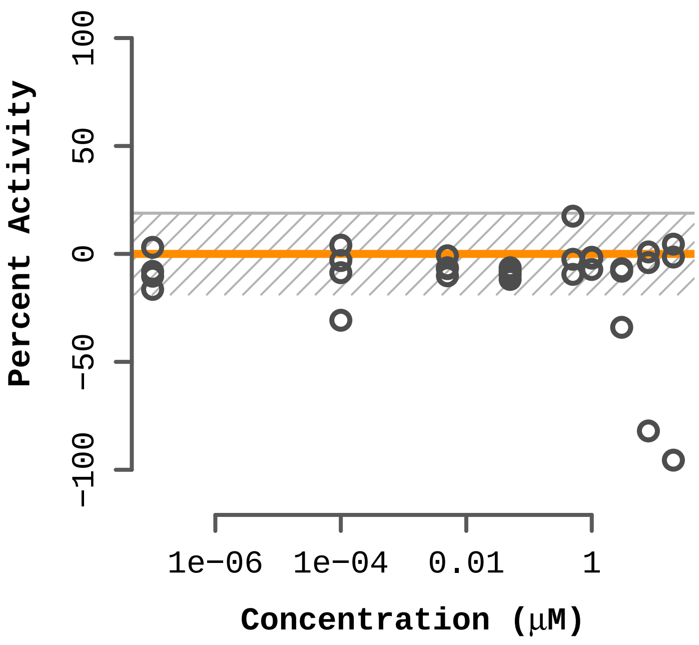

ASSAY: AEID3097 (CCTE_Deisenroth_DEVTOX_RUES2-GLR_Cel

NAME: Cyclopamine
CHID: 43709 CASRN: 4449-51-8
SPID(S): ES211_23_23
M4ID: 42676156

HILL MODEL: Not applicable.

GAIN-LOSS MODEL: Not applicable.

	CNST	HILL	GNLS
AIC:	266.73	NA	NA
PROB:	1	NA	NA
RMSE:	25.17	NA	NA

MAX_MEAN: 1.86 MAX_MED: -1.44 BMAD: 6.29

COFF: 18.9 HIT-CALL: 0 FITC: 4 ACTP: 0

FLAGS: 10

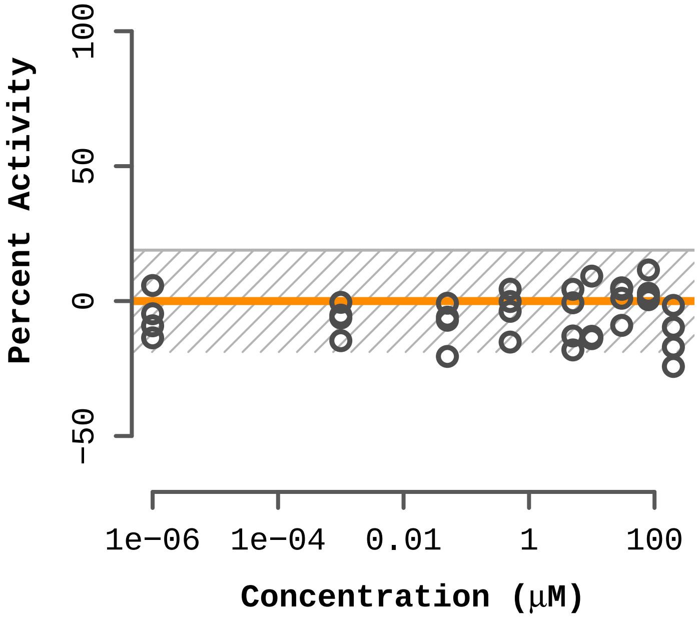

ASSAY: AEID3097 (CCTE_Deisenroth_DEVTOX_RUES2-GLR_Cel

NAME: Penicillin G potassium
CHID: 45106 CASRN: 113−98−4
SPID(S): ES211_49_49
M4ID: 42676168

HILL MODEL: Not applicable.

GAIN−LOSS MODEL: Not applicable.

	CNST	HILL	GNLS
AIC:	274.19	NA	NA
PROB:	1	NA	NA
RMSE:	10.23	NA	NA

MAX_MEAN: 4.27 MAX_MED: 2.71 BMAD: 6.29

COFF: 18.9 HIT−CALL: 0 FITC: 4 ACTP: 0

FLAGS:

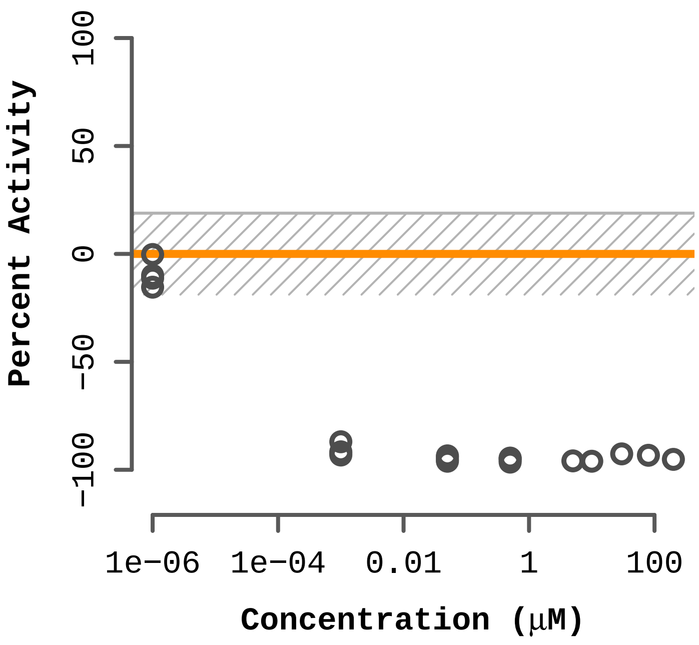

ASSAY: AEID3097 (CCTE_Deisenroth_DEVTOX_RUES2-GLR_Cel

NAME: Actinomycin D
CHID: 20031 CASRN: 50−76−0
SPID(S): ES211_11_11
M4ID: 42676175

HILL MODEL: Failed to converge.

GAIN−LOSS MODEL: Failed to converge.

	CNST	HILL	GNLS
AIC:	327.56	NA	NA
PROB:	1	NA	NA
RMSE:	84.69	NA	NA

MAX_MEAN: −9.2 MAX_MED: −10.6 BMAD: 6.29

COFF: 18.9 HIT−CALL: 0 FITC: 7 ACTP: 0

FLAGS: 10

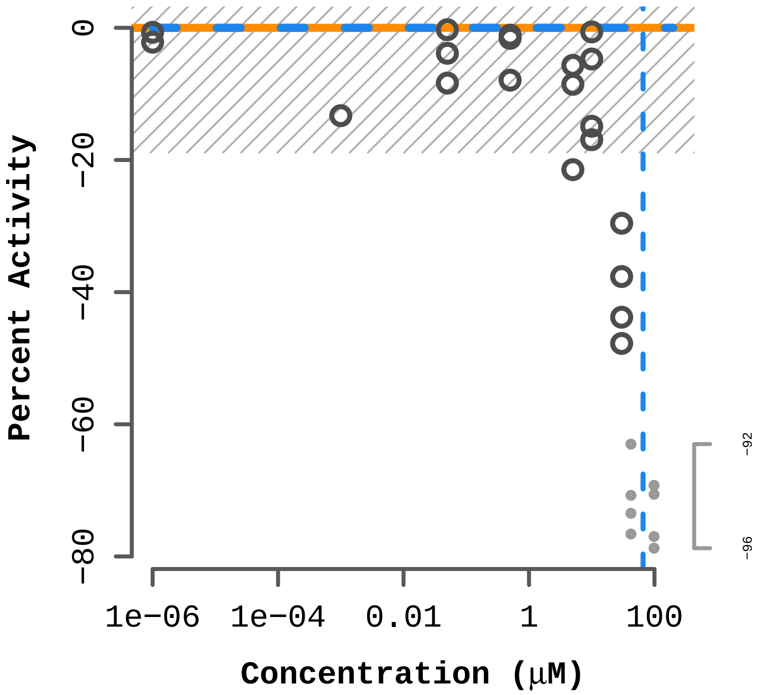

ASSAY: AEID3097 (CCTE_Deisenroth_DEVTOX_RUES2-GLR_Cel

NAME: Bisphenol A
CHID: 20182 CASRN: 80-05-7
SPID(S): ES211_16_16
M4ID: 42676166 BRK

HILL MODEL (in red):

	tp	ga	gw
val:	3.62e-05	2.76	5.04
sd:	NaN	NaN	NaN

GAIN-LOSS MODEL (in blue):

	tp	ga	gw	la	lw
val:	5.56e-08	1.82	5.9	2.32	8.88
sd:	NaN	NaN	NaN	NaN	NaN

	CNST	HILL	GNLS
AIC:	378.61	384.61	388.61
PROB:	0.95	0.05	0.01
RMSE:	47.32	47.32	47.32

MAX_MEAN: 9.39 MAX_MED: 8.13 BMAD: 6.29

COFF: 18.9 HIT-CALL: 0 FITC: 7 ACTP: 0.05

FLAGS: 10

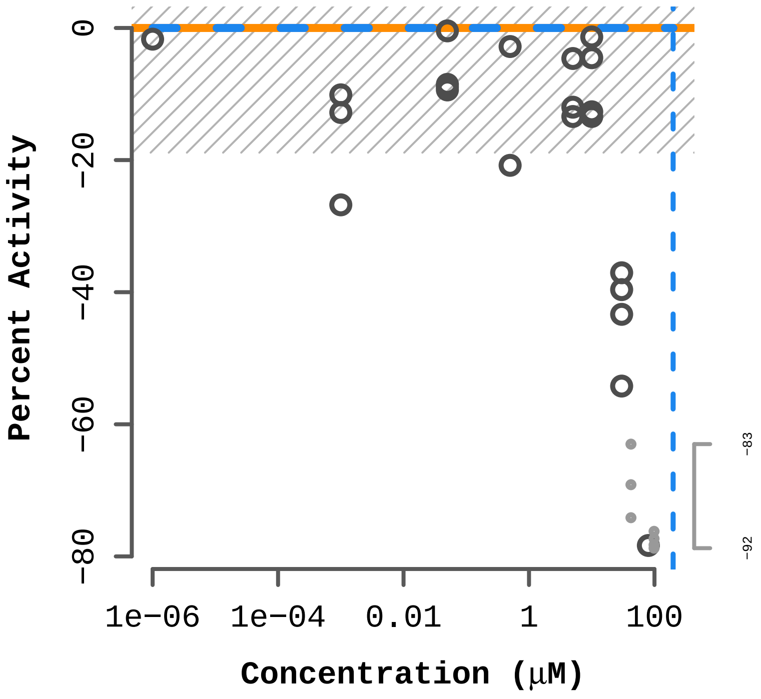

ASSAY: AEID3097 (CCTE_Deisenroth_DEVTOX_RUES2-GLR_Cel

NAME: 4-Hydroxybenzoic acid butyl ester
CHID: 20209 CASRN: 94-26-8
SPID(S): ES211_19_19
M4ID: 42676150 BRK

HILL MODEL (in red):

	tp	ga	gw
val:	0.0225	2.8	7.72
sd:	NaN	NaN	NaN

GAIN-LOSS MODEL (in blue):

	tp	ga	gw	la	lw
val:	2.73e-09	2.3	7.37	3.03	6.11
sd:	NA	NA	NA	NA	NA

	CNST	HILL	GNLS
AIC:	376.27	382.27	386.27
PROB:	0.95	0.05	0.01
RMSE:	45.14	45.14	45.14

MAX_MEAN: 8.92 MAX_MED: 8.22 BMAD: 6.29

COFF: 18.9 HIT-CALL: 0 FITC: 7 ACTP: 0.05

FLAGS: 10

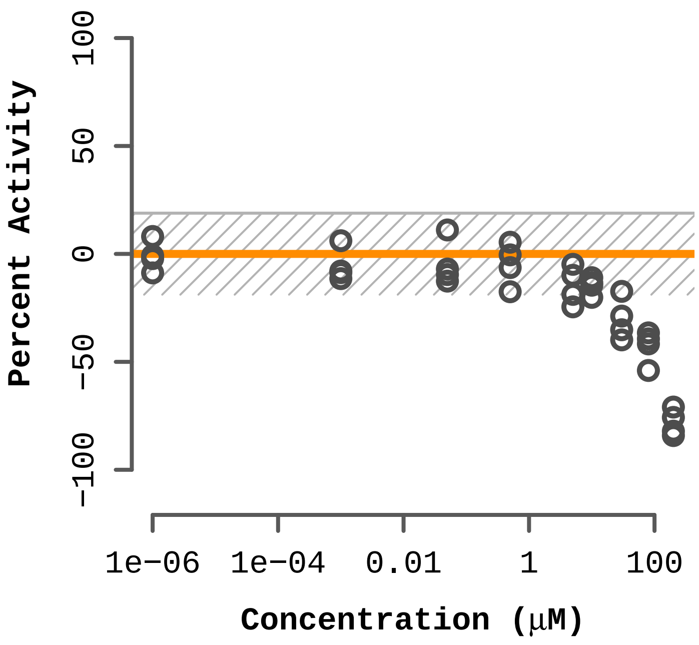

ASSAY: AEID3097 (CCTE_Deisenroth_DEVTOX_RUES2-GLR_Cel

NAME: Diphenhydramine hydrochloride
CHID: 20537 CASRN: 147-24-0
SPID(S): ES211_31_31
M4ID: 42676136

HILL MODEL: Failed to converge.

GAIN-LOSS MODEL: Failed to converge.

	CNST	HILL	GNLS
AIC:	352.61	NA	NA
PROB:	1	NA	NA
RMSE:	33.04	NA	NA

MAX_MEAN: -0.907 MAX_MED: -1.47 BMAD: 6.29

COFF: 18.9 HIT-CALL: 0 FITC: 7 ACTP: 0

FLAGS: 10

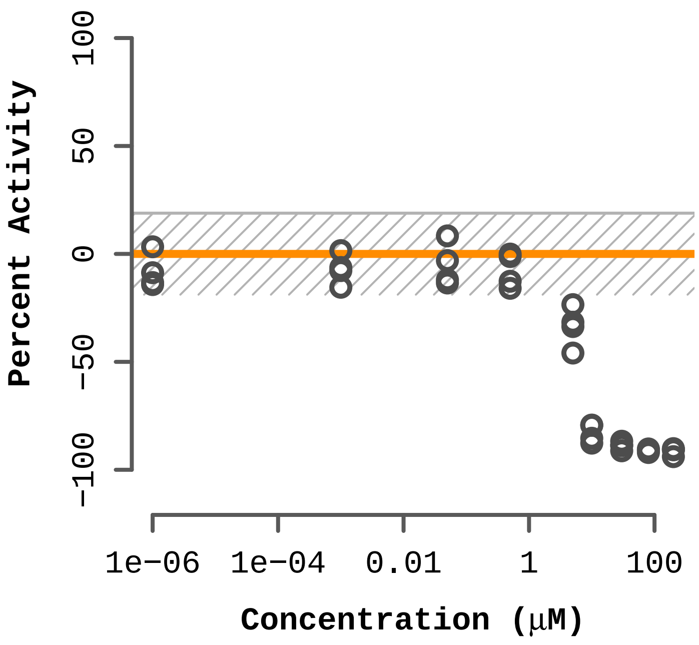

ASSAY: AEID3097 (CCTE_Deisenroth_DEVTOX_RUES2-GLR_Cel

NAME: 5-Fluorouracil
CHID: 20634 CASRN: 51-21-8
SPID(S): ES211_5_5
M4ID: 42676169

HILL MODEL: Failed to converge.

GAIN-LOSS MODEL: Failed to converge.

	CNST	HILL	GNLS
AIC:	403.55	NA	NA
PROB:	1	NA	NA
RMSE:	60.58	NA	NA

MAX_MEAN: -5.03 MAX_MED: -6.93 BMAD: 6.29

COFF: 18.9 HIT-CALL: 0 FITC: 7 ACTP: 0

FLAGS: 10

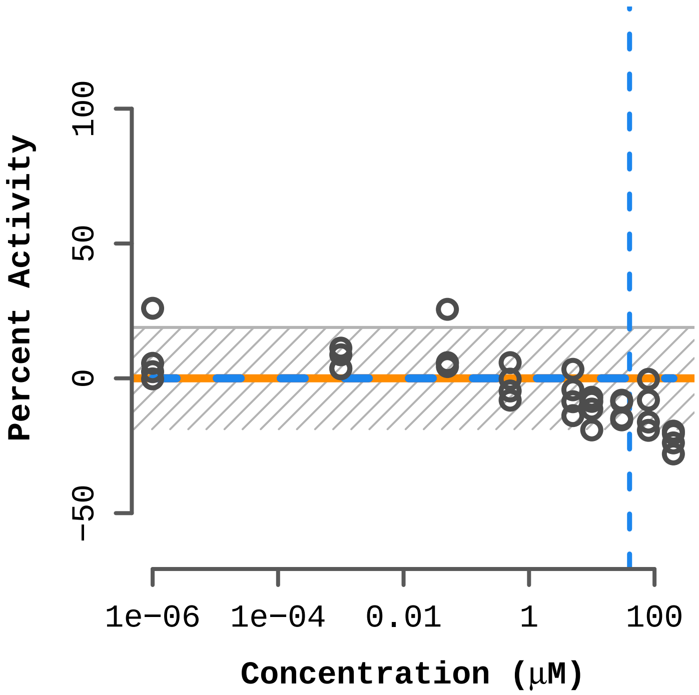

ASSAY: AEID3097 (CCTE_Deisenroth_DEVTOX_RUES2-GLR_Cel

NAME: Ibuprofen
CHID: 20732 CASRN: 15687-27-1
SPID(S): ES211_36_36
M4ID: 42676158

HILL MODEL (in red):

	tp	ga	gw
val:	0.0788	2.8	7.97
sd:	NaN	NaN	NaN

GAIN-LOSS MODEL (in blue):

	tp	ga	gw	la	lw
val:	3.39e-08	1.6	6.99	1.86	9.04
sd:	0.000131	2270	122000	966	173000

	CNST	HILL	GNLS
AIC:	291.97	297.97	301.97
PROB:	0.95	0.05	0.01
RMSE:	13.09	13.09	13.09

MAX_MEAN: 10.4 MAX_MED: 6.24 BMAD: 6.29

COFF: 18.9 HIT-CALL: 0 FITC: 7 ACTP: 0.05

FLAGS:

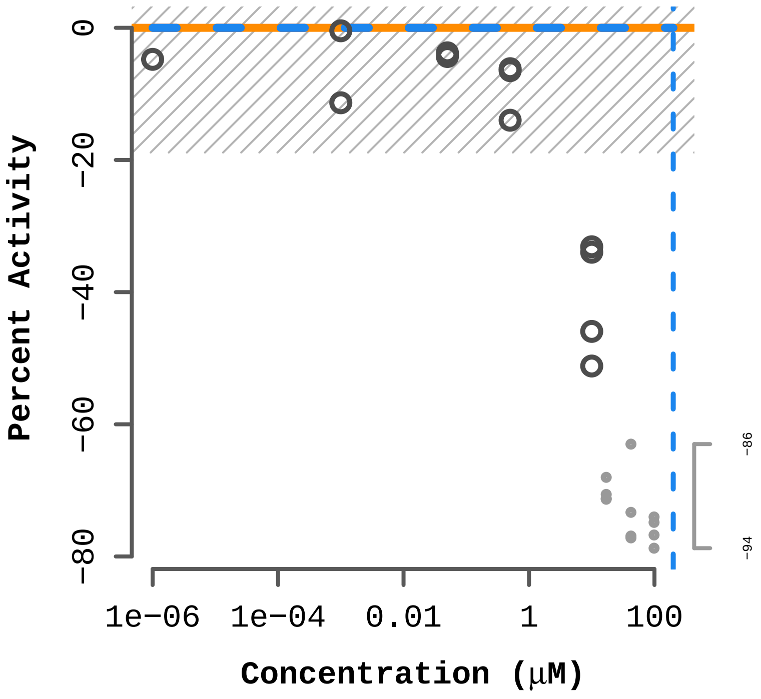

ASSAY: AEID3097 (CCTE_Deisenroth_DEVTOX_RUES2-GLR_Cel

NAME: 1-(p-Chlorobenzoyl)-5-methoxy-2-methyl-Indole-
CHID: 20740 CASRN: 53-86-1
SPID(S): ES211_38_38
M4ID: 42676114 BRK

HILL MODEL (in red):

	tp	ga	gw
val:	0.0031	2.79	6.83
sd:	NaN	NaN	NaN

GAIN-LOSS MODEL (in blue):

	tp	ga	gw	la	lw
val:	2.63e-07	2.3	7.02	2.69	0.444
sd:	NaN	NaN	NaN	NaN	NaN

	CNST	HILL	GNLS
AIC:	393.92	399.92	403.92
PROB:	0.95	0.05	0.01
RMSE:	54.85	54.85	54.85

MAX_MEAN: 7.75 MAX_MED: 8.22 BMAD: 6.29

COFF: 18.9 HIT-CALL: 0 FITC: 7 ACTP: 0.05

FLAGS: 10

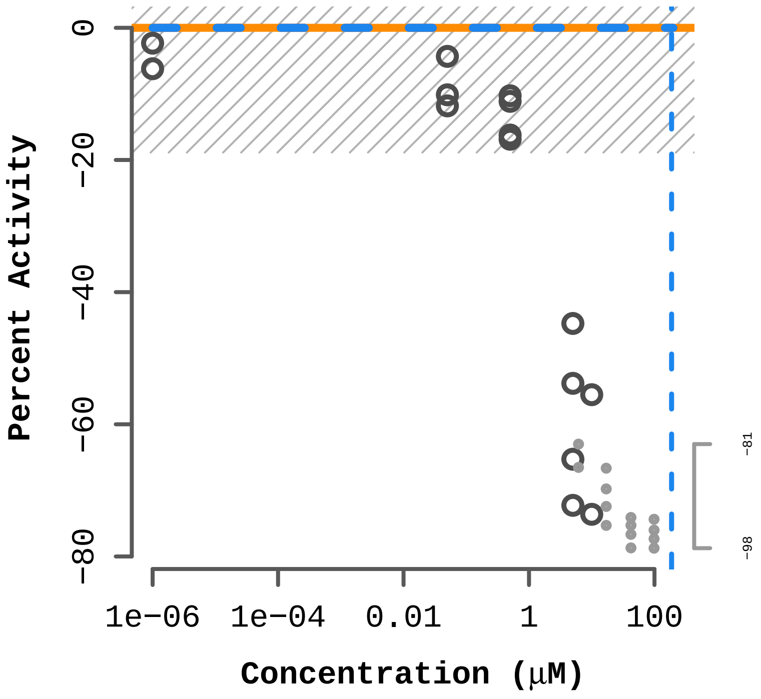

ASSAY: AEID3097 (CCTE_Deisenroth_DEVTOX_RUES2-GLR_Cel

NAME: Lovastatin
CHID: 20784 CASRN: 75330-75-5
SPID(S): ES211_42_42
M4ID: 42676162 BRK

HILL MODEL (in red):

	tp	ga	gw
val:	2.95e-06	2.74	3.79
sd:	NaN	NaN	NaN

GAIN-LOSS MODEL (in blue):

	tp	ga	gw	la	lw
val:	7.57e-08	2.27	3.69	3.02	1.15
sd:	NaN	NaN	NaN	NaN	NaN

	CNST	HILL	GNLS
AIC:	406.85	412.85	416.85
PROB:	0.95	0.05	0.01
RMSE:	62.93	62.93	62.93

MAX_MEAN: 5.38 MAX_MED: 3.28 BMAD: 6.29

COFF: 18.9 HIT-CALL: 0 FITC: 7 ACTP: 0.05

FLAGS: 10

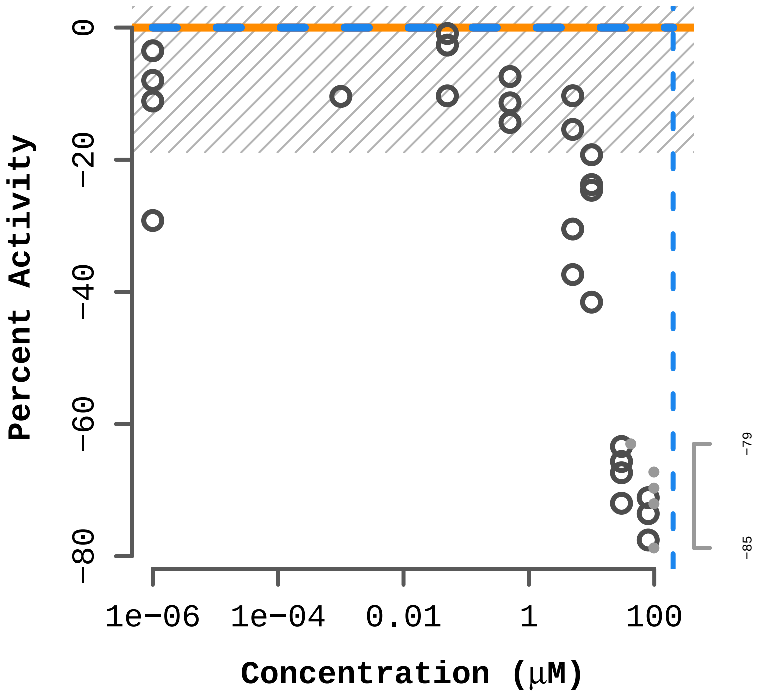

ASSAY: AEID3097 (CCTE_Deisenroth_DEVTOX_RUES2-GLR_Cel

NAME: Busulfan
CHID: 20910 CASRN: 55−98−1
SPID(S): ES211_18_18
M4ID: 42676142 BRK

HILL MODEL (in red):

	tp	ga	gw
val:	0.000557	2.77	6.36
sd:	NaN	NaN	NaN

GAIN−LOSS MODEL (in blue):

	tp	ga	gw	la	lw
val:	4.39e−08	2.3	6.92	4.24	4.77
sd:	NA	NA	NA	NA	NA

	CNST	HILL	GNLS
AIC:	382.06	388.06	392.06
PROB:	0.95	0.05	0.01
RMSE:	46.1	46.1	46.1

MAX_MEAN: 5.76 MAX_MED: 7.6 BMAD: 6.29

COFF: 18.9 HIT−CALL: 0 FITC: 7 ACTP: 0.05

FLAGS: 10

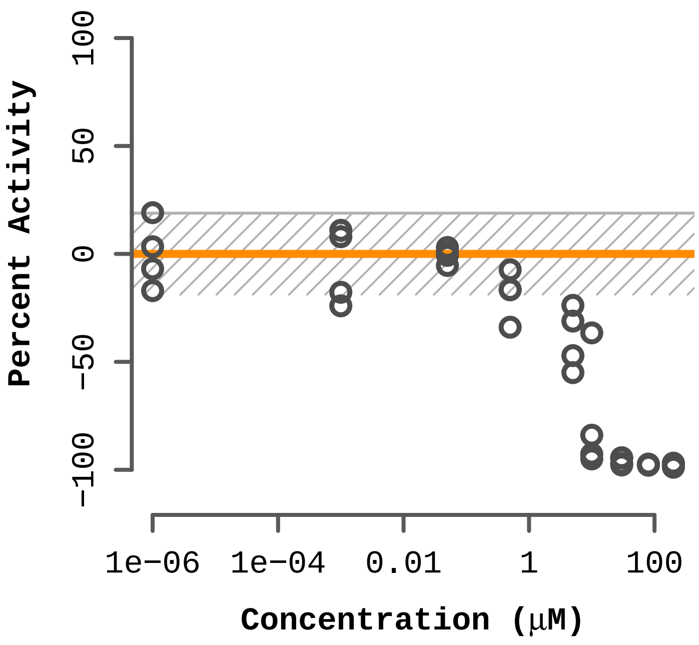

ASSAY: AEID3097 (CCTE_Deisenroth_DEVTOX_RUES2-GLR_Cel

NAME: all-trans-Retinoic acid
CHID: 21239 CASRN: 302-79-4
SPID(S): ES211_13_13
M4ID: 42676110

HILL MODEL: Failed to converge.

GAIN-LOSS MODEL: Failed to converge.

	CNST	HILL	GNLS
AIC:	408	NA	NA
PROB:	1	NA	NA
RMSE:	64.44	NA	NA

MAX_MEAN: -0.355 MAX_MED: -0.00578 BMAD: 6.29

COFF: 18.9 HIT-CALL: 0 FITC: 7 ACTP: 0

FLAGS: 10

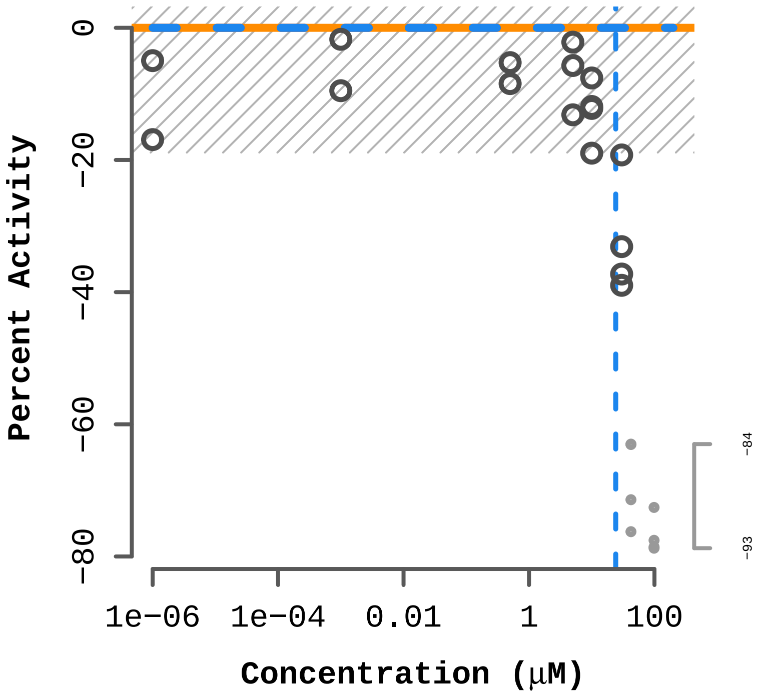

ASSAY: AEID3097 (CCTE_Deisenroth_DEVTOX_RUES2-GLR_Cel

NAME: Rifampicin
CHID: 21244 CASRN: 13292-46-1
SPID(S): ES211_52_52
M4ID: 42676167 BRK

HILL MODEL (in red):

	tp	ga	gw
val:	7.13e-06	2.79	4.41
sd:	NaN	NaN	NaN

GAIN-LOSS MODEL (in blue):

	tp	ga	gw	la	lw
val:	1.82e-09	1.38	4.67	1.88	10.2
sd:	NaN	NaN	NaN	NaN	NaN

	CNST	HILL	GNLS
AIC:	372.07	378.07	382.07
PROB:	0.95	0.05	0.01
RMSE:	44.16	44.16	44.16

MAX_MEAN: 7.56 MAX_MED: 5.58 BMAD: 6.29

COFF: 18.9 HIT-CALL: 0 FITC: 7 ACTP: 0.05

FLAGS: 10

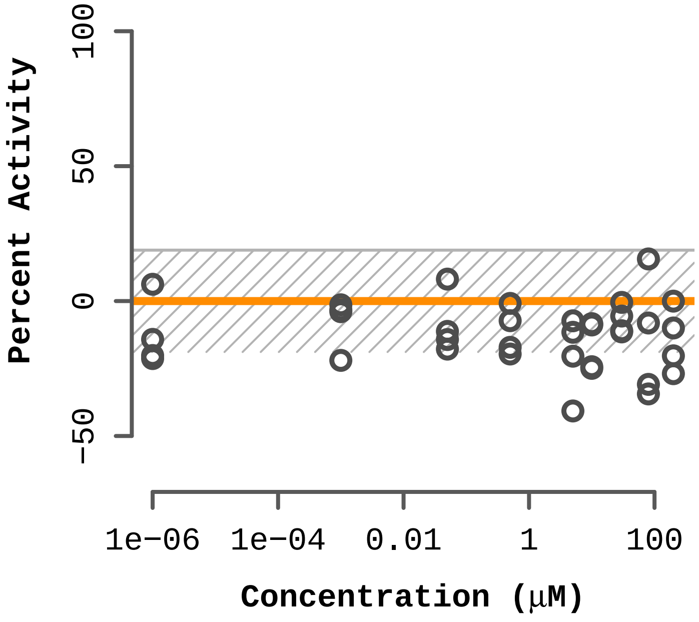

ASSAY: AEID3097 (CCTE_Deisenroth_DEVTOX_RUES2-GLR_Cel

NAME: Thalidomide
CHID: 22524 CASRN: 50-35-1
SPID(S): ES211_62_62
M4ID: 42676115

HILL MODEL: Failed to converge.

GAIN-LOSS MODEL: Failed to converge.

	CNST	HILL	GNLS
AIC:	312.26	NA	NA
PROB:	1	NA	NA
RMSE:	17.19	NA	NA

MAX_MEAN: -7.11 MAX_MED: -3.59 BMAD: 6.29

COFF: 18.9 HIT-CALL: 0 FITC: 7 ACTP: 0

FLAGS:

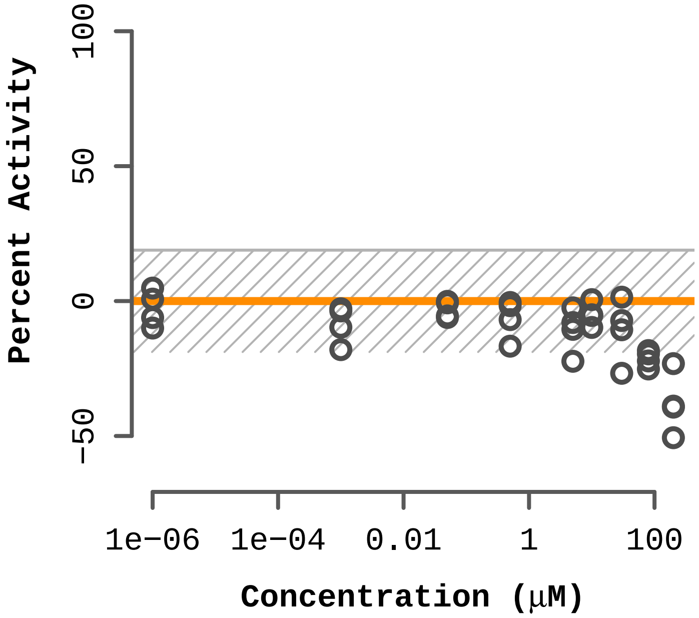

ASSAY: AEID3097 (CCTE_Deisenroth_DEVTOX_RUES2-GLR_Cel

NAME: Carbamazepine
CHID: 22731 CASRN: 298−46−4
SPID(S): ES211_21_21
M4ID: 42676145

HILL MODEL: Failed to converge.

GAIN−LOSS MODEL: Failed to converge.

	CNST	HILL	GNLS
AIC:	306.42	NA	NA
PROB:	1	NA	NA
RMSE:	17.24	NA	NA

MAX_MEAN: −2.64 MAX_MED: −2.69 BMAD: 6.29

COFF: 18.9 HIT−CALL: 0 FITC: 7 ACTP: 0

FLAGS:

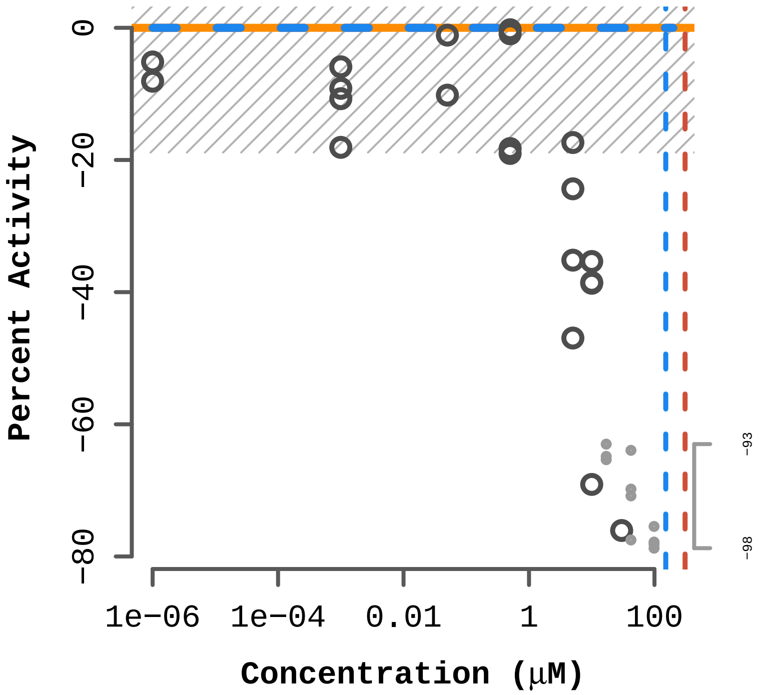

ASSAY: AEID3097 (CCTE_Deisenroth_DEVTOX_RUES2-GLR_Cel

NAME: 13-cis Retinoic acid
CHID: 23177 CASRN: 4759-48-2
SPID(S): ES211_2_2
M4ID: 42676120 BRK

HILL MODEL (in red):

	tp	ga	gw
val:	1.5e-07	2.49	1.51
sd:	NaN	NaN	NaN

GAIN-LOSS MODEL (in blue):

	tp	ga	gw	la	lw
val:	1.84e-08	2.18	1.75	2.7	5.95
sd:	NaN	NaN	NaN	NaN	NaN

	CNST	HILL	GNLS
AIC:	399.18	405.18	409.18
PROB:	0.95	0.05	0.01
RMSE:	58.18	58.18	58.18

MAX_MEAN: 3.97 MAX_MED: 2.18 BMAD: 6.29

COFF: 18.9 HIT-CALL: 0 FITC: 7 ACTP: 0.05

FLAGS: 10

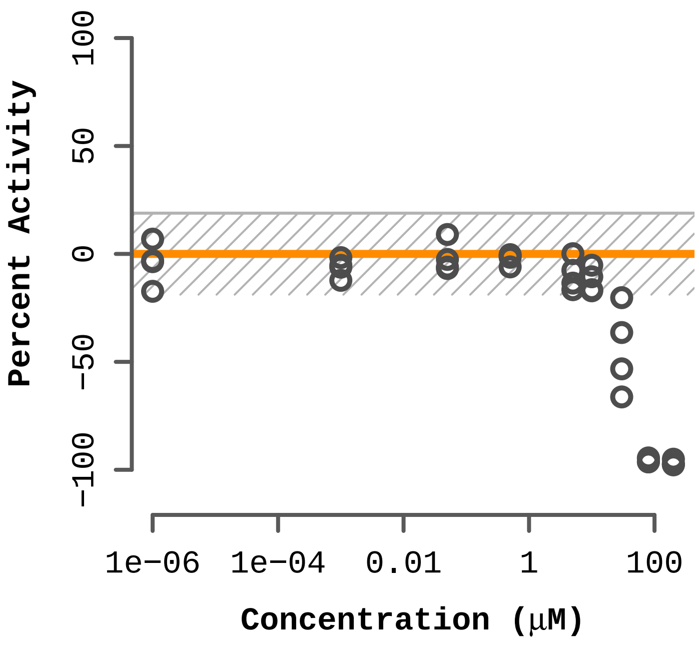

ASSAY: AEID3097 (CCTE_Deisenroth_DEVTOX_RUES2-GLR_Cel

NAME: Retinol
CHID: 23556 CASRN: 68−26−8
SPID(S): ES211_51_51
M4ID: 42676148

HILL MODEL: Failed to converge.

GAIN−LOSS MODEL: Failed to converge.

	CNST	HILL	GNLS
AIC:	379.94	NA	NA
PROB:	1	NA	NA
RMSE:	48.58	NA	NA

MAX_MEAN: −1.59 MAX_MED: −1.2 BMAD: 6.29

COFF: 18.9 HIT−CALL: 0 FITC: 7 ACTP: 0

FLAGS: 10

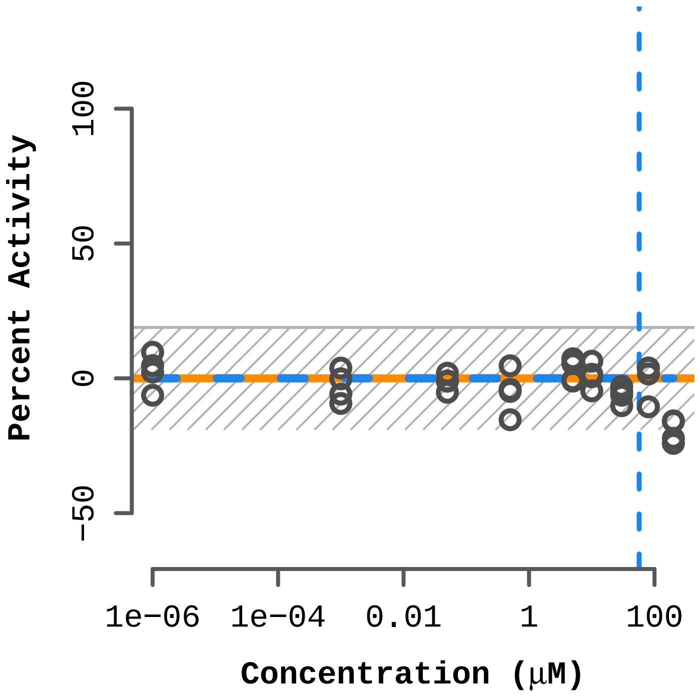

ASSAY: AEID3097 (CCTE_Deisenroth_DEVTOX_RUES2-GLR_Cel

NAME: Warfarin
CHID: 23742 CASRN: 81-81-2
SPID(S): ES211_65_65
M4ID: 42676132

HILL MODEL (in red):

	tp	ga	gw
val:	0.0557	2.79	7.99
sd:	NaN	NaN	NaN

GAIN-LOSS MODEL (in blue):

	tp	ga	gw	la	lw
val:	8.68e-10	1.76	5.08	2.14	3.41
sd:	NaN	NaN	NaN	NaN	NaN

	CNST	HILL	GNLS
AIC:	256.96	262.96	266.96
PROB:	0.95	0.05	0.01
RMSE:	8.62	8.62	8.62

MAX_MEAN: 2.79 MAX_MED: 3.54 BMAD: 6.29

COFF: 18.9 HIT-CALL: 0 FITC: 7 ACTP: 0.05

FLAGS:

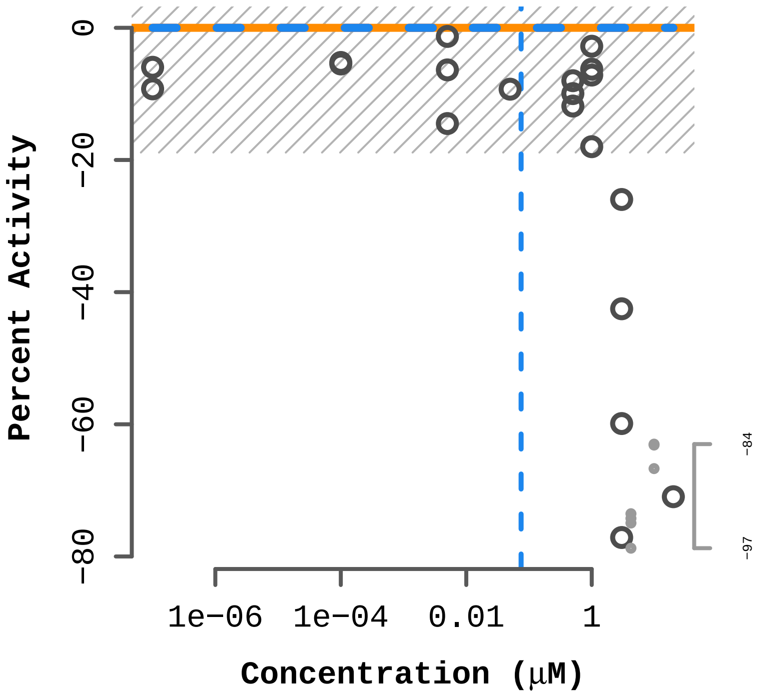

ASSAY: AEID3097 (CCTE_Deisenroth_DEVTOX_RUES2-GLR_Cel

NAME: Ziprasidone
CHID: 23753 CASRN: 146939-27-7
SPID(S): ES211_66_66
M4ID: 42676170 BRK

HILL MODEL (in red):

	tp	ga	gw
val:	2.6e-08	1.79	1.81
sd:	NaN	NaN	NaN

GAIN-LOSS MODEL (in blue):

	tp	ga	gw	la	lw
val:	0.000546	-1.13	3.32	-0.875	5.8
sd:	NaN	NaN	NaN	NaN	NaN

	CNST	HILL	GNLS
AIC:	376.67	382.67	386.67
PROB:	0.95	0.05	0.01
RMSE:	45.98	45.98	45.98

MAX_MEAN: 0.293 MAX_MED: 1.7 BMAD: 6.29

COFF: 18.9 HIT-CALL: 0 FITC: 7 ACTP: 0.05

FLAGS: 10

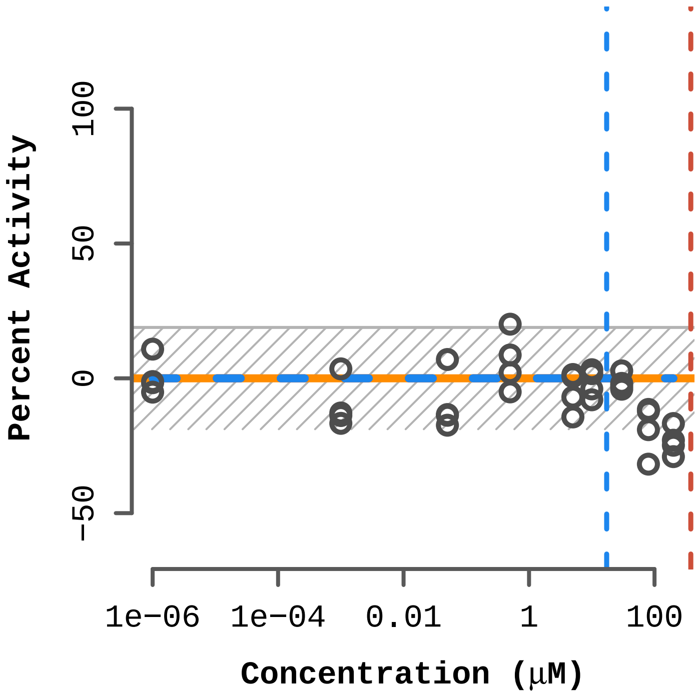

ASSAY: AEID3097 (CCTE_Deisenroth_DEVTOX_RUES2-GLR_Cel

NAME: Stavudine
CHID: 23819 CASRN: 3056-17-5
SPID(S): ES211_58_58
M4ID: 42676137

HILL MODEL (in red):

	tp	ga	gw
val:	7.22e-07	2.58	7.69
sd:	NaN	NaN	NaN

GAIN-LOSS MODEL (in blue):

	tp	ga	gw	la	lw
val:	4e-09	1.24	8	1.5	3.46
sd:	NaN	NaN	NaN	NaN	NaN

	CNST	HILL	GNLS
AIC:	291.98	297.98	301.98
PROB:	0.95	0.05	0.01
RMSE:	13.25	13.25	13.25

MAX_MEAN: 6.46 MAX_MED: 5.39 BMAD: 6.29

COFF: 18.9 HIT-CALL: 0 FITC: 7 ACTP: 0.05

FLAGS:

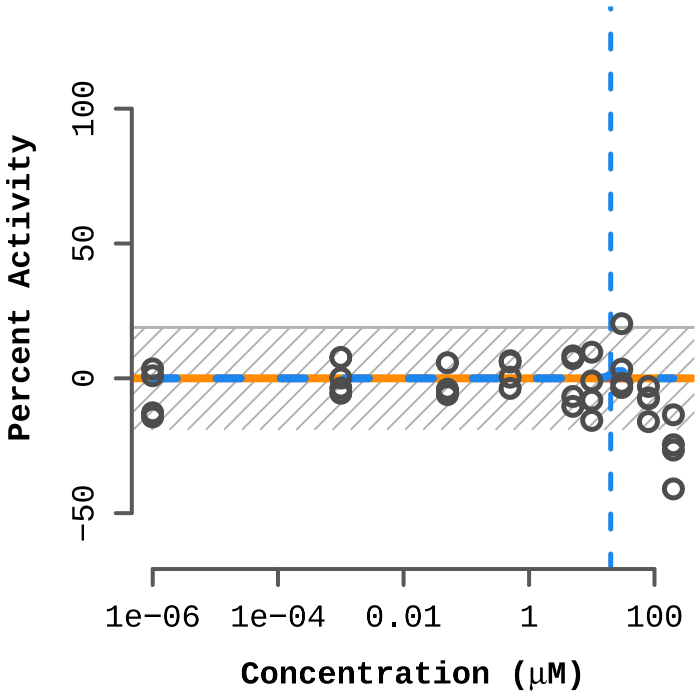

ASSAY: AEID3097 (CCTE_Deisenroth_DEVTOX_RUES2-GLR_Cel

NAME: Cyclophosphamide monohydrate
CHID: 24888 CASRN: 6055-19-2
SPID(S): ES211_24_24
M4ID: 42676140

HILL MODEL (in red):

	tp	ga	gw
val:	3.74e-08	2.74	4.17
sd:	NaN	NaN	NaN

GAIN-LOSS MODEL (in blue):

	tp	ga	gw	la	lw
val:	2.88	1.3	8	1.55	16.9
sd:	21	2.23	56.4	3.33	585

	CNST	HILL	GNLS
AIC:	280.31	286.31	290.02
PROB:	0.95	0.05	0.01
RMSE:	12.2	12.2	12.12

MAX_MEAN: 4.61 MAX_MED: 3.25 BMAD: 6.29

COFF: 18.9 HIT-CALL: 0 FITC: 7 ACTP: 0.05

FLAGS:

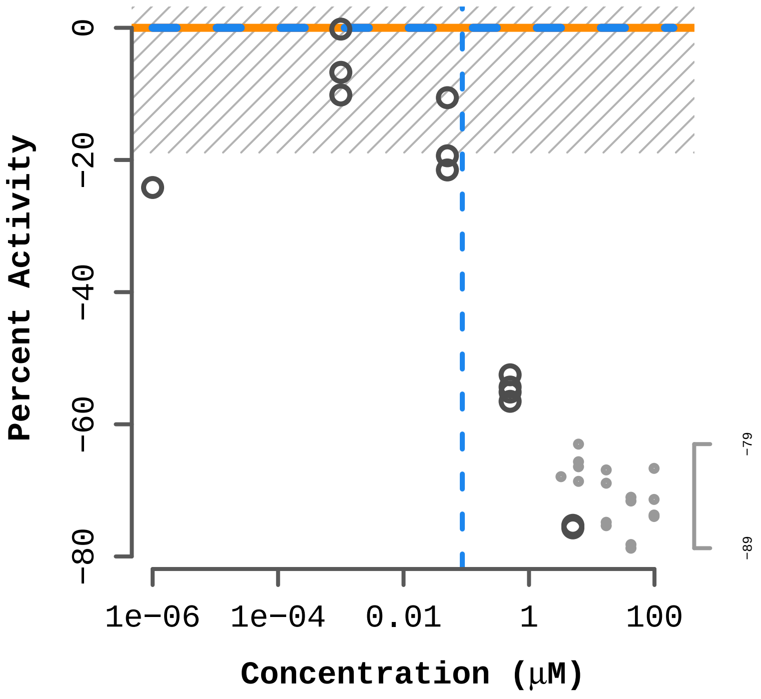

ASSAY: AEID3097 (CCTE_Deisenroth_DEVTOX_RUES2-GLR_Cel

NAME: Cytarabine hydrochloride
CHID: 24891 CASRN: 69-74-9
SPID(S): ES211_25_25
M4ID: 42676157 BRK

HILL MODEL (in red):

	tp	ga	gw
val:	2.35e-06	2.78	4
sd:	NaN	NaN	NaN

GAIN-LOSS MODEL (in blue):

	tp	ga	gw	la	lw
val:	1.22e-06	-1.06	5.75	-0.706	6.24
sd:	NaN	NaN	NaN	NaN	NaN

	CNST	HILL	GNLS
AIC:	410.86	416.86	420.86
PROB:	0.95	0.05	0.01
RMSE:	64.86	64.86	64.86

MAX_MEAN: 0.428 MAX_MED: 3.03 BMAD: 6.29

COFF: 18.9 HIT-CALL: 0 FITC: 7 ACTP: 0.05

FLAGS: 10

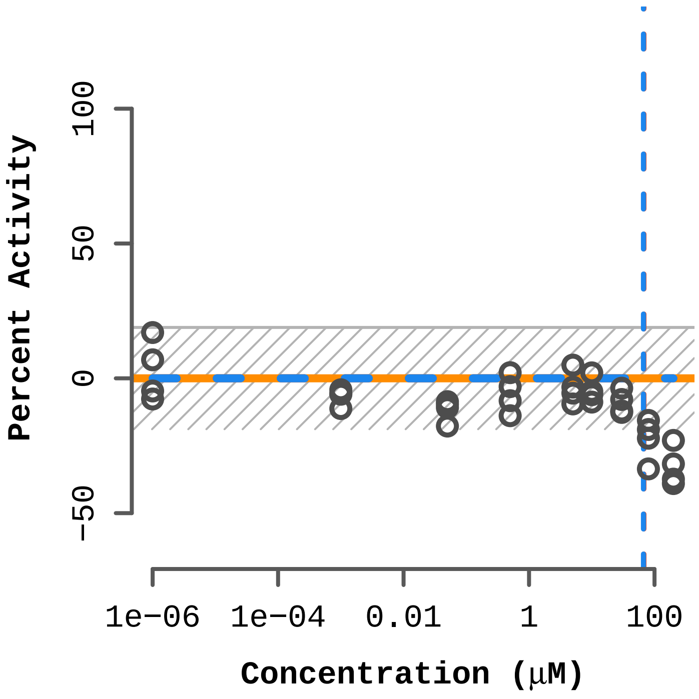

ASSAY: AEID3097 (CCTE_Deisenroth_DEVTOX_RUES2-GLR_Cel

NAME: Hydroxyurea
CHID: 25438 CASRN: 127−07−1
SPID(S): ES211_35_35
M4ID: 42676139

HILL MODEL (in red):

	tp	ga	gw
val:	1.15e−09	1.83	0.72
sd:	4.62e−05	4810	47300

GAIN−LOSS MODEL (in blue):

	tp	ga	gw	la	lw
val:	1.12e−08	1.82	1.7	2.16	5.41
sd:	NaN	NaN	NaN	NaN	NaN

	CNST	HILL	GNLS
AIC:	301.96	307.96	311.96
PROB:	0.95	0.05	0.01
RMSE:	15.68	15.68	15.68

MAX_MEAN: 2.88 MAX_MED: 1.1 BMAD: 6.29

COFF: 18.9 HIT−CALL: 0 FITC: 7 ACTP: 0.05

FLAGS:

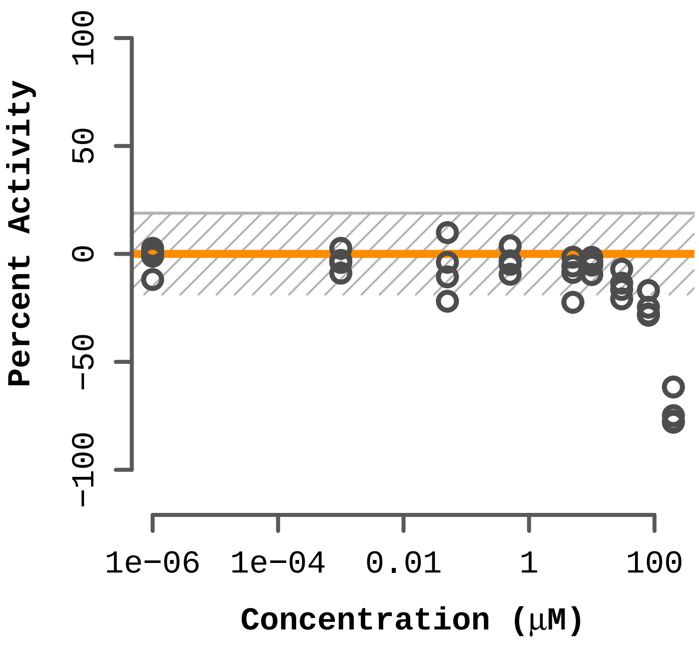

ASSAY: AEID3097 (CCTE_Deisenroth_DEVTOX_RUES2-GLR_Cel

NAME: MEHP
CHID: 25680 CASRN: 4376-20-9
SPID(S): ES211_44_44
M4ID: 42676173

HILL MODEL: Failed to converge.

GAIN-LOSS MODEL: Failed to converge.

	CNST	HILL	GNLS
AIC:	329.82	NA	NA
PROB:	1	NA	NA
RMSE:	27.25	NA	NA

MAX_MEAN: -2.45 MAX_MED: -0.279 BMAD: 6.29

COFF: 18.9 HIT-CALL: 0 FITC: 7 ACTP: 0

FLAGS: 10

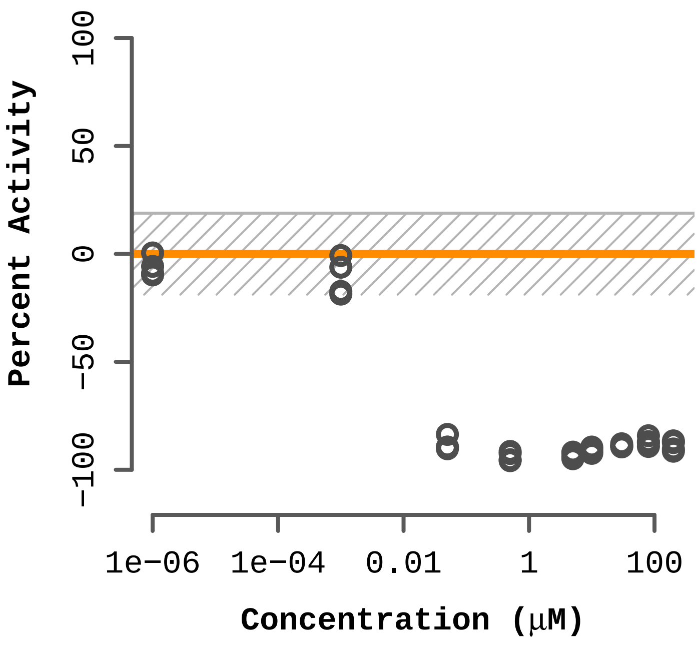

ASSAY: AEID3097 (CCTE_Deisenroth_DEVTOX_RUES2-GLR_Cel

NAME: Adriamycin hydrochloride
CHID: 30636 CASRN: 25316-40-9
SPID(S): ES211_67_67
M4ID: 42676163

HILL MODEL: Failed to converge.

GAIN-LOSS MODEL: Failed to converge.

	CNST	HILL	GNLS
AIC:	490.14	NA	NA
PROB:	1	NA	NA
RMSE:	79.67	NA	NA

MAX_MEAN: -5.98 MAX_MED: -7.33 BMAD: 6.29

COFF: 18.9 HIT-CALL: 0 FITC: 7 ACTP: 0

FLAGS: 10

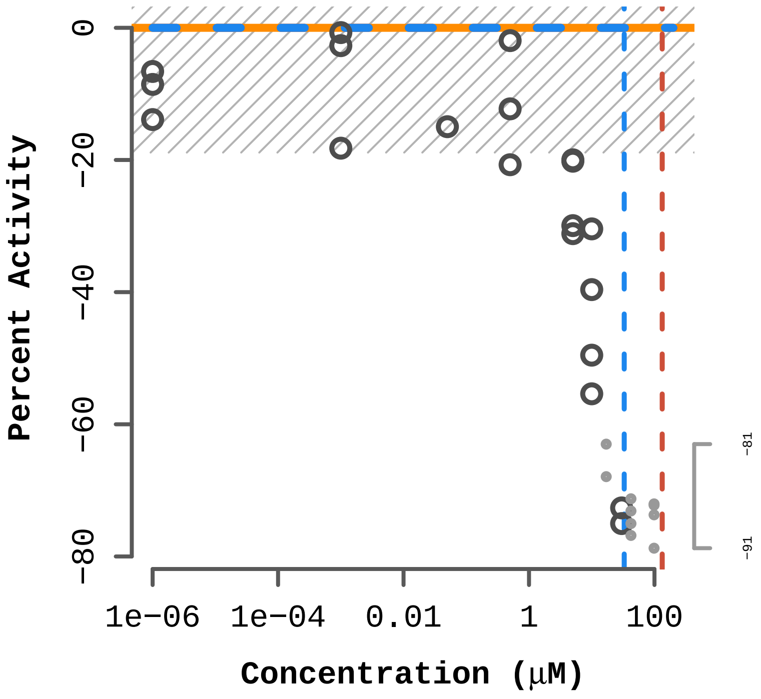

ASSAY: AEID3097 (CCTE_Deisenroth_DEVTOX_RUES2-GLR_Cel

NAME: 5-Bromo-2'-deoxyuridine
CHID: 33105 CASRN: 59-14-3
SPID(S): ES211_4_4
M4ID: 42676149 BRK

HILL MODEL (in red):

	tp	ga	gw
val:	3.51e-09	2.12	2.52
sd:	NaN	NaN	NaN

GAIN-LOSS MODEL (in blue):

	tp	ga	gw	la	lw
val:	1.61e-07	1.52	4.96	1.79	17.1
sd:	0.00174	637	68400	2950	441000

	CNST	HILL	GNLS
AIC:	391.52	397.52	401.52
PROB:	0.95	0.05	0.01
RMSE:	52.26	52.26	52.26

MAX_MEAN: 1.28 MAX_MED: 2.62 BMAD: 6.29

COFF: 18.9 HIT-CALL: 0 FITC: 7 ACTP: 0.05

FLAGS: 10

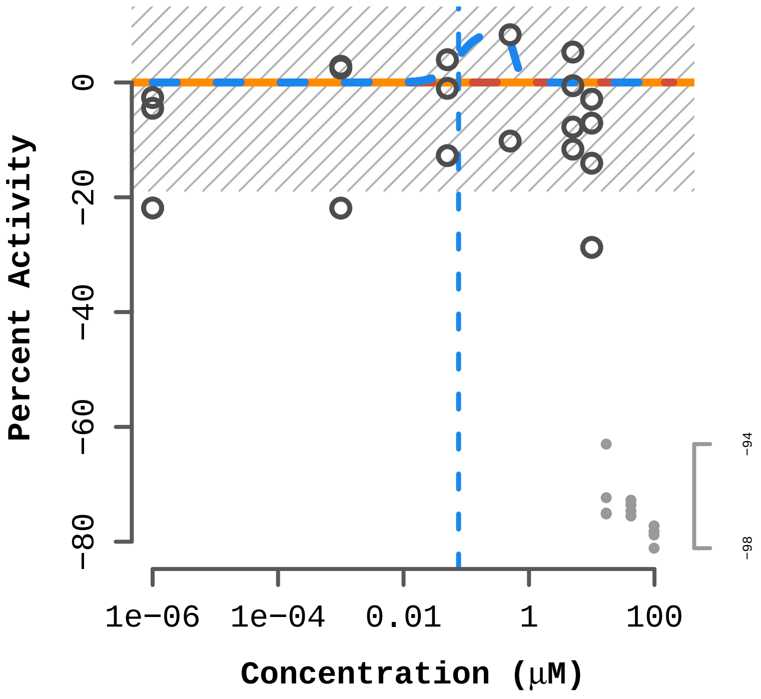

ASSAY: AEID3097 (CCTE_Deisenroth_DEVTOX_RUES2-GLR_Cel

NAME: Amiodarone hydrochloride
CHID: 37185 CASRN: 19774-82-4
SPID(S): ES211_14_14
M4ID: 42676131 BRK

HILL MODEL (in red):

	tp	ga	gw
val:	0.155	2.8	7.85
sd:	NaN	NaN	NaN

GAIN-LOSS MODEL (in blue):

	tp	ga	gw	la	lw
val:	9.07	-1.12	2.5	-0.228	7.46
sd:	NaN	NaN	NaN	NaN	NaN

	CNST	HILL	GNLS
AIC:	394.9	400.9	404.76
PROB:	0.95	0.05	0.01
RMSE:	56.63	56.63	56.58

MAX_MEAN: 6.91 MAX_MED: 9.03 BMAD: 6.29

COFF: 18.9 HIT-CALL: 0 FITC: 7 ACTP: 0.05

FLAGS: 10

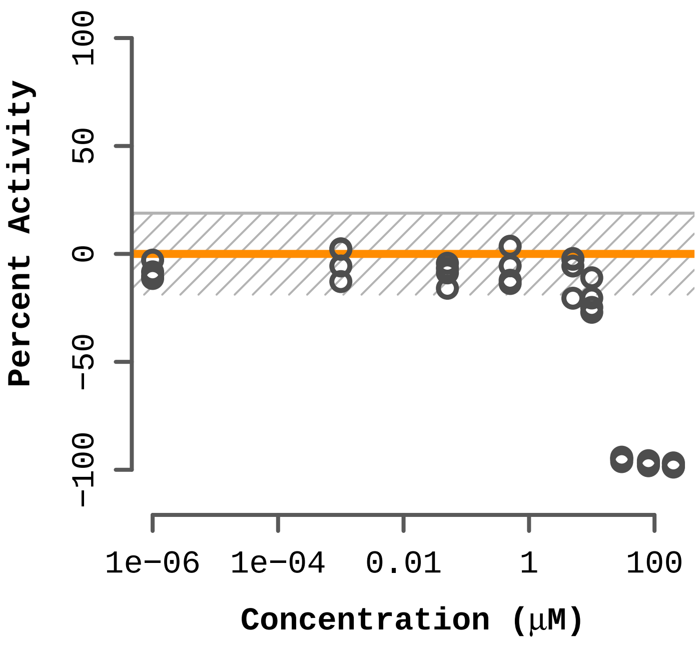

ASSAY: AEID3097 (CCTE_Deisenroth_DEVTOX_RUES2-GLR_Cel

NAME: Imatinib mesylate
CHID: 40502 CASRN: 220127-57-1
SPID(S): ES211_37_37
M4ID: 42676108

HILL MODEL: Failed to converge.

GAIN-LOSS MODEL: Failed to converge.

	CNST	HILL	GNLS
AIC:	394.76	NA	NA
PROB:	1	NA	NA
RMSE:	56.66	NA	NA

MAX_MEAN: -3.45 MAX_MED: -1.7 BMAD: 6.29

COFF: 18.9 HIT-CALL: 0 FITC: 7 ACTP: 0

FLAGS: 10

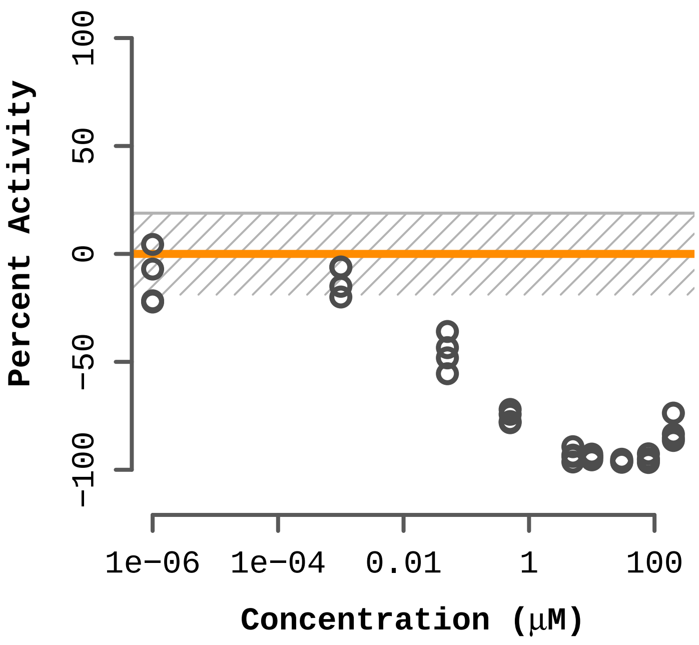

ASSAY: AEID3097 (CCTE_Deisenroth_DEVTOX_RUES2-GLR_Cel

NAME: Dasatinib
CHID: 40979 CASRN: 302962-49-8
SPID(S): ES211_26_26
M4ID: 42676118

HILL MODEL: Failed to converge.

GAIN-LOSS MODEL: Failed to converge.

	CNST	HILL	GNLS
AIC:	422.07	NA	NA
PROB:	1	NA	NA
RMSE:	75.12	NA	NA

MAX_MEAN: -11.6 MAX_MED: -10.6 BMAD: 6.29

COFF: 18.9 HIT-CALL: 0 FITC: 7 ACTP: 0

FLAGS: 10

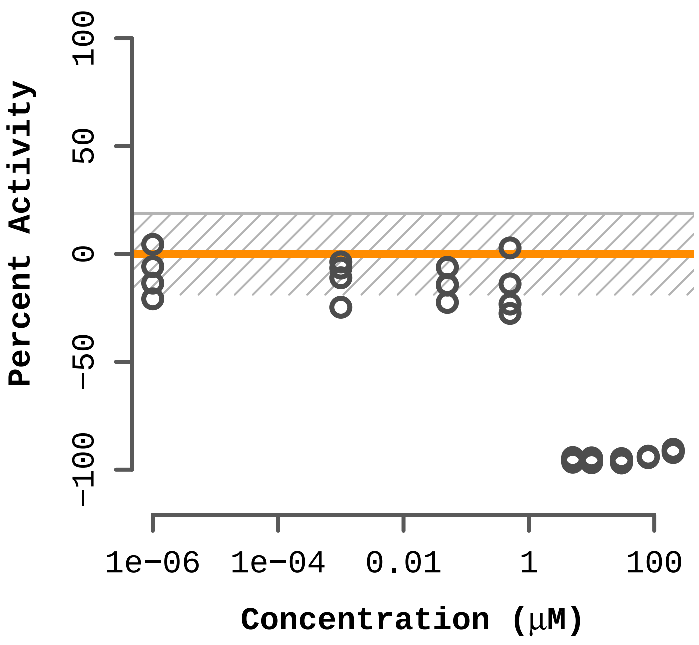

ASSAY: AEID3097 (CCTE_Deisenroth_DEVTOX_RUES2-GLR_Cel

NAME: Sorafenib
CHID: 41128 CASRN: 284461-73-0
SPID(S): ES211_57_57
M4ID: 42676127

HILL MODEL: Failed to converge.

GAIN-LOSS MODEL: Failed to converge.

	CNST	HILL	GNLS
AIC:	416.98	NA	NA
PROB:	1	NA	NA
RMSE:	71.16	NA	NA

MAX_MEAN: -8.91 MAX_MED: -8.77 BMAD: 6.29

COFF: 18.9 HIT-CALL: 0 FITC: 7 ACTP: 0

FLAGS: 10

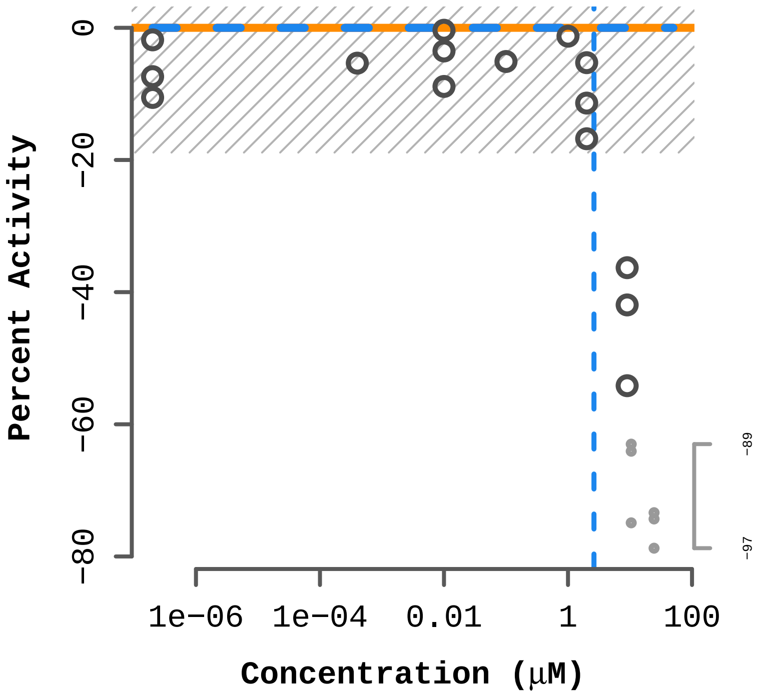

ASSAY: AEID3097 (CCTE_Deisenroth_DEVTOX_RUES2-GLR_Cel

NAME: Ritanserin
CHID: 42594 CASRN: 87051-43-2
SPID(S): ES211_53_53
M4ID: 42676124 BRK

HILL MODEL (in red):

	tp	ga	gw
val:	6.76e-06	2.17	4.8
sd:	NaN	NaN	NaN

GAIN-LOSS MODEL (in blue):

	tp	ga	gw	la	lw
val:	2.14e-08	0.419	6.49	0.845	10.5
sd:	NaN	NaN	NaN	NaN	NaN

	CNST	HILL	GNLS
AIC:	319.9	325.9	329.9
PROB:	0.95	0.05	0.01
RMSE:	44.13	44.13	44.13

MAX_MEAN: 9.3 MAX_MED: 9.13 BMAD: 6.29

COFF: 18.9 HIT-CALL: 0 FITC: 7 ACTP: 0.05

FLAGS: 10

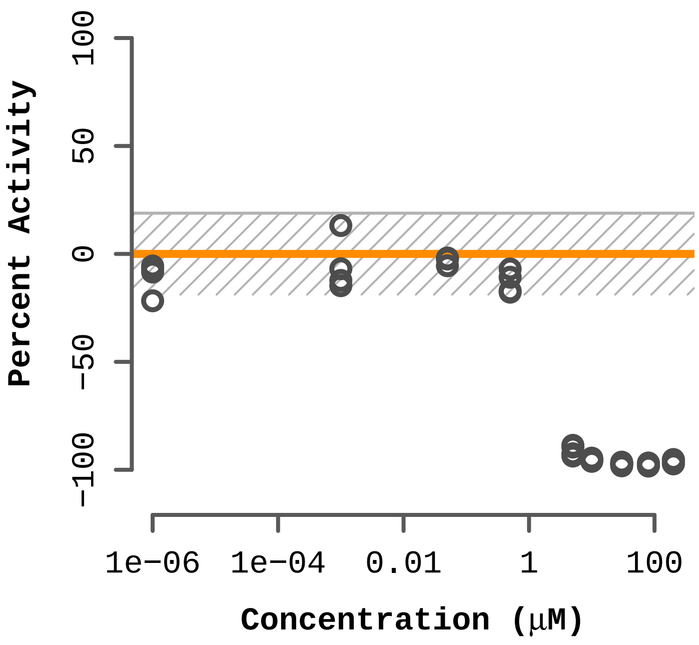

ASSAY: AEID3097 (CCTE_Deisenroth_DEVTOX_RUES2-GLR_Cel

NAME: Sunitinib malate
CHID: 46492 CASRN: 341031−54−7
SPID(S): ES211_60_60
M4ID: 42676159

HILL MODEL: Failed to converge.

GAIN−LOSS MODEL: Failed to converge.

	CNST	HILL	GNLS
AIC:	416.93	NA	NA
PROB:	1	NA	NA
RMSE:	71.6	NA	NA

MAX_MEAN: −2.92 MAX_MED: −2.16 BMAD: 6.29

COFF: 18.9 HIT−CALL: 0 FITC: 7 ACTP: 0

FLAGS: 10

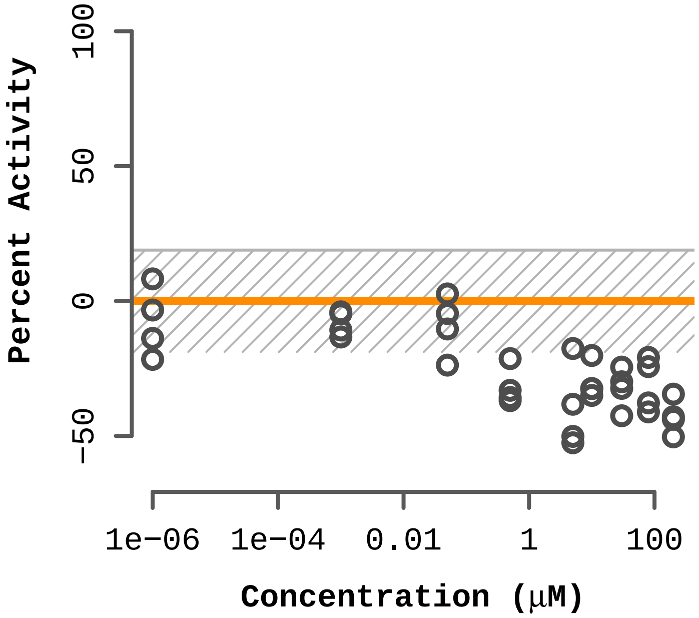

ASSAY: AEID3097 (CCTE_Deisenroth_DEVTOX_RUES2-GLR_Cel

NAME: Lenalidomide
CHID: 46664 CASRN: 191732-72-6
SPID(S): ES211_41_41
M4ID: 42676154

HILL MODEL: Failed to converge.

GAIN-LOSS MODEL: Failed to converge.

	CNST	HILL	GNLS
AIC:	355.18	NA	NA
PROB:	1	NA	NA
RMSE:	30.14	NA	NA

MAX_MEAN: -7.64 MAX_MED: -7.48 BMAD: 6.29

COFF: 18.9 HIT-CALL: 0 FITC: 7 ACTP: 0

FLAGS: 10

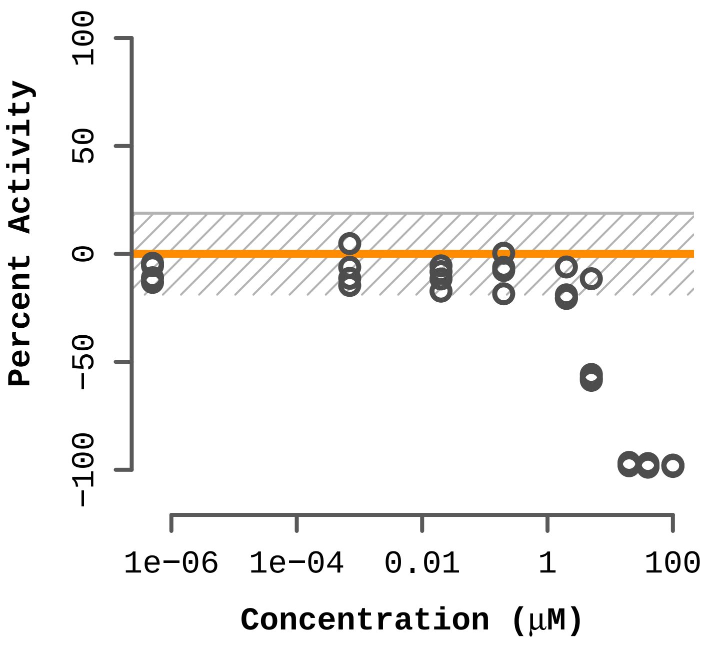

ASSAY: AEID3097 (CCTE_Deisenroth_DEVTOX_RUES2-GLR_Cel

NAME: Vandetanib
CHID: 46681 CASRN: 443913-73-3
SPID(S): ES211_64_64
M4ID: 42676112

HILL MODEL: Failed to converge.

GAIN-LOSS MODEL: Failed to converge.

	CNST	HILL	GNLS
AIC:	400.29	NA	NA
PROB:	1	NA	NA
RMSE:	59.58	NA	NA

MAX_MEAN: -6.81 MAX_MED: -6.9 BMAD: 6.29

COFF: 18.9 HIT-CALL: 0 FITC: 7 ACTP: 0

FLAGS: 10

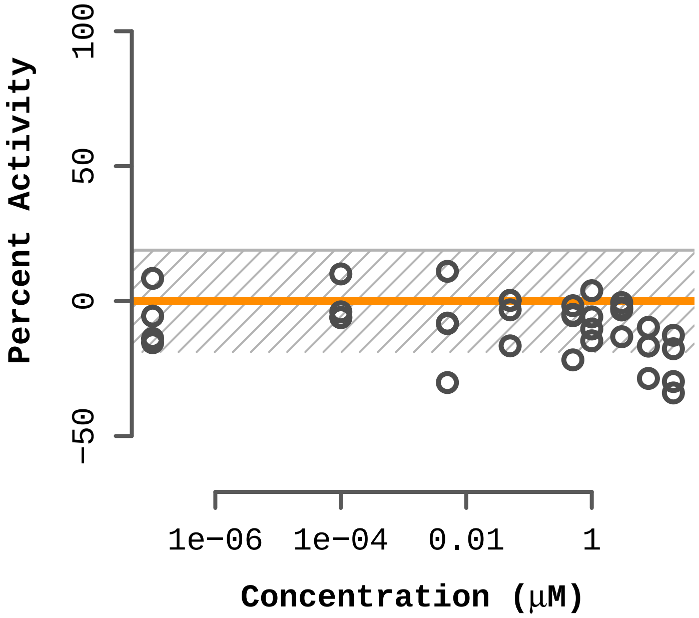

ASSAY: AEID3097 (CCTE_Deisenroth_DEVTOX_RUES2-GLR_Cel

NAME: Dexamethasone sodium phosphate
CHID: 47429 CASRN: 2392−39−4
SPID(S): ES211_28_28
M4ID: 42676121

HILL MODEL: Failed to converge.

GAIN−LOSS MODEL: Failed to converge.

	CNST	HILL	GNLS
AIC:	287.46	NA	NA
PROB:	1	NA	NA
RMSE:	14.13	NA	NA

MAX_MEAN: −1.52 MAX_MED: −1.5 BMAD: 6.29

COFF: 18.9 HIT−CALL: 0 FITC: 7 ACTP: 0

FLAGS:

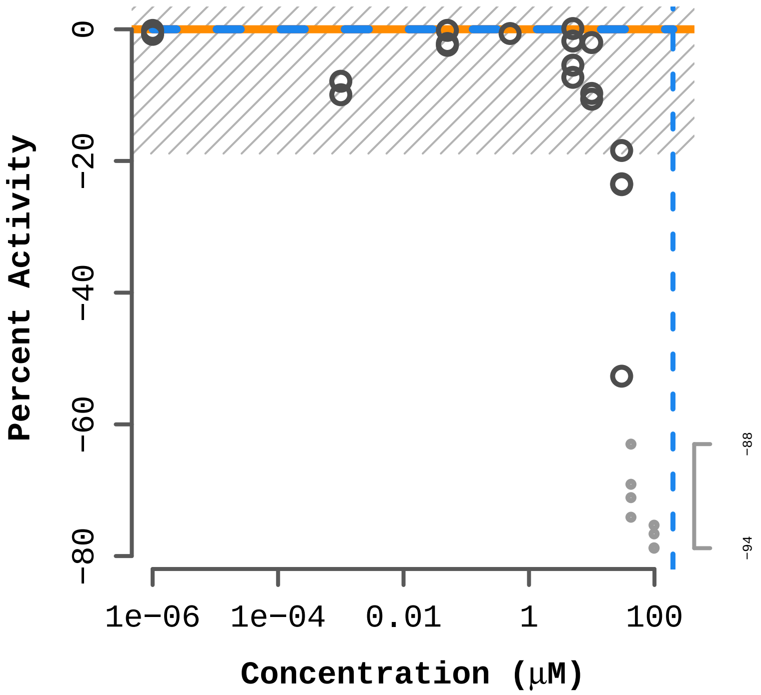

ASSAY: AEID3097 (CCTE_Deisenroth_DEVTOX_RUES2-GLR_Cel

NAME: Esomeprazole magnesium hydrate
CHID: 746660 CASRN: 668985-31-7
SPID(S): ES211_43_43
M4ID: 42676165 BRK

HILL MODEL (in red):

	tp	ga	gw
val:	0.213	2.8	7.93
sd:	NaN	NaN	NaN

GAIN-LOSS MODEL (in blue):

	tp	ga	gw	la	lw
val:	7.89e-09	2.3	7.68	4.3	9.01
sd:	NA	NA	NA	NA	NA

	CNST	HILL	GNLS
AIC:	373.34	379.34	383.34
PROB:	0.95	0.05	0.01
RMSE:	45.47	45.47	45.47

MAX_MEAN: 7.03 MAX_MED: 6.51 BMAD: 6.29

COFF: 18.9 HIT-CALL: 0 FITC: 7 ACTP: 0.05

FLAGS: 10

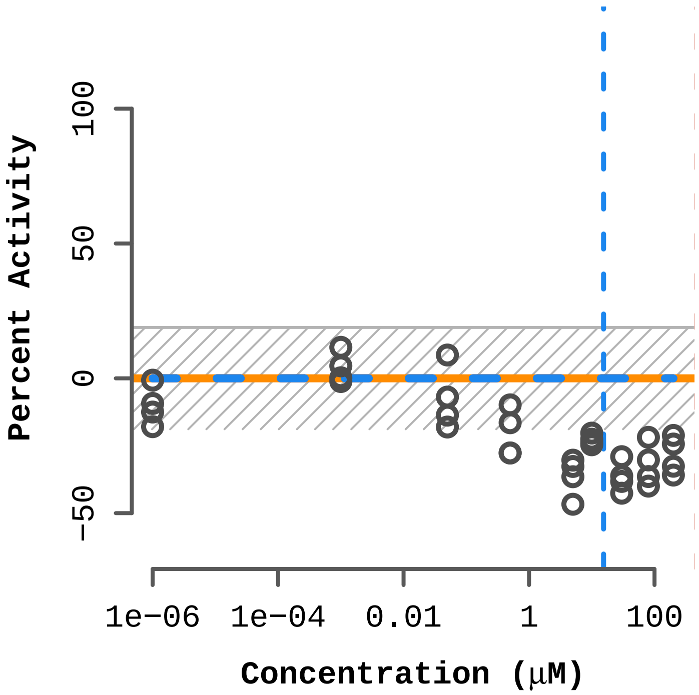

ASSAY: AEID3097 (CCTE_Deisenroth_DEVTOX_RUES2-GLR_Cel

NAME: Pomalidomide
CHID: 893458 CASRN: 19171-19-8
SPID(S): ES211_50_50
M4ID: 42676155

HILL MODEL (in red):

	tp	ga	gw
val:	9.84e-09	2.67	2.57
sd:	NaN	NaN	NaN

GAIN-LOSS MODEL (in blue):

	tp	ga	gw	la	lw
val:	1.86e-08	1.19	3.76	1.49	2.63
sd:	NaN	NaN	NaN	NaN	NaN

	CNST	HILL	GNLS
AIC:	343.99	349.99	353.99
PROB:	0.95	0.05	0.01
RMSE:	25.81	25.81	25.81

MAX_MEAN: 3.8 MAX_MED: 2.43 BMAD: 6.29

COFF: 18.9 HIT-CALL: 0 FITC: 7 ACTP: 0.05

FLAGS: 10

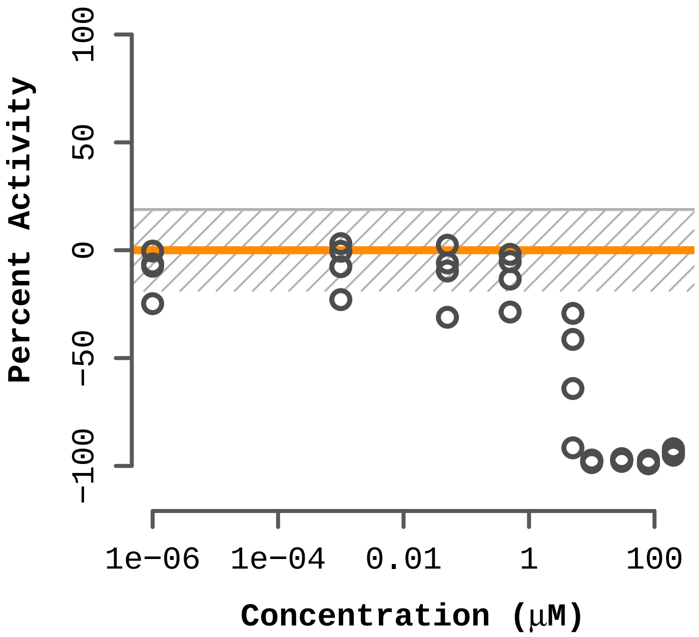

ASSAY: AEID3097 (CCTE_Deisenroth_DEVTOX_RUES2-GLR_Cel

NAME: Tegaserod maleate
CHID: 904761 CASRN: 189188-57-6
SPID(S): ES211_61_61
M4ID: 42676109

HILL MODEL: Failed to converge.

GAIN-LOSS MODEL: Failed to converge.

	CNST	HILL	GNLS
AIC:	413.04	NA	NA
PROB:	1	NA	NA
RMSE:	68.38	NA	NA

MAX_MEAN: -6.97 MAX_MED: -4.05 BMAD: 6.29

COFF: 18.9 HIT-CALL: 0 FITC: 7 ACTP: 0

FLAGS: 10

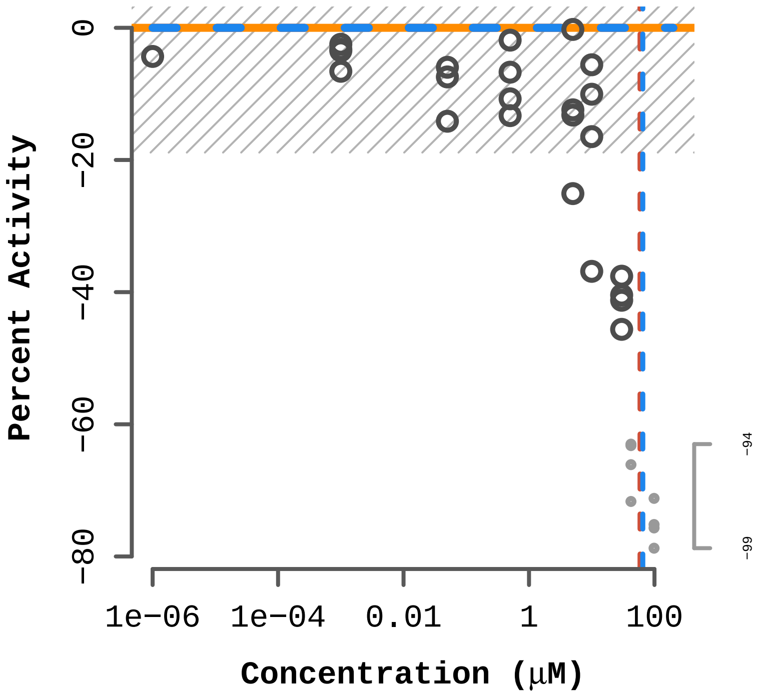

ASSAY: AEID3097 (CCTE_Deisenroth_DEVTOX_RUES2-GLR_Cel

NAME: Mianserin hydrochloride
CHID: 944145 CASRN: 21535-47-7
SPID(S): ES211_47_47
M4ID: 42676117 BRK

HILL MODEL (in red):

	tp	ga	gw
val:	2.23e-08	1.77	1.49
sd:	0.000915	1540	167000

GAIN-LOSS MODEL (in blue):

	tp	ga	gw	la	lw
val:	1.38e-08	1.81	1.23	2.56	5.17
sd:	NaN	NaN	NaN	NaN	NaN

	CNST	HILL	GNLS
AIC:	380.54	386.54	390.54
PROB:	0.95	0.05	0.01
RMSE:	48.52	48.52	48.52

MAX_MEAN: 3.09 MAX_MED: 0.606 BMAD: 6.29

COFF: 18.9 HIT-CALL: 0 FITC: 7 ACTP: 0.05

FLAGS: 10

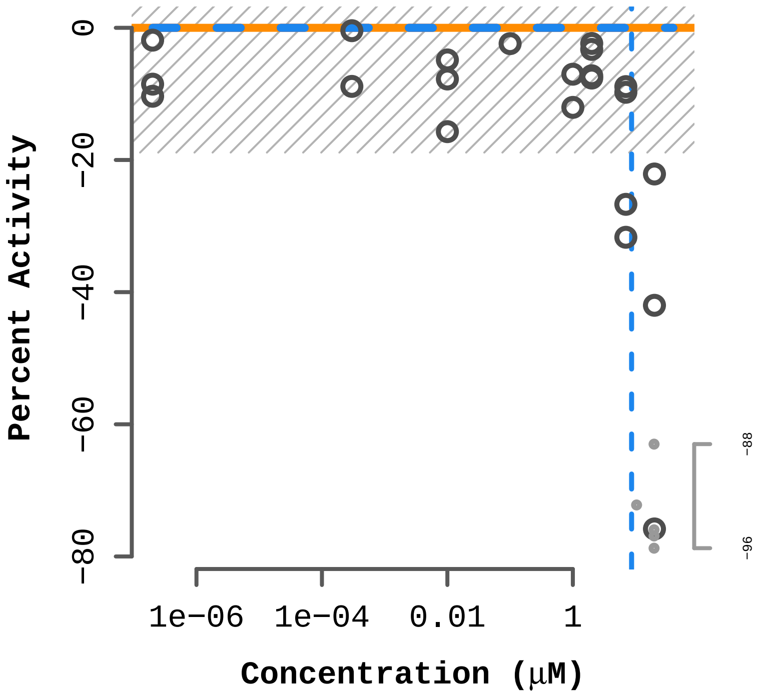

ASSAY: AEID3097 (CCTE_Deisenroth_DEVTOX_RUES2-GLR_Cel

NAME: Dorsomorphin
CHID: 1006988 CASRN: 866405-64-3
SPID(S): ES211_6_6
M4ID: 42676164 BRK

HILL MODEL (in red):

	tp	ga	gw
val:	0.00122	2.1	7.18
sd:	NaN	NaN	NaN

GAIN-LOSS MODEL (in blue):

	tp	ga	gw	la	lw
val:	2.85e-08	0.936	8	1.21	9.59
sd:	NaN	NaN	NaN	NaN	NaN

	CNST	HILL	GNLS
AIC:	356.44	362.44	366.44
PROB:	0.95	0.05	0.01
RMSE:	39.1	39.1	39.1

MAX_MEAN: 5.29 MAX_MED: 4.29 BMAD: 6.29

COFF: 18.9 HIT-CALL: 0 FITC: 7 ACTP: 0.05

FLAGS: 10

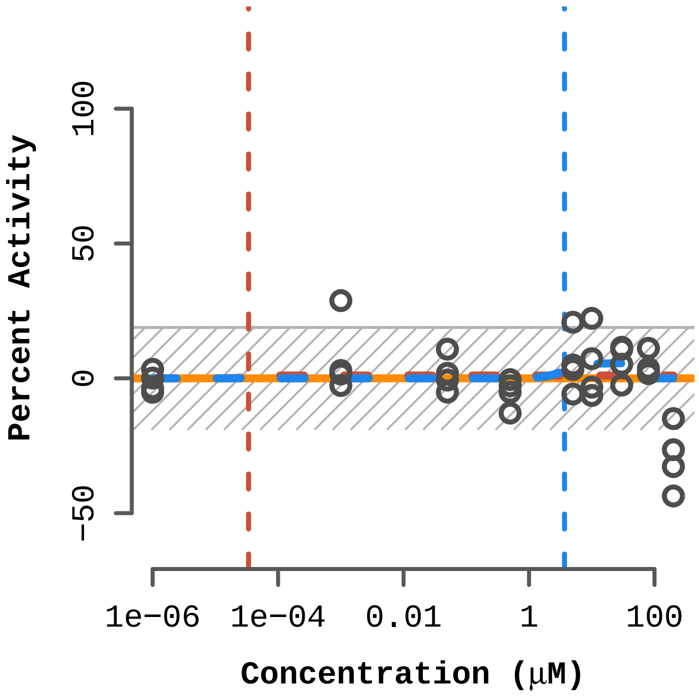

ASSAY: AEID3097 (CCTE_Deisenroth_DEVTOX_RUES2-GLR_Cel

NAME: (2R,3S)-2-(3,4-Dihydroxyphenyl)-3,4-dihydro-2H-
CHID: 1017388 CASRN: 225937-10-0
SPID(S): ES211_22_22
M4ID: 42676135

HILL MODEL (in red):

	tp	ga	gw
val:	1.04	-4.47	4.26
sd:	1.83	366	1050

GAIN-LOSS MODEL (in blue):

	tp	ga	gw	la	lw
val:	5.61	0.566	2.75	1.94	17.3
sd:	NaN	NaN	NaN	NaN	NaN

	CNST	HILL	GNLS
AIC:	285.31	290.99	290.97
PROB:	0.9	0.05	0.05
RMSE:	13.62	13.69	13.14

MAX_MEAN: 7.68 MAX_MED: 8.02 BMAD: 6.29

COFF: 18.9 HIT-CALL: 0 FITC: 7 ACTP: 0.1

FLAGS:

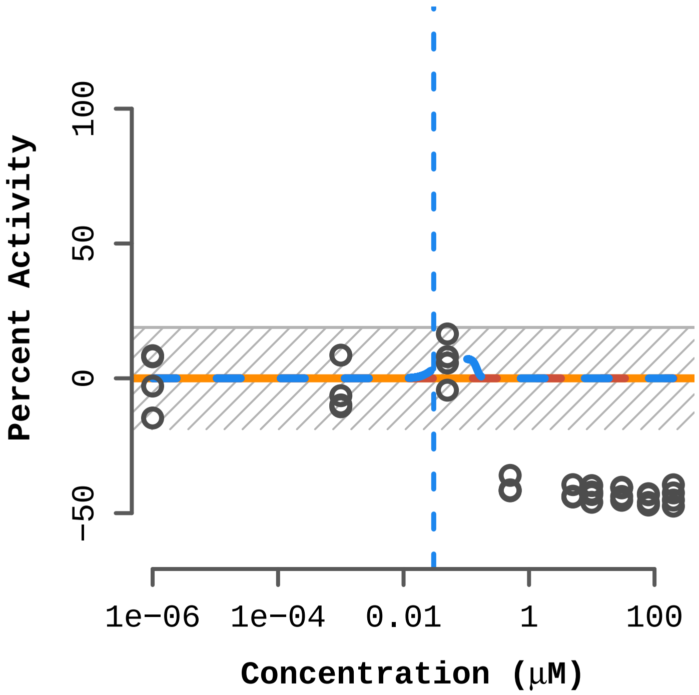

ASSAY: AEID3097 (CCTE_Deisenroth_DEVTOX_RUES2-GLR_Cel

NAME: Methotrexate hydrate(1:x)
CHID: 1017640 CASRN: 133073-73-1
SPID(S): ES211_45_45
M4ID: 42676144

HILL MODEL (in red):

	tp	ga	gw
val:	1.66e-05	2.8	4.55
sd:	NaN	NaN	NaN

GAIN-LOSS MODEL (in blue):

	tp	ga	gw	la	lw
val:	7.23	-1.52	4.27	-0.833	14
sd:	1030	125	1020	487	12800

	CNST	HILL	GNLS
AIC:	367.68	373.68	377.5
PROB:	0.95	0.05	0.01
RMSE:	35.4	35.4	35.34

MAX_MEAN: 6.46 MAX_MED: 6.87 BMAD: 6.29

COFF: 18.9 HIT-CALL: 0 FITC: 7 ACTP: 0.05

FLAGS: 10

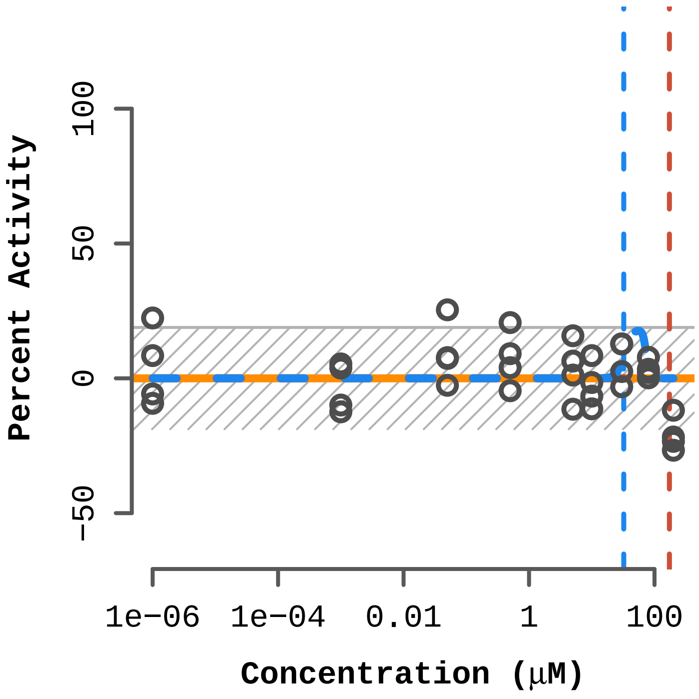

ASSAY: AEID3097 (CCTE_Deisenroth_DEVTOX_RUES2-GLR_Cel

NAME: 5,5-Diphenylhydantoin
CHID: 20541 CASRN: 57-41-0
SPID(S): ES211_3_3
M4ID: 42676111

HILL MODEL (in red):

	tp	ga	gw
val:	4.99e-08	2.24	0.377
sd:	NaN	NaN	NaN

GAIN-LOSS MODEL (in blue):

	tp	ga	gw	la	lw
val:	18	1.51	7.79	1.87	17.4
sd:	NaN	NaN	NaN	NaN	NaN

	CNST	HILL	GNLS
AIC:	285.61	291.61	293.59
PROB:	0.94	0.05	0.02
RMSE:	12.09	12.09	11.85

MAX_MEAN: 9.49 MAX_MED: 7.63 BMAD: 6.29

COFF: 18.9 HIT-CALL: 0 FITC: 9 ACTP: 0.06

FLAGS:

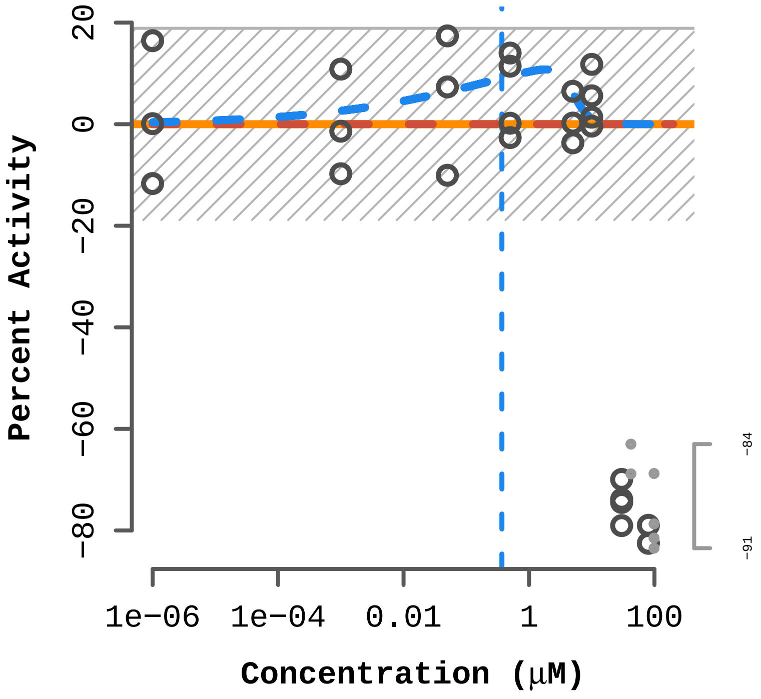

ASSAY: AEID3097 (CCTE_Deisenroth_DEVTOX_RUES2-GLR_Cel

NAME: 6-aminopyridine-3-carboxamide
CHID: 51446 CASRN: 329-89-5
SPID(S): ES211_7_7
M4ID: 42676172 BRK

HILL MODEL (in red):

	tp	ga	gw
val:	4.69e-06	2.75	4.18
sd:	NaN	NaN	NaN

GAIN-LOSS MODEL (in blue):

	tp	ga	gw	la	lw
val:	18.1	-0.432	0.3	0.694	3.33
sd:	NaN	NaN	NaN	NaN	NaN

	CNST	HILL	GNLS
AIC:	384.81	390.81	394.22
PROB:	0.94	0.05	0.01
RMSE:	48.93	48.93	48.73

MAX_MEAN: 9.62 MAX_MED: 12.4 BMAD: 6.29

COFF: 18.9 HIT-CALL: 0 FITC: 9 ACTP: 0.06

FLAGS: 10

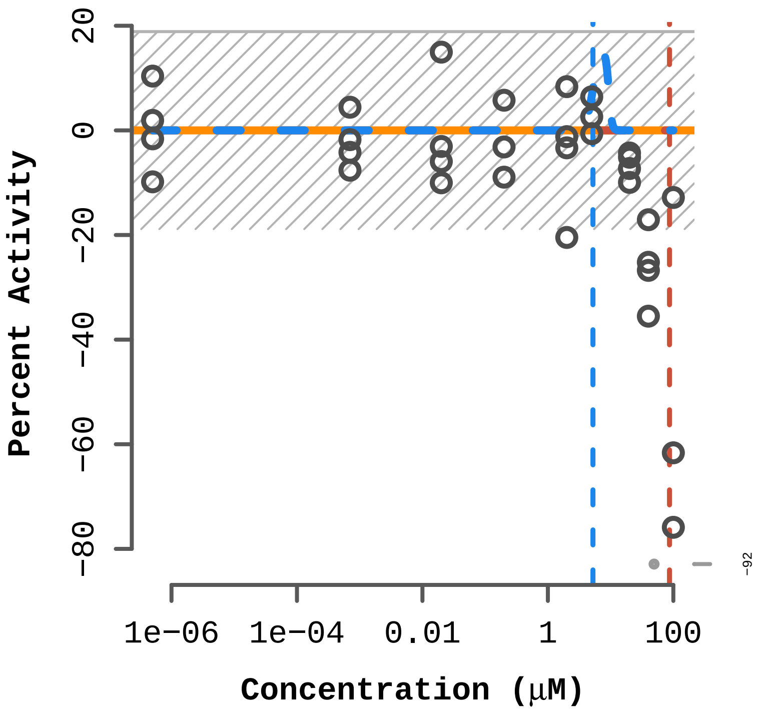

ASSAY: AEID3097 (CCTE_Deisenroth_DEVTOX_RUES2-GLR_Cel

NAME: Ketanserin tartrate
CHID: 1003945 CASRN: 83846-83-7
SPID(S): ES211_40_40
M4ID: 42676133 BRK

HILL MODEL (in red):

	tp	ga	gw
val:	9.22e-09	1.94	1.64
sd:	4.84e-05	4050	37600

GAIN-LOSS MODEL (in blue):

	tp	ga	gw	la	lw
val:	15.8	0.718	7.85	0.969	17.8
sd:	396	2.42	49.8	33.1	1770

	CNST	HILL	GNLS
AIC:	320.52	326.52	329.53
PROB:	0.94	0.05	0.01
RMSE:	25.47	25.47	25.36

MAX_MEAN: 7.16 MAX_MED: 4.52 BMAD: 6.29

COFF: 18.9 HIT-CALL: 0 FITC: 9 ACTP: 0.06

FLAGS: 10

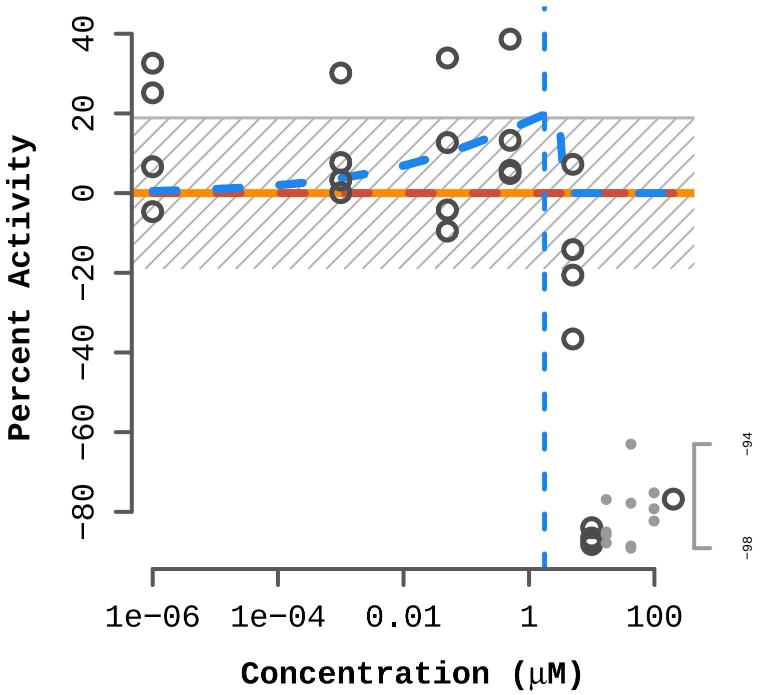

ASSAY: AEID3097 (CCTE_Deisenroth_DEVTOX_RUES2-GLR_Cel

NAME: Gefitinib
CHID: 41034 CASRN: 184475-35-2
SPID(S): ES211_34_34
M4ID: 42676129 BRK

HILL MODEL (in red):

	tp	ga	gw
val:	0.929	2.8	7.94
sd:	NaN	NaN	NaN

GAIN-LOSS MODEL (in blue):

	tp	ga	gw	la	lw
val:	39.5	0.247	0.3	0.519	17.9
sd:	NaN	NaN	NaN	NaN	NaN

	CNST	HILL	GNLS
AIC:	407.33	413.33	416.74
PROB:	0.94	0.05	0.01
RMSE:	63.76	63.76	63.42

MAX_MEAN: 15.6 MAX_MED: 15.9 BMAD: 6.29

COFF: 18.9 HIT-CALL: 0 FITC: 10 ACTP: 0.06

FLAGS: 10

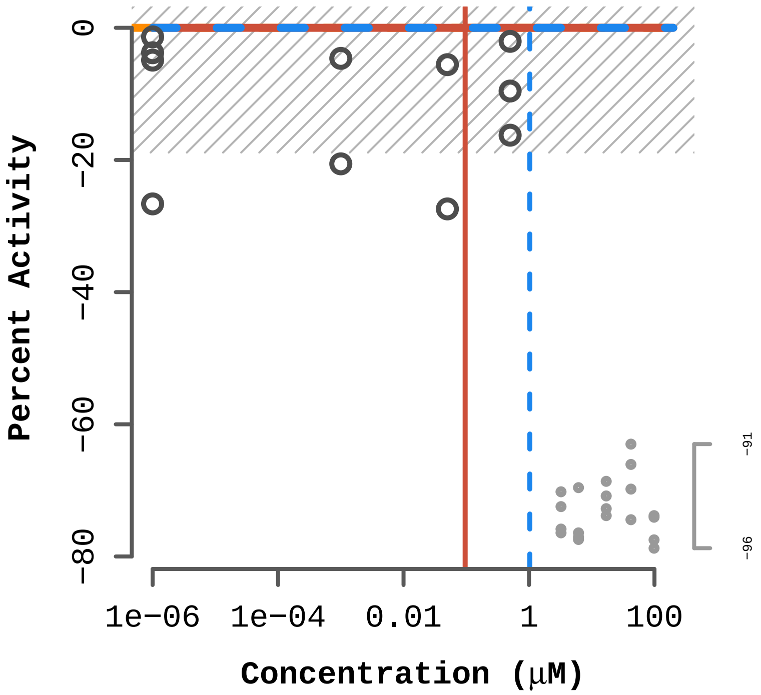

ASSAY: AEID3097 (CCTE_Deisenroth_DEVTOX_RUES2-GLR_Cel

NAME: Diethylstilbestrol
CHID: 20465 CASRN: 56−53−1
SPID(S): ES211_29_29
M4ID: 42676152 BRK

HILL MODEL (in red):

	tp	ga	gw
val:	7.73e−10	−1.02	0.507
sd:	NaN	NaN	NaN

GAIN−LOSS MODEL (in blue):

	tp	ga	gw	la	lw
val:	4.16e−08	0.0123	1.29	1.1	4.03
sd:	0.000695	110000	22500	5690	965000

	CNST	HILL	GNLS
AIC:	436.24	422.54	426.54
PROB:	0	0.88	0.12
RMSE:	71.09	71.09	71.09

MAX_MEAN: −1.55 MAX_MED: 0.78 BMAD: 6.29

COFF: 18.9 HIT−CALL: 0 FITC: 13 ACTP: 1

FLAGS: 10
