# Supplementary material for: The DevTox Germ Layer Reporter Platform: An Assay Adaptation of the Human Pluripotent Stem Cell Test
Source: Toxics. 2022 Jul 13;10(7):392. doi: 10.3390/toxics10070392 (PMC9321663; doi:10.3390/toxics10070392)
Supplement: Supplementary file 1 [file toxics-10-00392-s001.zip › AEID3098_L6_CCTE_Deisenroth_DEVTOX_RUES2-GLR_CellCount_dn_210917.pdf]

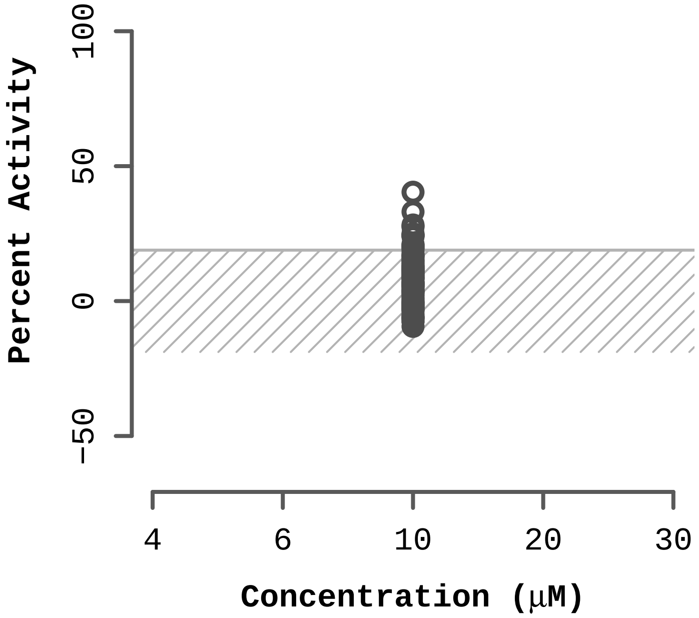

ASSAY: AEID3098 (CCTE_Deisenroth_DEVTOX_RUES2-GLR_Cel

NAME: SB-431542
CHID: 42693 CASRN: 301836-41-9
SPID(S): ES211_56_56
M4ID: 42676199

HILL MODEL: Not applicable.

GAIN-LOSS MODEL: Not applicable.

	CNST	HILL	GNLS
AIC:	NA	NA	NA
PROB:	NA	NA	NA
RMSE:	NA	NA	NA

MAX_MEAN: 8.7 MAX_MED: 8.21 BMAD: 6.29

COFF: 18.9 HIT-CALL: -1 FITC: 2 ACTP: NA

FLAGS:

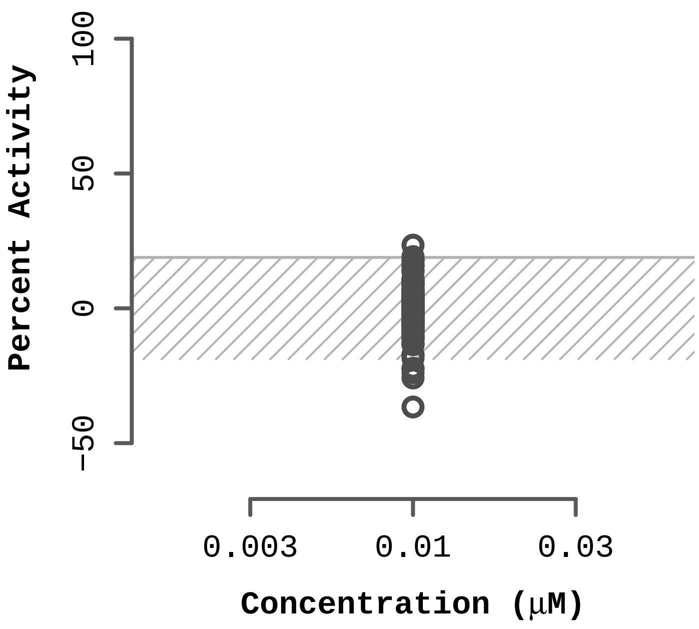

ASSAY: AEID3098 (CCTE_Deisenroth_DEVTOX_RUES2-GLR_Cel

NAME: DMSO (spid not in DB)
CHID: NA CASRN: NA
SPID(S): DMSO
M4ID: 42676201

HILL MODEL: Not applicable.

GAIN-LOSS MODEL: Not applicable.

	CNST	HILL	GNLS
AIC:	NA	NA	NA
PROB:	NA	NA	NA
RMSE:	NA	NA	NA

MAX_MEAN: -0.368 MAX_MED: 0 BMAD: 6.29

COFF: 18.9 HIT-CALL: -1 FITC: 2 ACTP: NA

FLAGS:

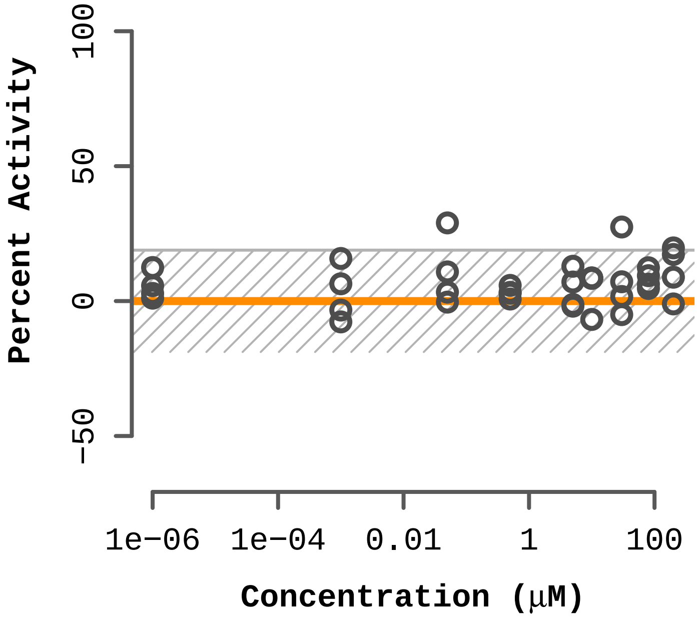

ASSAY: AEID3098 (CCTE_Deisenroth_DEVTOX_RUES2-GLR_Cel

NAME: Acetaminophen
CHID: 20006 CASRN: 103−90−2
SPID(S): ES211_9_9
M4ID: 42676223

HILL MODEL: Not applicable.

GAIN−LOSS MODEL: Not applicable.

	CNST	HILL	GNLS
AIC:	272.43	NA	NA
PROB:	1	NA	NA
RMSE:	10.5	NA	NA

MAX_MEAN: 11.2 MAX_MED: 13.1 BMAD: 6.29

COFF: 18.9 HIT−CALL: 0 FITC: 4 ACTP: 0

FLAGS:

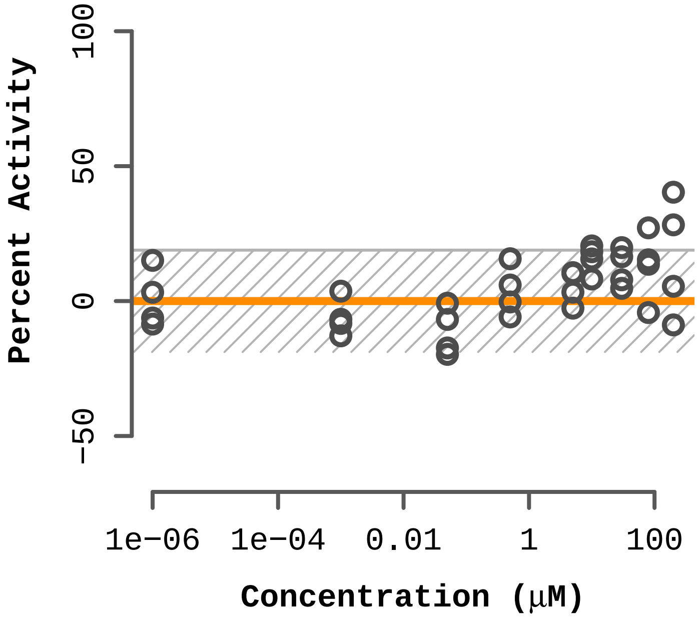

ASSAY: AEID3098 (CCTE_Deisenroth_DEVTOX_RUES2-GLR_Cel

NAME: Acrylamide
CHID: 20027 CASRN: 79-06-1
SPID(S): ES211_10_10
M4ID: 42676219

HILL MODEL: Not applicable.

GAIN-LOSS MODEL: Not applicable.

	CNST	HILL	GNLS
AIC:	297.76	NA	NA
PROB:	1	NA	NA
RMSE:	14.39	NA	NA

MAX_MEAN: 16.3 MAX_MED: 17 BMAD: 6.29

COFF: 18.9 HIT-CALL: 0 FITC: 4 ACTP: 0

FLAGS:

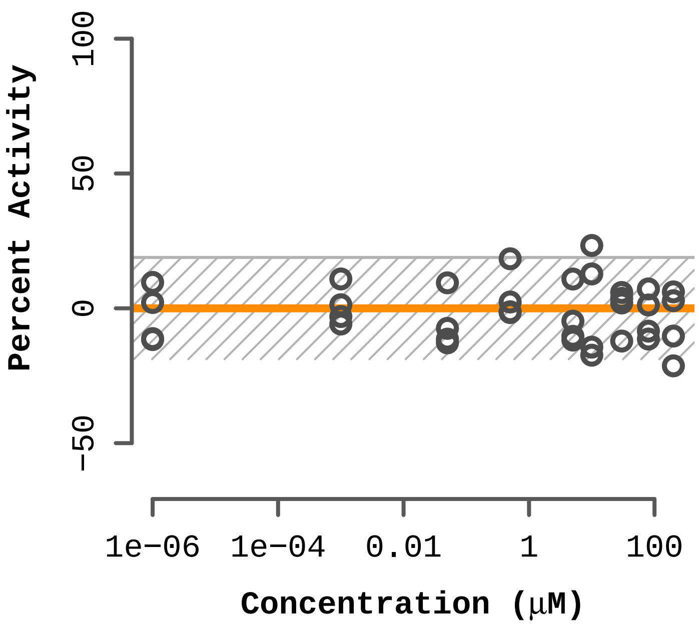

ASSAY: AEID3098 (CCTE_Deisenroth_DEVTOX_RUES2-GLR_Cel

NAME: Aspirin
CHID: 20108 CASRN: 50-78-2
SPID(S): ES211_15_15
M4ID: 42676239

HILL MODEL: Not applicable.

GAIN-LOSS MODEL: Not applicable.

	CNST	HILL	GNLS
AIC:	277.55	NA	NA
PROB:	1	NA	NA
RMSE:	10.5	NA	NA

MAX_MEAN: 4.48 MAX_MED: 2.85 BMAD: 6.29

COFF: 18.9 HIT-CALL: 0 FITC: 4 ACTP: 0

FLAGS:

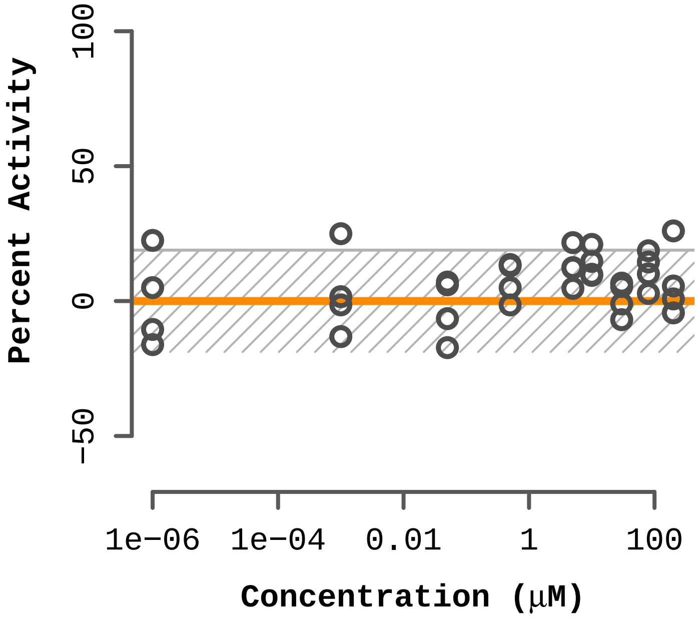

ASSAY: AEID3098 (CCTE_Deisenroth_DEVTOX_RUES2-GLR_Cel

NAME: Boric acid (H3BO3)
CHID: 20194 CASRN: 10043-35-3
SPID(S): ES211_17_17
M4ID: 42676191

HILL MODEL: Not applicable.

GAIN-LOSS MODEL: Not applicable.

	CNST	HILL	GNLS
AIC:	289.92	NA	NA
PROB:	1	NA	NA
RMSE:	12.55	NA	NA

MAX_MEAN: 13.8 MAX_MED: 12.3 BMAD: 6.29

COFF: 18.9 HIT-CALL: 0 FITC: 4 ACTP: 0

FLAGS:

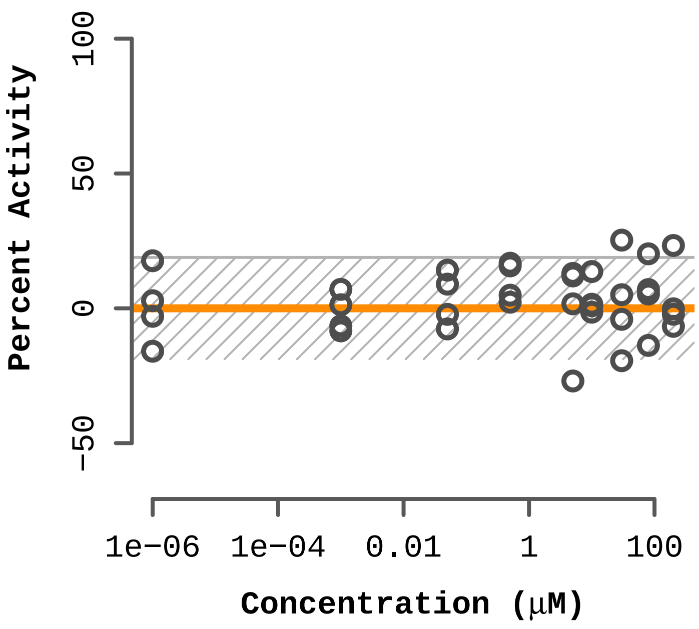

ASSAY: AEID3098 (CCTE_Deisenroth_DEVTOX_RUES2-GLR_Cel

NAME: Caffeine
CHID: 20232 CASRN: 58-08-2
SPID(S): ES211_20_20
M4ID: 42676214

HILL MODEL: Not applicable.

GAIN-LOSS MODEL: Not applicable.

	CNST	HILL	GNLS
AIC:	285.32	NA	NA
PROB:	1	NA	NA
RMSE:	12.03	NA	NA

MAX_MEAN: 9.91 MAX_MED: 10.3 BMAD: 6.29

COFF: 18.9 HIT-CALL: 0 FITC: 4 ACTP: 0

FLAGS:

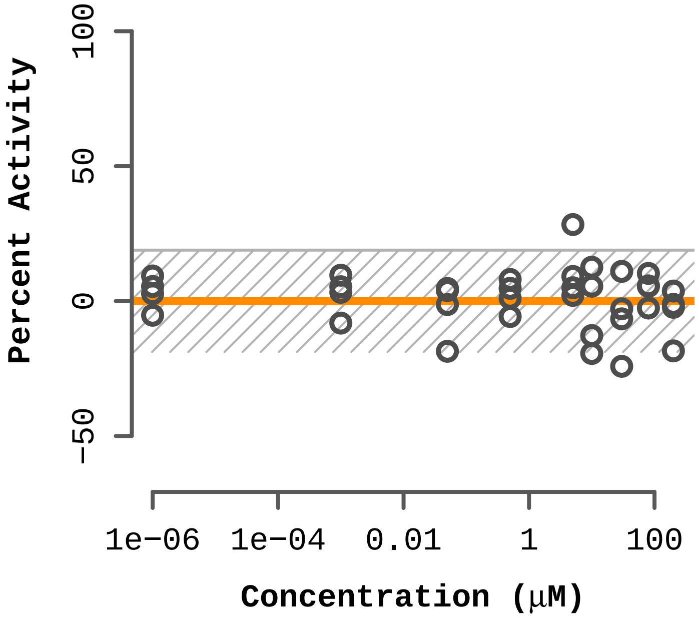

ASSAY: AEID3098 (CCTE_Deisenroth_DEVTOX_RUES2-GLR_Cel

NAME: Ethylene glycol
CHID: 20597 CASRN: 107-21-1
SPID(S): ES211_32_32
M4ID: 42676198

HILL MODEL: Not applicable.

GAIN-LOSS MODEL: Not applicable.

	CNST	HILL	GNLS
AIC:	271.02	NA	NA
PROB:	1	NA	NA
RMSE:	10.26	NA	NA

MAX_MEAN: 11.1 MAX_MED: 7.04 BMAD: 6.29

COFF: 18.9 HIT-CALL: 0 FITC: 4 ACTP: 0

FLAGS:

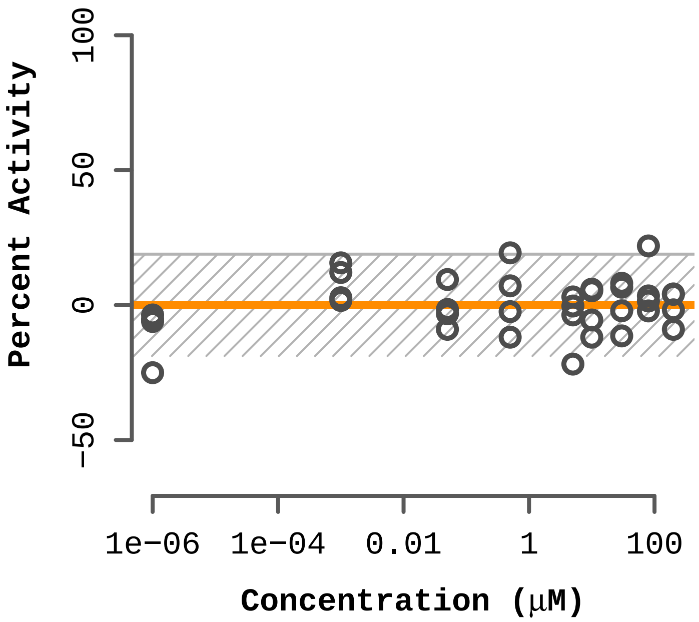

ASSAY: AEID3098 (CCTE_Deisenroth_DEVTOX_RUES2-GLR_Cel

NAME: Isoniazid
CHID: 20755 CASRN: 54−85−3
SPID(S): ES211_39_39
M4ID: 42676217

HILL MODEL: Not applicable.

GAIN−LOSS MODEL: Not applicable.

	CNST	HILL	GNLS
AIC:	267.93	NA	NA
PROB:	1	NA	NA
RMSE:	9.8	NA	NA

MAX_MEAN: 8.05 MAX_MED: 7.42 BMAD: 6.29

COFF: 18.9 HIT−CALL: 0 FITC: 4 ACTP: 0

FLAGS:

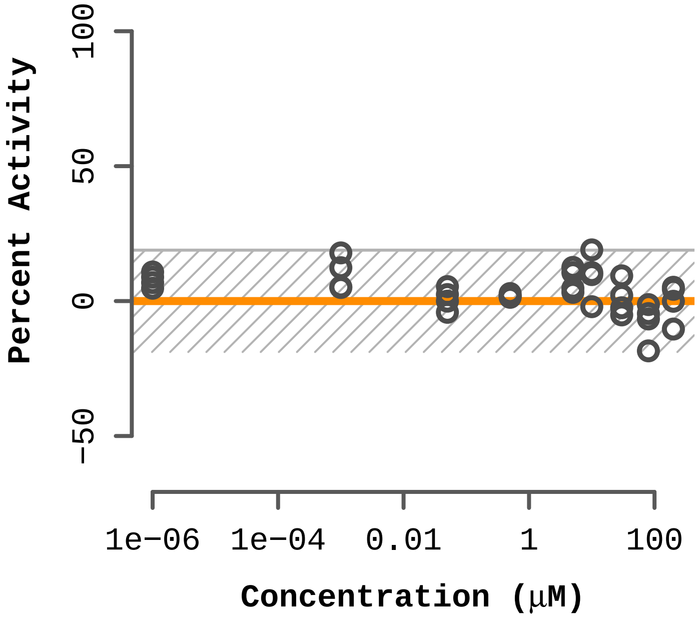

ASSAY: AEID3098 (CCTE_Deisenroth_DEVTOX_RUES2-GLR_Cel

NAME: Methyldopa sesquihydrate
CHID: 20863 CASRN: 41372-08-1
SPID(S): ES211_46_46
M4ID: 42676184

HILL MODEL: Not applicable.

GAIN-LOSS MODEL: Not applicable.

	CNST	HILL	GNLS
AIC:	257.13	NA	NA
PROB:	1	NA	NA
RMSE:	8.19	NA	NA

MAX_MEAN: 10.1 MAX_MED: 10.2 BMAD: 6.29

COFF: 18.9 HIT-CALL: 0 FITC: 4 ACTP: 0

FLAGS:

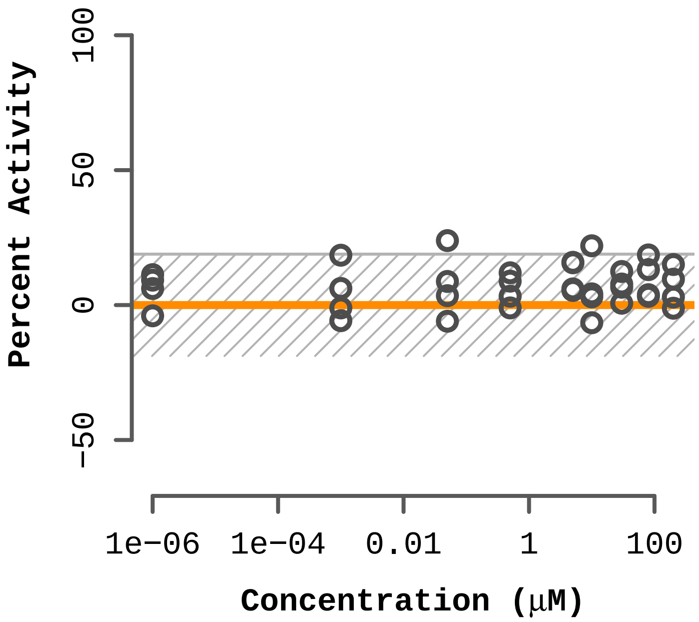

ASSAY: AEID3098 (CCTE_Deisenroth_DEVTOX_RUES2-GLR_Cel

NAME: Nicotinic acid
CHID: 20932 CASRN: 59−67−6
SPID(S): ES211_48_48
M4ID: 42676238

HILL MODEL: Not applicable.

GAIN−LOSS MODEL: Not applicable.

	CNST	HILL	GNLS
AIC:	274.93	NA	NA
PROB:	1	NA	NA
RMSE:	10.36	NA	NA

MAX_MEAN: 10.7 MAX_MED: 10.8 BMAD: 6.29

COFF: 18.9 HIT−CALL: 0 FITC: 4 ACTP: 0

FLAGS:

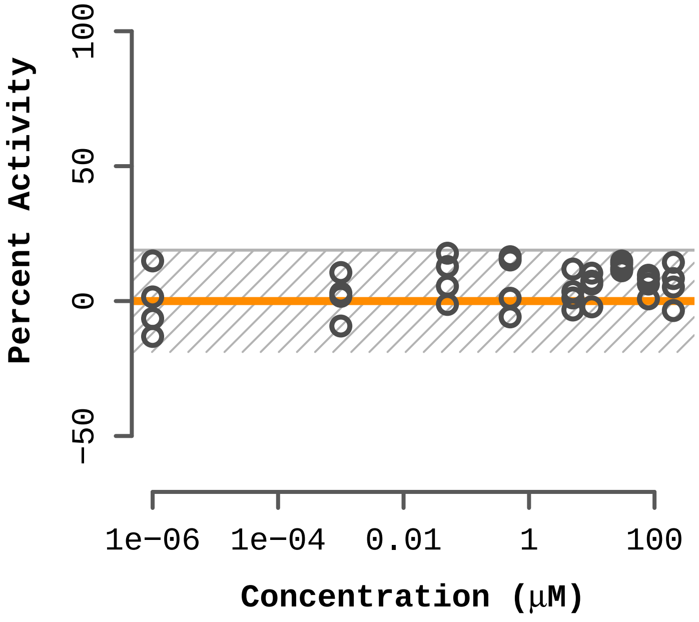

ASSAY: AEID3098 (CCTE_Deisenroth_DEVTOX_RUES2-GLR_Cel

NAME: 1,2-Propylene glycol
CHID: 21206 CASRN: 57-55-6
SPID(S): ES211_1_1
M4ID: 42676196

HILL MODEL: Not applicable.

GAIN-LOSS MODEL: Not applicable.

	CNST	HILL	GNLS
AIC:	271.37	NA	NA
PROB:	1	NA	NA
RMSE:	9.53	NA	NA

MAX_MEAN: 13 MAX_MED: 13 BMAD: 6.29

COFF: 18.9 HIT-CALL: 0 FITC: 4 ACTP: 0

FLAGS:

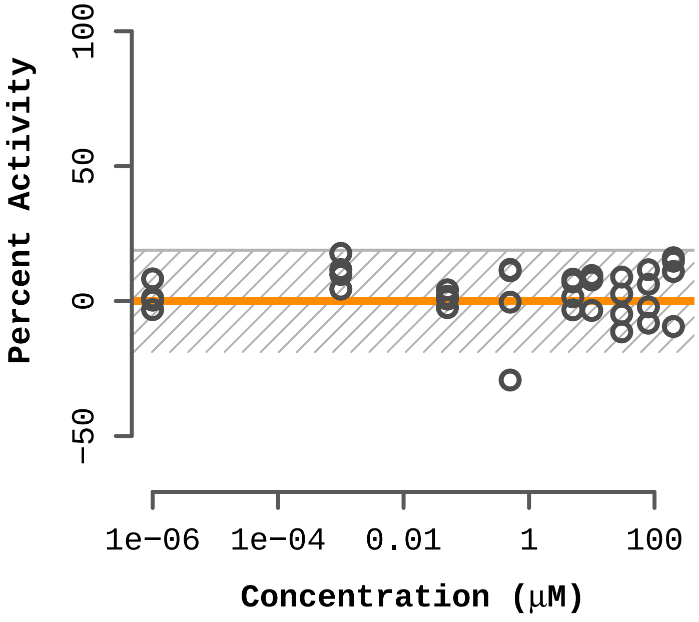

ASSAY: AEID3098 (CCTE_Deisenroth_DEVTOX_RUES2-GLR_Cel

NAME: 6-Propyl-2-thiouracil
CHID: 21209 CASRN: 51-52-5
SPID(S): ES211_8_8
M4ID: 42676182

HILL MODEL: Not applicable.

GAIN-LOSS MODEL: Not applicable.

	CNST	HILL	GNLS
AIC:	268.35	NA	NA
PROB:	1	NA	NA
RMSE:	9.63	NA	NA

MAX_MEAN: 10.9 MAX_MED: 12.9 BMAD: 6.29

COFF: 18.9 HIT-CALL: 0 FITC: 4 ACTP: 0

FLAGS:

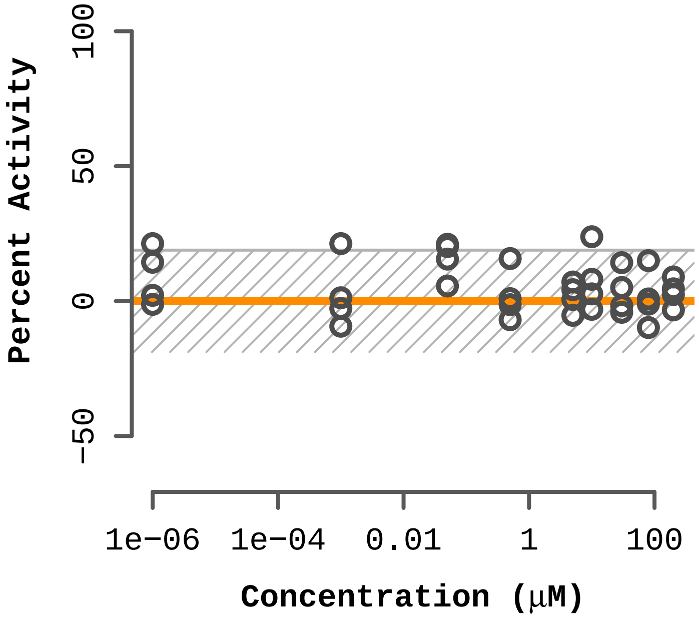

ASSAY: AEID3098 (CCTE_Deisenroth_DEVTOX_RUES2-GLR_Cel

NAME: Saccharin
CHID: 21251 CASRN: 81-07-2
SPID(S): ES211_54_54
M4ID: 42676229

HILL MODEL: Not applicable.

GAIN-LOSS MODEL: Not applicable.

	CNST	HILL	GNLS
AIC:	274.86	NA	NA
PROB:	1	NA	NA
RMSE:	10.64	NA	NA

MAX_MEAN: 15.6 MAX_MED: 17.9 BMAD: 6.29

COFF: 18.9 HIT-CALL: 0 FITC: 4 ACTP: 0

FLAGS:

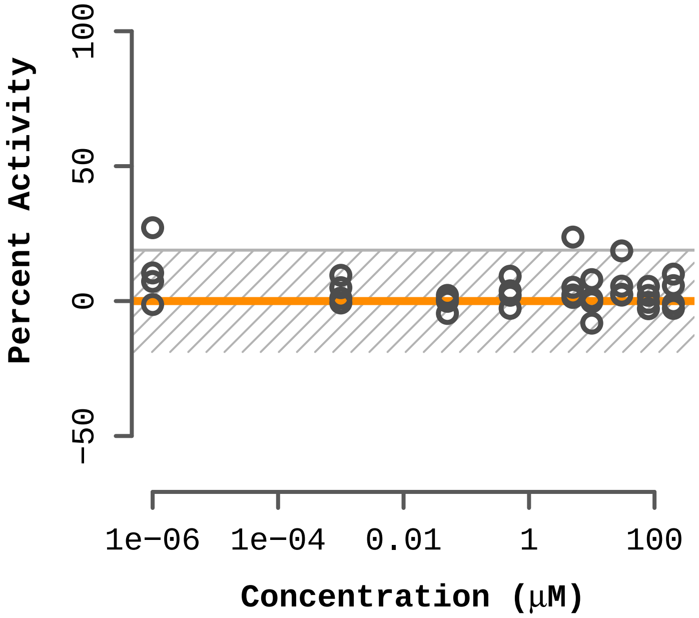

ASSAY: AEID3098 (CCTE_Deisenroth_DEVTOX_RUES2-GLR_Cel

NAME: Sulfasalazine
CHID: 21256 CASRN: 599-79-1
SPID(S): ES211_59_59
M4ID: 42676240

HILL MODEL: Not applicable.

GAIN-LOSS MODEL: Not applicable.

	CNST	HILL	GNLS
AIC:	247.51	NA	NA
PROB:	1	NA	NA
RMSE:	8.26	NA	NA

MAX_MEAN: 10.9 MAX_MED: 8.86 BMAD: 6.29

COFF: 18.9 HIT-CALL: 0 FITC: 4 ACTP: 0

FLAGS:

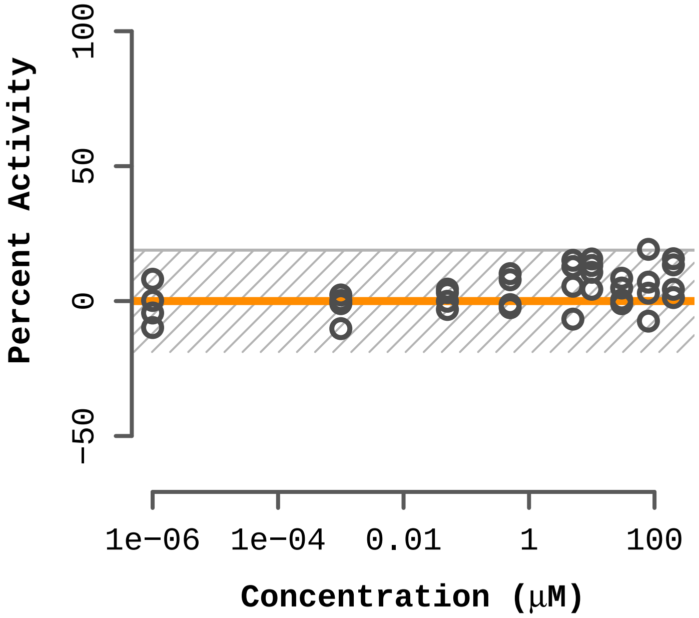

ASSAY: AEID3098 (CCTE_Deisenroth_DEVTOX_RUES2-GLR_Cel

NAME: Dimethyl phthalate
CHID: 22455 CASRN: 131-11-3
SPID(S): ES211_30_30
M4ID: 42676243

HILL MODEL: Not applicable.

GAIN-LOSS MODEL: Not applicable.

	CNST	HILL	GNLS
AIC:	260.09	NA	NA
PROB:	1	NA	NA
RMSE:	8.42	NA	NA

MAX_MEAN: 11 MAX_MED: 11.9 BMAD: 6.29

COFF: 18.9 HIT-CALL: 0 FITC: 4 ACTP: 0

FLAGS:

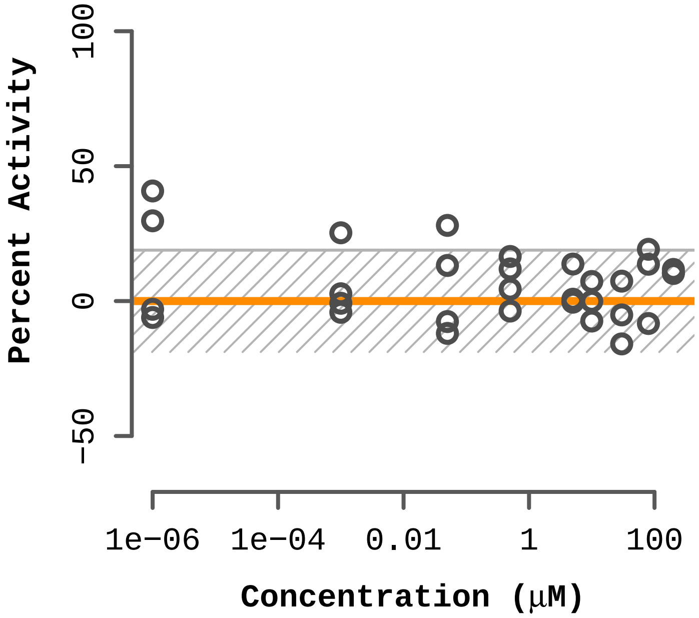

ASSAY: AEID3098 (CCTE_Deisenroth_DEVTOX_RUES2-GLR_Cel

NAME: Folic acid
CHID: 22519 CASRN: 59-30-3
SPID(S): ES211_33_33
M4ID: 42676206

HILL MODEL: Not applicable.

GAIN-LOSS MODEL: Not applicable.

	CNST	HILL	GNLS
AIC:	255.12	NA	NA
PROB:	1	NA	NA
RMSE:	14.46	NA	NA

MAX_MEAN: 15.4 MAX_MED: 13.7 BMAD: 6.29

COFF: 18.9 HIT-CALL: 0 FITC: 4 ACTP: 0

FLAGS:

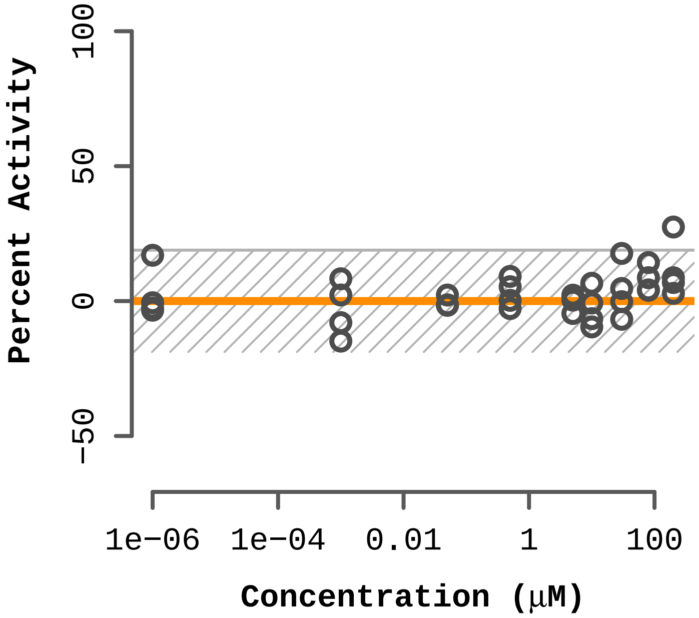

ASSAY: AEID3098 (CCTE_Deisenroth_DEVTOX_RUES2-GLR_Cel

NAME: Acyclovir
CHID: 22556 CASRN: 59277-89-3
SPID(S): ES211_12_12
M4ID: 42676216

HILL MODEL: Not applicable.

GAIN-LOSS MODEL: Not applicable.

	CNST	HILL	GNLS
AIC:	254.61	NA	NA
PROB:	1	NA	NA
RMSE:	8.47	NA	NA

MAX_MEAN: 11.5 MAX_MED: 7.77 BMAD: 6.29

COFF: 18.9 HIT-CALL: 0 FITC: 4 ACTP: 0

FLAGS:

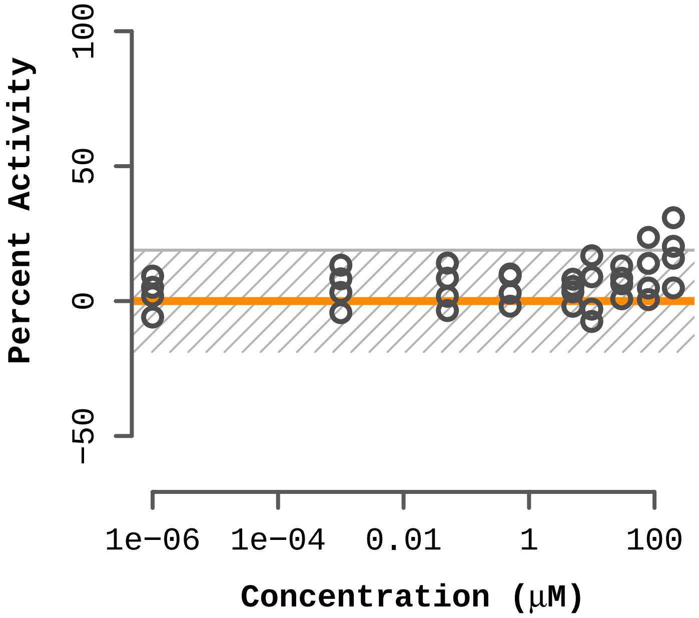

ASSAY: AEID3098 (CCTE_Deisenroth_DEVTOX_RUES2-GLR_Cel

NAME: Valproic acid
CHID: 23733 CASRN: 99−66−1
SPID(S): ES211_63_63
M4ID: 42676218

HILL MODEL: Not applicable.

GAIN−LOSS MODEL: Not applicable.

	CNST	HILL	GNLS
AIC:	275.23	NA	NA
PROB:	1	NA	NA
RMSE:	10.75	NA	NA

MAX_MEAN: 18 MAX_MED: 18.1 BMAD: 6.29

COFF: 18.9 HIT−CALL: 0 FITC: 4 ACTP: 0

FLAGS:

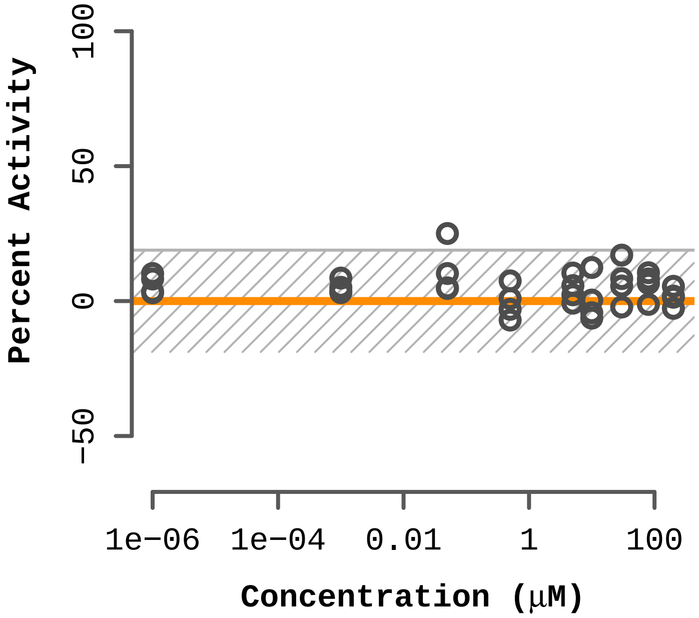

ASSAY: AEID3098 (CCTE_Deisenroth_DEVTOX_RUES2-GLR_Cel

NAME: D-Camphor
CHID: 24721 CASRN: 464-49-3
SPID(S): ES211_27_27
M4ID: 42676234

HILL MODEL: Not applicable.

GAIN-LOSS MODEL: Not applicable.

	CNST	HILL	GNLS
AIC:	252.32	NA	NA
PROB:	1	NA	NA
RMSE:	7.87	NA	NA

MAX_MEAN: 11.2 MAX_MED: 7.56 BMAD: 6.29

COFF: 18.9 HIT-CALL: 0 FITC: 4 ACTP: 0

FLAGS:

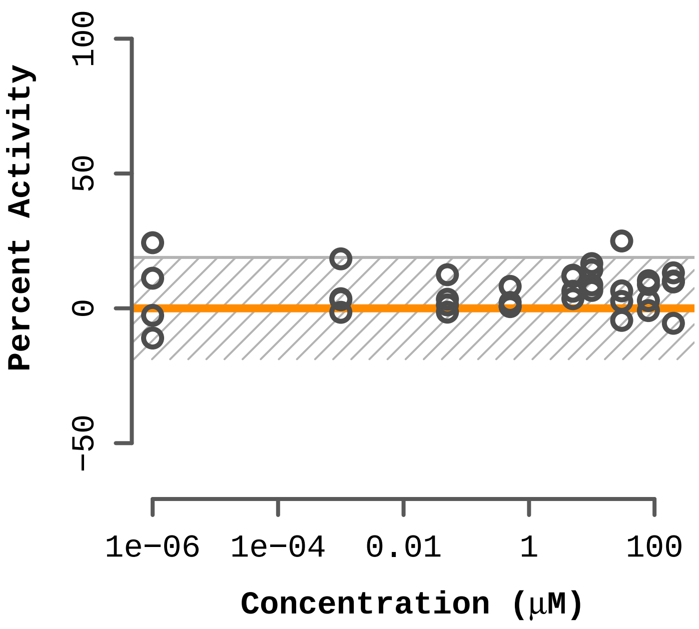

ASSAY: AEID3098 (CCTE_Deisenroth_DEVTOX_RUES2-GLR_Cel[illegible]

NAME: Salicylic acid
CHID: 26368 CASRN: 69-72-7
SPID(S): ES211_55_55
M4ID: 42676197

HILL MODEL: Not applicable.

GAIN-LOSS MODEL: Not applicable.

	CNST	HILL	GNLS
AIC:	272.2	NA	NA
PROB:	1	NA	NA
RMSE:	10.11	NA	NA

MAX_MEAN: 11.6 MAX_MED: 11.5 BMAD: 6.29

COFF: 18.9 HIT-CALL: 0 FITC: 4 ACTP: 0

FLAGS:

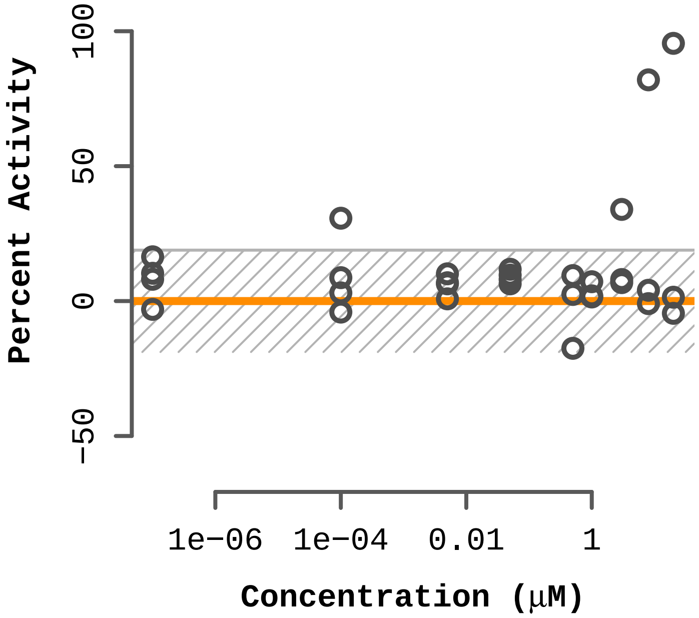

ASSAY: AEID3098 (CCTE_Deisenroth_DEVTOX_RUES2-GLR_Cel[illegible]

NAME: Cyclopamine
CHID: 43709 CASRN: 4449-51-8
SPID(S): ES211_23_23
M4ID: 42676177

HILL MODEL: Not applicable.

GAIN-LOSS MODEL: Not applicable.

	CNST	HILL	GNLS
AIC:	266.73	NA	NA
PROB:	1	NA	NA
RMSE:	25.17	NA	NA

MAX_MEAN: 30.8 MAX_MED: 9.2 BMAD: 6.29

COFF: 18.9 HIT-CALL: 0 FITC: 4 ACTP: 0

FLAGS: 10

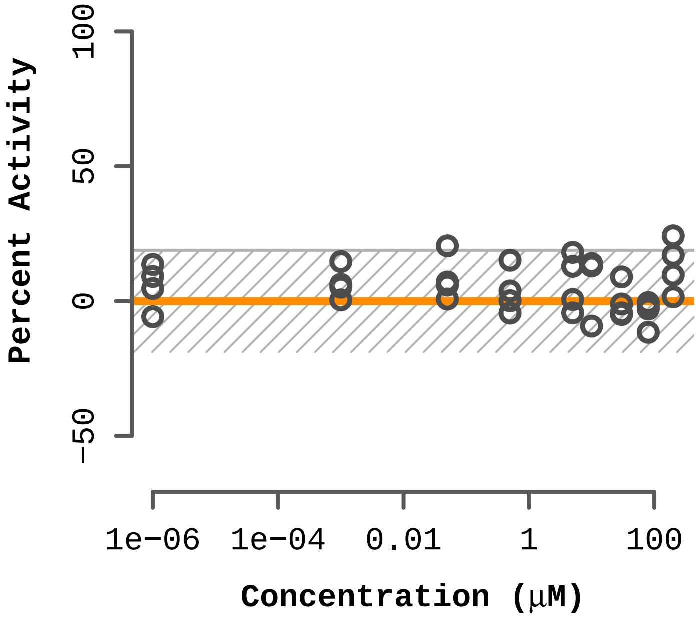

ASSAY: AEID3098 (CCTE_Deisenroth_DEVTOX_RUES2-GLR_Cel

NAME: Penicillin G potassium
CHID: 45106 CASRN: 113-98-4
SPID(S): ES211_49_49
M4ID: 42676180

HILL MODEL: Not applicable.

GAIN-LOSS MODEL: Not applicable.

	CNST	HILL	GNLS
AIC:	274.19	NA	NA
PROB:	1	NA	NA
RMSE:	10.23	NA	NA

MAX_MEAN: 13.1 MAX_MED: 13.3 BMAD: 6.29

COFF: 18.9 HIT-CALL: 0 FITC: 4 ACTP: 0

FLAGS:

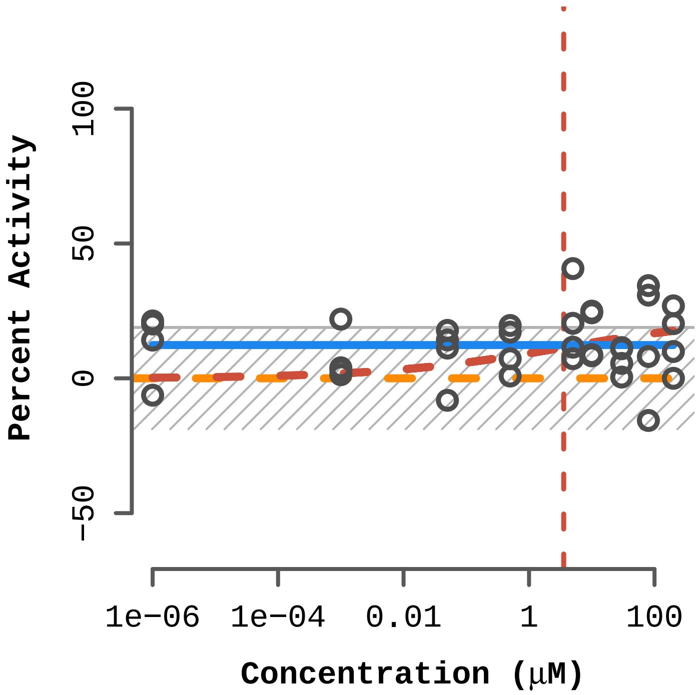

ASSAY: AEID3098 (CCTE_Deisenroth_DEVTOX_RUES2-GLR_Cel

NAME: Thalidomide
CHID: 22524 CASRN: 50-35-1
SPID(S): ES211_62_62
M4ID: 42676213

HILL MODEL (in red):

	tp	ga	gw
val:	22.9	0.553	0.3
sd:	NaN	NaN	NaN

GAIN-LOSS MODEL (in blue):

	tp	ga	gw	la	lw
val:	12.3	-7	6.53	4.06	10.3
sd:	NA	NA	NA	NA	NA

	CNST	HILL	GNLS
AIC:	312.26	295.66	293.36
PROB:	0	0.24	0.76
RMSE:	17.19	12.75	11.81

MAX_MEAN: 20 MAX_MED: 19.5 BMAD: 6.29

COFF: 18.9 HIT-CALL: 0 FITC: 27 ACTP: 1

FLAGS: 12

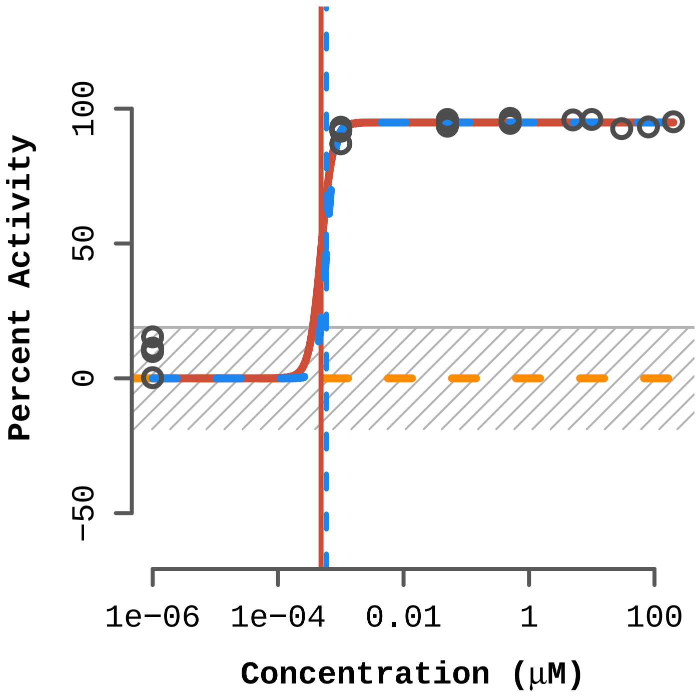

ASSAY: AEID3098 (CCTE_Deisenroth_DEVTOX_RUES2-GLR_Cel

NAME: Actinomycin D
CHID: 20031 CASRN: 50-76-0
SPID(S): ES211_11_11
M4ID: 42676222

HILL MODEL (in red):

	tp	ga	gw
val:	94.9	-3.32	4.59
sd:	0.57	0.176	2.54

GAIN-LOSS MODEL (in blue):

	tp	ga	gw	la	lw
val:	94.9	-3.23	6.31	3.39	12.2
sd:	0.57	0.08	2.17	93900	1210000

	CNST	HILL	GNLS
AIC:	327.56	122.79	126.79
PROB:	0	0.88	0.12
RMSE:	84.69	4.89	4.89

MAX_MEAN: 96 MAX_MED: 96 BMAD: 6.29

COFF: 18.9 HIT-CALL: 1 FITC: 41 ACTP: 1

FLAGS:

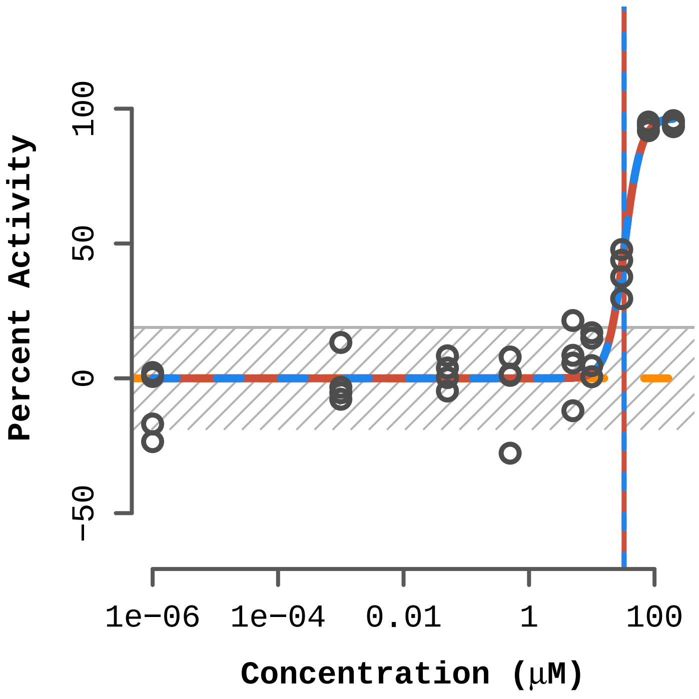

ASSAY: AEID3098 (CCTE_Deisenroth_DEVTOX_RUES2-GLR_Cel

NAME: Bisphenol A
CHID: 20182 CASRN: 80-05-7
SPID(S): ES211_16_16
M4ID: 42676195

HILL MODEL (in red):

	tp	ga	gw
val:	96.6	1.52	3.22
sd:	3.54	0.0256	1.1

GAIN-LOSS MODEL (in blue):

	tp	ga	gw	la	lw
val:	96.6	1.52	3.22	4.26	11.9
sd:	NA	NA	NA	NA	NA

	CNST	HILL	GNLS
AIC:	378.61	270.76	274.76
PROB:	0	0.88	0.12
RMSE:	47.32	9.72	9.72

MAX_MEAN: 94.4 MAX_MED: 94.4 BMAD: 6.29

COFF: 18.9 HIT-CALL: 1 FITC: 41 ACTP: 1

FLAGS:

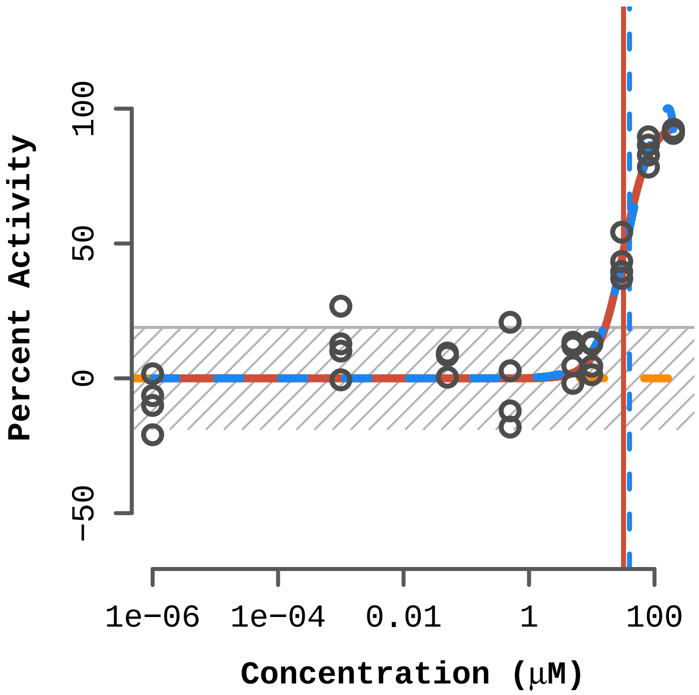

ASSAY: AEID3098 (CCTE_Deisenroth_DEVTOX_RUES2-GLR_Cel

NAME: 4-Hydroxybenzoic acid butyl ester
CHID: 20209 CASRN: 94-26-8
SPID(S): ES211_19_19
M4ID: 42676192

HILL MODEL (in red):

	tp	ga	gw
val:	94.9	1.51	2.07
sd:	4.32	0.0415	0.403

GAIN-LOSS MODEL (in blue):

	tp	ga	gw	la	lw
val:	111	1.6	1.67	2.37	12.6
sd:	27	0.164	0.508	0.0184	9.71

	CNST	HILL	GNLS
AIC:	376.27	272.74	275.77
PROB:	0	0.82	0.18
RMSE:	45.14	9.64	9.57

MAX_MEAN: 91.7 MAX_MED: 91.8 BMAD: 6.29

COFF: 18.9 HIT-CALL: 1 FITC: 41 ACTP: 1

FLAGS:

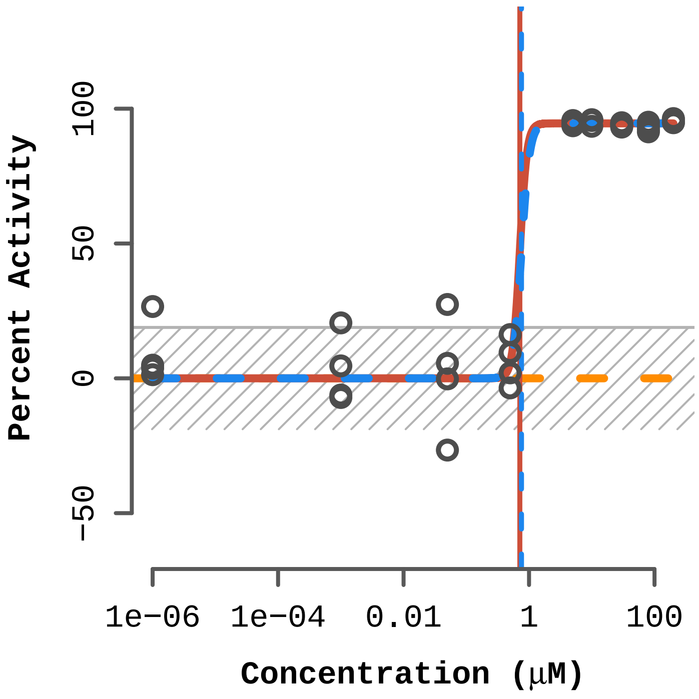

ASSAY: AEID3098 (CCTE_Deisenroth_DEVTOX_RUES2-GLR_Cel

NAME: Diethylstilbestrol
CHID: 20465 CASRN: 56-53-1
SPID(S): ES211_29_29
M4ID: 42676242

HILL MODEL (in red):

	tp	ga	gw
val:	94.6	-0.147	7.7
sd:	0.876	5.26	262

GAIN-LOSS MODEL (in blue):

	tp	ga	gw	la	lw
val:	94.6	-0.12	6.59	3.26	8.39
sd:	0.876	2.37	86.4	337	2950

	CNST	HILL	GNLS
AIC:	436.24	251.18	255.18
PROB:	0	0.88	0.12
RMSE:	71.09	9.17	9.17

MAX_MEAN: 95.5 MAX_MED: 95.7 BMAD: 6.29

COFF: 18.9 HIT-CALL: 1 FITC: 41 ACTP: 1

FLAGS:

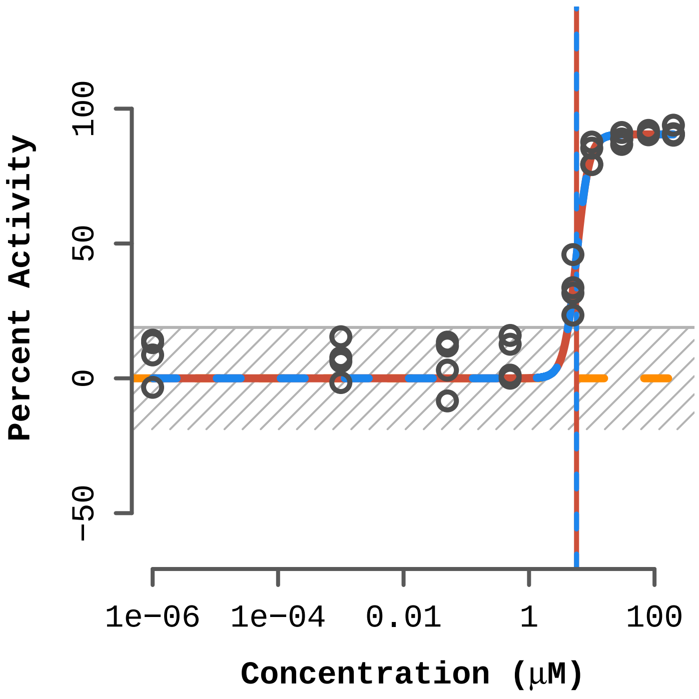

ASSAY: AEID3098 (CCTE_Deisenroth_DEVTOX_RUES2-GLR_Cel

NAME: 5-Fluorouracil
CHID: 20634 CASRN: 51-21-8
SPID(S): ES211_5_5
M4ID: 42676205

HILL MODEL (in red):

	tp	ga	gw
val:	90.5	0.757	4.26
sd:	1.53	0.0176	0.714

GAIN-LOSS MODEL (in blue):

	tp	ga	gw	la	lw
val:	90.5	0.757	4.26	3.81	17.8
sd:	NA	NA	NA	NA	NA

	CNST	HILL	GNLS
AIC:	403.55	254.25	258.25
PROB:	0	0.88	0.12
RMSE:	60.58	7.4	7.4

MAX_MEAN: 91.4 MAX_MED: 91 BMAD: 6.29

COFF: 18.9 HIT-CALL: 1 FITC: 41 ACTP: 1

FLAGS:

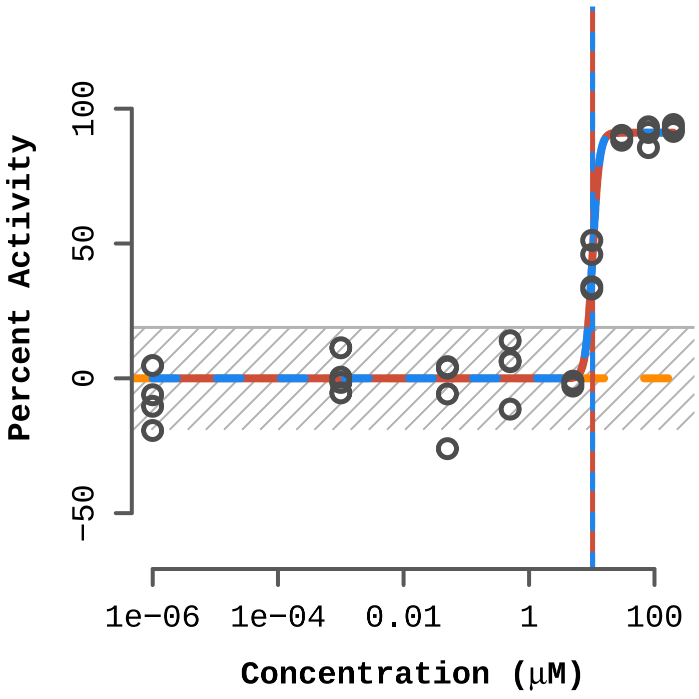

ASSAY: AEID3098 (CCTE_Deisenroth_DEVTOX_RUES2-GLR_Cel

NAME: 1-(p-Chlorobenzoyl)-5-methoxy-2-methyl-Indole-
CHID: 20740 CASRN: 53-86-1
SPID(S): ES211_38_38
M4ID: 42676225

HILL MODEL (in red):

	tp	ga	gw
val:	91.1	1.01	8
sd:	1.5	0.0153	5.39

GAIN-LOSS MODEL (in blue):

	tp	ga	gw	la	lw
val:	91.1	1.01	8	4.25	12
sd:	NA	NA	NA	NA	NA

	CNST	HILL	GNLS
AIC:	393.92	252.82	256.82
PROB:	0	0.88	0.12
RMSE:	54.85	7.78	7.78

MAX_MEAN: 92.7 MAX_MED: 92.5 BMAD: 6.29

COFF: 18.9 HIT-CALL: 1 FITC: 41 ACTP: 1

FLAGS:

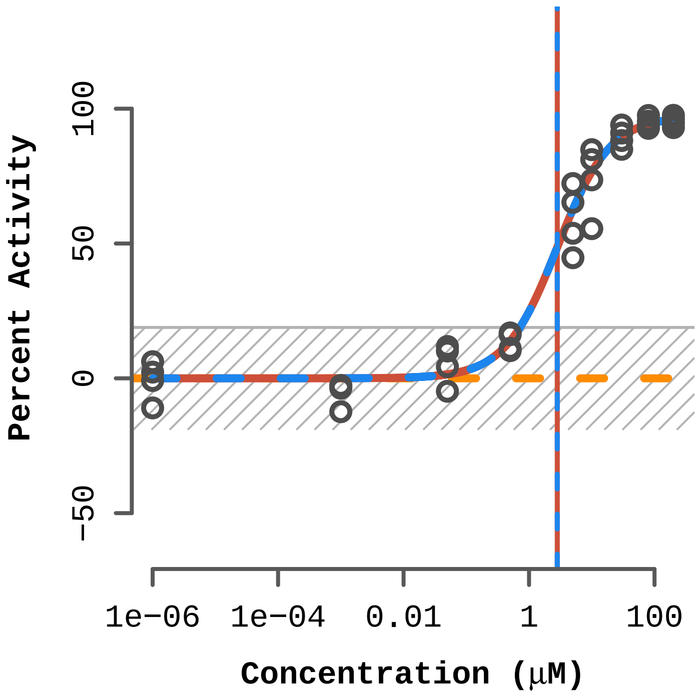

ASSAY: AEID3098 (CCTE_Deisenroth_DEVTOX_RUES2-GLR_Cel

NAME: Lovastatin
CHID: 20784 CASRN: 75330-75-5
SPID(S): ES211_42_42
M4ID: 42676194

HILL MODEL (in red):

	tp	ga	gw
val:	97.2	0.45	1.04
sd:	2.13	0.0633	0.105

GAIN-LOSS MODEL (in blue):

	tp	ga	gw	la	lw
val:	97.2	0.45	1.04	4.3	8.99
sd:	NA	NA	NA	NA	NA

	CNST	HILL	GNLS
AIC:	406.85	246.02	250.02
PROB:	0	0.88	0.12
RMSE:	62.93	6.92	6.92

MAX_MEAN: 95.3 MAX_MED: 95.4 BMAD: 6.29

COFF: 18.9 HIT-CALL: 1 FITC: 41 ACTP: 1

FLAGS:

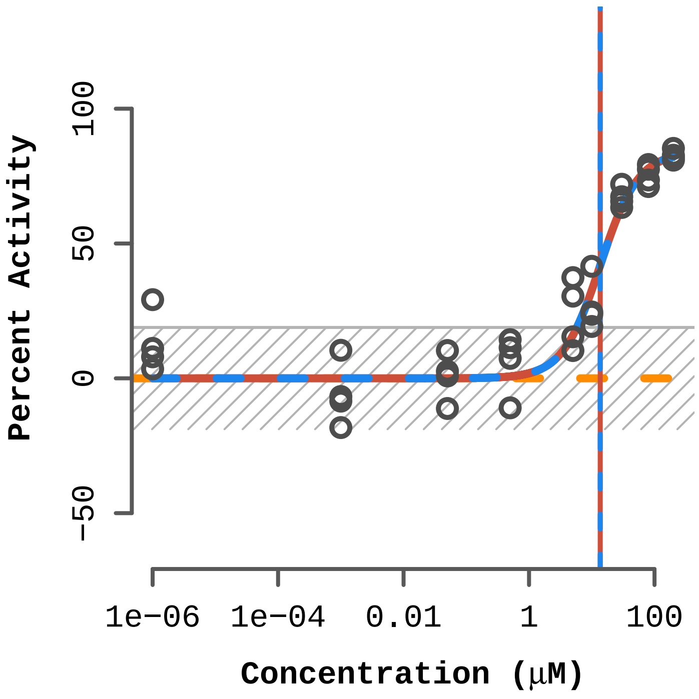

ASSAY: AEID3098 (CCTE_Deisenroth_DEVTOX_RUES2-GLR_Cel

NAME: Busulfan
CHID: 20910 CASRN: 55-98-1
SPID(S): ES211_18_18
M4ID: 42676183

HILL MODEL (in red):

	tp	ga	gw
val:	83.8	1.13	1.44
sd:	4.5	0.0675	0.356

GAIN-LOSS MODEL (in blue):

	tp	ga	gw	la	lw
val:	83.8	1.13	1.44	3.99	15.9
sd:	NA	NA	NA	NA	NA

	CNST	HILL	GNLS
AIC:	382.06	276.61	280.61
PROB:	0	0.88	0.12
RMSE:	46.1	10.01	10.01

MAX_MEAN: 82.7 MAX_MED: 82.3 BMAD: 6.29

COFF: 18.9 HIT-CALL: 1 FITC: 41 ACTP: 1

FLAGS:

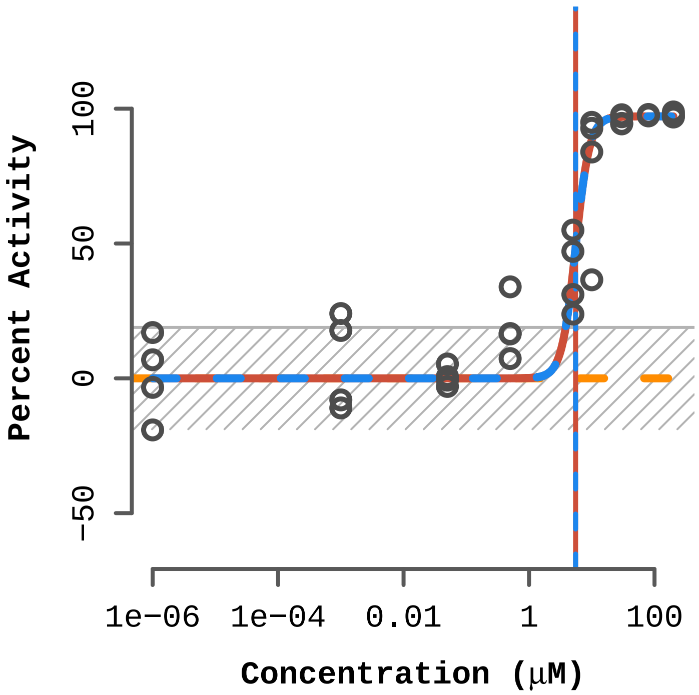

ASSAY: AEID3098 (CCTE_Deisenroth_DEVTOX_RUES2-GLR_Cel

NAME: all-trans-Retinoic acid
CHID: 21239 CASRN: 302-79-4
SPID(S): ES211_13_13
M4ID: 42676210

HILL MODEL (in red):

	tp	ga	gw
val:	97.2	0.742	3.9
sd:	2.32	0.0319	1.14

GAIN-LOSS MODEL (in blue):

	tp	ga	gw	la	lw
val:	97.2	0.742	3.9	3.11	8.97
sd:	2.32	0.0319	1.14	338	3720

	CNST	HILL	GNLS
AIC:	408	290.67	294.67
PROB:	0	0.88	0.12
RMSE:	64.44	13.93	13.93

MAX_MEAN: 97.7 MAX_MED: 97.6 BMAD: 6.29

COFF: 18.9 HIT-CALL: 1 FITC: 41 ACTP: 1

FLAGS:

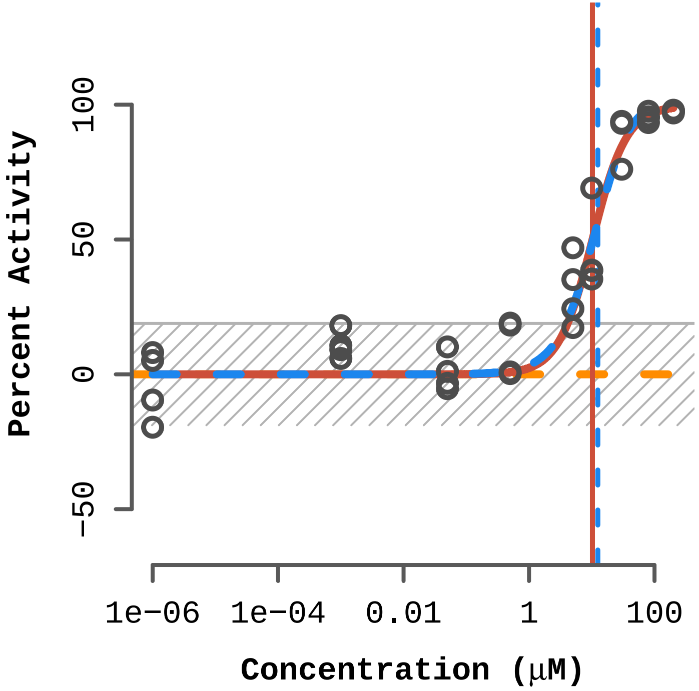

ASSAY: AEID3098 (CCTE_Deisenroth_DEVTOX_RUES2-GLR_Cel

NAME: 13-cis Retinoic acid
CHID: 23177 CASRN: 4759-48-2
SPID(S): ES211_2_2
M4ID: 42676208

HILL MODEL (in red):

	tp	ga	gw
val:	99.7	1.01	1.6
sd:	3.57	0.0628	0.346

GAIN-LOSS MODEL (in blue):

	tp	ga	gw	la	lw
val:	118	1.1	1.38	3.68	0.529
sd:	45.7	0.161	0.416	1.85	1.1

	CNST	HILL	GNLS
AIC:	399.18	279.48	282.51
PROB:	0	0.82	0.18
RMSE:	58.18	10.19	10.04

MAX_MEAN: 97.6 MAX_MED: 97.7 BMAD: 6.29

COFF: 18.9 HIT-CALL: 1 FITC: 41 ACTP: 1

FLAGS:

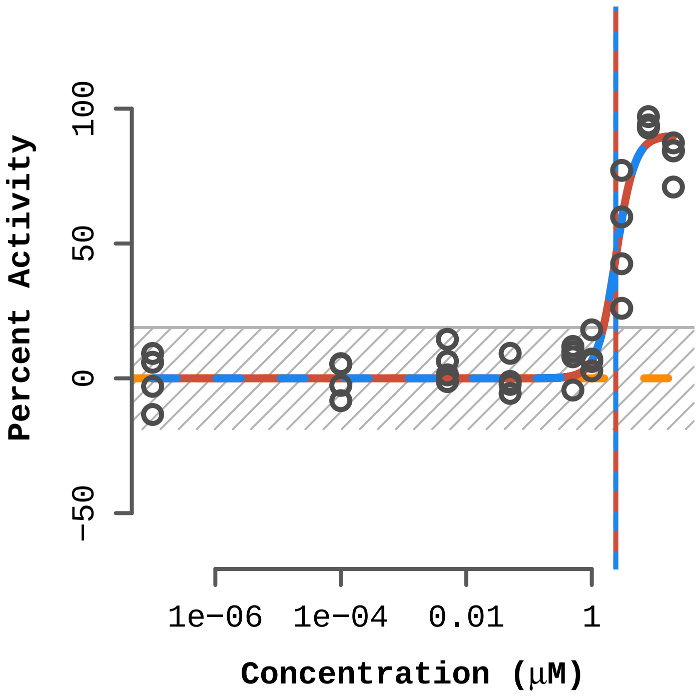

ASSAY: AEID3098 (CCTE_Deisenroth_DEVTOX_RUES2-GLR_Cel

NAME: Ziprasidone
CHID: 23753 CASRN: 146939-27-7
SPID(S): ES211_66_66
M4ID: 42676186

HILL MODEL (in red):

	tp	ga	gw
val:	90	0.384	2.88
sd:	3.59	0.0552	0.632

GAIN-LOSS MODEL (in blue):

	tp	ga	gw	la	lw
val:	90	0.384	2.88	2.41	12.5
sd:	3.59	0.0552	0.632	59300	811000

	CNST	HILL	GNLS
AIC:	376.67	273.73	277.73
PROB:	0	0.88	0.12
RMSE:	45.98	9.92	9.92

MAX_MEAN: 94.3 MAX_MED: 93.7 BMAD: 6.29

COFF: 18.9 HIT-CALL: 1 FITC: 41 ACTP: 1

FLAGS:

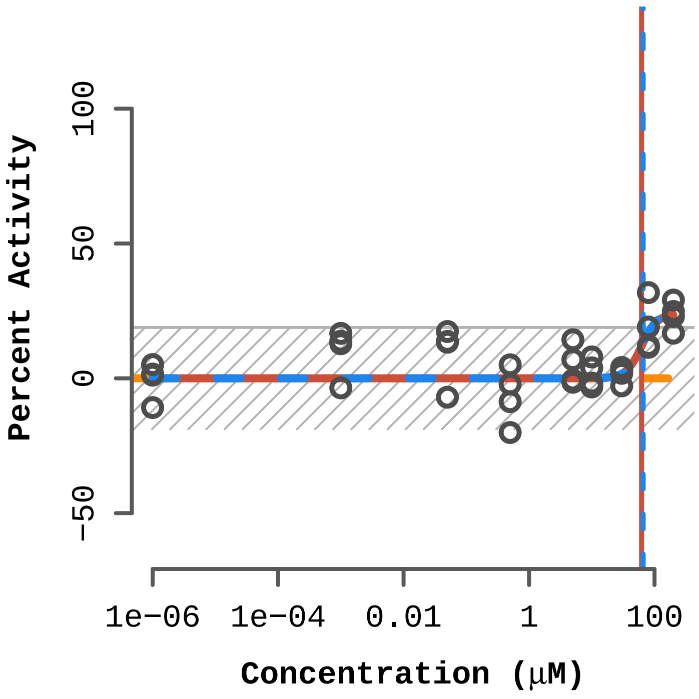

ASSAY: AEID3098 (CCTE_Deisenroth_DEVTOX_RUES2-GLR_Cel

NAME: Stavudine
CHID: 23819 CASRN: 3056−17−5
SPID(S): ES211_58_58
M4ID: 42676207

HILL MODEL (in red):

	tp	ga	gw
val:	23.9	1.79	3.56
sd:	4.16	0.119	2.62

GAIN−LOSS MODEL (in blue):

	tp	ga	gw	la	lw
val:	29.6	1.82	3.45	3.69	0.456
sd:	NaN	NaN	NaN	NaN	NaN

	CNST	HILL	GNLS
AIC:	291.98	267.74	271.74
PROB:	0	0.88	0.12
RMSE:	13.25	8.74	8.74

MAX_MEAN: 23.4 MAX_MED: 23.9 BMAD: 6.29

COFF: 18.9 HIT−CALL: 1 FITC: 41 ACTP: 1

FLAGS: 7; 17

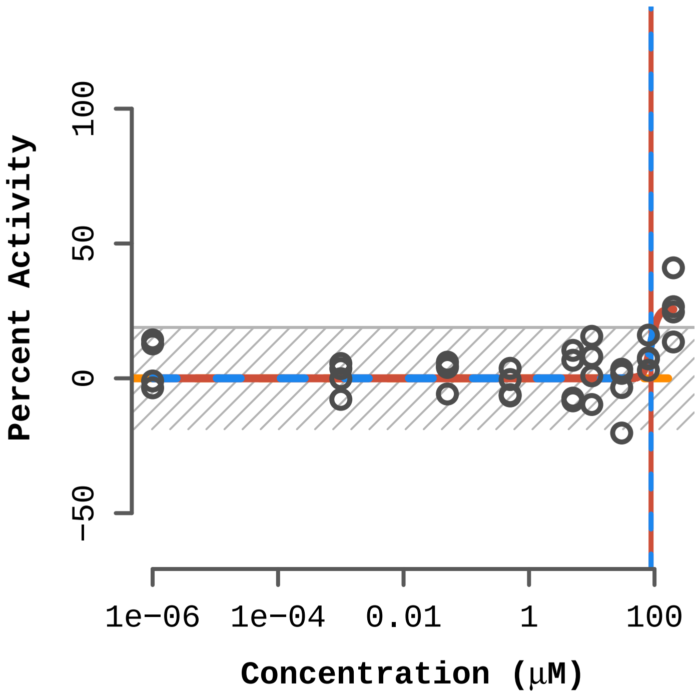

ASSAY: AEID3098 (CCTE_Deisenroth_DEVTOX_RUES2-GLR_Cel

NAME: Cyclophosphamide monohydrate
CHID: 24888 CASRN: 6055−19−2
SPID(S): ES211_24_24
M4ID: 42676224

HILL MODEL (in red):

	tp	ga	gw
val:	25.6	1.94	8
sd:	4.3	0.145	26.9

GAIN−LOSS MODEL (in blue):

	tp	ga	gw	la	lw
val:	25.6	1.94	8	3.72	11.1
sd:	NA	NA	NA	NA	NA

	CNST	HILL	GNLS
AIC:	280.31	261.16	265.16
PROB:	0	0.88	0.12
RMSE:	12.2	7.95	7.95

MAX_MEAN: 26.4 MAX_MED: 25.6 BMAD: 6.29

COFF: 18.9 HIT−CALL: 1 FITC: 41 ACTP: 1

FLAGS: 7; 17

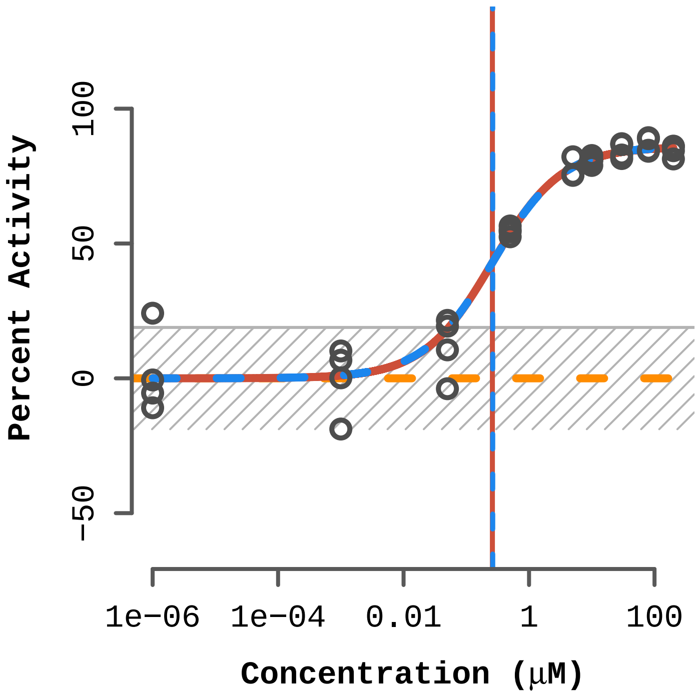

ASSAY: AEID3098 (CCTE_Deisenroth_DEVTOX_RUES2-GLR_Cel

NAME: Cytarabine hydrochloride
CHID: 24891 CASRN: 69−74−9
SPID(S): ES211_25_25
M4ID: 42676179

HILL MODEL (in red):

	tp	ga	gw
val:	85.9	−0.584	0.789
sd:	1.37	0.0507	0.0862

GAIN−LOSS MODEL (in blue):

	tp	ga	gw	la	lw
val:	86.2	−0.579	0.779	4.3	1.11
sd:	NaN	NaN	NaN	NaN	NaN

	CNST	HILL	GNLS
AIC:	410.86	237.96	241.9
PROB:	0	0.88	0.12
RMSE:	64.86	7.35	7.36

MAX_MEAN: 86.7 MAX_MED: 86.7 BMAD: 6.29

COFF: 18.9 HIT−CALL: 1 FITC: 41 ACTP: 1

FLAGS:

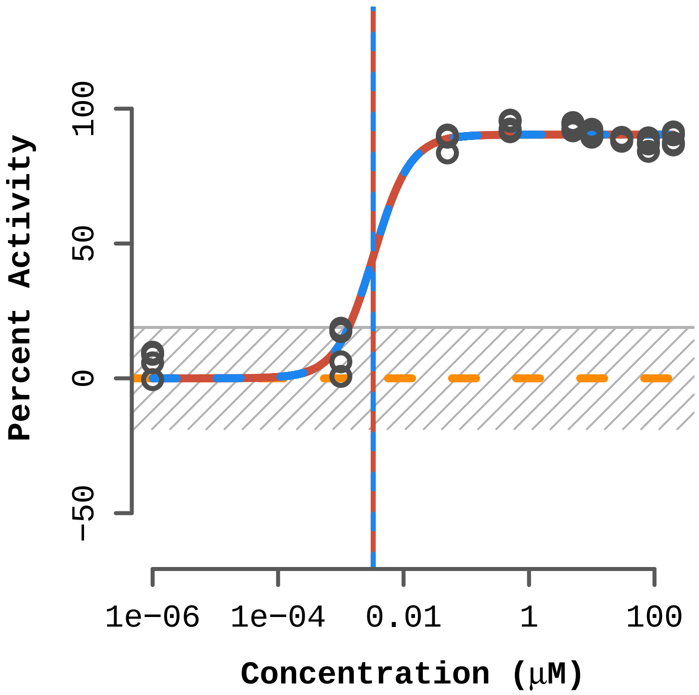

ASSAY: AEID3098 (CCTE_Deisenroth_DEVTOX_RUES2-GLR_Cel

NAME: Adriamycin hydrochloride
CHID: 30636 CASRN: 25316-40-9
SPID(S): ES211_67_67
M4ID: 42676193

HILL MODEL (in red):

	tp	ga	gw
val:	90.4	−2.48	1.47
sd:	0.753	0.146	0.316

GAIN-LOSS MODEL (in blue):

	tp	ga	gw	la	lw
val:	90.4	−2.48	1.47	3.25	11.6
sd:	NaN	NaN	NaN	NaN	NaN

	CNST	HILL	GNLS
AIC:	490.14	215.88	219.88
PROB:	0	0.88	0.12
RMSE:	79.67	4.39	4.39

MAX_MEAN: 93.8 MAX_MED: 93.9 BMAD: 6.29

COFF: 18.9 HIT-CALL: 1 FITC: 41 ACTP: 1

FLAGS:

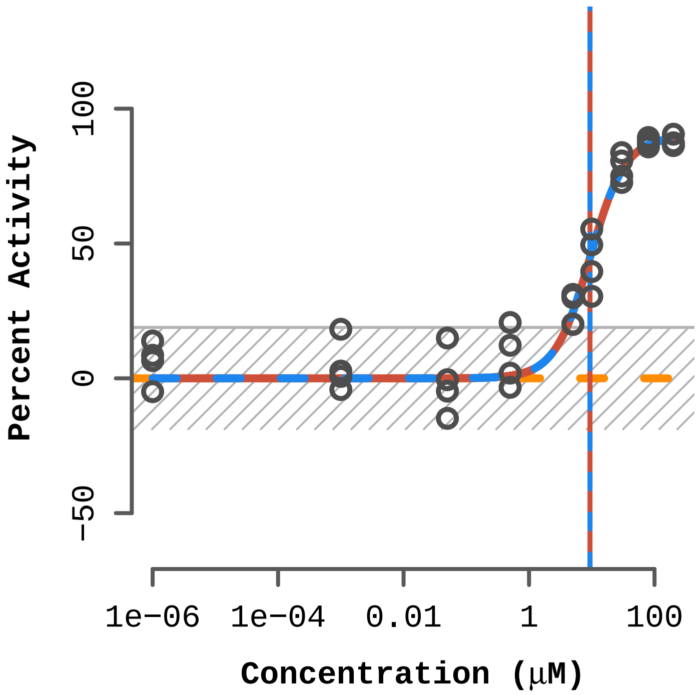

ASSAY: AEID3098 (CCTE_Deisenroth_DEVTOX_RUES2-GLR_Cel

NAME: 5-Bromo-2'-deoxyuridine
CHID: 33105 CASRN: 59-14-3
SPID(S): ES211_4_4
M4ID: 42676176

HILL MODEL (in red):

	tp	ga	gw
val:	89.7	0.972	1.55
sd:	2.7	0.0446	0.241

GAIN-LOSS MODEL (in blue):

	tp	ga	gw	la	lw
val:	89.7	0.972	1.55	3.78	8.93
sd:	NaN	NaN	NaN	NaN	NaN

	CNST	HILL	GNLS
AIC:	391.52	259.49	263.49
PROB:	0	0.88	0.12
RMSE:	52.26	8.04	8.04

MAX_MEAN: 87.7 MAX_MED: 87.6 BMAD: 6.29

COFF: 18.9 HIT-CALL: 1 FITC: 41 ACTP: 1

FLAGS:

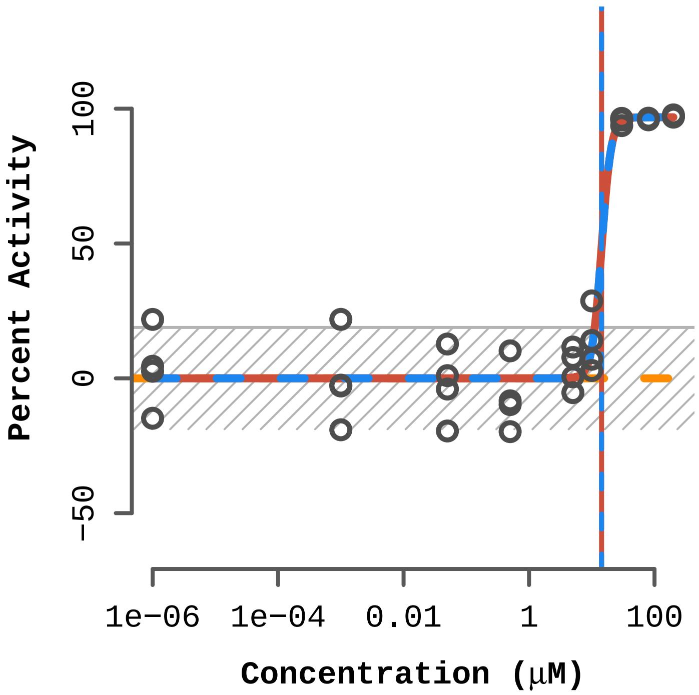

ASSAY: AEID3098 (CCTE_Deisenroth_DEVTOX_RUES2-GLR_Cel

NAME: Amiodarone hydrochloride
CHID: 37185 CASRN: 19774-82-4
SPID(S): ES211_14_14
M4ID: 42676232

HILL MODEL (in red):

	tp	ga	gw
val:	96.8	1.16	5.64
sd:	2.25	0.0694	2.43

GAIN-LOSS MODEL (in blue):

	tp	ga	gw	la	lw
val:	96.8	1.16	5.64	3.09	10
sd:	2.25	0.0694	2.43	594	7520

	CNST	HILL	GNLS
AIC:	394.9	272.56	276.56
PROB:	0	0.88	0.12
RMSE:	56.63	9.75	9.75

MAX_MEAN: 97.2 MAX_MED: 97.1 BMAD: 6.29

COFF: 18.9 HIT-CALL: 1 FITC: 41 ACTP: 1

FLAGS:

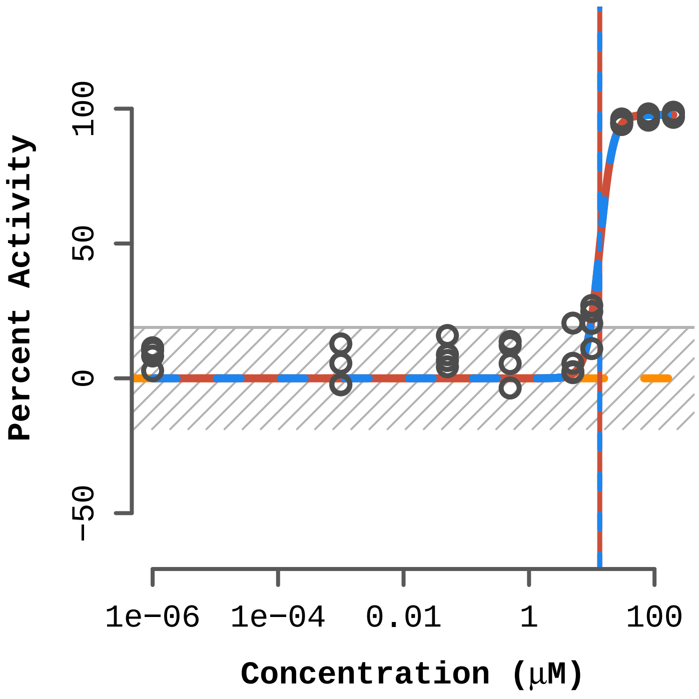

ASSAY: AEID3098 (CCTE_Deisenroth_DEVTOX_RUES2-GLR_Cel

NAME: Imatinib mesylate
CHID: 40502 CASRN: 220127-57-1
SPID(S): ES211_37_37
M4ID: 42676220

HILL MODEL (in red):

	tp	ga	gw
val:	97.7	1.13	4.11
sd:	1.67	0.027	0.753

GAIN-LOSS MODEL (in blue):

	tp	ga	gw	la	lw
val:	97.7	1.13	4.11	2.74	15.2
sd:	1.67	0.027	0.754	62.6	2010

	CNST	HILL	GNLS
AIC:	394.76	250.01	254.01
PROB:	0	0.88	0.12
RMSE:	56.66	7.14	7.14

MAX_MEAN: 97.8 MAX_MED: 97.8 BMAD: 6.29

COFF: 18.9 HIT-CALL: 1 FITC: 41 ACTP: 1

FLAGS:

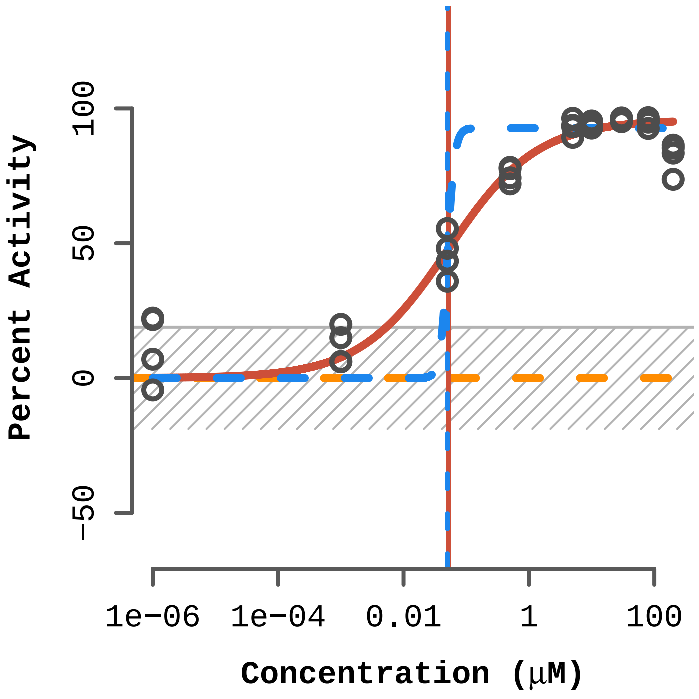

ASSAY: AEID3098 (CCTE_Deisenroth_DEVTOX_RUES2-GLR_Cel

NAME: Dasatinib
CHID: 40979 CASRN: 302962-49-8
SPID(S): ES211_26_26
M4ID: 42676188

HILL MODEL (in red):

	tp	ga	gw
val:	95.7	−1.28	0.617
sd:	1.88	0.0937	0.0755

GAIN-LOSS MODEL (in blue):

	tp	ga	gw	la	lw
val:	92.7	−1.3	7.5	2.36	15
sd:	NaN	NaN	NaN	NaN	NaN

	CNST	HILL	GNLS
AIC:	422.07	254.4	275.29
PROB:	0	1	0
RMSE:	75.12	8.07	9.64

MAX_MEAN: 95.9 MAX_MED: 96 BMAD: 6.29

COFF: 18.9 HIT-CALL: 1 FITC: 41 ACTP: 1

FLAGS:

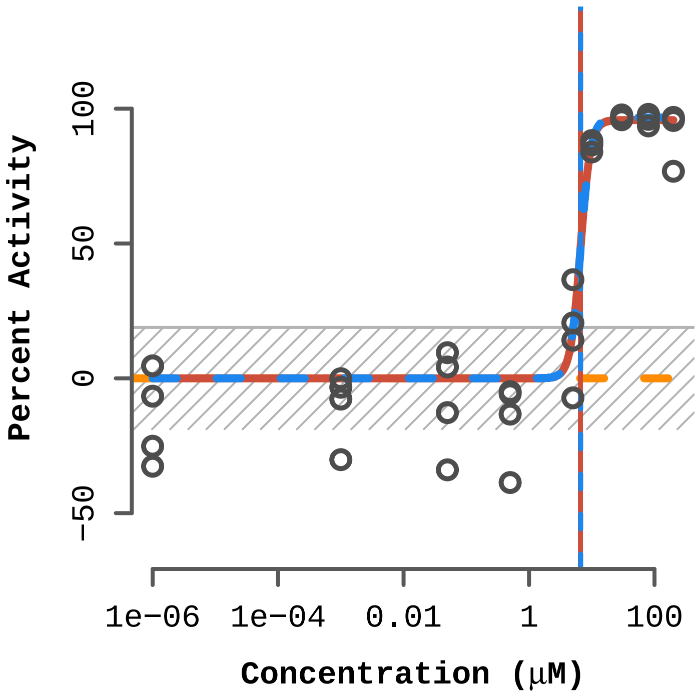

ASSAY: AEID3098 (CCTE_Deisenroth_DEVTOX_RUES2-GLR_Cel

NAME: Gefitinib
CHID: 41034 CASRN: 184475-35-2
SPID(S): ES211_34_34
M4ID: 42676241

HILL MODEL (in red):

	tp	ga	gw
val:	95.8	0.819	5.38
sd:	2.43	0.0278	0.988

GAIN-LOSS MODEL (in blue):

	tp	ga	gw	la	lw
val:	96.7	0.823	5.25	2.38	17.8
sd:	NaN	NaN	NaN	NaN	NaN

	CNST	HILL	GNLS
AIC:	407.33	294.51	298.09
PROB:	0	0.86	0.14
RMSE:	63.76	14.22	14.15

MAX_MEAN: 97.1 MAX_MED: 97.3 BMAD: 6.29

COFF: 18.9 HIT-CALL: 1 FITC: 41 ACTP: 1

FLAGS:

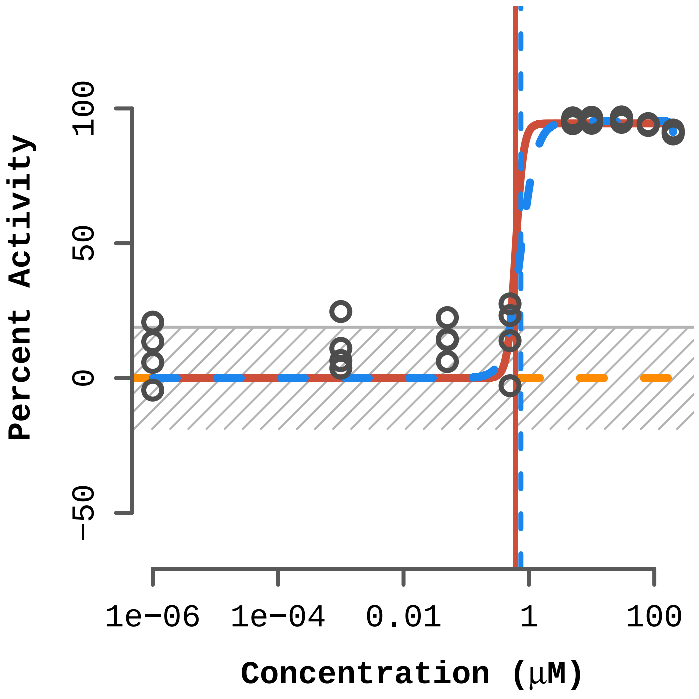

ASSAY: AEID3098 (CCTE_Deisenroth_DEVTOX_RUES2-GLR_Cel

NAME: Sorafenib
CHID: 41128 CASRN: 284461-73-0
SPID(S): ES211_57_57
M4ID: 42676233

HILL MODEL (in red):

	tp	ga	gw
val:	94.5	−0.214	6.86
sd:	1.23	1.97	155

GAIN-LOSS MODEL (in blue):

	tp	ga	gw	la	lw
val:	95.3	−0.128	3.45	2.39	16
sd:	NaN	NaN	NaN	NaN	NaN

	CNST	HILL	GNLS
AIC:	416.98	263.99	266.17
PROB:	0	0.75	0.25
RMSE:	71.16	9.23	9.16

MAX_MEAN: 95.7 MAX_MED: 95.8 BMAD: 6.29

COFF: 18.9 HIT-CALL: 1 FITC: 41 ACTP: 1

FLAGS:

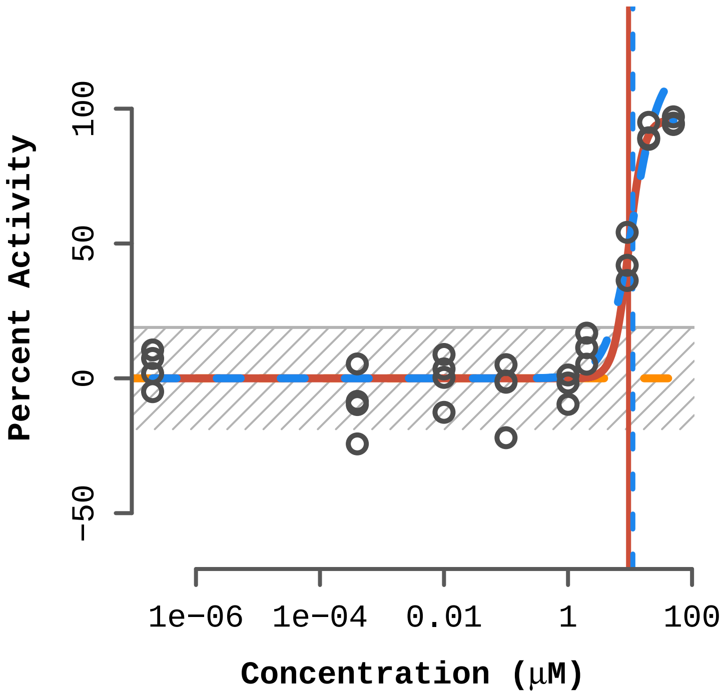

ASSAY: AEID3098 (CCTE_Deisenroth_DEVTOX_RUES2-GLR_Cel

NAME: Ritanserin
CHID: 42594 CASRN: 87051-43-2
SPID(S): ES211_53_53
M4ID: 42676237

HILL MODEL (in red):

	tp	ga	gw
val:	96.1	0.976	3.63
sd:	4.09	0.0265	1.39

GAIN-LOSS MODEL (in blue):

	tp	ga	gw	la	lw
val:	116	1.05	2.05	1.74	18
sd:	25.1	0.108	0.807	0.00788	10.7

	CNST	HILL	GNLS
AIC:	319.9	230.34	232.31
PROB:	0	0.73	0.27
RMSE:	44.13	8.9	8.65

MAX_MEAN: 95.3 MAX_MED: 94.7 BMAD: 6.29

COFF: 18.9 HIT-CALL: 1 FITC: 41 ACTP: 1

FLAGS:

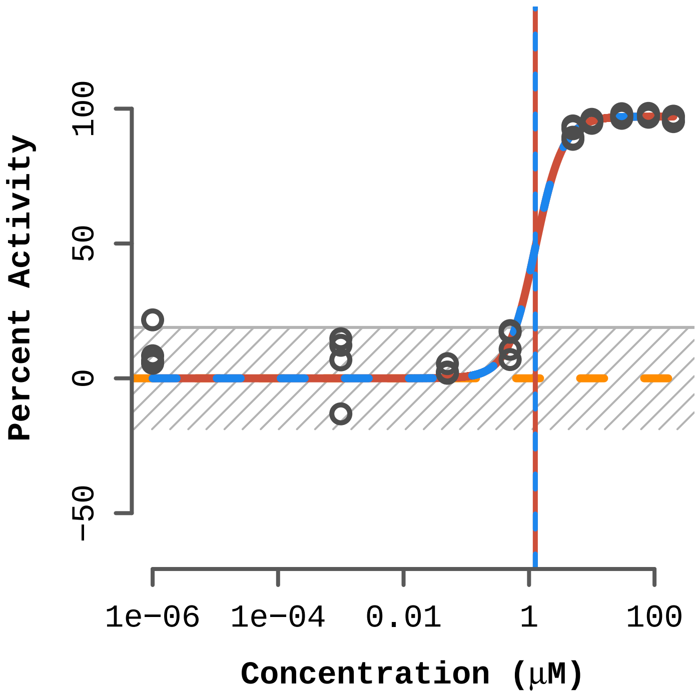

ASSAY: AEID3098 (CCTE_Deisenroth_DEVTOX_RUES2-GLR_Cel

NAME: Sunitinib malate
CHID: 46492 CASRN: 341031-54-7
SPID(S): ES211_60_60
M4ID: 42676215

HILL MODEL (in red):

	tp	ga	gw
val:	97.1	0.1	1.96
sd:	0.924	0.0433	0.18

GAIN-LOSS MODEL (in blue):

	tp	ga	gw	la	lw
val:	97.1	0.1	1.96	4.17	11.1
sd:	NA	NA	NA	NA	NA

	CNST	HILL	GNLS
AIC:	416.93	229.81	233.81
PROB:	0	0.88	0.12
RMSE:	71.6	6.13	6.13

MAX_MEAN: 97.6 MAX_MED: 97.7 BMAD: 6.29

COFF: 18.9 HIT-CALL: 1 FITC: 41 ACTP: 1

FLAGS:

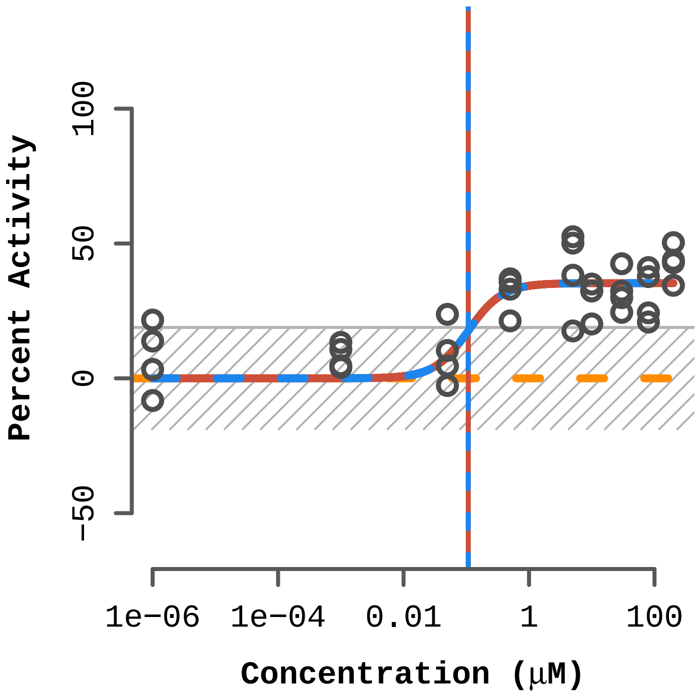

ASSAY: AEID3098 (CCTE_Deisenroth_DEVTOX_RUES2-GLR_Cel

NAME: Lenalidomide
CHID: 46664 CASRN: 191732-72-6
SPID(S): ES211_41_41
M4ID: 42676202

HILL MODEL (in red):

	tp	ga	gw
val:	35.3	-0.968	1.59
sd:	2.4	0.236	0.929

GAIN-LOSS MODEL (in blue):

	tp	ga	gw	la	lw
val:	35.3	-0.968	1.59	3.84	6.86
sd:	2.4	0.236	0.929	11700	52100

	CNST	HILL	GNLS
AIC:	355.18	279.9	283.9
PROB:	0	0.88	0.12
RMSE:	30.14	10.03	10.03

MAX_MEAN: 42.9 MAX_MED: 44.2 BMAD: 6.29

COFF: 18.9 HIT-CALL: 1 FITC: 41 ACTP: 1

FLAGS: 17

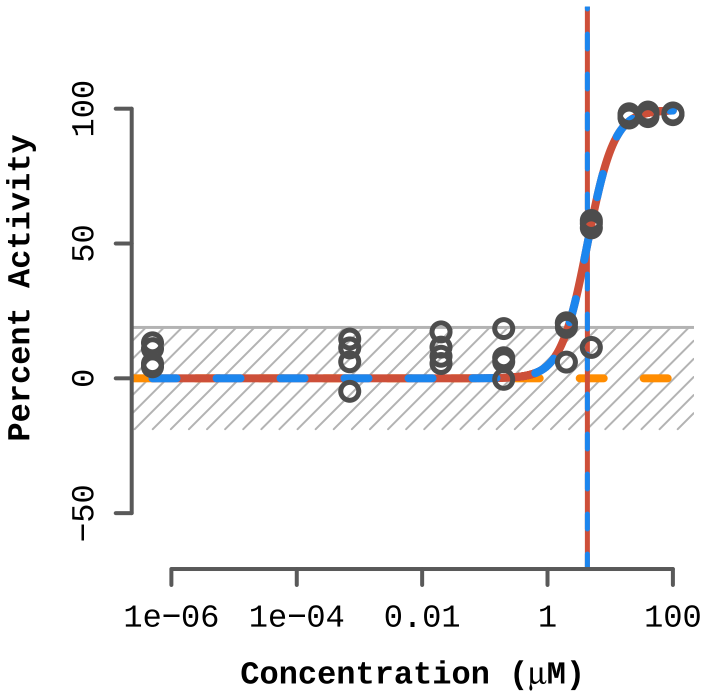

ASSAY: AEID3098 (CCTE_Deisenroth_DEVTOX_RUES2-GLR_Cel

NAME: Vandetanib
CHID: 46681 CASRN: 443913-73-3
SPID(S): ES211_64_64
M4ID: 42676212

HILL MODEL (in red):

	tp	ga	gw
val:	99.5	0.636	2.04
sd:	1.91	0.0271	0.271

GAIN-LOSS MODEL (in blue):

	tp	ga	gw	la	lw
val:	99.5	0.636	2.04	3.76	12.9
sd:	NA	NA	NA	NA	NA

	CNST	HILL	GNLS
AIC:	400.29	265.01	269.01
PROB:	0	0.88	0.12
RMSE:	59.58	10.49	10.49

MAX_MEAN: 98.1 MAX_MED: 98.3 BMAD: 6.29

COFF: 18.9 HIT-CALL: 1 FITC: 41 ACTP: 1

FLAGS:

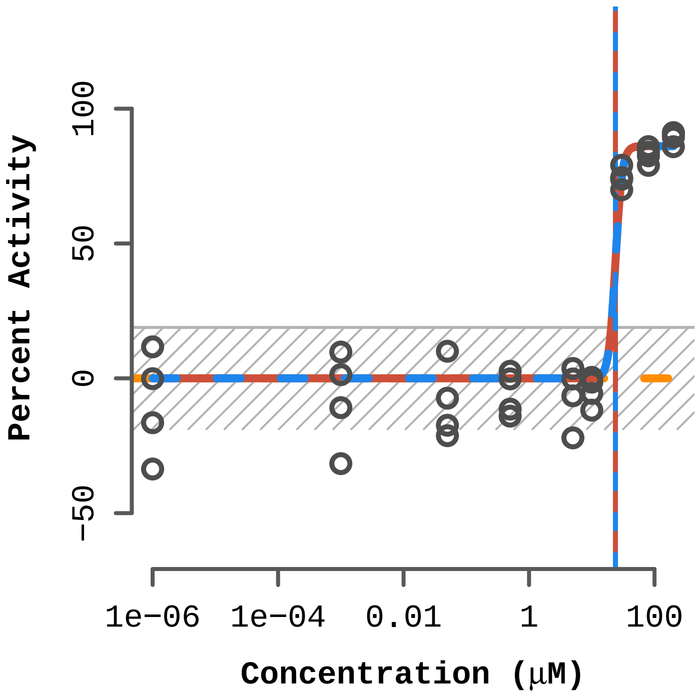

ASSAY: AEID3098 (CCTE_Deisenroth_DEVTOX_RUES2-GLR_Cel

NAME: 6-aminopyridine-3-carboxamide
CHID: 51446 CASRN: 329-89-5
SPID(S): ES211_7_7
M4ID: 42676203

HILL MODEL (in red):

	tp	ga	gw
val:	86.1	1.38	8
sd:	2.81	0.0859	6.72

GAIN-LOSS MODEL (in blue):

	tp	ga	gw	la	lw
val:	86.1	1.38	8	3.47	10.5
sd:	2.81	0.0859	6.72	27100	242000

	CNST	HILL	GNLS
AIC:	384.81	282.85	286.85
PROB:	0	0.88	0.12
RMSE:	48.93	11.64	11.64

MAX_MEAN: 89.2 MAX_MED: 89.9 BMAD: 6.29

COFF: 18.9 HIT-CALL: 1 FITC: 41 ACTP: 1

FLAGS:

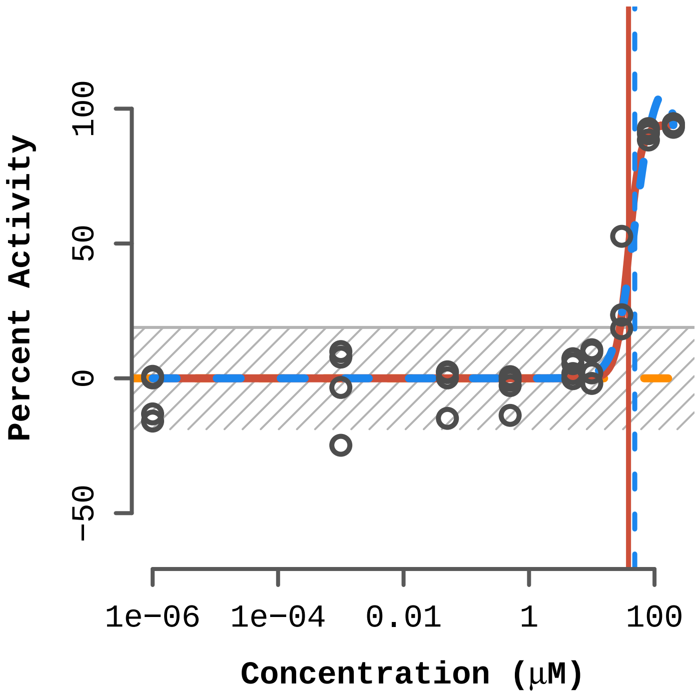

ASSAY: AEID3098 (CCTE_Deisenroth_DEVTOX_RUES2-GLR_Cel

NAME: Esomeprazole magnesium hydrate
CHID: 746660 CASRN: 668985-31-7
SPID(S): ES211_43_43
M4ID: 42676190

HILL MODEL (in red):

	tp	ga	gw
val:	94.2	1.59	4.43
sd:	2.43	0.0288	1.01

GAIN-LOSS MODEL (in blue):

	tp	ga	gw	la	lw
val:	113	1.69	2.76	2.38	9.86
sd:	35.9	0.152	1.25	0.01	10.1

	CNST	HILL	GNLS
AIC:	373.34	257.77	260.63
PROB:	0	0.81	0.19
RMSE:	45.47	8.86	8.75

MAX_MEAN: 93.9 MAX_MED: 94 BMAD: 6.29

COFF: 18.9 HIT-CALL: 1 FITC: 41 ACTP: 1

FLAGS:

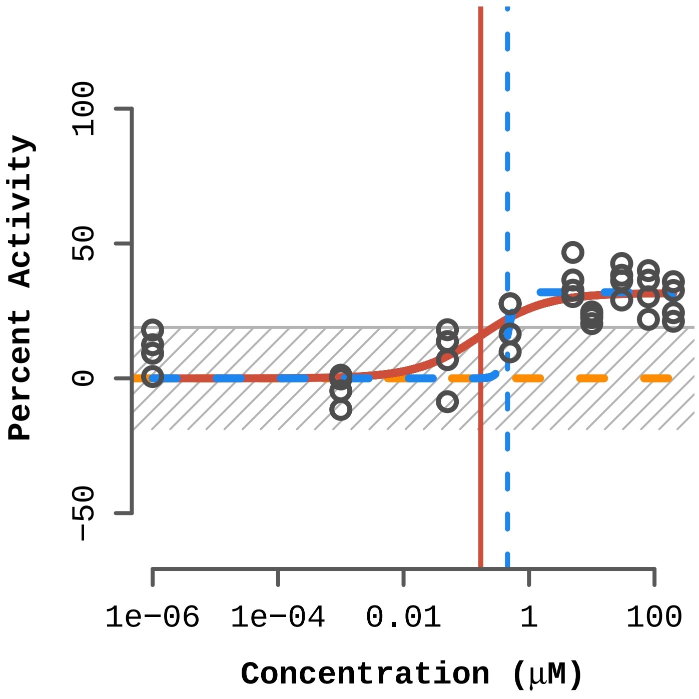

ASSAY: AEID3098 (CCTE_Deisenroth_DEVTOX_RUES2-GLR_Cel

NAME: Pomalidomide
CHID: 893458 CASRN: 19171-19-8
SPID(S): ES211_50_50
M4ID: 42676235

HILL MODEL (in red):

	tp	ga	gw
val:	31.6	-0.769	0.85
sd:	2.36	0.301	0.346

GAIN-LOSS MODEL (in blue):

	tp	ga	gw	la	lw
val:	31.9	-0.343	6.59	2.35	18
sd:	NaN	NaN	NaN	NaN	NaN

	CNST	HILL	GNLS
AIC:	343.99	267.49	273.73
PROB:	0	0.96	0.04
RMSE:	25.81	8.44	8.64

MAX_MEAN: 36.5 MAX_MED: 37.2 BMAD: 6.29

COFF: 18.9 HIT-CALL: 1 FITC: 41 ACTP: 1

FLAGS: 17

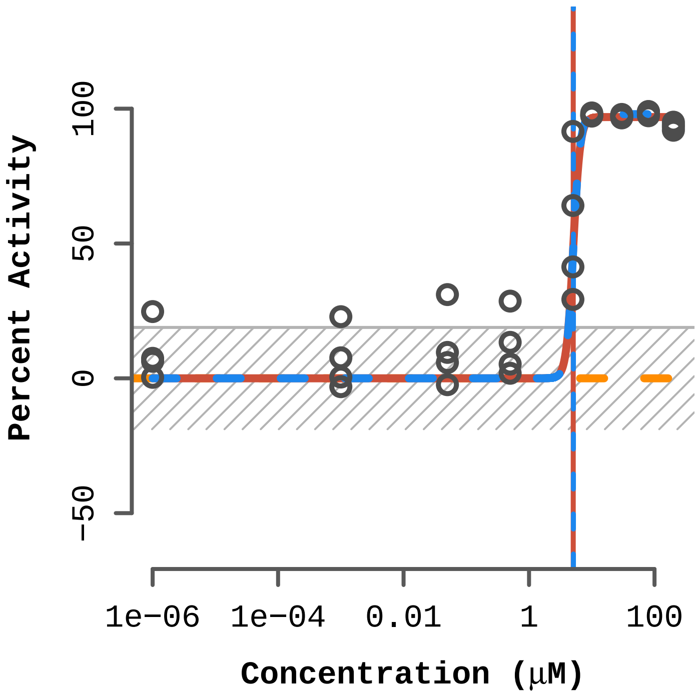

ASSAY: AEID3098 (CCTE_Deisenroth_DEVTOX_RUES2-GLR_Cel

NAME: Tegaserod maleate
CHID: 904761 CASRN: 189188-57-6
SPID(S): ES211_61_61
M4ID: 42676221

HILL MODEL (in red):

	tp	ga	gw
val:	96.9	0.704	8
sd:	1.75	0.0215	5.96

GAIN-LOSS MODEL (in blue):

	tp	ga	gw	la	lw
val:	98	0.707	8	2.44	9.81
sd:	NaN	NaN	NaN	NaN	NaN

	CNST	HILL	GNLS
AIC:	413.04	283.85	286.46
PROB:	0	0.79	0.21
RMSE:	68.38	13.12	13.14

MAX_MEAN: 98.5 MAX_MED: 98.8 BMAD: 6.29

COFF: 18.9 HIT-CALL: 1 FITC: 41 ACTP: 1

FLAGS:

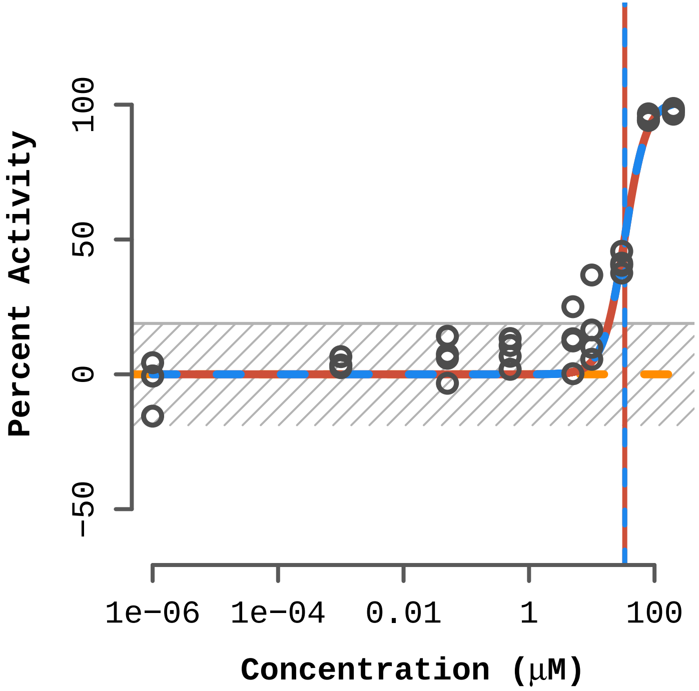

ASSAY: AEID3098 (CCTE_Deisenroth_DEVTOX_RUES2-GLR_Cel

NAME: Mianserin hydrochloride
CHID: 944145 CASRN: 21535-47-7
SPID(S): ES211_47_47
M4ID: 42676227

HILL MODEL (in red):

	tp	ga	gw
val:	101	1.53	2.5
sd:	4.06	0.0254	0.696

GAIN-LOSS MODEL (in blue):

	tp	ga	gw	la	lw
val:	101	1.53	2.5	3.32	13.2
sd:	NaN	NaN	NaN	NaN	NaN

	CNST	HILL	GNLS
AIC:	380.54	265.92	269.92
PROB:	0	0.88	0.12
RMSE:	48.52	9.43	9.43

MAX_MEAN: 97.6 MAX_MED: 97.7 BMAD: 6.29

COFF: 18.9 HIT-CALL: 1 FITC: 41 ACTP: 1

FLAGS:

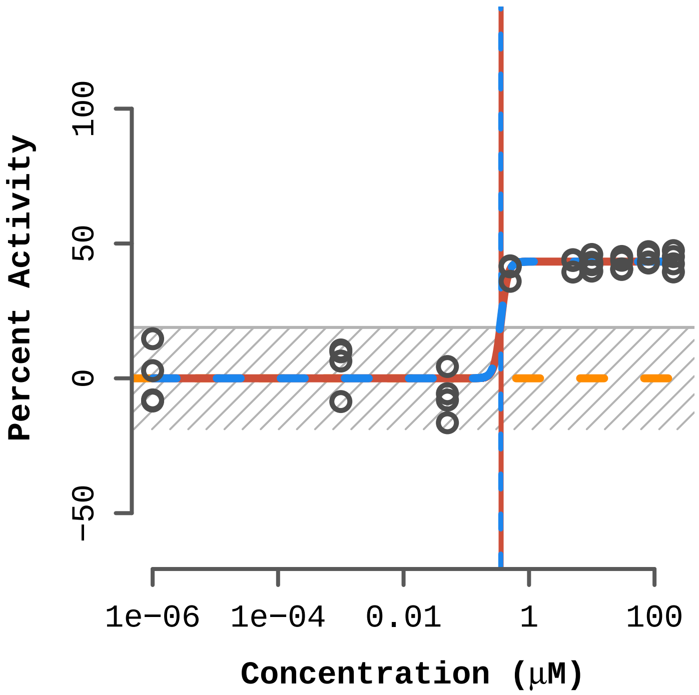

ASSAY: AEID3098 (CCTE_Deisenroth_DEVTOX_RUES2-GLR_Cel

NAME: Methotrexate hydrate(1:x)
CHID: 1017640 CASRN: 133073-73-1
SPID(S): ES211_45_45
M4ID: 42676185

HILL MODEL (in red):

	tp	ga	gw
val:	43.3	-0.445	7.93
sd:	0.949	3.63	200

GAIN-LOSS MODEL (in blue):

	tp	ga	gw	la	lw
val:	43.3	-0.451	7.64	2.86	13.1
sd:	0.949	3.64	186	210	4870

	CNST	HILL	GNLS
AIC:	367.68	234.33	238.33
PROB:	0	0.88	0.12
RMSE:	35.4	5.79	5.79

MAX_MEAN: 44.7 MAX_MED: 44.6 BMAD: 6.29

COFF: 18.9 HIT-CALL: 1 FITC: 41 ACTP: 1

FLAGS: 17

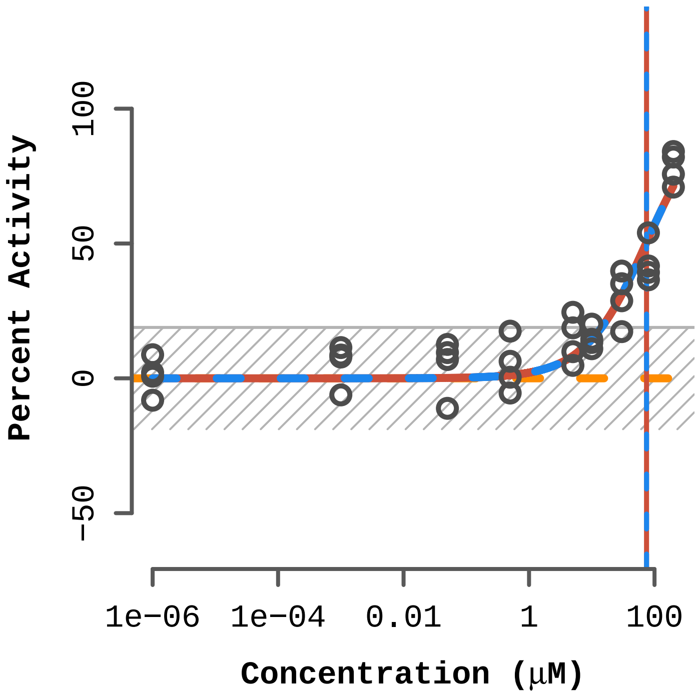

ASSAY: AEID3098 (CCTE_Deisenroth_DEVTOX_RUES2-GLR_Cel

NAME: Diphenhydramine hydrochloride
CHID: 20537 CASRN: 147-24-0
SPID(S): ES211_31_31
M4ID: 42676209

HILL MODEL (in red):

	tp	ga	gw
val:	101	1.87	0.891
sd:	18.2	0.201	0.15

GAIN-LOSS MODEL (in blue):

	tp	ga	gw	la	lw
val:	101	1.87	0.891	3.74	6.17
sd:	18.2	0.201	0.15	1220	5210

	CNST	HILL	GNLS
AIC:	352.61	270.72	274.72
PROB:	0	0.88	0.12
RMSE:	33.04	8.72	8.72

MAX_MEAN: 78.2 MAX_MED: 78.9 BMAD: 6.29

COFF: 18.9 HIT-CALL: 1 FITC: 42 ACTP: 1

FLAGS:

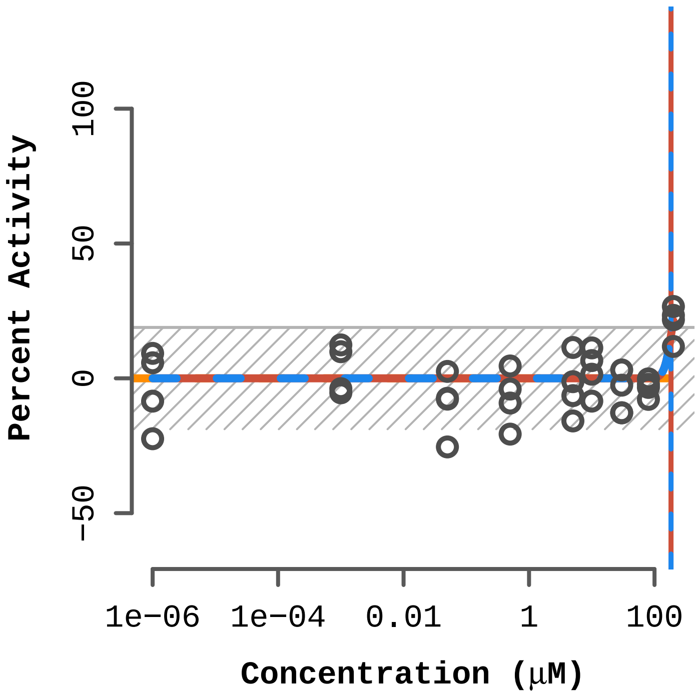

ASSAY: AEID3098 (CCTE_Deisenroth_DEVTOX_RUES2-GLR_Cel

NAME: 5,5−Diphenylhydantoin
CHID: 20541 CASRN: 57−41−0
SPID(S): ES211_3_3
M4ID: 42676211

HILL MODEL (in red):

	tp	ga	gw
val:	32	2.26	8
sd:	71.6	0.315	15.5

GAIN−LOSS MODEL (in blue):

	tp	ga	gw	la	lw
val:	32	2.26	8	4.14	18
sd:	NA	NA	NA	NA	NA

	CNST	HILL	GNLS
AIC:	285.61	276.36	280.36
PROB:	0.01	0.87	0.12
RMSE:	12.09	9.88	9.88

MAX_MEAN: 20.9 MAX_MED: 22.6 BMAD: 6.29

COFF: 18.9 HIT−CALL: 1 FITC: 42 ACTP: 0.99

FLAGS: 7; 11; 17

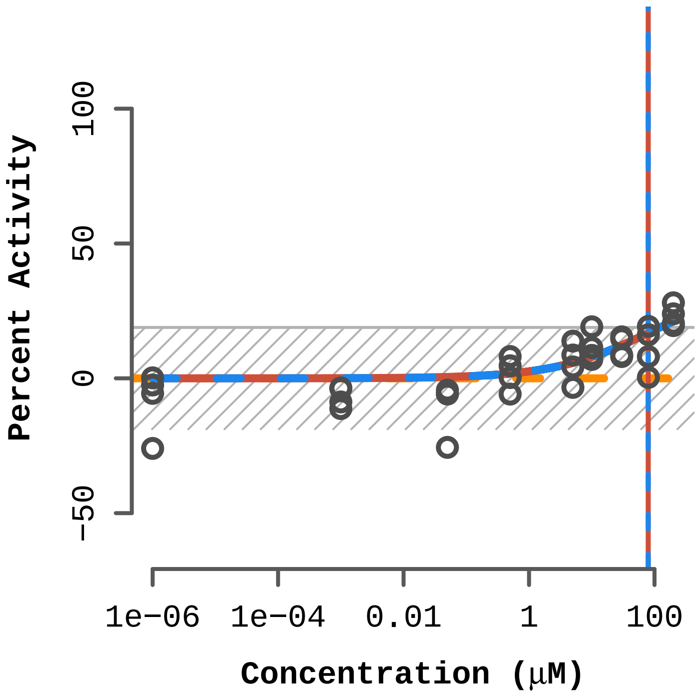

ASSAY: AEID3098 (CCTE_Deisenroth_DEVTOX_RUES2-GLR_Cel[illegible]

NAME: Ibuprofen
CHID: 20732 CASRN: 15687-27-1
SPID(S): ES211_36_36
M4ID: 42676187

HILL MODEL (in red):

	tp	ga	gw
val:	33.6	1.9	0.569
sd:	26	1.25	0.26

GAIN-LOSS MODEL (in blue):

	tp	ga	gw	la	lw
val:	33.6	1.9	0.569	3.64	6.21
sd:	26	1.25	0.26	1470	6840

	CNST	HILL	GNLS
AIC:	291.97	257.95	261.95
PROB:	0	0.88	0.12
RMSE:	13.09	8.49	8.49

MAX_MEAN: 23 MAX_MED: 22.2 BMAD: 6.29

COFF: 18.9 HIT-CALL: 1 FITC: 42 ACTP: 1

FLAGS: 7; 11; 17

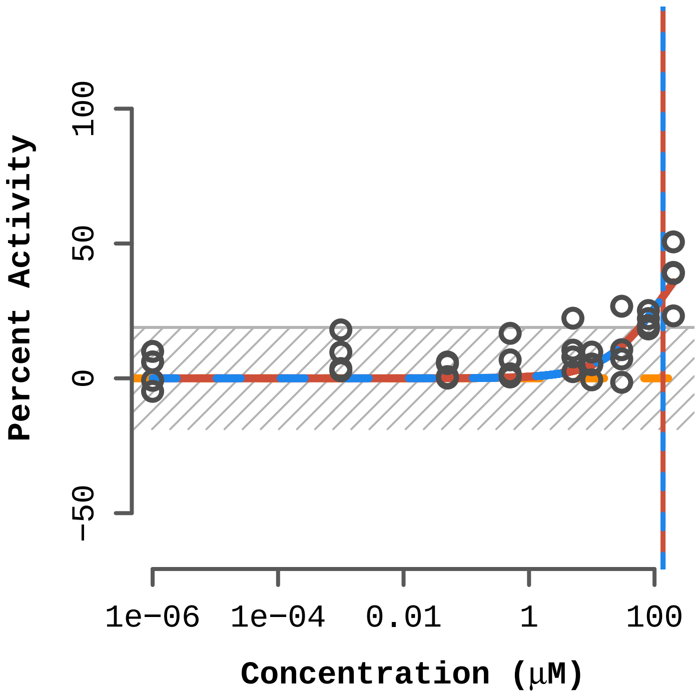

ASSAY: AEID3098 (CCTE_Deisenroth_DEVTOX_RUES2-GLR_Cel

NAME: Carbamazepine
CHID: 22731 CASRN: 298−46−4
SPID(S): ES211_21_21
M4ID: 42676226

HILL MODEL (in red):

	tp	ga	gw
val:	60.7	2.14	0.924
sd:	23.4	0.361	0.325

GAIN−LOSS MODEL (in blue):

	tp	ga	gw	la	lw
val:	60.7	2.14	0.927	3.25	18
sd:	23.4	0.36	0.328	0.00201	2030000

	CNST	HILL	GNLS
AIC:	306.42	260.61	264.61
PROB:	0	0.88	0.12
RMSE:	17.24	8.11	8.11

MAX_MEAN: 38 MAX_MED: 39.1 BMAD: 6.29

COFF: 18.9 HIT−CALL: 1 FITC: 42 ACTP: 1

FLAGS: 17

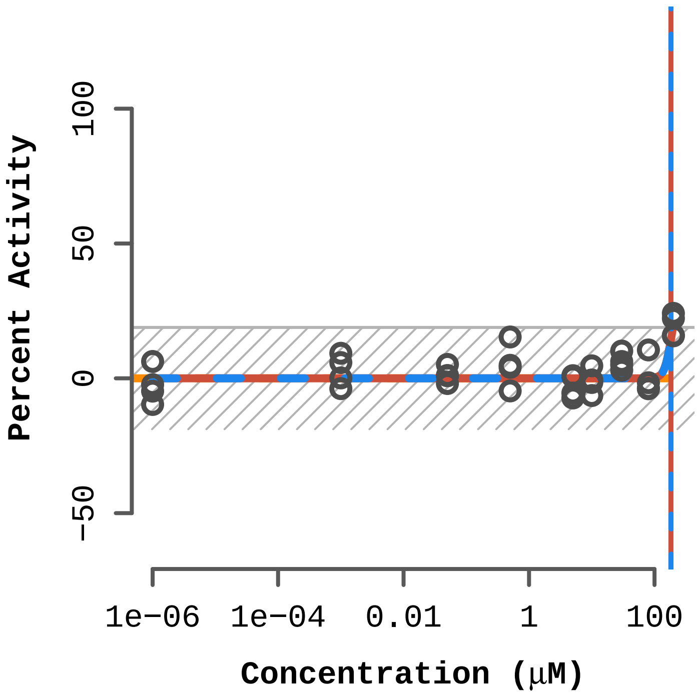

ASSAY: AEID3098 (CCTE_Deisenroth_DEVTOX_RUES2-GLR_Cel

NAME: Warfarin
CHID: 23742 CASRN: 81−81−2
SPID(S): ES211_65_65
M4ID: 42676231

HILL MODEL (in red):

	tp	ga	gw
val:	28.9	2.26	8
sd:	NaN	NaN	NaN

GAIN−LOSS MODEL (in blue):

	tp	ga	gw	la	lw
val:	28.9	2.26	8	2.81	7.78
sd:	NaN	NaN	NaN	NaN	NaN

	CNST	HILL	GNLS
AIC:	256.96	237.03	241.03
PROB:	0	0.88	0.12
RMSE:	8.62	5.65	5.65

MAX_MEAN: 19.5 MAX_MED: 19.1 BMAD: 6.29

COFF: 18.9 HIT−CALL: 1 FITC: 42 ACTP: 1

FLAGS: 7; 11; 17

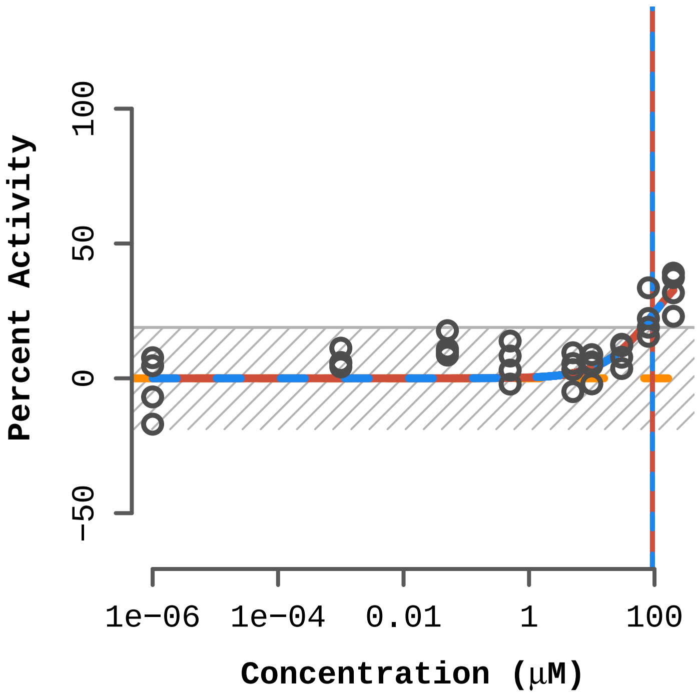

ASSAY: AEID3098 (CCTE_Deisenroth_DEVTOX_RUES2-GLR_Cel

NAME: Hydroxyurea
CHID: 25438 CASRN: 127-07-1
SPID(S): ES211_35_35
M4ID: 42676204

HILL MODEL (in red):

	tp	ga	gw
val:	46.8	1.97	1.09
sd:	20.3	0.399	0.476

GAIN-LOSS MODEL (in blue):

	tp	ga	gw	la	lw
val:	46.8	1.97	1.09	3.77	10.3
sd:	NaN	NaN	NaN	NaN	NaN

	CNST	HILL	GNLS
AIC:	301.96	259.18	263.18
PROB:	0	0.88	0.12
RMSE:	15.68	7.6	7.6

MAX_MEAN: 32.8 MAX_MED: 34.6 BMAD: 6.29

COFF: 18.9 HIT-CALL: 1 FITC: 42 ACTP: 1

FLAGS: 17

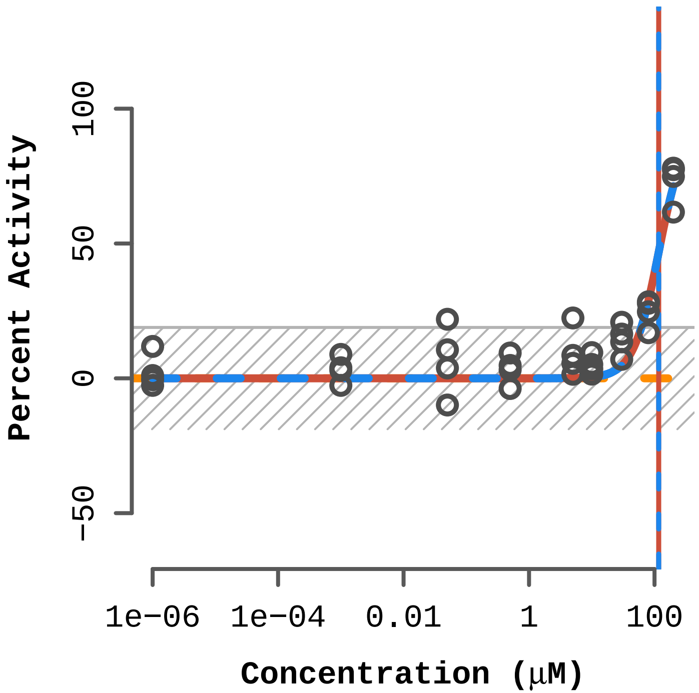

ASSAY: AEID3098 (CCTE_Deisenroth_DEVTOX_RUES2-GLR_Cel

NAME: MEHP
CHID: 25680 CASRN: 4376-20-9
SPID(S): ES211_44_44
M4ID: 42676200

HILL MODEL (in red):

	tp	ga	gw
val:	93.6	2.07	2.15
sd:	19.9	0.102	0.859

GAIN-LOSS MODEL (in blue):

	tp	ga	gw	la	lw
val:	93.6	2.07	2.15	4.3	6.49
sd:	20	0.102	0.862	161000	509000

	CNST	HILL	GNLS
AIC:	329.82	265.02	269.02
PROB:	0	0.88	0.12
RMSE:	27.25	8.56	8.56

MAX_MEAN: 73 MAX_MED: 76.2 BMAD: 6.29

COFF: 18.9 HIT-CALL: 1 FITC: 42 ACTP: 1

FLAGS:

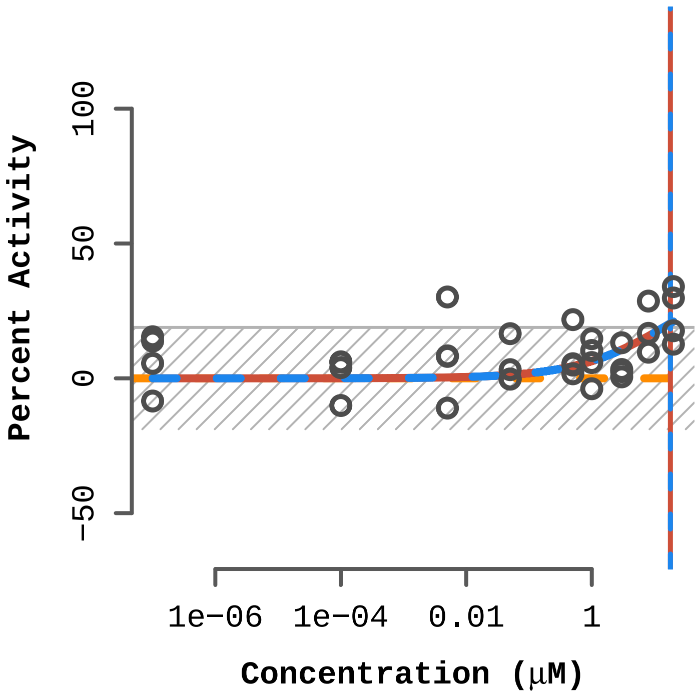

ASSAY: AEID3098 (CCTE_Deisenroth_DEVTOX_RUES2-GLR_Cel

NAME: Dexamethasone sodium phosphate
CHID: 47429 CASRN: 2392−39−4
SPID(S): ES211_28_28
M4ID: 42676228

HILL MODEL (in red):

	tp	ga	gw
val:	40.8	1.25	0.584
sd:	31	1.09	0.384

GAIN−LOSS MODEL (in blue):

	tp	ga	gw	la	lw
val:	40.8	1.25	0.584	2.32	7.45
sd:	31	1.09	0.384	816	5950

	CNST	HILL	GNLS
AIC:	287.46	268.16	272.16
PROB:	0	0.88	0.12
RMSE:	14.13	9.81	9.81

MAX_MEAN: 23.5 MAX_MED: 23.7 BMAD: 6.29

COFF: 18.9 HIT−CALL: 1 FITC: 42 ACTP: 1

FLAGS: 7; 17

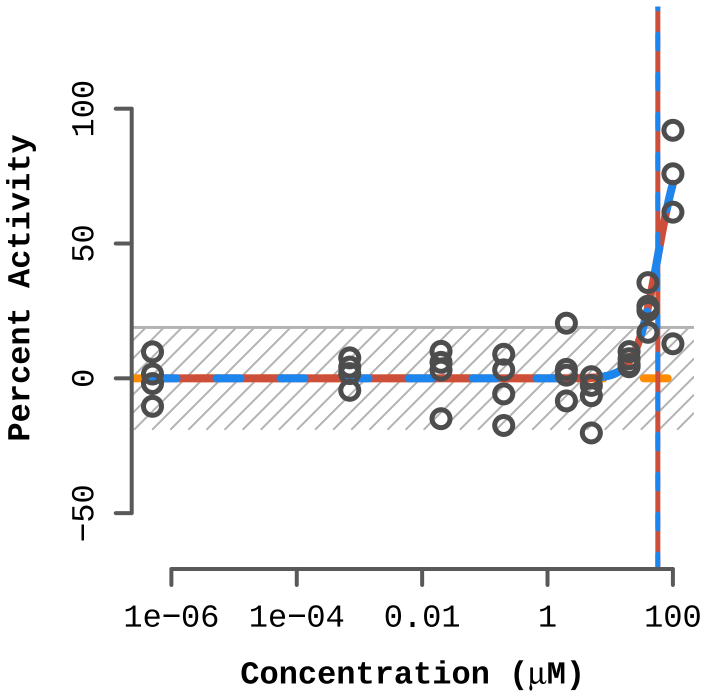

ASSAY: AEID3098 (CCTE_Deisenroth_DEVTOX_RUES2-GLR_Cel

NAME: Ketanserin tartrate
CHID: 1003945 CASRN: 83846-83-7
SPID(S): ES211_40_40
M4ID: 42676230

HILL MODEL (in red):

	tp	ga	gw
val:	90.5	1.76	2.48
sd:	29.2	0.159	0.988

GAIN-LOSS MODEL (in blue):

	tp	ga	gw	la	lw
val:	90.5	1.76	2.48	2.92	6.08
sd:	29.3	0.159	0.988	471	3140

	CNST	HILL	GNLS
AIC:	320.52	280.78	284.78
PROB:	0	0.88	0.12
RMSE:	25.47	13.24	13.24

MAX_MEAN: 60.6 MAX_MED: 68.7 BMAD: 6.29

COFF: 18.9 HIT-CALL: 1 FITC: 42 ACTP: 1

FLAGS:

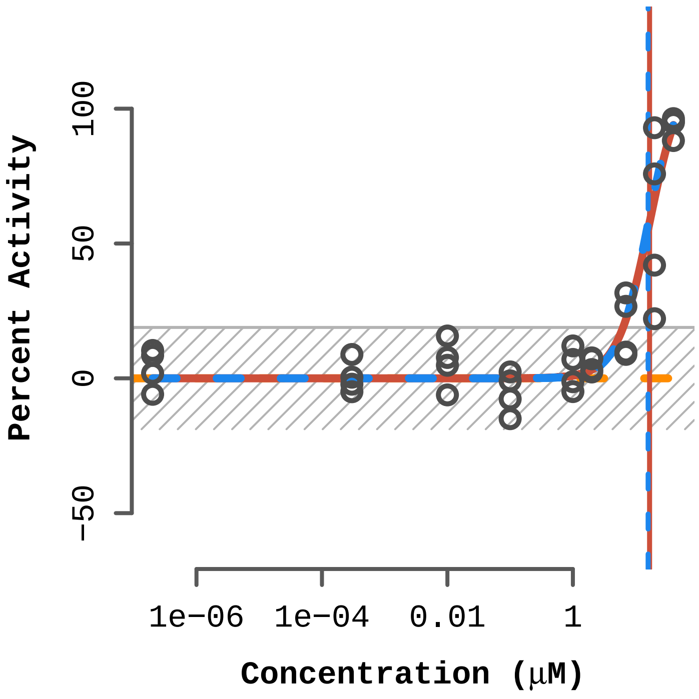

ASSAY: AEID3098 (CCTE_Deisenroth_DEVTOX_RUES2-GLR_Cel

NAME: Dorsomorphin
CHID: 1006988 CASRN: 866405-64-3
SPID(S): ES211_6_6
M4ID: 42676189

HILL MODEL (in red):

	tp	ga	gw
val:	116	1.22	1.68
sd:	50.6	0.377	1.03

GAIN-LOSS MODEL (in blue):

	tp	ga	gw	la	lw
val:	115	1.2	1.79	1.81	7.4
sd:	75.3	0.329	0.813	0.999	30.6

	CNST	HILL	GNLS
AIC:	356.44	280.32	284.3
PROB:	0	0.88	0.12
RMSE:	39.1	11.85	12.11

MAX_MEAN: 93.7 MAX_MED: 95.1 BMAD: 6.29

COFF: 18.9 HIT-CALL: 1 FITC: 42 ACTP: 1

FLAGS:

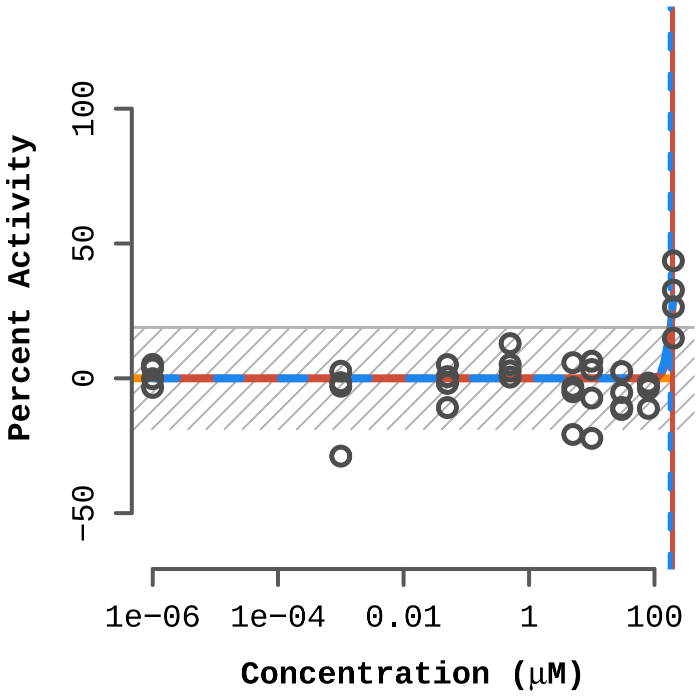

ASSAY: AEID3098 (CCTE_Deisenroth_DEVTOX_RUES2-GLR_Cel

NAME: (2R,3S)-2-(3,4-Dihydroxyphenyl)-3,4-dihydro-2H-
CHID: 1017388 CASRN: 225937-10-0
SPID(S): ES211_22_22
M4ID: 42676181

HILL MODEL (in red):

	tp	ga	gw
val:	52.3	2.29	8
sd:	37.6	0.0849	8.75

GAIN-LOSS MODEL (in blue):

	tp	ga	gw	la	lw
val:	41.1	2.25	8	4.29	16.3
sd:	NA	NA	NA	NA	NA

	CNST	HILL	GNLS
AIC:	285.31	268.41	272.43
PROB:	0	0.88	0.12
RMSE:	13.62	9.46	9.46

MAX_MEAN: 29.4 MAX_MED: 29.6 BMAD: 6.29

COFF: 18.9 HIT-CALL: 1 FITC: 42 ACTP: 1

FLAGS: 7; 17

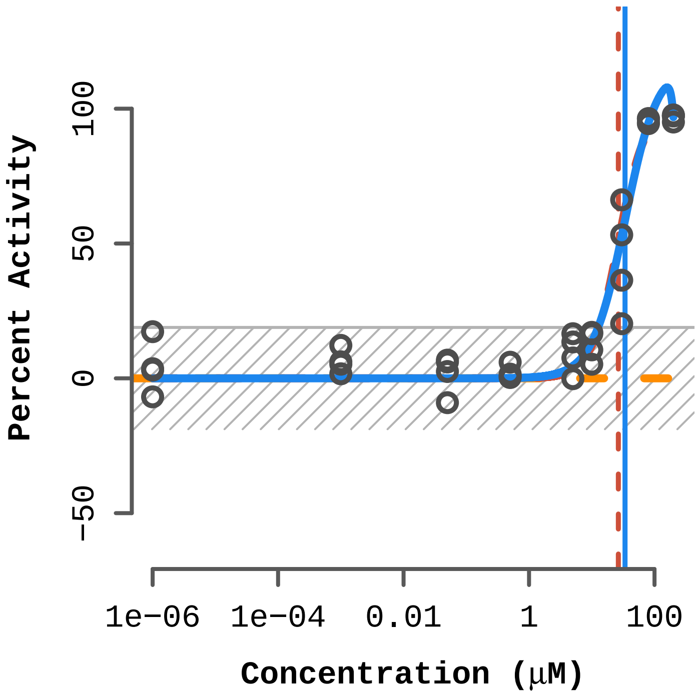

ASSAY: AEID3098 (CCTE_Deisenroth_DEVTOX_RUES2-GLR_Cel

NAME: Retinol
CHID: 23556 CASRN: 68−26−8
SPID(S): ES211_51_51
M4ID: 42676178

HILL MODEL (in red):

	tp	ga	gw
val:	101	1.42	2.05
sd:	3.27	0.0453	0.286

GAIN−LOSS MODEL (in blue):

	tp	ga	gw	la	lw
val:	117	1.53	1.67	2.37	11.7
sd:	14	0.097	0.29	0.0104	4.21

	CNST	HILL	GNLS
AIC:	379.94	262.48	261
PROB:	0	0.32	0.68
RMSE:	48.58	9.37	8.59

MAX_MEAN: 96.9 MAX_MED: 97.3 BMAD: 6.29

COFF: 18.9 HIT−CALL: 1 FITC: 50 ACTP: 1

FLAGS: 19

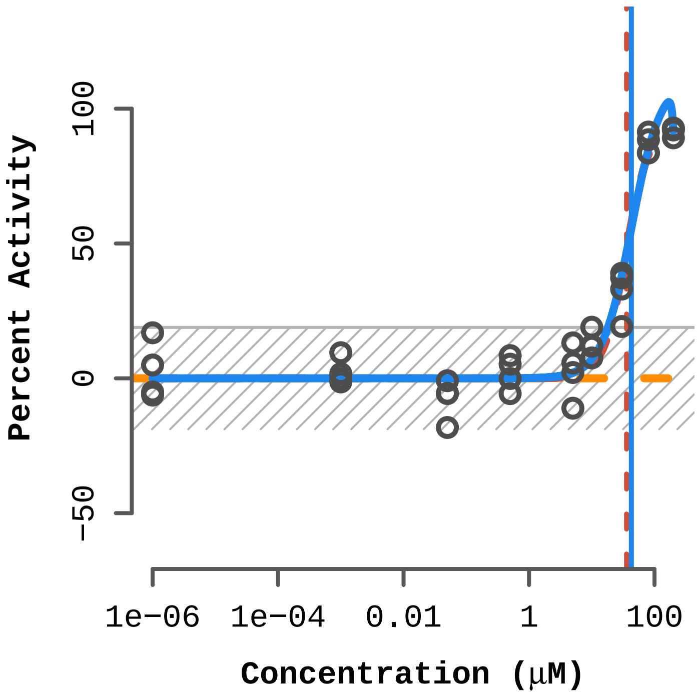


```
ASSAY:    AEID3098 (CCTE_Deisenroth_DEVTOX_RUES2-GLR_Cel

NAME:     Rifampicin
CHID:     21244    CASRN: 13292-46-1
SPID(S):  ES211_52_52
M4ID:     42676236

HILL MODEL (in red):
      tp        ga        gw
val:  95.4      1.55      2.36
sd:   3.93      0.0295    0.581

GAIN-LOSS MODEL (in blue):
      tp        ga        gw        la        lw
val:  111       1.63      1.85      2.35      17.5
sd:   14.6      0.0771    0.358     0.00657   6.56

      CNST        HILL        GNLS
AIC:  372.07      257.11      255.65
PROB: 0           0.33        0.67
RMSE: 44.16       7.72        7.35

MAX_MEAN: 91.7      MAX_MED: 92.4      BMAD: 6.29

COFF: 18.9   HIT-CALL: 1    FITC: 51   ACTP: 1

FLAGS: 19
```
