# Supplementary material for: The DevTox Germ Layer Reporter Platform: An Assay Adaptation of the Human Pluripotent Stem Cell Test
Source: Toxics. 2022 Jul 13;10(7):392. doi: 10.3390/toxics10070392 (PMC9321663; doi:10.3390/toxics10070392)
Supplement: Supplementary file 1 [file toxics-10-00392-s001.zip › Gamble_DevTox GLR_Sup Fig_v1.pdf]

SUPPLEMENTARY FIGURES

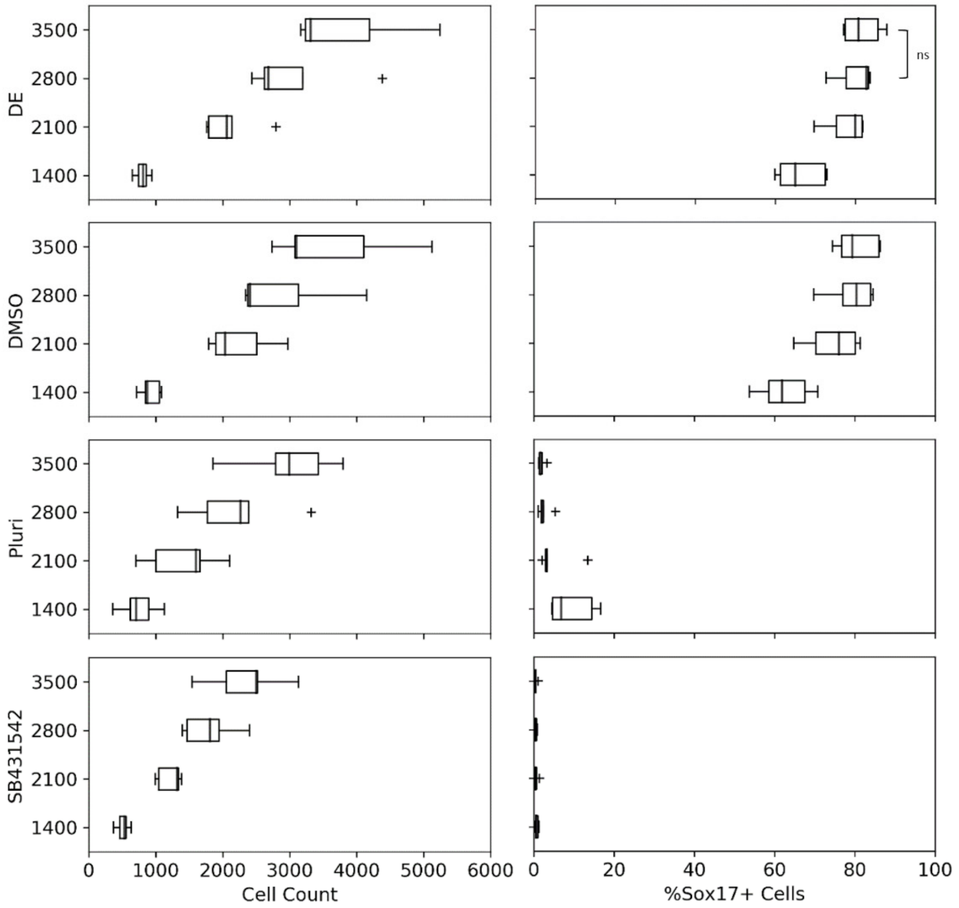

Figure S1: RUES2-GLR cell seeding effects on directed endoderm differentiation. Pluripotent RUES2-GLR cells were seeded at four seeding densities (1,400; 2,100; 2,800; 3,500 cells per well) in 384-well microplates were exposed to one of four conditions: directed endoderm (DE), DE with 0.1% DMSO (DMSO), pluripotent (Pluri), and DE with 1 µM TGF-ß receptor inhibitor (SB431542). Cells were counted and analyzed by high-content imaging with the left column graphs displaying average cells counted per well and the right column graphs displaying the average percentage of SOX17 positive cells per well. Box whisker plots; center line – median, box – first and third quartiles, whiskers – range, + outliers , ns – not significant; (*n* = 5).

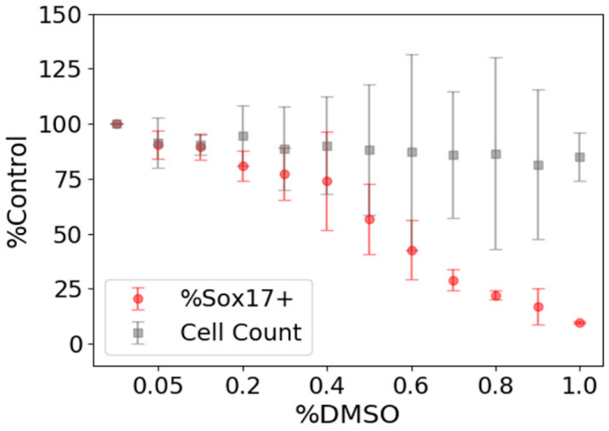

Figure S2: DevTox GLR-Endo DMSO tolerance test of RUES2-GLR. Graph depicting high-content image results after a 48 hour exposure to various concentration of DMSO during definitive endoderm differentiation. Gray points display average cells counted per well and red points display the average percentage of SOX17 positive cells per well. The 0.05% and 0.1% DMSO conditions were not significantly different from baseline control (0% DMSO) for percent SOX17 positive cells. No significant differences were identified between cell counts; Bars – 2*bmad. (*n* = 3).

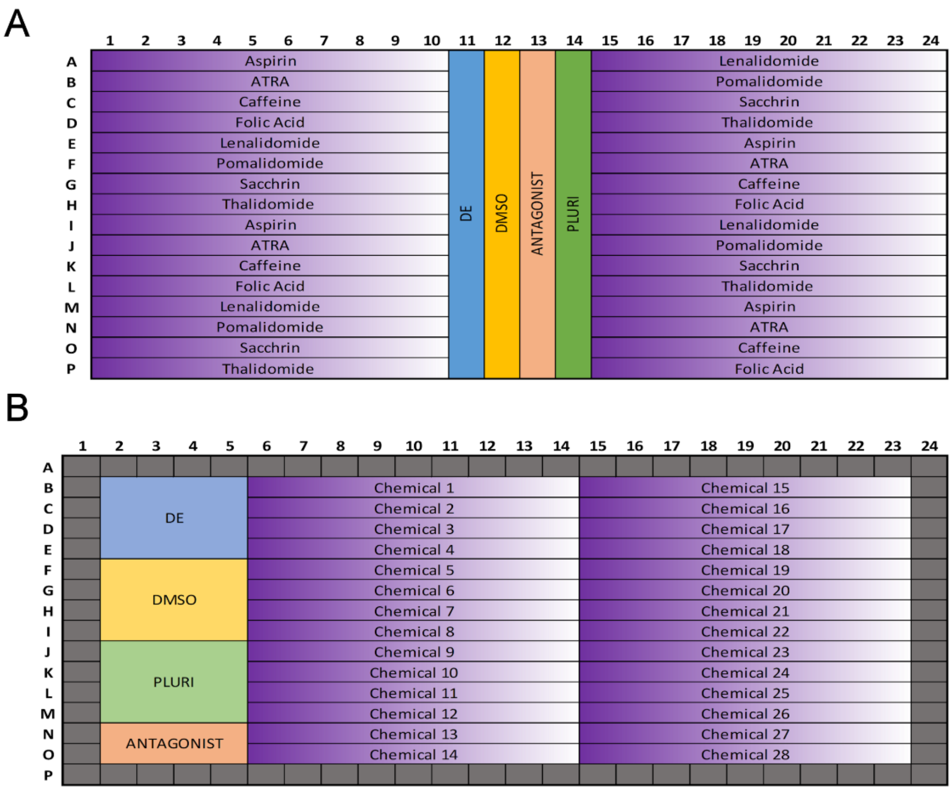

Figure S3: Plate maps. 384-well plate maps for (A) reference and (B) training chemical sets. DE – Directed endoderm control, DMSO – solvent control, ANTAGONIST – antagonist control (SB 431542), PLURI – pluripotent control. Gradient color for chemicals represents chemical exposure concentration from higher concentration (purple) to lower concentration (white). Gray border wells were unused but filled with sterile DPBS.
