# Supplementary material for: The DevTox Germ Layer Reporter Platform: An Assay Adaptation of the Human Pluripotent Stem Cell Test
Source: Toxics. 2022 Jul 13;10(7):392. doi: 10.3390/toxics10070392 (PMC9321663; doi:10.3390/toxics10070392)
Supplement: Supplementary file 1 [file toxics-10-00392-s001.zip › README_Gamble_DevTox GLR_Sup Data_v1.pdf]

GENERAL INFORMATION

1. Supplementary Data

The DevTox Germ Layer Reporter Platform: An Assay Adaptation of the Human Pluripotent Stem Cell Test (Manuscript)

Journal: Toxics

2. Author Information

   a. Principal Investigator Contact Information

Chad Deisenroth, PhD
Advanced Experimental Toxicology Models Branch
Biomolecular and Computational Toxicology Division
Center for Computational Toxicology and Exposure
U.S. Environmental Protection Agency
Research Triangle Park, NC 27711
deisenroth.chad@epa.gov
Phone: 919-541-3927

Supplementary Data File List

1. Gamble_DevTox GLR_Sup Fig_v1.doc
   a. **Figure S1: RUES2-GLR cell seeding effects on directed endoderm differentiation. Pluripotent** RUES2-GLR cells were seeded at four seeding densities (1,400; 2,100; 2,800; 3,500 cells per well) in 384-well microplates were exposed to one of four conditions: directed endoderm (DE), DE with 0.1% DMSO (DMSO), pluripotent (Pluri), and DE with 1 µM TGF-ß receptor inhibitor (SB431542). Cells were counted and analyzed by high-content imaging with the left column graphs displaying average cells counted per well and the right column graphs displaying the average percentage of SOX17 positive cells per well. Box whisker plots; center line – median, box – first and third quartiles, whiskers – range, + outliers , ns – not significant; (*n* = 5).
   b. **Figure S2: DevTox GLR-Endo DMSO tolerance test of RUES2-GLR.** Graph depicting high-content image results after a 48 hour exposure to various concentration of DMSO during definitive endoderm differentiation. Gray points display average cells counted per well and red points display the average percentage of SOX17 positive cells per well. The 0.05% and 0.1% DMSO conditions were not significantly different from baseline control (0% DMSO) for percent SOX17 positive cells. No significant differences were identified between cell counts; Bars – 2*bmad. (*n* = 3).
   c. **Figure S3: Plate maps.** 384-well plate maps for (A) reference and (B) training chemical sets. DE – Directed endoderm control, DMSO – solvent control, ANTAGONIST – antagonist control (SB 431542), PLURI – pluripotent control. Gradient color for chemicals represents chemical exposure concentration from higher concentration (purple) to lower concentration (white). Gray border wells were unused but filled with sterile DPBS.
2. Gamble_DevTox GLR_Tables_v1.xlsx

a. **Table 1. Reference chemicals**. Chemical name, CAS registry number (CASRN), DSSTox substance identifier (DTXSID), developmental toxicant classification, FDA pregnancy category, 50% inhibitory concentration (IC50) and percentage maximum inhibition (% max inhibition).
b. **Table 2. DevTox RUES2-GLR Endo Assay Endpoints**. Assay endpoint IDs (AEID); Assay endpoint name; interpretation: positive – increase in biomarker or cell count, negative – reduction in bi-omarker or cell count.
c. **Table 3. Comparison of chemical responses between the DevTox GLR-Endo and hPST assays.** Comparisons were drawn based on reported hPST assay analysis. Developmental toxicant clas-sification (DT Class.), positive (Pos), negative (Neg); cytotoxicity half-maximal concentration (TC50); cell count half-maximal concentration (CC50); SOX17 half-maximal activation/inhibition concentration (AC50/IC50); hit calls (TP—true positive; FP—false positive; TN—true negative; FN—false negative). Sensitivity, specificity, and balanced accuracy are noted.
d. **Table 4. Comparison of chemical responses between the DevTox GLR-Endo and devTOX quick-Predict assays**. Comparisons were drawn based on reported quickPredict assay analysis. Developmental toxicant classification (DT class.), positive (Pos), negative (Neg); CV-concentration at cell viability cutoff; concentration at cell count cutoff (CC); Teratogen Index (TI); activity concentration at cutoff (ACC); hit calls (TP—true positive; FP—false positive; TN—true negative; FN—false negative). Sensitivity, specificity, and balanced accuracy are noted. * Inferred inactivity from a single-concentration screen.

3. Gamble_DevTox GLR_Sup Tables_v1.xlsx
   a. **Tab1: Table S1 – Chemical List.** 67 control, reference and training chemicals used in evaluating the DevTox GLR-Endo assay with chemical identifications (CASRN, Chemical Name, DTXSID), supplier information (Supplier, Catalog Number, Lot number, % Purity, Molecular Weight) and testing conditions (Solvent, Stock Concentration, Highest Tested Concentration, Developmental Toxicant Classification, Category, Reference).
   b. **Tab 2: Table S2 – Chemical reference training set screen results.** Quantitative data for Figure 5 rank order plot. Hit calls are based on changes to the SOX17 population (DevTox). Activity concentration at cutoff (ACC) values and percent max inhibition for DevTox and cell count.
   c. **Tab 3: RUES2-GLR Z-test scores.** Average Z-Test scores generated from reference Scorecard data from Tsankov A, et al. 2015 (DOI: 10.1038/nbt.3387) for 10 human pluripotent stem cell lines and RUES2-GLR biological replicates at both directed endoderm and pluripotent states.
   d. **Tab 4: RUES2-GLR gene expression**. Average Ct values generated from reference Scorecard data from Tsankov A, et al. 2015 (DOI: 10.1038/nbt.3387) for 10 human pluripotent stem cell lines and RUES2-GLR biological replicates at both directed endoderm and pluripotent states.
   e. Tab 5: DevTox GLR-Endo seeding density. High content imaging data for RUES2-GLR seeding density testing with DevTox GLR-Endo assay. Treatment: PluriControl – Pluripotent; DMSOControl – 0.2% DMSO exposure with definite endoderm induction; 1 µM SB431542 exposure with definitive endoderm induction; DEControl – definitive endoderm induction. Seeding Cell Count: Number of cells seeded in 384-well plate well. Cell Count: Counted nuclei. SOX17/SOX2/BRA: biomarker cell intensity statistics for median, mean, standard deviation (std), median absolute deviation (MAD), percent biomarker positive cells in well.

f. **Tab 6: DMSO Tolerance Test.** High content imaging data for RUES2-GLR DMSO and SB431542 tolerance testing with DevTox GLR-Endo assay. Treatment: DMSO and SB431542 exposures with definitive endoderm induction; Conc. DMSO (%)/ SB431542 (µM): Percentage DMSO and SB4311542 concentration. Cell Count: Counted nuclei. SOX17/SOX2/BRA: biomarker cell intensity statistics for median, mean, percent biomarker positive cells in well.
g. **Tab 7: Reference chemical set testing.** High content imaging data for RUES2-GLR reference chemical set with DevTox GLR-Endo assay. Bio Rep: Biological replicate number (5 – 12). Treatment: PluriControl – Pluripotent; DMSOControl – 0.2% DMSO exposure with definite endoderm induction; 1 µM SB431542 exposure with definitive endoderm induction; DEControl – definitive endoderm induction; Reference chemicals exposure with definitive endoderm induction. Seeding Cell Count: Number of cells seeded in 384-well plate well. Cell Count: Counted nuclei. SOX17/SOX2/BRA: biomarker cell intensity statistics for median, mean, standard deviation (std), median absolute deviation (MAD), percent biomarker positive cells in well.

4. AEID3093_L6_CCTE_Deisenroth_DEVTOX_RUES2-GLR_Sox17_up_210917.pdf – TCPL curve plots for AEID3093.
5. AEID3094_L6_CCTE_Deisenroth_DEVTOX_RUES2-GLR_Sox17_dn_210917.pdf – TCPL curve plots for AEID3094.
6. AEID3095_L6_CCTE_Deisenroth_DEVTOX_RUES2-GLR_Sox2_up_210917.pdf – TCPL curve plots for AEID3095.
7. AEID3096_L6_CCTE_Deisenroth_DEVTOX_RUES2-GLR_Bra_up_210917.pdf – TCPL curve plots for AEID3096.
8. AEID3097_L6_CCTE_Deisenroth_DEVTOX_RUES2-GLR_CellCount_up_210917.pdf – TCPL curve plots for AEID3097.
9. AEID3098_L6_CCTE_Deisenroth_DEVTOX_RUES2-GLR_CellCount_dn_210917.pdf – TCPL curve plots for AEID3098.
